# FRMD6 determines the cell fate towards senescence: involvement of the Hippo-YAP-CCN3 axis

**DOI:** 10.1038/s41418-024-01333-2

**Published:** 2024-06-26

**Authors:** Jung-Jin Park, Su Jin Lee, Minwoo Baek, Ok-Jun Lee, Seungyoon Nam, Jaehong Kim, Jin Young Kim, Eun-Young Shin, Eung-Gook Kim

**Affiliations:** 1 Department of Biochemistry, Chungbuk National University, College of Medicine and Medical Research Center, Cheongju, 28644 Republic of Korea; 2 Department of Pathology, Chungbuk National University, College of Medicine and Medical Research Center, Cheongju, 28644 Republic of Korea; 3 Department of Health Sciences and Technology, Gachon Advanced Institute for Health Sciences and Technology (GAIHST), Gachon University, Incheon, 21565 Republic of Korea; 4 Department of Biochemistry, College of Medicine, Gachon University, Incheon, 21999 Republic of Korea; 5 Research Center for Bioconvergence Analysis, Korea Basic Science Institute, Cheongju, 28119 Republic of Korea

**Keywords:** Ageing, Respiratory tract diseases

## Abstract

Cellular senescence, a hallmark of aging, is pathogenically linked to the development of aging-related diseases. This study demonstrates that FRMD6, an upstream component of the Hippo/YAP signaling cascade, is a key regulator of senescence. Proteomic analysis revealed that FRMD6 is upregulated in senescent IMR90 fibroblasts under various senescence-inducing conditions. Silencing FRMD6 mitigated the senescence of IMR90 cells, suggesting its requirement in senescence. Conversely, the overexpression of FRMD6 alone induced senescence in cells and in lung tissue, establishing a causal link. The elevated FRMD6 levels correlated well with increased levels of the inhibitory phosphorylated YAP/TAZ. We identified cellular communication network factor 3 (CCN3), a key component of the senescence-associated secretory phenotype regulated by YAP, whose administration attenuated FRMD6-induced senescence in a dose-dependent manner. Mechanistically, FRMD6 interacted with and activated MST kinase, which led to YAP/TAZ inactivation. The expression of FRMD6 was regulated by the p53 and SMAD transcription factors in senescent cells. Accordingly, the expression of FRMD6 was upregulated by TGF-β treatment that activates those transcription factors. In TGF-β-treated IMR90 cells, FRMD6 mainly segregated with p21, a senescence marker, but rarely segregated with α-SMA, a myofibroblast marker, which suggests that FRMD6 has a role in directing cells towards senescence. Similarly, in TGF-β-enriched environments, such as fibroblastic foci (FF) from patients with idiopathic pulmonary fibrosis, FRMD6 co-localized with p16 in FF lining cells, while it was rarely detected in α-SMA-positive myofibroblasts that are abundant in FF. In sum, this study identifies FRMD6 as a novel regulator of senescence and elucidates the contribution of the FRMD6-Hippo/YAP-CCN3 axis to senescence.

## Introduction

Senescence is a cellular state characterized by cell cycle arrest and the onset of various features, including the senescence-associated secretory phenotype (SASP) [1]. As a hallmark of aging, senescence is widely accepted as a pathogenic mechanism in aging-related diseases, such as pulmonary fibrosis [2, 3]. Consequently, strategies targeting senescence have been the subject of extensive research.

The Hippo signaling pathway plays a crucial role in a wide range of cellular processes, including cell plasticity, cell cycle, epithelial-to-mesenchymal transition and migration [4, 5]. Activation of the Hippo core kinases, MST and LATS, negatively regulates the YAP/TAZ-mediated transcription. YAP/TAZ also responds to mechanical stresses such as tissue stiffness, playing a central role in mechanotransduction [6]. Tissue stiffness decreases with age due to the degradation of extracellular matrix proteins, which results in reduced YAP/TAZ activity. Recent studies have highlighted the role of this decreased YAP/TAZ activity in accelerating organismal aging and the induction of senescence [7, 8]. Despite significant efforts, the intracellular upstream regulators of the Hippo signaling pathway remain poorly understood.

FERM domain-containing protein 6 (FRMD6), also known as Willin, is a member of the 4.1 ezrin-radixin-moesin (FERM) domain-containing protein family [9]. The FERM domain (aa 14-322) mediates interactions with transmembrane proteins, which allows FRMD6 to regulate cell polarity and cell-cell adhesion in epithelial cells [9, 10]. FRMD6 expression activates the Hippo signaling pathway kinases, MST and LATS, and antagonizes oncogenic YAP [11]. Consistent with this role, FRMD6 exerts a tumor suppressive effect on most human cancers, including prostate cancer [12]. Merlin (mammalian neurofibromin 2 (NF2)) and KIBRA (mammalian WWC1), two other FERM domain-containing proteins, also function upstream of Hippo signaling through direct interaction with and the activation of LATS [13–16]. Merlin and KIBRA have been shown to form a complex with Expanded (Ex), a Drosophila ortholog of mammalian FRMD6 [17]. However, mammalian FRMD6 does not appear to interact with NF2 or WWC1, which suggests that FRMD6 may not be a mammalian ortholog of Ex [18]. Collectively, FRMD6, NF2 and WWC1 act as upstream components of the Hippo signaling pathway, but their distinct activation mechanisms and functions remain unclear.

Given that FRMD6 inhibits YAP activity [11] and that decreased YAP activity has been linked to senescence [7, 8], it seems plausible that FRMD6 expression may be causally linked to senescence. Therefore, this study aimed to elucidate the biological and mechanistic roles of FRMD6 in senescence. Taken together, our findings support the role of FRMD6 in determining the senescent cell fate through the Hippo-YAP-CCN3 axis.

## Results

### Identification of FRMD6 as a putative senescence regulator

**Fig. 1:**
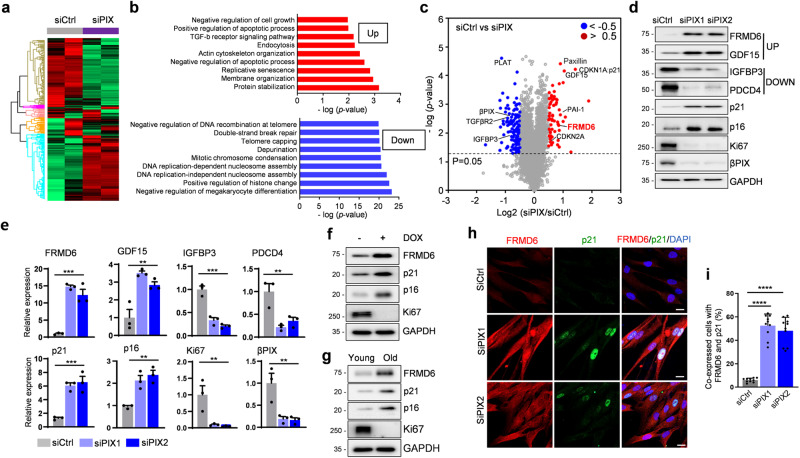
Upregulation of FRMD6 in senescent cells. **a**–**c** Proteomics analysis. **a** Heatmap of differentially expressed proteins (*p* < 0.05) in control vs. siPIX-treated IMR90 cells. ANOVA test. *p* < 0.05. **b** Pathway analysis. **c** Volcano plot of differentially expressed proteins. A Log2 fold change of >0.5 or <−0.5 (*p* < 0.05) was regarded as significant. Red, upregulated proteins; blue, downregulated proteins; gray, no differentially expressed proteins. **d**–**g** Immunoblotting analysis. Lysates from siPIX-treated (**d**) and doxorubicin (DOX)-treated (**f**), and replicative senescent (**g**) cells were subjected to immunoblotting for the indicated proteins. **e** Quantification of immunoblotting shown in (**d**). Error bars indicate means ± SEM from three independent experiments. **p < 0.01, ***p < 0.0001, one-way ANOVA. **h** Co-staining images for FRMD6 and p21. Control and siPIX-treated cells were subjected to immunofluorescence. **i** Quantification of FRMD6-and p21-positive cells shown in (**h**). Scale bar, 20 μm. Error bars indicate means ± SEM from three independent experiments. ****p < 0.0001, *t*-test.

Based on our previous study, which demonstrated that silencing beta p21-activated kinase interacting exchange factor (βPIX), a focal adhesion protein, triggers cellular senescence [19], we sought to identify a novel senescence regulator. To this end, we analyzed previous data from tandem mass tag (TMT)-labeling proteomic analysis [20] and found 4643 differentially expressed proteins following βPIX depletion (Fig. 1a and Table S1). Gene ontology enrichment analysis revealed altered expression patterns in diverse pathways linked to βPIX function, including cell growth, TGF-β signaling, endocytosis and actin cytoskeleton regulation (Fig. 1b). Notably, the replicative senescence pathway ranked highly among the upregulated pathways in βPIX-depleted cells (Fig. 1b, top), while nucleosome assembly and DNA repair pathways were downregulated (Fig. 1b, bottom). Volcano plot analysis further highlighted changes in FRMD6 and known senescence-related proteins, such as p16, p21, plasminogen activator inhibitor-1 (PAI-1), growth differentiation factor 15 (GDF15), and insulin-like growth factor binding protein 3 (IGFBP3) (Fig. 1c). Among those, we focused on FRMD6 due to its potential role upstream of the senescence-associated Hippo/YAP signaling pathway [7, 8, 11]. To characterize the role of FRMD6, we first assessed its expression in senescent cells. Cellular senescence was monitored by upregulation of p21 and p16, and downregulation of Ki67, a proliferation marker [21]. FRMD6 levels were elevated in βPIX-depleted senescent cells, as were the known senescence markers, including GDF15 (Fig. 1d; quantified in e). Conversely, the expression of IGFBP3 and programmed cell death 4 (PDCD4) was downregulated. Similar increases in FRMD6 were observed in doxorubicin (DOX)-induced and replicative senescent cells (Fig. 1f, g). While NF2 and WWC1 are known upstream regulators of the Hippo signaling pathway [13, 16], their potential role in senescence has been unclear. We also investigated their expression in senescent cells and found that, unlike FRMD6, levels of both NF2 and WWC1 decreased (Fig. S1a–c). However, knocking down NF2 or WWC1 had no impact on either senescence or FRMD6 levels (Fig. S1d, e). These results suggest that NF2 and WWC1 may have distinct functions independent of senescence.

To further explore the relationship between FRMD6 and senescence, we performed immunofluorescence staining. FRMD6 immunoreactivity was elevated in senescent cells compared to control cells (Fig. 1h; quantified in i). Notably, FRMD6 and nuclear p21 co-localized in approximately 50% of βPIX-depleted senescent cells (Fig. 1i) and in 75% of DOX-treated and replicative senescent cells (Fig. S2). To assess the functional relevance of FRMD6 in senescence, we knocked down FRMD6 using siRNA. FRMD6 knockdown significantly reduced SA-β-Gal positivity to ~10% in βPIX-depleted cells (Fig. S3a–c), accompanied by decreased levels of p16 and p21. In contrast, Ki67 levels increased in this condition (Fig. S3d; quantified in e). These effects were consistent across βPIX-depleted, DOX-treated and replicative senescent cells (Fig. S3f–i). These findings demonstrate that FRMD6 is required for the induction of senescence in three different model systems, suggesting its critical role in that process.

We further examined FRMD6 expression in two types of senescent epithelial cells. Cultured human small airway epithelial cells and rat alveolar epithelial (RLE-6TN) cells displayed a similar upregulation of FRMD6 in DOX-treated senescent cells, suggesting the generality of that expression pattern at least in fibroblast and epithelial cells (Fig. S4).

### Overexpression of FRMD6 induces senescence in IMR90 fibroblasts and in the lung

**Fig. 2:**
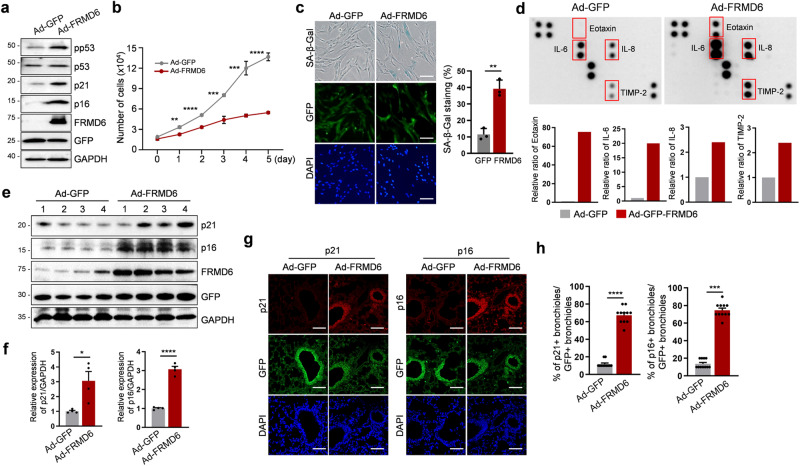
Overexpression of FRMD6 induces senescence in vitro and in vivo. **a** Immunoblotting of senescence markers. **b** Cell proliferation assay. Cells infected with Ad-GFP (GFP only) or Ad-FRMD6 expressing GFP as a reporter were counted over 5 days of culture. **c** SA-β-Gal staining and quantification. SA-β-Gal staining was performed (left) and quantified for SA-β-Gal positive cells (right). Scale bars, 50 μm. **d** Cytokine array analysis. Infected cells were cultured for 5 days and culture media were collected and incubated with the membranes for cytokine array (top). Densitometry was performed for quantification (bottom). **e** Immunoblotting analysis. Lysates from Ad-GFP and Ad-FRMD6-infected lungs were subjected to immunoblotting. *N* = 4 mice per group. **f** Quantification of the blot in (**e**). **g** Immunofluorescence images for p16 and p21. Scale bar, 100 μm. **h** Quantification of p16 or p21-positive bronchioles in GFP-positive bronchioles shown in (**g**). *N* = 11 mice per group. Error bars indicate means ± SEM. *p < 0.05, **p < 0.01, ***p < 0.001, *t*-test.

Based on the implication of the potential role of FRMD6 in senescence, we investigated whether the overexpression of FRMD6 alone could induce senescence in IMR90 fibroblasts. Adenoviral infection-mediated overexpression of FRMD6 increased levels of cell cycle proteins p21 and p16, along with phosphorylated p53 (Fig. 2a). Consistent with these observations, cells overexpressing FRMD6 displayed a decreased proliferative potential (Fig. 2b). Moreover, increased SA-β-Gal activity confirmed the induction of senescence (Fig. 2c). One of the hallmarks of cellular senescence is that SASP induces the production and secretion of senescence-associated factors. In order to identify the factors secreted, we employed a cytokine array. That analysis revealed elevated levels of eotaxin, IL-6, IL-8 and TIMP-2 in FRMD6-overexpressing cells (Fig. 2d). Collectively, these findings support the role of FRMD6 to induce senescence in IMR90 fibroblasts.

We further examined the in vivo effect of FRMD6 overexpression in the lung. Utilizing the intranasal route, we introduced adenovirus expressing GFP or FRMD6. Immunoblotting revealed a significant upregulation of p21 and p16 in response to FRMD6 overexpression (Fig. 2e; quantified in f). Similarly, immunofluorescence staining demonstrated a marked increase in p21 and p16 in Ad-FRMD6-infected bronchial epithelial cells compared to Ad-GFP-infected cells (Fig. 2g; quantified in h). Collectively, these results highlight the senescence-inducing role of FRMD6.

### Involvement of the Hippo/YAP signaling pathway in FRMD6-induced senescence

**Fig. 3:**
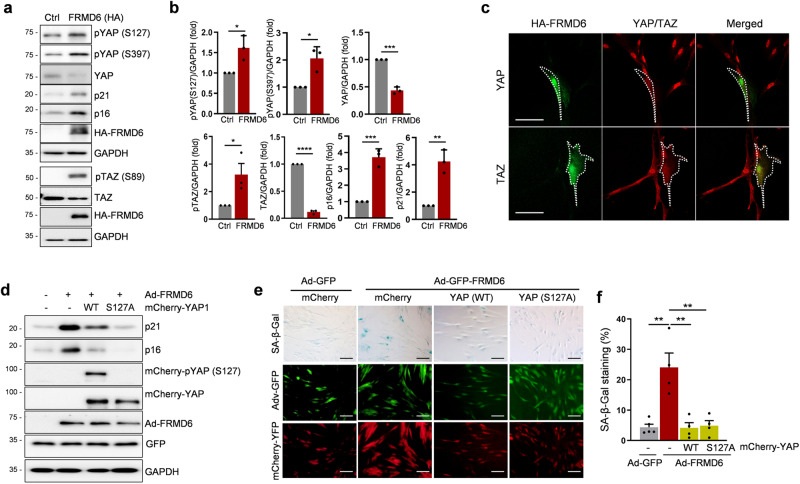
Constitutively active YAP rescues FRMD6-induced senescence. **a** Immunoblotting for the senescence markers and YAP/TAZ. Cells were transfected with plasmids for control or HA-tagged FRMD6 and lysates were subjected to immunoblotting. **b** Quantification of the blots shown in (**a**). Error bars indicate means ± SEM from three independent experiments. *p < 0.05, **p < 0.01, ***p < 0.001, *t*-test. **c** Immunofluorescence images of FRMD6 (HA-tagged) and endogenous YAP or TAZ using separate antibodies for YAP or TAZ. Cells were transfected with a plasmid encoding HA-FRMD6 and were co-stained with a combination of antibodies against HA/YAP or HA/TAZ. Note that the strong FRMD6-positive cell shows negligible YAP or TAZ staining. Scale bar, 50 μm. Immunoblotting (**d**) and SA-β-Gal staining (**e**). Cells were transfected with plasmids expressing mCherry-YAP, wild-type or a constitutively active form (S127A), followed by infection with Ad-FRMD6, after which immunoblotting and SA-β-Gal staining assays were conducted. Scale bar, 50 μm. **f** Quantification of SA-β-Gal positive cells shown in (**e**). *N* ≥ 200 cells per group from three independent experiments. Error bars indicate means ± SEM. **p < 0.01, *t*-test.

The established role of FRMD6 as an upstream activator of the Hippo signaling pathway prompted us to investigate whether the overexpression of FRMD6 affects Hippo/YAP signaling in IMR90 cells. Under FRMD6-induced senescent conditions as monitored by the upregulation of p16 and p21, YAP/TAZ had a significantly increased phosphorylation (Fig. 3a; quantified in b). In accordance, a marked decrease in total YAP levels was observed. We further investigated the status of YAP/TAZ in three additional senescence models: βPIX-depleted cells (Fig. S5a; quantified in b), replicative senescent cells (Fig. S5d; quantified in e) and DOX-treated cells (Fig. S5g; quantified in h). Consistent with the FRMD6-induced senescent condition, all three of those models showed increased phosphorylation and decreased levels of YAP/TAZ. Immunofluorescence images revealed more intense FRMD6 staining in senescent cells compared to control semi-confluent cells that displayed a nuclear localization of YAP (Fig. S5c, f, i). Notably, nuclear YAP was nearly undetectable in senescent cells. Immunofluorescence staining provided further insights into the relationship between FRMD6 and YAP. Semi-confluent cells overexpressing FRMD6 displayed weak nuclear staining of YAP/TAZ, indicating their inverse correlation (Fig. 3c). Next, we investigated if FRMD6 induces senescence in confluent cells, similar to its effect in semi-confluent cells. Cells were cultured at high density and infected with Ad-FRMD6. As expected, YAP localization was primarily cytoplasmic in both infected (yellow asterisk) and non-infected (white asterisk) cells, mirroring the quiescent state observed previously [22] (Fig. S6a). This cytoplasmic YAP localization made it challenging to distinguish between FRMD6-infected, potentially senescent cells, and non-infected, quiescent cells. To address this, we performed Western blotting to analyze protein levels of senescence markers along with YAP and pYAP (Fig. S6b). Notably, even under confluent conditions, Ad-FRMD6 infection resulted in increased levels of p21, p16, and pYAP/YAP compared to control cells, suggesting that FRMD6 functions similarly in both semi-confluent and confluent conditions.

These results strongly implicated the Hippo/YAP signaling pathway in the FRMD6-induced senescence. To further validate this, we conducted a rescue experiment by co-expressing FRMD6 with active YAP (S127A), which is resistant to proteolytic degradation. While cells overexpressing FRMD6 alone displayed clear senescence features, including elevated p21 and p16 levels (Fig. 3d, 2nd column) and increased SA-β-Gal activity (Fig. 3e, 2nd column; quantified in 3f), co-expression of either wild-type (WT) or active YAP (S127A) significantly attenuated these senescent features. This was evidenced by decreased p21 and p16 expression (Fig. 3d, 3rd and 4th columns) and reduced SA-β-Gal activity (Fig. 3e, 3rd and 4th columns). Collectively, these findings strongly suggest that the Hippo/YAP axis plays a crucial role in mediating FRMD6-induced senescence.

### Identification of *CCN3* as a YAP target gene that mediates FRMD6-induced senescence

**Fig. 4:**
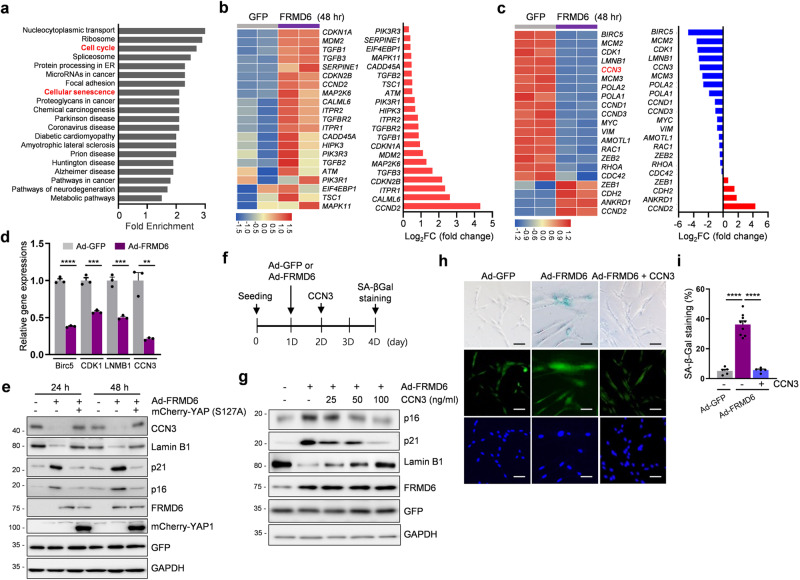
Identification of FRMD6-YAP/TAZ targets by RNA-seq analysis. **a** KEGG pathway analysis in cells overexpressing FRMD6. **b** Heatmap of increased senescence-related genes in cells overexpressing FRMD6 (left) and Log2 fold change for genes (right). **c** Heatmap of up- or down-regulated genes in the transcriptional targets of YAP (left) and Log2 fold change for genes (right). **d** Validation of downregulated genes by qRT-PCR. Error bars indicate means ± SEM. *N* = 3, **p < 0.01, ***p < 0.001, ****p < 0.0001, *t*-test. **e** Immunoblotting. **f** Experimental scheme for the rescue experiment. **g** Immunoblotting for senescence-related proteins. **h** SA-β-Gal staining. **i** Quantification of SA-β-Gal positive cells shown in (**h**). *N* ≥ 200 cells per group from three independent experiments. Error bars indicate means ± SEM. ****p < 0.0001, *t*-test.

To identify potential YAP target genes that contribute to the senescence pathway regulated by the FRMD6-YAP axis, we performed an unbiased RNA-seq analysis. Cells were infected with either Ad-GFP (control) or Ad-FRMD6 for 48 h, after which their RNA was extracted and analyzed. KEGG analysis revealed 5769 genes with altered expression levels between control and FRMD6-overexpressing cells, including genes involved in ‘cell cycle’ and ‘cellular senescence’ (Fig. 4a and Table S2). Notably, several known senescence-related genes were identified, including *CDKN1A* (p21), *CDKN2A* (p16), *CCND2* (cyclin D2) and *SerpinE1* (PAI-1) (Fig. 4b and Table S3), confirming the senescence-inducing role of FRMD6. Next, we focused on YAP target genes whose expression was altered in a FRMD6-dependent manner. Among the downregulated genes, *BIRC5* (baculoviral inhibitor of apoptosis repeat-containing 5), *MCM2* (minichromosome maintenance 2), *CDK1* (cyclin-dependent kinase 1), *LMNB1* (lamin B1) and *CCN3* (cellular communication network factor 3) ranked in the top 5 (Fig. 4c and Table S4). We validated the downregulation of *BIRC5*, *CDK1*, *LMNB1* and *CCN3* using qRT-PCR (Fig. 4d). Interestingly, *CCND1*, *ANKRD1* and *CDH2* were among the top 3 upregulated genes. This seemingly contradictory result suggests that some YAP target genes might be upregulated even when YAP activity is inhibited.

Considering the role of decreased YAP activity in inducing senescence, we focused on downregulated genes, particularly CCN3, which is secreted as a SASP factor. We confirmed that FRMD6 induced the downregulation of CCN3 at both the mRNA and protein levels (Fig. 4d, e). Importantly, this downregulation was rescued by constitutively active YAP, confirming CCN3 as a genuine YAP target. A rescue experiment further demonstrated the functional role of CCN3 in FRMD6-induced senescence. As shown schematically in Fig. 4f, increasing concentrations of CCN3 were added one day after Ad-FRMD6 infection, which inhibited the upregulation of p16, p21, and SA-β-gal activity induced by FRMD6 (Fig. 4g–i). We further examined a senescence-rescuing role of CCN3 by ectopic expression of CCN3 as shown in the scheme (Fig. S7a). Intracellular CCN3 showed similar results as incubation with soluble CCN3 (Fig. S7b, c). Overall, our findings identify CCN3 as a YAP target gene that plays a key role in FRMD6-induced senescence. Lamin B1, another potential YAP target, showed a similar expression pattern like CCN3 downstream of FRMD6. We therefore examined its involvement in FRMD6-induced senescence. Ectopically expressed lamin B1 also showed an inhibitory effect on FRMD6-induced senescence (Fig. S7d, e), suggesting involvement of multiple YAP targets.

### FRMD6 binds and activates MST kinase

**Fig. 5:**
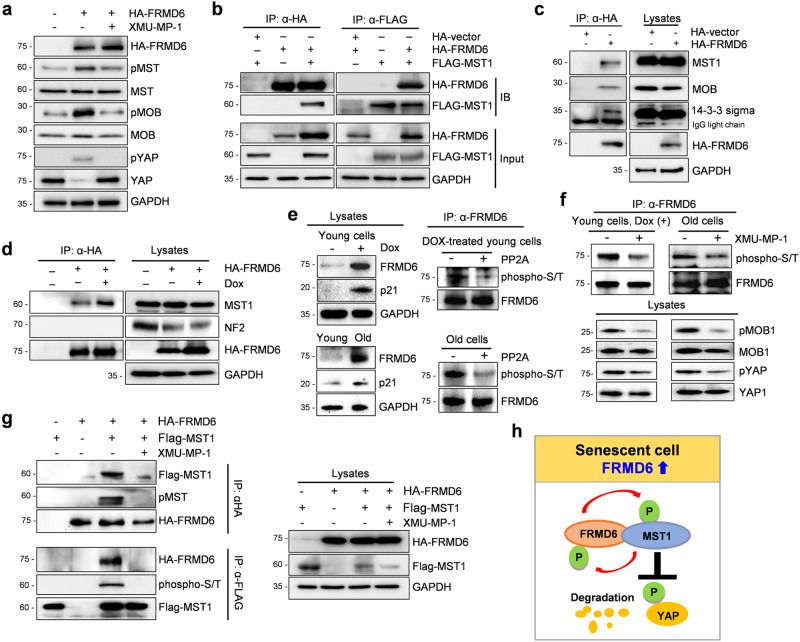
Identification of MST kinase as a binding partner of FRMD6. **a**–**g** Immunoblotting analysis. **a** IMR90 cells were transfected with a plasmid expressing HA-tagged FRMD6 and incubated with XMU-MP-1 (10 μM) for 2 h prior to harvest. Lysates were subjected to immunoblotting. **b** Cells were co-transfected with plasmids and lysates were immunoprecipitated using anti-HA or anti-FLAG antibody-coated Agarose beads followed by immunoblotting. **c** Cells were transfected with the indicated plasmids and lysates were subjected to immunoprecipitation with anti-HA beads followed by immunoblotting. **d** Cells transfected with the indicated plasmid were stimulated with DOX for 48 h. Lysates were immunoprecipitated by anti-HA beads and immunoblotted. **e** Left, lysates from DOX-treated young cells (top) or young and old cells (bottom) were immunoblotted. Right, lysates from DOX-treated young cells (top) or from old cells (bottom) were immunoprecipitated by an anti-FRMD6 antibody followed by the treatment with PP2A and immunoblotting. **f** DOX-treated young cells (left) and old cells (right) were incubated with XMU-MP-1 (10 μM) for 2 h prior to harvest. Lysates were then immunoprecipitated by an anti-FRMD6 antibody and immunoblotted. **g** Cells were co-transfected with plasmids and treated with XMU-MP-1 as described above. Lysates were immunoprecipitated with anti-HA or anti-FLAG beads followed by immunoblotting. GAPDH was used as a loading control. **h** Model for the interaction of FRMD6 and MST kinase, which leads to the inactivation of YAP.

FRMD6 is known to activate the Hippo signaling pathway, but the precise mechanism involved was elusive. To unravel it, we performed a series of experiments using IMR90 cells. First, we confirmed the role of FRMD6 in MST activation. Overexpressing FRMD6 increased the level of phosphorylated MST (pMST) (Fig. 5a) and inhibiting MST with XMU-MP-1 blocked the FRMD6-induced phosphorylation of MST and its downstream effectors, MOB and YAP (Fig. 5a). This suggested that MST lies directly downstream of FRMD6. Next, we investigated their physical interaction. For this purpose, we co-transfected IMR90 cells with plasmids encoding HA-tagged FRMD6 and FLAG-tagged MST1 and performed an immunoprecipitation experiment. Co-immunoprecipitation analyses revealed that FRMD6 pulled down MST1, and vice-versa (Fig. 5b). When expressed alone, HA-tagged FRMD6 could capture endogenous MST1 and MOB along with 14-3-3 sigma, a previously identified protein [18] (Fig. 5c). We next examined the interaction between FRMD6 and MST in DOX-treated senescent cells. Compared to control cells, DOX-induced senescence strengthened the FRMD6-MST1 interaction (Fig. 5d; compare the 2nd and 3rd lanes in the left panel). However, no such connection was observed between FRMD6 and NF2 (Fig. 5d). This enhanced interaction hinted at an MST-induced phosphorylation of FRMD6 in senescent cells. We therefore examined the phosphorylation status of FRMD6 in DOX-treated and in replicative senescent cells, where FRMD6 levels were elevated as shown in Fig. 1 (Fig. 5e, left). Treatment with PP2A, a phosphatase, reduced phosphorylated FRMD6 (Fig. 5e, right). Given their close interaction, we hypothesized that MST might phosphorylate FRMD6. Indeed, treatment with XMU-MP-1 significantly decreased levels of phosphorylated FRMD6 (Fig. 5f), implicating MST in this process. Finally, we investigated the impact of phosphorylation on their interaction. When co-expressed, FRMD6 and MST1 interacted strongly, but the presence of XMU-MP-1 nearly abolished that interaction (Fig. 5g). Together, the upregulation of FRMD6 in senescent cells promotes its interaction with and activation of MST (shown schematically in Fig. 5h). In turn, activated MST phosphorylates FRMD6, potentially solidifying their interaction and ultimately leading to YAP inactivation (Fig. 5h).

### TGF-β upregulates FRMD6 in a p53- and SMAD-dependent manner

**Fig. 6:**
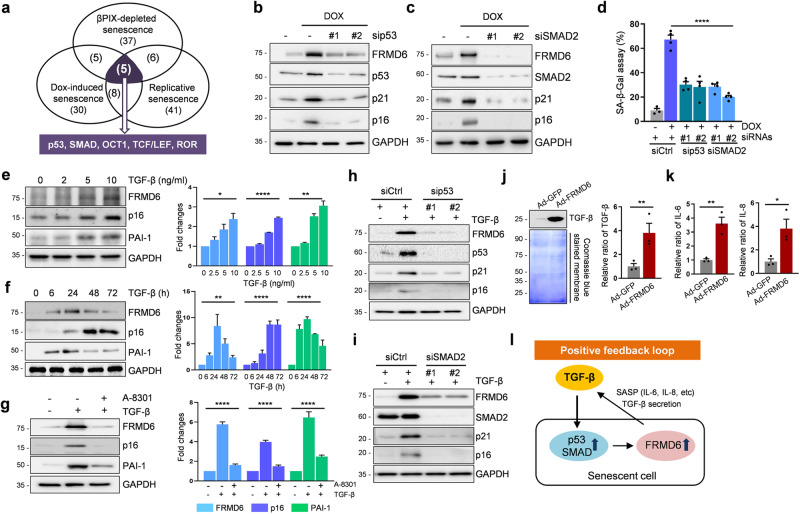
TGF-β upregulates FRMD6 expression in IMR90 fibroblasts. **a** Venn diagram for activated transcription factors. **b**, **c** Immunoblotting. Cells were treated with DOX (500 nM) in the presence of control siRNAs, p53- (**b**) or SMAD2-specific siRNAs (**c**). Representative blots are shown from two independent experiments. **d** Quantification of SA-β-Gal staining. *N* ≥ 200 cells per group from three independent experiments were counted. ****p < 0.0001, *t*-test. **e**–**g** Immunoblotting (left) and quantification (right). Dose- (**e**) and time-dependent (**f**) changes in FRMD6 along with senescence markers in response to TGF-β. **g** Effect of treatment with A-8301, a TGF-β receptor I inhibitor. **h**, **i** Immunoblotting. Cells were treated with TGF-β (10 ng/ml) in the presence of control siRNAs, p53- (**h**) or SMAD2-specific siRNAs (**i**). Representative blots are shown from three independent experiments. **j** Immunoblotting (left) and ELISA (right) of secreted TGF-β. Culture medium was collected at 2 days post Ad-GFP (control) and Ad-FRMD6 infection and subjected to immunoblotting and ELISA. As a loading control, the membrane was stained with Coomassie Brilliant Blue. *N* = 3, Error bars indicate means ± SEM. **p = 0.0028, *t*-test **k** ELISA. IL-6 (left) and IL-8 (right) were quantified by ELISA using the same culture medium from (**j**). *N* = 3, Error bars indicate means ± SEM. *p = 0.0285, **p = 0.0048, *t*-test. **l** Model for the positive feedback loop via upregulated FRMD6 in TGF-β-induced senescence.

While senescent cells exhibit elevated FRMD6 levels, the underlying regulatory mechanism was unknown. To identify potential transcription factors (TFs) that regulate FRMD6 expression, we employed a “TF activation profiling plate array” during senescence induction (Fig. S8). That analysis revealed five common TFs that were activated across three distinct senescence conditions: p53, SMAD, Oct1, TCF/Lef and ROR (Fig. 6a). Focusing on p53 and SMAD, we observed that silencing them in DOX-treated cells suppressed p21 and p16 expression, indicating their role as potential regulators (Fig. 6b, c). Furthermore, the depletion of those TFs diminished DOX-induced increases in SA-β-gal staining, confirming their involvement in the progression of senescence (Fig. 6d). Considering the broad regulatory role of TGF-β in cellular functions, including senescence [23], we explored its potential role in FRMD6 expression. TGF-β signaling activates SMAD2/3, which forms complexes with SMAD4 to activate SMAD-dependent genes. Notably, p53 interacts with SMAD, synergistically driving PAI-1 transcription in response to TGF-β [24]. Consistent with this, TGF-β induced the senescence of IMR90 cells, with SA-β-gal-positive cells reaching ~18% of the population (Fig. S9a, b). Moreover, TGF-β treatment upregulated FRMD6 expression in a dose- and time-dependent manner (Fig. 6e, f; quantification in their right panels). Treatment with A-8301, a TGF-β receptor I inhibitor, reduced TGF-β-induced upregulation of FRMD6 and senescence markers to control levels, confirming TGF-β as a FRMD6 regulator (Fig. 6g; quantification in right panel). The depletion of p53 and SMAD2 in TGF-β induced senescent cells dramatically suppressed FRMD6, p21 and p16 expression as well as DOX-treated cells (Fig. 6h, i). Interestingly, the overexpression of FRMD6 led to increased TGF-β secretion, confirmed by immunoblotting and ELISA on TGF-β in the culture medium (Fig. 6j). We were also able to detect IL-6 and IL-8 in the same medium using an ELISA (Fig. 6k), which is consistent with the cytokine array data (Fig. 2d). These findings suggest a positive feedback loop wherein TGF-β upregulates FRMD6, which contributes to senescence and stimulates TGF-β secretion as a SASP factor (shown schematically in Fig. 6l).

### FRMD6 determines the senescent cell fate in TGF-β-stimulated cells

**Fig. 7:**
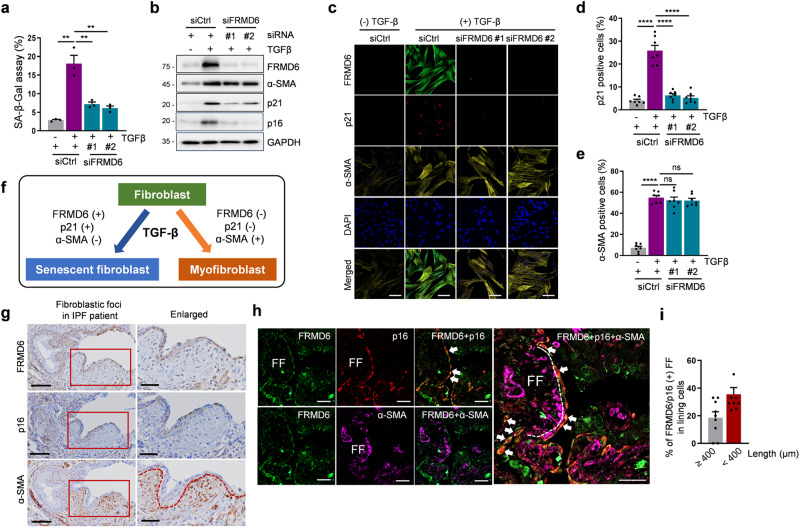
FRMD6 mediates TGF-β-induced senescence but not myofibroblast differentiation. **a**–**e** FRMD6 was knocked down and its effect was evaluated in TGF-β-stimulated cells. **a** Quantification of SA-β-Gal staining. **b** Immunoblotting analysis. **c** Immunofluorescence. Triple staining for FRMD6, p21 and α-SMA was conducted in control and FRMD6 knockdown cells after stimulation with TGF-β (10 ng/ml). Quantification of p21 (**d**) and α-SMA (**e**) positive cells in control or FRMD6 siRNA-treated cells. Error bars indicate means ± SEM from three independent experiments. ****p < 0.0001, *t*-test. **f** Schematic model for a specific role of FRMD6 in TGF-β-induced senescence but not myofibroblast differentiation. **g** Representative immunohistochemical images. Serially sectioned lung tissues from IPF patients were subjected to immunohistochemistry with the indicated antibodies. The rectangular areas in red (left column) are enlarged in the right column. Scale bars, 50 μm (left column); 100 μm (right column). **h** Representative Immunofluorescence images. Human IPF lung tissues were co-stained with FRMD6, p16, and α-SMA. FF, fibroblastic foci. Dotted lines denote the border between FF and lining cells that face the cystic space. Scale bars, 50 μm. **i** Quantification of FRMD6 and p16 co-staining FF in lining cells (shown in **h**). The size of FF denotes the long diagonal length in μm. *N* = 9 IPF patients.

TGF-β, a multifunctional cytokine, can induce both senescence and myofibroblast differentiation, but those events rarely occur simultaneously within the same cell [25]. Therefore, we investigated the specific role of FRMD6 in cell fate determination. Knocking down FRMD6 reduced the number of SA-β-gal-positive senescent cells to ~6-7% of the population (Fig. 7a). It also suppressed the TGF-β-induced rise in p21 and p16 levels in IMR90 (Fig. 7b). However, α-SMA levels were not affected, which suggested the specific involvement of FRMD6 in the senescence pathway but not in myofibroblast differentiation. To confirm this, we conducted triple staining for FRMD6, p21 and α-SMA. As illustrated in Fig. 7c, TGF-β treatment upregulated FRMD6, which mostly overlapped with nuclear p21 in control cells. FRMD6 knockdown drastically reduced p21 levels, whereas α-SMA expression remained unaffected (Fig. 7c; quantified in d, e). These results established FRMD6 as a critical determinant of TGF-β-induced senescence in IMR90 cells (Fig. 7f).

Next, we examined FRMD6 expression in a fibrotic environment mimicking the TGF-β-treated culture conditions. Fibrotic areas in patients with idiopathic pulmonary fibrosis (IPF) often contain fibroblastic foci (FF). FF are rich in α-SMA-positive myofibroblasts and extracellular matrix, while some lining cells exhibit senescent phenotypes [26, 27]. As expected, p16-positive cells were frequently found in the FF lining cells, while α-SMA was strongly positive in FF but was seldom detectable in the lining cells (Fig. 7g). FRMD6 was highly expressed in the lining cells, overlapping with p16 but not with α-SMA (Fig. 7g). Further analysis by triple immunofluorescence revealed the co-expression of FRMD6 and p16 but not α-SMA in the FF lining cells (Fig. 7h, white arrows). We quantified the prevalence of FF exhibiting co-localization of FRMD6 and p16 within the lining cell population, mirroring the pattern observed in Fig. 7h. Similar FF constituted 19% of the total foci with a long diagonal length greater than 400 μm and 36% of the total foci with a long diagonal length less than 400 μm (Fig. 7i). Interestingly, some FRMD6-positive cells were p16 negative, implying potential roles for FRMD6 beyond senescence. Analogous to the in vitro condition (Fig. 7c), FRMD6 and p16 were occasionally detected in FF that are marked by a strong reactivity of α-SMA (Fig. 7h), implying the presence of senescent myofibroblasts. Collectively, these findings suggest that FRMD6 may mediate TGF-β-induced senescence both in cultured cells and in fibrotic conditions, implicating its potential role in disease pathogenesis.

## Discussion

In the present study, we identified a novel regulator of senescence, FRMD6, whose expression is upregulated in diverse types of senescent conditions: replicative, βPIX depletion- and drug-induced senescence. The overexpression of FRMD6 functions as a causative factor that induces senescence in vitro and in vivo. Our results provide mechanistic insights into FRMD6-induced senescence by uncovering its downstream Hippo-YAP-CCN3 axis as well as its transcriptional regulation involving the p53 and SMAD complex.

Senescent cells exhibited an upregulated expression of FRMD6 but not of NF2 or WWC1, suggesting that a different regulatory mechanism controls their expression. Multiple TFs are involved in regulating the senescence programs. Cell cycle arrest converges on p53 and Rb, while NF-κB and C/EBPβ play a central role in SASP. Our findings uncovered an unexpected role for p53 that elevates the expression of FRMD6 leading to the inhibition of YAP activity. This crosstalk of p53 with YAP may ensure cell cycle arrest by downregulating G1/S components of YAP targets such as CDK1, MCM2/3, POLA1/A2 and cyclin D1/D3 (Fig. 4c). Considering that TGF-β activates p53 but inhibits YAP, this may also explain the inhibitory effect of TGF-β on cell proliferation. SMAD is a well-known TF that is involved in canonical TGF-β signaling and also functions as an upstream regulator of FRMD6, which suggests that TGF-β signaling is active in senescent cells. Indeed, FRMD6-induced SASP included TGF-β as well as IL-6 and IL-8 (Fig. 6). Thus, the TGF-β-p53-FRMD6 axis may form a positive feedback loop in senescent cells (Fig. 6i).

TGF-β is a pleiotropic agonist that regulates a wide range of biological functions, in particular promoting the differentiation of fibroblasts to myofibroblasts, a major pathogenic cell type involved in fibrotic diseases. Studies have also demonstrated a senescence-inducing role for TGF-β [23]. Together, TGF-β is likely to control myofibroblast differentiation and senescence in a mutually exclusive manner because those two cell fates are not concurrently activated in the same cell [25]. What then is the determinant(s) for TGF-β-induced senescence? We propose that FRMD6 is a major determinant in that event based on our findings. First, TGF-β upregulated FRMD6 expression, but the knockdown of FRMD6 did not affect TGF-β-induced myofibroblast differentiation as assessed by the expression levels of α-SMA in Western blot analysis (Fig. 7). Second, in the triple immunofluorescence analysis of TGF-β-induced events, FRMD6 co-localized with p21 but rarely with α-SMA. Importantly, the knockdown of FRMD6 resulted in few p21-positive cells concomitant with the disappearance of FRMD6, but a slight increase in the number of α-SMA-positive cells. In support of these in vitro data, the immunofluorescence of IPF lung tissues revealed the co-localization of FRMD6 with p16 in cells lining FF but not with α-SMA inside FF.

It has long been recognized that FRMD6 functions upstream of the Hippo signaling pathway, but the detailed mechanism has been unknown. Earlier studies have revealed 14-3-3 proteins as binding partners of FRMD6 [18]. Considering the role of 14-3-3 proteins, this interaction may sequester or degrade FRMD6. It has also been generally accepted that FRMD6 functions as a tumor suppressor gene. Surprisingly, FRMD6 bound and activated mTOR in lung cancer, suggesting its tumor promoting role [28]. However, those binding studies did not resolve the question linking FRMD6 to Hippo signaling. We addressed this issue by hypothesizing that if FRMD6 activates Hippo signaling, FRMD6 may bind and activate MST kinase and indeed, our binding study unveiled their interaction. Moreover, we found that activated MST in turn phosphorylates FRMD6, highlighting their reciprocal interaction. It is tempting to speculate that the MST-mediated phosphorylation of FRMD6 may enhance its stability as in the case of phosphorylated Sav, a MST substrate [29]. Alternatively, analogous to the phosphorylation of ERM proteins that confers an active open conformation [30], phosphorylated FRMD6 may have different biological activities such as cytoskeletal rearrangement as in neuronal cells [31, 32]. FRMD6 may also function independent of the canonical Hippo-YAP/TAZ axis [12, 33]. The activation of MST kinase not only inhibits YAP-mediated cellular activity but also performs other biological functions including apoptosis. The MST1-FOXO3 pathway has been extensively studied regarding MST-dependent apoptosis [34]. This pathway is particularly relevant in neuronal apoptosis [35]. Related to this, our RNA-seq data analyses revealed a notable enrichment of the pathways involved in neurodegeneration and in neurodegenerative diseases (Fig. 4a).

Both RASSF1A and FRMD6 act as tumor suppressors by binding to MST kinase and activating the Hippo pathway, ultimately reducing YAP activity, a key driver of tumorigenesis. Mechanistically, RASSF1A promotes apoptosis through pro-apoptotic gene expression [36] and may also induce differentiation in cancer stem cells by separating YAP from the β-catenin-TCF complex [37]. FRMD6, on the other hand, induces senescence, a state of cell cycle arrest that hinders tumor cell proliferation. Although they seem to influence distinct YAP functions, they likely cooperate to suppress tumor development. However, in advanced cancers with active TGF-β signaling, the tumor suppressor role of these proteins can be subverted. RASSF1A gets recruited to the TGF-β receptor I and degraded via the ITCH E3 ligase, effectively eliminating its tumor-suppressive activity and potentially promoting tumor invasion [38]. Conversely, our study highlighted the upregulation of FRMD6 in response to active TGF-β signaling, leading to senescence. Senescent cells can indirectly promote tumor progression by secreting senescence-associated factors such as growth factors and inflammatory cytokines. Therefore, in advanced cancers, the loss of RASSF1A and the upregulation of FRMD6 might act synergistically to create a complex tumor microenvironment that facilitates tumor progression.

Previous studies demonstrated that decreased YAP activity is closely linked to senescence [7, 8]. Those studies identified CDK6 and lamin B1 as critical YAP targets whose downregulation mediates senescence. In agreement with those findings, our data also identified lamin B1 as a mediator of FRMD6-induced senescence. Whereas, instead of CDK6, CDK1 emerged as a YAP target. Considering the role of CDK1 [39], our findings suggest that the cell cycle is blocked at the earlier phase of G1/S in FRMD6-induced senescence. We further identified CCN3, a secreted factor, as a novel YAP target which may affect senescence in a paracrine manner. More importantly, CCN3 rescued FRMD6-induced senescence in a dose-dependent manner, highlighting an anti-senescence activity. In contrast, evidence indicates the senescence-promoting role of CCN3 in trophoblast cells and chondrocytes, thus contributing to the pathogenesis of placental disease preeclampsia and the degeneration of articular cartilage, respectively [40–42]. This discrepancy may reflect the cell type- or context-dependent functions of CCN3. Further study is warranted to delineate the detailed mechanism underlying the anti-senescence effect of CCN3, either directly through its specific signaling pathways or indirectly through its cross-talk with other CCN proteins such as CCN1 and CCN2 [43, 44].

In summary, this study demonstrated that FRMD6 plays a key role in determining the fate of senescent cells. Considering the impact of cellular senescence in a wide range of aging-related diseases, targeting FRMD6 and its signaling may offer a new avenue for therapeutic interventions.

## Materials and methods

### Materials

Antibodies were purchased from Cell Signaling Technology (Danvers, MA, USA), Novus Biologicals (Centennial, CO, USA), BD Sciences (San Jose, CA, USA), Abcam (Cambridge, UK) and Santa Cruz Biotechnology (Santa Cruz, CA, USA); detailed information is provided in the Supplementary Materials. Lipofectamine 3000, RNAiMAX, Invivofectamine 3.0, fetal bovine serum (FBS), F12 and Opti-Minimum Essential Medium (MEM) were purchased from Thermo Fisher Scientific, Inc. (Waltham, MA, USA). ELISA Kits were obtained from Abcam. The TF activation profiling plate array kit was purchased from Signosis, Inc (Santa Clara, CA, USA). A-8301 was purchased from Sigma Aldrich (Saint Louis, MO, USA). Adeno viruses were prepared by Komabiotech (Seoul, Korea). HA-FRMD6 and FLAG-MST1 plasmids were purchased from Addgene (Watertown, MA, USA) and Sino Biological Inc. (Beijing, China), respectively. PCR primers and siRNAs were obtained from Bioneer (Daejeon, Korea) and Thermo Fisher Scientific, Inc. and a list of their sequences is provided in Supplementary Materials. Detailed information regarding the materials used in this study is provided in the Supplementary Materials.

### Methods

#### Cell culture and cellular senescence

IMR90 fibroblasts were obtained from Coriell institute (Camden, NJ, USA) and were cultured in MEM supplemented with 10% FBS and antibiotics in a humidified 5% CO_2_ incubator at 37 °C. To induce cellular senescence, cells were treated with 400 nM doxorubicin (Sigma-Adrich Inc., St. Louis, MO, USA) for 2 h, then washed and incubated with fresh culture medium for 2–3 days.

#### Animal experiments and adenoviruses infection

Six-week-old C57BL/6J male mice (Daehan Bio Link, Chungbuk, Korea) were used. Mice were randomly divided into two experimental groups; an intranasal infection with adenovirus (Ad)-GFP group and with Ad-FRMD6 group. Animal experiments were performed in accordance with approved animal protocols and guidelines established by the Ethics Review Committee of the Chungbuk National University for Animal Experiments (CBNUA-2202-233-02).

#### Cell proliferation assay

The cell proliferation assay was performed as previously described [19]. After culture in 6-well plates and transfection using Ad-GFP or Ad-FRMD6, harvesting of IMR90 cells using trypsin and staining with crystal violet solution was performed at each time point. A hemocytometer was used to count the number of cells.

#### SA-β-Gal staining

Cells were fixed with 2% formaldehyde and 0.2% glutaraldehyde for 10 min. Following fixation, cells were incubated in SA-β-Gal staining solution (1 mg/ml 5-bromo-4-chloro-3-indolyl-beta-d-galactopyranoside (X-gal), citric acid/sodium phosphate buffer (pH 6.0), 5 mM potassium ferricyanide, 5 mM potassium ferrocyanide, 150 mM NaCl and 2 mM MgCl_2_) at 37 °C for 20–24 h. To confirm total cell numbers, cells were incubated in 0.5 μg/ml DAPI solution for 10 min and then washed with Phosphate-buffered saline (PBS). Cellular senescence was scored as the ratio of SA-β-gal-positive cells (blue stained) relative to the total cell number.

#### Immunohistochemistry

Tissues were fixed with formalin and embedded in paraffin. Tissue sections were cut at a thickness of 4 μm, then deparaffinized and processed for antigen retrieval in 10 μg/ml proteinase K solution or 10 mM sodium citrate buffer (pH 6.0). After antigen retrieval, slides were incubated in universal blocking buffer [(1%BSA, 0.1% cold fish skin gelatin, 0.5% Triton X-100, 0.05% sodium azide, 0.01 M PBS (pH 7.4)] for 1 h. The slides were then incubated in the primary antibody solution overnight at 4 °C and washed three times with PBS-T (PBS containing 0.1% Tween-20). For diaminobenzidine-HCl (DAB) staining, slides were treated in methanol with 0.3% hydrogen peroxide for 20 min at room temperature (RT), then with a biotin-conjugated secondary antibody for 1 h at RT, and with peroxidase-conjugated streptavidin for 30 min at RT. Finally, signals were detected using the substrate DAB. After DAB staining, stained signals were observed under a microscope and were analyzed using ImageJ (Plugins, Colour deconvolution) and GraphPad Prism (Version 8.0) software.

#### qRT-PCR

Total cellular RNA was isolated from IMR90 cells with TRIzol reagent (Invitrogen) following the manufacturer’s instructions. Extracted RNA was quantified using a Thermo Fisher Scientific nano drop. cDNAs were synthesized using a LAVO pass cDNA synthesis kit (iNtRON, Sungnam, Korea) according to the manufacturer’s instructions. CFX Manager software (Bio-Rad, Hercules, CA, USA) was used for the setup of the experiment and analysis of the data. All values were normalized to those of actin.

#### Cytokine array

The conditioned medium from Ad-GFP infected IMR90 cells and from Ad-FRMD6 infected IMR90 cells was used for ELISA and cytokine arrays. ELISA and cytokine arrays were performed using a Human IL-6 ELISA kit (Abcam, ab178013), a Human IL-8 ELISA kit (Abcam, ab214030), a Human TGF-β ELISA kit (Abcam, ab100647) and a Human cytokine array kit (Abcam, ab211059) according to the manufacturer’s instructions. In the cytokine array, signal intensity was detected using a ChemiDoc imaging system (Bio-Rad Laboratories, Inc.).

#### Immunoprecipitation and Western blotting

Cells were lysed and extracted in RIPA buffer [50 mM Tris, pH 8.0; 150 mM NaCl; 1% Nonidet P-40; 0.5% sodium deoxycholate; 0.1% sodium dodecyl sulfate (SDS)] containing a GenDOPOT Xpert Phosphatase inhibitor cocktail solution (Thermo Fisher Scientific, Inc.) and a GenDOPOT Xpert Protease inhibitor cocktail solution (Thermo Fisher Scientific, Inc.). For Western blotting, equal amounts of protein were resolved on SDS-polyacrylamide gels and transferred to polyvinylidene fluoride membranes. After blocking each membrane in milk in TBS-T (50 mM Tris, 150 mM NaCl, and 0.2% Tween 20) for 1 h, membranes were probed with specific primary antibodies and horseradish peroxidase (HRP)-conjugated secondary antibodies. Immunoreactive bands were detected using the enhanced chemiluminescence (ECL) system using a ChemiDoc imaging system (Bio-Rad Laboratories, Inc.). Quantification of immunoreactive dot intensities was performed using ImageJ software. For immunoprecipitations, 1 mg of each cell lysate was immunoprecipitated with the specific primary antibody for 5 h and then incubated with protein A/G agarose for 3 h. Then, immunoprecipitates were washed three times with PBS-T, resolved on SDS-PAGE gels and immunoblotted with specific antibodies as described above.

#### Immunofluorescence

Cells on cover slips were fixed with 4% paraformaldehyde for 10 min. Subsequently, the fixation buffer was aspirated and the cover slips were rinsed three times with PBS. For permeabilization, cover slips were treated with 1 ml permeabilization buffer (0.2% Triton X-100 in PBS) in each well, incubated for 10 min, and then rinsed three times with PBS. For blocking, cover slips were incubated in blocking buffer (3% BSA) for 1 h. They were then incubated in the primary antibody solution at 4 °C overnight, and washed three times with PBS. Cover slips were then incubated in secondary antibody at 4 °C for 1 h, and then washed three times with PBS. After staining, positive signals were observed under a microscope and analyzed using ImageJ and GraphPad Prism (Version 8.0) software.

#### RNA seq analysis

Total RNAs were prepared from three biological replicates of IMR90 cells infected with Ad-GFP (control) or Ad-FRMD6 for 48 h. The libraries were prepared for 151 bp paired-end sequencing using a TruSeq stranded mRNA Sample Preparation Kit (Illumina, San Diego, CA, USA). Specifically, mRNAs were purified and fragmented from 1 μg of each total RNA using oligo (dT) magnetic beads. The process of preparing double-stranded cDNA from fragmented mRNA involved using random hexamer priming to synthesize single-stranded cDNA. This was then used as a template for second strand synthesis to create double-stranded cDNA. Following this, end repair, A-tailing, and adapter ligation were carried out in a sequential process to prepare cDNA libraries. These libraries were then amplified using PCR (Polymerase Chain Reaction). The quality of the cDNA libraries was evaluated using the Agilent 2100 BioAnalyzer, while their quantity was determined using the KAPA library quantification kit, both according to the manufacturer’s protocols. Subsequently, cluster amplification of denatured templates was performed, and sequencing was carried out as paired-end (2 × 151 bp) using the Illumina NovaSeq6000 platform.

#### Transcription factor assay

Each array assay was performed following the procedure described in the user manual for the TF activation profiling plate array kit (FA-1006, Signosis, Inc.). Briefly, 10 µg of each nuclear extract was prepared and incubated with a biotin-labeled probe mix at RT for 0.5 h. After protein/DNA complexes were isolated from unbound probes, the bound probes were eluted and hybridized in a plate pre-coated with captured oligos. The captured biotin-labeled probes were detected using streptavidin–HRP and were subsequently measured using a chemiluminescent plate reader.

#### DNA and siRNA transfection

IMR90 cells were transfected with plasmid DNAs or siRNAs using the Lipofectamine 3000 or RNAiMAX (Thermo Fisher), respectively, following the manufacturer’s instructions. After 2 or 3 days, cells were subjected to SA-β-Gal assays or Western blotting.

### Proteomic analysis

To analyze βPIX depletion-induced senescence, these data were reanalyzed from our previous experiment [20]. Briefly, labeling of trypsin-digested peptides obtained from siPIX treated cells was performed using 10-plex TMT reagent, and peptides were analyzed with LC-MS/MS using an EASY-nLC 1200 UPLC system coupled to an Orbitrap Fusion Lumos Tribrid mass spectrometer (Thermo Fisher Scientific Inc.). Identification and quantification of MS/MS spectra was performed using Integrated Proteomics Pipeline software with the UniProt human database (released on 06-02-2020). All raw MS data files generated in this study have been deposited in the MassIVE repository with the identifier PXD036234.

### Statistical analysis

Statistical analyses were performed using GraphPad Prism 8.0 (GraphPad Software, San Diego, CA). Data are expressed as mean ± standard error of mean (SEM). An analysis of representative data from at least three independent experiments was performed. *p* < 0.05 was considered statistically significant. Assessment of statistical significance was performed using an unpaired Student’s *t* test and one-way ANOVA.

## Supplementary information

Supplementary information

Table S1

Table S2

Table S3

Table S4

Original Data

## Data Availability

The data analyzed during this study are included in this published article and the Supplementary data files. Additional supporting data are available from the corresponding authors upon reasonable request.
