# Supplementary material for: FRMD6 determines the cell fate towards senescence: involvement of the Hippo-YAP-CCN3 axis
Source: Cell Death Differ. 2024 Jun 26;31(11):1398–409. doi: 10.1038/s41418-024-01333-2 (PMC11519602; doi:10.1038/s41418-024-01333-2)
Supplement: Supplementary file 1 — Supplementary information [file 41418_2024_1333_MOESM1_ESM.pdf]

SUPPLEMENTAL INFORMATION

FRMD6 determines the cell fate towards senescence: Involvement of the Hippo-YAP-CCN3 axis

Running title: FRMD6 as a novel regulator of senescence

Jung-Jin Park[1,§], Su Jin Lee[1,§], Minwoo Baek[1], Ok-Jun Lee[2], Seungyoon Nam[3], Jaehong Kim[4], Jin Young Kim[5], Eun-Young Shin[1,*] and Eung-Gook Kim[1,*]

Department of Biochemistry[1] and Pathology[2], Chungbuk National University College of Medicine and Medical Research Center, Cheongju, 28644, Republic of Korea

[3]Department of Health Sciences and Technology, Gachon Advanced Institute for Health Sciences and Technology (GAIHST), Gachon University, Incheon, 21565, Republic of Korea

[4]Department of Biochemistry, College of Medicine, Gachon University, Incheon 21999, Republic of Korea.

[5]Research Center for Bioconvergence Analysis, Korea Basic Science Institute, Cheongju, 28119, Republic of Korea

[§]These authors contributed equally to this work.

* Co-corresponding authors:

Eung-Gook Kim, M.D., Ph.D.

Professor

Department of Biochemistry and Medical Research Center,

Chungbuk National University College of Medicine

Tel: +82 10-2088-7555; fax: +82 43-272-1603. Email: egkim@chungbuk.ac.kr

Eun-Young Shin, Ph.D.

Professor

Department of Biochemistry and Medical Research Center,

Chungbuk National University College of Medicine

Tel: +82 10-9079-3255; fax: +82 43-272-1603. Email: eyshin@chungbuk.ac.kr

Supplementary figures

Fig. S1

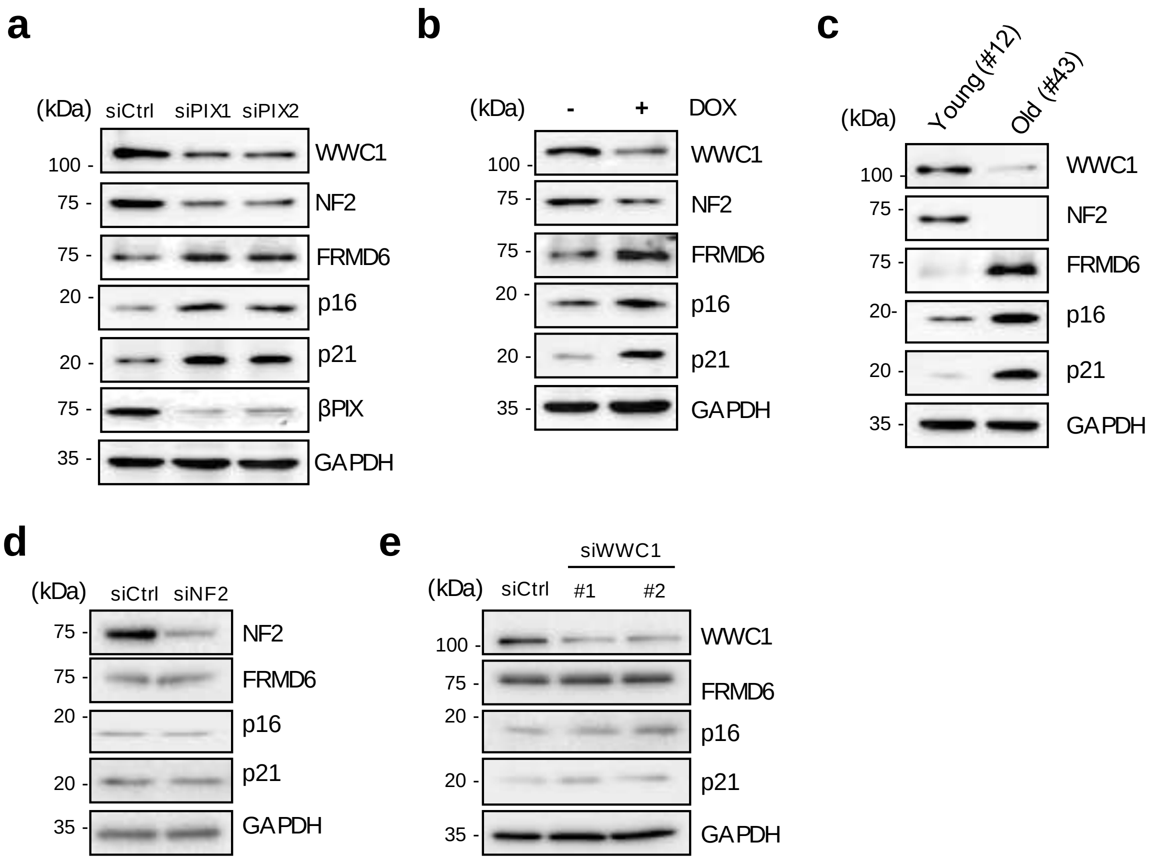

Fig. S1 Upregulation of FRMD6 but not WWC1 and NF2 in senescent cells. Lysates from cells in 3 different senescent conditions were immunoblotted for the indicated proteins. **a**, βPIX depletion-induced senescence; **b**, doxorubicin-induced senescence; **c**, replicative senescence. Senescence was monitored by the upregulation of p16 and p21. **d and e**, Lysates from siWWC1 (**d**) and siNF2 (**e**)-treated cells were immunoblotted for the indicated proteins.

Fig. S2

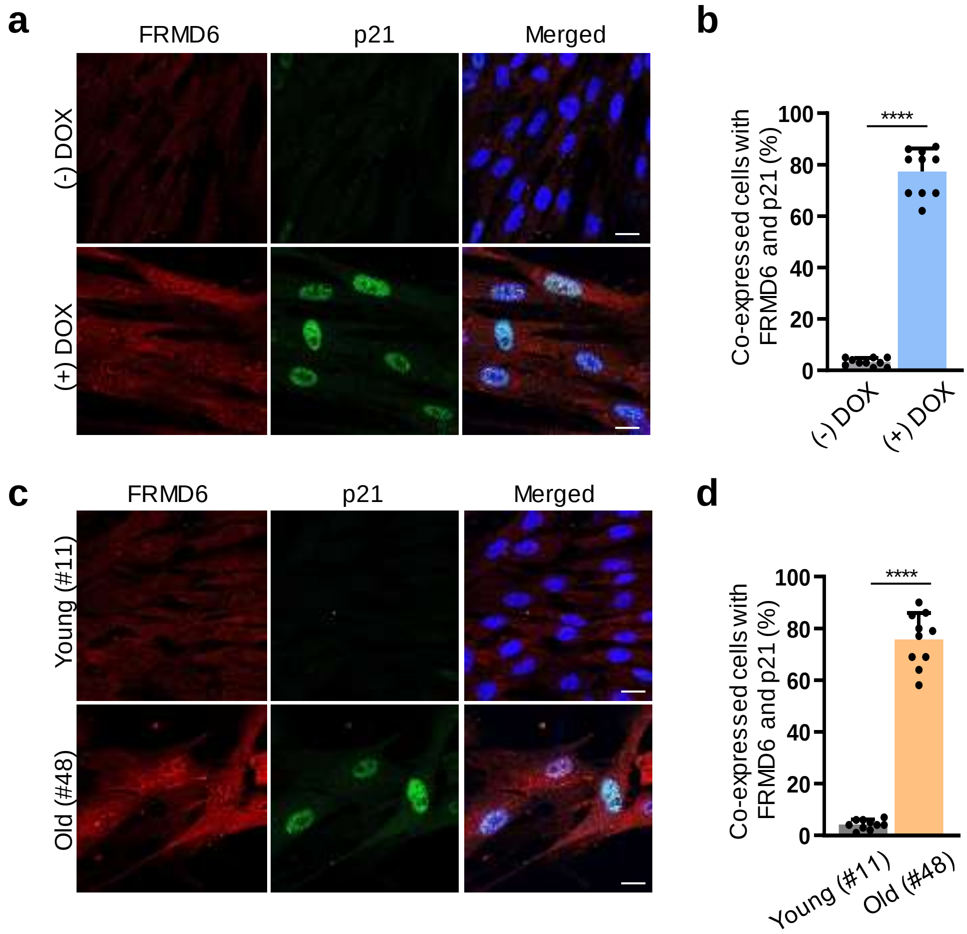

Fig. S2 Co-staining of FRMD6 and p21 in senescent cells. Immunofluorescence of FRMD6 and p21 in doxorubicin-induced senescent cells (**a**; quantified in **b**) and in replicative senescent cells (**c**; quantified in **d**) was performed. Scale bars, 20 μm. ****$p < 0.0001$, *t*-test.

Fig. S3

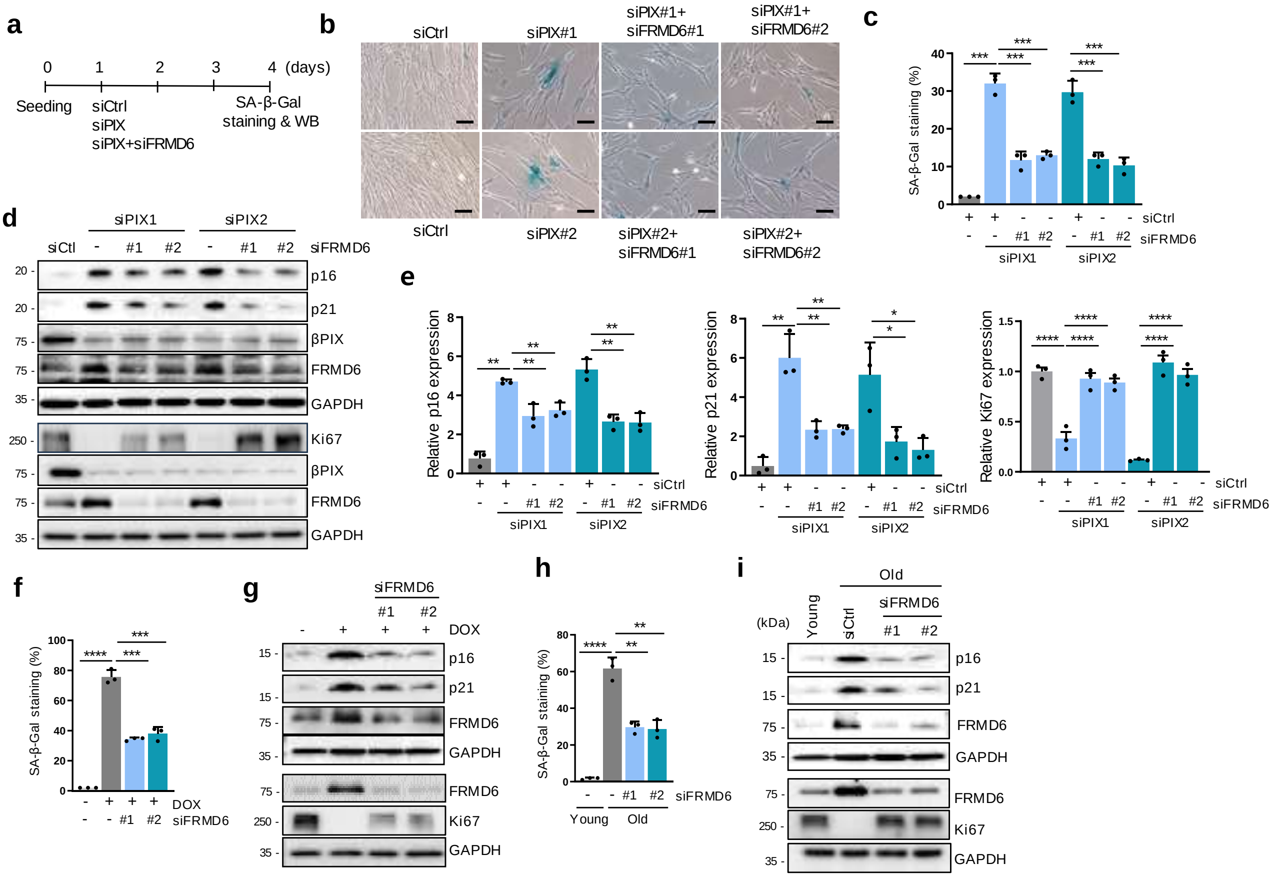

Fig. S3 Requirement of FRMD6 in 3 different types of senescence. a-e, βPIX knockdown-induced senescence. **f and g**, Doxorubicin-induced senescence, **h and i**, Replicative senescence. **a**, Experimental scheme. **b**, Representative images of SA-β-Gal staining. Scale bars, 100 μm. **c, f, h**, Quantification of SA-β-Gal positive cells. N ≥ 200 cells per group from three independent experiments. Error bars indicate means ± SEM. **p < 0.01, ***p < 0.001, ****p < 0.0001, *t*-test. **d**, Immunoblotting analysis. **e**, Quantification of the blot shown in (**d**). The expression of p16 (left), p21 (center), or Ki67 (right) was normalized to the GAPDH level. Error bars indicate means ± SEM. *p < 0.05, **p < 0.01, *t*-test. **g and i**, Immunoblotting analysis. Representative blots are shown from two independent experiments.

Fig. S4

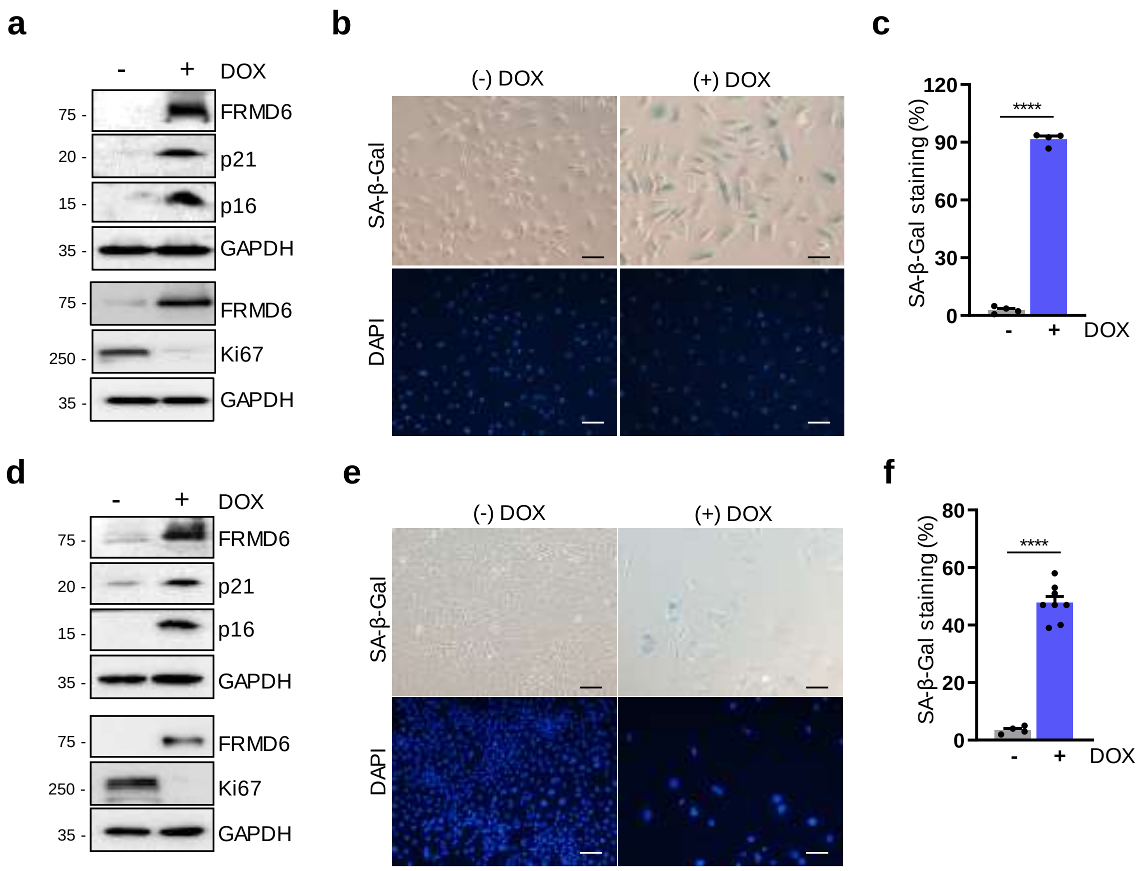

Fig. S4. Doxorubicin induces senescence in epithelial cells. Doxorubicin-treated human small airway epithelial cells (**a-c**) and rat alveolar epithelial (RLE-6TN) (**d-f**) cells. **a and d**, Immunoblotting for p16, p21, Ki67 and FRMD6. Representative blots are shown from two independent experiments. **b and e**, SA-β-Gal staining. Scale bars, 100 μm. **c and f**, Quantification of SA-βGal positive cells. N ≥ 200 cells per group from three independent experiments. Error bars indicate means ± SEM from three independent experiments. ****$p < 0.001$, *t*-test.

Fig. S5

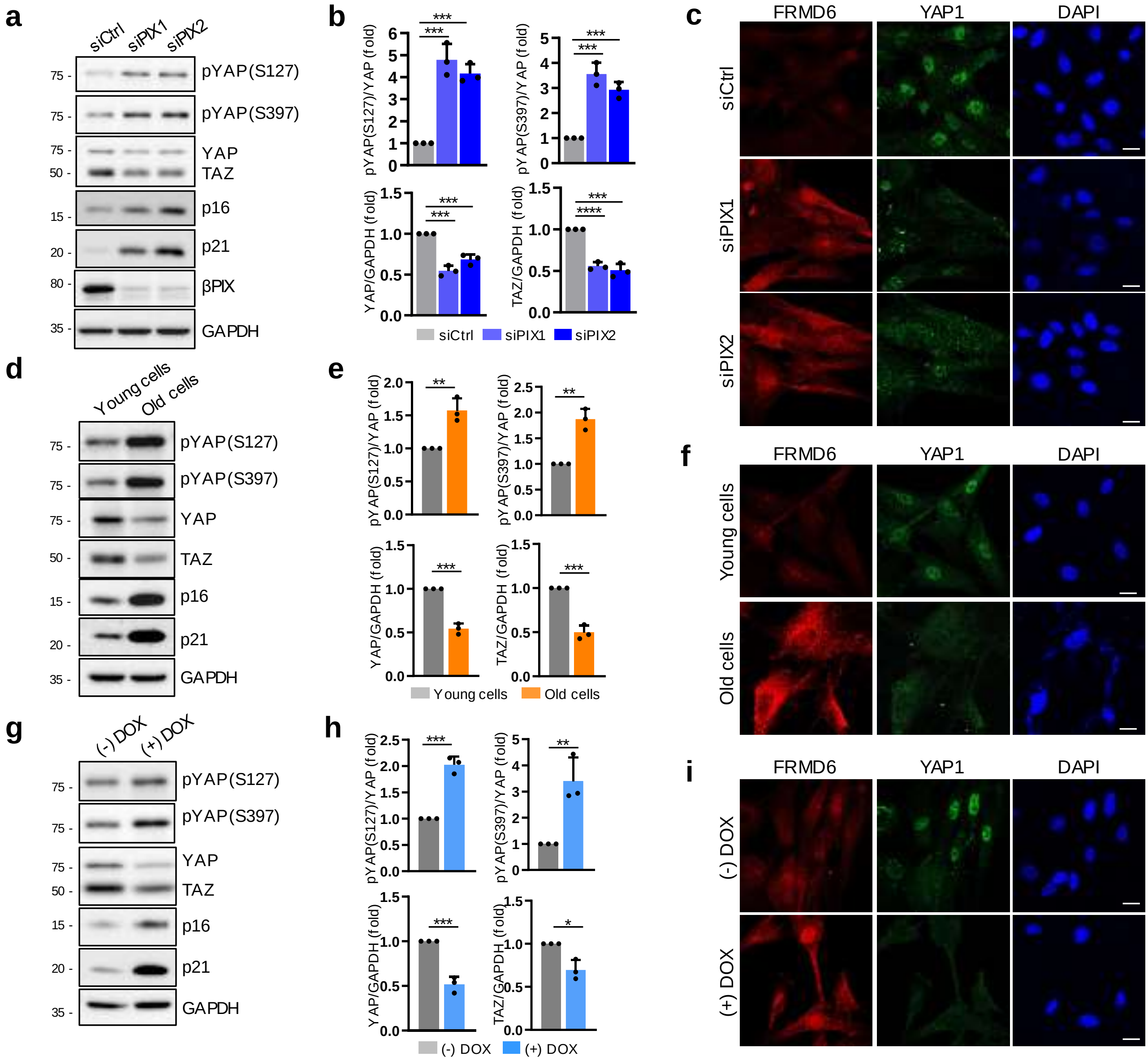

Fig. S5. Analysis of YAP/TAZ in three different senescent conditions. Immunoblot and immunofluorescence analyses were conducted in βPIX depletion-induced senescence (**a-c**), replicative senescence (**d-f**) and doxorubicin-induced senescence (**g-i**). Immunoblotting (**a, d, g**), quantification by densitometry (**b, e, h**) and immunofluorescence for FRMD6 and YAP1 (**c, f, i**) were performed. Error bars indicate means ± SEM from three independent experiments. *p < 0.05, **p < 0.01, ***p < 0.001, **** p< 0.0001, *t*-test. Scale bars, 20 μm.

Fig. S6

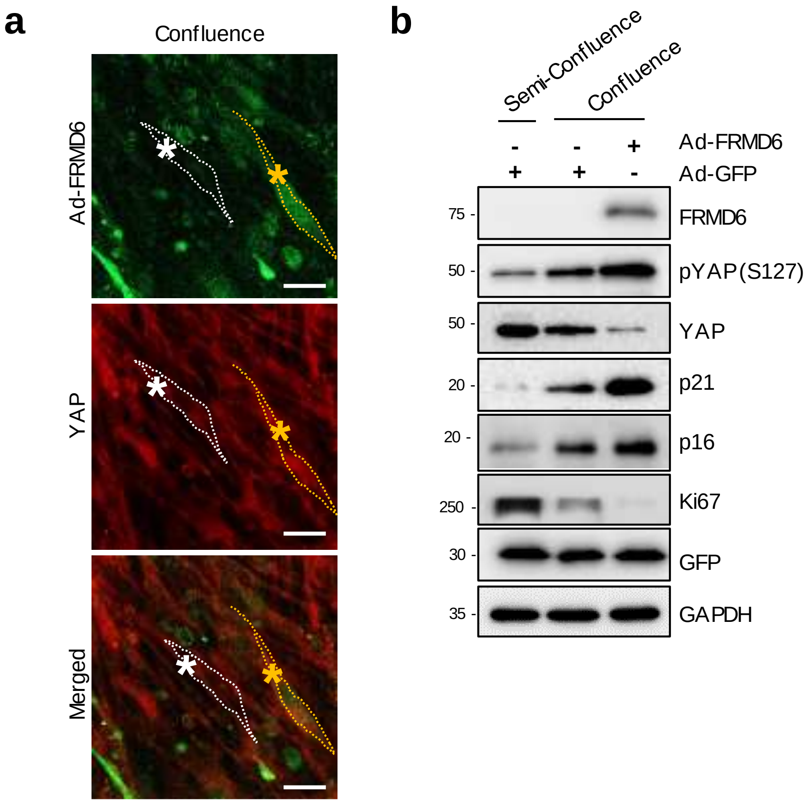

Fig. S6. FRMD6 also induces senescence in confluent IMR90 cells. a, Representative images for FRMD6 (green) and YAP (red) staining in cells from confluent culture conditions. Yellow asterisk, FRMD6-infected cell; white asterisk, non-infected cell. Scale bars, 50 μm. **b**, Immunoblotting for the senescence markers and pYAP/YAP proteins.

Fig. S7

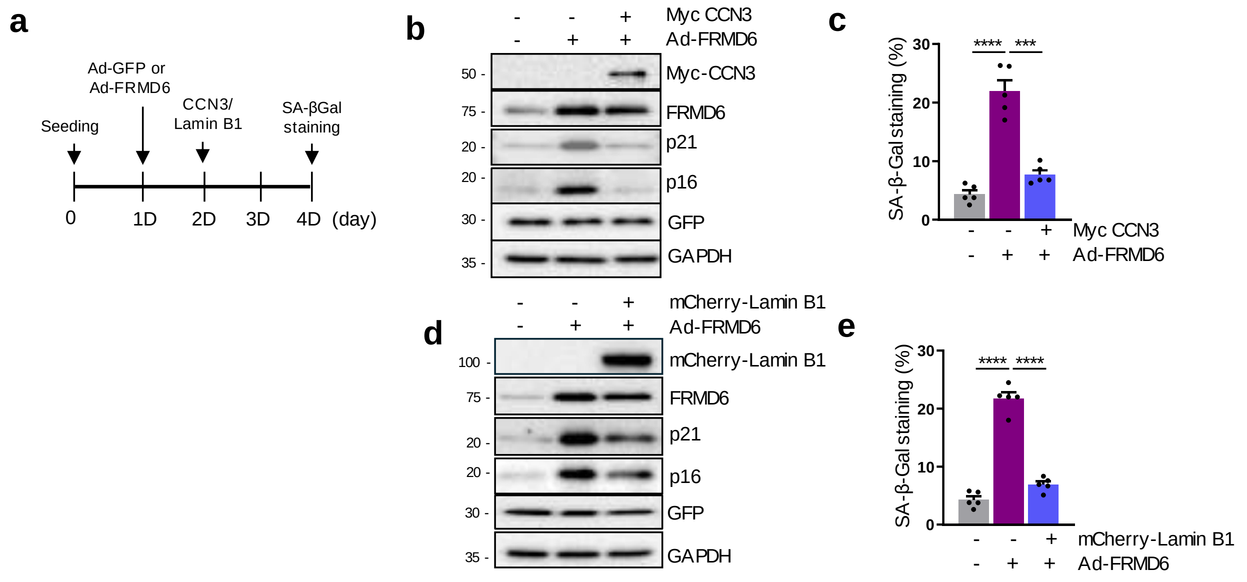

Fig. S7. Ectopically expressed CCN3 or lamin B1 also partly reverses FRMD6-induced senescence. **a**, Experimental scheme for the rescue experiment. **b, d**, Immunoblotting of senescence-related proteins in Ad-FRMD6-infected cells expressing with Myc-CCN3 (**b**) or mCherry-lamin B1 (**d**). **c, e**, Quantification of SA-β-Gal positive cells in Ad-FRMD6-infected cells with Myc-CCN3 (**c**) or mCherry-lamin B1 (**e**). N ≥ 200 cells per group from three independent experiments. Error bars indicate means ± SEM. ***p< 0.001, ****p < 0.0001, *t*-test.

Fig. S8

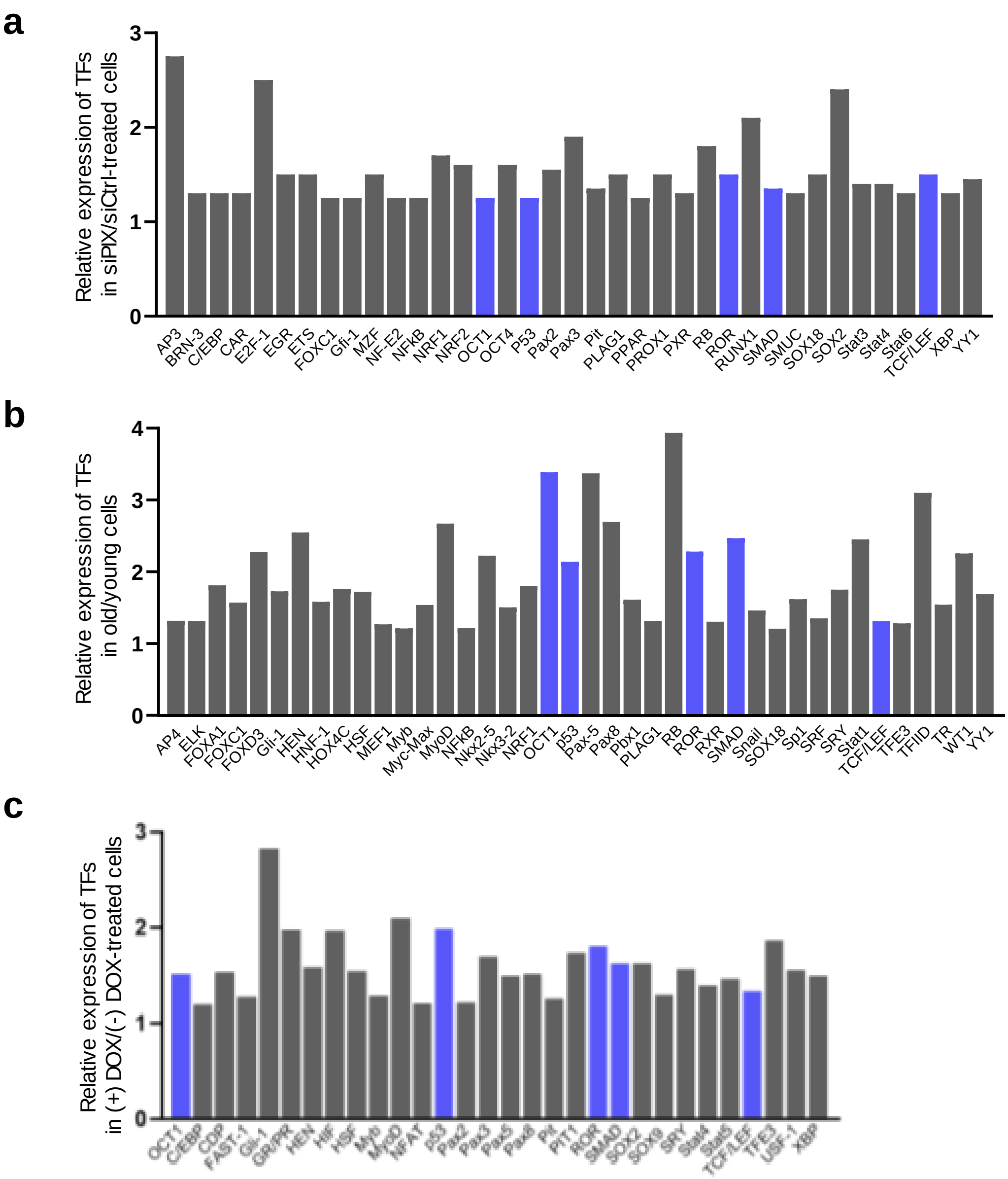

Fig. S8. Activated transcription factors (TF) in senescent cells. Analysis of TF assays was conducted as described in “Materials and Methods” in βPIX depletion-induced senescence (**a**), replicative senescence (**b**) and doxorubicin-induced senescence (**c**). The light blue bars denote shared activated TFs in the three senescent states.

Fig. S9

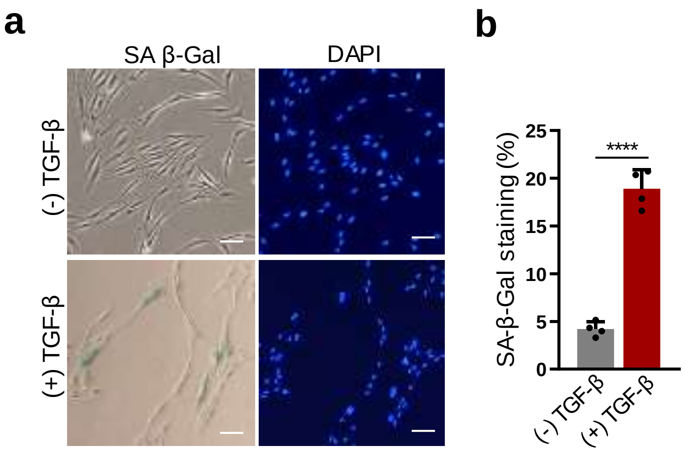

Fig. S9. TGF-β induces senescence in IMR90 fibroblasts. a, SA-β-Gal assay. SA-β-Gal staining was performed after incubation of cells with or without TGF-β. Scale bars, 50 μm. **b**, Quantification of SA-β-Gal positive cells. N ≥ 200 cells per group from three independent experiments. Error bars indicate means ± SEM from three independent experiments. ****p < 0.001, t-test.

Supplementary tables

Table S1. List of total proteins included in the heatmap in siCtrl- vs. siPIX-treated cells.

Table S2. List of differentially expressed genes in GFP vs. GFP-FRMD6 overexpressing cells.

Table S3. List of senescence-related genes in GFP vs. GFP-FRMD6 overexpressing cells.

Table S4. List of YAP target genes in GFP and GFP-FRMD6 overexpressing cells.

Supplementary materials and methods

Materials

Antibody list

Antibody	Use	Dilution	Company & Cat#
Primary Antibodies			
FRMD6	WB	1:1000	Cell Signaling Technology/#2992
	IHC	1:2000	Novus biologicals
p16	WB	1:1000	BD sciences/#554079
	IHC	1:200	
p21	WB	1:1000	Santacruze/#sc-6246
	IHC	1:200	
IGFBP3	WB	1:1000	Santacruze/#sc-365936
GDF15	WB	1:1000	Santacruze/#sc-377195
GAPDH	WB	1:5000	Invitrogen/#M45-15738

βPIX	WB	1:1000	Reference [19]
	IHC	1:400	
PDCD4	WB	1:1000	Santacruze/#sc-376430
WWC1	WB	1:1000	Cell Signaling Technology/#8774
NF2	WB	1:1000	Cell Signaling Technology/#12888
YAP	WB	1:1000	Cell Signaling Technology/#14074
	IHC	1:200	Proteintech#66900-1
$pYAP^{S127}$	WB	1:1000	Cell Signaling Technology/#13008
	IHC	1:200	
$pYAP^{S397}$	WB	1:1000	Cell Signaling Technology/#13619
TAZ	WB	1:1000	Cell Signaling Technology/#83669
	IHC	1:200	Proteintech#23306-1
$pTAZ^{S89}$	WB	1:1000	Cell Signaling Technology/#59971
p53	WB	1:1000	Cell Signaling Technology/#9282
$pp53^{S15}$	WB	1:1000	Cell Signaling Technology/#9284
phosphor-serine/threonine	WB	1:1000	Abcam#ab117253
alpha-SMA	WB	1:1000	Invitrogen#1MA1-0610
	IHC	1:1000	
alpha-SMA-Cy3	IHC	1:200	Sigma#C6198
CCN3	WB	1:1000	Cell Signaling Technology/#8767
HA	WB	1:1000	Cell Signaling Technology/#3724
SMAD2	WB	1:1000	Cell Signaling Technology/#5339
MOB1	WB	1:1000	Cell Signaling Technology/#13730
MST1	WB	1:1000	Cell Signaling Technology/#3682
SAV1	WB	1:1000	Cell Signaling Technology/#13301
Laminb1	WB	1:1000	Abcam#ab65986

Ki67	WB	1:1000	Abcam#ab15580
c-Myc	WB	1:1000	Santacruz#SC-40
Secondary Antibodies			
Anti-Mouse IgG HRP	WB	1:5000	Thermo Fisher/#31432
Anti-Rabbit IgG HRP	WB	1:5000	Thermo Fisher/#31212
Anti- Rabbit IgG Biotinylated	IHC	1:200	Vector/BA-1000
Anti- mouse IgG Biotinylated	IHC	1:200	Vector/BA-2000

PCR Primer sequence

Target	Sequence (5' → 3')
Human IL-1α FW	GGCACAAACTTTCAGAGACAGCAG
Human IL-1α RV	GTTTCTTCCTGGCTCTTGTCCTAG
Human IL-1β FW	AGGAGACTTGCCTGGTGAAA
Human IL-1β RV	GCATTTGTGGTTGGGTCAG
Human IL-6 FW	TGAGCTCGCCAGTGAAATGA
Human IL-6 RV	AGGAGCACTTCATCTGTTTAGGG
Human IL-8 FW	CATCCGGTTCGTCTACACCC
Human IL-8 RV	GGATAAACAGGGAAACACTGTGC
Human CXCL1 FW	CAGCCAGATGCAATCAATGCC
Human CXCL1 RV	TGGAATCCTGAACCCACTTCT
Human CXCL6 FW	CAGCCAGATGCAATCAATGCC
Human CXCL6 RV	TGGAATCCTGAACCCACTTCT
Human MMP3 FW	CAGCCAGATGCAATCAATGCC
Human MMP3 RV	TGGAATCCTGAACCCACTTCT
Human MMP10 FW	CAGCCAGATGCAATCAATGCC
Human MMP10 RV	TGGAATCCTGAACCCACTTCT
Human β actin FW	CATGTACGTTGCTATCCAGGC
Human β actin RV	CTCCTTAATGTCACGCACGAT
Human CCN3 FW	GGAACCGTCAATGTGAGATGCTG
Human CCN3 RV	GGCTTTGAGTGACTTCTTGGTGC
Human BIRC5 FW	CCACTGAGAACGAGCCAGACTT
Human BIRC5 RV	GTATTACAGGCGTAAGCCACCG
Human LaminB1 FW	ATGAGGACCAGGTGGAGCAGTA
Human LaminB1 RV	ACCAGGTTGCTGTTCCTCTCAG
Human CDK1 FW	GGAAACCAGGAAGCCTAGCATC
Human CDK1 RV	GGATGATTCAGTGCCATTTTGCC

siRNA sequences

siRNA (Target)	Sequence (5' → 3')
FRMD6#1	5'-GAG AGA UGA UUG CUC UGU A-3'
FRMD6#2	5'-CUC UGUAUA CCC AUU GCU U-3'
T53#1	5'-CAC UAC AAC UAC AUG UGU A-3'
T53#2	5'-GAG GUU GGC UCU GAC UGU A-3'
SMAD2#1	5'-GAC CUU CUG CUC UUC GAG U-3'
SMAD2#2	5'-CAC CAA UCA AGU CCC AUG A-3'
WWC1#1	5'-CUA AUC GCC AGA AAA GUA U-3'
WWC1#2	5'-GUG UUC UGG GUA UCC AUG U-3'

Materials and Methods

Materials

Myc-CCN3 and mcherry-lamin B1 plasmids were purchased by Sino Biological (HG-10264-CM) and Addgene (#55069), respectively. siNF and siWWC1 were obtained from Santacruz (SC-36052) and Bioneer, respectively.

Cell culture

RLE-6TN rat alveolar epithelial cells (ATCC, CRL-2300, Manassas, VA, USA) were cultured in F12 medium (Thermo Fischer Scientific, # 11765054) supplemented with 2 mM L-glutamine (Pan Biotech, P04–80100, Germany), 0.01 mg/ml bovine pituitary extract (Thermo Fischer Scientific #13028014), 0.005 mg/ml insulin (Sigma-Aldrich, # I0516), 2.5 ng/ml insulin-like growth factor (Sigma-Aldrich, # I3769), 0.00125 mg/ml transferrin (Sigma-Aldrich, # T1147), 2.5 ng/ml epidermal growth factor (Sigma-Aldrich, # E4127), 10% fetal bovine serum (heat-inactivated, PAN Biotech, P30–1506), 100 U/mL penicillin and 100 μg/mL streptomycin (PAN Biotech, P06–07100). Primary Small Airway Epithelial Cells (HSAEC) (PromoCell GmbH, Heidelberg, Germany) were cultured in Airway Epithelial Cell Basal Medium (AECBM) supplemented with Supplement-Mix. All cell lines were incubated in a humid atmosphere at 37 °C and 5% CO_2.
