# Supplementary material for: FRMD6 determines the cell fate towards senescence: involvement of the Hippo-YAP-CCN3 axis
Source: Cell Death Differ. 2024 Jun 26;31(11):1398–409. doi: 10.1038/s41418-024-01333-2 (PMC11519602; doi:10.1038/s41418-024-01333-2)
Supplement: Supplementary file 2 — Table S1 [file 41418_2024_1333_MOESM2_ESM.pdf]

Table S1. List of total proteins involved in volcano plot analysis of quantified proteins in siCtrl cells. and siPIX cell

Uniprot ID	Log2 Fold change (siPIX/siCtrl)	Log Student's T-test p-value	Gene names
Q9Y493	-1.768738747	0.608387832	ZAN
Q05D32	-1.704050541	1.594234205	CTDSPL2 HSPC058 HSPC129
Q02539	-1.201079369	1.393233269	H1-1 H1F1 HIST1H1A
P33991	-1.200072289	3.128486222	MCM4 CDC21
Q96T88	-1.188324928	2.212772116	UHRF1 ICBP90 NP95 RNF106
P49736	-1.180874825	2.859246595	MCM2 BM28 CCNL1 CDCL1 KIAA0030
Q13740	-1.141889572	1.669217497	ALCAM MEMD
P00750	-1.134943008	4.614515375	PLAT
P06454	-1.09397316	3.38036169	PTMA TMSA
P16234	-1.085287094	2.431079768	PDGFRA PDGFR2 RHEPDGFRA
P33992	-1.082644463	2.316385465	MCM5 CDC46
P16403	-1.070372581	1.936851284	H1-2 H1F2 HIST1H1C
P05204	-1.06139946	0.875096783	HMGN2 HMG17
O60216	-1.057975769	2.297995805	RAD21 HR21 KIAA0078 NXP1 SCC1
Q96CG8	-1.055996895	0.832805473	CTHRC1 UNQ762/PRO1550
Q15818	-1.007167816	2.726034461	NPTX1
P33993	-0.977548599	2.8694122	MCM7 CDC47 MCM2
P26583	-0.975671768	1.467265605	HMGB2 HMG2
P42166	-0.95720005	2.387875299	TMPO LAP2
P09429	-0.942286491	0.820810681	HMGB1 HMG1
P35580	-0.941631317	2.421897146	MYH10
Q9Y237	-0.940835953	0.591818896	PIN4
P25205	-0.935554504	2.959875862	MCM3
P16402	-0.935455322	1.520556822	H1-3 H1F3 HIST1H1D
P01889	-0.933382034	3.241179713	HLA-B HLAB
Q9Y248	-0.93110466	1.535708158	GINS2 PSF2 CGI-122 DC5 HSPC037
P37173	-0.922260284	2.522238774	TGFBR2
P32455	-0.92091465	2.267481893	GBP1
Q8IX30	-0.911137581	2.042286528	SCUBE3 CEGF3
Q14566	-0.897230148	2.38437737	MCM6
Q9UPQ9	-0.895504951	2.852366363	TNRC6B KIAA1093
P21246	-0.876507759	2.306113812	PTN HBNF1 NEGF1
Q99871	-0.861619473	2.677728676	HAUS7 UCHL5IP UIP1
O75475	-0.833770752	2.049812409	PSIP1 DFS70 LEDGF PSIP2
Q9NX05	-0.825147629	0.538183509	FAM120C CXorf17
P52292	-0.803821564	2.450414217	KPNA2 RCH1 SRP1
Q8N806	-0.803743362	2.335667979	UBR7 C14orf130
P21926	-0.803188324	1.402375049	CD9 MIC3 TSPAN29 GIG2
Q9UER7	-0.802348137	1.756652196	DAXX BING2 DAP6
P21980	-0.802144051	1.87776319	TGM2

Q14155	-0.799962997	2.558671804	ARHGEF7 COOL1 KIAA0142 P85SPR PAK3BP PIXB Nbla10314
Q9HCJ6	-0.784846306	2.334691801	VAT1L KIAA1576
Q9UBI6	-0.783507347	0.569605006	GNG12
P04439	-0.782561302	1.5206887	HLA-A HLAA
P16104	-0.778230667	0.846210075	H2AX H2AFX
Q9UHD9	-0.777501106	0.497599698	UBQLN2 N4BP4 PLIC2 HRIHFB2157
Q06945	-0.76685667	2.149063053	SOX4
P33316	-0.750356674	2.660627081	DUT
P16401	-0.749031067	1.310667731	H1-5 H1F5 HIST1H1B
Q96JJ7	-0.748718262	0.874464054	TMX3 KIAA1830 TXNDC10
Q5JVS0	-0.740912437	0.609698989	HABP4
Q9BWM7	-0.735649109	2.400839832	SFXN3
P17936	-0.735081673	1.857682121	IGFBP3 IBP3
P19320	-0.734727859	2.016619303	VCAM1
Q9H0W5	-0.731106281	2.044755733	CCDC8
O43556	-0.729112625	1.596013156	SGCE ESG UNQ433/PRO840
O95347	-0.717450142	2.446856727	SMC2 CAPE SMC2L1 PRO0324
Q99879	-0.716935158	1.445808827	H2BC14 H2BFE HIST1H2BM
Q8IWU6	-0.714920998	1.751303812	SULF1 KIAA1077
Q9NXG2	-0.714838982	2.2394238	THUMPD1
O95218	-0.706967354	2.786194921	ZRANB2 ZIS ZNF265
P45973	-0.705913544	2.092429733	CBX5 HP1A
P42167	-0.703160286	1.235048551	TMPO LAP2
P39748	-0.701975822	3.066561329	FEN1 RAD2
O14933	-0.696411133	1.933902646	UBE2L6 UBCH8
Q92629	-0.682047844	2.829643625	SGCD
P49321	-0.681056023	2.3126273	NASP
P39059	-0.67380619	3.720761398	COL15A1
P09493	-0.661658287	1.714946254	TPM1 C15orf13 TMSA
P12004	-0.661315918	2.062175775	PCNA
P80723	-0.660082817	1.755114806	BASP1 NAP22
Q9Y608	-0.657116413	2.626526749	LRRFIP2
P60903	-0.656974792	0.635217956	S100A10 ANX2LG CAL1L CLP11
O75391	-0.655556202	1.486017672	SPAG7
Q96I99	-0.654593468	2.523073641	SUCLG2
Q9H1E3	-0.654331207	1.470982287	NUCKS1 NUCKS JC7
Q99729	-0.645245552	0.861474416	HNRNPAB ABBP1 HNRPAB
Q9H694	-0.644305706	2.972775879	BICC1
O43175	-0.644062042	1.635274759	PHGDH PGDH3
Q58WW2	-0.640813351	1.424155662	DCAF6 IQWD1 MSTP055
Q8IVT5	-0.6404562	0.938395871	KSR1 KSR
Q6DN90	-0.638786793	1.322881192	IQSEC1 ARFGEP100 BRAG2 KIAA0763
Q9NR56	-0.63845253	2.592267496	MBNL1 EXP KIAA0428 MBNL

P09651	-0.630268097	1.051695246	HNRNPA1 HNRPA1
Q12792	-0.626092911	1.170310756	TWF1 PTK9
Q08AF3	-0.623060226	1.582238783	SLFN5
P35249	-0.620382309	2.351241345	RFC4
Q9H3N1	-0.619044304	3.207502159	TMX1 TMX TXNDC TXNDC1 PSEC0085 UNQ235/PRO268
P54687	-0.618718147	2.852564464	BCAT1 BCT1 ECA39
P50897	-0.617620468	1.865343571	PPT1 CLN1 PPT
P02765	-0.615999222	0.624845234	AHSG FETUA PRO2743
P23229	-0.614983559	2.153472957	ITGA6
P62805	-0.613865852	1.186013217	H4C1 H4/A H4FH HIST1H4H;
P23921	-0.611782074	1.819419242	RRM1 RR1
O95297	-0.6090765	0.564862976	MPZL1 PZR UNQ849/PRO1787
Q13480	-0.607919693	2.385808564	GAB1
Q86Y82	-0.606455803	1.859726555	STX12
Q9P246	-0.604791641	2.996936242	STIM2 KIAA1482
Q07157	-0.603795052	2.826406753	TJP1 ZO1
P43246	-0.602457047	1.900234755	MSH2
P00736	-0.598744392	1.812945225	C1R
Q13126	-0.598589897	0.921899405	MTAP MSAP
Q9NTJ3	-0.595846176	2.626105737	SMC4 CAPC SMC4L1
P59768	-0.594006538	4.119699008	GNG2
Q14141	-0.593481064	2.10901831	SEPTIN6 KIAA0128 SEP2 SEPT6
Q9UHB7	-0.588662148	1.71439483	AFF4 AF5Q31 MCEF HSPC092
Q7Z2K6	-0.588181496	1.884288707	ERMP1 FXNA KIAA1815
Q99986	-0.583572388	2.200878913	VRK1
Q9NWY4	-0.58094883	2.181433053	HPF1 C4orf27
P07910	-0.575037956	1.266748948	HNRNPC HNRPC
O15347	-0.574450493	2.064933646	HMGB3 HMG2A HMG4
Q96GM5	-0.573966503	1.374451247	SMARCD1 BAF60A
Q16363	-0.570606232	2.047958463	LAMA4
P24821	-0.568867683	1.478738855	TNC HXB
Q8NI27	-0.568551064	1.41355512	THOC2 CXorf3
P05556	-0.565094948	1.125209789	ITGB1 FNRB MDF2 MSK12
P49116	-0.564684391	2.621197756	NR2C2 TAK1 TR4
P52701	-0.558670998	1.971260443	MSH6 GTBP
P26358	-0.556130409	2.552782512	DNMT1 AIM CXXC9 DNMT
P13611	-0.553534508	2.026250746	VCAN CSPG2
O14683	-0.551847458	2.228005153	TP53I11 PIG11
P21589	-0.55050087	1.331225778	NT5E NT5 NTE
Q8N474	-0.550230026	1.742264219	SFRP1 FRP FRP1 SARP2
Q86UK7	-0.549386978	1.707646668	ZNF598
P20700	-0.546476364	1.351720214	LMNB1 LMN2 LMNB
P21741	-0.541397095	2.016572281	MDK MK1 NEGF2

Q8IVF2	-0.541191578	0.951446011	AHNAK2 C14orf78 KIAA2019
Q96PE1	-0.539745331	1.730320097	ADGRA2 GPR124 KIAA1531 TEM5
Q9NTI5	-0.538558006	1.770784146	PDS5B APRIN AS3 KIAA0979
O60245	-0.537146568	2.044521969	PCDH7 BHPCDH
P42892	-0.536910057	2.288811851	ECE1
P54727	-0.536296844	0.688765346	RAD23B
P35659	-0.534368515	2.121004589	DEK
P40938	-0.534332752	1.612384715	RFC3
Q14978	-0.527452469	2.256701762	NOLC1 KIAA0035 NS5ATP13
Q9NVR2	-0.527165413	2.425853155	INTS10 C8orf35
P16949	-0.525506973	0.609067961	STMN1 C1orf215 LAP18 OP18
P98179	-0.525484085	2.58959841	RBM3 RNPL
P63241	-0.52512455	3.026764277	EIF5A
P07585	-0.516659737	1.781380103	DCN SLRR1B
Q00839	-0.512821198	1.031839679	HNRNPU C1orf199 HNRPU SAFA U21.1
O60828	-0.511919975	2.7365134	PQBP1 NPW38 JM26
P63279	-0.511069298	0.762837252	UBE2I UBC9 UBCE9
P12111	-0.510883331	1.4940049	COL6A3
O15021	-0.510759354	1.026127551	MAST4 KIAA0303
P98155	-0.509789467	1.791726676	VLDLR
Q13151	-0.506804466	1.102661906	HNRNPA0 HNRPA0
Q00169	-0.506240845	0.973813372	PITPNA PITPN
O14561	-0.50557518	0.396493068	NDUFAB1
Q8TF01	-0.49913311	2.684157603	PNISR C6orf111 SFRS18 SRRP130 HSPC261 HSPC306
Q9Y2D5	-0.497359276	1.925219101	AKAP2 KIAA0920 PRKA2
P60484	-0.496009827	2.16941428	PTEN MMAC1 TEP1
P63208	-0.495542526	0.992527624	SKP1 EMC19 OCP2 SKP1A TCEB1L
P61956	-0.495243073	1.356533124	SUMO2 SMT3B SMT3H2
Q9Y5B9	-0.494398117	1.548545856	SUPT16H FACT140 FACTP140
Q9H788	-0.493913651	1.785452232	SH2D4A PPP1R38 SH2A
P10412	-0.493883133	2.505474302	H1-4 H1F4 HIST1H1E
P18084	-0.493294716	2.053291373	ITGB5
Q9Y617	-0.489522934	1.265776623	PSAT1 PSA
Q9GZX9	-0.489343643	2.963760511	TWSG1 TSG PSEC0250
Q15758	-0.488967896	0.933173008	SLC1A5 ASCT2 M7V1 RDR RDRC
Q9UEY8	-0.488516808	3.004846213	ADD3 ADDL
P40189	-0.488324165	2.517526963	IL6ST
Q9P0V9	-0.486515045	2.179932118	SEPTIN10 SEPT10
P43243	-0.485370636	1.327424261	MATR3 KIAA0723
Q92599	-0.484562874	2.49562748	SEPTIN8 KIAA0202 SEPT8
P11387	-0.484402657	1.958694779	TOP1
P29372	-0.483072758	0.983962773	MPG AAG ANPG MID1
O60331	-0.478388786	2.315005405	PIP5K1C KIAA0589

Q5BJD5	-0.477235794	1.683507402	TMEM41B KIAA0033
P78524	-0.476501465	2.349398021	DENND2B HTS1 ST5
Q92945	-0.475517273	0.900981682	KHSRP FUBP2
P35637	-0.474406242	1.87647273	FUS TLS
P56199	-0.470170021	2.02561097	ITGA1
P35611	-0.469486237	2.341537998	ADD1 ADDA
Q9NZ08	-0.468272209	2.146821934	ERAP1 APPILS ARTS1 KIAA0525 UNQ584/PRO1154
P67936	-0.467996597	1.441112359	TPM4
Q7Z4V5	-0.467024803	0.837987462	HDGFL2 HDGF2 HDGFRP2 HRP2 UNQ785/PRO1604
P23219	-0.466869354	3.1816689	PTGS1 COX1
Q9H0U4	-0.466535568	0.493475638	RAB1B
P31942	-0.465519905	0.625645184	HNRNPH3 HNRPH3
Q9NZN4	-0.464439392	1.31961335	EHD2 PAST2
P19338	-0.46426487	1.487621883	NCL
O43166	-0.463727951	1.435127737	SIPA1L1 E6TP1 KIAA0440
P49407	-0.462481499	1.746786196	ARRB1 ARR1
P17302	-0.461338043	2.169331216	GJA1 GJAL
P42677	-0.46030426	1.191292563	RPS27 MPS1
P15927	-0.459980965	2.402855026	RPA2 REPA2 RPA32 RPA34
P81877	-0.458394527	1.636222555	SSBP2 SSDP2
Q15003	-0.457172394	2.498185927	NCAPH BRRN BRRN1 CAPH KIAA0074
Q7Z7L1	-0.456671715	1.130934643	SLFN11
O75410	-0.455906868	1.288133057	TACC1 KIAA1103
Q9HCL0	-0.455506325	1.831745448	PCDH18 KIAA1562
Q9Y592	-0.455356598	0.424343354	CEP83 CCDC41
Q9UIG0	-0.45333004	2.81859894	BAZ1B WBSC10 WBSCR10 WBSCR9 WSTF
P12109	-0.452178955	1.779672883	COL6A1
Q15645	-0.451969147	3.242571121	TRIP13 PCH2
P09234	-0.451347351	1.905340714	SNRPC
O43390	-0.451079369	1.154588377	HNRNPR HNRPR
Q9BWU0	-0.45014286	0.573926379	SLC4A1AP HLC3
P11766	-0.447033882	0.92284632	ADH5 ADHX FDH
P23246	-0.446613312	1.35903777	SFPQ PSF
P27797	-0.445878983	1.100567443	CALR CRTC
Q6UB99	-0.445289135	1.677274661	ANKRD11 ANCO1
P62316	-0.444417	0.63995966	SNRPD2 SNRPD1
Q96PU8	-0.444155693	2.432275878	QKI HKQ
Q9H3Q1	-0.443754196	1.579242847	CDC42EP4 BORG4 CEP4
Q96S82	-0.442850113	1.810546569	UBL7 BMSCUBP SB132
P12110	-0.442707062	1.738267695	COL6A2
Q92522	-0.442596436	2.229685702	H1-10 H1FX

Q2TAL8	-0.442590714	2.031652189	QRICH1
P18621	-0.441574097	0.667673522	RPL17
Q9ULH1	-0.438445091	1.798635116	ASAP1 DDEF1 KIAA1249 PAG2
P27694	-0.438297272	2.423621363	RPA1 REPA1 RPA70
Q9P266	-0.437824249	2.406939366	JCAD KIAA1462
Q9P1F3	-0.43778038	1.277056186	ABRACL C6orf115 HSPC280 PRO2013
P06493	-0.437655449	1.157940766	CDK1 CDC2 CDC28A CDKN1 P34CDC2
Q9Y3Z3	-0.437391281	3.312647066	SAMHD1 MOP5
Q15059	-0.436366558	0.944885331	BRD3 KIAA0043 RING3L
Q8WWM9	-0.434672356	2.295640197	CYGB STAP
P08240	-0.434622765	2.131526622	SRPRA SRPR
Q9Y3D8	-0.434476852	1.634958317	AK6 CINAP AD-004 CGI-137
P37108	-0.433853149	0.712892249	SRP14
P35080	-0.4337883	0.823592704	PFN2
O00625	-0.432704926	2.542304358	PIR
P31949	-0.431818008	0.395310234	S100A11 MLN70 S100C
Q9HB71	-0.43136692	0.983152727	CACYBP S100A6BP SIP PNAS-107
Q9Y4K4	-0.430438042	1.818651878	MAP4K5
Q8WXX5	-0.430102348	1.960030179	DNAJC9
O00339	-0.429355621	1.082564228	MATN2 UNQ193/PRO219
Q16576	-0.429232597	2.741027283	RBBP7 RBAP46
Q5VUA4	-0.428512573	2.340687699	ZNF318 HRIHFB2436
P61254	-0.428114891	0.669843986	RPL26
Q3SY69	-0.427709579	1.568401054	ALDH1L2
P06748	-0.426762581	0.879747182	NPM1 NPM
Q8N5M1	-0.426252365	1.391434005	ATPAF2 ATP12 LP3663
Q14161	-0.426235199	1.952275702	GIT2 KIAA0148
P63104	-0.425116539	0.793250533	YWHAZ
P13726	-0.424905777	1.809552873	F3
P36639	-0.424633026	2.769083921	NUDT1 MTH1
Q9BTT0	-0.423962593	2.895159363	ANP32E
Q14683	-0.416812897	2.639014304	SMC1A DXS423E KIAA0178 SB1.8 SMC1 SMC1L1
Q07955	-0.416556358	0.696934853	SRSF1 ASF SF2 SF2P33 SFRS1 OK/SW-cl.3
P61586	-0.416146278	1.702235602	RHOA ARH12 ARHA RHO12
Q71DI3	-0.415699005	1.84440733	H3C15 HIST2H3A; H3C14 H3F2 H3FM HIST2H3C; H3C13 HIST2H3D
P15502	-0.415443897	3.120633805	ELN
P49458	-0.415284157	1.585744656	SRP9
O75223	-0.41508007	1.915402739	GGCT C7orf24 CRF21
Q460N5	-0.414755821	2.582457231	PARP14 BAL2 KIAA1268
P26368	-0.414358139	0.960311689	U2AF2 U2AF65
Q99622	-0.412782669	4.559075681	C12orf57 C10

Q14444	-0.411097527	1.038280961	CAPRIN1 GPIAP1 GPIP137 M11S1 RNG105
Q9NWS0	-0.408054352	0.821205937	PIH1D1 NOP17
Q08945	-0.408002853	2.343558421	SSRP1 FACT80
Q96FJ2	-0.407922745	1.76218625	DYNLL2 DLC2
Q03164	-0.407195091	2.000002776	KMT2A ALL1 CXXC7 HRX HTRX MLL MLL1 TRX1
Q96BY9	-0.403335571	1.635111519	SARAF TMEM66 XTP3 HSPC035 NPD003 PSEC0019 UNQ1967/PRO4499
Q9Y4C2	-0.40218544	1.996288799	TCAF1 FAM115A KIAA0738
P56545	-0.401609421	1.846122421	CTBP2
Q9GZZ1	-0.401283264	3.162180858	NAA50 MAK3 NAT13 NAT5
Q7LGA3	-0.398602486	0.785675097	HS2ST1 HS2ST KIAA0448
Q14676	-0.396269321	1.54808502	MDC1 KIAA0170 NFBD1
O00478	-0.395503044	2.410783823	BTN3A3 BTF3
Q06330	-0.395091057	1.177673209	RBPJ IGKJRB IGKJRB1 RBPJK RBPSUH
Q5H9R7	-0.393755913	2.402628688	PPP6R3 C11orf23 KIAA1558 PP6R3 SAPL SAPS3
Q9BTT6	-0.393550396	0.934819238	LRRC1 LANO
Q14938	-0.391447067	1.954911867	NFIX
P52272	-0.390715599	0.794358022	HNRNPM HNRPM NAGR1
Q8NBF2	-0.390245438	2.758633499	NHLRC2
Q9UQE7	-0.389738083	3.428776065	SMC3 BAM BMH CSPG6 SMC3L1
Q15424	-0.389696121	0.850239565	SAFB HAP HET SAFB1
P52594	-0.388849258	2.648665754	AGFG1 HRB RAB RIP
Q5BKZ1	-0.388422966	2.694685758	ZNF326 ZIRD
P10321	-0.387804031	2.319365711	HLA-C HLAC
Q08357	-0.386432648	1.730711106	SLC20A2 GLVR2 PIT2
Q96FV9	-0.38633728	2.228432389	THOC1 HPR1
P55285	-0.386200905	1.335669458	CDH6
Q8WZA0	-0.386071205	0.563657863	LZIC
P20962	-0.38548851	0.808285652	PTMS
Q06210	-0.385300636	1.982568284	GFPT1 GFAT GFPT
P55083	-0.385033607	1.444830423	MFAP4
Q9NZN3	-0.384982109	2.209446629	EHD3 EHD2 PAST3
O43670	-0.384318352	1.308118363	ZNF207 BUGZ
Q16270	-0.38409996	1.602279322	IGFBP7 MAC25 PSF
Q9UBE0	-0.383242607	1.384730052	SAE1 AOS1 SUA1 UBLE1A
P80217	-0.382091522	1.937444168	IFI35 IFP35
O00571	-0.382037163	0.571064135	DDX3X DBX DDX3
A6NHR9	-0.38197422	1.888094765	SMCHD1 KIAA0650
O14979	-0.381764412	1.655824647	HNRNPDL HNRPDL JKTBP
Q9Y221	-0.380991936	2.061860704	NIP7 CGI-37 HSPC031 HSPC180 OK/SW-cl.76 OK/SW-cl.78
Q9H0W9	-0.380867958	0.366313562	C11orf54 LP4947 PTD012
Q15011	-0.379534245	1.988590004	HERPUD1 HERP KIAA0025 MIF1

O14737	-0.379371643	0.681517521	PDCD5 TFAR19
P40424	-0.379033566	1.008387936	PBX1 PRL
Q5RKV6	-0.378769875	1.555502728	EXOSC6 MTR3
P30043	-0.378591537	0.506106194	BLVRB FLR
Q9Y399	-0.378552437	0.980885235	MRPS2 CGI-91
P31943	-0.377477646	2.81834115	HNRNPH1 HNRPH HNRPH1
P49915	-0.376314163	0.818093087	GMPS
O75531	-0.375863075	2.773103041	BANF1 BAF BCRG1
O95425	-0.374678612	3.102310338	SVIL
Q9NRG9	-0.374561787	1.1664486	AAAS ADRACALA GL003
Q3ZCQ8	-0.374076843	0.605983065	TIMM50 TIM50 PRO1512
Q9UKX5	-0.373916626	1.74833228	ITGA11 MSTP018
O60885	-0.372560501	1.5526653	BRD4 HUNK1
P08621	-0.372397423	1.2503444	SNRNP70 RNPU1Z RPU1 SNRP70 U1AP1
Q9BZJ0	-0.371832848	1.217279245	CRNKL1 CRN CGI-201 MSTP021
Q9BVK6	-0.371636391	0.608244241	TMED9 GP25L2
O43169	-0.370918274	1.53661108	CYB5B CYB5M OMB5
P13010	-0.370399475	1.116169161	XRCC5 G22P2
P49711	-0.370008469	2.292351901	CTCF
P05362	-0.366435051	2.306288268	ICAM1
Q14119	-0.366322517	1.633531152	VEZF1 DB1 ZNF161
Q9NZI8	-0.36504364	0.884636361	IGF2BP1 CRDBP VICKZ1 ZBP1
Q6P179	-0.364766121	2.319428651	ERAP2 LRAP
O60684	-0.363998413	1.948680286	KPNA6 IPOA7
O43665	-0.36169529	2.543951413	RGS10
O43719	-0.360637665	2.303029233	HTATSF1
Q9H4I2	-0.36060524	0.777043936	ZHX3 KIAA0395 TIX1
Q96EB1	-0.360517025	1.600349436	ELP4 C11orf19 PAXNEB
Q9BVV7	-0.360388756	2.994201092	TIMM21 C18orf55 TIM21 HSPC154
O95470	-0.360016823	2.644763957	SGPL1 KIAA1252
Q16836	-0.359747887	2.233237889	HADH HAD HAD1 HADHSC SCHAD
Q9UMY4	-0.359534264	3.116573079	SNX12
Q9NSI2	-0.358662605	1.022643919	SLX9 C21orf70 FAM207A PRED56
Q1KMD3	-0.358257294	0.993244613	HNRNPUL2 HNRPUL2
Q9BZZ5	-0.357564926	1.731133004	API5 MIG8
P62306	-0.357164383	0.622601069	SNRPF PBSCF
O00193	-0.356975555	1.070085209	SMAP C11orf58
P82094	-0.35696888	2.129134099	TMF1 ARA160
P61960	-0.355728149	2.246551974	UFM1 C13orf20 BM-002
P12956	-0.354637146	1.039315917	XRCC6 G22P1
Q01130	-0.353322029	2.241098059	SRSF2 SFRS2
P30825	-0.35250473	1.931859387	SLC7A1 ATRC1 ERR REC1L
Q96ST3	-0.352474213	1.314994677	SIN3A

P32119	-0.351458549	1.105057901	PRDX2 NKEFB TDPX1
Q92688	-0.351177216	2.474115434	ANP32B APRIL PHAPI2
P48506	-0.351154327	2.668346357	GCLC GLCL GLCLC
P22392	-0.351139069	1.908863799	NME2 NM23B
P50895	-0.350037575	3.159526307	BCAM LU MSK19
P18887	-0.348125458	2.045930497	XRCC1
Q09666	-0.347769737	1.44745891	AHNAK PM227
Q9H425	-0.34704113	2.840709017	C1orf198
P17252	-0.34696579	2.071922425	PRKCA PKCA PRKACA
P52926	-0.346921921	2.345184989	HMGA2 HMGIC
P53985	-0.345061302	1.98038356	SLC16A1 MCT1
P29353	-0.344795227	1.814199563	SHC1 SHC SHCA
P09486	-0.344640732	0.593742228	SPARC ON
P82979	-0.3445158	1.860805951	SARNP HCC1 HSPC316
Q14865	-0.343997955	2.13080665	ARID5B DESRT MRF2
P35250	-0.343429565	0.949415034	RFC2
Q9Y696	-0.34336853	1.117686527	CLIC4
Q49A26	-0.342736244	1.705988666	GLYR1 HIBDL NDF NP60
Q8WYA6	-0.342585564	2.354798339	CTNNBL1 C20orf33 PP8304
Q6EMK4	-0.341220856	2.260019799	VASN SLITL2 UNQ314/PRO357/PRO1282
Q9BYV8	-0.341160774	2.399846517	CEP41 TSGA14
P35251	-0.339732647	1.090184189	RFC1 RFC140
P09012	-0.339300156	1.552355756	SNRPA
P07332	-0.339299679	0.595900162	FES FPS
Q63ZY3	-0.339048386	1.927150781	KANK2 ANKRD25 KIAA1518 MXRA3 SIP
Q9UNN8	-0.336709023	2.223908743	PROCR EPCR
P38159	-0.335760117	1.764461064	RBMX HNRPG RBMXP1
O60343	-0.335294724	1.763811773	TBC1D4 AS160 KIAA0603
Q99459	-0.335185051	1.69888261	CDC5L KIAA0432 PCDC5RP
Q9UKA9	-0.334658623	1.457397133	PTBP2 NPTB PTB PTBLP
P40925	-0.334635735	0.634651369	MDH1 MDHA
Q12946	-0.334536552	1.042347002	FOXF1 FKHL5 FREAC1
Q6ZN30	-0.333882332	0.870182805	BNC2
Q96RY7	-0.333642006	1.572883951	IFT140 KIAA0590 WDTC2
Q92820	-0.333590508	3.075054893	GGH
Q8WVY7	-0.333078384	1.751032355	UBLCP1
Q9BXB5	-0.332479477	1.824687238	OSBPL10 ORP10 OSBP9
Q02952	-0.331660271	2.673384757	AKAP12 AKAP250
Q2TAY7	-0.331365585	1.774281794	SMU1
Q9NUU7	-0.329818726	1.21882186	DDX19A DDX19L
P39060	-0.329629898	2.147742204	COL18A1
Q9P016	-0.327606201	2.652269886	THYN1 THY28 HSPC144 MDS012 My0054
P14406	-0.326850891	0.319939054	COX7A2 COX7AL

Q13464	-0.326836586	2.366427554	ROCK1
O95232	-0.326816559	3.023878566	LUC7L3 CREAP1 CROP O48
Q9Y3X0	-0.325847626	3.189856737	CCDC9
Q5U5X0	-0.324775696	3.399797159	LYRM7 C5orf31 MZM1L
P40261	-0.324039459	1.694155375	NNMT
Q92879	-0.323552132	2.024641792	CELF1 BRUNOL2 CUGBP CUGBP1 NAB50
Q9Y3Y2	-0.323410988	0.772402806	CHTOP C1orf77 FOP HT031 PP7704
Q14103	-0.323093414	0.98038156	HNRNPD AUF1 HNRPD
O00401	-0.320777893	2.574485759	WASL
Q14004	-0.320726395	1.756885097	CDK13 CDC2L CDC2L5 CHED KIAA1791
Q96PU4	-0.31999588	2.579302927	UHRF2 NIRF RNF107
Q7Z2W4	-0.319809914	1.818033334	ZC3HAV1 ZC3HDC2 PRO1677
Q13257	-0.319471359	2.170053095	MAD2L1 MAD2
P17676	-0.318658829	1.799587129	CEBPB TCF5 PP9092
P46063	-0.317986488	1.728003953	RECQL RECQ1 RECQL1
Q15428	-0.31724453	2.267108711	SF3A2 SAP62
P22626	-0.316877365	1.659507755	HNRNPA2B1 HNRPA2B1
Q9NYF8	-0.316282272	1.549164468	BCLAF1 BTF KIAA0164
P28347	-0.316233635	1.883532905	TEAD1 TCF13 TEF1
Q13555	-0.315883636	1.102966749	CAMK2G CAMK CAMK-II CAMKG
Q9NWB6	-0.315826416	2.919381701	ARGLU1
Q9BYW2	-0.315241814	0.989436455	SETD2 HIF1 HYPB KIAA1732 KMT3A SET2 HSPC069
Q13310	-0.314936638	1.141766949	PABPC4 APP1 PABP4
P29966	-0.314709663	1.311434008	MARCKS MACS PRKCSL
P62979	-0.314398766	0.683514698	RPS27A UBA80 UBCEP1
Q9UNZ5	-0.313420296	1.643142087	C19orf53 HSPC023
Q9P258	-0.31338501	1.533814811	RCC2 KIAA1470 TD60
O14745	-0.312306404	1.74340895	SLC9A3R1 NHERF NHERF1
Q9UJY4	-0.312285423	1.044175592	GGA2 KIAA1080
Q13017	-0.312026978	1.203606214	ARHGAP5 RHOGAP5
Q9BT40	-0.311394215	1.041553659	INPP5K PPS SKIP
O95999	-0.311155319	1.454578684	BCL10 CIPER CLAP
P62847	-0.311114311	0.553911263	RPS24
Q9UKM9	-0.311095238	0.780207013	RALY HNRPCL2 P542
P51858	-0.310785294	2.483286546	HDGF HMG1L2
P16152	-0.310038567	0.775779427	CBR1 CBR CRN SDR21C1
Q99471	-0.309937477	0.340934424	PFDN5 MM1 PFD5
Q15633	-0.309826851	1.99671204	TARBP2 TRBP
P30260	-0.309603691	3.852262487	CDC27 ANAPC3 D0S1430E D17S978E
Q9BUJ2	-0.30930233	0.823605795	HNRNPUL1 E1BAP5 HNRPUL1
Q765P7	-0.309099197	1.33238499	MTSS2 MTSS1L
Q15291	-0.308119774	1.607936943	RBBP5 RBQ3
P17844	-0.305668831	0.991497176	DDX5 G17P1 HELR HLR1

Q9Y625	-0.305626869	1.943468994	GPC6 UNQ369/PRO705
Q01082	-0.305587769	1.418615419	SPTBN1 SPTB2
Q4LDE5	-0.305478096	1.310195047	SVEP1 C9orf13 CCP22 SELOB
Q7Z6K3	-0.30462265	1.624067231	PTAR1
O00425	-0.304401398	1.092579095	IGF2BP3 IMP3 KOC1 VICKZ3
Q9BWW4	-0.304385185	2.663107839	SSBP3 SSDP SSDP1
Q06203	-0.304139137	1.51457965	PPAT GPAT
Q9Y320	-0.303907394	1.441859284	TMX2 TXNDC14 CGI-31 My009 PIG26 PSEC0045 UNQ237/PRO270
Q5T8P6	-0.302928925	1.370194277	RBM26 C13orf10 PRO1777
P48507	-0.302863121	1.491579662	GCLM GLCLR
P61978	-0.302419662	0.938103524	HNRNPK HNRPK
Q9UKF6	-0.302370071	2.143831096	CPSF3 CPSF73
O95456	-0.301651001	1.170287334	PSMG1 C21LRP DSCR2 PAC1
Q15637	-0.301080704	0.813427503	SF1 ZFM1 ZNF162
O95071	-0.299695015	1.179935546	UBR5 EDD EDD1 HYD KIAA0896
P67870	-0.299380302	0.585267541	CSNK2B CK2N G5A
P35754	-0.298530579	4.658360611	GLRX GRX
Q96QD9	-0.298440933	1.890477015	FYTTD1 UIF
P00966	-0.29797554	1.536541838	ASS1 ASS
Q9NR30	-0.297525406	3.639669245	DDX21
P43487	-0.296675682	0.575449862	RANBP1
Q13185	-0.295990944	2.105543278	CBX3
Q9Y4C8	-0.295864105	3.24103865	RBM19 KIAA0682
Q07666	-0.294669151	1.46930764	KHDRBS1 SAM68
Q8N5M4	-0.294094086	3.012955194	TTC9C
P23919	-0.293884277	1.132419801	DTYMK CDC8 TMPK TYMK
Q9UBT2	-0.293159485	2.00457897	UBA2 SAE2 UBLE1B HRIHFB2115
P05091	-0.292790413	1.71208296	ALDH2 ALDM
Q8NFC6	-0.291960716	2.346559017	BOD1L1 BOD1L FAM44A KIAA1327
P62750	-0.291769028	0.678470422	RPL23A
O94776	-0.29165554	2.003130109	MTA2 MTA1L1 PID
Q5VT25	-0.290693283	1.645981317	CDC42BPA KIAA0451
Q9BZQ8	-0.29022789	2.547553498	NIBAN1 C1orf24 FAM129A NIBAN GIG39
P08243	-0.290079117	1.145032166	ASNS TS11
Q9BY42	-0.290062904	1.921565462	RTF2 C20orf43 RTFDC1 AD-007 CDA05 HSPC164 HSPC169
P24752	-0.289500237	0.596399415	ACAT1 ACAT MAT
Q96JB5	-0.289390564	1.979037146	CDK5RAP3 IC53 LZAP MSTP016 OK/SW-cl.114 PP1553
Q14767	-0.288762093	1.480149506	LTBP2 C14orf141 LTBP3

P68431	-0.288591385	1.347816386	H3C1 H3FA HIST1H3A; H3C2 H3FL HIST1H3B; H3C3 H3FC HIST1H3C; H3C4 H3FB HIST1H3D; H3C6 H3FD HIST1H3E; H3C7 H3FI HIST1H3F; H3C8 H3FH HIST1H3G; H3C10 H3FK
Q9P2K3	-0.288527489	1.325532813	RCOR3 KIAA1343
Q13099	-0.287886143	1.658517876	IFT88 TG737 TTC10
Q92896	-0.287386894	0.728472425	GLG1 CFR1 ESL1 MG160
A1XBS5	-0.287056446	0.396669338	CIBAR1 FAM92A FAM92A1
Q93062	-0.286384583	2.324329047	RBPMS HERMES
Q9UMS4	-0.286372185	2.547157417	PRPF19 NMP200 PRP19 SNEV
P25054	-0.286141396	1.771280625	APC DP2.5
Q12874	-0.285682678	2.058221158	SF3A3 SAP61
Q16825	-0.285396576	1.751936066	PTPN21 PTPD1
O43491	-0.285348892	2.628631235	EPB41L2
P52298	-0.285256386	1.831809541	NCBP2 CBP20 PIG55
O15164	-0.284042358	1.775516237	TRIM24 RNF82 TIF1 TIF1A
P10619	-0.28288269	0.640157199	CTSA PPGB
P63313	-0.282590866	1.251287438	TMSB10 PTMB10 THYB10
Q8TDR0	-0.282369137	1.794247752	TRAF3IP1 IFT54 MIPT3
Q08211	-0.28181839	1.226221094	DHX9 DDX9 LKP NDH2
Q96AY4	-0.281337738	1.17121241	TTC28 KIAA1043 TPRBK
Q16629	-0.28133297	2.433224066	SRSF7 SFRS7
Q13263	-0.281167984	1.276806724	TRIM28 KAP1 RNF96 TIF1B
Q8TBA6	-0.28112793	2.885064711	GOLGA5 RETII RFG5 PIG31
Q13813	-0.280480385	1.924956467	SPTAN1 NEAS SPTA2
Q96S38	-0.279120445	0.599948414	RPS6KC1 RPK118
Q9H1J1	-0.278961182	1.364256823	UPF3A RENT3A UPF3
O94874	-0.278616905	2.716197198	UFL1 KIAA0776 MAXER NLBP RCAD
P63173	-0.278419495	0.675214412	RPL38
P51884	-0.278349876	0.949200193	LUM LDC SLRR2D
Q9Y6C2	-0.2781744	2.646511438	EMILIN1 EMI
P53634	-0.278049469	0.894654137	CTSC CPPI
P17301	-0.277826309	0.943302316	ITGA2 CD49B
Q8WXI9	-0.277306557	1.434289325	GATAD2B KIAA1150
Q13523	-0.277220726	1.801762656	PRPF4B KIAA0536 PRP4 PRP4H PRP4K
P58397	-0.276702404	1.308608837	ADAMTS12 UNQ1918/PRO4389
P60660	-0.276581764	0.996322751	MYL6
Q14254	-0.276560783	1.938629081	FLOT2 ESA1 M17S1
Q96DI7	-0.276491165	2.002847176	SNRNP40 PRP8BP SFP38 WDR57
Q9H4A6	-0.276329994	2.135308344	GOLPH3 GPP34
P25686	-0.276136398	1.792732247	DNAJB2 HSJ1 HSPF3
Q9NZL4	-0.276070595	2.026957978	HSPBP1 HSPBP PP1845

Q6VMQ6	-0.275348663	1.154030435	ATF7IP MCAF MCAF1
P37837	-0.275030136	0.886045214	TALDO1 TAL TALDO TALDOR
Q9UEE9	-0.27493	2.562141461	CFDP1 BCNT CENP-29
Q96B26	-0.27454567	1.903446687	EXOSC8 OIP2 RRP43
Q9Y224	-0.274305344	3.434042521	RTRAF C14orf166 CGI-99
Q9NUM4	-0.274264336	0.971579537	TMEM106B
O00148	-0.274010658	1.310433737	DDX39A DDX39
P62258	-0.274000168	0.634407757	YWHAE
Q96AM1	-0.272953987	0.956329275	MRGPRF GPR140 GPR168 MRGF PSEC0142
P48509	-0.27264595	0.368250851	CD151 TSPAN24
P04792	-0.272283554	1.206843166	HSPB1 HSP27 HSP28
Q04656	-0.271603584	1.121191843	ATP7A MC1 MNK
P34932	-0.271596909	0.620781648	HSPA4 APG2
P04406	-0.271584511	0.625891445	GAPDH GAPD CDABP0047 OK/SW-cl.12
P41091	-0.271172523	0.449464782	EIF2S3 EIF2G
Q13642	-0.270362854	1.852397092	FHL1 SLIM1
Q9NZU5	-0.270205498	1.981264435	LMCD1
Q96I24	-0.270106316	2.620365474	FUBP3 FBP3
Q14692	-0.269648552	1.978153234	BMS1 BMS1L KIAA0187
Q13976	-0.268993378	1.181592476	PRKG1 PRKG1B PRKGR1A PRKGR1B
Q92889	-0.268687248	2.539711255	ERCC4 ERCC11 XPF
P15144	-0.268458366	2.605431768	ANPEP APN CD13 PEPN
P84022	-0.268388748	1.365174576	SMAD3 MADH3
Q8NHV4	-0.268007278	1.500537158	NEDD1
O14907	-0.267921448	2.594853787	TAX1BP3 TIP1
P78357	-0.267560005	2.605749857	CNTNAP1 CASPR NRXN4
Q96MU7	-0.26703167	1.880435973	YTHDC1 KIAA1966 YT521
Q8N4H5	-0.266787529	1.608517885	TOMM5 C9orf105 TOM5
Q92765	-0.266168594	2.31606238	FRZB FIZ FRE FRP FRZB1 SFRP3
P33240	-0.265245914	1.076429601	CSTF2
Q13838	-0.264962196	2.682486609	DDX39B BAT1 UAP56
Q7Z3B4	-0.264612198	1.607496979	NUP54
P78527	-0.264440536	1.578275134	PRKDC HYRC HYRC1
Q68CQ7	-0.263929367	1.137436065	GLT8D1 GALA4A AD-017 MSTP137 UNQ572/PRO1134
O14967	-0.263506889	2.258101036	CLGN
Q13451	-0.263253212	1.012071409	FKBP5 AIG6 FKBP51
O60353	-0.263113022	0.589025632	FZD6
Q7Z6E9	-0.262859344	1.107591079	RBBP6 P2PR PACT RBQ1 My038
P25116	-0.262573242	1.437487256	F2R CF2R PAR1 TR
O14980	-0.262460709	0.892140309	XPO1 CRM1
Q15233	-0.262298584	0.80804845	NONO NRB54
Q6PJT7	-0.261350632	1.680436322	ZC3H14

Q9BPZ3	-0.261109352	1.577276758	PAIP2 PAIP2A HSPC218
Q9BS40	-0.2610569	0.338046384	LXN
Q8N3X1	-0.260963917	2.144541335	FNBP4 FBP30 KIAA1014
P08754	-0.260424614	1.872731253	GNAI3
O43143	-0.260404587	1.947217999	DHX15 DBP1 DDX15
P05387	-0.260375977	2.772838077	RPLP2 D11S2243E RPP2
P04921	-0.259209156	1.001671743	GYPC GLPC GPC
P46778	-0.25908947	0.691717665	RPL21
P62826	-0.25903511	0.590196502	RAN ARA24 OK/SW-cl.81
Q9Y266	-0.25823307	1.072938785	NUDC
Q9UHI8	-0.257948875	1.769033116	ADAMTS1 KIAA1346 METH1
P49591	-0.257885933	0.966823375	SARS1 SARS SERS
Q9UJZ1	-0.257470131	0.786989006	STOML2 SLP2 HSPC108
Q9Y3I0	-0.257127762	0.949129819	RTCB C22orf28 HSPC117
P05388	-0.25711441	2.367013612	RPLP0
Q9Y2W1	-0.256773949	1.57859838	THRAP3 BCLAF2 TRAP150
Q5VT52	-0.255708694	1.79221281	RPRD2 KIAA0460 HSPC099
Q9UH99	-0.255556107	2.56930452	SUN2 FRIGG KIAA0668 RAB5IP UNC84B
P20908	-0.255518913	1.05969827	COL5A1
P62304	-0.255321503	1.601528271	SNRPE
Q9H1B7	-0.255005836	1.136724789	IRF2BPL C14orf4 EAP1 KIAA1865 My039
Q9H2H8	-0.254968643	1.231235006	PPIL3
Q9UK45	-0.254014015	2.68245717	LSM7
P20339	-0.253572464	2.188694534	RAB5A RAB5
P63167	-0.252041817	0.853566605	DYNLL1 DLC1 DNCL1 DNCLC1 HDLC1
Q5QJ74	-0.251939774	1.318980529	TBCEL LRRC35
O00161	-0.251795769	1.50685954	SNAP23
P13693	-0.251694679	2.111206084	TPT1
Q8WUH6	-0.251571655	0.662464228	TMEM263 C12orf23
Q9Y680	-0.251190186	1.399945919	FKBP7 FKBP23 UNQ670/PRO1304
Q86VY4	-0.251125336	1.387601392	TSPYL5 KIAA1750
Q9H7F0	-0.25080061	0.857087269	ATP13A3 AFURS1
Q9UBL3	-0.250273705	1.859405633	ASH2L ASH2L1
Q14195	-0.250264168	1.654233747	DPYSL3 CRMP4 DRP3 ULIP ULIP1
Q15113	-0.249834061	1.810563848	PCOLCE PCPE1
Q969X5	-0.249568939	2.034811369	ERGIC1 ERGIC32 KIAA1181 HT034
Q9HAU4	-0.24946022	2.477915019	SMURF2
P24043	-0.249128342	1.062468555	LAMA2 LAMM
Q9UH65	-0.249066353	2.366520737	SWAP70 KIAA0640 HSPC321
P29323	-0.248398781	1.408920283	EPHB2 DRT EPHT3 EPTH3 ERK HEK5 TYRO5
Q99417	-0.248386383	1.672703514	MYCBP AMY1
Q6NZY4	-0.248007774	1.656240464	ZCCHC8
Q9Y5B8	-0.247866631	1.742006009	NME7

P06400	-0.247549057	2.100626837	RB1
P49419	-0.247412682	0.895956562	ALDH7A1 ATQ1
P84090	-0.246888161	1.60572423	ERH
Q86U42	-0.246845245	1.388640967	PABPN1 PAB2 PABP2
P62318	-0.246816635	1.123360925	SNRPD3
Q9BQ69	-0.246560097	2.436471122	MACROD1 LRP16
Q96F85	-0.246446609	1.969602958	CNRIP1 C2orf32
P46060	-0.246172905	1.405331355	RANGAP1 KIAA1835 SD
O14981	-0.245802879	2.084127335	BTAF1 TAF172
P41212	-0.245663643	1.324485596	ETV6 TEL TEL1
Q03135	-0.245596886	1.447485212	CAV1 CAV
P42224	-0.245546341	2.971924584	STAT1
P62995	-0.24473381	1.73237363	TRA2B SFRS10
Q9H0C8	-0.244345665	1.953733667	ILKAP
Q7Z7K6	-0.244239807	1.727754803	CENPV PRR6
Q15392	-0.244059563	1.49402374	DHCR24 KIAA0018
O75940	-0.243142128	2.038048224	SMNDC1 SMNR SPF30
O75494	-0.242907524	2.316412712	SRSF10 FUSIP1 FUSIP2 SFRS13A TASR
Q8WVC6	-0.24277401	1.811078186	DCAKD
Q7Z6I8	-0.242703438	0.626856013	C5orf24
Q8WW12	-0.242531776	2.02895251	PCNP
O75787	-0.242454529	2.027931142	ATP6AP2 ATP6IP2 CAPER ELDF10 HT028 MSTP009 PSEC0072
P05455	-0.241928101	0.820450278	SSB
Q92841	-0.241651535	0.950999197	DDX17
Q07021	-0.241413116	2.392506083	C1QBP GC1QBP HABP1 SF2P32
Q15459	-0.241056442	1.227552635	SF3A1 SAP114
Q9H147	-0.240933895	0.797459669	DNTTIP1 C20orf167 TDIF1
O60841	-0.240744591	1.024996164	EIF5B IF2 KIAA0741
P23381	-0.240473747	0.694481629	WARS1 IFI53 WARS WRS
P20337	-0.240377426	1.804695906	RAB3B
Q9Y5A7	-0.239981651	2.166715981	NUB1 NYREN18
Q14257	-0.23928833	1.847660115	RCN2 ERC55
O60610	-0.239263535	3.323263355	DIAPH1 DIAP1
P57721	-0.239200592	1.879468902	PCBP3 PCBP3-OT1 PCBP3OT
Q96M27	-0.239164352	0.702663468	PRRC1
Q9UDY2	-0.23865509	1.50055325	TJP2 X104 ZO2
O14818	-0.238183022	0.444187365	PSMA7 HSPC
Q99707	-0.238175392	1.934892358	MTR
Q14624	-0.238139153	1.278463666	ITIH4 IHRP ITIHL1 PK120 PRO1851
P20042	-0.237874985	0.448833173	EIF2S2 EIF2B
Q13123	-0.237610817	1.86985585	IK RED RER
Q9NZR1	-0.237483978	0.6528146	TMOD2 NTMOD
Q92576	-0.237366676	1.334178707	PHF3 KIAA0244

Q14914	-0.236907959	0.576512996	PTGR1 LTB4DH
P61218	-0.236824036	0.639981799	POLR2F POLRF
Q5ZPR3	-0.236740112	2.947786121	CD276 B7H3 PSEC0249 UNQ309/PRO352
P29992	-0.23656559	2.104502563	GNA11 GA11
Q9NWL6	-0.236303329	1.711480351	ASNSD1 NS3TP1 Nbla00058
Q92609	-0.236079216	2.390847888	TBC1D5 KIAA0210
Q12905	-0.235825539	0.843709718	ILF2 NF45 PRO3063
Q86VM9	-0.235767365	2.258279446	ZC3H18 NHN1
Q96AE4	-0.235538483	1.232255354	FUBP1
Q96ME7	-0.2355299	1.359589429	ZNF512 KIAA1805
Q9UKV3	-0.235322952	3.147690789	ACIN1 ACINUS KIAA0670
P62249	-0.235251427	0.703591779	RPS16
O43809	-0.235228539	1.068007135	NUDT21 CFIM25 CPSF25 CPSF5
Q9NQZ5	-0.234964371	0.512798347	STARD7 GTT1
P11142	-0.234540939	0.856590265	HSPA8 HSC70 HSP73 HSPA10
P62280	-0.234415054	0.409763195	RPS11
Q9HCU0	-0.234253883	1.22583473	CD248 CD164L1 TEM1
Q9BQE5	-0.234155655	2.269205792	APOL2
P09661	-0.234039307	1.25144785	SNRPA1
O60264	-0.233782768	2.172731801	SMARCA5 SNF2H WCRF135
P29279	-0.233503342	2.125741947	CCN2 CTGF HCS24 IGFBP8
P62633	-0.233422279	2.627441742	CNBP RNF163 ZNF9
Q7L7X3	-0.232507706	1.553098971	TAOK1 KIAA1361 MAP3K16 MAPKK
Q9Y3E1	-0.232105255	0.805586028	HDGFL3 HDGF2 HDGFRP3 CGI-142
P53041	-0.232001305	0.987575708	PPP5C PPP5
P07355	-0.231911659	0.884906873	ANXA2 ANX2 ANX2L4 CAL1H LPC2D
O43660	-0.231579781	1.392591037	PLRG1
Q15185	-0.231445313	0.894722682	PTGES3 P23 TEBP
Q14839	-0.231171608	1.552086741	CHD4
Q9Y4Z0	-0.230927467	2.11806738	LSM4
Q9H4A5	-0.230855942	0.775635328	GOLPH3L GPP34R
Q96EP5	-0.230648994	0.523542281	DAZAP1
Q9H2P0	-0.230413437	1.489406072	ADNP ADNP1 KIAA0784
O00422	-0.230260849	2.300238748	SAP18 GIG38
Q15582	-0.230257988	0.98646521	TGFBI BIGH3
Q9GZU8	-0.230022907	1.151774143	PSME3IP1 C16orf94 FAM192A NIP30 PIP30 CDA018 CDA10
O95081	-0.229310989	0.61468508	AGFG2 HRBL RABR
Q8WXF1	-0.229279518	2.10699244	PSPC1 PSP1
P61769	-0.229254723	1.724690582	B2M CDABP0092 HDCMA22P
Q96KR1	-0.228845596	1.697343083	ZFR
P83731	-0.228322029	0.586596792	RPL24
Q14739	-0.227936745	3.000214653	LBR
Q9NWU2	-0.227909088	0.848714072	GID8 C20orf11 TWA1

Q09028	-0.227752686	1.402033371	RBBP4 RBAP48
O00483	-0.227683067	0.971355324	NDUFA4
Q15366	-0.227089882	0.93150939	PCBP2
Q03252	-0.226705551	1.994195495	LMNB2 LMN2
Q9NPL8	-0.226522446	1.004775648	TIMMDC1 C3orf1 UNQ247/PRO284
P85037	-0.225877762	2.040636234	FOXK1 MNF
Q9H6B4	-0.225715637	1.690465082	CLMP ACAM ASAM UNQ318/PRO363
Q9UBW7	-0.22565937	1.581168501	ZMYM2 FIM RAMP ZNF198
Q9H7N4	-0.225479126	1.474812265	SCAF1 SFRS19 SRA1
O75063	-0.225461006	1.022145149	FAM20B KIAA0475
Q9UHX1	-0.22521019	1.87016114	PUF60 FIR ROBPI SIAHBP1
Q9Y6N5	-0.225028992	1.064446898	SQOR SQRDL CGI-44
P03928	-0.224676132	0.274651577	MT-ATP8 ATP8 ATPASE8 MTATP8
P51153	-0.224285126	0.614577728	RAB13 GIG4
P13639	-0.224059105	0.799262354	EEF2 EF2
Q9GZT4	-0.223975182	2.382157092	SRR
Q96J01	-0.22395134	0.966038347	THOC3
Q13427	-0.223596573	1.960503335	PPIG
Q8NFH5	-0.223191261	1.132270459	NUP35 MP44 NUP53
O00391	-0.22317791	2.586449746	QSOX1 QSCN6 UNQ2520/PRO6013
O95361	-0.22315979	2.153145016	TRIM16 EBBP
Q13148	-0.222710609	0.691717068	TARDBP TDP43
P49755	-0.222316742	0.966170516	TMED10 TMP21
Q9Y547	-0.222307205	1.453109587	HSPB11 C1orf41 IFT25 HSPC034
P84103	-0.222191811	0.84078342	SRSF3 SFRS3 SRP20
Q7L1V2	-0.220963478	0.950444205	MON1B HSRG1 KIAA0872 SAND2
P35613	-0.220516205	0.914266676	BSG UNQ6505/PRO21383
P42574	-0.219903946	2.430145573	CASP3 CPP32
O75955	-0.219818115	1.653297165	FLOT1
Q9BWD1	-0.219813347	0.726400788	ACAT2 ACTL
Q8N392	-0.219382286	2.477721735	ARHGAP18
P38919	-0.218953133	1.357953096	EIF4A3 DDX48 KIAA0111
Q9UI08	-0.218251705	1.219349121	EVL RNB6
Q16630	-0.218062401	2.175709608	CPSF6 CFIM68
O60869	-0.217811584	1.706552144	EDF1
P26599	-0.217679024	0.833659206	PTBP1 PTB
Q15287	-0.217608452	1.189484261	RNPS1 LDC2
Q7L014	-0.217573166	1.446826637	DDX46 KIAA0801
Q7Z5K2	-0.217206001	1.571806816	WAPL FOE KIAA0261 WAPAL
P07093	-0.216600418	1.073923251	SERPINE2 PI7 PN1
O60493	-0.216567039	1.907839757	SNX3
Q8NC51	-0.216538429	1.207995014	SERBP1 PAIRBP1 CGI-55
P62312	-0.216504097	1.626675229	LSM6

Q6NZI2	-0.216363907	1.076561572	CAVIN1 PTRF FKSG13
Q9Y2I8	-0.216291428	2.620604069	WDR37 KIAA0982
Q9Y3A6	-0.21627903	1.705612438	TMED5 CGI-100 UNQ397/PRO733
O14497	-0.216253281	1.616510232	ARID1A BAF250 BAF250A C1orf4 OSA1 SMARCF1
O95379	-0.216192245	0.744348171	TNFAIP8
O00299	-0.21615696	1.019987923	CLIC1 G6 NCC27
Q9HC07	-0.21583271	1.587056372	TMEM165 TPARL
O75323	-0.215788841	1.129597777	NIPSNAP2 GBAS
Q68EM7	-0.215695381	1.342066772	ARHGAP17 RICH1 MSTP066 MSTP110
Q12996	-0.215665817	1.627556938	CSTF3
Q86W42	-0.215557098	1.514801602	THOC6 WDR58 PSEC0006
Q15126	-0.215385437	2.027846424	PMVK PMKI
O14672	-0.214485168	1.938936718	ADAM10 KUZ MADM
Q15843	-0.214388847	1.807717345	NEDD8
Q9NQ29	-0.214156151	1.526685964	LUC7L LUC7L1
Q96QC0	-0.213723183	1.446923623	PPP1R10 CAT53 FB19 PNUTS
Q08752	-0.213322639	2.376735534	PPID CYP40 CYPD
Q9NVM6	-0.213295937	1.305859698	DNAJC17
P01023	-0.213110924	1.364431748	A2M CPAMD5 FWP007
Q14498	-0.212870598	2.856606589	RBM39 HCC1 RNPC2
Q9Y5K5	-0.212827682	2.964975248	UCHL5 UCH37 AD-019 CGI-70
Q9Y3C6	-0.212353706	1.18381055	PPIL1 CYPL1 CGI-124 UNQ2425/PRO4984
Q9H8W4	-0.211813927	1.531760778	PLEKHF2 ZFYVE18
Q9BY44	-0.211668968	1.142883377	EIF2A CDA02 MSTP004 MSTP089
Q10570	-0.211248398	1.201818475	CPSF1 CPSF160
Q8WUD1	-0.211034775	1.256899993	RAB2B
Q9H9B4	-0.209971428	0.877695601	SFXN1
P36405	-0.209867477	0.578772853	ARL3 ARFL3
Q9Y383	-0.209553719	2.857531635	LUC7L2 CGI-59 CGI-74
P35556	-0.209549904	1.503047368	FBN2
Q92743	-0.209294319	1.991160539	HTRA1 HTRA PRSS11
P62879	-0.208963394	1.507524347	GNB2
O43865	-0.208628654	2.182762043	AHCYL1 DCAL IRBIT XPVKONA
P41250	-0.208582878	0.713566915	GARS1 GARS
P52597	-0.208426476	1.613517203	HNRNPF HNRPF
P21810	-0.208156586	1.825760195	BGN SLRR1A
P49207	-0.20783329	0.484020692	RPL34
P55081	-0.207681656	0.947669798	MFAP1
Q8NHH9	-0.207499504	0.923296417	ATL2 ARL6IP2
P60866	-0.20744133	1.036960715	RPS20
Q96RK0	-0.206886292	0.458316586	CIC KIAA0306
Q15363	-0.206711769	0.513165985	TMED2 RNP24
Q99829	-0.206450462	0.759327788	CPNE1 CPN1

O43813	-0.206439018	1.515018372	LANCL1 GPR69A
Q9UPU7	-0.206254005	2.309555437	TBC1D2B KIAA1055
Q7Z4I7	-0.206020355	1.242755762	LIMS2 PINCH2
Q96T37	-0.205913544	1.709491985	RBM15 OTT OTT1
Q92968	-0.205810547	1.594050111	PEX13
O14908	-0.205068588	2.05947016	GIPC1 C19orf3 GIPC RGS19IP1
Q9BX84	-0.204987526	1.820328697	TRPM6 CHAK2
P00167	-0.203784943	1.258663678	CYB5A CYB5
P04899	-0.203766823	0.650156341	GNAI2 GNAI2B
Q9Y282	-0.203158379	2.449380994	ERGIC3 C20orf47 ERV46 SDBCAG84 CGI-54 PRO0989
Q6UX04	-0.202820778	0.70166342	CWC27 SDCCAG10 UNQ438/PRO871
O15061	-0.202776909	1.105374512	SYNM DMN KIAA0353 SYN
Q9BW27	-0.202571869	2.855281099	NUP85 NUP75 PCNT1
P50148	-0.202553749	1.928213905	GNAQ GAQ
Q9Y2K1	-0.202476501	1.048219881	ZBTB1 KIAA0997
Q9GZT9	-0.201910019	1.216670647	EGLN1 C1orf12 PNAS-118 PNAS-137
P27816	-0.201753616	0.781234127	MAP4
Q92930	-0.201463699	1.673111728	RAB8B
P55060	-0.201210976	0.919973222	CSE1L CAS XPO2
Q92769	-0.200814247	2.544027107	HDAC2
Q9UL46	-0.200387001	1.51205944	PSME2
P41208	-0.200042725	2.010316108	CETN2 CALT CEN2
P00338	-0.199791908	0.545209569	LDHA PIG19
P00492	-0.19964695	1.151854407	HPRT1 HPRT
Q9NPA8	-0.199364662	1.828566781	ENY2 DC6
P80303	-0.199303627	3.091671942	NUCB2 NEFA
P11586	-0.199285507	2.146181781	MTHFD1 MTHFC MTHFD
Q15121	-0.19906044	2.25796986	PEA15
O00461	-0.198825836	0.878341953	GOLIM4 GIMPC GOLPH4 GPP130
Q6UXV4	-0.198814392	0.722501512	APOOL CXorf33 FAM121A MIC27 UNQ8193/PRO23204
P50416	-0.198646545	1.965868865	CPT1A CPT1
P37275	-0.198571205	0.817974006	ZEB1 AREB6 TCF8
Q96D15	-0.198569298	2.071727024	RCN3 UNQ239/PRO272
Q9UMR2	-0.198429108	0.807028101	DDX19B DBP5 DDX19 TDBP
Q9H792	-0.198316574	2.781927648	PEAK1 KIAA2002
Q9NVD7	-0.198150635	0.628226545	PARVA MXRA2
Q9HCE0	-0.198058605	1.191735331	EPG5 KIAA1632
P62269	-0.198039055	1.179458082	RPS18 D6S218E
P50579	-0.197166443	1.318804372	METAP2 MNPEP P67EIF2
P48681	-0.197099686	0.964246225	NES Nbla00170
Q9NVI7	-0.196861267	0.68362432	ATAD3A
P26196	-0.196643829	2.374250849	DDX6 HLR2 RCK

Q9HA64	-0.196015358	1.650096083	FN3KRP
Q15154	-0.195996284	1.085010985	PCM1
Q08722	-0.195747375	2.443736228	CD47 MER6
Q07954	-0.1957407	0.919677979	LRP1 A2MR APR
P30405	-0.195668221	0.885239078	PPIF CYP3
Q9Y3L5	-0.195415497	1.938595895	RAP2C
Q92793	-0.194621086	0.75343866	CREBBP CBP
Q9HAV0	-0.194549561	1.550441512	GNB4
Q15555	-0.194459915	2.279024981	MAPRE2 RP1
Q6KC79	-0.194426537	1.0632922	NIPBL IDN3 SCC2
Q9UQB8	-0.1938591	2.883522926	BAIAP2
Q12906	-0.19370079	0.985736057	ILF3 DRBF MPHOSPH4 NF90
P14625	-0.19334507	0.883321007	HSP90B1 GRP94 TRA1
Q9BX40	-0.193270683	2.44629895	LSM14B C20orf40 FAM61B RAP55B
Q96HY6	-0.193133354	1.43057941	DDRGK1 C20orf116 UFBP1
Q69YN2	-0.192984581	1.314090194	CWF19L1
Q96P47	-0.192946434	0.888626682	AGAP3 CENTG3
Q92499	-0.192768097	0.677526578	DDX1
Q8TDB6	-0.192751884	1.085469192	DTX3L BBAP
Q9P2J5	-0.192714691	1.027896538	LARS1 KIAA1352 LARS
P09382	-0.192321777	0.935772666	LGALS1
P20290	-0.192107201	0.847662604	BTF3 NACB OK/SW-cl.8
P61106	-0.191854477	0.636604904	RAB14
P62899	-0.191673279	0.977637524	RPL31
P24468	-0.19122982	0.999411298	NR2F2 ARP1 TFCOUP2
Q6DD87	-0.190926552	3.259799529	ZNF787
O14641	-0.190582275	1.490804582	DVL2
P36871	-0.190410614	1.164643348	PGM1
P06703	-0.190126419	0.809423285	S100A6 CACY
Q8N8A6	-0.189784527	1.738091077	DDX51
P49903	-0.189453125	1.634995571	SEPHS1 SELD SPS SPS1
Q96SQ9	-0.188824177	0.584824327	CYP2S1 UNQ891/PRO1906
Q9UKZ1	-0.188367844	1.409218956	CNOT11 C2orf29 C40
Q15269	-0.18828392	1.289057903	PWP2 PWP2H
P09601	-0.187364578	1.382565346	HMOX1 HO HO1
Q15057	-0.187128067	1.707474875	ACAP2 CENTB2 KIAA0041
Q13363	-0.186925888	1.072300333	CTBP1 CTBP
O00505	-0.18691349	1.110032086	KPNA3 QIP2
Q13573	-0.186565399	1.659058983	SNW1 SKIIP SKIP
Q14919	-0.186014175	0.524944988	DRAP1
Q13442	-0.185851097	0.585221666	PDAP1 HASPP28
Q05519	-0.184901237	2.195004752	SRSF11 SFRS11
Q9Y5V0	-0.184425354	0.747376947	ZNF706 HSPC038 PNAS-113

Q8TED1	-0.184394836	1.181131263	GPX8 UNQ847/PRO1785
Q7LBC6	-0.18430233	1.363141359	KDM3B C5orf7 JHDM2B JMJD1B KIAA1082
Q9Y2W2	-0.184049606	1.062683499	WBP11 NPWBP SIPP1 SNP70
Q8N2S1	-0.183602333	0.794895004	LTBP4
Q9UPY3	-0.182842255	1.252768125	DICER1 DICER HERNA KIAA0928
O00300	-0.182799339	1.214742682	TNFRSF11B OCIF OPG
Q9P1Z2	-0.182210922	0.931929781	CALCOCO1 KIAA1536 PP13275 UNQ2436/PRO4996
P61247	-0.182122231	0.647577952	RPS3A FTE1 MFTL
Q9H6Z4	-0.181941986	2.550644593	RANBP3
Q13428	-0.181780815	1.452462687	TCOF1
P04075	-0.18173027	1.091863111	ALDOA ALDA
Q00325	-0.181491852	0.868201193	SLC25A3 PHC OK/SW-cl.48
O15355	-0.181218147	1.640017086	PPM1G PPM1C
Q9NX62	-0.18116188	2.561506936	BPNT2 IMPA3 IMPAD1
Q13247	-0.18103981	0.508775509	SRSF6 SFRS6 SRP55
P52907	-0.180765152	1.786456093	CAPZA1
Q9Y6M5	-0.180685043	1.189103449	SLC30A1 ZNT1
Q9ULZ3	-0.180608749	1.749814107	PYCARD ASC CARD5 TMS1
P19823	-0.180187225	1.126214476	ITIH2 IGHEP2
Q15404	-0.18003273	3.522011732	RSU1 RSP1
Q7Z6B7	-0.179851532	1.016441707	SRGAP1 ARHGAP13 KIAA1304
P08579	-0.179778099	1.570221821	SNRPB2
P15531	-0.179553032	2.041069114	NME1 NDPKA NM23
O60573	-0.179406166	1.882253776	EIF4E2 EIF4EL3
Q01844	-0.179381371	1.095792939	EWSR1 EWS
Q9UBB9	-0.179261208	0.912254312	TFIP11 STIP HSPC006
P08195	-0.179158211	0.913162884	SLC3A2 MDU1
Q969U7	-0.179096222	1.215867077	PSMG2 HCCA3 PAC2 TNFSF5IP1
O60506	-0.178990364	1.284545184	SYNCRIP HNRPQ NSAP1
Q9Y3B4	-0.178837776	2.513834931	SF3B6 SAP14 SF3B14 SF3B14A CGI-110 HSPC175 HT006
Q7L5Y9	-0.178736687	1.237717039	MAEA EMP HLC10 PIG5
Q9Y3E7	-0.178707123	0.997450031	CHMP3 CGI149 NEDF VPS24 CGI-149
Q5VZL5	-0.178520203	2.650983464	ZMYM4 KIAA0425 ZNF262
Q14790	-0.178415298	0.625790422	CASP8 MCH5
P55265	-0.178395271	1.530878757	ADAR ADAR1 DSRAD G1P1 IFI4
O14558	-0.178385735	2.585593043	HSPB6
Q9H773	-0.178263664	0.637302007	DCTPP1 XTP3TPA CDA03
Q9H334	-0.177993774	0.955857103	FOXP1 HSPC215
Q96CT7	-0.177839279	1.818276573	CCDC124
P62273	-0.177833557	0.834203358	RPS29
P39019	-0.17774868	0.938883485	RPS19
Q9Y5L0	-0.177728653	1.634235442	TNPO3 IPO12

Q9UQ80	-0.177638054	0.752012884	PA2G4 EBP1
Q9P013	-0.177379608	0.644873827	CWC15 C11orf5 AD-002 HSPC148
Q9NVF7	-0.177284241	0.736028602	FBXO28 CENP-30 KIAA0483
Q13625	-0.17719841	0.65656029	TP53BP2 ASPP2 BBP
O00423	-0.177048683	1.630574385	EML1 EMAP1 EMAPL EMAPL1
P18754	-0.176808357	1.630894864	RCC1 CHC1
P62140	-0.176769257	1.472202926	PPP1CB
Q8N130	-0.17672348	1.743098728	SLC34A3 NPT2C NPTIIC
P50995	-0.176560402	0.801965735	ANXA11 ANX11
P24928	-0.176369667	2.803085615	POLR2A POLR2
P18077	-0.176017761	0.425923437	RPL35A GIG33
Q8NEY1	-0.175765038	1.328837627	NAV1 KIAA1151 KIAA1213 POMFIL3 STEERIN1
Q9Y5V3	-0.175569534	1.490151456	MAGED1 NRAGE PP2250 PRO2292
O75150	-0.175543785	2.401737992	RNF40 BRE1B KIAA0661
Q99436	-0.175423622	0.396027875	PSMB7 Z
Q9Y6A4	-0.175354004	1.222711725	CFAP20 BUG22 C16orf80 GTL3
Q15643	-0.174772263	1.632691958	TRIP11 CEV14
Q9HC36	-0.174379349	1.915724245	MRM3 RNMTL1 HC90
Q7Z6K5	-0.173810959	1.046506116	ARPIN C15orf38
Q01650	-0.173650742	1.667297359	SLC7A5 CD98LC LAT1 MPE16
Q16643	-0.173492432	0.655887238	DBN1 D0S117E
P27707	-0.173415184	0.743876558	DCK
P57105	-0.173359871	1.465670175	SYNJ2BP OMP25
Q8IZ07	-0.173268318	1.440515361	ANKRD13A ANKRD13
Q5VTL8	-0.173118591	1.567067205	PRPF38B
Q96DB5	-0.173051834	1.446176912	RMDN1 FAM82B CGI-90
Q9NY27	-0.172838211	1.890308628	PPP4R2 SBBI57
P49768	-0.171991825	0.814513156	PSEN1 AD3 PS1 PSNL1
Q6P1N0	-0.171843529	0.802318007	CC2D1A AKI1
Q96C23	-0.171771049	1.729136956	GALM BLOCK25
Q9BVG4	-0.171538353	2.135853473	PBDC1 CXorf26
P62913	-0.170948982	0.585322418	RPL11
Q86YT6	-0.170900345	1.352695822	MIB1 DIP1 KIAA1323 ZZANK2
Q9BTE3	-0.170882225	0.903997201	MCMBP C10orf119
Q8IZV5	-0.170675278	1.658109614	RDH10 SDR16C4 UNQ9375/PRO34191
P13716	-0.170316696	0.943704663	ALAD
P30049	-0.170166016	0.452529979	ATP5F1D ATP5D
Q9Y265	-0.170044899	1.587176413	RUVBL1 INO80H NMP238 TIP49 TIP49A
Q13443	-0.170005798	1.245644585	ADAM9 KIAA0021 MCMP MDC9 MLTNG
Q9GZZ9	-0.169582367	2.071344173	UBA5 UBE1DC1
Q5T200	-0.169471741	0.584242243	ZC3H13 KIAA0853
P40121	-0.169275284	0.817526416	CAPG AFCP MCP
P35579	-0.168700218	1.034339113	MYH9

P05198	-0.167747498	0.815004047	EIF2S1 EIF2A
P23526	-0.167736053	0.845612378	AHCY SAHH
P62380	-0.167734146	0.532643113	TBPL1 TLF TLP TLP21 TRF2 TRP
Q04837	-0.167321205	0.79919802	SSBP1 SSBP
P46779	-0.166687965	0.727190693	RPL28
P62487	-0.166318893	1.563885984	POLR2G RPB7
Q8N357	-0.165986061	0.647624576	SLC35F6 C2orf18 UNQ3047/PRO9863
O43765	-0.165904999	0.659111947	SGTA SGT SGT1
P05114	-0.165892601	0.842029368	HMGN1 HMG14
P10644	-0.165699005	0.31535769	PRKAR1A PKR1 PRKAR1 TSE1
Q9BQ61	-0.165511131	1.549310716	TRIR C19orf43
Q9UKX7	-0.165438652	1.515512692	NUP50 NPAP60L PRO1146
P08708	-0.165367126	1.569491716	RPS17 RPS17L
Q9UJ68	-0.165179253	4.512715614	MSRA
P50454	-0.164990425	0.808297677	SERPINH1 CBP1 CBP2 HSP47 SERPINH2 PIG14
Q9Y3B7	-0.164840698	1.992492436	MRPL11 CGI-113
Q9UBG0	-0.164730072	1.302748913	MRC2 CLEC13E ENDO180 KIAA0709 UPARAP
O14776	-0.164607048	1.878970744	TCERG1 CA150 TAF2S
O75122	-0.164488792	0.858133963	CLASP2 KIAA0627
P07954	-0.164001465	0.385467969	FH
P35270	-0.163428307	1.612058545	SPR
P23193	-0.163413048	1.227724407	TCEA1 GTF2S TFIIS
Q9UDT6	-0.163255692	1.572938211	CLIP2 CYLN2 KIAA0291 WBSCR3 WBSCR4 WSCR4
O94826	-0.16318512	0.725670695	TOMM70 KIAA0719 TOM70 TOMM70A
Q15907	-0.163098335	1.034156673	RAB11B YPT3
P02749	-0.162851334	1.049469361	APOH B2G1
P61353	-0.162707329	0.729140644	RPL27
P49959	-0.162658691	0.80074843	MRE11 HNGS1 MRE11A
Q0VDF9	-0.161875725	2.275416507	HSPA14 HSP60 HSP70L1
P62330	-0.161724091	1.98311486	ARF6
P08047	-0.161418915	1.462850418	SP1 TSFP1
P62873	-0.161417961	1.353348114	GNB1
O75508	-0.160957336	0.489374855	CLDN11 OSP OTM
P43235	-0.160716057	1.453406067	CTSK CTSO CTSO2
P48059	-0.160599709	0.505922963	LIMS1 PINCH PINCH1
Q9Y343	-0.160578251	2.295726079	SNX24 SBBI31 UNQ654/PRO1284
O75934	-0.160518646	0.64576049	BCAS2 DAM1
P15880	-0.160480499	0.712626392	RPS2 RPS4
Q9NUD5	-0.160377502	1.435159219	ZCCHC3 C20orf99
O75533	-0.160355568	1.607190607	SF3B1 SAP155
P10155	-0.160336494	1.400580112	RO60 SSA2 TROVE2

P10599	-0.160203934	0.8055134	TXN TRDX TRX TRX1
Q14966	-0.159949303	1.416057767	ZNF638 NP220 ZFML
Q9BXW6	-0.159920692	0.103308759	OSBPL1A ORP1 OSBP8 OSBPL1 OSBPL1B
Q96A57	-0.15991497	0.302788171	TMEM230 C20orf30 HSPC274 UNQ2432/PRO4992
Q9NW64	-0.15977478	2.075541402	RBM22 ZC3H16 199G4
Q92484	-0.159521103	1.166913157	SMPDL3A ASML3A
Q15436	-0.15944767	1.094745175	SEC23A
Q9Y296	-0.159337521	0.584171297	TRAPPC4 SBDN CGI-104 HSPC172 PTD009
Q9BV57	-0.158955574	2.047326439	ADI1 MTCBP1 HMFT1638
Q9NWM8	-0.158924103	2.125039356	FKBP14 FKBP22 UNQ322/PRO381
P35237	-0.158305168	0.437869824	SERPINB6 PI6 PTI
P30046	-0.157958031	3.044819002	DDT
Q9Y314	-0.157927513	2.795439286	NOSIP CGI-25
Q96CB8	-0.157923698	0.254061625	INTS12 PHF22 SBBI22
P28062	-0.157631874	0.903356294	PSMB8 LMP7 PSMB5i RING10 Y2
P24534	-0.157535553	0.597627987	EEF1B2 EEF1B EF1B
Q9UQ03	-0.157471657	2.64400838	CORO2B KIAA0925
Q14980	-0.157310486	1.79641965	NUMA1 NMP22 NUMA
P13995	-0.156871796	0.665716206	MTHFD2 NMDMC
Q86VP6	-0.156443596	1.35541852	CAND1 KIAA0829 TIP120 TIP120A
O43237	-0.156394005	0.281547784	DYNC1LI2 DNCLI2 LIC2
O43264	-0.156318665	1.547489056	ZW10
Q92610	-0.156084061	0.965973374	ZNF592 KIAA0211
P21333	-0.155939102	1.172261896	FLNA FLN FLN1
Q15365	-0.155755997	1.040320228	PCBP1
P54578	-0.155622482	0.76257249	USP14 TGT
P49916	-0.155479431	0.954412922	LIG3
P31483	-0.155405045	2.260102775	TIA1
O75935	-0.155303955	1.170263085	DCTN3 DCTN22
O94888	-0.15530014	1.325858451	UBXN7 KIAA0794 UBXD7
Q15386	-0.154826164	1.052290016	UBE3C KIAA0010 KIAA10
Q03111	-0.154167175	0.756807913	MLLT1 ENL LTG19 YEATS1
P07858	-0.154082298	1.966234115	CTSB CPSB
Q92797	-0.15404129	1.08725182	SYMPK SPK
Q15021	-0.153884888	1.310826882	NCAPD2 CAPD2 CNAP1 KIAA0159
Q15061	-0.153358459	1.127918124	WDR43 KIAA0007 UTP5
Q9Y5S9	-0.153255463	1.39396542	RBM8A RBM8 HSPC114 MDS014
O95163	-0.153130531	2.304349804	ELP1 IKAP IKBKAP
Q5XKP0	-0.153084755	0.312743814	MICOS13 C19orf70 MIC13 QIL1
Q9H814	-0.153065681	0.65841074	PHAX RNUXA
P54105	-0.15302372	1.319625294	CLNS1A CLCI ICLN

P43307	-0.152850151	0.967632577	SSR1 TRAPA PSEC0262
P53384	-0.15278244	2.020115671	NUBP1 NBP NBP1
P00441	-0.152755737	1.37663895	SOD1
P46777	-0.152623177	0.772908325	RPL5 MSTP030
Q8N5N7	-0.15251255	2.520684491	MRPL50
Q15046	-0.15242672	0.827436734	KARS1 KARS KIAA0070
P32969	-0.152241707	0.463474931	RPL9 OK/SW-cl.103; RPL9P7; RPL9P8; RPL9P9
P60900	-0.152175903	0.536985657	PSMA6 PROS27
Q96EY4	-0.152026176	1.68535845	TMA16 C4orf43
O94966	-0.151817322	1.158755965	USP19 KIAA0891 ZMYND9
Q96B49	-0.15141964	0.66265809	TOMM6 OBTP TOM6
P09455	-0.151319504	1.864512266	RBP1 CRBP1
Q06323	-0.150574684	0.970103383	PSME1 IFI5111
P07108	-0.150538445	0.781146382	DBI
Q9UL03	-0.149774551	1.051825929	INTS6 DBI1 DDX26 DDX26A
P20020	-0.149599075	1.337200876	ATP2B1 PMCA1
O00469	-0.14958477	0.848006392	PLOD2
Q6WKZ4	-0.149494171	0.258463498	RAB11FIP1 RCP
Q01813	-0.149280548	1.013020554	PFKP PFKF
P14866	-0.149167061	1.413707204	HNRNPL HNRPL P/OKcl.14
P53618	-0.148931503	1.722940069	COPB1 COPB MSTP026
Q5VTR2	-0.148808479	1.052874679	RNF20 BRE1A
Q92598	-0.148737907	0.984103939	HSPH1 HSP105 HSP110 KIAA0201
P51531	-0.148088455	1.544578887	SMARCA2 BAF190B BRM SNF2A SNF2L2
P26641	-0.14801693	0.926264118	EEF1G EF1G PRO1608
Q6P5R6	-0.147967339	1.384301374	RPL22L1
P35241	-0.147686958	1.174669323	RDX
Q92572	-0.147644043	2.283360804	AP3S1 CLAPS3
P07195	-0.146974564	0.694653331	LDHB
O75691	-0.146901131	1.001684541	UTP20 DRIM
Q9Y4F3	-0.146302223	2.153785638	MARF1 KIAA0430 LKAP
P52564	-0.146062851	0.877461756	MAP2K6 MEK6 MKK6 PRKMK6 SKK3
Q9NRP0	-0.145789146	1.21243031	OSTC DC2 HDCMD45P HSPC307
O43592	-0.145670891	3.382621055	XPOT
Q9Y676	-0.145405769	0.754393801	MRPS18B C6orf14 HSPC183 PTD017
Q5J8M3	-0.145308495	0.833008075	EMC4 TMEM85 HSPC184 PIG17
Q14318	-0.145300865	1.7225252	FKBP8 FKBP38
Q9Y4L1	-0.145223618	0.890292155	HYOU1 GRP170 ORP150
Q6P2Q9	-0.145201683	2.126450373	PRPF8 PRPC8
P04216	-0.145170212	1.06945711	THY1
Q92785	-0.145133018	0.981708551	DPF2 BAF45D REQ UBID4
Q9BST9	-0.144711494	0.823629329	RTKN RTKN1
Q9P273	-0.144450188	1.647225513	TENM3 KIAA1455 ODZ3 TNM3

Q8WWY3	-0.144407272	1.853125629	PRPF31 PRP31
P06753	-0.144405365	2.773041186	TPM3
Q9C0D5	-0.144166946	0.384732268	TANC1 KIAA1728
P67809	-0.143105507	0.691700636	YBX1 NSEP1 YB1
P42694	-0.142356873	0.520384247	HELZ DRHC KIAA0054
P55145	-0.142037392	1.680079001	MANF ARMET ARP
Q15811	-0.14193821	1.163596009	ITSN1 ITSN SH3D1A
O60256	-0.141793251	1.297266856	PRPSAP2
O00499	-0.14170742	1.740386284	BIN1 AMPHL
Q9H6T3	-0.141607285	1.173345213	RPAP3
Q9UKG1	-0.141498566	1.17908343	APPL1 APPL DIP13A KIAA1428
P60842	-0.141314507	2.13225085	EIF4A1 DDX2A EIF4A
Q12884	-0.141196251	1.273476266	FAP
P54136	-0.141196251	2.521644456	RARS1 RARS
P15311	-0.140986443	1.463101656	EZR VIL2
P31948	-0.140968323	0.41749102	STIP1
Q9UK59	-0.140883446	0.671050345	DBR1
Q7Z7A4	-0.140841007	0.461780618	PXK
Q7Z6Z7	-0.140645027	2.036487474	HUWE1 KIAA0312 KIAA1578 UREB1 HSPC272
P39023	-0.140568733	0.489598147	RPL3 OK/SW-cl.32
Q9Y4G6	-0.140558243	1.225696583	TLN2 KIAA0320
P04040	-0.14029026	1.514443703	CAT
P17096	-0.140237808	0.709083613	HMGA1 HMGIY
Q13418	-0.140217781	0.884092966	ILK ILK1 ILK2
P25788	-0.139834404	0.434985171	PSMA3 HC8 PSC8
Q15648	-0.139637947	0.729099599	MED1 ARC205 CRSP1 CRSP200 DRIP205 DRIP230 PBP PPARBP PPARGBP RB18A TRAP220 TRIP2
Q9Y2Q5	-0.139619827	1.354460355	LAMTOR2 MAPBPIP ROBLD3 HSPC003
P29084	-0.139109612	0.826291365	GTF2E2 TF2E2
Q9Y618	-0.138975143	0.754159327	NCOR2 CTG26
P04150	-0.138619423	1.213074904	NR3C1 GRL
O94830	-0.138554573	0.922885911	DDHD2 KIAA0725 SAMWD1
Q15024	-0.138508797	0.750931833	EXOSC7 KIAA0116 RRP42
Q9UBB4	-0.138484001	1.722667641	ATXN10 SCA10
P61513	-0.138332367	1.205385807	RPL37A
P26373	-0.138207436	0.684631653	RPL13 BBC1 OK/SW-cl.46
P35268	-0.138021469	2.395702576	RPL22
Q9HCD5	-0.137887955	1.844437842	NCOA5 KIAA1637
O76074	-0.1378479	1.184618457	PDE5A PDE5
P23396	-0.137787819	0.437061593	RPS3 OK/SW-cl.26
P55809	-0.13758564	2.413615026	OXCT1 OXCT SCOT
O75781	-0.137483597	3.485645027	PALM KIAA0270

Q5SNT2	-0.137364388	0.470061857	TMEM201 NET5 SAMP1
P50914	-0.137319565	0.645128464	RPL14
P22234	-0.137299538	0.632039334	PAICS ADE2 AIRC PAIS
O43768	-0.137216568	1.124427668	ENSA
O43426	-0.137142658	1.244134108	SYNJ1 KIAA0910
Q9Y5Z4	-0.13671875	1.457815104	HEBP2 C6orf34 SOUL
Q15631	-0.136716843	1.008511635	TSN
P69905	-0.13669014	0.551142849	HBA1; HBA2
Q9HAU0	-0.136479378	1.231685834	PLEKHA5 KIAA1686 PEPP2
Q96AG4	-0.136415482	1.384938405	LRRC59 PRO1855
Q7L2H7	-0.136407852	2.385045155	EIF3M HFLB5 PCID1 GA17 PNAS-125
Q8IUR0	-0.136239052	1.472167406	TRAPPC5
Q13561	-0.136136055	1.411051338	DCTN2 DCTN50
O00273	-0.135769844	1.036650498	DFFA DFF1 DFF45 H13
Q96D71	-0.135703087	0.785614917	REPS1
P09543	-0.135401726	0.727058249	CNP
P61604	-0.135394096	1.541751897	HSPE1
Q92878	-0.135201454	1.68086757	RAD50
O95674	-0.135046005	0.266653102	CDS2
P00734	-0.13498497	1.24086996	F2
Q9BZK7	-0.134795189	0.703300777	TBL1XR1 IRA1 TBLR1
Q9NPQ8	-0.134498596	1.874407673	RIC8A
P31323	-0.134467125	1.814137974	PRKAR2B
Q9NUQ6	-0.134104729	1.243890783	SPATS2L DNAPTP6 SP1224
P43304	-0.134099007	1.633524385	GPD2
Q9NTK5	-0.133687973	1.089982239	OLA1 GTPBP9 PRO2455 PTD004
Q13641	-0.133680344	1.378863151	TPBG 5T4
Q8WUU5	-0.133464336	0.347753901	GATAD1 ODAG
P02649	-0.133336067	0.79870907	APOE
Q14192	-0.133296013	0.849610287	FHL2 DRAL SLIM3
A1X283	-0.133150101	0.79062969	SH3PXD2B FAD49 KIAA1295 TKS4
Q14258	-0.13312912	1.629685415	TRIM25 EFP RNF147 ZNF147
Q16851	-0.132782936	0.593617964	UGP2 UGP1
Q8WXH0	-0.132657051	0.220538343	SYNE2 KIAA1011 NUA
P61758	-0.132479668	0.272582041	VBP1 PFDN3
Q86V81	-0.132410049	2.128158699	ALYREF ALY BEF THOC4
P35221	-0.132356644	0.835475111	CTNNA1
P53367	-0.13222599	2.305052481	ARFIP1
Q9Y4F1	-0.132089615	1.780195819	FARP1 CDEP PLEKHC2
Q9NVP1	-0.131972313	1.311987275	DDX18 cPERP-D
Q01085	-0.131920815	0.452515025	TIAL1
Q12769	-0.131714821	0.91879298	NUP160 KIAA0197 NUP120
Q15599	-0.13160038	0.729984581	SLC9A3R2 NHERF2

Q9BY67	-0.131592751	1.429152054	CADM1 IGSF4 IGSF4A NECL2 SYNCAM TSLC1
P55769	-0.131515503	0.876789729	SNU13 NHP2L1
P49721	-0.131485939	0.249052647	PSMB2
P02774	-0.131448746	1.31537346	GC
P61313	-0.131443024	0.853883269	RPL15 EC45 TCBAP0781
Q01968	-0.131323814	0.610835082	OCRL OCRL1
Q9UGU0	-0.131313801	0.543856272	TCF20 KIAA0292 SPBP
P06730	-0.130877495	1.048224873	EIF4E EIF4EL1 EIF4F
Q5VZ89	-0.130872726	2.335761678	DENND4C C9orf55 C9orf55B
P51610	-0.130847931	2.347055856	HCFC1 HCF1 HFC1
P18124	-0.130639076	0.772599212	RPL7
O60504	-0.130423546	1.547797735	SORBS3 SCAM1
P53597	-0.129954338	1.875349482	SUCLG1
Q9P2I0	-0.129372597	0.922942747	CPSF2 CPSF100 KIAA1367
O60231	-0.12930584	0.980321788	DHX16 DBP2 DDX16 KIAA0577 PRP2
P38646	-0.129289627	0.957253529	HSPA9 GRP75 HSPA9B mt-HSP70
Q96EY8	-0.12924099	0.851093333	MMAB
Q8NB46	-0.12917614	0.73130774	ANKRD52
P19388	-0.128918648	2.794615282	POLR2E
Q13619	-0.128529549	1.645506549	CUL4A
O43615	-0.128477097	1.781596305	TIMM44 MIMT44 TIM44
Q96JM3	-0.128453255	1.109994711	CHAMP1 C13orf8 CAMP CHAMP KIAA1802 ZNF828
Q5VWQ8	-0.128314018	0.747903647	DAB2IP AF9Q34 AIP1 KIAA1743
P30419	-0.12788105	2.310214493	NMT1 NMT
Q9H0X4	-0.127564907	0.9185277	FAM234A C16orf9 ITFG3
Q96C86	-0.127429962	1.129718226	DCPS DCS1 HINT5 HSPC015
Q96P70	-0.127099991	1.835827955	IPO9 IMP9 KIAA1192 RANBP9 HSPC273
Q01780	-0.12700367	1.005294502	EXOSC10 PMSCL PMSCL2 RRP6
Q8IWB9	-0.126965523	0.634084257	TEX2 KIAA1738 TMEM96
P17480	-0.126852036	1.421520786	UBTF UBF UBF1
Q96CW5	-0.126706123	0.825877119	TUBGCP3 GCP3
P01024	-0.12665844	1.477672706	C3 CPAMD1
Q9Y3Q8	-0.126653671	0.675369069	TSC22D4 THG1 TILZ2
Q32MZ4	-0.126607895	2.340658202	LRRFIP1 GCF2 TRIP
O15056	-0.126478195	1.417699345	SYNJ2 KIAA0348
Q5TC82	-0.126455784	0.340426634	RC3H1 KIAA2025 RNF198
O95352	-0.12637043	1.272972276	ATG7 APG7L
P24539	-0.126213074	1.128741662	ATP5PB ATP5F1
Q9NRN5	-0.126173019	1.154863973	OLFML3 PSEC0035 PSEC0173 PSEC0244 UNQ663/PRO1294
Q05193	-0.125969887	1.361910084	DNM1 DNM
Q14151	-0.125890732	0.551163802	SAFB2 KIAA0138

Q6GQQ9	-0.124901772	0.737194893	OTUD7B ZA20D1
P78330	-0.124684334	1.386022671	PSPH
O43684	-0.124630928	1.38631099	BUB3
P04035	-0.124534607	0.32408721	HMGCR
Q8IWE2	-0.124526978	1.36068562	FAM114A1 NOXP20
P41214	-0.124403954	0.886359772	EIF2D HCA56 LGTN
Q9H0L4	-0.124305725	1.39419275	CSTF2T KIAA0689
Q9UBX3	-0.124077797	0.534849234	SLC25A10 DIC
P01033	-0.124054909	0.823189587	TIMP1 CLGI TIMP
Q96J84	-0.12393856	0.89480019	KIRREL1 KIRREL NEPH1
Q9NYB0	-0.123487473	1.98219802	TERF2IP DRIP5 RAP1 PP8000
P36578	-0.123394012	0.589254059	RPL4 RPL1
P55345	-0.123357773	0.320191729	PRMT2 HMT1 HRMT1L1
Q14332	-0.123313904	1.067285022	FZD2
Q9Y3B3	-0.123206139	1.846254224	TMED7 CGI-109
Q32P28	-0.122795105	2.321949907	P3H1 GROS1 LEPRE1 PSEC0109
Q86UU1	-0.122771263	2.034128244	PHLDB1 KIAA0638 LL5A DLNB07
Q16181	-0.12272644	0.528357347	SEPTIN7 CDC10 SEPT7
P62888	-0.122713089	0.708110066	RPL30
Q9H8M9	-0.122570992	0.441837462	EVA1A FAM176A TMEM166 SP24
P02452	-0.122545242	1.058249581	COL1A1
Q9BRK3	-0.122385025	0.967513753	MXRA8
Q9UJX3	-0.122108459	0.400960858	ANAPC7 APC7
P22087	-0.121945381	0.891396165	FBL FIB1 FLRN
Q92520	-0.121901512	0.485860994	FAM3C ILEI GS3786
O95793	-0.121739388	1.926868714	STAU1 STAU
Q9UNF1	-0.121677399	1.693208193	MAGED2 BCG1
P29466	-0.121541023	1.801353829	CASP1 IL1BC IL1BCE
P63165	-0.121504784	2.56399205	SUMO1 SMT3C SMT3H3 UBL1 OK/SW-cl.43
Q9UN86	-0.121397972	0.915849462	G3BP2 KIAA0660
P62701	-0.121395111	0.943614943	RPS4X CCG2 RPS4 SCAR
Q9Y230	-0.121174812	1.678940118	RUVBL2 INO80J TIP48 TIP49B CGI-46
Q8IU81	-0.120879173	0.816989704	IRF2BP1
Q08170	-0.120770454	3.063802602	SRSF4 SFRS4 SRP75
Q16822	-0.120661736	1.959424417	PCK2 PEPCK2
Q9H089	-0.120410442	0.691493804	LSG1
O75683	-0.120400429	0.795580317	SURF6 SURF-6
P42766	-0.1203022	0.949294653	RPL35
P26022	-0.120203972	1.420871662	PTX3 TNFAIP5 TSG14
Q12816	-0.120124817	0.493137629	TRO KIAA1114 MAGED3
Q96K17	-0.120077133	1.716758589	BTF3L4
Q14019	-0.119837761	0.505112013	COTL1 CLP
P23142	-0.119686127	1.244995024	FBLN1 PP213

Q8IVL0	-0.119328499	0.295250307	NAV3 KIAA0938 POMFIL1 STEERIN3
Q96H79	-0.119289398	0.665645631	ZC3HAV1L C7orf39
P15121	-0.119124413	0.776103442	AKR1B1 ALDR1 ALR2
Q9HD45	-0.118914604	0.971618858	TM9SF3 SMBP UNQ245/PRO282
Q93009	-0.118623734	1.63059217	USP7 HAUSP
P31327	-0.118520737	0.665736579	CPS1
P28161	-0.118489265	1.057710151	GSTM2 GST4
P46782	-0.11824894	0.70494984	RPS5
O43583	-0.118026733	0.642883239	DENR DRP1 H14
Q8NEU8	-0.118017197	1.933070009	APPL2 DIP13B
Q8WYP5	-0.117726326	2.441384587	AHCTF1 ELYS TMBS62 MSTP108
Q9Y315	-0.11764431	1.792145025	DERA CGI-26
P49427	-0.117462158	0.85725757	CDC34 UBCH3 UBE2R1
P09001	-0.117402077	0.934497434	MRPL3 MRL3 RPML3
O60725	-0.117370605	1.291036654	ICMT PCCMT
Q9GZT8	-0.117349625	2.012469819	NIF3L1 ALS2CR1 MDS015 My018
Q13243	-0.116913795	1.258104524	SRSF5 HRS SFRS5 SRP40
O75420	-0.116849899	0.213041705	GIGYF1 CDS2 PERQ1 PP3360
Q9UBR2	-0.116322517	0.486442047	CTSZ
P07339	-0.116226196	0.670939672	CTSD CPSD
Q06830	-0.116127968	1.128722565	PRDX1 PAGA PAGB TDPX2
P12268	-0.115773201	2.658937782	IMPDH2 IMPD2
P46977	-0.115452766	0.619179792	STT3A ITM1 TMC
Q8WVV9	-0.115156174	0.380586257	HNRNPLL HNRPLL SRRF BLOCK24
P52788	-0.115154266	1.138471968	SMS
Q5T4B2	-0.114946365	0.568638466	CERCAM CEECAM1 GLT25D3 KIAA1502
P61964	-0.114933968	1.500769746	WDR5 BIG3
P16333	-0.114892006	1.587709634	NCK1 NCK
Q9NWV4	-0.114761353	2.125828117	CZIB C1orf123
Q8TDB4	-0.11472559	0.31389512	MGARP C4orf49 CESP1 HUMMR OSAP GS3582
Q9UNS2	-0.114685059	0.674460639	COPS3 CSN3
O14744	-0.114532471	1.56520541	PRMT5 HRMT1L5 IBP72 JBP1 SKB1
Q99538	-0.114462852	1.605829811	LGMN PRSC1
P62829	-0.114290237	0.630174842	RPL23
Q969X6	-0.114254951	1.330887711	UTP4 CIRH1A cPERP-E KIAA1988
Q7RTV0	-0.114086151	1.044895353	PHF5A
Q9BUK6	-0.113903046	1.087264859	MSTO1 LST005 SLTP005
P62857	-0.113823891	1.172061187	RPS28
O75694	-0.113770485	1.765710319	NUP155 KIAA0791
O14929	-0.113632202	0.996401071	HAT1 KAT1
Q9H900	-0.11349678	0.435336075	ZWILCH
Q15393	-0.113144875	1.341789787	SF3B3 KIAA0017 SAP130
P30086	-0.11296463	1.064641975	PEBP1 PBP PEBP

O43920	-0.112872124	1.977835761	NDUFS5
Q13601	-0.112856865	1.033151922	KRR1 HRB2
P62424	-0.11265564	0.613539939	RPL7A SURF-3 SURF3
Q8TDZ2	-0.112641335	1.908465853	MICAL1 MICAL NICAL
Q9NXW2	-0.112553596	0.918157523	DNAJB12
P29558	-0.112519264	0.206440518	RBMS1 C2orf12 MSSP MSSP1 SCR2
P29536	-0.112383842	2.021017139	LMOD1
P57740	-0.112296104	1.501347633	NUP107
Q8ND56	-0.112102509	1.406956943	LSM14A C19orf13 FAM61A RAP55 RAP55A
Q8N5K1	-0.111987114	0.723778891	CISD2 CDGSH2 ERIS ZCD2
O95433	-0.111973763	0.824157091	AHSA1 C14orf3 HSPC322
Q93052	-0.111805916	0.494556058	LPP
P30050	-0.111747742	0.619864044	RPL12
Q05707	-0.1117239	0.950152957	COL14A1 UND
P62277	-0.111717224	0.857459055	RPS13
Q9Y6F6	-0.111645699	0.33014746	IRAG1 IRAG JAW1L MRVI1
Q96A19	-0.111593246	0.53738894	CCDC102A
P52943	-0.110957146	0.467681118	CRIP2 CRP2
Q9BRJ2	-0.110880852	1.177747512	MRPL45
Q9BV44	-0.11070919	0.933566239	THUMPD3
O96000	-0.110595703	2.379158539	NDUFB10
P49662	-0.110582352	1.044966189	CASP4 ICH2
Q08623	-0.110563278	0.870861747	PUDP DXF68S1E FAM16AX GS1 HDHD1 HDHD1A
Q99590	-0.110510826	0.563281188	SCAF11 CASP11 SFRS2IP SIP1 SRSF2IP
O75208	-0.110404015	0.988332011	COQ9 C16orf49 HSPC326 PSEC0129
P11177	-0.110394478	2.663177749	PDHB PHE1B
P62753	-0.110245705	0.433077911	RPS6 OK/SW-cl.2
Q14554	-0.110135078	2.097316137	PDIA5 PDIR
Q06265	-0.110016823	2.548337692	EXOSC9 PMSCL1
P52756	-0.109974861	0.774451211	RBM5 H37 LUCA15
P49792	-0.109886169	1.118939758	RANBP2 NUP358
P31040	-0.109790802	0.979943971	SDHA SDH2 SDHF
Q9ULU4	-0.109765053	0.944950512	ZMYND8 KIAA1125 PRKCBP1 RACK7
O60462	-0.109724045	0.825129734	NRP2 VEGF165R2
Q96S66	-0.109693527	1.20007406	CLCC1 KIAA0761 MCLC
P08865	-0.109642982	1.209794782	RPSA LAMBR LAMR1
Q9P0M6	-0.109575272	1.018945012	MACROH2A2 H2AFY2
Q9UPT8	-0.109560966	1.655639113	ZC3H4 C19orf7 KIAA1064
Q7Z406	-0.109525681	0.166938454	MYH14 KIAA2034 FP17425
Q9UBP9	-0.109344482	0.672292867	GULP1 CED6 GULP
O15541	-0.109279633	1.000793906	RNF113A RNF113 ZNF183
Q9NQP4	-0.109178543	0.380533624	PFDN4 PFD4
P11172	-0.109154701	3.521415122	UMPS OK/SW-cl.21

Q6Y1H2	-0.10902977	1.256936849	HACD2 PTPLB
Q9NP58	-0.109003067	0.622395343	ABCB6 MTABC3 PRP UMAT
P02787	-0.108941078	0.574374256	TF PRO1400
Q9H583	-0.108839035	1.047368639	HEATR1 BAP28 UTP10
O00159	-0.108663559	0.526910972	MYO1C
O00291	-0.108615875	1.194166448	HIP1
P52888	-0.108362198	2.308884895	THOP1
O95989	-0.10833168	1.40512001	NUDT3 DIPP DIPP1
O00116	-0.108272552	0.458540063	AGPS AAG5
Q05048	-0.108192444	1.876448258	CSTF1
Q96CU9	-0.108103752	0.513794273	FOXRED1 FP634
Q9H981	-0.107995987	0.40449906	ACTR8 ARP8 INO80N
Q68E01	-0.107925415	0.653485334	INTS3 C1orf193 C1orf60
Q96T51	-0.1079216	2.6429327	RUFY1 RABIP4 ZFYVE12
Q96CP2	-0.107765198	1.660850796	FLYWCH2
P62917	-0.107757568	1.481296019	RPL8
Q9NQ50	-0.107163429	1.753289278	MRPL40 NLVCF URIM
P48047	-0.106851578	0.375770957	ATP5PO ATP5O ATPO
Q9P287	-0.106752396	1.020806275	BCCIP TOK1
P49756	-0.106515884	1.50879731	RBM25 RNPC7
Q9H930	-0.106397152	0.403329283	SP140L
Q9C0B1	-0.106076241	1.099464954	FTO KIAA1752
P61619	-0.105910301	1.191861824	SEC61A1 SEC61A
Q9UIU6	-0.105876923	0.399990094	SIX4
P62910	-0.105467796	1.250305879	RPL32 PP9932
P12270	-0.105463982	1.603653037	TPR
Q8IWA0	-0.105189323	1.130980548	WDR75 UTP17
P98175	-0.104842186	0.497138961	RBM10 DXS8237E GPATC9 GPATCH9 KIAA0122
Q8IX12	-0.104698181	2.247625188	CCAR1 CARP1 DIS
Q9C0C2	-0.104483604	2.316801003	TNKS1BP1 KIAA1741 TAB182
Q13308	-0.104401588	1.131439401	PTK7 CCK4
O75874	-0.104326248	0.60628571	IDH1 PICD
Q9Y6D9	-0.104202271	1.086656696	MAD1L1 MAD1 TXBP181
P62714	-0.104049683	1.454820838	PPP2CB
Q9NP77	-0.103824615	1.704178268	SSU72 HSPC182 PNAS-120
P20839	-0.103710175	1.121563885	IMPDH1 IMPD1
Q96D70	-0.103652	0.176896118	R3HDM4 C19orf22
Q13287	-0.103588104	1.016887678	NMI
P08559	-0.103476524	0.209205359	PDHA1 PHE1A
P62244	-0.103444099	0.301781474	RPS15A OK/SW-cl.82
Q9BXP5	-0.103191376	2.019466673	SRRT ARS2 ASR2
Q8N8R5	-0.103151321	0.696251391	C2orf69
P43034	-0.102684021	2.346977516	PAFAH1B1 LIS1 MDCR MDS PAFAHA

P62851	-0.102405548	1.937409388	RPS25
O95155	-0.102401733	1.7458334	UBE4B HDNB1 KIAA0684 UFD2
Q5TAQ9	-0.102378845	0.603144692	DCAF8 H326 WDR42A
P78537	-0.101970673	0.45979576	BLOC1S1 BLOS1 GCN5L1 RT14
Q13547	-0.101819992	1.207998782	HDAC1 RPD3L1
Q6PJG2	-0.101787567	0.646121774	MIDEAS C14orf117 C14orf43 ELMSAN1
P61927	-0.101442337	0.300242647	RPL37
P25705	-0.101290703	0.470985056	ATP5F1A ATP5A ATP5A1 ATP5AL2 ATPM
P47756	-0.101166725	2.156625875	CAPZB
O60341	-0.10114193	0.826878527	KDM1A AOF2 KDM1 KIAA0601 LSD1
A6NIH7	-0.101132393	0.902780822	UNC119B
Q9BTW9	-0.101081848	1.821044989	TBCD KIAA0988 SSD1 TFCD PP1096
Q8IUR7	-0.100974083	1.258643854	ARMC8 S863-2
Q5T0F9	-0.100943565	1.341710367	CC2D1B KIAA1836
P46736	-0.100916862	0.496412912	BRCC3 BRCC36 C6.1A CXorf53
Q9Y2Q9	-0.100708961	0.976618897	MRPS28 MRPS35 HSPC007
Q14203	-0.100605011	1.129792147	DCTN1
P24666	-0.100513458	1.946061981	ACP1
Q9NVX2	-0.100488663	1.153607281	NLE1 HUSSY-07
P62942	-0.100438118	1.391184453	FKBP1A FKBP1 FKBP12
O75643	-0.100402832	1.369561384	SNRNP200 ASCC3L1 HELIC2 KIAA0788
Q92922	-0.100395203	0.109449196	SMARCC1 BAF155
P25398	-0.100364685	2.130506469	RPS12
Q52LJ0	-0.100056648	0.515305554	FAM98B
O95104	-0.099928856	1.556562115	SCAF4 KIAA1172 SFRS15
P39687	-0.099926949	0.876714984	ANP32A C15orf1 LANP MAPM PHAP1
O43676	-0.099255562	0.950722217	NDUFB3
P17812	-0.098816872	0.558450955	CTPS1 CTPS
Q7Z4H8	-0.098662376	1.091159397	POGLUT3 KDELC2 UNQ1904/PRO4350
P06733	-0.098661423	0.666089565	ENO1 ENO1L1 MBPB1 MPB1
Q92905	-0.098423004	4.391749803	COPS5 CSN5 JAB1
Q9H5N1	-0.098236084	0.827535091	RABEP2 RABPT5B
O15230	-0.098129272	0.959609075	LAMA5 KIAA0533 KIAA1907
Q969T9	-0.098028183	1.030633703	WBP2
O43399	-0.097969055	0.343963055	TPD52L2
Q53E16	-0.097854614	0.23001078	SOWAHC ANKRD57 C2orf26
P36915	-0.097845078	2.136022008	GNL1 HSR1
Q9Y5B0	-0.097784996	0.263891122	CTDP1 FCP1
Q9ULC4	-0.097742081	0.89966835	MCTS1 MCT1
P49773	-0.09764576	0.394227512	HINT1 HINT PKCI1 PRKCNH1
P18583	-0.097585678	1.927090566	SON C21orf50 DBP5 KIAA1019 NREBP HSPC310 HSPC312
P16083	-0.09750843	0.647759475	NQO2 NMOR2

Q9Y262	-0.097074509	0.933060099	EIF3L EIF3EIP EIF3S6IP HSPC021 HSPC025 MSTP005
P30520	-0.097049713	0.982330364	ADSS2 ADSS
Q9HAV7	-0.096499443	0.330976461	GRPEL1 GREPEL1
Q4G0J3	-0.096295357	0.426868127	LARP7 HDCMA18P
P15954	-0.096111298	0.282128384	COX7C
Q9Y277	-0.096040726	0.690965988	VDAC3
Q86VI3	-0.095957756	0.908545748	IQGAP3
Q9NWH9	-0.095946312	0.574059747	SLTM MET
Q6PKG0	-0.095871925	1.193503895	LARP1 KIAA0731 LARP
Q96T60	-0.095846176	0.231931652	PNKP
P57088	-0.095801353	1.437788145	TMEM33 DB83
Q63HN8	-0.095767021	0.717280286	RNF213 ALO17 C17orf27 KIAA1554 KIAA1618 MYSTR
P48444	-0.095761299	0.359765275	ARCN1 COPD
Q8IXQ6	-0.095633507	0.372067004	PARP9 BAL BAL1
Q15382	-0.095609665	0.637108545	RHEB RHEB2
Q9BZG1	-0.095490456	0.385196347	RAB34 RAB39 RAH
Q8IWR0	-0.095422745	0.367199198	ZC3H7A ZC3H7 ZC3HDC7 HSPC055
Q7Z4Q2	-0.095403194	1.264785076	HEATR3
P04004	-0.095387459	1.18540606	VTN
P55287	-0.095385551	0.816080086	CDH11
Q9Y5X3	-0.095333099	1.311992321	SNX5
O43818	-0.095252037	0.831711501	RRP9 RNU3IP2 U355K
P48739	-0.095068932	2.049641972	PITPNB
P30530	-0.095067978	2.562943382	AXL UFO
Q9H8Y8	-0.095057487	0.952875268	GORASP2 GOLPH6
O95573	-0.094969749	2.650134617	ACSL3 ACS3 FACL3 LACS3
Q14644	-0.094967842	1.504224186	RASA3
Q8NAF0	-0.094884872	1.008447577	ZNF579
O43290	-0.094758987	2.216031446	SART1
P61201	-0.094705582	1.436934066	COPS2 CSN2 TRIP15
O15533	-0.094634056	0.282905739	TAPBP NGS17 TAPA
Q16626	-0.094529152	0.381843657	MEA1 MEA
Q9BQ39	-0.094427109	1.529658205	DDX50
Q9Y5M8	-0.094356537	3.60797423	SRPRB PSEC0230
Q9H0D6	-0.094171524	0.924389905	XRN2
P62906	-0.094027519	1.094701561	RPL10A NEDD6
Q9BQF6	-0.093818188	0.310551321	SENP7 KIAA1707 SSP2 SUSP2
O96008	-0.093699455	1.457650653	TOMM40 C19orf1 PEREC1 TOM40
Q9C005	-0.093465805	1.731514137	DPY30
Q9H0A0	-0.093434334	0.901613118	NAT10 ALP KIAA1709
P26006	-0.093349457	1.240884291	ITGA3 MSK18
O94906	-0.093213081	1.609060116	PRPF6 C20orf14

O75506	-0.093190193	0.731457871	HSBP1 HSF1BP
P57076	-0.093003273	0.386515129	CFAP298 C21orf48 C21orf59
Q8N5I4	-0.092995644	0.58645977	DHRSX CXorf11 DHRS5X SDR46C1 SDR7C6 UNQ6508/PRO21433
O75151	-0.092946053	2.044609633	PHF2 CENP-35 KIAA0662
Q16658	-0.092871666	0.977287132	FSCN1 FAN1 HSN SNL
Q9UPY5	-0.092809677	0.793288446	SLC7A11
P24385	-0.092720032	1.269118176	CCND1 BCL1 PRAD1
P61026	-0.092699051	0.187830871	RAB10
O75477	-0.09263134	1.202459414	ERLIN1 C10orf69 KE04 KEO4 SPFH1
P61163	-0.092560768	0.963424191	ACTR1A CTRN1
Q969J3	-0.092432976	1.540984981	BORCS5 LOH12CR1
P34059	-0.092418671	1.14072566	GALNS
O95249	-0.092190742	1.364795335	GOSR1 GS28
Q8WWI1	-0.092129707	1.951660554	LMO7 FBX20 FBXO20 KIAA0858
Q9H496	-0.092113018	0.534727561	TOR1AIP2 IFRG15
Q9UJK0	-0.092078209	0.101100171	TSR3 C16orf42 UND313L
Q8IWA4	-0.092057228	0.405035631	MFN1
P83881	-0.091992378	1.018333299	RPL36A RPL44 GIG15 MIG6
P49841	-0.091935158	2.844767528	GSK3B
Q07020	-0.091834068	0.548133851	RPL18
O15514	-0.091815948	1.541619409	POLR2D
Q16637	-0.091773987	0.354814258	SMN1 SMN SMNT; SMN2 SMNC
P61923	-0.091397285	0.272821345	COPZ1 COPZ CGI-120 HSPC181
Q9NX14	-0.091223717	0.778166512	NDUFB11 UNQ111/PRO1064
Q15043	-0.091172218	1.157236441	SLC39A14 KIAA0062 ZIP14
O43432	-0.09114933	1.115793056	EIF4G3
O76095	-0.091032982	0.679555945	JTB HSPC222
P14209	-0.090743065	0.171372673	CD99 MIC2 MIC2X MIC2Y
O00264	-0.090717316	1.3009588	PGRMC1 HPR6.6 PGRMC
P27348	-0.090679169	0.733867446	YWHAQ
Q8IWZ8	-0.090649605	1.07886711	SUGP1 SF4
Q9BRZ2	-0.090308189	0.51253469	TRIM56 RNF109
P22061	-0.090174675	1.584107453	PCMT1
O75828	-0.090107918	1.115716579	CBR3
Q13228	-0.089929581	0.612650802	SELENBP1 SBP
P11498	-0.089913368	0.844173937	PC
Q14657	-0.0897789	0.105363437	LAGE3 DXS9879E ESO3 ITBA2
P62875	-0.089711189	1.472075402	POLR2L
Q96T58	-0.08937645	1.154544889	SPEN KIAA0929 MINT SHARP
O00584	-0.089306831	0.596854116	RNASET2 RNASE6PL
P11171	-0.089166641	0.734179278	EPB41 E41P
P46939	-0.088675499	1.703736987	UTRN DMDL DRP1
P08133	-0.088486671	0.46738441	ANXA6 ANX6

Q99584	-0.08825016	1.394030481	S100A13
Q16666	-0.088135719	0.770206919	IFI16 IFNGIP1
O75821	-0.087731361	0.517163743	EIF3G EIF3S4
P60891	-0.087683678	0.694962992	PRPS1
Q6AI08	-0.087676525	0.890855131	HEATR6 ABC1
Q16401	-0.087619781	1.641142866	PSMD5 KIAA0072
P14314	-0.087322235	1.177903759	PRKCSH G19P1
Q92597	-0.087262154	0.584584276	NDRG1 CAP43 DRG1 RTP
P46937	-0.087097168	0.862558901	YAP1 YAP65
Q5VZF2	-0.086953163	0.785718898	MBNL2 MBLL MBLL39 MLP1
P19784	-0.086894989	0.63273591	CSNK2A2 CK2A2
Q9Y6I3	-0.086812973	1.697637963	EPN1
P51532	-0.086803436	2.804234225	SMARCA4 BAF190A BRG1 SNF2B SNF2L4
Q9Y6X9	-0.086788177	1.338307478	MORC2 KIAA0852 ZCWCC1
Q8IYB3	-0.086751938	1.462141535	SRRM1 SRM160
P21912	-0.086559296	0.819959198	SDHB SDH SDH1
P49006	-0.086512566	1.491791456	MARCKSL1 MLP MRP
Q9Y3A5	-0.086423874	0.317288285	SBDS CGI-97
Q99497	-0.086266518	2.119954946	PARK7
P24844	-0.086173058	0.690297861	MYL9 MLC2 MRLC1 MYRL2
P07919	-0.086082458	0.62294494	UQCRH
Q96KP4	-0.086045265	1.512485134	CNDP2 CN2 CPGL HEL-S-13 PEPA
Q9NYH9	-0.085976601	1.731394718	UTP6 C17orf40 HCA66 MHAT
P49720	-0.085969925	1.04315044	PSMB3
Q99447	-0.085965157	1.397282214	PCYT2
O75340	-0.085621834	1.026550741	PDCD6 ALG2
Q86YS6	-0.085309982	0.536988352	RAB43 RAB41
Q6UN15	-0.085027695	0.940638946	FIP1L1 FIP1 RHE
O15116	-0.084886551	0.223026826	LSM1 CASM
Q15029	-0.084758759	1.986959964	EFTUD2 KIAA0031 SNRP116
P28066	-0.084756851	0.585395729	PSMA5
Q9ULX3	-0.084732056	0.140645397	NOB1 ART4 NOB1P PSMD8BP1 MSTP158
Q15293	-0.084684372	1.67462417	RCN1 RCN
P17931	-0.084677696	1.023665617	LGALS3 MAC2
Q04917	-0.08447361	0.355729643	YWHAH YWHA1
Q9BPW8	-0.084150314	1.281374409	NIPSNAP1
Q12872	-0.0841465	1.299544228	SFSWAP SFRS8 SWAP
Q9H0F6	-0.08366394	1.180438986	SHARPIN SIPL1 PSEC0216
Q02818	-0.083575249	1.487939563	NUCB1 NUC
O75084	-0.083545685	0.700923417	FZD7
Q9UKU9	-0.083473206	0.181446586	ANGPTL2 ARP2 UNQ170/PRO196
P18206	-0.083409309	0.54561807	VCL
O75970	-0.083393097	0.4368393	MPDZ MUPP1

P25789	-0.083348274	0.459958302	PSMA4 HC9 PSC9
Q5SW79	-0.083239555	1.33445383	CEP170 FAM68A KAB KIAA0470
P56159	-0.083143234	0.639330754	GFRA1 GDNFRA RETL1 TRNR1
P27695	-0.082849503	0.588013724	APEX1 APE APE1 APEX APX HAP1 REF1
Q13098	-0.082779884	0.601554995	GPS1 COPS1 CSN1
Q9Y2X3	-0.082657814	1.910521485	NOP58 NOL5 NOP5 HSPC120
Q12972	-0.082431793	0.506274708	PPP1R8 ARD1 NIPP1
O75165	-0.082396507	1.39499124	DNAJC13 KIAA0678 RME8
Q04760	-0.08234024	0.32073434	GLO1
Q8N573	-0.082257271	0.523985419	OXR1 Nbla00307
Q13155	-0.082132339	0.422798154	AIMP2 JTV1 PRO0992
P61006	-0.081922531	1.750532707	RAB8A MEL RAB8
Q9BRJ6	-0.081790924	0.508744996	C7orf50 FP15621
Q96ST2	-0.081783295	1.629835608	IWS1 IWS1L
P43490	-0.081777573	1.381535309	NAMPT PBEF PBEF1
P60953	-0.081775665	0.857584084	CDC42
Q00613	-0.08156395	0.715185013	HSF1 HSTF1
Q96E11	-0.081505775	0.299329459	MRRF
Q9UHV9	-0.081469536	0.775275238	PFDN2 PFD2 HSPC231
Q6NYC1	-0.081272125	1.251944261	JMJD6 KIAA0585 PSR PTDSR
Q6ZRS2	-0.081218719	2.024630933	SRCAP KIAA0309
Q8TB61	-0.080858231	0.396215372	SLC35B2 PAPST1 PSEC0149
Q969T4	-0.08080101	1.559649179	UBE2E3 UBCE4 UBCH9
Q9NQC3	-0.080738068	0.457267578	RTN4 KIAA0886 NOGO My043 SP1507
Q9ULF5	-0.080675125	0.248820447	SLC39A10 KIAA1265 ZIP10
Q9BY77	-0.080339432	0.764564168	POLDIP3 KIAA1649 PDIP46
P47914	-0.080256462	0.656188511	RPL29
Q8IUI8	-0.080186844	1.365130483	CRLF3 CREME9 CRLM9 CYTOR4 P48
Q8TEW0	-0.080183029	0.363429756	PARD3 PAR3 PAR3A
O95394	-0.080043793	0.933372801	PGM3 AGM1
O94903	-0.079615593	1.671530029	PLPBP PROSC
O15067	-0.079604149	1.06295035	PFAS KIAA0361
P49588	-0.079582214	0.870684473	AARS1 AARS
Q9Y5P6	-0.079199791	3.155853372	GMPPB
Q92552	-0.079167366	0.71439484	MRPS27 KIAA0264
Q9BQG0	-0.078861237	1.105291918	MYBBP1A P160
O15344	-0.078759193	0.541069029	MID1 FXY RNF59 TRIM18 XPRF
Q9UM00	-0.078723907	1.688437838	TMCO1 TMCC4 PNAS-10 PNAS-136 UNQ151/PRO177
Q92626	-0.078644753	1.061170905	PXDN KIAA0230 MG50 PRG2 VPO VPO1
Q96PK6	-0.078475952	1.214288571	RBM14 SIP
O75937	-0.078193665	0.89221229	DNAJC8 SPF31 HSPC315 HSPC331
Q9Y606	-0.078144073	0.215418781	PUS1 PP8985
Q9NXH9	-0.077754021	0.902751023	TRMT1

Q9HAD4	-0.077576637	0.5671051	WDR41 MSTP048
Q9NZN8	-0.07753849	2.302807215	CNOT2 CDC36 NOT2 HSPC131 MSTP046
P12277	-0.077482224	3.10028072	CKB CKBB
P30740	-0.077044487	0.722335748	SERPINB1 ELANH2 MNEI PI2
Q06033	-0.077009201	0.469553957	ITIH3
P40429	-0.076723099	0.402077375	RPL13A
Q15102	-0.076675415	1.12751025	PAFAH1B3 PAFAHG
Q8IWS0	-0.076649666	0.607622212	PHF6 CENP-31 KIAA1823
P62854	-0.0766325	0.467892902	RPS26
P40222	-0.0766325	2.308831768	TXLNA TXLN
O43396	-0.07646656	0.950795516	TXNL1 TRP32 TXL TXNL
P53621	-0.076421738	0.755033041	COPA
Q00796	-0.076306343	0.935175198	SORD
O15213	-0.076173782	1.57809241	WDR46 BING4 C6orf11 FP221
Q7L1Q6	-0.076123238	1.309988237	BZW1 5MP2 BZAP45 KIAA0005
Q13033	-0.075875282	0.804396539	STRN3 GS2NA SG2NA
P52630	-0.07571888	1.174550856	STAT2
Q86U86	-0.075648308	1.069548845	PBRM1 BAF180 PB1
Q13136	-0.075591087	1.024470329	PPFIA1 LIP1
O75882	-0.075584412	0.688445075	ATRN KIAA0548 MGCA
O60870	-0.075565338	0.562026725	KIN BTCD KIN17
O76021	-0.075267792	0.981544888	RSL1D1 CATX11 CSIG PBK1 L12
P49418	-0.074873924	0.898738542	AMPH AMPH1
P60174	-0.074692726	0.340472674	TPI1 TPI
Q96G28	-0.074454308	0.68223933	CFAP36 CCDC104 UNQ163/PRO189
Q9BUQ8	-0.074411392	1.04528693	DDX23
Q99943	-0.074371338	0.84177472	AGPAT1 G15
Q6IE81	-0.07426548	0.086302519	JADE1 KIAA1807 PHF17
Q13485	-0.074160576	0.540820008	SMAD4 DPC4 MADH4
Q9Y3F4	-0.074154854	0.41421258	STRAP MAWD UNRIP
Q13541	-0.073752403	0.518522703	EIF4EBP1
P62070	-0.073660851	1.00492496	RRAS2 TC21
Q13045	-0.073422432	1.707726936	FLII FLIL
O00255	-0.073145866	0.857144651	MEN1 SCG2
Q13435	-0.073033333	1.45272505	SF3B2 SAP145
P41252	-0.072978973	0.340883924	IARS1 IARS
Q14534	-0.07281971	0.767611441	SQLE ERG1
P13489	-0.072760582	1.030539262	RNH1 PRI RNH
Q5T447	-0.072717667	0.677595368	HECTD3
P35222	-0.072690964	1.625472033	CTNNB1 CTNNB OK/SW-cl.35 PRO2286
O60934	-0.072630882	0.891410198	NBN NBS NBS1 P95
P57772	-0.072592735	0.498927606	EEFSEC SELB
Q13907	-0.072590828	1.306563284	IDI1

P48163	-0.072375298	0.8028886	ME1
P18031	-0.072107315	1.920225208	PTPN1 PTP1B
Q9NZ32	-0.07209301	0.822161545	ACTR10 ACTR11 ARP11
Q8N1F7	-0.072080612	0.898545983	NUP93 KIAA0095
Q9BYN8	-0.072068214	1.156565373	MRPS26 C20orf193 RPMS13
Q9Y6R0	-0.072051048	0.583437974	NUMBL
Q9Y2G5	-0.071949959	1.206326315	POFUT2 C21orf80 FUT13 KIAA0958
P08238	-0.071913719	0.655442083	HSP90AB1 HSP90B HSPC2 HSPCB
Q96K21	-0.071798325	0.145856267	ZFYVE19 ANCHR MPFYVE
O15446	-0.071789742	0.625466981	POLR1G ASE1 CAST CD3EAP PAF49
Q15397	-0.071602821	1.174221702	PUM3 cPERP-C KIAA0020 PUF-A XTP5
Q12873	-0.071517944	0.735435757	CHD3
O94855	-0.071167946	0.755636216	SEC24D KIAA0755
Q9H974	-0.071084023	0.27539876	QTRT2 QTRTD1
Q6IA86	-0.070845604	0.664916544	ELP2 STATIP1
Q12904	-0.070696831	1.508784028	AIMP1 EMAP2 SCYE1
O00233	-0.070593834	1.312385555	PSMD9
Q13492	-0.070456505	1.507481801	PICALM CALM
O75947	-0.070410728	0.461984092	ATP5PD ATP5H My032
Q96TA1	-0.070336342	0.507388544	NIBAN2 C9orf88 FAM129B
O95721	-0.070217133	0.858702543	SNAP29
Q00587	-0.069889069	0.536334328	CDC42EP1 BORG5 CEP1 MSE55
O96019	-0.069869995	0.750334141	ACTL6A BAF53 BAF53A INO80K
P21399	-0.069730759	1.169896259	ACO1 IREB1
Q8NI36	-0.069721222	0.575391689	WDR36
P68036	-0.069538116	1.014400439	UBE2L3 UBCE7 UBCH7
Q9ULX6	-0.069535255	1.497475964	AKAP8L NAKAP NAKAP95 HRIHFB2018
P49589	-0.069524765	0.695452205	CARS1 CARS
P31946	-0.069434166	0.388771451	YWHAB
P46776	-0.069386482	0.183059561	RPL27A
P25786	-0.069353104	0.300779305	PSMA1 HC2 NU PROS30 PSC2
Q01469	-0.069313049	0.419769293	FABP5
O95881	-0.069250107	0.942984928	TXNDC12 TLP19 UNQ713/PRO1376
Q9Y3U8	-0.069179535	0.772748321	RPL36
Q9NSY1	-0.069025993	0.462329248	BMP2K BIKE HRIHFB2017
Q9BRT2	-0.068940163	0.167901869	UQCC2 C6orf125 MNF1
Q9UMY1	-0.068886757	0.691280052	NOL7 C6orf90 NOP27
Q06124	-0.068636894	0.690458528	PTPN11 PTP2C SHPTP2
Q9BVQ7	-0.068604469	0.399933692	SPATA5L1
P20618	-0.06847477	0.893959046	PSMB1 PSC5
P30085	-0.06838131	0.110084453	CMPK1 CMK CMPK UCK UMK UMPK
Q01970	-0.068232536	2.395178891	PLCB3
Q9H7Z7	-0.06798172	0.783156385	PTGES2 C9orf15 PGES2

Q9H307	-0.067923546	1.264271259	PNN DRS MEMA
P14543	-0.067354202	1.45196911	NID1 NID
Q8TEA8	-0.067299843	0.949070487	DTD1 C20orf88 DUEB HARS2
Q99439	-0.067286491	0.722365475	CNN2
Q9HBR0	-0.066971779	0.50103587	SLC38A10 PP1744
P31153	-0.06695652	1.463514509	MAT2A AMS2 MATA2
P30153	-0.066929817	0.942492109	PPP2R1A
Q9HBL0	-0.066914558	1.396294928	TNS1 TNS
Q99873	-0.066837311	1.514583695	PRMT1 HMT2 HRMT1L2 IR1B4
P00505	-0.066743851	0.313935636	GOT2 KYAT4
Q86WB0	-0.066653252	4.481127356	ZC3HC1 NIPA HSPC216
P84243	-0.066599846	0.583508945	H3-3A H3.3A H3F3 H3F3A PP781; H3-3B H3.3B H3F3B
O94985	-0.066572666	0.206917277	CLSTN1 CS1 KIAA0911
P62081	-0.06656456	0.991502443	RPS7
Q9BZH6	-0.066512108	0.668169931	WDR11 BRWD2 KIAA1351 WDR15
Q9P2X0	-0.066449165	0.648456918	DPM3
Q8WU79	-0.066423416	0.500130703	SMAP2 SMAP1L
A8MVW0	-0.066328049	0.226658924	FAM171A2
Q71RC2	-0.066262245	0.73695186	LARP4 PP13296
Q14123	-0.066228867	0.660381108	PDE1C
Q9BW92	-0.065971375	0.505127405	TARS2 TARSL1
P17858	-0.065778732	0.918426521	PFKL
Q9HAS0	-0.065667629	0.405623957	C17orf75
O00139	-0.065590858	1.423863504	KIF2A KIF2 KNS2
Q02880	-0.065541267	0.903101952	TOP2B
Q01105	-0.065501213	0.181081427	SET
Q12802	-0.065443993	0.333012807	AKAP13 BRX HT31 LBC
O75027	-0.065358162	0.246315521	ABCB7 ABC7
O76031	-0.065266609	2.116174707	CLPX
P61289	-0.065153122	1.372468615	PSME3
Q99576	-0.065076828	0.147738174	TSC22D3 DSIPI GILZ
P55072	-0.064867973	0.963528572	VCP
Q13952	-0.064852238	0.226675626	NFYC
Q15286	-0.064552307	2.865808141	RAB35 RAB1C RAY
Q5VWZ2	-0.064458847	0.740137494	LYPLAL1
O14893	-0.064314842	0.206658536	GEMIN2 SIP1
Q9H0R4	-0.064249992	0.402486253	HDHD2
Q9HB40	-0.063931465	0.441033273	SCPEP1 RISC SCP1 MSTP034 UNQ265/PRO302
P07900	-0.063858986	0.348358184	HSP90AA1 HSP90A HSPC1 HSPCA
P11413	-0.063785553	0.442520714	G6PD
Q09161	-0.063773155	1.733988597	NCBP1 CBP80 NCBP
O43823	-0.063412666	1.091022006	AKAP8 AKAP95

Q9Y376	-0.063398361	0.873170934	CAB39 MO25 CGI-66
Q9UL40	-0.063345909	0.402828176	ZNF346 JAZ
O00154	-0.063123703	1.906061838	ACOT7 BACH
Q9Y3T9	-0.062978745	1.207633926	NOC2L NIR
P04843	-0.062965393	0.308590128	RPN1
Q99615	-0.062947273	0.907578108	DNAJC7 TPR2 TTC2
Q02790	-0.062825203	1.286312446	FKBP4 FKBP52
P42765	-0.062807083	0.419286091	ACAA2
Q14766	-0.062749863	1.03561487	LTBP1
P32322	-0.062708855	0.704516123	PYCR1
Q8TAT6	-0.062630653	0.201588432	NPLOC4 KIAA1499 NPL4
Q9UQ35	-0.062514305	1.882676591	SRRM2 KIAA0324 SRL300 SRM300 HSPC075
Q9BTC8	-0.062405586	0.292368081	MTA3 KIAA1266
Q8N163	-0.062233925	0.703940714	CCAR2 DBC1 KIAA1967
P27708	-0.06216526	1.348101191	CAD
P62841	-0.062140465	0.500560253	RPS15 RIG
Q5XPI4	-0.062077522	0.396404406	RNF123 KPC1 FP1477
P52948	-0.061606407	1.727900831	NUP98 ADAR2
P57678	-0.061518669	0.936296641	GEMIN4
P05141	-0.061410904	1.090925347	SLC25A5 AAC2 ANT2
P14927	-0.061166763	0.490454691	UQCRB UQBP
Q9H2W6	-0.061153412	0.681891226	MRPL46 C15orf4 LIECG2
Q9NQG1	-0.061065674	0.339952514	MANBAL
Q9NRY5	-0.061039925	0.547100789	FAM114A2 C5orf3
Q04721	-0.060263634	0.309766168	NOTCH2
O00303	-0.060128212	0.197109579	EIF3F EIF3S5
P63244	-0.060041428	0.431973401	RACK1 GNB2L1 HLC7 PIG21
O95292	-0.059929848	0.724590463	VAPB UNQ484/PRO983
O75400	-0.059871674	0.598975618	PRPF40A FBP11 FLAF1 FNBP3 HIP10 HYPA HSPC225
P52434	-0.059861183	1.564840408	POLR2H
Q6ZMZ3	-0.059827805	0.164264475	SYNE3 C14orf139 C14orf49 LINC00341
Q92733	-0.059811592	0.607836079	PRCC TPRC
Q6UXN9	-0.059642792	0.594702425	WDR82 TMEM113 WDR82A UNQ9342/PRO34047
O15254	-0.059529305	0.595754424	ACOX3 BRCOX PRCOX
P06132	-0.059384346	1.12203748	UROD
Q9H4L7	-0.05930233	1.053576264	SMARCAD1 KIAA1122
Q9UNZ2	-0.05916214	0.168491587	NSFL1C UBXN2C
Q8NCN4	-0.059103012	0.114311891	RNF169 KIAA1991
Q86YS7	-0.059041023	0.458776666	C2CD5 CDP138 KIAA0528
O15226	-0.058573723	1.497318234	NKRF ITBA4 NRF
Q8NCA5	-0.05848217	0.691532596	FAM98A

P58546	-0.058364868	1.306956471	MTPN
P46087	-0.05834198	0.750113311	NOP2 NOL1 NSUN1
Q15796	-0.058258057	0.856414688	SMAD2 MADH2 MADR2
Q9NX74	-0.057722092	0.3464637	DUS2 DUS2L
Q5VYK3	-0.057703972	2.002112759	ECPAS ECM29 KIAA0368
Q6P1N9	-0.057580948	0.387678162	TATDN1 CDA11
Q15172	-0.057579041	0.425292709	PPP2R5A
P07737	-0.05735302	0.284625542	PFN1
P61916	-0.057285309	0.199006451	NPC2 HE1
Q96EL3	-0.057257652	1.140069521	MRPL53
P06744	-0.057171822	0.250189542	GPI
Q16881	-0.057092667	0.29155264	TXNRD1 GRIM12 KDRF
O75489	-0.056855202	0.641456727	NDUFS3
O60701	-0.056599617	0.2783748	UGDH
Q9NW82	-0.05652523	0.513166781	WDR70
Q9BVJ6	-0.056491852	0.257366997	UTP14A SDCCAG16
Q8IY37	-0.056474209	0.134749694	DHX37 DDX37 KIAA1517
Q14118	-0.056413651	1.778056031	DAG1
P62328	-0.056090355	0.241152258	TMSB4X TB4X THYB4 TMSB4
O43488	-0.055831909	0.293706997	AKR7A2 AFAR AFAR1 AKR7
Q9P2N6	-0.055809975	0.285542802	KANSL3 KIAA1310 NSL3 PRTD SI1
P06756	-0.055800438	0.360085439	ITGAV MSK8 VNRA VTNR
P62877	-0.055636406	0.158435551	RBX1 RNF75 ROC1
Q9P2F8	-0.055591583	0.341421834	SIPA1L2 KIAA1389
P40425	-0.055571556	0.152877863	PBX2 G17
Q567U6	-0.055550575	0.988893136	CCDC93
P10809	-0.055445671	0.46712682	HSPD1 HSP60
Q9BX67	-0.055109024	0.162405432	JAM3 UNQ859/PRO1868
Q92979	-0.055046082	0.254272134	EMG1 C2F
P25685	-0.055019379	1.999683141	DNAJB1 DNAJ1 HDJ1 HSPF1
Q8ND24	-0.055006981	1.1589742	RNF214
Q99747	-0.054883957	1.364915633	NAPG SNAPG
P82930	-0.054864883	0.347688073	MRPS34
Q14738	-0.054793358	0.556668248	PPP2R5D
P53801	-0.054700851	1.033870335	PTTG1IP C21orf1 C21orf3
O95168	-0.054662704	0.963067219	NDUFB4
P45880	-0.054632187	0.961789934	VDAC2
P84098	-0.054498672	0.479076848	RPL19
Q15819	-0.05440712	0.381421915	UBE2V2 MMS2 UEV2
P13984	-0.054286957	0.367477762	GTF2F2 RAP30
Q86VN1	-0.054193497	0.824522015	VPS36 C13orf9 EAP45 CGI-145
Q15047	-0.054110527	0.343733862	SETDB1 ESET KIAA0067 KMT1E
P22102	-0.053990364	0.648670171	GART PGFT PRGS

Q92600	-0.05398941	0.985953457	CNOT9 RCD1 RQCD1
Q99614	-0.053944588	0.42907432	TTC1 TPR1
Q9H330	-0.053713799	0.173143082	TMEM245 C9orf5
Q9UIV1	-0.05360508	0.258411426	CNOT7 CAF1
Q9NS69	-0.053591728	1.52903325	TOMM22 TOM22
Q9Y3B9	-0.053530693	0.528420455	RRP15 KIAA0507 CGI-115
O14787	-0.053322792	0.355652064	TNPO2
Q15370	-0.053290367	0.889531591	ELOB TCEB2
P0C7P3	-0.05312252	0.842557899	SLFN14
Q53HC9	-0.053025246	0.365515474	EIPR1 TSSC1
P04844	-0.052666664	0.409646315	RPN2
O00534	-0.052612305	0.222205104	VWA5A BCSC1 LOH11CR2A
P09874	-0.052435875	0.591173688	PARP1 ADPRT PPOL
P19474	-0.052402496	0.538864372	TRIM21 RNF81 RO52 SSA1
Q8TD30	-0.052056313	0.740650821	GPT2 AAT2 ALT2
Q00059	-0.051699638	0.751437986	TFAM TCF6 TCF6L2
Q9C0J8	-0.05150795	0.148805976	WDR33 WDC146
Q9UFW8	-0.051413059	0.117712884	CGGBP1 CGGBP
Q8NAV1	-0.051404953	0.231057174	PRPF38A
P00749	-0.051332474	0.205963103	PLAU
P08574	-0.051322937	0.398988191	CYC1
Q63HR2	-0.051136017	0.159259667	TNS2 KIAA1075 TENC1
P13497	-0.050847054	0.363630061	BMP1 PCOLC
P31939	-0.050775528	0.230979566	ATIC PURH OK/SW-cl.86
Q8NBQ5	-0.050532341	0.700317484	HSD17B11 DHRS8 PAN1B SDR16C2 PSEC0029 UNQ207/PRO233
Q07092	-0.050433159	0.451410649	COL16A1 FP1572
Q14684	-0.05032444	1.203849803	RRP1B KIAA0179
Q15388	-0.050189972	0.344254852	TOMM20 KIAA0016
Q9Y678	-0.050177574	0.348684382	COPG1 COPG
Q4V9L6	-0.049882889	0.324412181	TMEM119 PSEC0199 UNQ731/PRO1415
P46100	-0.049882889	0.268119624	ATRX RAD54L XH2
P48634	-0.049724579	0.335561142	PRRC2A BAT2 G2
Q8IY67	-0.049659729	0.566292287	RAVER1 KIAA1978
Q96A35	-0.049659729	1.219995133	MRPL24
P55210	-0.049619675	1.811551346	CASP7 MCH3
Q9Y2E5	-0.049519539	0.374739335	MAN2B2 KIAA0935
P20810	-0.049476624	0.864880253	CAST
P05997	-0.049340248	0.590447296	COL5A2
P35269	-0.049304962	1.755166716	GTF2F1 RAP74
P56385	-0.049218178	0.767782979	ATP5ME ATP5I ATP5K
Q86Y56	-0.049084663	1.004577509	DNAAF5 HEATR2
Q02878	-0.048839569	0.604069002	RPL6 TXREB1
P37198	-0.048771858	0.527932667	NUP62

P62263	-0.048567772	0.366095114	RPS14 PRO2640
Q9NXV6	-0.048555374	0.630187263	CDKN2AIP CARF
Q15751	-0.048422813	0.211284142	HERC1
O75152	-0.04837656	0.376747966	ZC3H11A KIAA0663 ZC3HDC11A
Q9UNP9	-0.04826355	0.711900625	PPIE CYP33
P35625	-0.048251152	0.248643979	TIMP3
Q9Y6E2	-0.04816246	0.252066539	BZW2 5MP1 HSPC028 MSTP017
A6NDG6	-0.047966957	2.206524495	PGP
Q96EE3	-0.047849655	0.283019229	SEH1L SEC13L SEH1
P14923	-0.047758102	2.049505643	JUP CTNNG DP3
P83916	-0.04759407	0.384046609	CBX1 CBX
P14868	-0.047587395	1.251961912	DARS1 DARS PIG40
Q9BV20	-0.047532082	1.044531511	MRI1 MRDI UNQ6390/PRO21135
Q13445	-0.047422409	1.275136384	TMED1 IL1RL1L IL1RL1LG
O15372	-0.047401428	0.500456047	EIF3H EIF3S3
P61803	-0.047323227	0.515771844	DAD1
O94992	-0.047291756	0.643813661	HEXIM1 CLP1 EDG1 HIS1 MAQ1
Q99598	-0.047166824	0.80303153	TSNAX TRAX
O15305	-0.047165871	0.715708929	PMM2
P13804	-0.047151566	0.166789871	ETFA
P47974	-0.047070503	0.137078335	ZFP36L2 ERF2 RNF162C TIS11D
P36404	-0.046839714	0.56432263	ARL2
Q9H444	-0.046642303	0.690978856	CHMP4B C20orf178 SHAX1
P48426	-0.046409607	0.44400669	PIP4K2A PI5P4KA PIP5K2 PIP5K2A
P56192	-0.04638195	0.579971605	MARS1 MARS
Q86X55	-0.046339989	0.830602178	CARM1 PRMT4
Q9BVL2	-0.046318054	0.600782229	NUP58 KIAA0410 NUPL1
Q6IN85	-0.046242714	0.134810109	PPP4R3A KIAA2010 PP4R3A SMEK1 MSTP033
Q9BUV8	-0.046071053	0.855806951	RAB5IF C20orf24 RCAF1 PNAS-11
O14562	-0.045989037	0.258269487	UBFD1 UBPH
Q8TEX9	-0.045700073	0.590488814	IPO4 IMP4B RANBP4
Q86XP3	-0.045332909	1.06409225	DDX42
Q96A49	-0.045221329	0.365447958	SYAP1 PRO3113
O94880	-0.045125008	0.511585423	PHF14 KIAA0783
Q9Y371	-0.04502964	0.589875884	SH3GLB1 KIAA0491 CGI-61
Q9Y6B6	-0.04466629	1.482289746	SAR1B SARA2 SARB
P07951	-0.044361115	0.537405041	TPM2 TMSB
Q9BRP8	-0.044149399	1.007612541	PYM1 PYM WIBG
Q9Y2V2	-0.044115067	0.667061044	CARHSP1
Q9Y5J1	-0.044031143	1.049228237	UTP18 WDR50 CDABP0061 CGI-48
Q9Y6M9	-0.04390049	0.956108686	NDUFB9 LYRM3 UQOR22
Q12788	-0.043740273	0.306138664	TBL3 SAZD
P42285	-0.043717384	1.682316967	MTREX DOB1 KIAA0052 MTR4 SKIV2L2

Q5T8D3	-0.043437004	0.475619231	ACBD5 KIAA1996
Q8NBJ4	-0.043054581	0.205216361	GOLM1 C9orf155 GOLPH2 PSEC0242 UNQ686/PRO1326
Q14934	-0.042938232	0.647961885	NFATC4 NFAT3
O95149	-0.042900085	0.458494203	SNUPN RNUT1 SPN1
Q13114	-0.042856216	0.048536022	TRAF3 CAP1 CRAF1
P35232	-0.042794228	0.318646278	PHB1 PHB
P53611	-0.042642593	0.389342763	RABGGTB GGTB
Q96SK2	-0.04253006	0.179459369	TMEM209
P51687	-0.042133331	0.116886612	SUOX
P23368	-0.041964531	1.847842262	ME2
P29218	-0.041828156	0.564174583	IMPA1 IMPA
Q06787	-0.041797638	0.697322568	FMR1
O00429	-0.041764259	0.214121668	DNM1L DLP1 DRP1
P10301	-0.041630745	0.646181298	RRAS
Q12768	-0.041478157	0.798804131	WASHC5 KIAA0196
P61221	-0.041431427	1.582616675	ABCE1 RLI RNASEL1 RNASELI RNS4I OK/SW-cl.40
O00418	-0.041339874	0.483496623	EEF2K
O95819	-0.04131794	0.727166491	MAP4K4 HGK KIAA0687 NIK
Q9UNM6	-0.04128933	1.054110272	PSMD13
Q13617	-0.041264534	0.88022272	CUL2
Q9BTC0	-0.041202545	0.407515878	DIDO1 C20orf158 DATF1 KIAA0333
O14972	-0.041193008	0.516558578	VPS26C DCRA DSCR3 DSCRA
Q9NVT9	-0.041115761	0.380772766	ARMC1 ARCP
Q13283	-0.040885925	0.206999848	G3BP1 G3BP
O43837	-0.04056263	0.392602928	IDH3B
Q14699	-0.040549278	1.103656934	RFTN1 KIAA0084 MIG2
Q8TEJ3	-0.040519714	0.265944316	SH3RF3 POSH2 SH3MD4
Q9NZL9	-0.040413857	0.238060679	MAT2B TGR MSTP045 Nbla02999 UNQ2435/PRO4995
O75746	-0.040297508	0.997362627	SLC25A12 ARALAR1
O00267	-0.040063858	0.432023362	SUPT5H SPT5 SPT5H
Q9NY93	-0.040029526	0.499148665	DDX56 DDX21 NOH61
Q16531	-0.03991127	0.863049705	DDB1 XAP1
Q9UKN8	-0.039848328	0.575498892	GTF3C4
Q9H8H0	-0.039664268	0.313966068	NOL11 L14
Q9BTZ2	-0.039656639	0.13176333	DHRS4 SDR25C2 UNQ851/PRO1800
P27635	-0.039592743	0.920477479	RPL10 DXS648E QM
Q96SB4	-0.039414406	0.635693837	SRPK1
Q8NDI1	-0.03929615	0.161220937	EHBP1 KIAA0903 NACSIN
Q9H9A5	-0.039288521	0.836432126	CNOT10
O95400	-0.039217949	1.118704221	CD2BP2 KIAA1178
Q92934	-0.039180756	0.156277886	BAD BBC6 BCL2L8

O75083	-0.039155006	0.406340714	WDR1
Q96BP3	-0.038943291	0.433701428	PPWD1 KIAA0073
Q9UNN5	-0.038881302	0.281608785	FAF1 UBXD12 UBXN3A CGI-03
Q15020	-0.038582802	0.695672776	SART3 KIAA0156 TIP110
Q15528	-0.03853035	0.11167146	MED22 SURF5
P33897	-0.038485527	0.446247297	ABCD1 ALD
P14324	-0.038464546	0.513986488	FDPS FPS KIAA1293
Q9UHY8	-0.038459778	0.621948226	FEZ2
P82914	-0.038403511	0.40852604	MRPS15 RPMS15 DC37
P13798	-0.03832531	0.671075646	APEH D3F15S2 D3S48E DNF15S2
Q9UKJ3	-0.038291931	0.237648012	GPATCH8 GPATC8 KIAA0553
O43464	-0.038012028	0.161740942	HTRA2 OMI PRSS25
Q96BZ9	-0.037760735	0.091900527	TBC1D20 C20orf140
Q12962	-0.037736893	0.212282291	TAF10 TAF2A TAF2H TAFII30
Q12849	-0.037413597	0.317705309	GRSF1
P67775	-0.037314415	1.152769718	PPP2CA
Q13330	-0.037248611	0.216254375	MTA1
Q9UKY7	-0.037088394	2.051968741	CDV3 H41
Q14165	-0.036961555	0.676916299	MLEC KIAA0152
Q9UHG3	-0.036827087	0.761202827	PCYOX1 KIAA0908 PCL1 UNQ597/PRO1183
Q3V6T2	-0.036823273	0.288939708	CCDC88A APE GRDN KIAA1212
Q92804	-0.036780357	2.004413594	TAF15 RBP56 TAF2N
Q9NWV8	-0.036730766	0.566333109	BABAM1 C19orf62 MERIT40 NBA1 HSPC142
P17152	-0.036707878	0.534825103	TMEM11 C17orf35 PM1
Q96EK5	-0.036501884	0.266491633	KIFBP KBP KIAA1279 KIF1BP
P62266	-0.036431313	0.859088275	RPS23
Q32P41	-0.036257744	0.347321671	TRMT5 KIAA1393 TRM5
Q9UGV2	-0.03589344	0.235778991	NDRG3
Q96T23	-0.035874367	0.482512871	RSF1 HBXAP XAP8
Q14156	-0.035764694	0.608455653	EFR3A KIAA0143
Q6N022	-0.03570652	0.291618921	TENM4 KIAA1302 ODZ4 TNM4
A0JNW5	-0.03563118	0.489706899	UHRF1BP1L KIAA0701 SHIP164
P52815	-0.03561306	0.103733682	MRPL12 MRPL7 RPML12
O43318	-0.035510063	0.555975191	MAP3K7 TAK1
P05386	-0.035429955	0.313152769	RPLP1 RRP1
Q9H3K6	-0.035086632	0.356632017	BOLA2 BOLA2A My016; BOLA2B
Q14671	-0.035004616	0.809459908	PUM1 KIAA0099 PUMH1
Q6UWP7	-0.034915924	0.676262708	LCLAT1 AGPAT8 ALCAT1 LYCAT UNQ1849/PRO3579
O15212	-0.034873962	0.84767235	PFDN6 HKE2 PFD6
Q96C36	-0.034729958	0.185024632	PYCR2
Q9NY12	-0.034694672	0.146111555	GAR1 NOLA1

Q96HS1	-0.034656525	0.428688562	PGAM5
Q96S44	-0.034628868	0.329878531	TP53RK C20orf64 PRPK
P54819	-0.034565926	0.549604273	AK2 ADK2
Q14185	-0.034432411	0.625093864	DOCK1
Q5EBL8	-0.034386635	0.069811079	PDZD11 AIPP1 PDZK11 PISP HSPC227 UNQ6486/PRO21335
P42785	-0.034326553	0.125757172	PRCP PCP
P04156	-0.034309387	0.346987276	PRNP ALTPRP PRIP PRP
O14735	-0.034090996	0.734145741	CDIPT PIS PIS1
Q9GZN8	-0.033939362	0.402042027	C20orf27
Q9Y3E0	-0.033922195	1.137133848	GOLT1B GCT2 GOT1A CGI-141 HDCMA39P UNQ432/PRO793
Q9NRF8	-0.033892632	0.310621999	CTPS2
Q92890	-0.033805847	0.454868996	UFD1 UFD1L
P55735	-0.033783913	0.145396846	SEC13 D3S1231E SEC13A SEC13L1 SEC13R
P20338	-0.033712387	0.332862101	RAB4A RAB4
Q15007	-0.033644676	0.401592364	WTAP KIAA0105
Q15526	-0.033572197	0.373437381	SURF1 SURF-1
P28074	-0.033360481	0.177211463	PSMB5 LMPX MB1 X
Q13137	-0.033305168	0.429825628	CALCOCO2 NDP52
P30876	-0.033234596	0.899889264	POLR2B
Q9UJX2	-0.033156395	0.748583624	CDC23 ANAPC8
Q7Z7N9	-0.033065796	0.141686474	TMEM179B
Q9HC38	-0.033062935	0.874019365	GLOD4 C17orf25 CGI-150 My027
Q8TAF3	-0.033044815	0.651259023	WDR48 KIAA1449 UAF1
Q14232	-0.03303051	1.193888048	EIF2B1 EIF2BA
Q01804	-0.032974243	0.087668692	OTUD4 HIN-1 KIAA1046
P55039	-0.032924652	0.402625597	DRG2
P51149	-0.032704353	0.216337141	RAB7A RAB7
P46781	-0.032469749	0.698532895	RPS9
Q9Y3L3	-0.032447338	0.320132031	SH3BP1
Q8IVD9	-0.032411575	0.461058334	NUDCD3 KIAA1068
P98095	-0.032333374	0.69477719	FBLN2
P28072	-0.031923294	0.459551978	PSMB6 LMPY Y
O75534	-0.03192234	0.269601459	CSDE1 D1S155E KIAA0885 NRU UNR
O43707	-0.031900406	0.455493282	ACTN4
Q8NB90	-0.031766891	0.435869224	SPATA5 AFG2 SPAF
A1A4S6	-0.031586647	0.732470497	ARHGAP10 GRAF2
A0FGR8	-0.031534195	1.24030174	ESYT2 FAM62B KIAA1228
Q15149	-0.031520844	0.390934524	PLEC PLEC1
P19404	-0.031466484	0.333097333	NDUFV2
P60983	-0.031240463	0.192282829	GMFB
O43181	-0.031092644	0.102520506	NDUFS4

Q9NZW5	-0.030968666	0.413124945	PALS2 MPP6 VAM1
Q15813	-0.030757904	0.171961123	TBCE
P21796	-0.030748367	0.172677902	VDAC1 VDAC
Q9UBU9	-0.030603409	0.950987933	NXF1 TAP
Q7Z7F7	-0.030394554	0.396711967	MRPL55 UNQ5835/PRO19675
Q13162	-0.030333519	0.532548086	PRDX4
Q8TED0	-0.030293465	0.141011171	UTP15
Q9NQS3	-0.030241013	0.068582709	NECTIN3 PRR3 PVRL3
O95453	-0.029834747	0.109559031	PARN DAN
Q9UKD2	-0.0296278	0.630835709	MRTO4 C1orf33 MRT4
Q9P0P0	-0.029553413	0.06858573	RNF181 HSPC238
Q96SY0	-0.029549599	0.058922789	INTS14 C15orf44 VWA9
P18074	-0.029297829	0.014819325	ERCC2 XPD XPDC
P46379	-0.029093742	1.013467572	BAG6 BAT3 G3
O95295	-0.029092789	0.664287832	SNAPIN BLOC1S7 SNAP25BP SNAPAP
Q13510	-0.028985023	0.214656619	ASAH1 ASAH HSD-33 HSD33
Q9GZS3	-0.028909683	0.726132719	WDR61
Q96K58	-0.028833389	0.079578484	ZNF668
Q12800	-0.02871418	0.125867036	TFCP2 LSF SEF
Q9NYL2	-0.028530121	0.231280122	MAP3K20 MLTK ZAK HCCS4
P50213	-0.028526306	0.225598099	IDH3A
Q9BTY7	-0.028524399	0.295195275	HGH1 C8orf30A C8orf30B FAM203A FAM203B
O43150	-0.028374672	0.352666379	ASAP2 DDEF2 KIAA0400
O00567	-0.028357506	0.386499609	NOP56 NOL5A
P22307	-0.028056145	0.169780836	SCP2
Q05086	-0.027982712	0.213447005	UBE3A E6AP EPVE6AP HPVE6A
Q9H845	-0.027911186	0.881242804	ACAD9
P47813	-0.02776432	0.165152451	EIF1AX EIF1A EIF4C
P49356	-0.027748108	0.558314231	FNTB
O60318	-0.027621269	1.343038149	MCM3AP GANP KIAA0572 MAP80
Q9UBQ5	-0.027560234	0.381033747	EIF3K EIF3S12 ARG134 HSPC029 MSTP001 PTD001
Q14764	-0.027356148	0.713313724	MVP LRP
Q8TF68	-0.027228355	0.300001109	ZNF384 CAGH1 CIZ NMP4 TNRC1
P35555	-0.027223587	0.398948084	FBN1 FBN
P09622	-0.027153969	0.092474767	DLD GCSL LAD PHE3
Q14669	-0.027104378	0.449953798	TRIP12 KIAA0045 ULF
Q969S3	-0.027061462	0.318964567	ZNF622 ZPR9
Q9NSK0	-0.027004242	0.14477487	KLC4 KNSL8
P52209	-0.026860237	0.2031106	PGD PGDH
P02545	-0.026725769	0.172119841	LMNA LMN1
Q13107	-0.026377678	0.10971474	USP4 UNP UNPH
O15145	-0.026226044	0.439015789	ARPC3 ARC21

Q96EV8	-0.026002884	0.044528449	DTNBP1 BLOC1S8 My031
Q12830	-0.025995731	0.24589386	BPTF FAC1 FALZ
Q9Y613	-0.025961876	0.579368596	FHOD1 FHOS FHOS1
Q9UHA4	-0.025938988	0.187648265	LAMTOR3 MAP2K1IP1 MAPKSP1 PRO2783
Q96EM0	-0.025767326	0.257521001	L3HYPDH C14orf149
Q9H0G5	-0.025730133	0.085961974	NSRP1 CCDC55 NSRP70
Q14974	-0.025650024	0.170960625	KPNB1 NTF97
Q9UHI6	-0.025474548	0.291655634	DDX20 DP103 GEMIN3
Q9NVM1	-0.025461197	0.070504323	EVA1B C1orf78 FAM176B
Q9H223	-0.025383949	0.51109519	EHD4 HCA10 HCA11 PAST4 FKSG7
P30566	-0.02534771	0.655980848	ADSL AMPS
P78347	-0.025346756	0.569537275	GTF2I BAP135 WBSCR6
Q14697	-0.025274277	0.118230938	GANAB G2AN KIAA0088
Q9UJ70	-0.025235176	0.512709189	NAGK
Q96EK6	-0.025214195	1.145787135	GNPNAT1 GNA1
O15160	-0.02491951	0.671712847	POLR1C POLR1E
Q9NSD9	-0.02482605	0.309777082	FARSB FARSLB FRSB HSPC173
Q9NPI6	-0.024587631	0.109165374	DCP1A SMIF
Q96RQ1	-0.024486542	0.299879252	ERGIC2 ERV41 PTX1 CDA14
Q9UBW8	-0.02437973	0.397572708	COPS7A CSN7A DERP10
P07686	-0.024074554	1.377404368	HEXB HCC7
Q9HB63	-0.023948193	0.079158063	NTN4
Q9NZ43	-0.023932457	0.21250259	USE1 USE1L MDS032
Q9GZL7	-0.023891449	0.08228932	WDR12
P09619	-0.023755074	0.349460008	PDGFRB PDGFR PDGFR1
Q9UNK0	-0.02365303	0.10071448	STX8
Q07866	-0.023646355	0.303144677	KLC1 KLC KNS2
P06576	-0.023591042	0.418476722	ATP5F1B ATP5B ATPMB ATPSB
P19838	-0.023566246	0.333460405	NFKB1
O43447	-0.02342701	0.82307505	PPIH CYP20 CYPH
Q96HW7	-0.023351669	0.615844813	INTS4 MSTP093
P28070	-0.023283005	0.123045448	PSMB4 PROS26
Q9Y3D7	-0.023222923	0.617698924	PAM16 MAGMAS TIM16 TIMM16 CGI-136
O00566	-0.023189545	0.602085997	MPHOSPH10 MPP10
O14880	-0.023177147	0.046772289	MGST3
O00232	-0.023151398	0.284057615	PSMD12
Q5R3I4	-0.023057938	0.18925245	TTC38
Q15019	-0.023030281	0.258939954	SEPTIN2 DIFF6 KIAA0158 NEDD5 SEPT2
P19623	-0.023005486	1.15114772	SRM SPS1 SRML1
Q96C90	-0.022955894	0.132906534	PPP1R14B PLCB3N PNG
Q8IWC1	-0.022892952	0.229531233	MAP7D3 MDP3
P22695	-0.022784233	0.216845802	UQCRC2
Q9UJV9	-0.022749901	0.099185658	DDX41 ABS

O43815	-0.022530556	0.589521619	STRN
P09211	-0.022425652	0.19673459	GSTP1 FAEES3 GST3
Q8N1G2	-0.022384644	0.059072678	CMTR1 FTSJD2 KIAA0082 MTR1
Q9NPD3	-0.022358894	0.367845521	EXOSC4 RRP41 SKI6
P82933	-0.02199173	0.212237022	MRPS9 RPMS9
Q8WUA2	-0.021942139	0.959162003	PPIL4
Q70E73	-0.021713257	0.117273181	RAPH1 ALS2CR18 ALS2CR9 KIAA1681 LPD PREL2 RMO1
Q9NQR4	-0.021588326	1.096640927	NIT2 CUA002
O60232	-0.021366119	0.120358031	ZNRD2 SSSCA1
Q68DK2	-0.021299362	0.112120088	ZFYVE26 KIAA0321
Q12931	-0.020932198	1.296003878	TRAP1 HSP75
Q9BVP2	-0.020884514	0.203584871	GNL3 E2IG3 NS
Q9HB19	-0.020759583	0.074203463	PLEKHA2 TAPP2
Q6IQ22	-0.020698547	0.194098961	RAB12
Q13501	-0.020640373	0.693640265	SQSTM1 ORCA OSIL
P09669	-0.020630836	0.131295412	COX6C
Q9NX63	-0.020628929	0.212169431	CHCHD3 MIC19 MINOS3
Q9HAV4	-0.020547867	0.189364587	XPO5 KIAA1291 RANBP21
A5A3E0	-0.020484924	0.041332929	POTEF A26C1B
Q96MW5	-0.020339012	0.226492514	COG8
Q53H12	-0.020281792	0.382961285	AGK MULK
Q9P299	-0.020234108	0.145283057	COPZ2
Q14152	-0.020232201	0.542568687	EIF3A EIF3S10 KIAA0139
Q9H2V7	-0.0201931	0.180068726	SPNS1 SPIN1 PP20300
P35606	-0.020179749	0.180096361	COPB2
Q9NP66	-0.020144463	0.062877433	HMG20A HMGX1 HMGXB1
Q86WR0	-0.019965172	0.182664727	CCDC25
O43310	-0.019758224	0.092368725	CTIF KIAA0427
Q9BRQ6	-0.019748688	0.039151735	CHCHD6 CHCM1 MIC25
P01130	-0.019741058	0.103229852	LDLR
Q969M3	-0.019687653	0.531278982	YIPF5 FINGER5 YIP1A PP12723 SB140 UNQ3123/PRO10275
Q15542	-0.019429207	0.109753928	TAF5 TAF2D
Q9H446	-0.019251823	0.720280406	RWDD1 DFRP2 CGI-24 PTD013
P62241	-0.019235611	0.12930555	RPS8 OK/SW-cl.83
P57764	-0.019096375	0.103567789	GSDMD DFNA5L GSDMDC1 FKSG10
Q8N612	-0.019036293	0.106254446	FHIP1B C11orf56 FAM160A2 KIAA1759
Q9NRR5	-0.018946648	0.245068337	UBQLN4 C1orf6 CIP75 UBIN
Q9UGP4	-0.018943787	0.156728392	LIMD1
P68104	-0.018897057	0.611650025	EEF1A1 EEF1A EF1A LENG7
Q8NFH4	-0.018749237	0.229977674	NUP37
P29692	-0.018656731	0.382732821	EEF1D EF1D
P01008	-0.018593788	0.235032375	SERPINC1 AT3 PRO0309

Q03154	-0.018458366	0.189980024	ACY1
P47897	-0.018425941	0.227584995	QARS1 QARS
Q8TD43	-0.018296242	0.10854505	TRPM4 LTRPC4
Q8NBZ7	-0.018281937	0.122695685	UXS1 UNQ2538/PRO6079
P00533	-0.018249512	0.564562789	EGFR ERBB ERBB1 HER1
O43314	-0.018099785	0.882651409	PPIP5K2 HISPPD1 KIAA0433 VIP2
Q9HAU5	-0.018074036	0.535201237	UPF2 KIAA1408 RENT2
Q16740	-0.017802238	0.349320777	CLPP
P19525	-0.017742157	0.875283405	EIF2AK2 PKR PRKR
Q15075	-0.017673492	0.209861752	EEA1 ZFYVE2
Q9BX68	-0.017663956	0.172090522	HINT2
P10620	-0.017252922	0.567313217	MGST1 GST12 MGST
O15439	-0.017141342	0.131028949	ABCC4 MOATB MRP4
Q96FJ0	-0.016998291	0.331759778	STAMBPL1 AMSHLP KIAA1373
P23588	-0.01679039	0.277242333	EIF4B
P05026	-0.01666832	0.791610052	ATP1B1 ATP1B
O75884	-0.01663208	0.217639925	RBBP9 BOG RBBP10
Q92544	-0.016543388	0.43741507	TM9SF4 KIAA0255 TUCAP1
Q9HD33	-0.016379356	0.425174896	MRPL47 NCM1 CGI-204
Q15435	-0.016348839	0.172176367	PPP1R7 SDS22
P63220	-0.016282082	0.125760505	RPS21
Q14677	-0.01622963	0.106310711	CLINT1 ENTH EPN4 EPNR KIAA0171
Q9Y512	-0.016104698	0.049469041	SAMM50 SAM50 CGI-51 TRG3
Q14149	-0.016072273	0.024910975	MORC3 KIAA0136 NXP2 ZCWCC3
Q92871	-0.015867233	0.092087007	PMM1 PMMH22
Q9BVC4	-0.01586628	0.035243196	MLST8 GBL LST8
P41743	-0.015860558	0.337649622	PRKCI DXS1179E
Q9NTZ6	-0.015757561	0.442582512	RBM12 KIAA0765 HRIHFB2091
Q9UGR2	-0.015616417	0.316264373	ZC3H7B KIAA1031
P08758	-0.015418053	0.10348721	ANXA5 ANX5 ENX2 PP4
Q8TER5	-0.015415192	0.055643719	ARHGEF40 SOLO
O95302	-0.015361786	0.099668586	FKBP9 FKBP60 FKBP63
Q9NYL9	-0.015350342	0.152826492	TMOD3
Q12805	-0.014951706	0.072938548	EFEMP1 FBLN3 FBNL
Q9H2J4	-0.014606476	0.019878063	PDCL3 PhLP2A VIAF1
Q8TDX7	-0.014508247	0.110471453	NEK7
O43301	-0.01448822	0.141662017	HSPA12A KIAA0417
P68371	-0.014482498	0.170108247	TUBB4B TUBB2C
Q5F1R6	-0.014456749	0.127455446	DNAJC21 DNAJA5
O43172	-0.014365196	0.137042145	PRPF4 PRP4
Q9H4L4	-0.014310837	0.069873534	SENP3 SSP3 SUSP3
Q9NV88	-0.014288902	0.054416543	INTS9 RC74
P51570	-0.014090538	0.079834816	GALK1 GALK

P48735	-0.014084816	0.248557495	IDH2
Q9H4A3	-0.014042854	0.158014502	WNK1 HSN2 KDP KIAA0344 PRKWNK1
Q9HCU5	-0.014040947	0.214589104	PREB SEC12
P51114	-0.013858795	0.049991381	FXR1
Q9P0L0	-0.013791084	0.251526153	VAPA VAP33
Q96AT9	-0.013776779	0.07876534	RPE HUSSY-17
Q13438	-0.013725281	0.357495481	OS9
Q14008	-0.01348877	0.139328141	CKAP5 KIAA0097
P08670	-0.013468742	0.154147933	VIM
Q9H1A4	-0.013450146	0.034345602	ANAPC1 TSG24
O95602	-0.013288498	0.222151427	POLR1A
Q86SF2	-0.013251305	0.109472211	GALNT7
Q9Y4G8	-0.013178825	0.041244158	RAPGEF2 KIAA0313 NRAPGEP PDZGEF1
P18859	-0.013150215	0.110844977	ATP5PF ATP5A ATP5J ATPM
Q8WWC4	-0.013114929	0.159974375	MAIP1 C2orf47
Q8N1G4	-0.012732506	0.207429059	LRRC47 KIAA1185
Q9C040	-0.012633324	0.060340965	TRIM2 KIAA0517 RNF86
Q6FI81	-0.012553215	0.143342459	CIAPIN1 CUA001 PRO0915
O75822	-0.012272835	0.122200083	EIF3J EIF3S1 PRO0391
Q92541	-0.012152672	0.031144483	RTF1 KIAA0252
Q9H9E3	-0.01198864	0.151881269	COG4
Q9UJA5	-0.011913776	0.057518793	TRMT6 KIAA1153 TRM6 CGI-09
O43633	-0.011865616	0.205760467	CHMP2A BC2 CHMP2
P61970	-0.011723518	0.075998027	NUTF2 NTF2
Q99627	-0.011615753	0.117332496	COPS8 CSN8
P39210	-0.011554718	0.209915158	MPV17
Q9BTX1	-0.011376381	0.044481926	NDC1 TMEM48
Q9NY33	-0.011285782	0.14735416	DPP3
Q9NT62	-0.011213303	0.062318816	ATG3 APG3 APG3L
P04626	-0.011080742	0.079895078	ERBB2 HER2 MLN19 NEU NGL
P30040	-0.010807037	0.076044461	ERP29 C12orf8 ERP28
P55957	-0.010614395	0.032550986	BID
P68400	-0.010534286	0.076470827	CSNK2A1 CK2A1
Q9GZP4	-0.010513306	0.055406667	PITHD1 C1orf128 AD039 HT014 PP603
P60468	-0.010498047	0.029039286	SEC61B
P25787	-0.010300636	0.203621289	PSMA2 HC3 PSC3
P78318	-0.010175705	0.066106203	IGBP1 IBP1
Q9NX46	-0.010151863	0.182019071	ADPRS ADPRHL2 ARH3
P63096	-0.010090351	0.038077344	GNAI1
Q6IBS0	-0.010019302	0.326569856	TWF2 PTK9L MSTP011
O00217	-0.009936333	0.094318894	NDUFS8
Q8WWM7	-0.009931564	0.093945924	ATXN2L A2D A2LG A2LP A2RP
P10253	-0.009858131	0.06602208	GAA

Q9NVC6	-0.00976944	0.212834002	MED17 ARC77 CRSP6 DRIP77 DRIP80 TRAP80
Q9NZ09	-0.009362221	0.267052953	UBAP1 NAG20
P48643	-0.009293556	0.043617431	CCT5 CCTE KIAA0098
P40616	-0.009290695	0.100185618	ARL1
P78381	-0.009050369	0.037211354	SLC35A2 UGALT UGT UGTL
Q9UHD8	-0.008854866	0.058791399	SEPTIN9 KIAA0991 MSF SEPT9
Q92614	-0.00860405	0.160101614	MYO18A CD245 KIAA0216 MYSPD7
Q4ADV7	-0.008603573	0.034314944	RIC1 CIP150 KIAA1432
O14579	-0.008353233	0.178572402	COPE
O75592	-0.008298874	0.083102229	MYCBP2 KIAA0916 PAM
Q92882	-0.008191109	0.117745878	OSTF1
Q86YP4	-0.008162498	0.222145889	GATAD2A
P49902	-0.008078575	0.059881266	NT5C2 NT5B NT5CP PNT5
Q9NYK5	-0.007979393	0.260855026	MRPL39 C21orf92 MRPL5 RPML5 MSTP003 PRED22
P10515	-0.007876396	0.077543134	DLAT DLTA
Q8TE73	-0.007834435	0.083907973	DNAH5 DNAHC5 HL1 KIAA1603
O75044	-0.0078125	0.059594462	SRGAP2 ARHGAP34 FNBP2 KIAA0456 SRGAP2A
P11940	-0.007702827	0.048188199	PABPC1 PAB1 PABP PABP1 PABPC2
O95299	-0.007216454	0.102508891	NDUFA10
Q92888	-0.00717926	0.055380661	ARHGEF1
P19174	-0.007053375	0.04037984	PLCG1 PLC1
Q99543	-0.006968498	0.130809828	DNAJC2 MPHOSPH11 MPP11 ZRF1
P01137	-0.006860256	0.035015512	TGFB1 TGFB
Q7Z2T5	-0.006793976	0.03110392	TRMT1L C1orf25 TRM1L MSTP070
O96013	-0.006663322	0.047836682	PAK4 KIAA1142
Q86TI2	-0.006608009	0.402710815	DPP9 DPRP2
P31749	-0.006604195	0.089330649	AKT1 PKB RAC
Q8TBC4	-0.006569862	0.10114543	UBA3 UBE1C
P43897	-0.006556511	0.077706897	TSFM
P35475	-0.00653553	0.024002531	IDUA
P50402	-0.006509781	0.063999026	EMD EDMD STA
Q10567	-0.006496429	0.049306124	AP1B1 ADTB1 BAM22 CLAPB2
P62937	-0.006467819	0.032092526	PPIA CYPA
O60762	-0.006387711	0.123329002	DPM1
O43395	-0.006334305	0.042674293	PRPF3 HPRP3 PRP3
Q9Y2S7	-0.006052971	0.017382711	POLDIP2 PDIP38 POLD4 HSPC017
Q96EU6	-0.00564909	0.024006922	RRP36 C6orf153 HSPC253
O94763	-0.005610466	0.014812234	URI1 C19orf2 NNX3 PPP1R19 RMP URI
P55263	-0.005425453	0.069602742	ADK
P82912	-0.005278587	0.088170749	MRPS11 RPMS11 HCC2
Q9Y6E0	-0.005271912	0.050494962	STK24 MST3 STK3

Q14197	-0.005265236	0.438806572	MRPL58 DS1 ICT1
Q7Z7H8	-0.005264282	0.015696462	MRPL10 MRPL8 RPML8
Q9NRX1	-0.005171776	0.01787376	PNO1
O60306	-0.005036354	0.02252197	AQR KIAA0560
Q9UJW0	-0.004905701	0.084045226	DCTN4
Q9Y2A7	-0.004706383	0.050704481	NCKAP1 HEM2 KIAA0587 NAP1
P61081	-0.004675865	0.078562713	UBE2M UBC12
O14647	-0.004530907	0.035832653	CHD2
Q8IUD2	-0.004441261	0.041580887	ERC1 ELKS KIAA1081 RAB6IP2
Q5JTV8	-0.004415512	0.019521947	TOR1AIP1 LAP1
P30041	-0.004385948	0.152676854	PRDX6 AOP2 KIAA0106
Q9BTE6	-0.004190445	0.050823421	AARSD1
Q15005	-0.004142761	0.013622527	SPCS2 KIAA0102 SPC25
P19387	-0.004117966	0.041907598	POLR2C A-152E5.7
Q99570	-0.004079819	0.048806613	PIK3R4 VPS15
Q6IAA8	-0.00356102	0.032401973	LAMTOR1 C11orf59 PDRO PP7157
Q14CX7	-0.003560066	0.021070986	NAA25 C12orf30 MDM20 NAP1
P30084	-0.003386497	0.011734216	ECHS1
Q92616	-0.003308296	0.085485805	GCN1 GCN1L1 KIAA0219
Q7L2J0	-0.003212929	0.013359719	MEPCE BCDIN3
O95479	-0.003133774	0.077937201	H6PD GDH
Q99832	-0.003076553	0.01294028	CCT7 CCTH NIP7-1
Q5T6F2	-0.003041267	0.023188153	UBAP2 KIAA1491
O95870	-0.002993584	0.016341447	ABHD16A BAT5 G5 NG26 PP199
Q6NXT6	-0.002953529	0.008346288	TAPT1 CMVFR
Q9Y3C8	-0.002934456	0.018810036	UFC1 CGI-126 HSPC155
P02656	-0.002925873	0.006247668	APOC3
Q9UG63	-0.002919197	0.039729305	ABCF2 HUSSY-18
O00203	-0.002652168	0.060058812	AP3B1 ADTB3A
Q8NFZ4	-0.002590179	0.008413064	NLGN2 KIAA1366
P61019	-0.002503395	0.044381412	RAB2A RAB2
Q96JC1	-0.002462387	0.015027584	VPS39 KIAA0770 TLP VAM6
P00558	-0.002386093	0.023046169	PGK1 PGKA MIG10 OK/SW-cl.110
P46783	-0.002248764	0.02055153	RPS10
Q8WXA9	-0.00206852	0.010553247	SREK1 SFRS12 SRRP86
Q13042	-0.001836777	0.015476279	CDC16 ANAPC6
P30038	-0.001820564	0.022837925	ALDH4A1 ALDH4 P5CDH
P38571	-0.001655579	0.010178436	LIPA
O00764	-0.001516342	0.056203034	PDXK C21orf124 C21orf97 PKH PNK PRED79
Q9Y4X5	-0.001483917	0.035177215	ARIH1 ARI MOP6 UBCH7BP HUSSY-27
Q9NQ55	-0.001460075	0.005719006	PPAN BXDC3 SSF1
Q8IX01	-0.001312256	0.002670489	SUGP2 KIAA0365 SFRS14
Q96G21	-0.00092411	0.003265148	IMP4 BXDC4

P53396	-0.000835419	0.005619347	ACLY
P53004	-0.000799179	0.011243248	BLVRA BLVR BVR
Q9Y520	-0.000754356	0.022107512	PRRC2C BAT2D1 BAT2L2 KIAA1096 XTP2
Q9UDY8	-0.000723839	0.00598277	MALT1 MLT
O95340	-0.000220299	0.004691032	PAPSS2 ATPSK2
Q03519	-0.000166416	0.000765293	TAP2 ABCB3 PSF2 RING11 Y1
P15848	-7.15256E-05	0.001687912	ARSB
O14974	-6.48499E-05	0.000865677	PPP1R12A MBS MYPT1
O75663	0.000256538	0.003030958	TIPRL
Q9H3U1	0.000535965	0.004767563	UNC45A SMAP1
Q8WVQ1	0.000662804	0.002650268	CANT1 SHAPY
Q9NVJ2	0.000733376	0.005317992	ARL8B ARL10C GIE1
P23497	0.000847816	0.003473625	SP100
Q13362	0.001114845	0.014594096	PPP2R5C KIAA0044
O14949	0.001125336	0.009963493	UQCRQ
Q96SU4	0.001318932	0.045804347	OSBPL9 ORP9 OSBP4
A0AVT1	0.001493454	0.008716378	UBA6 MOP4 UBE1L2
Q86V85	0.001627922	0.028868193	GPR180 ITR
Q96CN7	0.00174427	0.016750636	ISOC1 CGI-111
P33176	0.001907349	0.014004109	KIF5B KNS KNS1
P22033	0.001948357	0.006431991	MMUT MUT
Q99567	0.002036095	0.026180012	NUP88
Q969M7	0.002206802	0.014023103	UBE2F NCE2
O00479	0.0022192	0.027917808	HMGN4 HMG17L3 NHC
Q99704	0.002279282	0.015569206	DOK1
P51151	0.002427101	0.023068605	RAB9A RAB9
P42126	0.002560616	0.009092458	ECI1 DCI
P09972	0.002580643	0.046227278	ALDOC ALDC
O60271	0.002788544	0.066632992	SPAG9 HSS KIAA0516 MAPK8IP4 SYD1 HLC6
Q9Y5X2	0.002832413	0.025694873	SNX8
P36542	0.002934456	0.014805719	ATP5F1C ATP5C ATP5C1 ATP5CL1
Q03426	0.0029459	0.038476312	MVK
Q96LD4	0.002983093	0.015417808	TRIM47 GOA RNF100
Q8TDX9	0.003120422	0.016820736	PKD1L1 UNQ5785/PRO19563
Q8TEQ6	0.003172874	0.025440861	GEMIN5
O95684	0.003213406	0.011126599	CEP43 FGFR1OP FOP
P12259	0.00331974	0.146185349	F5
Q15814	0.003367424	0.065302654	TBCC
Q969H6	0.003481865	0.119822266	POP5 AD-008 HSPC004 x0003
P53582	0.003549576	0.359504068	METAP1 KIAA0094
Q8N3V7	0.00368309	0.01962085	SYNPO KIAA1029
P48449	0.003883362	0.085524421	LSS OSC
Q8WUH2	0.003932953	0.032418148	TGFBRAP1

Q96JP5	0.004076004	0.016570884	ZFP91 ZNF757 FKSG11
Q92917	0.004095078	0.011682423	GPKOW GPATC5 GPATCH5 T54
Q8N3U4	0.004226685	0.002693193	STAG2 SA2
O75608	0.004324913	0.026405316	LYPLA1 APT1 LPL1
Q9BV79	0.004341125	0.013244982	MECR NBRF1 CGI-63
Q96BJ3	0.004459381	0.177055827	AIDA C1orf80
O95630	0.004502296	0.028117608	STAMBP AMSH
P11233	0.004528046	0.040858818	RALA RAL
O95376	0.004587173	0.020060189	ARIH2 ARI2 TRIAD1 HT005
P15291	0.004602432	0.028129452	B4GALT1 GGTB2
Q7Z3J2	0.004647255	0.016322347	VPS35L C16orf62 101F10.2
P04049	0.004813194	0.041078513	RAF1 RAF
Q93100	0.004871368	0.012134421	PHKB
P17174	0.004909515	0.038170516	GOT1
Q14694	0.004914284	0.078676719	USP10 KIAA0190
Q9UPN4	0.004943848	0.021718299	CEP131 AZI1 KIAA1118
O15127	0.004977226	0.048794469	SCAMP2
Q92621	0.005025864	0.057976007	NUP205 C7orf14 KIAA0225
Q9Y5L4	0.005026817	0.008789468	TIMM13 TIM13B TIMM13A TIMM13B
Q92504	0.005251884	0.059596568	SLC39A7 HKE4 RING5
Q8N0X7	0.005369186	0.15799483	SPART KIAA0610 SPG20 TAHCCP1
Q9Y478	0.00538063	0.056247511	PRKAB1 AMPK
Q9Y4W2	0.005440712	0.093177706	LAS1L MSTP060
Q9NRL2	0.005530357	0.027706407	BAZ1A ACF1 WCRF180 HSPC317
Q9H074	0.005592346	0.064368958	PAIP1
Q7L273	0.005647182	0.018528759	KCTD9
Q9BU89	0.005756378	0.046607077	DOHH HLRC1
P78559	0.005846024	0.056254504	MAP1A MAP1L
Q9NUQ8	0.005954742	0.169888733	ABCF3
P50991	0.006100655	0.028942444	CCT4 CCTD SRB
P39656	0.006251335	0.023672388	DDOST KIAA0115 OST48 OK/SW-cl 45
P56945	0.006261826	0.103022823	BCAR1 CAS CASS1 CRKAS
O43772	0.006420612	0.027378679	SLC25A20 CAC CACT
O60832	0.006591797	0.089252676	DKC1 NOLA4
Q15181	0.006592751	0.086423449	PPA1 IOPPP PP
Q13190	0.006881714	0.110649823	STX5 STX5A
Q8IY81	0.007107735	0.10363694	FTSJ3 SB92
Q8N9B5	0.00711441	0.006651409	JMY
O95967	0.00740242	0.091384579	EFEMP2 FBLN4 UNQ200/PRO226
Q13595	0.00757885	0.115125832	TRA2A
Q8N4C8	0.007799149	0.014221275	MINK1 B55 MAP4K6 MINK YSK2 ZC3
Q86SQ0	0.00833416	0.024184093	PHLDB2 LL5B
Q99848	0.008400917	0.252551342	EBNA1BP2 EBP2

Q99816	0.008756638	0.266206459	TSG101
Q5GLZ8	0.008829117	0.10933203	HERC4 KIAA1593
Q92530	0.008903503	0.384103087	PSMF1
Q9NWX6	0.008962631	0.033054356	THG1L ICF45
Q9BRG1	0.008986473	0.070558896	VPS25 DERP9 EAP20
Q69YQ0	0.009235382	1.589227403	SPECC1L CYTSA KIAA0376
Q15717	0.009392738	0.288429773	ELAVL1 HUR
O00754	0.00963974	0.095469836	MAN2B1 LAMAN MANB
P63151	0.009699821	0.590003698	PPP2R2A
Q9BXK5	0.009747982	0.015194815	BCL2L13 MIL1 CD003
O15143	0.009765625	0.040570629	ARPC1B ARC41
Q9BYT8	0.009984016	0.142352888	NLN AGTBP KIAA1226
O95785	0.00998497	0.058085579	WIZ ZNF803
Q5HYI8	0.009991646	1.001103185	RABL3
Q9BTU6	0.010091782	0.076300941	PI4K2A
P11234	0.010110855	0.173053783	RALB
Q9H9J2	0.010128975	0.044757277	MRPL44
Q9NQ48	0.010165215	0.063261642	LZTFL1
P48960	0.010210037	0.030286955	ADGRE5 CD97
Q9UKG9	0.010315895	0.109143663	CROT COT
P34897	0.010399818	0.104287143	SHMT2
Q99623	0.010581017	0.078331829	PHB2 BAP REA
Q12797	0.010772705	0.311520744	ASPH BAH
Q9NUJ1	0.011123657	0.167008064	ABHD10
Q13425	0.011146545	0.220319449	SNTB2 D16S2531E SNT2B2 SNTL
Q9H3H3	0.011190414	0.956915824	C11orf68 BLES03
P38935	0.011602402	0.257956032	IGHMBP2 SMBP2 SMUBP2
P22670	0.011603355	0.056351166	RFX1
Q96FZ7	0.011681557	0.097337668	CHMP6 VPS20
Q9NQ66	0.011681557	0.049171734	PLCB1 KIAA0581
Q96KG9	0.011767387	0.277445144	SCYL1 CVAK90 GKLP NTKL TAPK TEIF TRAP HT019
Q8WTS6	0.011963844	0.669571675	SETD7 KIAA1717 KMT7 SET7 SET9
Q9BYD6	0.012162209	0.180770196	MRPL1 BM-022
Q14558	0.012247086	0.306127908	PRPSAP1
Q16891	0.012317657	0.143862047	IMMT HMP MIC60 MINOS2 PIG4 PIG52
P63000	0.012340546	0.078875381	RAC1 TC25 MIG5
Q9HCS7	0.012475014	0.046075682	XAB2 HCNP KIAA1177 SYF1 PP3898
Q96A26	0.012481689	0.05070895	FAM162A C3orf28 E2IG5 DC16 FWP001
Q02978	0.012485504	0.09911301	SLC25A11 SLC20A4
Q8NB49	0.012587547	0.092273637	ATP11C ATPIG ATPIQ
Q9UPA5	0.012703896	0.127006279	BSN KIAA0434 ZNF231
O60907	0.012840748	0.050867073	TBL1X TBL1
P13073	0.012856483	0.330468269	COX4I1 COX4

Q9BUR5	0.013163567	0.108829936	APOO FAM121B MIC23 MIC26 My025 UNQ1866/PRO4302
O43252	0.013199806	0.587296113	PAPSS1 ATPSK1 PAPSS
P22413	0.013424873	0.052871307	ENPP1 M6S1 NPPS PC1 PDNP1
P49411	0.013697624	0.210498495	TUFM
P31146	0.013716698	1.441415114	CORO1A CORO1
Q76M96	0.013811111	0.815209849	CCDC80 DRO1 URB HBE245
Q5T5Y3	0.013869286	0.034011377	CAMSAP1
P23284	0.013991356	0.110305572	PPIB CYPB
O00170	0.014012337	0.306008203	AIP XAP2
Q8NBJ5	0.014037132	0.881216746	COLGALT1 GLT25D1 PSEC0241
P28838	0.014054298	0.450381146	LAP3 LAPEP PEPS
O15111	0.014101028	0.04355069	CHUK IKKA TCF16
Q92538	0.014321327	1.177843394	GBF1 KIAA0248
Q99996	0.014340401	0.178129011	AKAP9 AKAP350 AKAP450 KIAA0803
Q8IXH7	0.014719963	0.061954153	NELFCD NELFD TH1 TH1L HSPC130
Q9H0X9	0.014836311	0.096309653	OSBPL5 KIAA1534 OBPH1 ORP5
Q9NR45	0.015051842	0.162496946	NANS SAS
Q9BXJ9	0.015163422	0.567409863	NAA15 GA19 NARG1 NATH TBDN100
P17661	0.015242577	0.157682964	DES
P14735	0.015446663	1.030491742	IDE
Q96DA6	0.015473366	0.098873447	DNAJC19 TIM14 TIMM14
Q9Y2T2	0.015476227	0.747430828	AP3M1
P46940	0.01554966	0.071480893	IQGAP1 KIAA0051
O00442	0.015600204	0.438603411	RTCA RPC RPC1 RTC1 RTCD1
Q9HBH5	0.015641212	0.592148868	RDH14 PAN2 SDR7C4 UNQ529/PRO1072
Q6PGP7	0.015904427	0.132025506	TTC37 KIAA0372
Q7L0Y3	0.01595974	0.134498099	TRMT10C MRPP1 RG9MTD1
Q13868	0.015986443	0.090164362	EXOSC2 RRP4
Q9BRS2	0.01615715	0.16194412	RIOK1 RIO1
P49790	0.016238213	0.105005966	NUP153
Q15050	0.016385078	0.295386397	RRS1 KIAA0112 RRR
Q08209	0.016447067	0.33023041	PPP3CA CALNA CNA
Q9HAN9	0.016526222	0.183668784	NMNAT1 NMNAT
O60508	0.01660347	0.099812879	CDC40 EHB3 PRP17 PRPF17
Q9BQA1	0.01669693	0.315947378	WDR77 MEP50 WD45 HKMT1069 Nbla10071
Q16539	0.016701698	0.076178168	MAPK14 CSBP CSBP1 CSBP2 CSPB1 MXI2 SAPK2A
P01112	0.016878128	0.061738789	HRAS HRAS1
Q8NBI6	0.016921997	0.131235932	XXYLT1 C3orf21 PSEC0251
Q15427	0.016994476	0.063283625	SF3B4 SAP49
Q8NCH0	0.017148972	0.101834226	CHST14 D4ST1 UNQ1925/PRO4400
Q9Y6C9	0.017149925	0.163918263	MTCH2 MIMP HSPC032

Q9HDC9	0.017181396	0.237199572	APMAP C20orf3 UNQ1869/PRO4305
Q96Q11	0.017281532	0.66808902	TRNT1 CGI-47
Q04206	0.017469406	0.536184612	RELA NFKB3
Q96RT7	0.017677784	0.446030922	TUBGCP6 GCP6 KIAA1669
P16435	0.017689705	0.204077955	POR CYPOR
P49821	0.017696381	0.305243676	NDUFV1 UQOR1
Q16527	0.017730713	0.295246461	CSRP2 LMO5 SMLIM
Q8IZQ1	0.017730713	0.185192821	WDFY3 KIAA0993
Q9NPE3	0.017813683	0.356047494	NOP10 NOLA3
Q9NP81	0.01812458	0.149119174	SARS2 SARSM
Q53FT3	0.018156052	0.1672531	HIKESHI C11orf73 HSPC138 HSPC179 HSPC248
Q92665	0.018239021	0.276874306	MRPS31 IMOGN38
Q9NZB2	0.018314362	0.601869889	FAM120A C9orf10 KIAA0183 OSSA
Q8NC54	0.018348694	0.128752502	KCT2 C5orf15 HTGN29
P23434	0.018356323	0.181600087	GCSH
Q86W56	0.01838398	0.358210776	PARG
Q13564	0.018418312	0.399880719	NAE1 APPBP1 HPP1
P48723	0.018439293	0.238705589	HSPA13 STCH
Q9Y285	0.018457413	0.253402404	FARSA FARS FARSL FARSLA
P18440	0.018489838	0.080088748	NAT1 AAC1
Q5T4S7	0.018574715	0.97857459	UBR4 KIAA0462 KIAA1307 RBAF600 ZUBR1
Q9P2E2	0.018591404	0.021748892	KIF17 KIAA1405 KIF3X
Q13084	0.018676758	0.263291858	MRPL28 MAAT1
Q9BV19	0.018689156	0.076310907	C1orf50
P49368	0.018699646	0.16207178	CCT3 CCTG TRIC5
Q8WV24	0.018766403	0.147203828	PHLDA1 PHRIP TDAG51
P84085	0.018844604	0.400732651	ARF5
P38117	0.018931389	0.188629012	ETFB FP585
Q9BXB4	0.018953323	0.211980061	OSBPL11 ORP11 OSBP12
Q9H6Y7	0.018987656	0.089857844	RNF167 LP2254
Q4VC31	0.019231796	0.120168878	MIX23 CCDC58
Q92845	0.019282818	0.028277572	KIFAP3 KIF3AP SMAP
P78417	0.019350052	0.124819758	GSTO1 GSTTLP28
Q9NQG5	0.019587517	0.197679287	RPRD1B C20orf77 CREPT
Q9BW61	0.019684792	0.244916032	DDA1 C19orf58 PCIA1
Q8NBM4	0.019685745	0.284907147	UBAC2 PHGDHL1 PSEC0110
Q9Y646	0.01970768	0.110176233	CPQ LCH1 PGCP
P49750	0.019752502	0.432982322	YLPM1 C14orf170 ZAP3
P07602	0.019837379	0.214881788	PSAP GLBA SAP1
Q9BUL8	0.01991272	0.724393136	PDCD10 CCM3 TFAR15
P82650	0.019962311	0.198643185	MRPS22 C3orf5 RPMS22 GK002
Q96G03	0.020030022	0.139985207	PGM2 MSTP006
Q9BZE1	0.020152092	0.307908753	MRPL37 MRPL2 RPML2 HSPC235

P09417	0.020195007	0.092478129	QDPR DHPR SDR33C1
P08397	0.020201683	0.113520138	HMBS PBGD UPS
Q13620	0.020299911	0.15810816	CUL4B KIAA0695
P51608	0.020311356	0.568633405	MECP2
Q9Y3D9	0.020338058	0.288426829	MRPS23 CGI-138 HSPC329
Q13011	0.020339966	0.138161295	ECH1
Q5T280	0.020402908	0.070160379	SPOUT1 C9orf114
Q6NW29	0.020428658	0.216146446	RWDD4 FAM28A RWDD4A
Q92747	0.020464897	0.163871786	ARPC1A SOP2L
Q9P0U3	0.020641804	0.118832628	SENP1
O75718	0.020829201	0.089385323	CRTAP CASP
P42336	0.021008492	0.929449501	PIK3CA
Q9Y5U2	0.021077156	0.21445092	TSSC4
Q96F10	0.021082878	0.265804761	SAT2 SSAT2
O75369	0.021103859	0.477293705	FLNB FLN1L FLN3 TABP TAP
P27361	0.021271706	0.112782708	MAPK3 ERK1 PRKM3
Q92805	0.021315575	0.405687014	GOLGA1
Q5VY43	0.021398544	0.051227551	PEAR1 MEGF12
Q9UHR6	0.021429539	0.068245568	ZNHIT2 C11orf5
Q96SW2	0.021542549	0.075111519	CRBN AD-006
Q9HC35	0.02159214	0.13355267	EML4 C2orf2 EMAPL4
P53999	0.021648407	0.091742809	SUB1 PC4 RPO2TC1
Q12965	0.021652222	0.354836438	MYO1E MYO1C
Q6ZMP0	0.02172184	0.236782012	THSD4 UNQ9334/PRO34005
O15371	0.021724701	0.165728284	EIF3D EIF3S7
O76071	0.021840096	0.056748802	CIAO1 CIA1 WDR39
Q9HB07	0.021876335	0.522236678	MYG1 C12orf10
Q9Y570	0.021877289	0.182807567	PPME1 PME1 PP2593 PRO0750
Q13200	0.021956444	0.371239941	PSMD2 TRAP2
Q9P2E9	0.021966934	0.284576422	RRBP1 KIAA1398
Q9BV38	0.021986008	0.271780244	WDR18
Q9BZE4	0.02202034	0.183210252	GTPBP4 CRFG NOG1
O43708	0.022096634	0.062127041	GSTZ1 MAAI
Q6P1J9	0.022386551	0.189192424	CDC73 C1orf28 HRPT2
P52758	0.022439957	0.065316964	RIDA HRSP12
Q86Y39	0.022483826	0.181616664	NDUFA11
P62166	0.022545815	0.046614523	NCS1 FLUP FREQ
Q9NXE4	0.022953987	0.246640635	SMPD4 KIAA1418 SKNY
Q9Y2E4	0.023066521	0.146995404	DIP2C KIAA0934
Q99798	0.023107529	0.174194503	ACO2
P02795	0.023121834	0.3634349	MT2A CES1 MT2
Q13596	0.023348808	0.47139707	SNX1
Q6NYC8	0.023359299	0.439224846	PPP1R18 HKMT1098 KIAA1949

P52565	0.023581505	0.162901553	ARHGDIA GDIA1
Q96RT1	0.023583412	0.229040078	ERBIN ERBB2IP KIAA1225 LAP2
O00231	0.023603439	0.318386708	PSMD11
Q9H8M7	0.023645401	0.4822053	MINDY3 C10orf97 CARP DERP5 FAM188A MSTP126 My042
Q03405	0.023690224	0.251039827	PLAUR MO3 UPAR
Q9NXR7	0.023691177	0.42479761	BABAM2 BRCC45 BRE
P25325	0.023749352	0.225750765	MPST TST2
Q99836	0.02382946	0.79474686	MYD88
Q6UVY6	0.023877144	0.79271656	MOXD1 MOX UNQ2493/PRO5780
Q9Y450	0.023933411	1.328306936	HBS1L HBS1 KIAA1038
Q5QJE6	0.023944855	0.419888822	DNTTIP2 ERBP TDIF2
Q8WW01	0.023947716	0.152039325	TSEN15 C1orf19 SEN15
Q9HA77	0.024095535	0.472235849	CARS2 OK/SW-cl.10
Q13557	0.024107933	0.126396433	CAMK2D CAMKD
Q9NRX2	0.024170876	0.247075738	MRPL17 LIP2
Q5T0N5	0.024292946	0.363831486	FNBP1L C1orf39 TOCA1
Q6P2E9	0.024321556	0.286020017	EDC4 HEDLS
Q9P121	0.024341583	0.830110009	NTM IGLON2 NT UNQ297/PRO337
Q9P0V3	0.024487495	0.396367844	SH3BP4 BOG25 EHB10 TTP
Q14562	0.02449131	0.156654286	DHX8 DDX8
P05120	0.024496078	0.271823631	SERPINB2 PAI2 PLANH2
Q9UI30	0.024562836	0.245962577	TRMT112 AD-001 HSPC152 HSPC170
Q6IAN0	0.024688721	0.072174951	DHRS7B SDR32C1 CGI-93 UNQ212/PRO238
Q8IZD4	0.024938583	0.378451562	DCP1B
P00367	0.025094032	0.430973276	GLUD1 GLUD
Q9Y2X7	0.025129318	0.554776307	GIT1
Q96FW1	0.025296211	0.291485492	OTUB1 OTB1 OTU1 HSPC263
Q9P2R3	0.02530098	0.13248398	ANKFY1 ANKHZN KIAA1255
O75964	0.025388718	1.922214518	ATP5MG ATP5L
Q8TCF1	0.025561333	0.174840475	ZFAND1
P61587	0.025835991	0.075475553	RND3 ARHE RHO8 RHOE
Q04323	0.026019096	0.410918549	UBXN1 SAKS1
P62834	0.026107788	0.194559637	RAP1A KREV1
Q04637	0.026125908	0.282719923	EIF4G1 EIF4F EIF4G EIF4GI
P61962	0.026377201	0.186736661	DCAF7 HAN11 WDR68
P78346	0.026406288	0.17383929	RPP30 RNASEP2
Q96JA1	0.026421547	0.111459007	LRIG1 LIG1
Q9UBC2	0.026558876	0.617383248	EPS15L1 EPS15R
Q6PD74	0.026576042	0.57758338	AAGAB
Q9NR31	0.026844978	0.794510018	SAR1A SAR1 SARA SARA1
Q5JPH6	0.026854515	0.187565844	EARS2 KIAA1970
O15084	0.026864052	0.20644046	ANKRD28 KIAA0379

Q9NZ01	0.026974678	0.501032742	TECR GPSN2 SC2
Q5VYS8	0.026979446	0.155474662	TUT7 HS2 KIAA1711 ZCCHC6
Q9Y6D6	0.027247429	0.255678242	ARFGEF1 ARFGEP1 BIG1
Q5VW38	0.027445793	0.577399465	GPR107 KIAA1624 LUSTR1
Q92604	0.027582169	0.325706732	LPGAT1 FAM34A KIAA0205
Q13823	0.027587891	0.198928824	GNL2 NGP1
O75175	0.027594566	0.58304314	CNOT3 KIAA0691 LENG2 NOT3
Q92643	0.027648926	0.202654115	PIGK GPI8
Q9Y5Q9	0.027658463	0.089578991	GTF3C3
Q16795	0.027741432	1.356568078	NDUFA9 NDUFS2L
Q86XL3	0.027988434	0.058357492	ANKLE2 KIAA0692 LEM4
Q9BV73	0.028013706	0.129203684	CEP250 CEP2 CNAP1
Q96JQ0	0.028131485	0.30402459	DCHS1 CDH19 CDH25 FIB1 KIAA1773 PCDH16
Q15084	0.0281353	0.277241378	PDIA6 ERP5 P5 TXNDC7
O15511	0.0284729	0.371100891	ARPC5 ARC16
Q13616	0.028536797	0.459609979	CUL1
Q15345	0.028653145	0.382053234	LRRC41 MUF1 PP7759
Q15008	0.02865696	0.221933862	PSMD6 KIAA0107 PFAAP4
Q9BRK5	0.028830528	0.409662579	SDF4 CAB45 PSEC0034
O95865	0.02884388	0.620788156	DDAH2 DDAH G6A NG30
P28370	0.028906822	0.227379566	SMARCA1 SNF2L SNF2L1
O43847	0.029006958	0.737040022	NRDC NRD1
O75131	0.029105186	0.122815826	CPNE3 CPN3 KIAA0636
Q9Y2P8	0.02911377	0.103825219	RCL1 RNAC RPC2 RPCL1 RTC2 HSPC338
Q92947	0.029273987	0.168847589	GCDH
Q8TB37	0.029310226	0.481903213	NUBPL C14orf127
P55209	0.029341698	0.128558007	NAP1L1 NRP
O60678	0.029497147	0.192842372	PRMT3 HRMT1L3
P42696	0.02954483	0.267436504	RBM34 KIAA0117
Q99575	0.029819489	0.463905608	POP1 KIAA0061
Q9BRF8	0.029892921	0.235429855	CPPED1 CSTP1
Q9ULK4	0.029983044	0.145114955	MED23 ARC130 CRSP3 DRIP130 KIAA1216 SUR2
P17568	0.03010273	1.514779497	NDUFB7
O95478	0.030191422	0.364830691	NSA2 TINP1 HUSSY-29
O00541	0.030399323	0.226637443	PES1
Q8NDH3	0.030405998	0.342061222	NPEPL1 KIAA1974
Q9H9A6	0.030441284	0.320154832	LRRC40
Q9UJ14	0.030455589	0.12520153	GGT7 GGTL3 GGTL5
O43776	0.030511856	0.474386477	NARS1 NARS NRS
Q9Y3B2	0.030619621	0.269872535	EXOSC1 CSL4 CGI-108
O00462	0.030892372	0.130539425	MANBA MANB1
Q9NV06	0.030940056	0.108042085	DCAF13 WDSOF1 HSPC064

Q86U44	0.031017303	0.214017802	METTL3 MTA70
Q96EY1	0.031048775	0.478009473	DNAJA3 HCA57 TID1
Q9Y6K5	0.031087399	0.038459463	OAS3 P/OKcl.4
P46459	0.031324387	1.364104037	NSF
P80404	0.031364918	0.66578179	ABAT GABAT
O60884	0.031414032	0.454340368	DNAJA2 CPR3 HIRIP4
Q9NR19	0.031535149	0.555084595	ACSS2 ACAS2
P16278	0.031604767	0.733118034	GLB1 ELNR1
O75390	0.032037735	0.269196883	CS
Q9Y3A3	0.032086372	1.382595258	MOB4 MOB3 MOBKL3 PHOCN PREI3 CGI-95
Q14353	0.032248497	0.239049251	GAMT
Q8NFQ8	0.032286644	1.123226029	TOR1AIP2 IFRG15 LULL1
O15294	0.032320023	0.153713557	OGT
P41227	0.032384872	0.163987084	NAA10 ARD1 ARD1A TE2
Q8IXM2	0.03246212	0.202290278	BAP18 C17orf49
O76094	0.032550812	0.47406378	SRP72
P50395	0.032625198	0.876775525	GDI2 RABGDIB
Q9UIA9	0.032640457	1.344773069	XPO7 KIAA0745 RANBP16
P62861	0.032780647	0.319121701	FAU
P55884	0.032793045	0.680760134	EIF3B EIF3S9
Q9Y333	0.032834053	0.303065104	LSM2 C6orf28 G7B
Q9Y2R9	0.032865524	1.620084753	MRPS7
Q92974	0.032956123	0.369734809	ARHGEF2 KIAA0651 LFP40
Q76FK4	0.033026218	0.262162618	NOL8 C9orf34 NOP132
Q92620	0.033037186	1.231744248	DHX38 DDX38 KIAA0224 PRP16
Q9H078	0.033056259	0.601067732	CLPB HSP78 SKD3
Q8N201	0.033184052	0.359115158	INTS1 KIAA1440 UNQ1821/PRO3434
Q96JG6	0.033302307	0.476591632	VPS50 CCDC132 KIAA1861
P54652	0.033308983	1.690406624	HSPA2
Q86WJ1	0.033347607	0.133352193	CHD1L ALC1
Q96PE2	0.033364296	0.21396214	ARHGEF17 KIAA0337 TEM4
Q9Y2R0	0.033408165	0.464000003	COA3 CCDC56 MITRAC12 HSPC009
Q7Z3K3	0.033735275	1.807198217	POGZ KIAA0461 SUHW5 ZNF280E ZNF635 Nbla00003
P00568	0.03374958	0.448826711	AK1
Q9Y2B0	0.034097672	0.51034147	CNPY2 MSAP TMEM4 ZSIG9 UNQ1943/PRO4426
Q9BVG9	0.034219742	0.03241553	PTDSS2 PSS2
Q9NUI1	0.034246445	0.351742597	DECR2 PDCR SDR17C1
Q9H6S0	0.034264565	1.84234449	YTHDC2
Q6NXE6	0.034292221	0.561170292	ARMC6
Q13405	0.034309387	0.580566198	MRPL49 C11orf4 NOF1 OK/SW-cl.67
O00399	0.03431797	0.266165968	DCTN6 WS3

Q9Y4D7	0.034321785	0.429353835	PLXND1 KIAA0620
P78316	0.03456974	0.399889253	NOP14 C4orf9 NOL14 RES4-25
Q8WUW1	0.034661293	0.107502369	BRK1 C3orf10 HSPC300 MDS027
P60228	0.034679413	0.1966116	EIF3E EIF3S6 INT6
P50750	0.034708977	0.23241498	CDK9 CDC2L4 TAK
Q969Z3	0.034797668	0.121787087	MTARC2 MARC2 MOSC2
Q8TDN6	0.03499794	0.791893423	BRIX1 BRIX BXDC2
P12081	0.035025597	0.790777831	HARS1 HARS HRS
Q5TCZ1	0.035079002	0.479904307	SH3PXD2A FISH KIAA0418 SH3MD1 TKS5
Q8N0U8	0.035152435	0.634150238	VKORC1L1
Q9NPI1	0.035182953	0.081995138	BRD7 BP75 CELTIX1
Q7Z3C6	0.035190105	0.1508051	ATG9A APG9L1
O75368	0.035232544	1.013911124	SH3BGRL
O14734	0.035589218	0.220853887	ACOT8 ACTEIII PTE1
Q9NUP9	0.035729408	0.258820536	LIN7C MALS3 VELI3
P13797	0.035732269	0.30715284	PLS3
O75695	0.035763741	0.634282596	RP2
O94901	0.035793304	0.22549197	SUN1 KIAA0810 UNC84A
Q6UVK1	0.036367416	1.131118169	CSPG4 MCSP
Q9Y2U8	0.036371231	0.172819517	LEMD3 MAN1
P48729	0.036403656	0.350516177	CSNK1A1
Q96JH7	0.036479473	0.192115079	VCPIP1 KIAA1850 VCIP135
P11310	0.036480904	0.244166568	ACADM
P49585	0.036717415	0.359611325	PCYT1A CTPCT PCYT1
Q6P158	0.036828041	0.449169224	DHX57
P23610	0.036883354	0.433232181	F8A1; F8A2; F8A3
Q8IZ83	0.037134171	1.610088517	ALDH16A1
P56182	0.037242889	0.365780669	RRP1 D21S2056E NNP1 NOP52 RRP1A
P51991	0.037530899	0.503506984	HNRNPA3 HNRPA3
P78371	0.037650108	0.476086166	CCT2 99D8.1 CCTB
P53701	0.037797928	0.162329938	HCCS CCHL
P04632	0.037989616	0.320478373	CAPNS1 CAPN4 CAPNS
Q8IZH2	0.038007736	0.959381627	XRN1 SEP1
O00410	0.038165092	0.587798107	IPO5 KPNB3 RANBP5
P41223	0.038374901	0.484459068	BUD31 EDG2
P61981	0.038398743	0.190356661	YWHAG
Q5T9A4	0.03845787	0.54662281	ATAD3B KIAA1273 TOB3
Q00653	0.038567543	0.154233077	NFKB2 LYT10
O76003	0.038755417	0.924772386	GLRX3 PICOT TXNL2 HUSSY-22
P61086	0.038955688	0.276862015	UBE2K HIP2 LIG
A6NDU8	0.038960457	0.275707608	RIMOC1 C5orf51
P18669	0.039039612	0.718871879	PGAM1 PGAMA CDABP0006
P00390	0.039413452	0.86722485	GSR GLUR GRD1

Q6ICB4	0.039669991	0.157586655	PHETA2 FAM109B
Q8IXT5	0.039855957	0.22623877	RBM12B
P36969	0.039945602	0.498025883	GPX4
Q9UET6	0.040018082	0.863763436	FTSJ1 JM23
Q13563	0.040044785	0.445336902	PKD2 TRPP2
Q7Z434	0.040203094	0.688504242	MAVS IPS1 KIAA1271 VISA
Q8WUM0	0.040343285	0.562555084	NUP133
Q14320	0.040484428	0.611061799	FAM50A DXS9928E HXC26 XAP5
P31930	0.040484428	1.187096142	UQCRC1
Q12923	0.040587425	0.327832977	PTPN13 PNP1 PTP1E PTPL1
Q8TDQ7	0.040588379	0.479444729	GNPDA2 GNP2
Q8IYU8	0.040709496	0.490602147	MICU2 EFHA1
Q5VZE5	0.040813446	0.541018004	NAA35 EGAP MAK10
Q9ULC3	0.040948868	0.232472612	RAB23 HSPC137
Q7L5D6	0.040974617	0.561496578	GET4 C7orf20 CEE TRC35 CGI-20
P53602	0.041034698	1.091795211	MVD MPD
P40227	0.041061401	0.684580388	CCT6A CCT6 CCTZ
Q9P2K6	0.041203022	0.108090233	KLHL42 KIAA1340 KLHDC5
Q9NVH1	0.041370392	0.604718662	DNAJC11
Q96QK1	0.041539192	0.157577089	VPS35 MEM3 TCCCTA00141
Q969N2	0.041750908	1.028627175	PIGT CGI-06 PSEC0163 UNQ716/PRO1379
O95983	0.041825294	0.47142971	MBD3
Q9Y6W3	0.041970253	0.326897871	CAPN7 PALBH
Q9BYG3	0.041978836	1.546229901	NIFK MKI67IP NOPP34
Q9BZV1	0.042011261	1.019151022	UBXN6 UBXD1 UBXDC2
O75976	0.042089462	0.757312307	CPD
P84077	0.042161942	0.390542451	ARF1
Q9H936	0.042196274	0.325030493	SLC25A22 GC1
Q9NZ52	0.042238235	0.280647149	GGA3 KIAA0154
Q9NQW7	0.042294502	0.866074875	XPNPEP1 XPNPEPL XPNPEPL1
Q9ULA0	0.042448044	0.769301119	DNPEP ASPEP DAP
O00178	0.042468071	1.05644839	GTPBP1
Q9NX20	0.042506218	0.390566094	MRPL16 PNAS-111
Q5VW32	0.042562485	1.08985706	BROX BROFTI C1orf58
P55011	0.042691231	0.676346947	SLC12A2 NKCC1
Q8N122	0.042692184	0.283929117	RPTOR KIAA1303 RAPTOR
O43819	0.042843819	0.308934023	SCO2
O60749	0.04304409	1.53947002	SNX2 TRG9
P46108	0.043085098	0.44247834	CRK
Q8IWW6	0.043120384	0.230461265	ARHGAP12
Q7Z460	0.043246269	0.409721801	CLASP1 KIAA0622 MAST1
Q00535	0.043395042	0.606574525	CDK5 CDKN5
P61011	0.04380703	0.372145855	SRP54

Q9BT78	0.043828964	1.189376706	COPS4 CSN4
P13667	0.04391861	0.550097689	PDIA4 ERP70 ERP72
Q96GQ5	0.043951988	1.003752205	RUSF1 C16orf58
P36957	0.044021606	0.156915761	DLST DLTS
Q6P1M0	0.044202805	0.981359176	SLC27A4 ACSVL4 FATP4
P54920	0.044239998	0.958101172	NAPA SNAPA
Q3KQV9	0.04426384	0.262517743	UAP1L1
P00403	0.044462204	1.128774069	MT-CO2 COII COX2 COXII MTCO2
Q13347	0.044504166	0.670308795	EIF3I EIF3S2 TRIP1
Q9BQ70	0.044582367	0.187021138	TCF25 KIAA1049 NULP1 FKSG26
Q69YN4	0.044582844	0.28300259	VIRMA KIAA1429 MSTP054
P13674	0.04459095	0.918642072	P4HA1 P4HA
P61158	0.04484272	0.295570665	ACTR3 ARP3
Q92990	0.044983864	0.610903212	GLMN FAP48 FAP68 VMGLOM
P51116	0.045326233	0.18529417	FXR2 FMR1L2
P16989	0.045355797	0.456624993	YBX3 CSDA DBPA
Q5JTH9	0.04551506	0.825218311	RRP12 KIAA0690
Q8TD19	0.04552269	1.198447421	NEK9 KIAA1995 NEK8 NERCC
O95817	0.045564651	1.313102766	BAG3 BIS
Q9HCE5	0.045851707	0.240064682	METTL14 KIAA1627
Q13895	0.045851707	0.532288215	BYSL ENP1
Q9NVH2	0.045859337	0.368753265	INTS7 C1orf73
Q8NE71	0.046063423	0.977311693	ABCF1 ABC50
Q9BSH4	0.046126366	0.339791383	TACO1 CCDC44 PRO0477
P13473	0.04632473	1.960085646	LAMP2
Q9C0E8	0.046364784	0.97648675	LNPK KIAA1715 LNP
Q9UFG5	0.046390057	0.60757828	C19orf25
O43896	0.046420097	1.188882593	KIF1C KIAA0706
P23786	0.046649933	1.060933743	CPT2 CPT1
Q9UK76	0.046664238	1.258408015	JPT1 ARM2 HN1
P54886	0.046754837	0.36188338	ALDH18A1 GSAS P5CS PYCS
Q9BT22	0.046976089	0.57296864	ALG1 HMAT1 HMT1 PSEC0061 UNQ861/PRO1870
Q7Z6M1	0.047046661	0.614375924	RABEPK RAB9P40
Q12979	0.047133446	0.343409377	ABR
O43716	0.047213554	0.12097461	GATC 15E1.2
Q5TDH0	0.047390938	0.338961404	DDI2
Q9Y2L5	0.047398567	0.735461273	TRAPPC8 KIAA1012
Q9H871	0.04741621	0.063422258	RMND5A
Q9NVR5	0.047424316	0.277730683	DNAAF2 C14orf104 KTU
Q9NR09	0.047513008	0.653262937	BIRC6 KIAA1289
Q6QNY0	0.047513485	0.083226193	BLOC1S3 BLOS3
Q6V0I7	0.047549248	1.322200559	FAT4 CDHF14 FATJ Nbla00548
Q9NQX3	0.047626495	0.203732116	GPHN GPH KIAA1385

O15031	0.047642708	0.774177217	PLXNB2 KIAA0315
Q9BQ52	0.047808647	0.512506192	ELAC2 HPC2
Q9H9C1	0.047839642	0.817965547	VIPAS39 C14orf133 SPE39 VIPAR
Q9Y2I7	0.047920227	0.563397946	PIKFYVE KIAA0981 PIP5K3
Q8N3E9	0.047933578	0.24370195	PLCD3 KIAA1964
P49406	0.047942162	0.720084315	MRPL19 KIAA0104 MRPL15
P78406	0.047950745	1.002843034	RAE1 MRNP41
P17980	0.048575401	0.705485157	PSMC3 TBP1
Q96HD1	0.048624039	0.620100387	CRELD1 CIRRIN UNQ188/PRO214
Q9P035	0.048711777	1.033533784	HACD3 BIND1 PTPLAD1
Q6ZNB6	0.048786163	0.417088736	NFXL1 OZFP
Q13867	0.048927307	1.138313061	BLMH
Q9NWZ3	0.049092293	0.267289054	IRAK4
P26640	0.049506187	0.901606221	VARS1 G7A VARS VARS2
Q7L5N1	0.049721718	1.750390754	COPS6 CSN6 HVIP
P51812	0.049731255	1.896948173	RPS6KA3 ISPK1 MAPKAPK1B RSK2
Q3KQU3	0.04992485	0.784699047	MAP7D1 KIAA1187 PARCC1 RPRC1 PP2464
O00192	0.049969673	0.237561935	ARVCF
P14550	0.050039291	0.810079114	AKR1A1 ALDR1 ALR
Q96PV7	0.050044537	2.251890243	FAM193B IRIZIO KIAA1931
Q13409	0.050085068	0.557783922	DYNC1I2 DNCI2 DNCIC2
P36776	0.050138474	0.598752414	LONP1 PRSS15
P06280	0.050197601	0.681170392	GLA
Q9BZF1	0.050519943	0.792823979	OSBPL8 KIAA1451 ORP8 OSBP10
Q27J81	0.050556183	1.944706618	INF2 C14orf151 C14orf173
Q9UGP8	0.0505867	2.112429299	SEC63 SEC63L
A5YKK6	0.050614357	0.508337158	CNOT1 CDC39 KIAA1007 NOT1 AD-005
Q96Q15	0.050761223	0.521052047	SMG1 ATX KIAA0421 LIP
Q13423	0.050765991	1.25534282	NNT
Q9Y5N5	0.050887108	0.089305843	N6AMT1 C21orf127 HEMK2 KMT9 PRED28
Q8TAQ2	0.051057816	0.520419164	SMARCC2 BAF170
Q6VY07	0.05109024	0.736905852	PACS1 KIAA1175
Q71UM5	0.05120182	0.122868254	RPS27L
P14174	0.051233292	0.662564807	MIF GLIF MMIF
Q96S59	0.051254272	1.033621841	RANBP9 RANBPM
P15170	0.051255226	0.453946006	GSPT1 ERF3A
Q8N3C0	0.051291466	0.966889546	ASCC3 HELIC1 RQT2
P14618	0.05163002	1.470154985	PKM OIP3 PK2 PK3 PKM2
Q6ZXV5	0.051745415	1.01483988	TMTC3
O14772	0.05179882	0.257574739	FPGT GFPP
P56556	0.051851273	0.668607925	NDUFA6 LYRM6 NADHB14
Q99714	0.051994324	1.721317076	HSD17B10 ERAB HADH2 MRPP2 SCHAD SDR5C1 XH98G2

Q9H2U1	0.05212307	1.232534777	DHX36 DDX36 KIAA1488 MLEL1 RHAU
Q9NVM9	0.052219391	0.451029428	INTS13 ASUN C12orf11 GCT1
O00743	0.052256584	1.711839215	PPP6C PPP6
Q96CC6	0.052614212	0.282178294	RHBDF1 C16orf8 DIST1 IRHOM1
O14653	0.052786827	0.85513783	GOSR2 GS27
Q96EQ0	0.05291748	0.251916693	SGTB SGT2
O15321	0.052951813	0.226642871	TM9SF1
Q66K14	0.053072929	0.311563385	TBC1D9B KIAA0676
P28331	0.05307579	1.657781091	NDUFS1
P04083	0.053086281	0.385954294	ANXA1 ANX1 LPC1
Q9HCE1	0.053107262	0.743250996	MOV10 KIAA1631
Q9UL18	0.053421974	0.247686296	AGO1 EIF2C1
P49770	0.053460121	0.31392368	EIF2B2 EIF2BB
Q6P587	0.053476334	0.612009919	FAHD1 C16orf36 YISKL
O15498	0.053477287	0.886203433	YKT6
Q14653	0.053500175	0.356956188	IRF3
P60510	0.05355835	1.326240019	PPP4C PPP4 PPX
Q9BZF9	0.053741455	2.013936976	UACA KIAA1561
Q9NYV4	0.05374527	0.428946188	CDK12 CRK7 CRKRS KIAA0904
P54725	0.05375576	0.745450787	RAD23A
P16615	0.053797722	0.642079018	ATP2A2 ATP2B
Q9UHA3	0.053841114	0.223229332	RSL24D1 C15orf15 RPL24L My024
Q96GX9	0.053876877	0.557892569	APIP CGI-29
Q6P4I2	0.053879738	0.501600125	WDR73 HSPC264
Q15036	0.053969383	0.290779025	SNX17 KIAA0064
Q9BXY0	0.054347992	0.963732706	MAK16 RBM13
O75116	0.054512024	0.858828833	ROCK2 KIAA0619
Q6ZS11	0.054515839	0.236617958	RINL
Q9Y263	0.054560661	0.794269474	PLAA PLAP
P49354	0.054586411	0.400005226	FNTA
Q8N983	0.054774284	1.224606351	MRPL43
P07437	0.054836273	0.410380433	TUBB TUBB5 OK/SW-cl.56
P68402	0.054898262	0.703169866	PAFAH1B2 PAFAHB
P10768	0.054976463	0.981414043	ESD
Q12888	0.055036545	0.963767579	TP53BP1
Q9UBB6	0.055244446	0.928603554	NCDN KIAA0607
Q53F19	0.055387497	1.153552076	NCBP3 C17orf85
Q96GY0	0.055399895	0.321382552	ZC2HC1A C8orf70 FAM164A CGI-62
Q92783	0.055545807	1.340652746	STAM STAM1
Q02543	0.055569649	0.541931432	RPL18A
Q08AM6	0.055577278	0.977117119	VAC14 TAX1BP2 TRX
Q9UBI1	0.055583	1.01285824	COMMD3 BUP C10orf8
P61009	0.055823326	0.300793799	SPCS3 SPC22 UNQ1841/PRO3567

Q9H2U2	0.055884361	0.785818963	PPA2 HSPC124
P42566	0.055949211	0.290489841	EPS15 AF1P
Q9H2D6	0.056005478	1.199031711	TRIOBP KIAA1662 TARA HRIHFB2122
P61225	0.056154251	0.430278124	RAP2B
Q7Z392	0.056181908	0.823735604	TRAPPC11 C4orf41
P26639	0.056288719	0.555299103	TARS1 TARS
Q9Y4I1	0.056425095	1.741848063	MYO5A MYH12
Q9BTY2	0.056441307	0.318132876	FUCA2 PSEC0151 UNQ227/PRO260
P07741	0.056455612	0.874578685	APRT
O60333	0.056503296	0.887644117	KIF1B KIAA0591 KIAA1448
Q16718	0.05651474	0.740654622	NDUFA5
Q9Y3D0	0.056570053	1.002182913	CIAO2B CIAB FAM96B MIP18 CGI-128 HSPC118
Q9NUQ9	0.056686401	0.56325114	CYRIB CYRI FAM49B BM-009
P17655	0.056785583	0.617321842	CAPN2 CANPL2
Q7Z2W9	0.056880951	0.868682422	MRPL21
Q9BV68	0.056923389	0.219239196	RNF126
Q969Z0	0.05709362	0.960161726	TBRG4 CPR2 FASTKD4 KIAA0948
P51665	0.057155609	1.943086363	PSMD7 MOV34L
P42345	0.057184219	1.757347207	MTOR FRAP FRAP1 FRAP2 RAFT1 RAPT1
Q969G3	0.057394981	0.806516079	SMARCE1 BAF57
O95571	0.057637215	1.015581977	ETHE1 HSCO
Q16540	0.057872772	0.746375057	MRPL23 L23MRP RPL23L
Q0VDG4	0.057958603	0.266094756	SCRN3
Q8NCN5	0.058051109	0.283151594	PDPR KIAA1990
Q9H4I3	0.058066368	0.990838303	TRABD TTG2 PP2447
Q9H9Q2	0.058069229	0.294615716	COPS7B CSN7B
Q9NX24	0.058624268	0.525662062	NHP2 NOLA2 HSPC286
Q8WUM4	0.058685303	0.845155713	PDCD6IP AIP1 ALIX KIAA1375
P14854	0.058913231	0.946890367	COX6B1 COX6B
O96005	0.058917999	1.020198849	CLPTM1
Q96DE0	0.059020996	0.592466063	NUDT16
Q8N9N7	0.059091568	0.788411525	LRRC57
Q5JRX3	0.059163094	0.779209896	PITRM1 KIAA1104 MP1 PREP
Q8WU90	0.059175491	0.944042067	ZC3H15 DFRP1 LEREPO4 HSPC303 HT010 MSTP012 PP730
Q4G0F5	0.059205055	1.486412748	VPS26B
O43617	0.05922699	0.751379767	TRAPPC3 BET3 CDABP0066
P19367	0.059234619	2.238689435	HK1
Q969G5	0.059238434	1.138421139	CAVIN3 PRKCDBP SRBC
Q9H832	0.05946064	0.230510294	UBE2Z HOYS7
Q9NP97	0.059499741	0.474679777	DYNLRB1 BITH DNCL2A DNLC2A ROBLD1 HSPC162
Q8TEA7	0.05952549	0.299520812	TBCK TBCKL HSPC302

Q8WTT2	0.059643745	0.798877445	NOC3L AD24 C10orf117 FAD24
Q9P2X3	0.059720993	0.184992519	IMPACT
P09104	0.059762955	0.846858605	ENO2
Q9NWZ5	0.059781075	0.442079134	UCKL1 URKL1 F538
Q99613	0.05982399	1.147902893	EIF3C EIF3S8
Q9P015	0.059850693	0.659563923	MRPL15 HSPC145
Q9Y5S2	0.059867859	3.414315664	CDC42BPB KIAA1124
Q9BWJ5	0.060270309	0.469700432	SF3B5 SF3B10
Q13546	0.06027317	1.209866246	RIPK1 RIP RIP1
O15042	0.060333252	1.116659762	U2SURP KIAA0332 SR140
O75431	0.060400963	0.172248973	MTX2
O96011	0.060601234	0.457584243	PEX11B
Q16134	0.060900688	0.554504734	ETFDH
O75113	0.060955524	0.529295554	N4BP1 KIAA0615
Q66K74	0.061139107	0.800742238	MAP1S BPY2IP1 C19orf5 MAP8 VCY2IP1
P22830	0.061349869	1.438040306	FECH
Q6KCM7	0.061357498	0.27568262	SLC25A25 APC3 KIAA1896 MCSC3 SCAMC2 UNQ549/PRO1106
Q7Z333	0.061371803	0.141833633	SETX ALS4 KIAA0625 SCAR1
P51970	0.061509132	0.425487099	NDUFA8
Q8NCW5	0.061543465	0.500612505	NAXE AIBP APOA1BP YJEFN1
P20645	0.061605453	0.700051066	M6PR MPR46 MPRD
Q9UNE7	0.061667442	0.528295307	STUB1 CHIP PP1131
Q9H3P2	0.061713219	0.333973964	NELFA WHSC2 P/OKcl.15
Q9H0U3	0.061717987	0.675739133	MAGT1 IAG2 PSEC0084 UNQ628/PRO1244
O94923	0.06183815	0.535032043	GLCE KIAA0836
Q96RS6	0.061924934	0.839371297	NUDCD1 CML66
Q9BS26	0.061959267	1.325655882	ERP44 KIAA0573 TXNDC4 UNQ532/PRO1075
Q9P0I2	0.062104702	0.254976785	EMC3 TMEM111
P28482	0.062145233	0.649493639	MAPK1 ERK2 PRKM1 PRKM2
Q2NL82	0.062292099	0.947604554	TSR1 KIAA1401
Q96FV2	0.062302589	0.248692929	SCRN2
Q8IY33	0.062355042	0.644151156	MICALL2 JRAB
O60716	0.06248188	2.065467869	CTNND1 KIAA0384
P82673	0.062550545	0.456889596	MRPS35 MRPS28 HDCMD11P MDS023 PSEC0213
Q13671	0.062614441	0.288510812	RIN1
Q9BQD3	0.062673569	0.954496973	KXD1 C19orf50
A6NJ78	0.062884331	0.210829315	METTL15 METT5D1
P10606	0.063035965	0.892952017	COX5B
P07237	0.063087463	0.41824247	P4HB ERBA2L PDI PDIA1 PO4DB
Q9UK41	0.063100815	0.421090377	VPS28
P05166	0.06327343	0.288906338	PCCB

Q7Z4W1	0.063286781	0.293540891	DCXR SDR20C1
Q9UDR5	0.063288689	0.576222527	AASS
Q8WZ42	0.063303947	0.386514656	TTN
P59998	0.063387871	0.321815453	ARPC4 ARC20
O75306	0.063398361	0.892026873	NDUFS2
Q6XZF7	0.063430309	0.2231422	DNMBP ARHGEF36 KIAA1010 TUBA
O75448	0.063476563	0.601802124	MED24 ARC100 CRSP4 DRIP100 KIAA0130 THRAP4 TRAP100
Q8TF42	0.063490868	1.191975222	UBASH3B KIAA1959 STS1
Q6Y7W6	0.063523293	1.935736982	GIGYF2 KIAA0642 PERQ2 TNRC15
O75808	0.063614368	0.805909507	CAPN15 SOLH
Q15018	0.063679695	1.877275516	ABRAXAS2 ABRO1 FAM175B KIAA0157
P98160	0.063689232	1.080828055	HSPG2
O15382	0.063734055	0.792226415	BCAT2 BCATM BCT2 ECA40
O95182	0.063911438	0.634396332	NDUFA7
Q9NTX5	0.063990593	0.266237355	ECHDC1
Q9GZR7	0.064045906	0.320310636	DDX24
Q9UKA4	0.064334393	0.468616095	AKAP11 AKAP220 KIAA0629
P43378	0.06438446	0.381873824	PTPN9
Q8WX92	0.064739227	0.293040797	NELFB COBRA1 KIAA1182
O95260	0.064748764	0.26989798	ATE1
Q96J02	0.064922333	0.139335325	ITCH
P06865	0.065052032	1.589129046	HEXA
Q9H1Z4	0.065132141	0.457066973	WDR13
Q9NQT4	0.065278053	0.843905297	EXOSC5 CML28 RRP46
Q9H267	0.065295219	0.581416478	VPS33B
P10746	0.065342903	2.078007041	UROS
Q9UPU9	0.065414429	0.194563486	SAMD4A KIAA1053 SAMD4 SMAUG1
P09960	0.065488815	1.0872397	LTA4H LTA4
Q9HD42	0.065505028	0.448362877	CHMP1A CHMP1 KIAA0047 PCOLN3 PRSM1
P15153	0.065670013	1.338035272	RAC2
P61088	0.065735817	0.614194054	UBE2N BLU
Q92615	0.065962791	0.747981753	LARP4B KIAA0217 LARP5
Q9H488	0.066007614	0.618771035	POFUT1 FUT12 KIAA0180
Q4G0X4	0.066046715	0.832532111	KCTD21
Q8N3R9	0.066102982	0.847392863	PALS1 MPP5
Q6UXH1	0.066196442	2.037387874	CRELD2 UNQ185/PRO211
Q8IWF6	0.066244125	0.13789683	DENND6A FAM116A
P39880	0.066390038	0.545027754	CUX1 CUTL1
Q8IYS2	0.066437721	1.538348252	KIAA2013
P00519	0.066589355	0.65132646	ABL1 ABL JTK7
Q6DKI1	0.066734314	0.890122734	RPL7L1
Q9UHQ9	0.066810608	0.751356718	CYB5R1 NQO3A2 UNQ3049/PRO9865

Q96LW7	0.066917419	0.572246489	CARD19 C9orf89
Q969V6	0.066935539	0.667208883	MRTFA KIAA1438 MAL MKL1
Q9UFC0	0.066943169	0.378888023	LRWD1 CENP-33 ORCA
Q8N4A0	0.066951752	0.218809077	GALNT4
Q9NQZ2	0.067027092	0.605963071	UTP3 CRLZ1 SAS10
Q53FA7	0.067063332	0.742337918	TP53I3 PIG3
Q96KC8	0.067118645	0.517539315	DNAJC1 HTJ1
Q9BZL1	0.067251205	0.914405757	UBL5
P35573	0.067369461	1.420812101	AGL GDE
Q29RF7	0.067418098	0.911826517	PDS5A KIAA0648 PDS5 PIG54
Q05209	0.067440987	0.992776771	PTPN12
Q9Y2C4	0.067453384	0.966100028	EXOG ENDOGL1 ENDOGL2 ENGL
Q9Y4P3	0.067499161	1.171288503	TBL2 WBSCR13 UNQ563/PRO1125
O75607	0.067658424	0.263488027	NPM3
Q9BPX6	0.067703247	0.505020571	MICU1 CALC CBARA1
Q8TE77	0.067708015	1.704200141	SSH3 SSH3L
Q6NVY1	0.067708015	0.700497585	HIBCH
Q96A33	0.067954063	1.29607558	CCDC47 GK001 MSTP041 PSEC0077
Q96HE7	0.067985535	0.89662511	ERO1A ERO1L UNQ434/PRO865
Q5SRE5	0.068318367	0.524081673	NUP188 KIAA0169
P29401	0.06832695	0.481944635	TKT
O14828	0.068450928	0.251516565	SCAMP3 C1orf3 PROPIN1
Q9P265	0.068476677	0.683405617	DIP2B KIAA1463
O60502	0.068561554	1.206182554	OGA HEXC KIAA0679 MEA5 MGEA5
Q53H82	0.068570137	0.556485369	LACTB2 CGI-83
P27487	0.06874752	0.448648817	DPP4 ADCP2 CD26
Q00577	0.068768501	0.332671111	PURA PUR1
Q9C0E2	0.069042206	0.950714811	XPO4 KIAA1721
P50990	0.069074631	0.55552282	CCT8 C21orf112 CCTQ KIAA0002
Q9UBP4	0.069597244	0.468667374	DKK3 REIC UNQ258/PRO295
P46734	0.069645882	0.616484543	MAP2K3 MEK3 MKK3 PRKMK3 SKK2
O94813	0.069658279	0.753008355	SLIT2 SLIL3
Q2TAA2	0.069694519	1.798340835	IAH1
O00400	0.069918633	1.225328593	SLC33A1 ACATN AT1
O75569	0.069937706	2.470020576	PRKRA PACT RAX HSD-14 HSD14
Q9UBQ0	0.069970131	0.295566175	VPS29 DC15 DC7 MDS007
Q96IU4	0.070152283	0.454520406	ABHD14B CIB
P56377	0.070292473	1.071890427	AP1S2 DC22
Q6NUQ1	0.070356369	0.322068706	RINT1
Q9Y4W6	0.070412636	0.239531277	AFG3L2
O14656	0.070490837	0.589451636	TOR1A DQ2 DYT1 TA TORA
Q86UP2	0.070721626	0.87272325	KTN1 CG1 KIAA0004
P52306	0.07075119	0.944277529	RAP1GDS1

Q3MHD2	0.070836067	1.068730126	LSM12
Q9BQQ3	0.070953369	0.742813226	GORASP1 GOLPH5 GRASP65
Q9Y4B5	0.070991516	0.296863143	MTCL1 CCDC165 KIAA0802 SOGA2
O94885	0.071043968	0.425753863	SASH1 KIAA0790 PEPE1
Q9P2E3	0.071137428	1.139361876	ZNFX1 KIAA1404
Q9BRX2	0.071347237	0.936313668	PELO CGI-17
Q8N129	0.07145977	0.205374449	CNPY4 PSEC0237 UNQ1909/PRO4354
Q96I25	0.071489334	1.008308812	RBM17 SPF45
Q96C19	0.071643829	2.366855826	EFHD2 SWS1
A4D1P6	0.071675301	1.148746182	WDR91 HSPC049
O75688	0.071735382	0.797316513	PPM1B PP2CB
Q6NUK1	0.071915627	0.384709865	SLC25A24 APC1 MCSC1 SCAMC1
P12236	0.071979523	1.523714685	SLC25A6 AAC3 ANT3 CDABP0051
Q9BYM8	0.072092056	0.620252512	RBCK1 C20orf18 RNF54 UBCE7IP3 XAP3 XAP4
Q9BV86	0.072106361	0.885460497	NTMT1 C9orf32 METTL11A NRMT NRMT1 AD-003
Q86YZ3	0.07216692	0.471703121	HRNR S100A18
Q9UK61	0.072322845	0.488540178	TASOR C3orf63 FAM208A KIAA1105
P43155	0.07232666	0.694178467	CRAT CAT1
Q13242	0.072773933	2.677730065	SRSF9 SFRS9 SRP30C
Q7KZ85	0.072943687	0.881770948	SUPT6H KIAA0162 SPT6H
P62699	0.073143959	0.598448385	YPEL5 CGI-127
Q14241	0.073215485	0.459200524	ELOA TCEB3 MSTP059
Q6PI48	0.073468208	0.212989891	DARS2
Q9H7D0	0.073484421	0.676591031	DOCK5
Q96BY6	0.073524475	1.559553743	DOCK10 KIAA0694 ZIZ3
Q9NQX7	0.073735237	0.432792166	ITM2C BRI3 nucep-14 NPD018 PSEC0047
O43164	0.073762894	0.459265096	PJA2 KIAA0438 RNF131
Q14145	0.073900223	0.444207795	KEAP1 INRF2 KIAA0132 KLHL19
Q9H299	0.073939323	1.010993827	SH3BGRL3 P1725
Q00688	0.074141502	0.604295126	FKBP3 FKBP25
Q9NTJ4	0.074195862	0.341698038	MAN2C1 MANA MANA1
O95831	0.074244499	2.857887582	AIFM1 AIF PDCD8
Q9UHB9	0.074359894	1.227514759	SRP68
Q01518	0.074547768	0.43465914	CAP1 CAP
P54760	0.074614525	0.433418043	EPHB4 HTK MYK1 TYRO11
Q9UI09	0.07461834	1.346491095	NDUFA12 DAP13
Q86UE4	0.07469368	1.029356455	MTDH AEG1 LYRIC
Q92466	0.0747118	1.576608135	DDB2
Q9H6Y2	0.074766159	0.935369909	WDR55
P29144	0.074783325	2.770754634	TPP2
O95183	0.074808121	0.651131159	VAMP5 HSPC191
Q9P0J0	0.074864388	0.652860639	NDUFA13 GRIM19 CDA016 CGI-39

Q6L8Q7	0.075059891	0.958101553	PDE12
Q9NP61	0.075193405	1.327356895	ARFGAP3 ARFGAP1
Q9BXW7	0.075198174	0.392466776	HDHD5 CECR5
Q6SZW1	0.075309753	0.215753318	SARM1 KIAA0524 SAMD2 SARM
Q8N183	0.075339317	0.648317035	NDUFAF2 NDUFA12L
O43681	0.075340271	0.710381372	GET3 ARSA ASNA1 TRC40
Q5VW36	0.075548172	0.547554891	FOCAD KIAA1797
O43678	0.075636864	1.307818665	NDUFA2
Q9BQS8	0.075695992	0.655925938	FYCO1 ZFYVE7
P51398	0.075709343	0.636341467	DAP3 MRPS29
P52735	0.0757761	0.571809416	VAV2
Q9BRT3	0.075938225	1.785246201	MIEN1 C17orf37 RDX12 XTP4
P55789	0.075965881	0.4309942	GFER ALR HERV1 HPO
Q969Q5	0.075969696	1.302795799	RAB24
Q8TCJ2	0.076174736	1.739501855	STT3B SIMP
Q13630	0.076257706	0.871228967	GFUS SDR4E1 TSTA3
Q96JB2	0.076571465	0.569395829	COG3 SEC34
Q9NX40	0.076678276	1.02148486	OCIAD1 ASRIJ OCIA
O43324	0.076878548	0.603791893	EEF1E1 AIMP3 P18
Q9H6R4	0.076910973	0.801874008	NOL6
Q9P2K8	0.076913834	2.07861154	EIF2AK4 GCN2 KIAA1338
P06737	0.077036858	0.578128996	PYGL
P08123	0.077254295	0.903451063	COL1A2
Q8IWA5	0.077302933	0.981695075	SLC44A2 CTL2 PSEC0210
P54619	0.077315331	0.529274346	PRKAG1
Q14376	0.077802658	1.258313917	GALE
P17987	0.077876091	0.872284843	TCP1 CCT1 CCTA
P49815	0.077896118	0.77631067	TSC2 TSC4
P30101	0.077978134	0.949399939	PDIA3 ERP57 ERP60 GRP58
O95487	0.078038216	1.421180201	SEC24B
Q9NZZ3	0.078087807	1.966253993	CHMP5 C9orf83 SNF7DC2 CGI-34 HSPC177 PNAS-114 PNAS-2
Q7Z478	0.078103065	1.687545256	DHX29 DDX29
P46531	0.078216553	0.371743367	NOTCH1 TAN1
P35658	0.078398705	1.285112298	NUP214 CAIN CAN KIAA0023
Q9BWS9	0.078727722	0.762419359	CHID1 GL008 PSEC0104 SB139
Q96T76	0.078727722	0.849527004	MMS19 MMS19L
Q8IVL6	0.078853607	0.617121788	P3H3 LEPREL2
Q9Y316	0.078900337	0.952121314	MEMO1 C2orf4 MEMO NS5ATP7 CGI-27
O75347	0.078904152	0.306568037	TBCA
Q9UKK9	0.078965187	0.596489393	NUDT5 NUDIX5 HSPC115
Q9BWH6	0.079129696	0.213324647	RPAP1 KIAA1403
Q53GS9	0.079166412	0.150325942	USP39 CGI-21 HSPC332 PRO2855
Q8N7H5	0.079292297	0.81687961	PAF1 PD2

P78332	0.079362869	1.096166346	RBM6 DEF3
Q14BN4	0.079392433	0.354407381	SLMAP KIAA1601 SLAP UNQ1847/PRO3577
Q147X3	0.079508781	1.140829555	NAA30 C14orf35 MAK3 NAT12
O00459	0.079562187	1.593172245	PIK3R2
Q53EL6	0.079566002	1.792692002	PDCD4 H731
Q9UBQ7	0.079634666	2.150444486	GRHPR GLXR MSTP035
Q9Y3E5	0.079741478	0.755496742	PTRH2 BIT1 PTH2 CGI-147
Q15750	0.079877853	0.531716564	TAB1 MAP3K7IP1
O15381	0.079885483	0.952159836	NVL NVL2
Q15257	0.079888344	0.779171228	PTPA PPP2R4
Q9Y6W5	0.07999897	0.324630353	WASF2 WAVE2
O95167	0.08003521	0.311061157	NDUFA3
Q86VR2	0.080105782	0.503114949	RETREG3 FAM134C
Q14157	0.080140114	1.840652286	UBAP2L KIAA0144 NICE4
Q9C0C9	0.080174446	0.653355802	UBE2O KIAA1734
O15027	0.080190659	1.113180624	SEC16A KIAA0310 SEC16 SEC16L
Q13232	0.080393791	1.615952524	NME3
Q9BYJ9	0.080415726	0.804575258	YTHDF1 C20orf21
Q5JRA6	0.080533981	1.854100386	MIA3 KIAA0268 TANGO UNQ6077/PRO20088
Q6PCE3	0.080559731	1.132531272	PGM2L1 BM32A
O95628	0.080652237	0.543027399	CNOT4 NOT4
P04424	0.08079052	1.587359387	ASL
P02794	0.08082962	1.088858261	FTH1 FTH FTHL6 OK/SW-cl.84 PIG15
P17900	0.080908298	0.55419154	GM2A
Q6XQN6	0.080989838	0.717093828	NAPRT FHIP NAPRT1
Q9P2N5	0.081012726	0.225178938	RBM27 KIAA1311
O60524	0.08102417	0.961088244	NEMF SDCCAG1
Q5VU43	0.081036568	0.412352269	PDE4DIP CMYA2 KIAA0454 KIAA0477 MMGL
O60568	0.081063271	0.989114011	PLOD3
Q8N7R7	0.081100464	0.345577781	CCNYL1
Q96QU8	0.081249237	0.19262531	XPO6 KIAA0370 RANBP20
A3KMH1	0.081336975	2.139374781	VWA8 KIAA0564
P55196	0.081410408	0.987483928	AFDN AF6 MLLT4
Q13454	0.081586838	1.342164816	TUSC3 N33
Q96EY7	0.081686974	1.857795772	PTCD3 MRPS39 TRG15
O43747	0.082105637	1.158029906	AP1G1 ADTG CLAPG1
Q9Y2R5	0.082109451	0.781001548	MRPS17 RPMS17 HSPC011
Q9Y3D6	0.082325935	1.244055148	FIS1 TTC11 CGI-135
P08962	0.082336426	0.645131969	CD63 MLA1 TSPAN30
P32321	0.082341194	3.379407069	DCTD
Q13393	0.082382679	0.498965269	PLD1
P00395	0.082384109	0.410403704	MT-CO1 COI COXI MTCO1

Q9UPN3	0.082393646	1.65815091	MACF1 ABP620 ACF7 KIAA0465 KIAA1251
Q8TD16	0.082612991	0.927220829	BICD2 KIAA0699
Q9BXF6	0.082652092	1.259737504	RAB11FIP5 GAF1 KIAA0857 RIP11
Q8N3D4	0.082672596	0.862241462	EHBP1L1
P20674	0.082763672	1.182982925	COX5A
O94973	0.082838058	0.833309966	AP2A2 ADTAB CLAPA2 HIP9 HYPJ KIAA0899
Q9BSJ2	0.082876205	0.638207397	TUBGCP2 GCP2
Q96GQ7	0.082969666	0.378304329	DDX27 cPERP-F RHLP HSPC259 PP3241
Q8IYD1	0.082998276	0.920533769	GSPT2 ERF3B
Q8NBU5	0.083155632	0.549111193	ATAD1 FNP001
Q99594	0.08324337	0.308159278	TEAD3 TEAD5 TEF5
O60826	0.083258629	1.648179017	CCDC22 CXorf37 JM1
Q68CP9	0.083275795	0.538578026	ARID2 BAF200 KIAA1557
Q9UBX5	0.083400726	0.55734293	FBLN5 DANCE UNQ184/PRO210
Q92692	0.083456039	0.746074802	NECTIN2 HVEB PRR2 PVRL2
Q12907	0.083745956	2.5348403	LMAN2 C5orf8
Q9UBP0	0.083755493	0.269798939	SPAST ADPSP FSP2 KIAA1083 SPG4
Q96AB3	0.083785057	0.66635683	ISOC2
O95139	0.083820343	1.123940909	NDUFB6
Q03701	0.084026337	0.36139908	CEBPZ CBF2
Q9Y2Z4	0.084039688	0.369407513	YARS2 CGI-04
Q86Y07	0.084080696	1.6609901	VRK2
Q7L576	0.08412075	1.060431412	CYFIP1 KIAA0068
Q00341	0.084269524	0.808399325	HDLBP HBP VGL
Q6JQN1	0.084423542	0.17252996	ACAD10
Q13356	0.084620953	0.670631719	PPIL2
P55010	0.084644318	0.518848879	EIF5
Q16186	0.084659576	2.208519923	ADRM1 GP110
Q9UJ83	0.084686279	1.29710641	HACL1 HPCL HPCL2 PHYH2 HSPC279
Q14393	0.084710121	1.095206938	GAS6 AXLLG
P98172	0.084742546	1.013522522	EFNB1 EFL3 EPLG2 LERK2
Q15369	0.0849123	0.487804428	ELOC TCEB1
Q96JJ3	0.084962845	1.300257997	ELMO2 CED12A KIAA1834
Q14344	0.085061073	0.692286787	GNA13
Q9UIQ6	0.085095406	0.717845218	LNPEP OTASE
Q96GS4	0.085326672	1.671253647	BORCS6 C17orf59
Q8TCS8	0.085483551	1.037731573	PNPT1 PNPASE
P13987	0.086470604	0.815396087	CD59 MIC11 MIN1 MIN2 MIN3 MSK21
Q5JTZ9	0.086495399	1.30274693	AARS2 AARSL KIAA1270
Q03518	0.086540222	1.047458737	TAP1 ABCB2 PSF1 RING4 Y3
Q9UJU6	0.086590767	1.878347751	DBNL CMAP SH3P7 PP5423
Q13153	0.086680412	0.481623654	PAK1
Q15311	0.086709976	1.827499904	RALBP1 RLIP RLIP1 RLIP76

Q712K3	0.086801529	1.458775751	UBE2R2 CDC34B UBC3B
P47985	0.086807251	0.394301598	UQCRFS1
Q9UEW8	0.086811066	1.56905482	STK39 SPAK
P04080	0.086948395	0.486359089	CSTB CST6 STFB
P02788	0.087082863	0.867681807	LTF GIG12 LF
Q96L91	0.087111473	0.538867335	EP400 CAGH32 KIAA1498 KIAA1818 TNRC12
Q9NRY6	0.087139606	0.37396519	PLSCR3
Q5BJH7	0.087287903	0.484187875	YIF1B PP4519 UNQ3073/PRO9905
Q9BT09	0.087450027	1.715654796	CNPY3 CTG4A ERDA5 PRAT4A TNRC5 HSPC084 UNQ1934/PRO4409
Q969E2	0.087471008	0.513463556	SCAMP4
Q9H3S7	0.087585449	1.354353198	PTPN23 KIAA1471
P09038	0.087835312	0.509162337	FGF2 FGFB
Q9BYD1	0.087844849	2.479806664	MRPL13
Q9BVC6	0.088090897	1.299210576	TMEM109
Q4G176	0.088137627	0.523010478	ACSF3 PSEC0197
O75251	0.088573456	1.167419904	NDUFS7
P14210	0.088681221	1.738666924	HGF HPTA
Q9BU23	0.088687897	1.991239873	LMF2 TMEM112B TMEM153
Q96BX8	0.088871002	1.001639509	MOB3A MOBKL2A
Q8IXM3	0.088973045	0.989854491	MRPL41 BMRP MRPL27 RPML27 PIG3
Q9NP92	0.089010239	0.722435805	MRPS30 PDCD9 BM-047
P08253	0.089093208	0.808390406	MMP2 CLG4A
P51178	0.08938694	1.58191448	PLCD1
Q9P2G1	0.089524269	1.992287783	ANKIB1 KIAA1386
P19022	0.089756012	1.582820934	CDH2 CDHN NCAD
Q5H8A4	0.089769363	0.435338852	PIGG GPI7 UNQ1930/PRO4405
O95816	0.089856148	0.723710391	BAG2
Q92766	0.090119362	1.305808954	RREB1 FINB
O14646	0.090317726	0.435379247	CHD1
Q96RS0	0.090456009	0.254216836	TGS1 HCA137 NCOA6IP PIMT
Q9NYU1	0.090465546	0.67028307	UGGT2 UGCGL2 UGT2
Q9Y5X1	0.090504646	2.848551867	SNX9 SH3PX1 SH3PXD3A
Q8NC56	0.090667725	1.225081358	LEMD2
Q96A65	0.09069252	1.855656862	EXOC4 KIAA1699 SEC8 SEC8L1
O94979	0.090847969	0.810691516	SEC31A KIAA0905 SEC31L1 HSPC275 HSPC334
P07942	0.090898514	0.928990674	LAMB1
Q5RI15	0.091083527	0.551674751	COX20 FAM36A
P30044	0.091223717	0.7241419	PRDX5 ACR1 SBBI10
O15155	0.091373444	1.057869707	BET1
P57737	0.09141922	0.374374453	CORO7
P16930	0.091927528	1.748004129	FAH

Q9UKK3	0.091962814	1.614902223	PARP4 ADPRTL1 KIAA0177 PARPL
Q96GP6	0.091980934	0.653132264	SCARF2 SREC2 SREPCR
P62314	0.092049599	0.727581947	SNRPD1
Q8TD55	0.092104912	1.460808557	PLEKHO2 PLEKHQ1 PP9099
P62191	0.092215538	2.196482666	PSMC1
O76054	0.092340469	1.929802348	SEC14L2 C22orf6 KIAA1186 KIAA1658
Q9Y2J2	0.092394829	0.804324325	EPB41L3 DAL1 KIAA0987
O95429	0.092744827	0.817813646	BAG4 SODD
Q9NYL4	0.09277916	2.133286384	FKBP11 FKBP19 UNQ336/PRO535
Q6NUQ4	0.092787743	1.220672249	TMEM214 PP446
P61966	0.092812538	0.766622343	AP1S1 AP19 CLAPS1
Q15031	0.092881203	0.453600483	LARS2 KIAA0028
Q8NDT2	0.093152046	0.568727785	RBM15B OTT3
Q16543	0.093158722	1.471412288	CDC37 CDC37A
Q13488	0.093363762	0.758860608	TCIRG1 ATP6N1C ATP6V0A3
Q9Y3C4	0.093655586	0.705459589	TPRKB CGI-121 My019
Q9UL15	0.09386158	0.847510685	BAG5 KIAA0873
Q9BPX5	0.094025612	0.53863162	ARPC5L
Q96GA7	0.094191551	1.170502257	SDSL
Q99519	0.094286919	1.346047865	NEU1 NANH
P60981	0.094307899	1.035467849	DSTN ACTDP DSN
O60613	0.09431839	1.105242609	SELENOF SEP15
Q4J6C6	0.094378471	1.584542359	PREPL KIAA0436
Q86WA6	0.094437599	0.507505391	BPHL MCNAA
P29590	0.094605446	1.610987441	PML MYL PP8675 RNF71 TRIM19
Q9NZJ4	0.094711304	1.760396872	SACS KIAA0730
Q86SX6	0.094781876	0.890454946	GLRX5 C14orf87
Q86V48	0.09516716	2.71551772	LUZP1
Q68CZ2	0.095212936	2.158846882	TNS3 TEM6 TENS1 TPP
Q7Z7H5	0.095239639	1.063905532	TMED4 ERS25
Q9BU61	0.095337391	0.11947782	NDUFAF3 C3orf60
O60645	0.095351219	0.626277172	EXOC3 SEC6 SEC6L1
P23258	0.095516682	0.462284436	TUBG1 TUBG
P20340	0.095678329	1.907439073	RAB6A RAB6
Q8IWV8	0.095722198	1.130597371	UBR2 C6orf133 KIAA0349
Q9UGJ1	0.095752716	0.439115703	TUBGCP4 76P GCP4
Q9H2G2	0.095754623	1.486281392	SLK KIAA0204 STK2
Q9P000	0.095786095	1.662321117	COMMD9 HSPC166
Q9H0E2	0.095872879	1.256096699	TOLLIP
Q5T160	0.09606266	1.518750375	RARS2 RARSL
Q9Y5A9	0.09625721	0.944027519	YTHDF2 HGRG8
Q9UBF8	0.096344948	1.685097641	PI4KB PIK4CB
O14530	0.096403122	1.939188986	TXNDC9 APACD

Q9BWF3	0.096549988	0.912151286	RBM4 RBM4A
Q9NY61	0.096741676	0.371809942	AATF CHE1 DED HSPC277
Q9UNH7	0.096747398	1.591876556	SNX6
Q9NQS1	0.096992493	0.48485166	AVEN
Q8IY95	0.097255707	0.581806809	TMEM192
P47755	0.097378731	0.937828399	CAPZA2
P12429	0.09748745	1.241070305	ANXA3 ANX3
Q96P48	0.097670555	1.184898939	ARAP1 CENTD2 KIAA0782
Q96RU3	0.097750664	1.702558999	FNBP1 FBP17 KIAA0554
Q99650	0.097937584	1.151436331	OSMR OSMRB
O15173	0.098152161	1.526304462	PGRMC2 DG6 PMBP
P49189	0.098546982	1.224549857	ALDH9A1 ALDH4 ALDH7 ALDH9
Q9H7D7	0.098667145	1.828075624	WDR26 CDW2 MIP2 PRO0852
Q13618	0.098812103	2.68733197	CUL3 KIAA0617
Q08426	0.098831177	0.649059998	EHHADH ECHD
Q7L5N7	0.099373817	1.436913204	LPCAT2 AGPAT11 AYTL1
P50552	0.099516869	0.786252372	VASP
P62495	0.099635124	2.153534506	ETF1 ERF1 RF1 SUP45L1
Q9Y312	0.09978199	1.051280424	AAR2 C20orf4 CGI-23 PRO0225
O00487	0.099906921	1.370133914	PSMD14 POH1
O43795	0.099917412	1.004957463	MYO1B
Q9BUI4	0.100013256	0.887597973	POLR3C
Q14112	0.100028992	1.627267346	NID2
A2RRP1	0.100108147	0.995307612	NBAS NAG
Q14204	0.100221634	1.09116522	DYNC1H1 DHC1 DNCH1 DNCL DNECL DYHC KIAA0325
Q9UI10	0.100248337	1.749557406	EIF2B4 EIF2BD
Q86W50	0.100252151	1.918529526	METTL16 METT10D
Q5T9L3	0.100377083	0.628271909	WLS C1orf139 GPR177 UNQ85/PRO18667
Q9UNH6	0.100584984	0.558435017	SNX7
O95486	0.100601196	1.233225648	SEC24A
O15400	0.100608826	0.501869392	STX7
Q9UP83	0.100794792	1.340257453	COG5 GOLTC1 GTC90
Q15070	0.100858688	0.865703374	OXA1L
Q9Y385	0.100948334	0.647329827	UBE2J1 NCUBE1 CGI-76 HSPC153 HSPC205
Q13315	0.10106802	0.327203484	ATM
Q9C0B5	0.101079941	1.570885283	ZDHHC5 KIAA1748 ZNF375
Q14160	0.101086617	0.88719756	SCRIB CRIB1 KIAA0147 LAP4 SCRB1 VARTUL
Q92545	0.101175308	1.171131258	TMEM131 KIAA0257 RW1
Q8IVH8	0.101215363	0.753602623	MAP4K3 RAB8IPL1
Q7Z4G1	0.101413727	1.17775169	COMMD6 MSTP076
Q7Z4H3	0.101415634	1.435157191	HDDC2 C6orf74 NS5ATP2 CGI-130

P63172	0.101448059	1.016389515	DYNLT1 TCTEL1 TCTEX-1 TCTEX1
Q14690	0.10174942	2.052569907	PDCD11 KIAA0185
O60763	0.101806641	0.917050688	USO1 VDP
Q9NP84	0.10187006	0.79789638	TNFRSF12A FN14
P30837	0.101892471	1.882527901	ALDH1B1 ALDH5 ALDHX
Q9Y6K9	0.102063179	0.807867308	IKBKG FIP3 NEMO
P02786	0.10228157	2.112654342	TFRC
P08572	0.102413177	0.899376568	COL4A2
P55268	0.102537155	1.774810196	LAMB2 LAMS
Q9P253	0.102540016	1.427471709	VPS18 KIAA1475
Q13505	0.102615356	1.737426435	MTX1 MTX MTXN
O75381	0.102645874	0.418546688	PEX14
Q9P0J7	0.102748871	1.095646219	KCMF1 FIGC ZZZ1
Q13158	0.102842331	0.808895913	FADD MORT1 GIG3
P30622	0.102965355	0.989953348	CLIP1 CYLN1 RSN
P26572	0.103134155	0.786491103	MGAT1 GGNT1 GLCT1 GLYT1 MGAT
Q9BX10	0.103452682	0.51359329	GTPBP2
Q99653	0.103462219	1.587913898	CHP1 CHP
P49441	0.103634834	1.216509391	INPP1
Q16610	0.103653908	1.336559895	ECM1
Q6WCQ1	0.103696823	1.485093992	MPRIP KIAA0864 MRIP RHOIP3
Q9UPY6	0.10381794	1.368652087	WASF3 KIAA0900 SCAR3 WAVE3
O95985	0.103981972	0.984576627	TOP3B TOP3B1
P42226	0.104055405	2.270036043	STAT6
Q9Y5P4	0.104184151	1.463498157	CERT1 CERT COL4A3BP STARD11
P20585	0.104623795	0.131966349	MSH3 DUC1 DUG
O75600	0.104743958	1.102067319	GCAT KBL
Q9BQ67	0.104767799	0.627108642	GRWD1 GRWD KIAA1942 WDR28
Q15629	0.104888916	1.650811166	TRAM1 TRAM
Q86U70	0.104896545	1.205684105	LDB1 CLIM2
O43148	0.105158806	1.630892007	RNMT KIAA0398
Q9NXH8	0.105208397	0.819430856	TOR4A C9orf167
Q8WUX9	0.105400085	0.629186991	CHMP7
P84095	0.10548687	1.109466482	RHOG ARHG
O95373	0.105537415	1.236039433	IPO7 RANBP7
P61020	0.105560303	0.990921337	RAB5B
Q5T3I0	0.105834961	0.259478076	GPATCH4 GPATC4
Q9Y2K7	0.106079102	0.901650816	KDM2A CXXC8 FBL11 FBL7 FBXL11 JHDM1A KIAA1004
Q9H7B2	0.106204987	1.865600603	RPF2 BXDC1
Q9BYD3	0.106208801	1.704074594	MRPL4 CDABP0091 CGI-28
P50583	0.106268883	1.58082046	NUDT2 APAH1
P20933	0.106302261	1.897218321	AGA
O75521	0.106514931	2.69649928	ECI2 DRS1 HCA88 PECI

O95169	0.106577873	0.981905229	NDUFB8
Q92900	0.106636047	2.035950792	UPF1 KIAA0221 RENT1
Q96FN4	0.106637001	0.755334785	CPNE2
Q9HCM4	0.106852531	1.561491519	EPB41L5 KIAA1548
P63218	0.106890678	0.810590688	GNG5 GNGT5
P55212	0.107226372	0.952606376	CASP6 MCH2
Q9UBF2	0.107456207	2.170391668	COPG2
Q01995	0.107484818	1.69032698	TAGLN SM22 WS3-10
Q86YV9	0.107736588	1.845720431	HPS6
Q8IVB5	0.10819149	0.776325868	LIX1L
Q00978	0.108222961	0.952939841	IRF9 ISGF3G
O95202	0.10822773	1.616075069	LETM1
Q9UBV2	0.108240128	1.048236021	SEL1L TSA305 UNQ128/PRO1063
O60547	0.10825634	1.100243025	GMDS
O15417	0.108380795	0.901566867	TNRC18 CAGL79 KIAA1856
P46934	0.10839653	0.928708173	NEDD4 KIAA0093 NEDD4-1 RIG53
Q9UFN0	0.108602524	2.169186286	NIPSNAP3A NIPSNAP4 HSPC299
P45877	0.108675003	0.834747638	PPIC CYPC
Q8IXJ6	0.10892868	0.779639125	SIRT2 SIR2L SIR2L2
Q5VSL9	0.108981133	0.692987636	STRIP1 FAM40A KIAA1761
Q9GZT3	0.109013557	0.581933237	SLIRP C14orf156 DC23 DC50 PD04872
O14548	0.109116554	0.406550034	COX7A2L COX7AR COX7RP
P21283	0.10915184	2.042371976	ATP6V1C1 ATP6C ATP6D VATC
P38435	0.109434128	0.710569875	GGCX GC
Q9NPH3	0.109573364	0.552189419	IL1RAP C3orf13 IL1R3
P67812	0.109614372	1.436049141	SEC11A SEC11L1 SPC18 SPCS4A
Q10713	0.109689713	2.286382007	PMPCA INPP5E KIAA0123 MPPA
Q6UWE0	0.109704971	1.339995942	LRSAM1 TAL UNQ6496/PRO21356
Q9UPT5	0.109964371	0.482548373	EXOC7 EXO70 KIAA1067
Q5T1M5	0.110138893	0.963357406	FKBP15 KIAA0674
Q7Z3T8	0.110191345	1.193268895	ZFYVE16 KIAA0305
Q16774	0.110360146	0.997062315	GUK1 GMK GMPK
Q9BR76	0.110600471	0.892475805	CORO1B
Q6UW02	0.110798836	0.381571001	CYP20A1 UNQ667/PRO1301
Q9NYU2	0.111020088	1.905207145	UGGT1 GT UGCGL1 UGGT UGT1 UGTR
P61160	0.111183167	1.397509909	ACTR2 ARP2
Q8WVC0	0.111217499	0.886207365	LEO1 RDL
O94919	0.111366749	1.427848182	ENDOD1 KIAA0830
Q7Z6L1	0.111479282	0.477064634	TECPR1 KIAA1358
Q96EP0	0.111712456	0.630809222	RNF31 ZIBRA
Q14789	0.112194061	2.4935961	GOLGB1
Q8N766	0.112280846	1.302474496	EMC1 KIAA0090 PSEC0263
P33527	0.112417221	2.79805857	ABCC1 MRP MRP1

Q9H6U6	0.112531662	0.915431735	BCAS3
Q9H857	0.112561226	0.797684965	NT5DC2
P32519	0.112689972	0.224504308	ELF1
Q969V3	0.113002777	0.88639518	NCLN
Q8IYB7	0.113009453	0.26637385	DIS3L2 FAM6A
P53680	0.113251686	3.477854126	AP2S1 AP17 CLAPS2
P83436	0.113302231	0.67920153	COG7 UNQ3082/PRO10013
O94925	0.113705635	1.44032727	GLS GLS1 KIAA0838
Q96EI5	0.113819599	0.617269337	TCEAL4 NPD017
Q7Z569	0.114362717	1.016331546	BRAP RNF52
Q8N6T3	0.114406586	0.624656161	ARFGAP1 ARF1GAP
Q9Y2Z0	0.114445686	0.982163743	SUGT1
Q16537	0.114530563	1.451367935	PPP2R5E
Q9UNX4	0.114701271	0.956172949	WDR3
P11117	0.114804268	1.4159092	ACP2
Q9BXS5	0.114984512	0.887859914	AP1M1 CLTNM
Q9NSC5	0.115042686	0.78186697	HOMER3
Q96N66	0.115269661	1.719410743	MBOAT7 BB1 LENG4 OACT7
P31937	0.115339279	1.198135808	HIBADH
P78344	0.115351677	1.064560011	EIF4G2 DAP5 OK/SW-cl.75
Q9BRA0	0.115400791	0.668186066	NAA38 LSMD1 MAK31 PFAAP2
P02461	0.115420341	1.661184871	COL3A1
P10398	0.115652084	0.92424518	ARAF ARAF1 PKS PKS2
Q8NF91	0.115877151	2.145478585	SYNE1 C6orf98 KIAA0796 KIAA1262 KIAA1756 MYNE1
Q9ULP9	0.115943909	1.042482197	TBC1D24 KIAA1171
O75164	0.116071224	0.680553172	KDM4A JHDM3A JMJD2 JMJD2A KIAA0677
Q7L3B6	0.116205215	0.917270119	CDC37L1 CDC37B HARC
Q8NFF5	0.11626339	0.744207089	FLAD1 PP591
O95747	0.116365433	0.291855678	OXSR1 KIAA1101 OSR1
Q14011	0.116412163	1.939161457	CIRBP A18HNRNP CIRP
P53992	0.116497993	2.052616714	SEC24C KIAA0079
P46109	0.116545677	2.068671986	CRKL
Q96CN4	0.116617203	0.835013374	EVI5L
Q9BYC9	0.116724968	1.476040485	MRPL20
Q96CD2	0.11684227	0.738051709	PPCDC COAC MDS018 UNQ9365/PRO34154
Q9H2P9	0.11707592	0.497886604	DPH5 AD-018 CGI-30 HSPC143 NPD015
Q16832	0.117178917	1.780129291	DDR2 NTRKR3 TKT TYRO10
P26038	0.117321968	1.364598779	MSN
Q9UI26	0.118103981	0.392732021	IPO11 RANBP11
O75312	0.11838913	0.315658996	ZPR1 ZNF259
Q9UJY5	0.118443489	1.330312762	GGA1
P51805	0.118555069	0.905880712	PLXNA3 PLXN4 SEX

Q02388	0.118605137	0.201266445	COL7A1
P25098	0.118684769	1.951242344	GRK2 ADRBK1 BARK BARK1
Q15262	0.118904114	1.331673477	PTPRK PTPK
Q9UKS6	0.119024277	0.128866546	PACSIN3
Q96BH1	0.11913681	0.525685073	RNF25
Q8TDJ6	0.119143486	1.104099088	DMXL2 KIAA0856
Q9NRY4	0.11914444	3.441477446	ARHGAP35 GRF1 GRLF1 KIAA1722 P190A p190ARHOGAP
P40926	0.119379997	0.999942451	MDH2
Q7L2E3	0.119439125	1.963799934	DHX30 DDX30 KIAA0890
Q13057	0.119564056	0.822611624	COASY PSEC0106
Q12929	0.119590759	1.279662602	EPS8
Q14247	0.119716644	0.524638259	CTTN EMS1
Q96D46	0.119840622	1.170319977	NMD3 CGI-07
Q12846	0.119895935	1.048253185	STX4 STX4A
P11166	0.119959831	1.505693313	SLC2A1 GLUT1
P51659	0.119996071	2.672539897	HSD17B4 EDH17B4 SDR8C1
P07099	0.120125771	2.799369185	EPHX1 EPHX EPOX
Q9H4M9	0.120335579	2.411826557	EHD1 PAST PAST1 CDABP0131
P28799	0.120340347	1.487461894	GRN
Q8WW59	0.120364189	0.655308176	SPRYD4
Q9UBP6	0.120473862	0.94214249	METTL1 C12orf1
Q96KM6	0.120512962	0.73781749	ZNF512B KIAA1196
Q96L92	0.12054348	1.068437437	SNX27 KIAA0488 My014
Q12824	0.120573044	0.514824059	SMARCB1 BAF47 INI1 SNF5L1
Q86TU7	0.121142387	1.028712842	SETD3 C14orf154
Q9H993	0.121296883	1.271546394	ARMT1 C6orf211
Q15056	0.121299744	1.737575295	EIF4H KIAA0038 WBSCR1 WSCR1
Q16555	0.121366501	1.912487201	DPYSL2 CRMP2 ULIP2
Q8TCT9	0.121418953	1.36604077	HM13 H13 IMP1 PSL3 SPP MSTP086
Q96IF1	0.121446609	1.27920245	AJUBA JUB
Q8NFD5	0.121534348	0.5563004	ARID1B BAF250B DAN15 KIAA1235 OSA2
Q96K76	0.121671677	1.29641935	USP47
Q9GZY8	0.121704102	0.242830446	MFF C2orf33 AD030 AD033 GL004
Q6PD62	0.121749878	0.880018744	CTR9 KIAA0155 SH2BP1
Q86X27	0.121750355	0.999705619	RALGPS2
Q96JI7	0.121756077	0.211001367	SPG11 KIAA1840
Q99549	0.122276783	0.520492722	MPHOSPH8 MPP8
O00622	0.122502327	1.224678878	CCN1 CYR61 GIG1 IGFBP10
Q5HYK7	0.122605324	1.89844421	SH3D19
Q9UPP1	0.122833729	0.198882551	PHF8 KIAA1111 ZNF422
Q9NRL3	0.123066902	1.589448688	STRN4 ZIN
P17677	0.123120308	0.315623553	GAP43
Q9Y305	0.123140335	1.883802952	ACOT9 CGI-16

Q8NBJ7	0.123274803	1.87471796	SUMF2 PSEC0171 UNQ1968/PRO4500
Q9NW13	0.123657227	2.688326449	RBM28
Q9HD15	0.124121666	0.883127118	SRA1 PP7684
Q99715	0.124206543	0.676666758	COL12A1 COL12A1L
Q9Y5K8	0.124237061	2.611863561	ATP6V1D ATP6M VATD
Q99523	0.124324799	0.482193474	SORT1
Q12765	0.124387741	2.135674037	SCRN1 KIAA0193
Q99470	0.124401093	1.001090641	SDF2
Q9UBS4	0.12446785	1.065943908	DNAJB11 EDJ ERJ3 HDJ9 PSEC0121 UNQ537/PRO1080
Q9HCN4	0.12451458	0.736244962	GPN1 MBDIN RPAP4 XAB1 HUSSY-23
Q9Y4G2	0.124763012	0.225936749	PLEKHM1 KIAA0356
Q9P0K7	0.124834061	1.731898047	RAI14 KIAA1334 NORPEG
Q8TBX8	0.125155449	1.076584453	PIP4K2C PIP5K2C
Q14126	0.12532711	1.699543757	DSG2 CDHF5
Q96C12	0.125499249	0.100921789	ARMC5
Q86UV5	0.125587463	0.226852305	USP48 USP31
P35914	0.125645638	1.378640201	HMGCL
Q9BRR6	0.125710487	2.87142584	ADPGK PSEC0260
Q16762	0.1258955	2.862301584	TST
P12955	0.125945091	2.416499261	PEPD PRD
Q9UMX0	0.12603569	1.646240764	UBQLN1 DA41 PLIC1
Q13610	0.126163483	2.081209519	PWP1
Q9Y240	0.12621069	1.259464843	CLEC11A CLECSF3 LSLCL SCGF
Q02218	0.126259804	2.69869439	OGDH
P10909	0.126422882	2.15767577	CLU APOJ CLI KUB1 AAG4
O94964	0.126434326	0.649148304	SOGA1 C20orf117 KIAA0889 SOGA
Q92859	0.126570702	0.65052728	NEO1 IGDCC2 NGN
Q99611	0.126596451	0.130142847	SEPHS2 SPS2
Q86UY6	0.126953125	1.057818144	NAA40 NAT11 PATT1
Q14137	0.126963615	2.270080501	BOP1 KIAA0124
Q9UH62	0.127296448	1.316840595	ARMCX3 ALEX3 BM-017 UNQ2517/PRO6007
Q9Y4D8	0.127378941	0.832623857	HECTD4 C12orf51 KIAA0614
P55084	0.127408028	1.22751731	HADHB MSTP029
Q8IYB5	0.127464294	1.149331679	SMAP1
O95248	0.127498627	1.614746896	SBF1 MTMR5
Q02750	0.127536774	1.127916017	MAP2K1 MEK1 PRKMK1
Q9Y6N7	0.127551079	1.106423528	ROBO1 DUTT1
Q8N6Y2	0.127990723	0.450014471	LRRC17 P37NB UNQ3076/PRO9909
Q01658	0.127997398	1.174611107	DR1
Q15477	0.128334045	2.673084729	SKIV2L DDX13 SKI2W SKIV2 W
Q9HCK8	0.128435135	0.458368895	CHD8 HELSNF1 KIAA1564
P21359	0.128647804	1.124570479	NF1

Q6UB35	0.12866497	3.238920049	MTHFD1L FTHFSDC1
Q8WXF7	0.12869072	1.009876985	ATL1 GBP3 SPG3A
P49748	0.128692627	1.251666092	ACADVL VLCAD
Q9UI15	0.128990173	0.871426968	TAGLN3 NP25
Q9BV81	0.129109383	0.46713036	EMC6 TMEM93
Q9NVE7	0.129115105	0.861833332	PANK4
Q7Z7M9	0.129266739	1.150002882	GALNT5
P62333	0.1292696	1.765226294	PSMC6 SUG2
Q7KZF4	0.12936306	1.893877136	SND1 TDRD11
P33121	0.129365921	1.752290205	ACSL1 FACL1 FACL2 LACS LACS1 LACS2
Q92791	0.129454613	2.354121161	P3H4 LEPREL4 NOL55 SC65
Q8IVH4	0.12947464	2.359900768	MMAA
P51580	0.129673958	1.419637911	TPMT
P52789	0.129798889	0.972562718	HK2
Q8IXB1	0.130029678	3.02839009	DNAJC10 ERDJ5 UNQ495/PRO1012
P53677	0.130228996	0.765637088	AP3M2
P07384	0.130336761	1.160050084	CAPN1 CANPL1 PIG30
P21266	0.130361557	1.71175637	GSTM3 GST5
P84157	0.130580902	0.940237264	MXRA7
Q9NR50	0.130636215	2.006569702	EIF2B3
O15397	0.130672455	1.5381615	IPO8 RANBP8
O14786	0.130866051	1.772560069	NRP1 NRP VEGF165R
Q8N684	0.130895615	1.886619054	CPSF7
Q96DV4	0.130897522	1.488498931	MRPL38 HSPC262
Q96AQ6	0.130905151	1.303361558	PBXIP1 HPIP
Q96B97	0.130997658	1.441430742	SH3KBP1 CIN85
Q14108	0.13104248	0.436947178	SCARB2 CD36L2 LIMP2 LIMPII
Q8N3S3	0.131162643	0.388250539	PHTF2
Q9BY43	0.131237984	2.305397332	CHMP4A C14orf123 SHAX2 CDA04 HSPC134
Q96B36	0.131241798	2.437112742	AKT1S1 PRAS40
O00330	0.131341934	0.75503137	PDHX PDX1
Q9BZ67	0.131472588	1.176815005	FRMD8 FKSG44
Q8NE86	0.131498337	2.172023251	MCU C10orf42 CCDC109A
Q93008	0.131523132	1.660094622	USP9X DFFRX FAM USP9
Q6RW13	0.131606102	0.893248497	AGTRAP ATRAP
P40939	0.131615639	0.986842975	HADHA HADH
O75351	0.131714821	1.541333223	VPS4B SKD1 VPS42 MIG1
Q8N3Z6	0.131745338	0.383094589	ZCCHC7 HSPC086
O75817	0.131813049	1.419619809	POP7 RPP20
P56537	0.131817818	0.523149523	EIF6 EIF3A ITGB4BP OK/SW-cl 27
P46821	0.131885529	1.079838861	MAP1B
Q13724	0.132012367	2.140570134	MOGS GCS1
Q15678	0.132174492	0.477995206	PTPN14 PEZ PTPD2

Q13643	0.132418633	1.438456827	FHL3 SLIM2
Q8N2F6	0.133206367	1.097485684	ARMC10 SVH PSEC0198
Q14847	0.133428574	0.835200664	LASP1 MLN50
Q9Y295	0.133447647	1.504704268	DRG1 NEDD3
Q9UJS0	0.133534431	1.704507231	SLC25A13 ARALAR2
Q92574	0.133574486	1.808128333	TSC1 KIAA0243 TSC
O75844	0.133592606	1.008688929	ZMPSTE24 FACE1 STE24
Q5R372	0.13367939	1.963910002	RABGAP1L HHL KIAA0471
Q9BT17	0.133788109	0.239424161	MTG1 GTPBP7
Q8NEZ2	0.13403511	0.661365001	VPS37A HCRP1
Q8NBX0	0.134745598	1.839071548	SCCPDH CGI-49
Q9H1I8	0.134792328	1.807328081	ASCC2 ASC1P100 RQT3
Q9Y2Q3	0.135142326	2.704365021	GSTK1 HDCMD47P
O60879	0.135260582	0.708916316	DIAPH2 DIA
O94766	0.135374069	1.461192184	B3GAT3
P50570	0.135391235	1.768115276	DNM2 DYN2
Q9NQT8	0.135409355	1.609279966	KIF13B GAKIN KIAA0639
Q676U5	0.135436058	2.170244217	ATG16L1 APG16L UNQ9393/PRO34307
P54802	0.135453224	2.502033474	NAGLU UFHSD1
Q9UL25	0.135806084	1.374487143	RAB21 KIAA0118
P62993	0.135971069	1.878131846	GRB2 ASH
Q2TB10	0.136102676	1.186401499	ZNF800 PP902
Q14997	0.136458397	1.274091058	PSME4 KIAA0077
O14686	0.136475563	1.98438915	KMT2D ALR MLL2 MLL4
O14617	0.136704445	1.68612979	AP3D1 PRO0039
Q92973	0.13670826	2.565742636	TNPO1 KPNB2 MIP1 TRN
Q5T2E6	0.136752129	1.907561864	ARMH3 C10orf76
P54289	0.137003899	1.896631432	CACNA2D1 CACNL2A CCHL2A MHS3
Q9H1C3	0.137120247	0.871700624	GLT8D2 GALA4A UNQ1901/PRO4347
Q8IW45	0.137214661	1.872517787	NAXD CARKD
Q8TDD1	0.137571335	1.415025267	DDX54
P09132	0.137656212	0.648664839	SRP19
P00491	0.137735367	1.732304428	PNP NP
Q9BR61	0.13796711	0.836173427	ACBD6
Q14746	0.137991905	1.295076954	COG2 LDLC
Q12899	0.138001442	1.851894423	TRIM26 RNF95 ZNF173
P45974	0.138134956	2.333780515	USP5 ISOT
Q96Q05	0.138208389	0.885344276	TRAPPC9 KIAA1882 NIBP T1
Q53FP2	0.138354301	1.836505501	TMEM35A NACHO TMEM35
Q6P1L8	0.138362885	0.848911477	MRPL14 MRPL32 RPML32
Q7LG56	0.138510704	0.998344863	RRM2B P53R2
Q9NXC5	0.138606071	0.716552687	MIOS
Q96F86	0.138619423	1.482864596	EDC3 LSM16 YJDC YJEFN2 PP844

Q06190	0.13873148	0.579231374	PPP2R3A PPP2R3
Q15124	0.138784885	0.239010628	PGM5 PGMRP
Q6XE24	0.138816357	0.391240913	RBMS3
P37802	0.138853073	0.706072675	TAGLN2 KIAA0120 CDABP0035
Q8WVM8	0.138988495	2.464188257	SCFD1 C14orf163 KIAA0917 STXBP1L2 FKSG23
Q5SY16	0.139069557	2.793485232	NOL9
O14920	0.139072418	1.337319579	IKBKB IKKB
Q9UHD1	0.1391325	1.492762051	CHORDC1 CHP1
Q8N4Q0	0.139146805	0.647368063	ZADH2 PTGR3
P51798	0.139186859	1.489155934	CLCN7
Q7Z5G4	0.13923645	1.379795613	GOLGA7 GCP16 HDCKB03P HSPC041
Q15650	0.139416695	1.70458058	TRIP4 RQT4
O94915	0.139425278	2.8004437	FRYL AF4P12 KIAA0826
P78536	0.139715195	0.907738475	ADAM17 CSVP TACE
Q9Y223	0.139748573	2.149408938	GNE GLCNE
Q08397	0.139756203	0.822357087	LOXL1 LOXL
Q8IV08	0.140143394	2.162733135	PLD3
Q6YP21	0.140221596	1.889868802	KYAT3 CCBL2 KAT3
Q92508	0.140295506	1.230222903	PIEZO1 FAM38A KIAA0233
Q9Y2S2	0.140843391	0.220535014	CRYL1 CRY
O75439	0.140845299	2.513562103	PMPCB MPPB
Q96AX1	0.14085865	1.289618297	VPS33A
P35998	0.140997887	1.924950779	PSMC2 MSS1
Q8N668	0.141036034	1.038363747	COMMD1 C2orf5 MURR1
P07814	0.14118576	1.775201924	EPRS1 EPRS GLNS PARS QARS QPRS PIG32
Q5JSZ5	0.141433239	0.340470137	PRRC2B BAT2L BAT2L1 KIAA0515
Q13043	0.141500473	0.893689093	STK4 KRS2 MST1
Q9Y281	0.14157486	1.088513426	CFL2
O15260	0.141951561	0.604057121	SURF4 SURF-4
P49590	0.142160416	0.923243996	HARS2 HARSL HARSR HO3
O15126	0.142314911	1.182884826	SCAMP1 SCAMP
Q12959	0.142413139	0.906031391	DLG1
Q96TA2	0.142699242	2.738792377	YME1L1 FTSH1 YME1L UNQ1868/PRO4304
P23458	0.142708778	0.767121451	JAK1 JAK1A JAK1B
O00151	0.142726898	1.819868116	PDLIM1 CLIM1 CLP36
Q96N67	0.142875671	2.537592144	DOCK7 KIAA1771
P08648	0.142995834	2.909521681	ITGA5 FNRA
P40855	0.143445969	1.753171867	PEX19 HK33 PXF OK/SW-cl.22
Q9BT73	0.143578529	1.1099357	PSMG3 C7orf48 PAC3
P0C7T5	0.143705845	1.935168777	ATXN1L BOAT BOAT1
O00468	0.143903732	0.358201538	AGRN AGRIN
P17612	0.14397049	0.328433561	PRKACA PKACA

Q13586	0.144242287	1.330937429	STIM1 GOK
P49754	0.144246101	1.077376936	VPS41
O43149	0.144298553	0.663067483	ZZEF1 KIAA0399
Q8IX18	0.144599438	0.216218473	DHX40 DDX40 ARG147
Q8IXI1	0.144699097	1.432757863	RHOT2 ARHT2 C16orf39
Q5JSH3	0.144730568	1.29641284	WDR44
Q8WZ82	0.14474678	2.056375608	OVCA2
Q9Y639	0.144756317	1.900906363	NPTN SDFR1 SDR1
Q01459	0.144824028	1.079691193	CTBS CTB
Q9NP72	0.144948006	2.526431679	RAB18
Q9NRV9	0.145064354	2.151216611	HEBP1 HBP
O00115	0.145076752	1.365456351	DNASE2 DNASE2A DNL2
O94822	0.145112991	1.162942205	LTN1 C21orf10 C21orf98 KIAA0714 RNF160 ZNF294 HSPC087
P61764	0.145120621	2.02172752	STXBP1 UNC18A
Q9UGU5	0.145479202	0.564384215	HMGXB4 HMG2L1 HMGBCG
Q9Y485	0.145860672	1.035915171	DMXL1 XL1
Q05655	0.145874023	1.232283704	PRKCD PKCD
O15144	0.145886421	0.871209243	ARPC2 ARC34 PRO2446
O75179	0.146127701	1.500790924	ANKRD17 GTAR KIAA0697
Q68DQ2	0.146238327	2.048855589	CRYBG3
Q15006	0.146285057	1.514550265	EMC2 KIAA0103 TTC35
Q08379	0.146291733	1.737438004	GOLGA2
Q9Y490	0.146452904	2.016752192	TLN1 KIAA1027 TLN
P28288	0.146492958	1.530827818	ABCD3 PMP70 PXMP1
O43292	0.146521568	0.877499174	GPAA1 GAA1
Q5MNZ6	0.146573067	1.451750766	WDR45B WDR45L WIPI3
Q9NVR0	0.146659374	0.58133932	KLHL11
P30626	0.146838188	1.15531193	SRI
O43572	0.146855354	0.658542306	AKAP10
Q9Y6D5	0.14719677	1.77731235	ARFGEF2 ARFGEP2 BIG2
Q9HAB8	0.147393227	1.126727624	PPCS COAB
O94804	0.14810276	0.797631967	STK10 LOK
O43752	0.14812851	0.750848495	STX6
Q9Y5Q0	0.148253441	0.564176734	FADS3 CYB5RP
Q9Y2D4	0.148745537	1.387644654	EXOC6B KIAA0919 SEC15B SEC15L2
P54577	0.148759842	1.340583298	YARS1 YARS
Q9H1P3	0.148768425	1.037507934	OSBPL2 KIAA0772 ORP2
Q6AWC2	0.148797989	1.193897763	WWC2 BOMB
Q86V21	0.148803711	0.912600541	AACS ACSF1
Q9UEG4	0.148933411	0.835240704	ZNF629 KIAA0326 ZNF65
O43402	0.149056435	2.017868905	EMC8 C16orf2 C16orf4 COX4AL COX4NB FAM158B NOC4
Q86WG5	0.149546623	2.565490671	SBF2 CMT4B2 KIAA1766 MTMR13

P41240	0.149726868	2.348562808	CSK
Q9NNW7	0.149889946	1.272448459	TXNRD2 KIAA1652 TRXR2
Q15904	0.149900436	0.571776565	ATP6AP1 ATP6IP1 ATP6S1 VATPS1 XAP3
P07305	0.150037766	1.560299531	H1-0 H1F0 H1FV
Q15334	0.15007019	1.681448527	LLGL1 DLG4 HUGL HUGL1
O75190	0.150157928	1.493420138	DNAJB6 HSJ2 MRJ MSJ1
Q9NSE4	0.150265694	3.475812149	IARS2
O75427	0.150288582	1.872344614	LRCH4 LRN LRRN1 LRRN4
Q5TAX3	0.150369644	1.860403554	TUT4 KIAA0191 ZCCHC11
Q9BSJ8	0.150384903	1.732473175	ESYT1 FAM62A KIAA0747 MBC2
Q8WWV3	0.150391579	0.692882406	RTN4IP1 NIMP
Q9UNW1	0.150541306	1.295390761	MINPP1 MIPP UNQ900/PRO1917
Q96EY5	0.15054512	1.480337793	MVB12A CFBP FAM125A
Q15738	0.150800705	1.848200683	NSDHL H105E3
Q9NX08	0.150935173	1.648113374	COMMD8 MDS022
Q96GG9	0.150948524	1.256177639	DCUN1D1 DCN1 DCUN1L1 RP42 SCCRO
Q9NW15	0.151047707	1.313537951	ANO10 TMEM16K
Q8IWX8	0.151292801	1.673882891	CHERP DAN26 SCAF6
P68366	0.15137291	1.527929024	TUBA4A TUBA1
Q00403	0.152013779	3.417393005	GTF2B TF2B TFIIB
Q9UQN3	0.152340889	2.018817101	CHMP2B CGI-84
Q9BTE1	0.152438164	1.332504676	DCTN5
Q9H2M9	0.152661324	2.459865709	RAB3GAP2 KIAA0839
Q08380	0.152667046	1.397492638	LGALS3BP M2BP
Q9BW91	0.152823448	1.624484901	NUDT9 NUDT10 PSEC0099 UNQ3012/PRO9771
Q01831	0.152900696	1.641228347	XPC XPCC
Q9NPA0	0.152921677	1.854384884	EMC7 C11orf3 C15orf24 HT022 UNQ905/PRO1926
Q9P2R7	0.153030396	1.507102231	SUCLA2
Q96CW1	0.153153419	1.63735462	AP2M1 CLAPM1 KIAA0109
Q9Y5J6	0.153162479	1.296793994	TIMM10B FXC1 TIM9B TIMM9B
P12814	0.153164864	2.848527084	ACTN1
Q8TAE8	0.153244972	1.047265012	GADD45GIP1 MRPL59 PLINP1 PRG6
O60831	0.153250694	1.258242257	PRAF2 JM4
Q5T5C0	0.153291702	1.522778149	STXBP5 LLGL3
Q9H4A4	0.153436661	2.284183368	RNPEP APB
Q02447	0.153438568	1.075995399	SP3
Q07065	0.153494835	2.177589482	CKAP4
P30533	0.153558731	1.243169517	LRPAP1 A2MRAP
O75528	0.153956413	1.23596085	TADA3 ADA3 TADA3L
Q9BRA2	0.154169083	0.837530237	TXNDC17 TXNL5
Q9Y6G9	0.15426445	1.090344144	DYNC1LI1 DNCLI1
Q9NPJ3	0.154308319	1.260788719	ACOT13 THEM2 HT012 PNAS-27

Q9Y276	0.154469013	1.300347894	BCS1L BCS1
Q8WTW3	0.154612541	1.298186063	COG1 KIAA1381 LDLB
Q9NUL7	0.154621124	0.666506992	DDX28 MDDX28
Q9NNW5	0.154719353	1.072762201	WDR6
Q9UNF0	0.154967308	1.615874428	PACSIN2
P42025	0.155118942	1.071006833	ACTR1B CTRN2
Q4G0N4	0.155185699	2.640816575	NADK2 C5orf33 MNADK NADKD1
Q8TCD5	0.155201912	0.454967012	NT5C DNT1 UMPH2
Q9Y679	0.155344963	1.146817471	AUP1
Q08378	0.155578613	1.943889668	GOLGA3
P18615	0.155617714	0.616819423	NELFE RD RDBP
O75832	0.155719757	0.954182915	PSMD10
Q99501	0.155737877	0.321148158	GAS2L1 GAR22
Q5SSJ5	0.155917168	1.211736175	HP1BP3
Q13404	0.156142235	2.899213744	UBE2V1 CROC1 UBE2V UEV1 P/OKcl.19
Q7L311	0.156366348	1.130347343	ARMCX2 ALEX2 KIAA0512
O75155	0.15641737	0.397132059	CAND2 KIAA0667 TIP120B
Q15067	0.156452179	1.260021164	ACOX1 ACOX
Q7Z2Z2	0.156594276	1.325180513	EFL1 EFTUD1 FAM42A
O00629	0.156604767	1.214570865	KPNA4 QIP1
Q9BQ95	0.15666151	0.73192497	ECSIT
P42356	0.156713486	0.548061228	PI4KA PIK4 PIK4CA
O95749	0.156945229	2.568465293	GGPS1
Q9H269	0.15719986	2.407641428	VPS16
Q96HY7	0.157269478	0.855600734	DHTKD1 KIAA1630
Q96TC7	0.157359123	1.073284407	RMDN3 FAM82A2 FAM82C PTPIP51 hucep-10 UNQ3122/PRO10274
Q9Y3B8	0.157635689	0.882160674	REXO2 SFN SMFN CGI-114
P57081	0.157679558	0.969531122	WDR4
Q9BY32	0.157773972	1.295904146	ITPA C20orf37 My049 OK/SW-cl.9
P22694	0.158341408	1.710120691	PRKACB
Q9BSD7	0.158455849	1.892183512	NTPCR C1orf57
Q7L211	0.158493042	0.584503431	ABHD13 C13orf6
Q5K651	0.158547401	1.504369553	SAMD9 C7orf5 DRIF1 KIAA2004 OEF1
Q8IXK0	0.158550262	1.477456209	PHC2 EDR2 PH2
P17535	0.158594131	0.899926566	JUND
Q9NZT2	0.15893364	1.363576964	OGFR
O43852	0.159065247	1.556737001	CALU
P82675	0.159376144	1.114606798	MRPS5
P63098	0.159379005	1.548458972	PPP3R1 CNA2 CNB
Q93063	0.159381866	2.778627596	EXT2
Q9H4G4	0.159420967	1.064365991	GLIPR2 C9orf19 GAPR1
Q7L8J4	0.159498215	0.755276241	SH3BP5L KIAA1720 UNQ2766/PRO7133
P32418	0.159651756	0.708926994	SLC8A1 CNC NCX1

Q53GQ0	0.159692764	1.491408938	HSD17B12 SDR12C1
O95834	0.159992218	0.880158362	EML2 EMAP2 EMAPL2
Q96RE7	0.16016674	0.906792959	NACC1 BTBD14B NAC1
Q07889	0.16027832	0.786857635	SOS1
P18858	0.160365105	0.86812516	LIG1
Q7L0J3	0.160531998	0.581128792	SV2A KIAA0736 PSEC0174
Q8IVL5	0.160635948	0.9999953	P3H2 LEPREL1 MLAT4
P08237	0.160752296	2.141397917	PFKM PFKX
Q8TB22	0.161234856	1.86193603	SPATA20
P51148	0.16130352	2.680398697	RAB5C RABL
Q3LXA3	0.16193676	0.880071939	TKFC DAK
P23508	0.161984444	1.912090496	MCC
P11182	0.162431717	1.604054076	DBT BCATE2
O60507	0.162512779	1.536393115	TPST1
P83111	0.1633358	2.113454377	LACTB MRPL56 UNQ843/PRO1781
P28290	0.163467407	1.773278149	ITPRID2 CS1 KIAA1927 KRAP SSFA2
Q8IYS1	0.163601398	1.081035954	PM20D2 ACY1L2
Q9UBV8	0.163811684	2.217897858	PEF1 ABP32 UNQ1845/PRO3573
A1L0T0	0.163864136	2.135668098	ILVBL AHAS HACL2
Q641Q2	0.164129257	0.827993363	WASHC2A FAM21A FAM21B
Q9H3P7	0.164293289	2.238902774	ACBD3 GCP60 GOCAP1 GOLPH1
O95208	0.164307594	1.195680514	EPN2 KIAA1065
Q9P2W9	0.16444397	1.38701791	STX18 GIG9
P07711	0.164530754	0.762234134	CTSL CTSL1
Q9UBS8	0.164959908	2.006820758	RNF14 ARA54 HRIHFB2038
Q9NPH2	0.165054321	0.971015281	ISYNA1 INO1
Q96KP1	0.165117264	1.866905476	EXOC2 SEC5 SEC5L1
Q9UHY1	0.165366173	0.980956053	NRBP1 BCON3 NRBP
P31689	0.165472031	2.292270246	DNAJA1 DNAJ2 HDJ2 HSJ2 HSPF4
Q96PE7	0.165734291	2.868228342	MCEE
Q8WVT3	0.166173935	1.38154648	TRAPPC12 TRAMM TTC15 CGI-87
Q9NUQ7	0.166506767	1.048113713	UFSP2 C4orf20
Q96SB3	0.166622162	2.276475973	PPP1R9B PPP1R6
Q9H0V9	0.166629791	0.448615024	LMAN2L VIPL PSEC0028 UNQ368/PRO704
O95219	0.166641235	1.972006646	SNX4
Q9H173	0.166694641	0.530383187	SIL1 UNQ545/PRO836
Q8NBN3	0.166733742	1.130297064	TMEM87A PSEC0094
Q8N6R0	0.16696167	0.899435247	METTL13 EEF1AKNMT FEAT KIAA0859 CGI-01
Q9Y6K0	0.166969299	0.793926941	CEPT1 PRO1101
Q9NV31	0.167120934	0.808118187	IMP3 C15orf12 MRPS4
Q9NW68	0.167122841	1.833266255	BSDC1 UNQ2494/PRO5781
Q9HD26	0.167251587	0.997520484	GOPC CAL FIG
O95905	0.167535305	1.19207178	ECD

Q8NDA8	0.167578697	0.629609296	MROH1 HEATR7A KIAA1833
Q9Y2I1	0.167732716	0.941006247	NISCH IRAS KIAA0975
O95503	0.167749882	0.459614672	CBX6
O75157	0.167822838	1.221204661	TSC22D2 KIAA0669 TILZ4
P55786	0.167964935	1.454038012	NPEPPS PSA
Q14139	0.168358803	1.429263898	UBE4A KIAA0126
Q9Y3M8	0.168444633	1.09461683	STARD13 DLC2 GT650
Q96S52	0.168829918	1.299523403	PIGS UNQ1873/PRO4316
Q15417	0.168839455	2.091270842	CNN3
O00443	0.16901207	1.396778151	PIK3C2A
Q9NV70	0.169361115	1.600226143	EXOC1 SEC3 SEC3L1 BM-012
P49840	0.169607162	1.244997092	GSK3A
Q96IJ6	0.169670105	1.63204564	GMPPA
O15066	0.169678688	0.685953832	KIF3B KIAA0359
Q53EP0	0.169920921	1.851562144	FNDC3B FAD104 NS5ABP37 UNQ2421/PRO4979/PRO34274
Q96DZ1	0.170180321	1.011716362	ERLEC1 C2orf30 XTP3TPB UNQ1878/PRO4321
Q96RF0	0.170248032	1.000859971	SNX18 SH3PXD3B
O75436	0.170283318	1.756468768	VPS26A VPS26
P62136	0.170383453	1.086172517	PPP1CA PPP1A
O14964	0.170391083	1.603423015	HGS HRS
Q96HP0	0.170522213	3.375794566	DOCK6 KIAA1395
Q8IXI2	0.170560837	1.64325797	RHOT1 ARHT1
Q32P44	0.17098999	1.339349902	EML3
P40123	0.171177864	1.575120348	CAP2
O43715	0.171211243	0.95191675	TRIAP1 15E1.1 HSPC132
Q9HBF4	0.171387672	0.543470837	ZFYVE1 DFCP1 KIAA1589 TAFF1 ZNFN2A1 PP10436
Q9H270	0.171517372	0.849649874	VPS11 RNF108 PP3476
Q08431	0.17164135	1.164025063	MFGE8
P15289	0.171777725	1.448783392	ARSA
Q8N4V1	0.17202282	1.483971424	MMGT1 EMC5 TMEM32
P22059	0.172141075	2.788917343	OSBP OSBP1
Q96AG3	0.172387123	0.604695801	SLC25A46 TB1
Q16204	0.172451019	1.820144489	CCDC6 D10S170 TST1
P49137	0.172863007	2.270001599	MAPKAPK2
Q9HB90	0.172887325	0.446677541	RRAGC
Q9C0I1	0.17294693	1.197678469	MTMR12 KIAA1682 PIP3AP
O43159	0.17304039	0.992567792	RRP8 KIAA0409 NML hucep-1
Q92542	0.173176765	1.463441739	NCSTN KIAA0253 UNQ1874/PRO4317
Q05397	0.173536301	3.459188537	PTK2 FAK FAK1
P51553	0.173707008	1.71373674	IDH3G
Q14696	0.173978806	2.797648468	MESD KIAA0081 MESDC2 MESDM UNQ1911/PRO4369

Q9UPZ3	0.174049377	0.420827596	HPS5 AIBP63 KIAA1017
Q13325	0.174351692	1.524776439	IFIT5 ISG58 RI58
O95140	0.174363136	1.535547523	MFN2 CPRP1 KIAA0214
P16035	0.174407959	1.377912995	TIMP2
Q96CS3	0.174512863	1.72346926	FAF2 ETEA KIAA0887 UBXD8 UBXN3B
Q9UJ41	0.174624443	1.51090198	RABGEF1 RABEX5
Q969S9	0.174646378	1.622392748	GFM2 EFG2 MSTP027
Q9ULV4	0.174764633	1.088718344	CORO1C CRN2 CRNN4
P42704	0.175106049	1.882731308	LRPPRC LRP130
P48147	0.175160408	1.419558899	PREP PEP
Q99733	0.175383568	1.855309242	NAP1L4 NAP2
O14976	0.175497055	1.445053441	GAK
Q9BSU1	0.175528526	2.463089799	PHAF1 C16orf6 C16orf70
Q70UQ0	0.176034927	1.572348557	IKBIP IKIP
Q9BXR0	0.176435471	1.424555738	QTRT1 TGT TGUT
P34949	0.176540375	1.352219392	MPI PMI1
Q13085	0.17663002	1.93121103	ACACA ACAC ACC1 ACCA
Q13459	0.176991463	2.505953082	MYO9B MYR5
Q9H553	0.177082539	2.124728823	ALG2 UNQ666/PRO1298
A2VDF0	0.177174091	0.635459034	FUOM C10orf125
Q9ULR0	0.177205086	1.468257378	ISY1 KIAA1160
O60218	0.177407265	0.938533269	AKR1B10 AKR1B11
Q9UHD2	0.177458763	2.039180742	TBK1 NAK
Q14693	0.177502632	1.974050163	LPIN1 KIAA0188
P55036	0.177513123	1.901355254	PSMD4 MCB1
Q9BQE3	0.177750587	1.38330566	TUBA1C TUBA6
P09497	0.177798271	1.983954744	CLTB
Q8TF05	0.177882195	1.716359097	PPP4R1 MEG1 PP4R1
O43598	0.177940369	2.860098442	DNPH1 C6orf108 RCL
P98194	0.178229332	2.668050279	ATP2C1 KIAA1347 PMR1L HUSSY-28
Q01415	0.178233147	0.1722876	GALK2 GK2
Q5TFE4	0.178266525	2.196451206	NT5DC1 NT5C2L1 LP2642
P43686	0.178302765	2.017905272	PSMC4 MIP224 TBP7
Q8NHP8	0.178721428	1.904987983	PLBD2
Q9H0S4	0.178771496	0.743411917	DDX47
P45954	0.178941727	1.879503697	ACADSB
Q9UPU5	0.179017067	1.020500892	USP24 KIAA1057
O75503	0.179065704	2.127465054	CLN5
Q5T5P2	0.179068565	0.920764225	KIAA1217 SKT
Q99460	0.179245949	1.234057078	PSMD1
Q9NNX1	0.179389954	1.410650776	TUFT1
O00186	0.179507256	1.312987124	STXBP3
Q96KA5	0.179535866	2.266532944	CLPTM1L CRR9

Q9UL26	0.17986393	1.577154563	RAB22A RAB22
Q13439	0.180021286	1.344629481	GOLGA4
O60503	0.18010664	2.275418841	ADCY9 KIAA0520
Q9UHQ4	0.180152893	1.710024337	BCAP29 BAP29
Q9NXW9	0.180343628	0.854452568	ALKBH4 ABH4
Q9H000	0.180877686	2.241296145	MKRN2 RNF62 HSPC070
Q9Y217	0.180912971	2.561509144	MTMR6
O14618	0.181492805	1.37305757	CCS
Q969G6	0.181632996	2.13993167	RFK
Q9Y6R4	0.18256855	0.792902439	MAP3K4 KIAA0213 MAPKKK4 MEKK4 MTK1
O95786	0.183204651	0.942817044	DDX58
Q14168	0.183445454	0.809699779	MPP2 DLG2
P16298	0.183489799	2.000909286	PPP3CB CALNA2 CALNB CNA2
P49327	0.183663368	2.104343542	FASN FAS
Q5M775	0.183828831	1.993869899	SPECC1 CYTSB NSP5
Q9NRN7	0.183947563	1.325522438	AASDHPPT CGI-80 HAH-P HSPC223 x0005
Q8WU76	0.184112549	0.797120673	SCFD2 STXBP1L1
P46976	0.184686661	3.804518049	GYG1 GYG
Q12851	0.185060501	0.656244245	MAP4K2 GCK RAB8IP
Q6PIJ6	0.18512392	0.935738898	FBXO38 SP329
Q14C86	0.185474396	2.414717556	GAPVD1 GAPEX5 KIAA1521 RAP6
O75396	0.185482025	2.348243405	SEC22B SEC22L1
Q8IZL8	0.185571671	1.456371744	PELP1 HMX3 MNAR
Q15942	0.185611725	1.161377189	ZYX
Q9ULH0	0.185688019	1.127011589	KIDINS220 ARMS KIAA1250
Q9UIW2	0.185790062	0.963927037	PLXNA1 NOV PLXN1
O75110	0.185845852	0.859560934	ATP9A ATPIIA KIAA0611
O95671	0.186184883	2.181676902	ASMTL
P05165	0.186234474	2.016028668	PCCA
P00387	0.186290741	1.739814405	CYB5R3 DIA1
P60604	0.186421394	1.793358211	UBE2G2 UBC7
Q8IZP0	0.186974525	2.031882451	ABI1 SSH3BP1
Q86UL3	0.187211037	1.463402464	GPAT4 AGPAT6 TSARG7 UNQ551/PRO1108
Q9Y2V7	0.187252998	0.973847869	COG6 KIAA1134
P26885	0.187473297	2.333150094	FKBP2 FKBP13
O60476	0.187784195	1.263802399	MAN1A2 MAN1B
Q09328	0.187837601	2.324016159	MGAT5 GGNT5
Q0ZGT2	0.188481331	2.029520944	NEXN
Q70CQ2	0.188566208	0.305720639	USP34 KIAA0570 KIAA0729
Q9Y3P9	0.18857193	1.062733169	RABGAP1 HSPC094
Q9BTV4	0.188711166	2.124737031	TMEM43 UNQ2564/PRO6244
Q99426	0.188815117	2.05288206	TBCB CG22 CKAP1
Q9Y5Y2	0.188853264	1.625483175	NUBP2

Q9H061	0.188867569	2.568069465	TMEM126A
P11216	0.188908577	1.971103978	PYGB
Q8TEU7	0.18893671	1.322325292	RAPGEF6 PDZGEF2
Q9Y4E8	0.188946724	2.176307265	USP15 KIAA0529
Q8WUK0	0.189429283	1.024272373	PTPMT1 MOSP PLIP PNAS-129
P01034	0.189691544	0.691040672	CST3
Q96II8	0.189917564	1.855605328	LRCH3
Q9Y6M1	0.190480232	2.567919044	IGF2BP2 IMP2 VICKZ2
Q9P2B4	0.190582275	1.332380477	CTTNBP2NL KIAA1433
Q02809	0.190735817	2.010901746	PLOD1 LLH PLOD
P08174	0.190766335	1.679880966	CD55 CR DAF
Q9UK99	0.190814495	1.213548952	FBXO3 FBX3
P45985	0.191821098	2.008066649	MAP2K4 JNKK1 MEK4 MKK4 PRKMK4 SEK1 SERK1 SKK1
Q13233	0.191946983	1.224443236	MAP3K1 MAPKKK1 MEKK MEKK1
P51784	0.192129135	1.291068585	USP11 UHX1
Q13144	0.192308426	1.797056869	EIF2B5 EIF2BE
P02751	0.192453384	2.316312746	FN1 FN
Q96RN5	0.192543507	1.684671369	MED15 ARC105 CTG7A PCQAP TIG1 TNRC7
Q14240	0.192552567	1.646559577	EIF4A2 DDX2B EIF4F
O14531	0.192559242	1.694148515	DPYSL4 CRMP3 ULIP4
Q86YR5	0.193153381	1.021311062	GPSM1 AGS3
Q15165	0.193445206	0.936492995	PON2
P21964	0.193914413	2.117860403	COMT
O75794	0.194020271	0.718940219	CDC123 C10orf7 D123
Q2M389	0.194167137	2.167753685	WASHC4 KIAA1033
Q13636	0.194586754	1.324747907	RAB31 RAB22B
Q16850	0.194677353	1.648805674	CYP51A1 CYP51
Q96AY3	0.194802284	2.327849068	FKBP10 FKBP65 PSEC0056
P55899	0.194994926	1.314772151	FCGRT FCRN
Q16698	0.195001602	1.667635657	DECR1 DECR SDR18C1
Q6YHK3	0.195138454	1.891891319	CD109 CPAMD7
P29373	0.195225716	1.970742157	CRABP2
Q5VIR6	0.195281982	1.175488907	VPS53 PP13624
P51572	0.195562363	1.671789215	BCAP31 BAP31 DXS1357E
Q5VU97	0.195780754	0.872385468	CACHD1 KIAA1573 VWCD1
P25440	0.195982933	0.739481932	BRD2 KIAA9001 RING3
Q96PX9	0.19645977	1.378901674	PLEKHG4B KIAA1909
Q9H0B6	0.196492195	2.583323256	KLC2
O94829	0.196652412	1.014062889	IPO13 KIAA0724 RANBP13
P63010	0.196673393	2.595003556	AP2B1 ADTB2 CLAPB1
P07203	0.196980476	1.927328476	GPX1
Q9NXV2	0.197117805	1.25456079	KCTD5

Q15642	0.197177887	1.166340541	TRIP10 CIP4 STOT STP
Q8NBL1	0.19753933	1.750594691	POGLUT1 C3orf9 CLP46 KTELC1 MDSRP MDS010 UNQ490/PRO1006
Q969P0	0.197738647	1.028344481	IGSF8 CD81P3 EWI2 KCT4
Q08AD1	0.197768211	1.075491945	CAMSAP2 CAMSAP1L1 KIAA1078
Q68D91	0.197813511	1.407473565	MBLAC2
Q9Y4P8	0.198263168	1.393545727	WIPI2 CGI-50
P42338	0.198508263	0.843502234	PIK3CB PIK3C1
Q96QG7	0.198942184	3.940849429	MTMR9 C8orf9 MTMR8
Q14166	0.199199677	2.757060554	TTLL12 KIAA0153
O95810	0.199259758	1.575836549	CAVIN2 SDPR
Q9UN37	0.199296951	1.016369871	VPS4A VPS4
Q6P3W7	0.199368477	2.132478777	SCYL2 CVAK104 KIAA1360
Q7Z739	0.199703217	1.563033201	YTHDF3
Q9UFF9	0.199729919	1.447625792	CNOT8 CALIF POP2
Q9BQC3	0.19978857	0.658073233	DPH2 DPH2L2
Q15746	0.199877739	1.833492427	MYLK MLCK MLCK1 MYLK1
Q9Y3Q3	0.199947357	0.640031349	TMED3 C15orf22 UNQ5357/PRO1078
P36873	0.199998856	1.00152469	PPP1CC
Q6ZMI0	0.200148582	1.42643982	PPP1R21 CCDC128 KLRAQ1
Q06136	0.200348854	1.800679017	KDSR FVT1 SDR35C1
Q8IYB8	0.200393677	1.2297966	SUPV3L1 SUV3
Q6NUM9	0.200530052	1.17830329	RETSAT PPSIG UNQ439/PRO872
Q7KZI7	0.200536728	1.438072684	MARK2 EMK1
P20073	0.200660706	2.812107507	ANXA7 ANX7 SNX OK/SW-cl.95
P62195	0.200808525	1.977164971	PSMC5 SUG1
Q9UBV7	0.201043129	0.893160353	B4GALT7 XGALT1 UNQ748/PRO1478
P36543	0.201129913	1.218667445	ATP6V1E1 ATP6E ATP6E2
O60518	0.201216698	2.525885831	RANBP6
Q9GZP9	0.201682091	2.358227288	DERL2 DER2 FLANA CGI-101 SBBI53
Q9H9S5	0.20179224	0.857787429	FKRP
Q9P2T1	0.201858521	0.935733423	GMPR2
Q9Y426	0.202010632	0.953549	C2CD2 C21orf25 C21orf258 TMEM24L
Q9UPT9	0.202257156	1.235786259	USP22 KIAA1063 USP3L
Q15042	0.202548027	1.916925903	RAB3GAP1 KIAA0066 RAB3GAP
Q8NB37	0.202565193	1.210556332	GATD1 PDDC1
Q13509	0.202680588	1.032794712	TUBB3 TUBB4
P30519	0.202877045	1.484441169	HMOX2 HO2
Q86WN1	0.202885151	0.938932327	FCHSD1 UNQ737/PRO1431
Q6ZSR9	0.203186989	1.38522991	
Q9NVA2	0.203329086	2.139111975	SEPTIN11 SEPT11
Q12913	0.203474045	1.152162488	PTPRJ DEP1
P12694	0.203667641	1.91354687	BCKDHA
Q9Y3C0	0.204248428	0.996230744	WASHC3 AD-016 CCDC53 CGI-116 x0009

Q9NRW7	0.204270363	1.76469264	VPS45 VPS45A VPS45B
P61018	0.204359055	0.539350883	RAB4B PP1596
Q9ULJ7	0.204640388	1.735847917	ANKRD50 KIAA1223
Q8N465	0.204764366	3.049483628	D2HGDH D2HGD
Q96ME1	0.204776764	0.854235811	FBXL18 FBL18
Q92546	0.205024719	0.812277366	RGP1 KIAA0258
Q9NVS9	0.205112457	2.450805321	PNPO
O60664	0.206187248	3.611488303	PLIN3 M6PRBP1 TIP47
Q96DG6	0.206437111	2.02968093	CMBL
P78362	0.207109451	1.279498504	SRPK2
P08134	0.207296371	2.328897744	RHOC ARH9 ARHC
P41221	0.207438469	1.152571086	WNT5A
Q8IWE4	0.207539558	2.131823844	DCUN1D3 SCCRO3
Q13371	0.208057404	1.004662547	PDCL PHLOP1 PhLP1
Q9BW60	0.208245277	0.947624467	ELOVL1 SSC1 CGI-88
P50135	0.208747864	1.505683612	HNMT
Q9P291	0.208810806	1.241881423	ARMCX1 ALEX1 AD032
Q9UHN6	0.208931923	1.167164035	CEMIP2 KIAA1412 TMEM2
P35813	0.209124565	2.411993607	PPM1A PPPM1A
Q8IWI9	0.20914793	1.54872073	MGA KIAA0518 MAD5
P23634	0.209179878	2.650157256	ATP2B4 ATP2B2 MXRA1
Q9NZJ7	0.209330559	1.883877935	MTCH1 PSAP CGI-64 UNQ1871/PRO4314
Q4V328	0.209400177	1.286679742	GRIPAP1 KIAA1167
P19883	0.209444046	1.547527646	FST
Q8NEW0	0.209486961	1.373074268	SLC30A7 ZNT7 ZNTL2
P28300	0.209996223	1.78404357	LOX
P05023	0.210172653	2.21755761	ATP1A1
Q7L775	0.210588455	0.809966003	EPM2AIP1 KIAA0766 My007
Q9P270	0.210609436	1.417422829	SLAIN2 KIAA1458
Q4KMP7	0.210857391	1.289256937	TBC1D10B FP2461
Q9P0J1	0.210980415	1.438141719	PDP1 PDP PPM2C
Q7Z3U7	0.21138382	2.340032536	MON2 KIAA1040 SF21
Q96CM8	0.211750031	1.104376089	ACSF2 UNQ493/PRO1009
P07902	0.212610245	1.230651553	GALT
O00471	0.212647438	2.641922648	EXOC5 SEC10 SEC10L1
Q01433	0.212778091	2.311733733	AMPD2
P30048	0.212792397	1.507823977	PRDX3 AOP1
Q7Z6J0	0.213153362	0.36560609	SH3RF1 KIAA1494 POSH POSH1 RNF142 SH3MD2
Q9NR12	0.213291168	2.012090139	PDLIM7 ENIGMA
Q9UKU7	0.213359833	2.38012768	ACAD8 ARC42 IBD
Q9H7C4	0.213418961	2.533918214	SYNC SYNC1
Q3SXM5	0.213779449	1.465276604	HSDL1 SDR12C3
Q92696	0.213806152	2.601360656	RABGGTA

Q6ZS17	0.214191437	1.423160648	RIPOR1 FAM65A KIAA1930
Q8NBS9	0.214382172	2.298653811	TXNDC5 TLP46 UNQ364/PRO700
O60888	0.214513779	1.29854295	CUTA ACHAP C6orf82
Q9NS86	0.214960098	0.843536688	LANCL2 GPR69B TASP
Q02252	0.214961052	1.403424221	ALDH6A1 MMSDH
Q658Y4	0.21518755	1.318617849	FAM91A1
O43293	0.215512276	1.795491182	DAPK3 ZIPK
O95336	0.215723991	3.726014426	PGLS
Q93034	0.215871811	1.400393194	CUL5 VACM1
Q6YN16	0.216423035	2.532697766	HSDL2 C9orf99 SDR13C1
P27448	0.216582298	1.13621594	MARK3 CTAK1 EMK2
Q9Y2L1	0.217173576	2.025629173	DIS3 KIAA1008 RRP44
Q8IYI6	0.217478752	2.727233127	EXOC8
P29083	0.217579842	0.903028013	GTF2E1 TF2E1
Q9UIC8	0.217662811	1.909491592	LCMT1 LCMT CGI-68
Q12841	0.217903137	1.910370785	FSTL1 FRP
Q96CV9	0.217967033	2.595868914	OPTN FIP2 GLC1E HIP7 HYPL NRP
O14773	0.218379974	1.28319503	TPP1 CLN2 GIG1 UNQ267/PRO304
Q9BZL4	0.218575478	2.551602658	PPP1R12C LENG3 MBS85
Q86XZ4	0.218597412	1.431899189	SPATS2 SCR59 SPATA10 Nbla00526
O60313	0.218976021	2.450280419	OPA1 KIAA0567
P06396	0.219548225	1.551600419	GSN
Q9HCC0	0.219808578	2.303309867	MCCC2 MCCB
Q92575	0.219909668	2.303615712	UBXN4 KIAA0242 UBXD2 UBXDC1
Q96AJ9	0.219955444	1.302519172	VTI1A
Q0JRZ9	0.220396996	1.899881033	FCHO2
Q8NDV7	0.220425606	0.899756489	TNRC6A CAGH26 KIAA1460 TNRC6
O14936	0.220468521	0.970694643	CASK LIN2
Q8IWJ2	0.221331596	1.270862623	GCC2 KIAA0336 RANBP2L4
Q8WYL5	0.221516609	1.250535777	SSH1 KIAA1298 SSH1L
P09110	0.221597672	2.383139898	ACAA1 ACAA PTHIO
O95864	0.221761703	1.268152745	FADS2
O14545	0.222356796	1.147125377	TRAFD1 FLN29
Q96ER9	0.222417355	0.779926468	CCDC51 MITOK
P17813	0.222470284	2.140276719	ENG END
Q9NUQ2	0.223455906	1.794918214	AGPAT5
P04062	0.223753929	2.28630153	GBA GC GLUC
Q96AC1	0.224152565	2.927883273	FERMT2 KIND2 MIG2 PLEKHC1
P54764	0.224165916	2.078917946	EPHA4 HEK8 SEK TYRO1
Q9H4Z3	0.224246979	1.332710469	PCIF1 C20orf67 CAPAM PPP1R121
Q13131	0.224559784	1.846795362	PRKAA1 AMPK1
P04179	0.224667549	2.861795985	SOD2
Q6ZT21	0.224895477	1.11178059	TMPPE

Q86VS8	0.225122452	2.952243348	HOOK3
Q9UMS0	0.225369453	2.856074987	NFU1 HIRIP5 CGI-33
Q9BZE9	0.225448608	0.717056956	ASPSCR1 ASPL RCC17 TUG UBXD9 UBXN9
Q8WUP2	0.225588799	2.867882148	FBLIM1 FBLP1
O43933	0.225614548	0.882841364	PEX1
Q13614	0.226272583	0.979333866	MTMR2 KIAA1073
Q9UM47	0.226807594	1.992801275	NOTCH3
Q68DK7	0.226919174	0.530849382	MSL1 MSL1L1
Q15654	0.227005959	1.643675619	TRIP6 OIP1
O43520	0.227363586	1.10531667	ATP8B1 ATPIC FIC1 PFIC
Q16775	0.227915764	1.428386355	HAGH GLO2 HAGH1
Q9UK97	0.227982998	0.99646942	FBXO9 FBX9 VCIA1
P27824	0.228067398	2.035862294	CANX
Q9NQC7	0.228331089	1.536904314	CYLD CYLD1 KIAA0849 HSPC057
P37268	0.228912354	1.812699312	FDFT1
Q9H4L5	0.229151726	1.671900979	OSBPL3 KIAA0704 ORP3 OSBP3
Q96MX6	0.229166985	1.481570679	DNAAF10 WDR92
Q6PIU2	0.230237961	1.960343709	NCEH1 AADACL1 KIAA1363
Q9Y5T5	0.230344772	1.19620037	USP16 MSTP039
O96017	0.230354309	1.509924613	CHEK2 CDS1 CHK2 RAD53
Q13613	0.230441093	1.633129837	MTMR1
O95861	0.230555534	2.637294337	BPNT1
Q96GF1	0.230600357	1.624744126	RNF185
Q6PML9	0.230913162	0.735756536	SLC30A9 C4orf1 HUEL
Q9Y6Y8	0.231080055	2.964718265	SEC23IP MSTP053
Q6ZVM7	0.23121357	2.467568919	TOM1L2
Q5JPI3	0.231451511	0.981035657	C3orf38
Q13177	0.231499672	3.348997609	PAK2
O15091	0.231588364	1.31276438	PRORP KIAA0391 MRPP3
Q05682	0.2318964	2.859300449	CALD1 CAD CDM
Q9NZI7	0.232541084	0.38136989	UBP1 LBP1
P52294	0.232615471	1.826378056	KPNA1 RCH2
Q9NR46	0.232884407	1.257723334	SH3GLB2 KIAA1848 PP578
Q6GMV3	0.232892036	0.870852898	PTRHD1 C2orf79
Q9NWT6	0.233286858	1.64440799	HIF1AN FIH1
Q8WZA9	0.233638763	2.006060913	IRGQ IRGQ1 FKSG27
Q86X76	0.233834267	1.174714681	NIT1
Q9UHL4	0.234132767	1.278863628	DPP7 DPP2 QPP
P12931	0.234333038	1.075479799	SRC SRC1
P07947	0.234444618	2.855847254	YES1 YES
P57723	0.234712601	2.049799211	PCBP4
O95396	0.234770298	0.876259327	MOCS3 UBA4

Q8IWT6	0.234847069	1.54329933	LRRC8A KIAA1437 LRRC8 SWELL1 UNQ221/PRO247
P11279	0.234946251	2.565627921	LAMP1
Q8NHP6	0.234985352	1.809629929	MOSPD2
Q8TC07	0.234998703	2.324483321	TBC1D15
Q9Y3S2	0.235125542	0.756496327	ZNF330 NOA36
Q96RP9	0.235255241	2.310343283	GFM1 EFG EFG1 GFM
Q96FQ6	0.235448837	1.31874744	S100A16 S100F AAG13
Q9P2E5	0.235741138	1.338664654	CHPF2 CHSY3 CSGLCAT KIAA1402 UNQ299/PRO339
Q9P260	0.235777855	1.75187234	RELCH KIAA1468
Q9NXG6	0.236035347	1.573032212	P4HTM PH4
O75153	0.236241341	1.698982584	CLUH KIAA0664
Q14315	0.236535072	3.635499214	FLNC ABPL FLN2
Q08J23	0.236554146	2.161791323	NSUN2 SAKI TRM4
O15357	0.23686409	1.739599893	INPPL1 SHIP2
Q9NUY8	0.237007141	2.397825183	TBC1D23 NS4ATP1
P51571	0.237109184	1.502514601	SSR4 TRAPD
Q96BN8	0.237307549	2.445679876	OTULIN FAM105B
Q969H8	0.23739624	2.996643304	MYDGF C19orf10
Q9HC98	0.238547325	1.954161259	NEK6
O43741	0.238801956	1.289396442	PRKAB2
Q9BXP2	0.239355087	1.606026646	SLC12A9 CCC6 CIP1
P05106	0.239733696	2.332380358	ITGB3 GP3A
P09496	0.240712166	2.302470309	CLTA
P60033	0.24105072	1.94973408	CD81 TAPA1 TSPAN28
Q86U38	0.241684437	0.625032526	NOP9 C14orf21 KIAA2021
P53007	0.241704941	2.932971289	SLC25A1 SLC20A3
Q8TC12	0.241804123	2.560531019	RDH11 ARSDR1 PSDR1 SDR7C1 CGI-82
Q9HA65	0.241846085	1.225082828	TBC1D17
Q9UQ13	0.241911888	1.661962932	SHOC2 KIAA0862
P49593	0.24245739	1.598092262	PPM1F KIAA0015 POPX2
Q5T6V5	0.242923737	0.802854239	C9orf64
O94953	0.243155479	1.116624692	KDM4B JHDM3B JMJD2B KIAA0876
Q96PC5	0.243257523	1.681187096	MIA2 CTAGE5 MEA11 MEA6 MGEA11 MGEA6
Q9UKV5	0.244033337	1.424493361	AMFR RNF45
P61204	0.244076252	1.350462456	ARF3
O14662	0.244707584	2.240047368	STX16
Q99685	0.244794846	1.67193825	MGLL
Q9BVA1	0.244894028	1.181912202	TUBB2B
Q86W92	0.2450037	1.444964131	PPFIBP1 KIAA1230
Q9H6F5	0.245066643	1.420124413	CCDC86 CYCLON
Q8IYB1	0.245485306	2.096553731	MB21D2 C3orf59

Q9H9B1	0.245967388	0.724391422	EHMT1 EUHMTASE1 GLP KIAA1876 KMT1D
P62820	0.246585846	1.370184586	RAB1A RAB1
Q9Y394	0.247069359	3.56338125	DHRS7 DHRS7A RETSDR4 SDR34C1 CGI-86 UNQ285/PRO3448
O75886	0.247488022	1.431138925	STAM2 HBP
P35610	0.247988701	1.014677477	SOAT1 ACACT ACACT1 ACAT ACAT1 SOAT STAT
Q16352	0.248700142	1.906079618	INA NEF5
Q9UNL2	0.24878788	2.016038783	SSR3 TRAPG
Q9H2K8	0.249034882	3.569030048	TAOK3 DPK JIK KDS MAP3K18
Q15785	0.25053215	1.346291635	TOMM34 URCC3
Q9H0A8	0.250650883	1.598544883	COMMD4
Q96NT0	0.250987053	1.227904384	CCDC115
Q9Y6M7	0.251243591	1.442420426	SLC4A7 BT NBC2 NBC2B NBC3 NBCn1 SBC2 SLC4A6
Q9Y6K8	0.251808167	1.390527332	AK5
Q9H1E5	0.251983643	1.545529109	TMX4 KIAA1162 TXNDC13 PSEC0095 UNQ475/PRO938
P48556	0.252030373	2.169240426	PSMD8
Q9NVV4	0.252187729	0.615918191	MTPAP PAPD1
P12107	0.252744198	1.363902994	COL11A1 COLL6
Q5TC12	0.252752304	0.363407766	ATPAF1 ATP11
Q96QR8	0.252828598	1.444468015	PURB
Q8IZ21	0.253377914	1.322641905	PHACTR4 PRO2963
Q9NXU5	0.253671646	1.333338405	ARL15 ARFRP2
Q96QB1	0.253744125	0.890025564	DLC1 ARHGAP7 KIAA1723 STARD12
O43242	0.255161285	1.759638443	PSMD3
Q7LBR1	0.255588531	1.1979488	CHMP1B C18orf2
Q99856	0.255822182	1.788093931	ARID3A DRIL1 DRIL3 DRX E2FBP1
P16591	0.256501198	1.78862057	FER TYK3
O95782	0.256797791	3.003769019	AP2A1 ADTAA CLAPA1
P32929	0.257043839	1.297897683	CTH
Q5W111	0.257236481	1.558480205	SPRYD7 C13orf1 CLLD6
P17020	0.25752449	1.681219538	ZNF16 HZF1 KOX9
P51948	0.258427143	0.821857148	MNAT1 CAP35 MAT1 RNF66
Q8NEB9	0.258496284	2.417013038	PIK3C3 VPS34
Q7Z7E8	0.25869751	1.09893399	UBE2Q1 NICE5 UBE2Q PRO3094
P07738	0.25888443	3.829856888	BPGM
Q9C035	0.259013176	3.462938971	TRIM5 RNF88
Q9NVG8	0.259142876	3.444576303	TBC1D13
Q8WV41	0.259642601	1.361319352	SNX33 SH3PX3 SH3PXD3C SNX30
Q9H6U8	0.259830475	0.71654124	ALG9 DIBD1
Q96G46	0.259841919	1.068234203	DUS3L
Q9HD20	0.259952545	2.416531443	ATP13A1 ATP13A KIAA1825 CGI-152
P30154	0.261199951	1.77146832	PPP2R1B

Q12789	0.261327744	1.724861618	GTF3C1
P22681	0.26170826	1.013575954	CBL CBL2 RNF55
Q13685	0.262432098	1.848372931	AAMP
Q5JTD0	0.262444496	1.748013495	TJAP1 PILT TJP4
P54709	0.262587547	1.44485234	ATP1B3
Q6DKJ4	0.263044357	2.795044408	NXN NRX
Q6NUK4	0.263148308	1.045362428	REEP3 C10orf74
O94967	0.263299942	0.967956193	WDR47 KIAA0893
P15104	0.263547897	1.248622676	GLUL GLNS
P46926	0.263900757	2.295269143	GNPDA1 GNPI HLN KIAA0060
O15228	0.264509201	2.287362808	GNPAT DAPAT DHAPAT
Q6P996	0.265455246	2.37597329	PDXDC1 KIAA0251
Q9ULT8	0.26584816	2.463488947	HECTD1 KIAA1131
Q8N8R3	0.265885353	0.25872649	SLC25A29 C14orf69 ORNT3
P21953	0.266159058	1.486055373	BCKDHB
O75170	0.266179085	1.626851325	PPP6R2 KIAA0685 PP6R2 SAPS2
Q66LE6	0.266475677	1.19788363	PPP2R2D KIAA1541
Q709C8	0.266602516	3.079556491	VPS13C KIAA1421
Q04446	0.266851425	1.72557601	GBE1
Q9UP95	0.266922951	0.97136	SLC12A4 KCC1
O95825	0.267449379	1.421828025	CRYZL1 4P11
Q9NXF1	0.267915726	0.996758651	TEX10 L18 Nbla10363
Q9Y4K0	0.267924309	2.87927841	LOXL2
Q12893	0.268185616	2.728345204	TMEM115 PL6 LUCA11.2
Q9H1H9	0.268578053	0.82585839	KIF13A RBKIN
Q96S97	0.268694878	0.326280043	MYADM UNQ553/PRO1110
Q99700	0.269697189	2.208767201	ATXN2 ATX2 SCA2 TNRC13
Q8IY17	0.269827843	1.952736352	PNPLA6 NTE
Q99805	0.26991272	1.903907708	TM9SF2
Q9Y2R4	0.270136833	1.192677511	DDX52 ROK1 HUSSY-19
O43294	0.271035194	1.621017254	TGFB1I1 ARA55
Q03001	0.271038055	2.768655022	DST BP230 BP240 BPAG1 DMH DT KIAA0728
Q96EB6	0.271585464	2.338714184	SIRT1 SIR2L1
Q10471	0.272368431	1.658129506	GALNT2
P48454	0.272773266	2.374841953	PPP3CC CALNA3 CNA3
Q9UMX5	0.272974014	2.961274922	NENF CIR2 SPUF
Q9Y2J4	0.273532867	1.795194623	AMOTL2 KIAA0989
Q9NTJ5	0.273789406	1.432111827	SACM1L KIAA0851 SAC1
Q9UBU8	0.274299622	3.334405054	MORF4L1 MRG15 FWP006 HSPC008 HSPC061 PP368
Q6UW63	0.274652481	1.785464004	POGLUT2 EP58 KDELC1 UNQ1910/PRO4357
P35052	0.274860382	2.692973911	GPC1

Q9BUT1	0.275557518	1.73225521	BDH2 DHRS6 SDR15C1 UNQ6308/PRO20933
Q9H7P9	0.276159286	0.767522952	PLEKHG2
Q9NZQ3	0.276256561	1.791464268	NCKIPSD AF3P21 SPIN90
Q8N8S7	0.276643753	2.176246789	ENAH MENA
P05067	0.27716732	1.455782865	APP A4 AD1
Q96S55	0.278047562	2.039710014	WRNIP1 WHIP
Q16595	0.27820015	1.141837122	FXN FRDA X25
Q15276	0.278496742	2.052972544	RABEP1 RAB5EP RABPT5 RABPT5A
Q96EK7	0.278529167	1.928587459	FAM120B CCPG KIAA1838
O95714	0.279020309	0.615129098	HERC2
Q9UHY7	0.27939415	2.332180945	ENOPH1 MASA MSTP145
Q7L8L6	0.279547691	3.749215306	FASTKD5 KIAA1792
P20936	0.27977562	2.177975138	RASA1 GAP RASA
P35790	0.280004501	2.006540675	CHKA CHK CKI
Q16222	0.280088425	2.574568124	UAP1 SPAG2
Q13217	0.280285835	4.013623594	DNAJC3 P58IPK PRKRI
Q6ZUT6	0.2806077	0.515415727	CCDC9B C15orf52
Q9NRW3	0.280877113	1.11530331	APOBEC3C APOBEC1L PBI
Q9BU02	0.281811714	2.201253056	THTPA
P53990	0.282479286	1.982481405	IST1 KIAA0174
Q9UKM7	0.282557964	0.957313937	MAN1B1 UNQ747/PRO1477
P21281	0.283078194	3.168261526	ATP6V1B2 ATP6B2 VPP3
Q8NBN7	0.283182144	2.155065356	RDH13 SDR7C3 PSEC0082 UNQ736/PRO1430
P05452	0.284026146	0.833890196	CLEC3B TNA
Q96LJ7	0.284679413	1.573347733	DHRS1 SDR19C1
Q9NZN5	0.285131454	1.571510807	ARHGEF12 KIAA0382 LARG
O15269	0.285478592	2.187571555	SPTLC1 LCB1
P09525	0.285636902	1.497392006	ANXA4 ANX4
Q56VL3	0.285719872	1.1152571	OCIAD2
Q13951	0.287427902	4.036884466	CBFB
Q8N9R8	0.287546635	1.635863499	SCAI C9orf126
O75382	0.288421631	1.496078819	TRIM3 BERP RNF22 RNF97
O94760	0.288598061	2.559448622	DDAH1 DDAH
Q8N1F8	0.288624763	0.591079878	STK11IP KIAA1898 LIP1 LKB1IP STK11IP1
Q96EK9	0.288723946	1.129988886	KTI12 SBBI81
Q8N4L2	0.289021492	1.299189017	PIP4P2 TMEM55A
Q8N5A5	0.28907156	0.964593389	ZGPAT GPATC6 GPATCH6 KIAA1847 ZC3H9 ZC3HDC9 ZIP
Q68CQ4	0.290438175	0.188266137	UTP25 C1orf107 DEF DIEXF
Q9UQ16	0.290630341	1.001265252	DNM3 KIAA0820
Q96CN9	0.291714668	1.125491497	GCC1
O14867	0.291940689	1.241571937	BACH1

Q08257	0.29204464	2.965026944	CRYZ
Q8IV38	0.292262077	1.278067164	ANKMY2
Q7Z3B1	0.292536736	1.434920457	NEGR1 IGLON4 UNQ2433/PRO4993
P05787	0.293613911	1.681480648	KRT8 CYK8
P16070	0.294906616	1.143580959	CD44 LHR MDU2 MDU3 MIC4
Q10472	0.294946671	1.815605428	GALNT1
Q8IX04	0.295135498	1.551336443	UEVLD UEV3
Q9Y6I9	0.295667648	2.850062877	TEX264 ZSIG11 UNQ337/PRO536
Q99504	0.29864645	1.764380089	EYA3
P50479	0.299797058	2.42244904	PDLIM4 RIL
Q13049	0.300373077	0.639714082	TRIM32 HT2A
Q7RTP6	0.300840378	1.295004998	MICAL3 KIAA0819 KIAA1364
P32856	0.300880909	1.857402655	STX2 EPIM STX2A STX2B STX2C
O60784	0.301419258	1.258249725	TOM1
Q8N3F8	0.301555634	1.303036722	MICALL1 KIAA1668 MIRAB13
Q8N511	0.302610397	1.501253908	TMEM199 C17orf32
Q2M2I8	0.303469658	2.881431381	AAK1 KIAA1048
P31150	0.304646492	2.675155897	GDI1 GDIL OPHN2 RABGDIA XAP4
Q8NFW8	0.304779053	2.204685672	CMAS
Q8IZ81	0.306406975	1.973990379	ELMOD2
Q07352	0.307082176	1.47759345	ZFP36L1 BERG36 BRF1 ERF1 RNF162B TIS11B
P06241	0.307739258	1.632124453	FYN
O43854	0.307743073	2.463612613	EDIL3 DEL1
P11274	0.307952881	1.394476808	BCR BCR1 D22S11
Q16513	0.309002876	2.073379491	PKN2 PRK2 PRKCL2
Q9P2K5	0.309605598	1.11443513	MYEF2 KIAA1341
Q16706	0.309706688	0.882794291	MAN2A1 MANA2
Q7L099	0.310004234	1.709701379	RUFY3 KIAA0871
P13807	0.310173988	1.703817053	GYS1 GYS
P02792	0.310675621	2.262020828	FTL
Q6PCB7	0.311266899	2.125396259	SLC27A1 ACSVL5 FATP1
P58107	0.311585426	1.808155037	EPPK1 EPIPL
P24386	0.311940193	1.044167075	CHM REP1 TCD
Q5T7W0	0.311964989	1.52038307	ZNF618 KIAA1952
Q12974	0.312535763	1.20446719	PTP4A2 PRL2 PTPCAAX2 BM-008
Q9H939	0.31273365	2.158761542	PSTPIP2
Q86SK9	0.31310606	1.438820625	SCD5 ACOD4 SCD2 SCD4
Q9NP79	0.313598633	1.940988058	VTA1 C6orf55 HSPC228 My012
Q13796	0.315807343	0.578508244	SHROOM2 APXL
Q99536	0.316975594	2.355829128	VAT1
Q9UHB6	0.317150116	3.669516213	LIMA1 EPLIN SREBP3 PP624
Q9HBM0	0.318646431	2.055370387	VEZT
Q9H9H4	0.318861961	2.370419066	VPS37B

Q16512	0.320874214	1.358570381	PKN1 PAK1 PKN PRK1 PRKCL1
P17050	0.321419716	2.044673329	NAGA
Q07960	0.321471214	2.407425948	ARHGAP1 CDC42GAP RHOGAP1
Q9UMZ2	0.321530342	1.169197856	SYNRG AP1GBP1 SYNG
P55795	0.322346687	1.554462496	HNRNPH2 FTP3 HNRPH2
Q14517	0.322431564	3.110536724	FAT1 CDHF7 FAT
P13861	0.322536469	2.869429772	PRKAR2A PKR2 PRKAR2
Q8IWB7	0.322985649	1.236967575	WDFY1 FENS1 KIAA1435 WDF1 ZFYVE17
Q8IWV7	0.323083878	2.538148919	UBR1
P50281	0.323897362	2.380535519	MMP14
Q8NCE2	0.323955059	1.597599807	MTMR14 C3orf29
Q15063	0.324006081	2.092371142	POSTN OSF2
Q9H7S9	0.324191093	0.528193696	ZNF703 ZEPPO1 ZPO1
P36507	0.324381828	3.655259582	MAP2K2 MEK2 MKK2 PRKMK2
Q8NBK3	0.324625969	3.276512803	SUMF1 PSEC0152 UNQ3037/PRO9852
Q9BRP4	0.326272011	1.808860779	PAAF1 WDR71
Q9NU19	0.326387405	1.09015144	TBC1D22B C6orf197
Q13637	0.326662064	2.133106452	RAB32
Q9Y2H0	0.32755661	2.377890999	DLGAP4 DAP4 KIAA0964 SAPAP4
Q9Y4F5	0.327798843	1.634786431	CEP170B FAM68C KIAA0284
P42858	0.328255653	1.26231865	HTT HD IT15
Q07812	0.328796387	2.218288267	BAX BCL2L4
Q9UPN7	0.329373837	1.957513452	PPP6R1 KIAA1115 PP6R1 SAPS1
Q9UMX1	0.330397606	2.686798597	SUFU UNQ650/PRO1280
Q9BUF5	0.330868721	2.279212011	TUBB6
Q96G23	0.332865715	1.696405736	CERS2 LASS2 TMSG1
Q00765	0.332874298	2.2791828	REEP5 C5orf18 DP1 TB2
Q8N3Y1	0.333946228	0.698981593	FBXW8 FBW6 FBW8 FBX29 FBXO29 FBXW6
Q9BYX2	0.334799767	1.38665668	TBC1D2 PARIS1 PP8997 TBC1D2A
Q15836	0.335180283	0.999702556	VAMP3 SYB3
Q5UCC4	0.335494041	1.94939376	EMC10 C19orf63 INM02 UNQ764/PRO1556
Q9Y508	0.336174965	3.065601312	RNF114 ZNF228 ZNF313
P43121	0.336705208	2.365622744	MCAM MUC18
Q9UDY4	0.337346077	3.520094018	DNAJB4 DNAJW HLJ1
Q8WUJ3	0.337715149	2.572782313	CEMIP KIAA1199
Q9H019	0.337875366	0.77984697	MTFR1L FAM54B HYST1888 MSTP116
Q92734	0.338018417	2.17927931	TFG
P61224	0.338815689	1.646691843	RAP1B OK/SW-cl.11
Q15434	0.339106083	0.696781299	RBMS2 SCR3
Q9UGI8	0.339788437	2.151706713	TES
Q9UI12	0.340019226	2.699898392	ATP6V1H CGI-11
Q495W5	0.340476036	1.865637349	FUT11
P26440	0.343216896	1.440134764	IVD

Q13449	0.343238831	1.117743489	LSAMP IGLON3 LAMP
Q8WUI4	0.343370438	1.898474401	HDAC7 HDAC7A
P11047	0.343494415	3.239276192	LAMC1 LAMB2
Q9Y294	0.343764782	0.682387037	ASF1A CGI-98 HSPC146
Q93074	0.344423294	1.247081178	MED12 ARC240 CAGH45 HOPA KIAA0192 TNRC11 TRAP230
Q6P9B6	0.345856667	2.318580847	MEAK7 KIAA1609 TLDC1
Q96RQ3	0.346367836	1.520165528	MCCC1 MCCA
Q13948	0.347244263	2.439982342	CUX1 CUTL1
Q4KMQ2	0.347681999	1.924904893	ANO6 TMEM16F
Q7Z4R8	0.348324776	1.797576504	C6orf120
P25445	0.349282265	2.920325017	FAS APT1 FAS1 TNFRSF6
Q8NCC3	0.349918365	1.868158507	PLA2G15 LYPLA3 UNQ341/PRO540
Q6PJG6	0.350906372	1.111367134	BRAT1 BAAT1 C7orf27
Q9NZM1	0.352513313	2.506511803	MYOF FER1L3 KIAA1207
Q5EBL4	0.353176594	1.478545284	RILPL1 RLP1
Q9Y605	0.353362083	1.5418569	MRFAP1 PAM14 PGR1
Q96T83	0.353958607	2.026956481	SLC9A7 NHE7
Q9UBM7	0.355384827	1.70843671	DHCR7 D7SR
P48651	0.355813026	1.46888557	PTDSS1 KIAA0024 PSSA
P82932	0.355949879	0.506539055	MRPS6 C21orf101 RPMS6
Q15691	0.356471062	2.800351053	MAPRE1
P02462	0.356946945	1.585243696	COL4A1
P51911	0.357121468	1.664090277	CNN1
Q86X02	0.358026981	2.428017759	CDR2L HUMPPA
Q96QD8	0.358503342	2.085474414	SLC38A2 ATA2 KIAA1382 SAT2 SNAT2
P55290	0.359090805	1.964233565	CDH13 CDHH
Q7Z3G6	0.360975742	0.840346737	PRICKLE2
Q9Y4P1	0.361297607	2.385406646	ATG4B APG4B AUTL1 KIAA0943
Q16798	0.361647129	1.325442969	ME3
Q13885	0.361813545	2.502656304	TUBB2A TUBB2
P54252	0.362114906	2.786173704	ATXN3 ATX3 MJD MJD1 SCA3
Q08AE8	0.362321854	3.499013374	SPIRE1 KIAA1135 SPIR1
Q9Y2G8	0.362776756	2.475843676	DNAJC16 KIAA0962
Q9UPM8	0.362878323	1.147187659	AP4E1
Q9Y2H6	0.366394043	1.693349411	FNDC3A FNDC3 HUGO KIAA0970
Q9NR28	0.367416382	1.523953915	DIABLO SMAC
O15013	0.367508888	2.058636704	ARHGEF10 KIAA0294
Q8N6H7	0.368735313	2.180763187	ARFGAP2 ZNF289 Nbla10535
Q5NDL2	0.369120598	2.221463469	EOGT AER61 C3orf64 EOGT1
Q15041	0.36914444	1.859419507	ARL6IP1 ARL6IP ARMER KIAA0069
Q9UIJ7	0.369594574	2.525432868	AK3 AK3L1 AK6 AKL3L
Q6P6C2	0.370970726	0.861080432	ALKBH5 ABH5 OFOXD1
Q9BVI4	0.371921539	0.726954317	NOC4L

Q96L58	0.372005939	0.728932068	B3GALT6
P10646	0.372438431	2.093541973	TFPI LACI TFPI1
Q86U90	0.372959137	1.277068858	YRDC DRIP3 IRIP
Q6GYQ0	0.373073578	1.268031003	RALGAPA1 GARNL1 KIAA0884 TULIP1
P61421	0.373113632	2.077967175	ATP6V0D1 ATP6D VPATPD
Q8N5I9	0.375750542	2.281998308	NOPCHAP1 C12orf45
P53609	0.376247883	0.993599666	PGGT1B
P05413	0.377093315	2.22608421	FABP3 FABP11 MDGI
Q8WUH1	0.378836632	1.711276952	CHURC1 C14orf52 CHCH My015
O15121	0.379025459	2.097837636	DEGS1 DES1 MLD MIG15
O95758	0.379217148	1.302879127	PTBP3 ROD1
P38606	0.380449295	2.649291317	ATP6V1A ATP6A1 ATP6V1A1 VPP2
O75348	0.380898476	3.661532818	ATP6V1G1 ATP6G ATP6G1 ATP6J
P05161	0.383351326	2.662030654	ISG15 G1P2 UCRP
P99999	0.385243416	3.078760141	CYCS CYC
P40763	0.385453224	2.767123517	STAT3 APRF
O60488	0.385546684	2.731305426	ACSL4 ACS4 FACL4 LACS4
P48060	0.385595322	4.389502328	GLIPR1 GLIPR RTVP1
P23528	0.386582375	1.952630223	CFL1 CFL
Q6R327	0.389755726	2.219192212	RICTOR KIAA1999
O60443	0.389761925	2.638289109	GSDME DFNA5 ICERE1
Q14194	0.390304565	3.331778983	CRMP1 DPYSL1 ULIP3
Q8N2K0	0.390910149	2.73116132	ABHD12 C20orf22
P17081	0.393248558	1.07015114	RHOQ ARHQ RASL7A TC10
O75367	0.393878937	3.156794691	MACROH2A1 H2AFY
Q9Y664	0.395650864	1.882614398	KPTN
Q99797	0.396131516	2.148855913	MIPEP MIP
Q9C0H2	0.402293205	2.840821565	TTYH3 KIAA1691
P35240	0.402376175	2.981659455	NF2 SCH
P0CG39	0.402939796	2.086052374	POTEJ
P11717	0.405602455	2.541313777	IGF2R MPRI
Q8NEZ5	0.406580925	1.703815499	FBXO22 FBX22
Q9UPY8	0.406889915	3.06614335	MAPRE3
P15924	0.408429146	2.016899411	DSP
Q9Y3R5	0.408520222	0.884901022	DOP1B C21orf5 DOPEY2 KIAA0933
Q9NZD2	0.411138535	1.620857603	GLTP
Q96FS4	0.412682533	0.999148956	SIPA1 SPA1
Q00610	0.413873672	3.323446343	CLTC CLH17 CLTCL2 KIAA0034
P49257	0.414266586	3.331291953	LMAN1 ERGIC53 F5F8D
Q9NRX4	0.415996552	2.362993625	PHPT1 PHP14 CGI-202 HSPC141
O00221	0.416180134	1.502498583	NFKBIE IKBE
P32456	0.416247368	2.014962717	GBP2
Q9UHW9	0.416790009	3.862064562	SLC12A6 KCC3

Q01581	0.418470383	2.003486533	HMGCS1 HMGCS
P15586	0.418727875	3.18461306	GNS
Q96I15	0.420982838	2.021000458	SCLY SCL
Q9UII2	0.423874378	0.879117291	ATP5IF1 ATPI ATPIF1
Q4L180	0.426509857	2.504720932	FILIP1L COL4A3BPIP DOC1 GIP90
Q8IVM0	0.427118301	1.557667608	CCDC50 C3orf6
P15529	0.428370476	3.09926467	CD46 MCP MIC10
Q8WXC6	0.429533005	0.826555335	COPS9 MYEOV2
P36551	0.42983532	3.217946642	CPOX CPO CPX
Q6UX15	0.431861877	2.27193252	LAYN UNQ208/PRO234
Q9UEU0	0.431930542	1.719496159	VTI1B VTI1 VTI1L VTI1L1 VTI2
P23743	0.433045387	2.228146688	DGKA DAGK DAGK1
Q9BXI6	0.433787346	1.507824698	TBC1D10A EPI64 TBC1D10
Q66PJ3	0.4349823	0.376669312	ARL6IP4
Q9H6S3	0.435682297	2.19687024	EPS8L2 EPS8R2 PP13181
Q99961	0.436306	2.229020212	SH3GL1 CNSA1 SH3D2B
Q9UKI2	0.436682701	3.255735448	CDC42EP3 BORG2 CEP3
O15460	0.438581467	3.944408581	P4HA2 UNQ290/PRO330
O00560	0.438673973	2.5221506	SDCBP MDA9 SYCL
P48637	0.440360069	2.752779553	GSS
O15085	0.441429138	0.883084058	ARHGEF11 KIAA0380
Q96K49	0.442915916	2.133346466	TMEM87B
Q86YV5	0.44337368	2.150181782	PRAG1 SGK223
O95980	0.4433918	3.074684633	RECK ST15
Q9NVZ3	0.443619728	3.099096127	NECAP2
Q86V87	0.444342613	1.701189541	FHIP2B FAM160B2 RAI16 FP13191
O60565	0.445688248	2.877613374	GREM1 CKTSF1B1 DAND2 DRM PIG2
Q99595	0.447628975	1.804093187	TIMM17A MIMT17 TIM17 TIM17A TIMM17
P78356	0.448935509	1.779261171	PIP4K2B PIP5K2B
P84101	0.449851036	2.519006041	SERF2 FAM2C
Q00534	0.450716019	3.335894112	CDK6 CDKN6
P04181	0.452062607	2.064430948	OAT
P21291	0.45281601	2.539360047	CSRP1 CSRP CYRP
Q8N556	0.453255653	3.236741473	AFAP1 AFAP
B2RTY4	0.457158089	1.059150712	MYO9A MYR7
Q9NQ92	0.457162857	0.2883005	COPRS C17orf79 COPR5
P58004	0.457891464	2.209758075	SESN2 Hi95 SEST2
O14763	0.458345413	2.128176473	TNFRSF10B DR5 KILLER TRAILR2 TRICK2 ZTNFR9 UNQ160/PRO186
Q9P2J3	0.458759785	0.764130754	KLHL9 KIAA1354
O14727	0.459291458	2.035578722	APAF1 KIAA0413
Q13219	0.464003563	1.313123329	PAPPA
Q8N6M0	0.468738556	0.55947028	OTUD6B DUBA5 CGI-77
O75962	0.470426559	2.661035574	TRIO

O95372	0.472448349	2.588249827	LYPLA2 APT2
Q14CN4	0.473219872	1.511779848	KRT72 K6IRS2 KB35 KRT6 KRT6IRS2
Q6ZT12	0.478568554	1.492215731	UBR3 KIAA2024 ZNF650
Q9BT23	0.478667259	2.333265242	LIMD2 SB143
O94929	0.488790512	2.932472713	ABLIM3 KIAA0843 HMFN1661
O95757	0.490322113	2.462391926	HSPA4L APG1 OSP94
Q9NV96	0.49151516	1.819181999	TMEM30A C6orf67 CDC50A
Q9Y5K6	0.494235039	2.219537305	CD2AP
O15270	0.496396065	1.983948704	SPTLC2 KIAA0526 LCB2
O94916	0.497974396	2.20111683	NFAT5 KIAA0827 TONEBP
Q9Y673	0.502048492	1.547811765	ALG5 HSPC149
O76024	0.507332802	2.466125939	WFS1
P56730	0.51394558	2.883203938	PRSS12
Q8N5C1	0.514136314	1.906549692	CALHM5 C6orf188 FAM26E
P50502	0.514390945	2.591618113	ST13 AAG2 FAM10A1 HIP SNC6
O00635	0.514691353	0.87084124	TRIM38 RNF15 RORET
Q9NVH0	0.51544714	1.012039141	EXD2 C14orf114 EXDL2
Q8WX93	0.516162872	3.415414798	PALLD KIAA0992 CGI-151
Q8TBM8	0.51998806	2.12596026	DNAJB14 UNQ9427/PRO34683
Q7Z417	0.520668983	3.191089539	NUFIP2 KIAA1321 PIG1
Q9Y2K6	0.522326469	1.120951399	USP20 KIAA1003 LSFR3A VDU2
Q9NUJ3	0.539466858	2.684136602	TCP11L1
O75146	0.546984673	2.995672437	HIP1R HIP12 KIAA0655
Q6DD88	0.547951698	2.491528392	ATL3
P22570	0.552438736	2.016586651	FDXR ADXR
P08172	0.553114891	1.84313113	CHRM2
O95685	0.558028221	1.78144236	PPP1R3D PPP1R6
P42771	0.558980465	1.683707425	CDKN2A CDKN2 MTS1
Q96IZ0	0.559772491	2.028629315	PAWR PAR4
P29317	0.560633659	3.208414166	EPHA2 ECK
Q9NQ88	0.564351082	3.021545003	TIGAR C12orf5
Q96F24	0.574288368	3.086808082	NRBF2 COPR
P53814	0.575078964	2.294184527	SMTN SMSMO
Q8NF37	0.575182915	1.892588769	LPCAT1 AYTL2 PFAAP3
Q6PJI9	0.584370613	0.915544482	WDR59 KIAA1923 FP977
P15151	0.589907646	3.30606339	PVR PVS
Q93050	0.598067284	1.592352878	ATP6V0A1 ATP6N1 ATP6N1A VPP1
P24593	0.599580765	3.247742581	IGFBP5 IBP5
Q15014	0.607756615	1.856291456	MORF4L2 KIAA0026 MRGX
Q8IWZ3	0.611660004	2.136326729	ANKHD1 KIAA1085 MASK VBARP PP2500
Q9Y5J5	0.620266914	2.486329669	PHLDA3 TIH1
P01111	0.620295525	2.446863548	NRAS HRAS1
O94851	0.625668526	3.650582754	MICAL2 KIAA0750 MICAL2PV1 MICAL2PV2 MICALCL

P13645	0.627813339	1.668127207	KRT10 KPP
O00748	0.6287117	2.62091117	CES2 ICE
Q9UM54	0.632953644	2.591680943	MYO6 KIAA0389
Q96PE3	0.642374516	1.029497262	INPP4A
O75915	0.643289566	3.16315557	ARL6IP5 DERP11 JWA PRA2 PRAF3 HSPC127
O94905	0.64330864	2.622778852	ERLIN2 C8orf2 SPFH2 UNQ2441/PRO5003/PRO9924
Q6IA69	0.672712326	2.960387252	NADSYN1
O43493	0.681466103	2.957987437	TGOLN2 TGN46 TGN51
Q96HC4	0.683624268	3.19454425	PDLIM5 ENH L9
Q9NZV1	0.691151619	3.267535573	CRIM1 S52 UNQ1886/PRO4330
P27105	0.694794655	3.438740401	STOM BND7 EPB72
P51452	0.698898315	2.431028811	DUSP3 VHR
Q13526	0.712094307	2.056268337	PIN1
Q9NZR2	0.739098072	1.160294367	LRP1B LRPDIT
Q99442	0.744425774	3.009868364	SEC62 TLOC1
Q15437	0.767608643	3.055167173	SEC23B
Q5W0V3	0.774477959	3.169076805	FHIP2A FAM160B1 KIAA1600
P98082	0.785861969	3.152379901	DAB2 DOC2
Q04771	0.805949211	2.383909111	ACVR1 ACVRLK2
P35527	0.806170464	1.593073286	KRT9
P53365	0.832258224	1.740973722	ARFIP2 POR1
P31431	0.833063126	2.299611819	SDC4
Q96NE9	0.853417397	2.274023613	FRMD6 C14orf31
P04264	0.858105659	1.519843227	KRT1 KRTA
Q9UI42	0.860266685	2.088050837	CPA4 CPA3 UNQ694/PRO1339
P18085	0.863610268	3.713187994	ARF4 ARF2
Q96PD2	0.87255764	2.806144027	DCBLD2 CLCP1 ESDN
P05121	0.905243874	2.644165245	SERPINE1 PAI1 PLANH1
P49023	0.912911415	4.409686905	PXN
P11169	0.998412609	3.74899899	SLC2A3 GLUT3
Q9UBN6	1.006098747	2.407171916	TNFRSF10D DCR2 TRAILR4 TRUNDD UNQ251/PRO288
Q99988	1.044356346	4.15954683	GDF15 MIC1 PDF PLAB PTGFB
P07996	1.070644379	2.567342072	THBS1 TSP TSP1
Q5T2D3	1.268454075	1.342469243	OTUD3 KIAA0459
P38936	1.420875549	4.220505896	CDKN1A CAP20 CDKN1 CIP1 MDA6 PIC1 SDI1 WAF1
P62987	1.893033028	3.126474389	UBA52 UBCEP2
