# Supplementary material for: FRMD6 determines the cell fate towards senescence: involvement of the Hippo-YAP-CCN3 axis
Source: Cell Death Differ. 2024 Jun 26;31(11):1398–409. doi: 10.1038/s41418-024-01333-2 (PMC11519602; doi:10.1038/s41418-024-01333-2)
Supplement: Supplementary file 3 — Table S2 [file 41418_2024_1333_MOESM3_ESM.pdf]

Table S2. List of differentially expressed genes in GFP and GFP-FRMD6 expressed cells.

Symbol	EXP:GFP-1	EXP:GFP-2	EXP:GFP-FRMD6-1	EXP:GFP-FRMD6-2	DEG: LOG2FC (GFP-FRMD6 vs	P-value	Q-value
AC113189.4	0.00	0.00	0.11	0.13	5.12	0.000256	0.001584
PWAR5	0.00	0.00	0.19	0.11	4.56	0.002016	0.010258
DMD	2.32	2.62	3.22	2.54	0.69	0.000109	0.000727
LRR1	7.57	6.42	1.58	1.96	-1.40	0.000041	0.000288
PRPF6	28.43	29.10	15.95	18.46	-0.40	0.000213	0.001337
FHL1	18.07	19.28	18.55	20.05	0.56	0.000017	0.000129
KCNMB1	0.03	0.00	0.12	0.11	4.83	0.007982	0.034630
ANOS1	0.16	0.02	1.32	1.39	4.51	0.000000	0.000000
WNT4	0.00	0.00	0.07	0.22	4.39	0.003442	0.016545
SMIM34A	0.03	0.00	0.39	0.43	4.21	0.005710	0.025832
CCND2-AS1	0.04	0.11	0.69	0.80	3.59	0.000173	0.001103
PDZD2	0.01	0.12	0.10	0.51	3.31	0.009717	0.041159
STRA6	0.37	0.12	1.48	1.87	3.30	0.000000	0.000002
FUT1	0.02	0.07	0.60	0.34	3.29	0.000109	0.000723
HSD17B6	1.38	0.93	10.41	9.21	3.28	0.000000	0.000000
GUCY1A1	0.14	0.07	0.58	0.42	3.16	0.000449	0.002659
LINC01914	0.56	0.55	2.04	1.79	2.12	0.002155	0.010903
AC011450.1	1.90	0.87	4.94	5.25	2.03	0.001130	0.006099
JAG2	0.72	0.37	1.10	0.52	1.81	0.000556	0.003220
TMC7	0.30	0.09	0.50	0.44	1.79	0.005952	0.026768
EFCAB13	0.79	0.63	1.52	1.76	1.74	0.000544	0.003155
SESN3	0.44	0.27	0.75	0.75	1.61	0.000000	0.000001
PTPDC1	2.32	1.96	5.51	5.57	1.61	0.000000	0.000000
AC008537.3	0.51	0.31	0.82	1.13	1.59	0.011142	0.046323
AL356056.2	0.82	0.39	0.74	1.17	1.48	0.004954	0.022751
HSF4	4.38	5.73	8.36	10.95	1.35	0.000044	0.000310
TP53INP2	5.40	5.60	8.63	8.93	0.99	0.000000	0.000000
CU633904.2	4.14	4.14	3.11	5.97	0.88	0.007544	0.032935
RBM48	1.90	1.33	2.57	2.12	0.86	0.000615	0.003532
PHGDH	128.47	145.12	169.77	180.12	0.74	0.000000	0.000000
HERC2P2	49.78	43.93	57.38	56.70	0.73	0.000000	0.000000
NFKBIL1	5.46	4.75	5.81	6.81	0.69	0.007775	0.033844
ZNF91	3.91	3.90	4.98	5.30	0.58	0.002327	0.011666
SLC29A4	6.30	8.59	8.60	8.33	0.57	0.000932	0.005133
ZBTB34	1.54	1.23	1.74	1.52	0.57	0.010909	0.045491
ZNF75A	17.19	14.41	18.33	16.32	0.54	0.000391	0.002340
ANO6	19.25	18.89	22.90	20.18	0.51	0.000000	0.000000
COMT	124.76	122.00	131.72	153.12	0.50	0.000000	0.000000
PEX13	3.33	4.11	3.85	3.38	0.49	0.007027	0.030924

ARGLU1	45.54	42.38	49.36	45.54	0.48	0.000000	0.000000
LRSAM1	6.62	6.87	7.97	7.31	0.48	0.002796	0.013751
MFSD4B	3.73	2.57	3.30	4.25	0.47	0.004816	0.022207
ATP2C1	41.33	43.06	48.95	41.78	0.46	0.000000	0.000000
ERAP2	6.10	5.41	6.12	5.62	0.40	0.007254	0.031781
ARSK	7.20	6.59	7.49	7.00	0.39	0.009185	0.039118
TMEM44	15.29	17.34	17.47	16.59	0.39	0.009283	0.039502
PHC3	7.46	4.17	5.52	6.67	0.35	0.003293	0.015930
APPBP2	9.21	8.50	7.06	7.61	0.33	0.005204	0.023755
CLPTM1	104.09	102.64	93.90	103.32	0.31	0.000035	0.000252
EFCAB14	11.88	12.52	13.44	11.80	0.31	0.002775	0.013654
MAFG	9.66	10.07	9.61	9.92	0.30	0.007443	0.032525
TEP1	12.29	12.42	13.43	13.16	0.30	0.011096	0.046147
COPG1	127.98	128.80	93.00	88.05	-0.23	0.002155	0.010903
FXR2	29.83	31.34	20.71	19.22	-0.31	0.003894	0.018444
CCAR2	70.62	62.99	44.33	43.38	-0.31	0.000338	0.002046
ZMYM4	10.82	12.08	6.87	7.98	-0.32	0.002035	0.010351
YAP1	32.42	31.38	20.78	21.77	-0.33	0.000140	0.000913
UNC45A	36.10	41.19	23.21	25.52	-0.41	0.000080	0.000541
TOP1	43.44	42.63	27.32	24.13	-0.41	0.000001	0.000008
TRABD	16.07	15.22	8.58	10.36	-0.44	0.002884	0.014146
CDC42EP4	9.00	8.32	4.71	4.66	-0.44	0.006660	0.029538
NUS1	9.30	8.71	5.68	4.73	-0.46	0.000562	0.003250
RTEL1-TNFRS	7.03	7.69	3.59	4.92	-0.47	0.002983	0.014573
SPOP	10.62	10.68	7.86	5.35	-0.47	0.006762	0.029911
PDLIM1	91.70	89.87	52.14	53.63	-0.49	0.000000	0.000000
ASH2L	11.07	10.45	6.88	5.61	-0.50	0.001292	0.006892
STK4	5.18	4.47	3.64	2.58	-0.51	0.001506	0.007902
CBX3	47.55	48.16	30.01	24.26	-0.55	0.000002	0.000016
NOL9	4.86	3.74	3.00	2.71	-0.57	0.001291	0.006890
PNPLA2	25.91	33.43	16.49	17.79	-0.60	0.000003	0.000025
ZC3H18	21.40	22.29	11.73	12.25	-0.64	0.000000	0.000000
RBM18	4.48	3.34	1.40	1.79	-0.68	0.006516	0.029022
SIRT1	5.01	5.38	2.90	2.42	-0.68	0.000552	0.003201
AGO1	4.87	4.63	2.29	1.85	-0.68	0.000000	0.000001
HAT1	21.67	18.70	10.40	9.41	-0.69	0.000013	0.000100
BRD9	21.01	22.58	10.42	10.19	-0.72	0.000000	0.000000
TRIM16	15.53	15.13	8.16	6.49	-0.79	0.000000	0.000001
GPN3	6.32	5.63	2.53	2.32	-0.81	0.011104	0.046173
WDCP	3.05	2.82	1.44	1.32	-0.85	0.000731	0.004123
GSTCD	1.90	2.03	1.69	1.30	-0.85	0.006689	0.029634
FAM136A	11.37	11.08	4.57	4.89	-0.87	0.000005	0.000043
RBBP9	2.96	3.30	1.06	1.14	-0.87	0.002379	0.011905

XRCC5	102.77	100.60	52.00	42.30	-0.87	0.000000	0.000000
BUD13	6.17	7.08	2.82	2.84	-0.88	0.000131	0.000858
NEDD1	7.10	6.00	2.79	2.15	-0.91	0.000010	0.000081
GLE1	8.27	8.19	3.50	3.37	-0.91	0.000000	0.000000
NTHL1	8.62	8.80	3.63	3.70	-0.97	0.002675	0.013226
HMGXB4	8.58	7.99	3.20	3.31	-0.97	0.000000	0.000000
NDUFA2	9.69	10.26	4.38	3.80	-0.98	0.004206	0.019753
G3BP1	55.58	51.52	21.02	16.95	-0.98	0.000000	0.000000
DTNBP1	4.35	3.49	1.49	2.20	-1.02	0.009482	0.040299
NUP93	42.56	42.89	16.65	17.06	-1.02	0.000000	0.000000
EMB	2.06	1.53	0.84	0.52	-1.07	0.004661	0.021589
BRI3BP	2.89	2.68	1.19	0.99	-1.09	0.000005	0.000041
FANCE	2.53	2.64	1.05	0.84	-1.12	0.000966	0.005302
MRM3	3.86	4.31	1.73	1.49	-1.12	0.001021	0.005578
LINC00346	0.70	0.68	0.26	0.24	-1.14	0.004652	0.021561
TRAF2	7.35	7.48	2.05	2.89	-1.23	0.000000	0.000002
RAET1G	4.11	4.58	1.25	1.52	-1.30	0.004847	0.022308
RPL39L	8.57	9.00	2.87	2.65	-1.32	0.001086	0.005893
ACSS1	1.25	1.13	0.38	0.40	-1.34	0.001738	0.008986
MASTL	5.76	5.05	1.75	1.49	-1.38	0.000000	0.000000
AC145207.5	1.05	0.93	0.34	0.20	-1.53	0.007171	0.031490
AGFG2	4.49	6.34	0.85	1.71	-1.57	0.000002	0.000019
SLC19A1	17.69	16.20	5.06	4.61	-1.60	0.000000	0.000000
DUT	30.69	26.35	8.49	5.60	-1.76	0.000000	0.000000
STRIP2	1.26	1.74	0.30	0.36	-1.91	0.000002	0.000021
PTPN3	0.49	0.38	0.07	0.09	-1.91	0.000475	0.002795
HMGN2P5	34.44	32.17	4.45	8.42	-1.95	0.004453	0.020752
ARHGAP11B	2.06	1.51	0.19	0.47	-2.13	0.000814	0.004542
MLKL	1.40	1.49	0.28	0.30	-2.16	0.000066	0.000456
GINS4	10.78	10.74	1.93	0.80	-2.43	0.000000	0.000000
MT1L	5.74	3.97	0.00	0.00	-4.97	0.000234	0.001455
COA1	15.16	15.48	9.50	7.45	-0.50	0.004700	0.021752
AL139220.2	2.02	1.47	0.76	0.16	-2.37	0.003307	0.015985
MYDGF	138.60	135.49	150.39	143.32	0.45	0.000000	0.000000
FAM107B	16.51	14.96	8.64	6.54	-0.72	0.000006	0.000052
CEP128	1.94	1.96	0.28	0.26	-2.31	0.000000	0.000000
ZNF823	1.59	1.43	0.95	0.44	-1.30	0.008680	0.037238
C19orf48	33.04	35.56	14.29	16.45	-0.83	0.000000	0.000000
KCNK6	2.82	2.83	2.95	2.93	0.54	0.001936	0.009910
DIPK2A	4.98	4.91	8.16	7.10	0.96	0.000000	0.000000
ZNF180	2.45	1.98	1.10	1.05	-0.73	0.011987	0.049433
PSME3IP1	38.73	37.56	23.52	23.09	-0.36	0.004556	0.021168
CCDC93	7.02	7.86	5.29	5.17	-0.39	0.006236	0.027894

PTGES3L-AAR	1.19	0.98	3.46	3.44	2.80	0.000000	0.000001
U2SURP	41.57	35.54	28.61	26.03	-0.32	0.000200	0.001259
KLHL28	1.55	1.37	2.18	2.40	0.90	0.000073	0.000499
SIK1B	0.00	0.00	0.04	0.26	5.21	0.000327	0.001985
AC048341.1	0.87	0.81	1.27	1.14	0.86	0.000129	0.000847
STARD13	11.89	10.78	5.42	5.37	-0.74	0.000000	0.000000
ALDH3B1	6.59	7.20	2.41	2.49	-1.09	0.000000	0.000001
ELMOD1	0.30	0.38	0.96	0.79	2.03	0.001621	0.008447
TBC1D12	1.30	1.27	1.65	1.48	0.62	0.011058	0.046013
AC068631.3	24.71	18.99	32.64	27.56	0.83	0.001335	0.007099
PIMREG	15.07	14.85	0.42	0.36	-4.89	0.000000	0.000000
EEF1AKMT2	5.51	6.35	2.57	2.70	-0.91	0.002633	0.013049
FHDC1	0.04	0.09	0.80	0.80	3.90	0.000000	0.000000
HMGB3	20.95	22.45	8.71	7.63	-0.86	0.000001	0.000010
RPRD1A	11.25	10.16	7.00	5.85	-0.57	0.000356	0.002146
RNF166	11.03	11.65	7.17	6.61	-0.62	0.001463	0.007702
AC093157.1	5.28	6.08	7.19	5.47	0.64	0.000901	0.004973
ATR	9.17	9.84	7.33	8.00	-0.32	0.006521	0.029039
LMLN	2.14	1.87	3.73	3.01	0.86	0.000001	0.000005
KIDINS220	24.01	21.05	28.09	27.90	0.61	0.000000	0.000000
ALKBH1	3.76	4.23	1.88	1.50	-0.73	0.011665	0.048228
TAC3	0.49	0.61	2.39	2.55	2.68	0.000471	0.002777
PCDHB9	0.22	0.18	0.43	1.11	2.72	0.000052	0.000359
CEP78	6.26	5.60	2.70	2.60	-0.91	0.000002	0.000013
AC068473.3	0.07	0.05	0.37	0.27	2.83	0.001318	0.007015
NEFH	2.06	2.00	0.76	0.61	-1.23	0.000097	0.000650
MRM2	9.32	8.95	3.78	4.08	-0.84	0.000132	0.000860
ST3GAL5	8.56	8.47	10.12	9.76	0.50	0.001533	0.008031
TMEM175	24.24	22.44	26.17	26.27	0.53	0.000078	0.000532
XRN2	34.16	33.10	17.88	16.64	-0.63	0.000000	0.000000
FAM71F2	1.27	1.04	3.25	4.19	1.72	0.000000	0.000000
HPCAL1	9.21	10.82	3.42	3.58	-1.10	0.000000	0.000004
SDHD	17.61	17.15	9.53	7.34	-0.72	0.000169	0.001080
BCL9	5.10	6.67	7.34	5.44	0.38	0.004508	0.020984
ZDHHC17	12.22	13.93	19.15	14.53	0.48	0.000043	0.000300
ADAMTSL3	0.15	0.00	0.12	0.28	4.01	0.006509	0.029005
GCDH	10.55	8.55	4.48	4.88	-0.58	0.008742	0.037475
AL161891.1	0.28	0.23	0.02	0.02	-3.29	0.002206	0.011130
H4C5	0.19	0.16	1.14	0.78	2.79	0.000815	0.004544
CPPED1	1.96	1.89	0.95	0.90	-0.80	0.009866	0.041675
AL445649.1	0.60	1.01	2.77	2.48	2.06	0.005688	0.025745
MRPL45	11.48	11.31	6.34	5.88	-0.56	0.005076	0.023240
KHDRBS1	53.11	53.21	28.94	23.96	-0.73	0.000000	0.000000

DDX55	13.64	12.58	6.33	8.25	-0.50	0.003471	0.016658
HPF1	10.62	12.02	4.92	4.69	-1.24	0.000009	0.000069
GDNF-AS1	1.02	0.88	1.01	1.01	1.68	0.000338	0.002045
RAB43	4.22	4.93	4.15	5.31	0.57	0.008775	0.037602
PAPSS1	21.26	20.36	14.06	12.72	-0.33	0.006719	0.029746
TTF1	3.43	3.41	2.29	2.28	-0.65	0.011751	0.048557
SLC44A3	0.32	0.31	0.81	0.86	2.75	0.000126	0.000828
TTC9	0.01	0.02	0.14	0.33	4.29	0.000142	0.000922
ROCK2	10.67	9.31	7.07	5.24	-0.41	0.000114	0.000758
WFS1	28.12	30.71	39.51	46.04	0.82	0.000000	0.000000
LNPK	9.00	7.27	14.35	11.79	0.78	0.000000	0.000000
ADI1	18.60	19.04	9.27	7.31	-0.73	0.000001	0.000005
ZNF605	6.78	6.03	8.27	7.43	0.66	0.000000	0.000003
ZFHX2	0.19	0.22	0.48	0.95	1.76	0.000004	0.000033
DDIAS	2.95	2.47	0.45	0.13	-3.83	0.000000	0.000000
ENDOV	9.11	10.12	12.38	13.80	0.74	0.000282	0.001734
SLC4A2	126.42	121.92	106.24	107.70	0.31	0.000035	0.000248
SMARCD2	23.66	21.87	13.55	14.52	-0.45	0.000431	0.002557
LINC00707	0.28	0.33	0.02	0.02	-3.74	0.008215	0.035516
AC083843.4	0.19	0.11	1.52	1.37	3.62	0.000000	0.000000
MBP	1.09	0.86	0.04	0.15	-2.97	0.000009	0.000071
ZNF708	2.37	1.93	3.10	2.41	0.78	0.003774	0.017918
ARHGAP24	2.84	2.54	3.92	3.85	0.95	0.000024	0.000174
NTN1	0.15	0.04	1.43	1.63	4.30	0.000000	0.000000
LRP1B	0.02	0.00	0.10	0.13	3.03	0.001356	0.007200
TNIK	5.38	5.61	8.52	10.52	1.26	0.000000	0.000000
CSTF1	17.43	18.55	9.69	11.30	-0.58	0.000175	0.001114
TEX2	13.38	12.82	12.79	12.01	0.28	0.004976	0.022849
RIOK3	9.74	9.33	11.69	10.63	0.56	0.000013	0.000098
SLC25A10	6.40	6.45	0.89	1.76	-2.02	0.000000	0.000000
CNTNAP3P2	0.16	0.18	0.66	0.58	2.26	0.000068	0.000464
LINC00607	0.11	0.24	1.06	0.74	2.46	0.000200	0.001260
FASTKD2	11.90	12.67	8.28	7.05	-0.37	0.007054	0.031028
CHD9	7.95	7.78	7.88	8.17	0.34	0.001236	0.006627
TMEM268	2.85	2.64	4.41	3.55	1.06	0.000000	0.000001
NUP188	30.41	33.08	13.08	14.56	-0.88	0.000000	0.000000
DSG2	6.02	5.57	7.36	6.55	0.60	0.000001	0.000012
ZFPM2	5.46	5.64	3.28	3.00	-0.50	0.002125	0.010764
PLOD3	176.04	186.36	179.86	208.55	0.47	0.000000	0.000000
RAB9B	0.25	0.22	0.53	0.52	1.49	0.008021	0.034754
RPA1	26.56	31.26	11.26	12.54	-1.09	0.000000	0.000000
ISLR2	0.00	0.00	0.28	0.47	5.17	0.000211	0.001327
ACOT1	3.05	3.13	3.96	4.42	0.76	0.007983	0.034630

AL136169.1	1.12	1.11	2.14	1.63	1.09	0.004088	0.019272
APOD	0.00	0.09	0.84	0.88	4.85	0.005025	0.023048
SUV39H2	2.84	3.67	1.08	0.91	-1.20	0.001158	0.006239
FAM72B	1.76	1.88	0.35	0.91	-1.78	0.000481	0.002828
AC022506.2	0.49	0.54	0.92	1.25	1.42	0.009095	0.038765
CTCF	10.75	10.63	6.64	5.62	-0.48	0.000449	0.002659
GLUL	34.60	36.82	18.50	20.50	-0.58	0.000000	0.000001
FAM160A2	12.06	14.61	12.80	13.19	0.38	0.006923	0.030543
MEIS3	17.11	19.91	21.64	23.59	0.58	0.000019	0.000145
PAPPA2	0.35	0.23	1.56	1.46	3.75	0.000000	0.000000
PIR	7.91	8.03	4.59	2.87	-0.88	0.001889	0.009699
C2orf69	2.36	3.07	1.18	1.07	-0.91	0.000932	0.005132
UBE2O	14.73	15.62	9.66	10.83	-0.28	0.007224	0.031687
USP32P1	12.59	9.50	12.77	13.32	0.58	0.000579	0.003346
AC068946.2	1.45	1.73	1.70	2.23	0.65	0.006998	0.030814
DHX36	23.67	24.60	15.63	13.91	-0.30	0.008350	0.036054
LRFN4	12.40	14.87	5.79	4.94	-1.07	0.000000	0.000000
SAMD9L	1.02	0.94	1.40	1.66	1.43	0.000000	0.000000
PLEKHG1	0.01	0.00	0.11	0.27	4.17	0.003837	0.018188
TSPAN33	0.03	0.04	0.62	0.36	3.23	0.004507	0.020984
NLGN1	2.06	1.21	1.98	2.29	0.94	0.000245	0.001519
PKNOX2	3.67	4.47	5.57	6.05	0.80	0.000007	0.000056
ANKS1A	5.67	5.33	6.11	6.87	0.67	0.000000	0.000001
CCDC180	0.37	0.51	1.39	1.26	1.31	0.006512	0.029010
SEPTIN6	8.80	9.23	11.54	10.55	0.65	0.000007	0.000059
PCDH1	0.22	0.15	2.14	1.89	3.78	0.000000	0.000000
USP4	25.61	22.30	16.92	16.14	-0.28	0.007090	0.031172
ANAPC7	42.89	50.34	25.28	31.39	-0.45	0.000058	0.000402
PLIN2	16.08	15.84	9.11	7.49	-0.60	0.000228	0.001426
HNRNPU	167.59	161.62	83.55	74.54	-0.63	0.000000	0.000000
TCHP	8.12	8.62	3.87	4.67	-0.80	0.000025	0.000184
DHX29	11.50	11.19	7.62	6.45	-0.31	0.011575	0.047899
CHEK1	7.64	8.47	3.96	3.63	-1.04	0.000000	0.000005
CPNE3	10.90	8.96	4.89	4.45	-0.94	0.000000	0.000000
NAE1	14.23	13.95	9.14	5.79	-0.92	0.000001	0.000013
CD96	0.27	0.04	0.77	0.60	2.14	0.005768	0.026037
GOLGA2P10	14.05	15.10	16.48	16.47	0.50	0.008563	0.036822
MMP15	0.94	1.17	2.59	2.89	1.71	0.000000	0.000000
TMEM222	18.84	19.18	9.20	10.06	-0.46	0.002411	0.012043
DOK6	1.70	0.42	1.01	2.84	1.39	0.000013	0.000101
MXRA7	74.35	75.87	98.90	84.24	0.54	0.000000	0.000000
LPAR2	7.57	7.11	12.27	12.15	1.05	0.000003	0.000028
SLC22A31	4.59	4.90	1.66	2.22	-1.17	0.001562	0.008172

SNUPN	8.57	10.33	5.20	4.98	-0.68	0.004581	0.021266
NELFCD	31.47	30.07	15.32	15.89	-0.64	0.000000	0.000002
GINS2	5.09	4.33	0.17	0.31	-4.38	0.000000	0.000000
PTGES3L	0.12	0.43	1.34	0.72	2.25	0.006946	0.030622
AC018638.6	0.40	0.20	1.07	0.98	2.11	0.009424	0.040073
ELP1	12.27	11.97	8.27	7.51	-0.30	0.004422	0.020644
FGD5-AS1	11.63	11.67	7.55	6.35	-0.37	0.006747	0.029857
SARDH	2.57	2.67	1.10	1.23	-0.80	0.006077	0.027237
CDKN2C	8.80	8.66	1.30	1.69	-2.41	0.000000	0.000000
NKD1	0.00	0.00	0.02	0.01	4.01	0.010452	0.043838
ATAD2B	2.28	2.55	3.10	2.52	0.72	0.000058	0.000400
CTHRC1	82.60	73.98	88.90	70.35	0.37	0.000765	0.004292
COLGALT1	95.43	103.58	108.25	102.35	0.34	0.000001	0.000005
COX4I1	174.37	191.43	118.52	116.96	-0.26	0.003643	0.017379
MED24	42.41	40.40	25.37	24.35	-0.34	0.002448	0.012210
PMPCA	40.93	37.67	23.13	23.80	-0.48	0.000014	0.000105
MRPL2	18.94	15.71	10.60	8.26	-0.73	0.001203	0.006464
SFXN5	7.84	6.46	8.35	7.87	0.53	0.000275	0.001693
IMPG2	0.03	0.03	0.11	0.21	2.92	0.000460	0.002713
MROH1	6.69	8.34	4.34	4.78	-0.41	0.010897	0.045450
HNRNPH2	33.93	30.98	22.00	18.73	-0.34	0.002354	0.011794
IGSF10	0.26	0.15	0.41	0.36	1.72	0.000201	0.001263
NNAT	8.26	8.80	17.69	17.37	1.39	0.000000	0.000000
TCF4	13.23	11.07	15.89	15.67	0.81	0.000000	0.000000
ROBO3	28.10	29.60	43.00	53.29	1.05	0.000000	0.000000
RFNG	33.47	34.84	32.83	37.59	0.38	0.000918	0.005060
SCAF8	12.02	10.64	7.48	7.06	-0.30	0.008343	0.036035
JRK	4.69	5.27	2.38	2.59	-0.50	0.000429	0.002543
ZNF503	11.79	11.71	5.27	5.44	-0.80	0.000000	0.000000
IPO11	6.03	7.83	2.45	3.19	-0.89	0.000005	0.000044
ITPR3	3.08	3.31	0.73	0.99	-1.56	0.000000	0.000000
MIR99AHG	0.39	0.50	0.90	1.35	2.48	0.000136	0.000886
AC008969.1	2.53	2.23	4.62	4.05	1.24	0.000000	0.000000
RAB4B	9.75	10.76	12.02	11.92	0.66	0.001430	0.007541
DMTF1	43.25	45.55	52.59	51.27	0.57	0.000000	0.000000
TBC1D9B	53.57	55.15	49.25	54.86	0.20	0.010448	0.043838
PRPF40A	36.67	35.58	25.38	23.54	-0.25	0.006647	0.029486
IDH3B	52.10	46.48	32.12	36.98	-0.31	0.012086	0.049744
PEX19	14.75	15.10	8.15	7.07	-0.48	0.000715	0.004043
PFKM	35.09	36.00	21.09	20.67	-0.52	0.000000	0.000003
MFAP1	17.67	19.19	8.94	8.77	-0.73	0.000001	0.000005
ZNHIT3	9.59	9.13	3.90	5.43	-0.78	0.009004	0.038473
LY6K	2.13	1.59	0.02	0.04	-4.83	0.000002	0.000015

TEX30	4.02	4.06	1.14	0.97	-1.57	0.000182	0.001153
SLC30A1	7.97	7.69	8.67	7.31	0.36	0.001685	0.008740
AKR1B10	1.40	1.04	0.00	0.23	-3.70	0.000362	0.002174
KDM4C	5.14	4.84	6.68	5.41	0.52	0.001350	0.007172
AC107871.1	2.19	4.50	0.46	0.54	-1.88	0.000000	0.000004
ADGRG1	5.24	4.64	1.57	1.52	-1.95	0.000000	0.000000
CCND2	0.19	0.34	3.26	2.87	4.32	0.000000	0.000000
AL358472.7	0.00	0.00	0.18	0.36	4.89	0.000652	0.003714
EXOSC9	25.32	22.95	8.61	8.71	-0.99	0.000000	0.000000
KCNC3	0.02	0.02	0.26	0.29	4.08	0.000319	0.001940
TMSB15A	2.85	4.60	0.67	0.33	-2.55	0.002446	0.012203
ARMCX3	26.77	25.08	26.68	23.19	0.29	0.006965	0.030682
UQCC1	26.08	21.77	14.53	14.44	-0.36	0.005393	0.024548
TSLP	0.65	0.45	0.98	1.24	1.34	0.006663	0.029543
TSPAN14	21.80	22.15	22.97	25.24	0.52	0.000000	0.000001
IRAK2	2.17	1.63	0.61	0.58	-1.33	0.000173	0.001104
GINS1	3.06	2.30	0.39	0.14	-3.41	0.000000	0.000000
GALE	15.63	22.51	7.67	7.33	-1.07	0.000000	0.000000
ALG11	12.53	10.54	12.06	11.08	0.29	0.007879	0.034251
ANKRD26	3.10	2.65	1.80	1.45	-0.51	0.007135	0.031361
MPV17	39.52	37.22	24.60	20.70	-0.58	0.000406	0.002420
FLRT3	0.90	0.91	1.35	1.08	0.79	0.009535	0.040502
PARP8	2.17	2.07	3.82	3.65	1.32	0.000000	0.000000
HLCS	5.01	4.67	6.38	6.00	0.53	0.000452	0.002672
NOMO2	217.67	216.01	240.46	244.19	0.49	0.000000	0.000000
AGPAT1	37.52	39.48	39.62	40.56	0.38	0.000046	0.000321
HDGFL2	41.73	43.82	23.31	25.86	-0.48	0.000002	0.000019
ABHD11	5.35	4.09	1.83	1.31	-1.62	0.000258	0.001593
FAM72C	1.19	0.82	0.21	0.24	-1.81	0.004949	0.022733
SFSWAP	22.09	22.60	21.08	21.12	0.27	0.004215	0.019790
ENTPD5	3.18	3.18	4.44	3.96	0.62	0.000173	0.001103
MXI1	2.07	1.67	3.72	4.54	1.34	0.000143	0.000930
TMCO6	6.10	7.66	2.63	3.76	-0.92	0.003588	0.017160
PCDHGB3	2.59	2.87	4.36	3.93	0.94	0.000000	0.000001
ZDHHC9	21.54	20.00	34.17	32.64	0.99	0.000000	0.000000
VRK1	7.64	7.91	1.05	0.59	-3.02	0.000000	0.000000
CTDSP2	47.55	47.22	31.78	31.95	-0.31	0.000158	0.001018
ATP1A2	0.51	0.30	1.61	1.39	2.45	0.000000	0.000000
GRHL1	0.36	0.03	0.63	0.34	2.42	0.009768	0.041344
SERPINB6	39.41	41.15	38.94	37.23	0.32	0.001633	0.008505
CIP2A	8.26	7.24	1.30	0.86	-2.47	0.000000	0.000000
SLC4A11	1.19	1.05	1.78	1.63	1.08	0.002597	0.012886
AC068234.1	0.00	0.00	1.23	1.29	7.45	0.000000	0.000000

EDRF1	11.62	13.32	7.56	7.25	-0.41	0.005587	0.025330
SLC17A5	8.12	7.33	11.53	10.55	0.85	0.000000	0.000000
PCDHB11	0.06	0.05	0.26	0.28	2.62	0.002256	0.011346
DCAF5	14.82	14.62	19.07	20.30	0.65	0.000000	0.000000
TAB3	2.94	3.41	3.31	3.33	0.48	0.003121	0.015182
AC106886.6	7.58	7.37	3.82	4.57	-0.50	0.000009	0.000069
RTEL1	22.46	20.05	10.08	11.66	-0.59	0.000007	0.000054
MIS18A	3.93	3.69	0.53	0.47	-2.59	0.000000	0.000000
AL031777.2	0.06	0.08	1.61	1.29	4.88	0.000002	0.000015
TSPAN11	0.14	0.05	1.29	1.01	3.71	0.000000	0.000000
DACH1	0.02	0.04	0.33	0.44	4.40	0.000003	0.000024
BIRC5	14.34	13.76	0.35	0.88	-4.72	0.000000	0.000000
IQCC	1.64	1.39	0.47	0.53	-1.29	0.010866	0.045344
DGUOK	23.39	25.29	11.57	9.39	-0.93	0.000010	0.000079
BAG6	90.99	101.24	66.97	73.87	-0.26	0.003113	0.015148
DKK1	27.57	26.13	5.61	4.56	-2.19	0.000000	0.000000
OSER1	9.93	10.79	5.74	6.23	-0.50	0.011222	0.046623
C8orf33	7.86	7.32	5.61	4.46	-0.48	0.010587	0.044326
H3-2	3.81	5.00	6.49	6.09	1.04	0.000000	0.000001
ZBED6	3.63	2.57	4.06	3.68	0.66	0.000004	0.000037
SAFB	46.66	47.75	30.96	27.92	-0.43	0.000002	0.000014
ATP6V1G2-DD	0.93	2.04	2.71	3.02	1.34	0.000357	0.002148
SLFN5	9.66	9.73	16.71	14.83	1.02	0.000000	0.000000
ARRB1	3.98	4.54	4.19	4.06	0.76	0.000030	0.000217
KDM6A	7.58	5.72	7.66	8.58	0.52	0.000300	0.001832
TBC1D5	6.21	6.29	4.58	3.57	-0.46	0.001313	0.006993
NBPF3	5.80	5.34	2.68	2.54	-0.62	0.003076	0.014988
RPA2	10.36	9.41	3.92	3.68	-1.04	0.000005	0.000045
TRIM59	6.49	4.78	1.46	1.62	-1.35	0.000000	0.000000
GPSM2	6.70	6.36	2.18	2.34	-1.40	0.000000	0.000000
RPS6KA1	1.25	1.44	0.42	0.47	-1.43	0.001932	0.009895
C4orf46	3.53	3.28	1.22	1.04	-1.57	0.000000	0.000001
ABHD14A-ACY	1.60	0.97	0.12	0.17	-2.03	0.001692	0.008771
SBSN	1.44	1.10	0.28	0.10	-2.36	0.001565	0.008185
CDC6	17.17	15.64	1.73	1.81	-2.76	0.000000	0.000000
WNK3	0.23	0.16	0.41	0.31	1.24	0.001427	0.007529
ACAD9	19.71	22.60	9.38	10.40	-0.76	0.000000	0.000000
ZEB1	31.96	29.15	39.04	36.69	0.67	0.000000	0.000000
LAT2	0.53	0.61	1.80	2.06	1.93	0.000095	0.000640
AL662795.2	2.46	2.60	3.48	3.16	0.73	0.000476	0.002804
DDX28	3.63	3.33	1.51	1.56	-0.84	0.006095	0.027314
PTCD1	2.05	2.73	1.40	1.27	-0.62	0.007796	0.033922
SNHG8	46.15	40.44	18.85	22.13	-0.59	0.003216	0.015603

PCDHB8	0.04	0.00	0.29	0.17	4.01	0.006765	0.029919
SNX2	16.65	17.83	10.47	9.82	-0.49	0.005630	0.025511
H2BC5	1.93	2.28	3.76	4.66	1.64	0.002083	0.010569
ATRX	19.29	17.00	17.91	17.55	0.24	0.005893	0.026539
BBOF1	1.07	1.64	4.31	3.64	1.55	0.000020	0.000152
PGA5	0.00	0.06	0.68	0.80	4.54	0.000422	0.002503
POLE2	5.12	4.15	1.25	0.62	-2.76	0.000000	0.000000
SHOX2	0.48	0.40	0.92	0.63	1.39	0.003364	0.016224
SAMM50	21.77	20.46	13.10	12.53	-0.38	0.005780	0.026089
BMP2	0.04	0.03	0.48	0.61	4.22	0.000001	0.000012
IGF1	0.01	0.00	0.48	0.17	5.01	0.004980	0.022862
COL26A1	0.00	0.04	0.14	0.31	3.79	0.010295	0.043273
YJEFN3	5.56	5.83	7.89	8.76	0.92	0.000000	0.000000
PCDHGA6	4.18	4.03	4.92	4.96	0.56	0.000144	0.000935
SH3BP2	30.39	30.31	24.85	29.93	0.31	0.001474	0.007756
LARP4	8.94	7.08	5.12	4.09	-0.50	0.000193	0.001221
VPS26A	8.31	7.31	4.42	3.83	-0.63	0.000272	0.001679
NR1D2	4.39	4.09	1.31	1.52	-1.04	0.000000	0.000002
DIAPH1	28.76	30.16	13.52	12.45	-0.80	0.000000	0.000000
CTNNBL1	19.72	14.48	8.35	9.95	-0.44	0.006909	0.030498
TP53I3	17.02	16.01	27.57	25.76	0.97	0.000000	0.000000
AC012321.1	15.47	16.46	15.99	17.23	0.40	0.007602	0.033178
RAD18	3.50	2.78	1.28	1.41	-1.58	0.000000	0.000000
DHX57	8.44	7.45	5.46	5.30	-0.57	0.000121	0.000800
CENPW	7.67	9.03	0.94	0.68	-2.99	0.000000	0.000001
EML1	9.14	8.78	4.97	3.82	-0.74	0.000004	0.000032
TOPBP1	12.59	11.10	5.96	5.91	-0.93	0.000000	0.000000
TSR2	5.07	4.56	2.67	2.60	-0.54	0.003394	0.016346
PRRX2	4.50	4.90	1.63	1.40	-1.29	0.000646	0.003681
MMACHC	1.70	1.46	0.48	0.60	-1.31	0.002011	0.010237
ZNF768	11.47	12.84	5.63	6.61	-0.67	0.000141	0.000914
ID4	0.51	0.67	0.06	0.15	-2.14	0.001499	0.007870
ACTL6A	23.82	23.25	11.67	11.36	-0.79	0.000000	0.000001
FMNL3	6.53	7.61	6.78	7.98	0.58	0.000001	0.000005
CDA	2.10	1.34	0.09	0.17	-3.53	0.003322	0.016051
CSTF2	10.92	11.58	6.36	4.46	-0.72	0.000289	0.001772
FBXO10	4.63	4.80	6.07	7.53	0.83	0.000000	0.000000
ZBTB24	2.09	2.08	0.80	0.94	-0.92	0.000224	0.001398
KMT5A	30.31	29.55	30.95	30.36	0.46	0.000033	0.000234
IFI44L	0.46	0.57	1.07	0.72	2.63	0.000000	0.000000
ADAM8	1.00	0.67	0.84	2.48	1.21	0.008011	0.034716
NUBP1	10.10	9.68	5.23	4.80	-0.65	0.010807	0.045131
NALCN	2.61	2.22	5.57	5.35	1.38	0.000000	0.000000

SGMS1	4.88	6.46	5.98	5.82	0.51	0.001925	0.009859
NDUFS3	35.17	37.55	21.12	20.32	-0.43	0.009187	0.039120
MYO18A	20.42	23.06	12.35	16.60	-0.30	0.004065	0.019181
TDO2	0.03	0.11	0.71	0.90	3.86	0.000152	0.000983
CHRNB1	0.91	1.66	2.73	1.98	1.77	0.000035	0.000250
FBXL19-AS1	1.44	1.39	3.28	3.37	1.57	0.000000	0.000000
PCDHGA2	2.96	2.96	4.91	4.86	1.06	0.000000	0.000000
HYPK	2.32	2.01	3.17	3.52	1.00	0.000004	0.000032
AP3M2	9.43	9.70	12.25	10.53	0.54	0.000382	0.002289
PRR11	15.97	19.20	1.40	1.31	-3.10	0.000000	0.000000
CCNA2	14.78	15.80	1.31	1.06	-3.36	0.000000	0.000000
PHLPP1	1.60	1.50	0.74	0.78	-0.66	0.011997	0.049456
INPPL1	149.18	157.01	137.07	165.82	0.33	0.000029	0.000207
COL21A1	0.10	0.02	4.77	4.67	6.73	0.000000	0.000000
NPPB	0.00	0.11	4.20	3.32	6.47	0.000022	0.000164
EFHD1	0.00	0.00	0.65	0.63	5.70	0.000023	0.000170
ITIH5	0.00	0.00	0.16	0.18	5.59	0.000036	0.000257
AC073610.2	0.00	0.00	1.33	0.91	5.48	0.000061	0.000419
P2RX1	0.02	0.04	0.75	1.06	5.26	0.000000	0.000001
SLC2A5	0.00	0.01	0.22	0.39	4.85	0.005501	0.024992
NRTN	0.00	0.00	0.37	0.66	4.63	0.001608	0.008392
SGCG	0.20	0.11	3.23	2.55	4.55	0.000000	0.000000
TNFSF18	0.10	0.15	2.64	1.88	4.51	0.000000	0.000000
MYL4	0.00	0.00	0.97	0.72	4.48	0.002572	0.012775
H19	0.24	0.20	1.99	1.61	4.25	0.000000	0.000000
TBC1D3C	0.00	0.00	0.33	0.06	4.22	0.007617	0.033229
ASIC4	0.00	0.00	0.20	0.12	4.22	0.005785	0.026096
MUC19	0.00	0.00	0.30	0.20	4.13	0.002871	0.014090
C1QTNF3-AMA	0.00	0.00	0.19	0.01	4.12	0.011542	0.047778
CSTA	0.00	0.00	0.98	0.26	4.12	0.008803	0.037706
TG	0.00	0.00	0.16	0.47	4.11	0.007693	0.033515
TRIL	0.11	0.13	1.37	1.77	4.05	0.000000	0.000000
AC087379.1	0.00	0.00	0.34	0.15	4.01	0.010452	0.043838
KANK3	0.00	0.00	0.29	0.12	4.01	0.010302	0.043288
SLC7A4	0.07	0.05	0.58	0.83	3.94	0.000017	0.000127
ACTN2	0.00	0.04	0.37	0.36	3.91	0.005812	0.026202
KCNJ6	0.00	0.02	0.16	0.09	3.85	0.006959	0.030668
PNCK	0.25	0.19	1.04	1.21	3.84	0.000184	0.001168
FMO2	0.00	0.02	0.82	0.59	3.73	0.010100	0.042534
INHBE	7.14	7.33	81.12	81.61	3.67	0.000000	0.000000
PPM1E	0.01	0.03	0.21	0.20	3.67	0.000097	0.000649
HSPA6	0.09	0.10	0.90	0.86	3.58	0.000002	0.000019
MYH11	1.19	1.41	11.63	11.74	3.57	0.000000	0.000000

PCDHB6	0.09	0.13	0.83	0.89	3.49	0.000000	0.000000
INMT	0.04	0.09	0.50	0.57	3.40	0.000160	0.001029
SLC2A4	0.16	0.03	0.64	0.54	3.37	0.000013	0.000101
SULT1C4	0.55	0.39	2.61	2.71	3.00	0.000000	0.000000
PCDHB15	0.44	0.29	1.64	1.82	2.96	0.000000	0.000000
AC008397.2	0.09	0.06	0.34	0.57	2.92	0.000001	0.000012
CCDC68	0.30	0.58	1.72	1.63	2.90	0.000000	0.000000
C1QL1	0.36	0.48	2.21	2.64	2.88	0.000000	0.000000
RBP1	6.35	7.66	42.45	38.79	2.85	0.000000	0.000000
DHRS3	1.85	1.73	10.27	10.21	2.79	0.000000	0.000000
ARHGAP9	1.30	1.38	6.06	7.95	2.78	0.000000	0.000000
GPR1	1.53	0.62	4.64	4.88	2.77	0.000000	0.000000
PARM1	0.22	0.17	1.10	0.95	2.74	0.000000	0.000000
RORA	0.50	0.41	1.04	0.80	2.71	0.000000	0.000000
CA9	0.11	0.51	2.22	1.99	2.70	0.000092	0.000620
HGFAC	0.21	0.20	0.92	0.70	2.69	0.002728	0.013448
ULBP1	2.29	2.18	11.64	11.11	2.68	0.000000	0.000000
TYRP1	0.22	0.24	0.67	1.23	2.66	0.001662	0.008640
ASXL3	0.06	0.03	0.12	0.34	2.66	0.000860	0.004774
PCDHB12	0.12	0.11	0.57	0.50	2.61	0.000088	0.000595
PCDHB3	0.03	0.12	0.48	0.22	2.60	0.003646	0.017389
H3C6	0.48	0.42	2.07	2.02	2.55	0.000000	0.000000
OSR2	0.34	0.19	1.22	1.31	2.51	0.000166	0.001063
ENPEP	0.04	0.02	0.25	0.14	2.48	0.010350	0.043449
FRY	1.79	1.85	7.65	5.45	2.47	0.000000	0.000000
ABCB1	0.22	0.23	0.58	1.24	2.43	0.000646	0.003682
FLACC1	0.73	0.42	0.75	0.94	2.33	0.007514	0.032809
H2BC18	0.48	0.21	1.26	1.28	2.27	0.000154	0.000992
RAB27B	0.06	0.09	0.38	0.30	2.18	0.000430	0.002551
ANK2	0.85	0.39	1.32	1.68	2.16	0.000000	0.000000
C4A	5.23	6.51	18.36	18.93	2.15	0.000000	0.000000
XAF1	1.41	1.37	6.04	4.56	2.15	0.000000	0.000000
NCAM1	0.19	0.37	0.39	0.46	2.13	0.006999	0.030814
AC093503.3	0.18	0.32	0.81	0.88	2.12	0.000741	0.004172
FGF11	0.90	0.35	1.75	1.94	2.09	0.000008	0.000061
EXTL1	0.23	0.31	0.94	0.90	2.04	0.000020	0.000152
AC010618.3	2.23	1.47	5.45	6.35	2.00	0.000027	0.000199
SERPINI1	0.28	0.31	1.59	0.80	1.95	0.003292	0.015926
RFTN2	0.61	0.67	1.24	1.74	1.91	0.000004	0.000036
WNT2	2.16	1.94	6.25	5.96	1.89	0.000000	0.000000
CRNDE	0.51	0.81	1.20	1.85	1.84	0.000004	0.000035
P4HA2	104.42	103.31	297.97	289.27	1.83	0.000000	0.000000
ARHGEF3	0.48	0.47	1.22	1.15	1.80	0.000010	0.000079

AC005618.1	0.24	0.17	0.48	0.67	1.75	0.008088	0.035022
TSPAN13	2.13	2.73	6.86	5.82	1.72	0.000000	0.000000
GCNA	0.57	0.72	2.20	1.39	1.70	0.000078	0.000532
TP53INP1	2.86	2.88	7.65	6.67	1.65	0.000000	0.000000
SORCS2	0.77	0.48	2.48	2.93	1.64	0.000000	0.000002
EDIL3	43.18	40.10	107.47	97.79	1.63	0.000000	0.000000
BACH2	0.23	0.19	0.61	0.48	1.55	0.000036	0.000256
SPINT2	0.96	1.25	2.22	1.92	1.55	0.011270	0.046807
PPFIA3	0.46	1.02	1.09	1.47	1.54	0.000665	0.003783
P4HA1	113.24	101.20	265.24	229.35	1.54	0.000000	0.000000
OGT	60.98	58.65	148.16	147.41	1.52	0.000000	0.000000
ABTB2	1.17	0.99	2.18	2.21	1.52	0.000000	0.000000
HTR2B	0.55	0.53	1.48	0.99	1.52	0.002665	0.013186
IZUMO4	1.43	0.77	1.99	2.81	1.47	0.009621	0.040808
LINC00472	1.08	0.90	2.05	2.55	1.46	0.000000	0.000000
AC104825.1	0.70	0.41	1.28	1.12	1.46	0.000532	0.003095
GALNT16	1.34	1.00	2.46	2.76	1.44	0.000000	0.000000
ISL2	1.32	1.26	2.75	3.51	1.41	0.000139	0.000905
ZNF528-AS1	2.50	2.75	5.19	5.84	1.41	0.000000	0.000000
LINC01133	1.27	1.45	2.87	2.70	1.38	0.000577	0.003337
SLC2A14	0.90	0.62	1.77	1.36	1.36	0.006039	0.027093
MEGF6	1.06	1.39	2.39	2.32	1.35	0.000000	0.000000
AP003119.3	0.57	0.66	1.38	1.08	1.33	0.000131	0.000858
PCDHGA5	1.90	1.44	3.43	3.16	1.32	0.000000	0.000000
SLC1A2	0.19	0.22	0.66	0.54	1.32	0.005139	0.023482
ZBTB46	1.30	1.48	2.75	2.88	1.31	0.000000	0.000000
MAP2K6	1.21	1.33	3.68	2.73	1.31	0.000000	0.000004
PIK3IP1	2.90	3.29	6.16	6.13	1.31	0.000000	0.000000
AC007191.1	0.56	0.57	1.04	1.08	1.26	0.003451	0.016579
LINC01410	0.92	0.79	2.08	3.04	1.25	0.002667	0.013195
MAN2A1	17.97	15.79	33.87	31.83	1.25	0.000000	0.000000
SGPL1	9.94	10.69	20.02	17.09	1.21	0.000000	0.000000
AL109811.2	3.62	3.46	6.33	5.86	1.21	0.001696	0.008788
KIAA1958	0.37	0.28	0.58	0.52	1.20	0.003136	0.015246
NTM	22.75	23.13	45.71	44.35	1.19	0.000000	0.000000
LINC01409	1.26	2.60	2.40	2.81	1.19	0.003024	0.014761
AC118344.4	0.64	0.88	1.19	1.53	1.19	0.000185	0.001169
SCAND2P	2.42	2.72	2.84	3.04	1.18	0.000000	0.000000
BGN	454.00	486.39	804.91	872.72	1.18	0.000000	0.000000
PIK3C2B	1.92	1.64	3.28	3.52	1.17	0.000000	0.000000
RHBDL1	2.25	2.56	4.11	4.31	1.15	0.000223	0.001395
ZNF596	2.88	2.39	4.14	4.33	1.15	0.000209	0.001314
FP236241.2	1.02	2.99	3.63	3.15	1.13	0.000019	0.000139

BSCL2	13.10	10.66	16.12	18.21	1.12	0.000000	0.000001
STBD1	1.09	0.73	1.88	1.26	1.12	0.001802	0.009297
AP001062.1	1.25	1.13	1.88	2.18	1.11	0.000020	0.000146
PCDHGB2	2.26	2.85	3.87	3.62	1.11	0.000000	0.000002
DNAJB9	6.70	5.76	11.71	9.28	1.10	0.000000	0.000000
MAML3	0.74	0.75	1.30	1.23	1.10	0.000015	0.000114
LINC01278	5.96	6.41	8.78	8.20	1.10	0.000000	0.000000
AC025171.2	2.94	2.53	5.45	4.13	1.08	0.000202	0.001270
ZCCHC18	0.91	0.76	2.35	1.84	1.08	0.012080	0.049729
TUBE1	9.20	6.89	13.90	11.19	1.08	0.000000	0.000000
MUC1	6.02	6.35	16.18	9.60	1.07	0.000001	0.000011
RCOR2	2.88	2.57	3.83	4.57	1.06	0.000019	0.000139
FAM43A	3.51	4.18	6.62	6.06	1.06	0.000000	0.000000
CHPF	65.22	72.16	105.70	118.02	1.06	0.000000	0.000000
AC012360.3	1.12	1.06	1.81	1.73	1.04	0.000615	0.003532
VWCE	1.88	1.91	2.74	4.20	1.02	0.000324	0.001969
HECTD2	4.77	4.32	7.53	6.64	1.01	0.000000	0.000000
PCBD2	0.81	0.89	1.74	1.32	0.98	0.006846	0.030242
AC124312.3	3.01	3.37	5.63	4.23	0.97	0.003250	0.015756
MIRLET7A1HG	4.20	1.43	2.51	3.18	0.97	0.002061	0.010469
SEMA3C	26.22	22.96	38.81	34.41	0.97	0.000000	0.000000
GSDMB	9.40	10.69	14.70	16.43	0.96	0.000000	0.000000
PMEPA1	6.54	7.22	10.74	9.95	0.95	0.000000	0.000000
SUSD6	7.04	6.08	10.11	10.67	0.94	0.000000	0.000000
ISCU	25.36	23.54	36.44	32.13	0.94	0.000000	0.000000
CCDC144A	0.50	0.54	0.71	0.85	0.92	0.001261	0.006747
UBE2Q2P1	4.90	3.67	8.60	7.40	0.91	0.001500	0.007870
CBSL	4.23	4.36	4.43	7.56	0.89	0.004172	0.019617
PCDHGB6	2.18	2.21	3.29	3.13	0.89	0.000004	0.000037
TBC1D3L	6.23	5.68	8.07	9.19	0.88	0.000002	0.000021
AC008443.1	3.00	2.59	4.19	3.73	0.86	0.007772	0.033836
NR2F1-AS1	6.73	5.02	10.88	7.91	0.85	0.000025	0.000184
TET3	1.69	1.83	2.50	2.59	0.85	0.000000	0.000000
CERS5	44.24	46.68	73.10	60.92	0.85	0.000000	0.000000
TMEM119	19.46	19.70	24.91	25.89	0.83	0.000000	0.000000
MAPK8IP3	83.22	86.71	96.91	116.37	0.83	0.000000	0.000000
LPCAT2	6.64	4.03	13.00	9.11	0.82	0.000000	0.000000
ATP9A	4.89	4.53	7.26	6.25	0.81	0.000000	0.000000
HOXB4	6.19	6.93	8.80	9.27	0.80	0.000012	0.000096
TTLL7	6.10	5.87	10.57	10.02	0.79	0.000000	0.000000
SNHG14	43.56	42.40	51.38	51.00	0.78	0.000000	0.000000
PLEKHM1P1	6.39	6.68	7.68	9.67	0.77	0.000004	0.000031
SOX6	2.03	1.39	1.98	2.33	0.76	0.000084	0.000568

AC005261.2	2.55	2.82	3.59	3.63	0.76	0.000056	0.000385
KCTD7	5.14	4.53	7.11	5.79	0.76	0.000004	0.000032
ZNF337	10.67	9.59	13.42	13.55	0.74	0.000000	0.000000
TMEM41B	11.67	10.30	14.52	12.89	0.74	0.000000	0.000000
ECE1	135.52	143.14	180.93	191.75	0.73	0.000000	0.000000
LZTS3	1.59	1.59	1.93	2.15	0.73	0.002617	0.012972
AC069281.2	1.16	1.30	1.62	1.62	0.73	0.006714	0.029731
ZNF84	13.03	12.90	16.80	16.42	0.72	0.000000	0.000000
LYNX1	2.54	2.14	3.17	3.03	0.72	0.002399	0.011987
EPHA4	28.30	29.21	36.39	32.78	0.71	0.000000	0.000000
SPACA6	10.61	13.10	14.32	14.29	0.71	0.000068	0.000464
RTN2	7.61	5.11	6.18	7.93	0.71	0.005020	0.023028
SLC35C2	17.06	19.04	22.57	22.91	0.69	0.000000	0.000000
ALG9	15.90	15.68	20.88	16.74	0.67	0.000000	0.000005
VEZT	28.35	27.34	38.79	31.47	0.67	0.000000	0.000000
MZF1	9.26	9.37	11.70	11.86	0.67	0.000001	0.000008
ANKRD44	1.52	1.65	5.50	3.10	0.65	0.003292	0.015926
PRKG1	3.50	3.22	4.01	3.91	0.65	0.000154	0.000995
ADAM10	83.45	77.31	118.89	104.46	0.64	0.000000	0.000000
NRBP2	29.29	28.18	32.08	37.21	0.63	0.000000	0.000000
ZBTB40	6.28	6.25	7.35	7.79	0.62	0.000000	0.000000
AC018638.4	13.21	12.58	13.97	17.12	0.62	0.001622	0.008453
TEPSIN	17.94	22.54	20.68	22.34	0.61	0.000002	0.000017
RABL2B	11.97	13.93	15.72	15.57	0.61	0.000103	0.000690
B4GALT5	10.14	9.45	12.08	11.40	0.60	0.000000	0.000000
TMEM135	10.74	3.16	5.00	7.81	0.59	0.008002	0.034694
TRPM4	19.51	19.96	22.79	26.85	0.58	0.000005	0.000040
CPSF4	22.23	21.60	28.83	29.50	0.58	0.000035	0.000250
EIF1B	14.09	15.23	17.72	17.39	0.58	0.002721	0.013423
HOXB3	39.87	43.72	48.71	51.65	0.58	0.000000	0.000000
ZNF292	7.10	8.09	10.21	7.60	0.58	0.000000	0.000000
TMEM184C	11.62	12.14	13.43	14.61	0.57	0.000004	0.000035
DNHD1	5.04	5.24	6.15	5.86	0.57	0.000229	0.001430
NPIPA9	232.70	214.07	240.67	273.70	0.57	0.000000	0.000000
ENDOD1	2.47	2.48	2.89	2.91	0.56	0.003460	0.016620
SUCO	14.58	13.72	17.27	15.46	0.56	0.000000	0.000000
XIST	257.90	236.28	267.76	252.18	0.56	0.000000	0.000000
PHTF1	8.97	9.21	11.71	10.58	0.56	0.000235	0.001465
A1BG-AS1	7.23	8.34	7.85	8.93	0.55	0.005247	0.023930
KLHDC8B	7.88	9.45	10.72	10.06	0.55	0.007610	0.033206
KDM6B	9.46	16.58	9.63	17.28	0.55	0.000001	0.000008
RNF146	15.17	14.09	18.05	17.07	0.54	0.000105	0.000702
C20orf194	4.49	4.55	5.16	5.20	0.54	0.000017	0.000125

SYVN1	37.34	40.15	42.90	48.26	0.53	0.000000	0.000000
BFAR	25.98	23.33	34.88	27.21	0.53	0.000001	0.000012
ZNF37A	4.22	4.57	4.41	4.90	0.52	0.000103	0.000688
CALCOCO2	30.01	26.38	30.85	30.82	0.52	0.000019	0.000142
ZSWIM8	80.17	84.16	79.82	102.37	0.51	0.000000	0.000001
ZNF521	3.11	4.49	4.94	4.46	0.50	0.006944	0.030620
ZNF621	5.81	5.44	5.91	6.34	0.50	0.000198	0.001248
DNASE1	7.97	7.00	7.66	8.48	0.49	0.001707	0.008838
TBC1D17	12.24	12.49	11.85	12.79	0.49	0.000716	0.004050
CEP120	3.70	3.56	4.30	4.27	0.49	0.002306	0.011573
CALHM5	4.09	3.76	4.92	3.78	0.48	0.000285	0.001753
MTHFR	4.05	3.99	4.16	4.18	0.48	0.002554	0.012692
PLP2	84.44	75.69	93.15	83.03	0.48	0.000001	0.000010
ABCB10	4.90	5.43	5.60	5.73	0.47	0.002288	0.011490
RCOR3	13.51	14.78	14.03	14.31	0.47	0.000727	0.004103
ZFP90	5.45	4.56	5.43	5.05	0.46	0.003491	0.016735
PHYKPL	15.49	13.98	14.17	18.94	0.46	0.004882	0.022464
CYB5R1	27.13	23.95	27.62	29.10	0.46	0.000170	0.001086
ARL10	2.75	3.45	2.64	3.36	0.46	0.001381	0.007308
SPHK2	9.37	11.71	10.00	12.66	0.46	0.007079	0.031128
INPP5F	11.49	11.32	13.02	11.63	0.45	0.000070	0.000476
SLC38A5	37.03	37.63	36.39	40.70	0.44	0.000341	0.002060
BRAF	3.99	3.02	3.17	3.17	0.44	0.003462	0.016624
AUP1	84.27	83.68	82.50	86.96	0.44	0.000000	0.000003
FZD8	5.77	5.65	5.89	6.22	0.42	0.004136	0.019473
ARRDC3	5.48	5.40	5.80	5.09	0.42	0.008504	0.036633
CHPF2	57.91	60.29	59.69	65.06	0.41	0.000000	0.000001
PIK3C3	13.07	13.10	16.10	11.43	0.41	0.002821	0.013855
SRGAP2B	6.11	5.76	6.63	6.13	0.40	0.007197	0.031581
KLHL29	5.72	6.07	5.90	6.40	0.40	0.001903	0.009764
BSDC1	18.36	18.60	18.44	19.32	0.39	0.000368	0.002208
FAM3C	29.68	29.62	31.04	27.67	0.38	0.001007	0.005509
PIGS	41.25	43.41	41.78	43.35	0.37	0.000013	0.000097
KIFC2	10.31	11.21	11.47	11.42	0.37	0.006943	0.030620
CCNT2	9.70	9.06	11.92	9.97	0.37	0.000622	0.003566
ZRANB2	38.78	35.65	36.85	34.74	0.36	0.000029	0.000210
TMBIM1	45.10	47.00	47.32	46.42	0.36	0.000052	0.000360
KLHL42	10.27	10.06	10.35	10.71	0.36	0.001874	0.009630
XRN1	6.44	4.37	5.08	5.12	0.36	0.005958	0.026790
SELENOM	85.73	84.30	85.65	81.02	0.35	0.001967	0.010039
ETV5	8.40	8.30	8.96	7.75	0.35	0.011991	0.049440
TMEM120B	12.94	10.74	10.74	11.25	0.35	0.002065	0.010485
B4GALT3	17.87	15.50	18.47	17.46	0.34	0.011203	0.046565

RALGDS	17.63	16.34	16.25	18.25	0.34	0.004895	0.022511
WWTR1	22.01	22.95	23.37	22.95	0.33	0.001126	0.006081
CNPY2	68.90	67.36	65.45	65.99	0.32	0.006549	0.029127
PHF12	14.27	17.88	14.69	16.90	0.31	0.004655	0.021570
LAPTM4B	56.12	50.25	55.71	48.72	0.31	0.000478	0.002811
IRF2BPL	9.89	10.45	10.52	9.31	0.30	0.011663	0.048228
TNKS	6.97	5.64	6.20	6.19	0.28	0.005660	0.025631
MBOAT2	33.84	28.89	37.04	35.98	0.27	0.009738	0.041229
PGAP6	37.55	38.26	29.54	42.82	0.27	0.009701	0.041120
MARS1	188.14	223.55	214.39	218.42	0.27	0.000025	0.000181
PCNX4	42.90	35.35	42.43	40.65	0.23	0.009129	0.038893
PLBD2	51.04	48.73	45.56	45.34	0.20	0.002888	0.014161
NPIPB5	104.68	98.47	109.18	100.24	0.20	0.004831	0.022257
RPN1	236.10	233.62	217.45	205.16	0.19	0.001284	0.006854
PRRC2B	57.41	52.87	36.22	43.60	-0.19	0.010527	0.044125
HADHA	37.01	36.91	25.26	24.09	-0.25	0.004896	0.022511
CPSF6	28.57	27.82	18.93	18.56	-0.26	0.007043	0.030988
UBE2Z	39.92	38.02	28.52	27.24	-0.26	0.003620	0.017286
SLC4A7	15.57	13.62	11.81	9.99	-0.26	0.009128	0.038893
CASC3	27.02	25.53	17.90	18.82	-0.27	0.006116	0.027398
DIS3	12.82	12.62	9.25	7.80	-0.29	0.010522	0.044111
ADAMTS6	14.92	14.25	8.02	8.24	-0.30	0.005229	0.023859
ACO2	35.43	35.42	21.96	23.82	-0.31	0.001906	0.009776
NFX1	12.86	12.71	8.56	8.16	-0.31	0.008993	0.038435
IGFBP6	59.99	66.13	37.94	42.54	-0.31	0.011026	0.045915
PBRM1	15.49	14.12	9.36	10.01	-0.32	0.000827	0.004602
XPO1	59.80	56.69	45.97	38.95	-0.32	0.000032	0.000227
THOC5	32.35	29.82	18.30	18.85	-0.33	0.008765	0.037568
JAK1	35.64	30.89	23.67	20.75	-0.33	0.000054	0.000372
VEZF1	13.17	11.60	8.28	7.29	-0.34	0.008344	0.036035
SBNO2	28.70	27.27	16.91	17.41	-0.34	0.000382	0.002289
CHD1	17.61	13.97	9.73	10.35	-0.35	0.003686	0.017551
RBM17	59.12	62.41	36.12	41.92	-0.35	0.002064	0.010481
CRAT	16.00	15.90	9.79	10.71	-0.35	0.009861	0.041661
PLEKHO2	16.88	17.65	9.84	11.54	-0.35	0.003359	0.016204
MMADHC	31.16	30.66	20.50	17.93	-0.36	0.008471	0.036497
TRMT1	32.48	33.68	21.17	25.82	-0.36	0.004448	0.020734
SAFB2	30.63	28.98	24.91	26.55	-0.37	0.000680	0.003859
DPP3	25.81	28.09	16.64	19.55	-0.37	0.003027	0.014771
DHX16	17.78	18.02	11.22	11.91	-0.37	0.003373	0.016263
QRICH1	45.96	42.06	27.78	29.31	-0.38	0.000127	0.000833
TOP2B	48.77	44.30	28.53	28.45	-0.38	0.000007	0.000058
GIGYF2	12.80	13.36	9.29	8.67	-0.39	0.000177	0.001127

HCFC1	23.58	25.04	14.01	14.74	-0.39	0.000020	0.000149
POM121	17.49	16.66	9.76	10.66	-0.40	0.000066	0.000452
MLH1	20.05	18.77	13.83	10.44	-0.40	0.004402	0.020561
RAB11B	28.43	31.17	17.88	18.87	-0.41	0.002532	0.012599
NDUFV1	82.89	90.43	53.81	57.63	-0.41	0.000114	0.000757
NOP9	8.80	8.99	5.34	5.77	-0.41	0.000621	0.003561
GRK2	17.70	17.53	10.59	11.97	-0.41	0.001296	0.006908
MICU1	15.63	14.50	9.66	8.72	-0.41	0.003925	0.018570
CUL4B	14.40	13.97	9.51	9.07	-0.42	0.000352	0.002122
SCAF4	13.42	13.07	8.24	7.77	-0.42	0.000303	0.001853
PCID2	21.01	22.16	11.93	12.40	-0.43	0.001144	0.006175
TAF6	35.23	37.94	18.96	22.27	-0.43	0.000162	0.001041
CDC23	12.20	11.46	7.48	6.13	-0.44	0.002502	0.012454
DGKD	6.13	7.04	5.01	4.40	-0.44	0.003679	0.017524
PCCB	13.39	14.25	8.53	7.72	-0.44	0.009058	0.038641
MAP2K4	5.97	5.87	3.66	3.24	-0.44	0.011461	0.047494
PITPNA	20.22	15.28	8.52	8.22	-0.45	0.000122	0.000804
RANBP3	27.94	27.51	14.87	16.96	-0.45	0.000109	0.000723
DROSHA	25.70	25.86	15.29	17.37	-0.46	0.000005	0.000038
CC2D1B	13.80	13.45	8.71	8.10	-0.46	0.000440	0.002605
CUL2	17.59	14.54	11.65	7.82	-0.46	0.000703	0.003982
METTL2B	8.91	8.57	5.16	4.52	-0.47	0.011940	0.049261
FUS	330.55	352.61	181.24	174.59	-0.47	0.000000	0.000000
MAML2	6.14	5.60	3.31	3.33	-0.47	0.000448	0.002649
TTC19	7.80	8.08	4.77	4.31	-0.47	0.006545	0.029112
RBM27	4.36	6.57	2.19	3.64	-0.48	0.003707	0.017631
OAZ2	29.12	28.58	14.07	14.86	-0.48	0.000345	0.002084
PKNOX1	6.27	6.95	3.87	3.03	-0.48	0.009015	0.038500
SALL2	5.37	4.96	2.73	2.86	-0.48	0.005972	0.026840
WDR7	2.83	3.18	2.92	1.69	-0.49	0.009896	0.041772
NBN	10.83	8.76	7.08	4.81	-0.49	0.000551	0.003196
RAB11FIP1	3.51	3.31	2.39	2.08	-0.49	0.004155	0.019542
WWP2	10.36	9.66	4.89	6.04	-0.49	0.001263	0.006754
CDK14	5.95	4.59	3.10	4.03	-0.49	0.003375	0.016265
SLC17A9	23.25	23.32	13.26	13.79	-0.49	0.000016	0.000118
SMPD4	41.52	42.37	23.67	24.97	-0.49	0.000000	0.000001
RBBP5	4.31	4.02	2.58	2.12	-0.49	0.009831	0.041567
MAD1L1	11.86	13.61	6.59	9.62	-0.50	0.006703	0.029689
H1-10	13.48	14.81	8.57	7.29	-0.50	0.007964	0.034559
KLHDC4	19.94	20.20	10.76	11.35	-0.50	0.000451	0.002667
VPS35L	16.32	14.88	9.61	9.14	-0.50	0.000033	0.000239
DPH1	27.23	28.14	16.70	17.32	-0.50	0.000070	0.000478
RAB11FIP3	18.73	17.34	11.00	9.67	-0.50	0.000082	0.000553

ECD	13.46	11.52	7.30	5.92	-0.50	0.001053	0.005731
MTREX	19.31	19.21	11.94	12.43	-0.50	0.000006	0.000047
CERK	9.81	9.93	5.68	5.24	-0.51	0.000066	0.000453
ERCC3	22.12	23.20	13.37	12.31	-0.51	0.000019	0.000141
MPG	27.01	30.84	14.90	18.05	-0.51	0.001632	0.008502
CBLL1	9.50	9.60	6.33	4.98	-0.51	0.003136	0.015246
EIF3A	85.54	85.98	48.84	47.28	-0.51	0.000000	0.000000
TSN	15.39	13.98	10.75	7.36	-0.51	0.000876	0.004851
NELFA	11.89	11.65	6.29	7.07	-0.51	0.003913	0.018521
PPIG	22.12	22.42	12.98	12.08	-0.51	0.000044	0.000309
DUS3L	18.63	21.33	12.35	13.06	-0.52	0.005106	0.023349
ATG7	12.21	15.15	7.28	6.39	-0.52	0.001475	0.007757
VEGFB	21.18	23.10	10.97	13.33	-0.52	0.004517	0.021010
GTF3C5	27.15	27.24	14.55	15.64	-0.53	0.000047	0.000329
MED14	19.88	16.46	10.18	10.38	-0.53	0.000001	0.000008
GNPDA1	9.06	9.75	6.49	5.26	-0.53	0.006627	0.029406
SNW1	36.63	25.54	18.60	16.10	-0.53	0.000023	0.000172
PLEKHG5	9.46	10.89	5.31	5.42	-0.53	0.000071	0.000483
NGRN	12.82	11.84	7.06	5.89	-0.54	0.000179	0.001138
TTC4	7.34	7.51	4.75	4.71	-0.55	0.006535	0.029079
CUEDC2	29.15	35.38	18.11	17.15	-0.55	0.000904	0.004992
RARG	10.76	13.52	5.73	6.64	-0.56	0.000712	0.004027
VPS72	16.55	19.31	9.78	10.12	-0.56	0.001866	0.009593
RPUSD4	13.84	10.82	7.24	6.98	-0.56	0.001784	0.009210
TEAD1	33.45	31.77	19.82	16.28	-0.56	0.000000	0.000000
ECI2	26.83	26.34	15.13	11.62	-0.57	0.000152	0.000983
SCLT1	5.08	5.00	2.77	2.37	-0.57	0.007493	0.032729
THADA	8.37	11.01	5.00	6.21	-0.57	0.000419	0.002491
CLIP2	17.21	18.74	9.52	9.63	-0.57	0.000000	0.000000
TTC7A	11.32	12.14	7.58	7.58	-0.57	0.000032	0.000232
SUCLG1	30.05	27.24	17.20	14.61	-0.57	0.000180	0.001142
NUBP2	25.54	28.09	14.14	12.68	-0.58	0.000068	0.000464
TRUB2	8.51	8.04	4.82	5.19	-0.58	0.001681	0.008725
DOK4	6.27	7.73	3.27	4.18	-0.58	0.006020	0.027013
LDLRAP1	4.01	4.24	2.27	2.15	-0.58	0.012064	0.049679
ALDH7A1	34.84	34.57	17.06	22.53	-0.59	0.000000	0.000005
EED	7.54	7.73	4.69	3.81	-0.59	0.011538	0.047773
DDX39A	52.28	52.36	26.53	30.94	-0.59	0.000001	0.000010
DCAF1	7.80	7.50	3.69	4.34	-0.60	0.000036	0.000259
KIAA1143	3.77	3.82	1.35	1.32	-0.60	0.007791	0.033907
UTP11	22.28	18.92	10.91	8.63	-0.60	0.001215	0.006522
CSTF2T	6.90	6.25	3.46	3.41	-0.60	0.000175	0.001114
PCIF1	9.47	9.65	4.84	4.90	-0.60	0.000652	0.003717

OBI1	4.12	4.11	2.39	1.89	-0.61	0.005096	0.023305
SHKBP1	34.58	34.57	26.34	19.50	-0.61	0.000001	0.000008
CNOT10	9.53	8.41	6.14	5.34	-0.61	0.003324	0.016054
BAZ1B	25.14	23.87	12.92	12.66	-0.62	0.000000	0.000000
MRPL10	12.25	12.34	6.28	7.07	-0.62	0.000760	0.004271
TFAM	5.80	4.16	3.48	3.73	-0.62	0.007185	0.031533
ERAL1	20.21	19.59	10.67	10.45	-0.62	0.000043	0.000305
ARHGEF10L	4.39	4.86	1.82	2.17	-0.62	0.006262	0.028006
CNOT9	18.66	18.16	10.60	8.27	-0.63	0.000002	0.000020
ZSWIM9	2.94	3.88	1.85	1.64	-0.63	0.010152	0.042733
WTAP	21.38	20.43	9.65	9.94	-0.63	0.000006	0.000046
CENPB	19.64	21.68	10.10	10.97	-0.63	0.000000	0.000002
PPP1R13B	3.85	3.06	1.73	1.60	-0.63	0.011012	0.045872
BCS1L	27.11	31.12	15.11	13.98	-0.64	0.000216	0.001349
RBMX2	6.79	7.58	4.53	3.68	-0.64	0.009080	0.038716
SAP130	8.66	8.19	4.24	4.29	-0.65	0.000006	0.000048
BST1	7.63	5.72	3.58	3.95	-0.65	0.011024	0.045913
POLR2G	33.98	31.58	15.37	14.99	-0.65	0.000341	0.002060
RNF8	6.41	7.57	3.08	3.61	-0.65	0.005353	0.024397
LOXL1	257.00	266.57	129.68	141.77	-0.65	0.000000	0.000000
DHX30	31.45	36.85	16.14	18.99	-0.66	0.000000	0.000000
TMEM200B	5.59	5.24	2.78	2.61	-0.66	0.003478	0.016680
MRPL16	17.21	15.40	8.71	7.92	-0.66	0.001861	0.009578
UTP6	33.45	25.09	16.96	15.43	-0.67	0.000001	0.000009
FLI1	5.17	5.30	2.39	2.67	-0.67	0.004086	0.019267
MRPS18B	19.88	20.66	9.14	9.89	-0.68	0.000096	0.000642
CCP110	3.56	3.50	2.10	1.77	-0.68	0.000769	0.004315
CNTLN	2.92	2.40	1.33	1.16	-0.68	0.002917	0.014283
TTLL5	7.18	4.31	2.67	1.85	-0.69	0.000840	0.004670
ATP5MG	46.57	45.75	28.01	25.20	-0.69	0.000007	0.000057
BAD	12.97	13.53	5.72	7.77	-0.70	0.010738	0.044872
LSM14B	14.02	15.10	6.71	6.58	-0.70	0.000025	0.000186
MFF	29.18	27.27	15.06	12.53	-0.70	0.000001	0.000011
RPUSD2	4.70	4.82	2.43	2.28	-0.70	0.009747	0.041261
NEU3	3.48	3.84	1.76	1.65	-0.70	0.000271	0.001676
ARHGEF19	6.07	6.11	2.68	3.02	-0.70	0.005511	0.025026
SACS	23.06	21.06	11.61	10.69	-0.71	0.000000	0.000000
IWS1	17.74	17.79	9.69	8.65	-0.71	0.000000	0.000000
GMPS	28.68	26.67	15.86	11.35	-0.72	0.000000	0.000000
UCHL5	8.37	9.58	6.22	4.14	-0.72	0.001212	0.006509
CDKN2AIP	3.97	3.75	1.93	1.96	-0.73	0.004188	0.019687
TRMT5	4.42	3.12	3.89	3.31	-0.75	0.002036	0.010356
FBXO17	12.24	12.07	6.38	5.66	-0.75	0.000053	0.000368

MTERF3	7.87	8.29	4.38	3.23	-0.76	0.004810	0.022189
CCDC51	5.52	6.04	3.14	2.63	-0.77	0.007934	0.034451
GCFC2	5.30	3.84	3.39	2.39	-0.77	0.006529	0.029063
KAT14	3.25	2.88	1.70	1.41	-0.77	0.002141	0.010841
PDE4A	3.57	4.27	1.73	1.98	-0.77	0.000392	0.002342
NUP153	14.64	13.83	6.84	6.42	-0.77	0.000000	0.000000
RFC1	14.78	16.33	8.17	7.38	-0.78	0.000000	0.000000
CCDC136	9.81	5.66	4.70	4.10	-0.79	0.002651	0.013130
KCTD17	11.97	13.64	6.15	8.00	-0.79	0.000586	0.003380
PRMT3	5.14	4.53	1.83	1.85	-0.79	0.001291	0.006890
DAGLA	2.29	2.46	0.91	1.24	-0.81	0.000715	0.004044
CELSR2	4.47	4.37	2.08	2.11	-0.82	0.000000	0.000000
BDNF	46.74	45.71	20.87	20.50	-0.82	0.000000	0.000000
TRAPPC4	22.69	23.98	10.73	10.80	-0.82	0.000030	0.000218
ASMTL	15.31	15.61	7.24	6.75	-0.82	0.000000	0.000002
CASP2	9.32	9.19	3.94	3.73	-0.83	0.000000	0.000000
SELENOH	21.15	23.24	10.40	8.52	-0.83	0.000013	0.000098
DHRS11	4.84	4.47	1.96	2.57	-0.83	0.002926	0.014316
UTP15	6.28	5.99	4.76	2.85	-0.83	0.000231	0.001441
DARS2	6.72	6.87	3.35	2.72	-0.83	0.000005	0.000044
SNAPC2	9.73	11.34	4.15	4.79	-0.84	0.000494	0.002900
SNHG26	7.74	6.54	3.37	2.68	-0.84	0.005031	0.023065
TUBGCP3	5.97	6.83	2.65	3.10	-0.84	0.000009	0.000073
NDE1	14.11	11.56	3.58	4.35	-0.84	0.000037	0.000262
FAH	18.83	22.70	8.68	8.08	-0.84	0.000000	0.000003
BARD1	17.46	1.96	1.54	0.89	-0.85	0.002471	0.012309
TFB2M	5.07	5.41	2.66	1.92	-0.86	0.002950	0.014427
SNRPA	45.62	47.99	21.45	20.44	-0.86	0.000000	0.000000
SLC39A3	16.16	19.29	9.63	7.53	-0.88	0.000035	0.000249
FECH	9.85	11.37	5.17	4.84	-0.88	0.000005	0.000042
FAF1	16.55	16.10	7.67	7.40	-0.88	0.000000	0.000002
KCNJ2	1.50	1.04	0.49	0.52	-0.89	0.010260	0.043152
RPP30	10.40	8.93	4.28	4.81	-0.89	0.000996	0.005461
MGMT	5.33	5.59	2.71	1.93	-0.90	0.008353	0.036060
CDC5L	7.70	7.54	3.56	2.92	-0.90	0.000000	0.000000
QSOX2	14.82	16.32	7.42	6.59	-0.90	0.000000	0.000000
CLCN4	1.69	2.42	0.56	0.57	-0.90	0.005199	0.023734
SUPT16H	47.06	43.95	21.48	18.16	-0.90	0.000000	0.000000
NUP85	30.76	28.80	10.66	11.34	-0.91	0.000000	0.000000
SENP1	5.08	4.35	2.04	2.47	-0.91	0.000019	0.000144
SPATA5L1	4.88	4.61	2.35	2.08	-0.91	0.002845	0.013967
SAAL1	7.44	6.82	3.27	2.39	-0.92	0.001958	0.010009
MCMBP	16.97	16.60	8.39	6.84	-0.92	0.000000	0.000000

SRBD1	3.82	3.08	1.41	1.47	-0.92	0.000173	0.001103
ZNF551	2.30	1.85	0.68	0.68	-0.92	0.003164	0.015371
PSME1	39.41	38.33	17.89	14.63	-0.93	0.000000	0.000000
SNHG4	19.09	18.64	8.56	8.21	-0.93	0.000000	0.000000
NIF3L1	12.77	12.14	5.22	5.20	-0.94	0.000034	0.000246
SAMHD1	4.38	5.39	2.82	2.19	-0.94	0.000002	0.000019
NEK4	3.15	4.77	1.55	1.31	-0.94	0.000349	0.002108
SNIP1	3.10	3.57	1.79	1.50	-0.95	0.000155	0.000997
DAZAP2	37.67	34.85	12.33	12.62	-0.95	0.000000	0.000000
SIMC1	4.44	4.80	2.08	2.22	-0.96	0.000024	0.000175
SIGIRR	7.71	8.13	3.70	2.90	-0.96	0.008210	0.035502
MRPL1	10.88	11.26	4.35	4.27	-0.98	0.000134	0.000873
RSRC1	5.66	3.88	2.32	1.36	-0.98	0.002184	0.011034
TMEM177	4.87	4.24	1.53	1.69	-0.99	0.010697	0.044756
UBALD2	6.69	5.53	1.91	3.03	-1.00	0.005719	0.025858
MPLKIP	0.87	1.00	0.38	0.36	-1.00	0.001450	0.007640
CDH4	5.86	5.90	2.37	3.04	-1.00	0.000000	0.000000
KBTBD6	3.05	2.63	1.02	1.21	-1.01	0.000009	0.000073
CLN6	25.17	23.20	8.73	10.33	-1.02	0.000000	0.000000
AC106795.1	12.94	8.71	6.94	3.88	-1.03	0.000376	0.002252
ANP32B	74.48	77.20	31.15	27.65	-1.04	0.000000	0.000000
CABLES2	2.67	2.41	1.04	0.90	-1.04	0.000172	0.001097
TTYH2	3.45	4.45	1.45	1.49	-1.05	0.000025	0.000185
APOO	5.75	6.35	2.86	1.51	-1.06	0.011370	0.047159
MCRIP2	6.28	7.27	2.12	2.64	-1.07	0.000539	0.003130
ANAPC1	9.10	8.40	3.43	3.82	-1.08	0.000000	0.000000
BUB3	51.93	52.30	21.93	18.21	-1.09	0.000000	0.000000
PNPT1	9.36	8.38	3.75	3.08	-1.10	0.000000	0.000000
TUBGCP4	4.61	4.12	1.55	1.68	-1.10	0.000000	0.000000
USP13	4.86	4.27	2.25	1.44	-1.12	0.000000	0.000000
CEP131	8.72	10.42	3.31	4.04	-1.12	0.000000	0.000000
NAF1	4.86	4.25	2.59	1.61	-1.12	0.000339	0.002051
RHNO1	9.32	7.85	2.39	4.09	-1.12	0.000020	0.000147
GPATCH11	2.50	1.73	0.72	0.67	-1.14	0.000313	0.001905
TDP1	8.59	9.76	4.11	3.83	-1.15	0.000000	0.000000
DCLRE1A	1.75	1.46	0.57	0.56	-1.15	0.000521	0.003043
GALK1	9.25	10.73	3.81	2.95	-1.15	0.000024	0.000176
H2AZ2	37.81	36.04	13.22	12.95	-1.15	0.000000	0.000000
IRS1	15.45	16.28	5.22	5.94	-1.15	0.000000	0.000000
TMEM201	11.04	10.51	3.58	4.06	-1.16	0.000000	0.000000
SAC3D1	7.78	8.01	2.26	2.16	-1.16	0.000316	0.001923
CCDC138	2.25	2.32	0.80	0.57	-1.17	0.009477	0.040285
COA6	11.23	11.87	4.09	3.69	-1.17	0.000820	0.004567

GSTO1	48.97	50.77	18.61	16.06	-1.18	0.000000	0.000000
SMC3	19.74	18.41	6.91	6.64	-1.18	0.000000	0.000000
STRBP	1.19	1.32	0.51	0.46	-1.18	0.006592	0.029281
CDKN2AIPNL	4.42	4.74	1.85	1.39	-1.21	0.002502	0.012454
KCNAB3	5.93	7.09	2.48	2.60	-1.21	0.000013	0.000103
SPA17	4.05	3.29	1.72	0.96	-1.21	0.012016	0.049515
UMPS	7.34	10.33	2.51	2.62	-1.22	0.000000	0.000000
SRGN	8.77	10.09	3.37	3.01	-1.22	0.000013	0.000102
HMGB1P5	10.65	9.69	3.51	3.16	-1.22	0.002475	0.012324
TOE1	6.66	6.51	2.90	2.07	-1.22	0.000005	0.000040
AC012513.3	1.98	1.48	0.64	0.52	-1.24	0.008582	0.036883
GRAMD1B	2.08	2.27	0.62	0.63	-1.24	0.000003	0.000027
PMEL	1.57	1.69	0.85	0.57	-1.25	0.011932	0.049237
UNG	6.44	6.74	2.23	2.02	-1.27	0.000000	0.000002
FERMT1	0.91	0.74	0.29	0.16	-1.27	0.009672	0.041008
TAF5	1.71	1.55	0.69	0.38	-1.27	0.001484	0.007795
FAM98B	5.32	8.02	3.37	2.97	-1.27	0.000000	0.000000
DCLRE1B	3.15	2.74	1.01	0.82	-1.28	0.000075	0.000509
WRAP53	5.45	6.58	1.75	1.62	-1.28	0.000002	0.000016
MMS22L	3.46	3.75	1.23	0.71	-1.29	0.000000	0.000001
ABHD17C	3.76	3.66	1.27	1.12	-1.30	0.000024	0.000176
ZNF530	1.72	1.66	0.48	0.37	-1.31	0.001264	0.006758
GMDS	6.86	9.29	4.07	2.39	-1.31	0.000002	0.000016
HIRIP3	6.37	7.29	1.83	2.53	-1.31	0.000005	0.000038
EMC3-AS1	2.17	1.49	0.42	1.33	-1.32	0.007689	0.033505
BRIP1	5.75	4.70	2.02	1.75	-1.32	0.000000	0.000000
HYLS1	2.81	2.26	0.82	0.79	-1.35	0.001505	0.007895
NUDT11	3.99	3.65	1.27	1.10	-1.35	0.000007	0.000059
HAUS4	6.58	5.73	2.88	1.41	-1.36	0.000137	0.000893
PFAS	27.25	27.22	8.97	9.84	-1.39	0.000000	0.000000
RFC4	13.80	13.52	4.06	4.81	-1.41	0.000000	0.000000
STARD8	1.18	1.20	0.30	0.37	-1.43	0.000179	0.001140
DHODH	3.32	2.37	0.61	0.66	-1.43	0.000034	0.000240
ACOT11	1.20	1.10	0.26	0.34	-1.43	0.005324	0.024270
CEND1	3.25	2.78	0.97	0.95	-1.44	0.001226	0.006576
CENPO	13.06	13.08	5.18	4.22	-1.45	0.000000	0.000000
PXMP2	7.00	5.75	1.86	1.64	-1.45	0.000607	0.003488
BCL2L12	9.96	11.67	2.76	3.42	-1.45	0.000013	0.000100
HAUS1	8.78	8.67	2.51	2.05	-1.47	0.000012	0.000096
TMEM155	5.96	5.41	1.23	2.07	-1.48	0.000002	0.000017
ZWILCH	8.19	7.78	2.46	2.38	-1.50	0.000000	0.000000
SNHG19	18.67	20.61	3.71	6.66	-1.52	0.009450	0.040176
CNRIP1	8.65	7.15	2.36	2.07	-1.54	0.000000	0.000000

STX1B	6.65	6.41	2.06	1.81	-1.54	0.000000	0.000000
VAMP5	11.81	11.93	2.86	3.44	-1.54	0.000088	0.000592
CENPX	17.10	18.99	4.95	4.67	-1.55	0.000000	0.000001
CCNE1	2.18	2.64	0.86	0.77	-1.55	0.000773	0.004333
SLC29A1	16.84	15.89	4.01	4.06	-1.59	0.000000	0.000000
PRIM2	5.70	5.82	1.53	1.26	-1.60	0.000000	0.000000
FAM111A-DT	1.10	1.21	0.28	0.22	-1.62	0.008729	0.037427
FN3KRP	12.32	9.93	2.66	3.39	-1.64	0.000000	0.000000
ARRB2	6.35	5.93	1.90	2.41	-1.65	0.000001	0.000008
TIPIN	4.28	4.71	1.27	1.45	-1.66	0.000038	0.000267
KNSTRN	18.98	18.65	4.35	4.82	-1.71	0.000000	0.000000
NAV2	9.03	8.16	3.00	2.54	-1.73	0.000000	0.000000
RFC5	6.51	7.38	1.55	1.62	-1.74	0.000000	0.000000
KPNA2	138.98	134.50	33.32	30.76	-1.77	0.000000	0.000000
MCM6	14.56	14.69	3.64	3.10	-1.78	0.000000	0.000000
DBF4	6.45	7.43	1.80	1.61	-1.78	0.000000	0.000000
GMNN	12.92	11.63	3.19	2.48	-1.88	0.000000	0.000000
STEAP1B	7.27	7.05	1.69	1.41	-1.94	0.000000	0.000001
PRPS2	8.55	10.15	2.02	1.30	-1.95	0.000000	0.000000
RPA3	5.73	4.19	0.83	0.63	-1.99	0.000527	0.003076
RFC2	10.39	11.12	2.53	2.16	-1.99	0.000000	0.000000
TNFAIP8L1	2.54	2.41	0.49	0.49	-2.02	0.000000	0.000000
NUDT1	11.38	14.64	2.42	2.52	-2.02	0.000000	0.000003
SFRP1	38.57	37.16	7.97	7.05	-2.03	0.000000	0.000000
CKS2	28.66	28.47	6.58	4.10	-2.07	0.000000	0.000000
Z83844.3	0.74	1.09	0.15	0.20	-2.07	0.000506	0.002964
AVPI1	10.17	9.06	1.58	2.02	-2.07	0.000000	0.000000
FAM72D	1.20	1.19	0.21	0.22	-2.12	0.000297	0.001816
TEDC1	5.36	8.09	1.42	1.52	-2.14	0.000000	0.000000
BIRC3	0.54	0.60	0.08	0.12	-2.17	0.003498	0.016759
CENPN	9.89	8.63	1.09	0.95	-2.21	0.000000	0.000000
IKBKGP1	2.07	2.75	0.32	0.47	-2.25	0.001005	0.005503
PSMC3IP	10.84	11.21	1.71	1.76	-2.27	0.000000	0.000000
DHFR	13.46	10.61	2.25	2.04	-2.32	0.000000	0.000000
CEP72	2.45	1.64	0.39	0.42	-2.34	0.000009	0.000071
CD68	23.86	28.11	4.05	4.79	-2.34	0.000000	0.000000
MNS1	1.68	1.29	0.28	0.17	-2.37	0.000107	0.000712
SPINT1	1.78	2.11	0.15	0.62	-2.41	0.000019	0.000145
AC112777.1	2.50	1.64	0.39	0.23	-2.42	0.000186	0.001176
SLC37A2	0.88	0.67	0.15	0.32	-2.43	0.000335	0.002028
DSCC1	2.27	1.86	0.26	0.29	-2.44	0.000002	0.000019
FANCB	2.62	2.30	0.36	0.27	-2.61	0.000000	0.000000
GINS3	2.92	2.77	0.39	0.32	-2.65	0.000000	0.000000

CENPI	3.82	3.94	0.32	0.53	-2.67	0.000000	0.000000
MND1	2.55	2.49	0.46	0.11	-2.67	0.002754	0.013562
RAD51	8.49	5.89	0.42	1.28	-2.82	0.000000	0.000000
S100A2	1.34	1.63	0.32	0.19	-2.85	0.007168	0.031482
CENPH	3.11	3.64	0.67	0.58	-2.89	0.000001	0.000012
CENPK	8.35	7.91	0.59	0.80	-2.90	0.000000	0.000000
PSG1	0.78	0.88	0.15	0.09	-2.90	0.000894	0.004945
NTSR1	0.78	0.69	0.11	0.05	-2.90	0.000042	0.000297
RFX8	2.59	2.28	0.25	0.38	-3.01	0.000000	0.000002
C16orf74	3.17	2.25	0.14	0.26	-3.02	0.000412	0.002452
PKP3	0.27	1.03	0.27	0.02	-3.15	0.005161	0.023575
AURKA	35.51	33.13	3.62	2.88	-3.17	0.000000	0.000000
CHAF1B	3.80	3.84	0.27	0.32	-3.19	0.000000	0.000000
AC009549.1	1.06	0.78	0.10	0.06	-3.22	0.000999	0.005472
AC092718.4	4.76	5.20	0.28	0.54	-3.23	0.000010	0.000077
HASPIN	1.73	1.67	0.18	0.09	-3.33	0.000000	0.000000
TCF19	6.01	5.93	0.39	0.44	-3.59	0.000000	0.000000
CDT1	8.34	7.56	0.73	0.73	-3.61	0.000000	0.000000
MYBL2	17.72	18.85	1.11	1.21	-3.65	0.000000	0.000000
MTFR2	2.65	2.34	0.21	0.21	-3.70	0.000000	0.000003
ZNF367	1.72	1.39	0.12	0.06	-3.73	0.000000	0.000000
TRIP13	10.72	11.31	1.10	0.53	-3.75	0.000000	0.000000
NMRAL2P	0.62	0.92	0.00	0.07	-3.83	0.006917	0.030523
SHCBP1	12.18	14.80	0.65	1.00	-3.83	0.000000	0.000000
DTL	8.34	7.62	0.69	0.62	-3.84	0.000000	0.000000
MIR924HG	0.37	0.42	0.00	0.00	-3.86	0.009015	0.038500
SGO1	2.25	1.78	0.08	0.12	-3.88	0.000000	0.000000
THSD8	3.05	2.98	0.16	0.17	-3.94	0.000000	0.000001
FAM83D	12.34	12.66	0.65	0.52	-4.08	0.000000	0.000000
ANXA10	0.37	0.55	0.00	0.00	-4.19	0.003616	0.017272
CDKN3	12.28	12.84	0.56	0.38	-4.25	0.000000	0.000000
CENPM	8.24	6.44	0.26	0.38	-4.25	0.000000	0.000000
NCAPH	11.30	12.27	0.59	0.35	-4.26	0.000000	0.000000
SKA3	5.85	6.05	0.17	0.31	-4.50	0.000000	0.000000
CDC20P1	1.50	1.63	0.04	0.07	-4.53	0.000014	0.000103
MYB	0.32	0.17	0.03	0.00	-4.55	0.011304	0.046908
MCM10	7.15	5.74	0.20	0.22	-4.63	0.000000	0.000000
SAPCD2	2.74	3.05	0.10	0.07	-4.69	0.000000	0.000000
ASF1B	8.48	9.34	0.16	0.49	-5.20	0.000000	0.000000
SPC25	3.20	3.62	0.25	0.04	-5.45	0.000000	0.000000
PPIH	16.01	13.00	4.04	3.03	-1.69	0.000000	0.000005
MAGT1	27.35	23.86	31.77	27.13	0.55	0.000000	0.000000
ZNF207	74.25	72.18	42.27	42.76	-0.28	0.000325	0.001973

CCDC17	0.20	0.34	0.64	0.96	1.80	0.003679	0.017524
ESYT2	24.36	24.71	25.68	26.15	0.23	0.004283	0.020061
WDR75	20.44	16.96	11.07	8.76	-0.55	0.000130	0.000849
CYB5A	10.67	9.58	3.48	3.03	-1.10	0.000768	0.004310
TUBG1	34.97	39.85	13.32	14.45	-1.07	0.000000	0.000000
CEP83	7.91	9.23	4.72	4.08	-0.56	0.009359	0.039818
FSD1	11.74	10.00	3.74	4.28	-0.90	0.000089	0.000601
KIF9-AS1	0.93	0.90	1.22	1.18	0.79	0.009052	0.038623
YPEL5	9.83	9.43	13.71	10.48	0.73	0.000065	0.000447
BMS1	12.18	11.51	6.48	6.28	-0.56	0.000000	0.000000
AC243772.2	0.07	0.05	0.25	0.30	2.60	0.004724	0.021838
CAST	52.56	48.33	32.94	30.07	-0.38	0.000012	0.000092
URGCP	11.65	15.48	6.33	5.49	-0.93	0.000000	0.000000
VSTM2L	0.14	0.12	3.37	3.23	4.83	0.000000	0.000000
DCK	3.66	2.94	1.28	1.37	-0.97	0.001277	0.006819
SLC6A7	0.00	0.00	0.11	0.09	4.22	0.005700	0.025795
KCNK3	0.03	0.03	0.16	0.23	3.54	0.000898	0.004960
ARHGEF37	0.11	0.15	0.57	0.63	2.03	0.000592	0.003410
RC3H1	2.17	2.20	1.98	3.26	0.42	0.007332	0.032068
FBLN7	0.70	0.61	0.10	0.11	-2.62	0.005356	0.024405
NOP53	52.87	52.50	29.78	28.21	-0.64	0.000000	0.000000
DUSP22	3.74	3.45	5.50	4.43	0.89	0.000079	0.000536
KNOP1	12.37	10.35	6.60	7.03	-0.63	0.000467	0.002755
SOS1	8.52	9.93	11.90	12.93	0.62	0.000000	0.000001
MRPS26	11.52	14.25	5.10	4.68	-1.05	0.000079	0.000538
LINC00514	0.05	0.07	0.53	0.56	3.35	0.000731	0.004123
MTCO2P12	5.11	4.91	9.07	6.43	0.98	0.011717	0.048434
DENND6B	1.54	2.16	2.52	3.45	0.94	0.002004	0.010206
OIP5	1.87	1.75	0.05	0.05	-4.94	0.000050	0.000347
SCN2A	0.55	0.47	1.52	1.57	2.04	0.000000	0.000000
MFHAS1	1.76	2.13	1.09	0.76	-0.98	0.000341	0.002059
METTL1	11.80	14.04	6.57	8.71	-0.64	0.006376	0.028476
AUNIP	1.18	1.32	0.17	0.19	-2.15	0.001767	0.009130
CU633967.1	6.83	6.43	5.29	3.65	-0.63	0.010787	0.045052
DACT1	3.45	3.21	7.00	6.75	1.40	0.000000	0.000000
TNIP2	12.30	14.24	6.84	7.61	-0.55	0.003642	0.017375
AC006504.1	0.26	0.24	0.95	0.79	2.14	0.000010	0.000080
CHD7	0.56	0.80	1.27	1.17	1.03	0.001365	0.007234
ZNF419	8.63	7.51	11.36	10.72	0.82	0.000000	0.000000
TUG1	42.00	39.68	46.19	43.46	0.47	0.000000	0.000000
AHCTF1	11.76	10.85	7.01	5.67	-0.56	0.000000	0.000000
TMEM70	7.80	6.96	4.53	3.27	-0.84	0.001252	0.006706
SFXN2	6.12	5.34	2.39	2.69	-0.88	0.000182	0.001157

NFKB1	10.63	9.92	4.41	3.77	-0.98	0.000000	0.000000
PRXL2B	11.27	11.74	4.61	4.75	-1.07	0.000000	0.000000
SLC30A4	0.68	0.69	0.95	0.92	0.72	0.010989	0.045799
FGFR1OP2	11.52	10.27	11.50	12.75	0.62	0.000070	0.000478
TMEM52B	1.08	0.52	1.22	1.35	1.64	0.000438	0.002598
REEP3	5.30	4.63	7.63	7.11	0.53	0.000172	0.001098
GRIPAP1	19.74	18.41	19.82	21.34	0.44	0.000130	0.000852
POP5	16.60	17.66	10.73	7.07	-0.69	0.006678	0.029596
PTGR1	35.07	33.57	14.44	14.83	-0.90	0.000000	0.000000
CEBPZ	18.80	15.19	6.71	6.23	-0.97	0.000000	0.000000
ALDH1L2	17.74	15.86	29.29	26.50	1.03	0.000000	0.000000
CNNM2	5.00	4.57	9.90	9.98	1.43	0.000000	0.000000
ZBTB9	7.93	7.11	3.41	3.97	-0.52	0.011002	0.045847
WBP4	6.66	6.94	3.55	3.22	-0.67	0.001220	0.006550
CCDC34	3.18	3.31	1.02	0.75	-1.44	0.000942	0.005183
SHMT2	103.73	122.44	101.00	115.53	0.22	0.011298	0.046900
ATG9B	0.13	0.14	1.22	1.02	2.91	0.000009	0.000069
BCLAF1	53.24	47.15	40.60	34.83	-0.38	0.000016	0.000121
CDCA7L	10.39	10.51	2.35	1.87	-1.78	0.000000	0.000000
RBBP7	56.56	57.02	35.30	31.35	-0.53	0.000000	0.000000
SUCLG2	16.04	15.27	6.41	5.46	-1.08	0.000000	0.000000
PKD2	35.61	36.59	33.23	34.51	0.31	0.000205	0.001286
NCOA4	30.94	28.21	19.77	17.10	-0.33	0.001106	0.005990
NDUFA4	20.80	17.56	23.35	17.36	0.37	0.010820	0.045167
SECISBP2	13.82	10.86	13.41	14.35	0.52	0.000071	0.000482
NDUFS7	31.21	30.00	15.13	16.87	-0.43	0.006862	0.030307
KBTBD4	6.36	6.28	2.10	2.84	-0.87	0.000364	0.002189
CALML6	0.68	0.22	2.21	1.71	2.62	0.000015	0.000112
CCDC144B	0.87	0.84	1.21	1.40	0.76	0.002446	0.012203
FAM120B	11.29	9.73	7.02	6.51	-0.37	0.004273	0.020033
TSPAN3	115.94	112.43	132.26	125.33	0.53	0.000000	0.000000
AC138393.1	1.64	2.31	3.14	2.99	1.09	0.000848	0.004710
NCOA7	3.68	3.13	6.31	5.65	0.86	0.000001	0.000010
THRAP3	42.69	41.05	26.37	23.91	-0.44	0.000000	0.000000
SRRT	110.93	116.80	50.48	66.93	-0.56	0.000000	0.000000
HIBADH	8.89	8.52	5.27	3.91	-0.59	0.006557	0.029151
ALPL	0.77	0.66	2.57	2.47	2.25	0.000000	0.000000
SFI1	13.16	15.13	5.70	7.39	-0.83	0.000007	0.000053
SORD	10.22	9.20	3.43	2.57	-1.33	0.000000	0.000000
EVI2A	0.63	0.33	0.04	0.00	-4.74	0.007324	0.032047
JMJD1C	14.31	14.10	17.23	14.87	0.38	0.000272	0.001679
UNC13A	0.04	0.04	0.36	0.28	2.91	0.000004	0.000030
COMMD1	9.65	10.92	4.12	3.40	-1.07	0.002273	0.011417

AQR	16.44	15.88	10.57	9.55	-0.50	0.000005	0.000040
NCAPH2	17.28	18.14	6.59	6.72	-1.18	0.000000	0.000000
NEIL3	3.42	3.38	0.20	0.10	-4.14	0.000000	0.000000
FAM219B	27.43	26.72	29.98	30.91	0.52	0.000000	0.000001
TRIT1	10.75	10.41	12.31	13.22	0.50	0.003078	0.014991
MIB2	40.32	47.41	39.41	54.96	0.49	0.000138	0.000898
ARID4A	3.66	3.48	5.03	4.16	0.51	0.006171	0.027631
PCDHB2	0.26	0.18	0.45	0.70	2.14	0.000490	0.002877
PCDHB7	0.53	0.51	1.58	1.46	1.87	0.000000	0.000001
AAR2	11.23	11.80	5.62	5.96	-0.66	0.000052	0.000360
NUDCD3	24.29	23.71	13.34	13.06	-0.53	0.000003	0.000024
N4BP2L1	0.57	0.90	1.54	2.26	1.92	0.000515	0.003012
CYC1	59.44	61.05	29.68	31.61	-0.57	0.000001	0.000006
ASTN1	0.02	0.02	0.42	0.47	4.35	0.000000	0.000004
NIBAN3	0.03	0.09	0.46	0.47	3.65	0.000529	0.003081
UBC	667.52	687.53	423.59	422.19	-0.36	0.000000	0.000000
UBIAD1	4.58	5.15	2.43	2.15	-0.69	0.001054	0.005736
LRRC66	0.27	0.23	0.53	0.59	1.48	0.010616	0.044442
CNTNAP3B	1.12	1.16	2.52	2.35	1.77	0.000000	0.000000
AQP1	0.47	0.71	0.88	0.62	1.67	0.003910	0.018511
MRPL28	55.05	59.69	24.88	33.15	-0.65	0.000006	0.000045
TNFRSF25	13.76	13.31	6.05	7.51	-0.62	0.001461	0.007693
RIF1	7.69	7.91	4.15	3.90	-0.69	0.000000	0.000000
C12orf43	6.76	6.35	3.07	3.90	-0.66	0.006536	0.029079
SLC35B3	8.55	7.44	9.68	9.04	0.57	0.001275	0.006814
ERI1	3.38	3.70	1.40	1.25	-1.15	0.000002	0.000016
FAM13C	0.83	1.07	2.80	2.90	1.80	0.000000	0.000003
PFDN5	184.79	185.85	118.56	108.59	-0.31	0.002205	0.011130
MRPL35	14.86	14.63	7.22	6.56	-0.58	0.001069	0.005807
SULT1E1	0.15	0.08	1.54	1.53	3.95	0.000000	0.000004
SRD5A3	3.18	3.20	5.58	5.15	0.99	0.000024	0.000176
RASGRP2	0.02	0.22	1.05	1.30	4.61	0.000012	0.000091
OPCML	0.02	0.09	1.01	0.22	2.77	0.006185	0.027682
OGA	40.20	37.93	45.88	42.72	0.40	0.000000	0.000004
BRD7	21.69	15.84	8.28	8.55	-0.56	0.000040	0.000285
PREB	20.36	22.95	11.22	12.18	-0.57	0.000027	0.000196
ADAMTS14	0.58	0.61	3.02	3.36	2.74	0.000000	0.000000
AKAP13	15.66	14.33	14.11	14.92	0.42	0.000003	0.000027
NR2C2AP	4.73	5.53	1.01	1.10	-1.80	0.000017	0.000129
AC016588.2	0.72	0.46	1.37	1.15	1.43	0.000685	0.003887
AC021078.1	3.88	3.29	4.81	4.05	0.64	0.000002	0.000018
TBCD	27.14	31.11	12.40	12.91	-1.03	0.000000	0.000000
CCDC88A	17.53	15.31	13.35	10.77	-0.26	0.008780	0.037619

NIBAN1	9.06	8.81	22.65	21.67	1.67	0.000000	0.000000
SMIM34B	0.03	0.04	1.15	1.19	5.96	0.000148	0.000958
CDCA2	6.98	6.19	0.27	0.31	-4.24	0.000000	0.000000
SMN2	17.76	14.83	5.80	8.26	-0.62	0.005652	0.025602
RNF220	25.56	25.26	13.04	15.26	-0.42	0.002088	0.010589
IL18BP	4.45	5.22	5.39	6.63	0.68	0.000326	0.001976
SORD2P	5.06	3.51	1.96	1.07	-1.16	0.000947	0.005209
AC083843.3	0.43	0.47	1.72	1.49	2.17	0.000000	0.000000
DACT3	1.68	2.36	5.75	5.08	1.77	0.000000	0.000000
LUZP2	1.04	1.10	2.79	2.17	1.99	0.000000	0.000000
NDUFA4L2	1.58	2.21	5.69	6.97	2.06	0.000000	0.000000
CFI	0.27	0.11	1.05	1.16	2.62	0.000151	0.000974
LAT	13.98	10.00	14.02	20.40	0.97	0.000000	0.000003
KIAA2013	34.74	34.01	32.04	33.75	0.28	0.001843	0.009489
MYADM	120.51	126.73	79.62	90.97	-0.27	0.000157	0.001009
NUDT21	26.40	28.80	17.59	17.92	-0.80	0.000000	0.000000
PLEKHG4B	1.34	1.67	0.56	0.85	-0.87	0.003219	0.015613
KIF21B	0.30	0.36	0.08	0.07	-1.51	0.006003	0.026957
S100A4	4.44	2.50	0.74	0.15	-2.66	0.008837	0.037833
VPS26B	21.87	21.43	13.74	14.15	-0.45	0.000124	0.000815
ELL2	25.74	26.78	15.29	15.16	-0.69	0.000000	0.000000
CD276	90.99	95.10	114.29	112.95	0.61	0.000000	0.000000
GUF1	6.14	6.19	4.80	3.58	-0.49	0.005316	0.024237
SOX5	0.85	0.46	1.63	1.63	1.50	0.000159	0.001024
TRIM26	12.36	12.30	13.87	12.37	0.40	0.000795	0.004446
WDR70	10.79	9.00	6.15	4.92	-0.49	0.011432	0.047380
APH1B	5.08	3.66	5.30	5.82	0.69	0.000141	0.000915
BZW1	95.72	87.79	49.74	37.79	-0.68	0.000000	0.000000
ARHGAP26	0.35	0.56	2.20	1.99	2.86	0.000000	0.000000
ATP2B1	24.87	20.58	34.36	30.26	0.68	0.000000	0.000000
PLRG1	44.93	49.95	31.85	28.88	-0.32	0.008660	0.037169
TMEM208	24.48	24.70	31.74	30.78	0.70	0.000033	0.000238
ERLIN2	32.64	31.23	37.85	33.81	0.39	0.000003	0.000025
MLLT1	24.53	29.41	15.23	19.06	-0.60	0.000000	0.000000
DLD	27.14	31.08	19.65	17.77	-0.41	0.001025	0.005595
ZNF221	0.77	0.89	2.03	2.01	1.60	0.000091	0.000610
EP400	18.43	17.49	11.19	11.32	-0.41	0.000001	0.000010
BMPR1A	11.94	10.02	14.38	14.93	0.60	0.000000	0.000001
DPYSL2	25.19	23.18	24.59	22.84	0.28	0.000630	0.003604
LAMP3	0.08	0.37	1.07	0.82	3.21	0.000000	0.000005
CLCN5	2.23	2.18	3.77	3.50	1.01	0.000000	0.000000
SYNGAP1	20.06	20.22	19.97	26.55	0.36	0.000383	0.002294
UPF3B	6.73	5.05	3.33	2.69	-0.61	0.010250	0.043134

LRRC8E	4.12	4.73	1.84	1.42	-1.03	0.000020	0.000149
ALG12	8.31	8.32	9.81	8.87	0.55	0.000202	0.001274
SPRY3	0.19	0.21	0.43	0.34	1.26	0.002147	0.010871
POMT2	14.63	14.32	21.47	18.65	0.62	0.000000	0.000000
CKS1B	27.06	28.69	4.27	4.13	-2.34	0.000000	0.000000
PRR26	0.18	0.35	0.46	0.63	2.00	0.001127	0.006085
ST3GAL2	14.95	14.63	21.01	20.49	0.72	0.000000	0.000000
TMEM219	24.48	25.07	26.29	24.61	0.39	0.011053	0.046001
IKBKG	20.94	23.14	10.60	13.75	-0.49	0.001274	0.006810
MZT1	2.59	1.70	0.95	0.42	-1.31	0.004262	0.019994
NAP1L4	39.81	37.15	24.56	20.09	-0.59	0.000000	0.000000
NUP54	14.96	11.82	7.17	5.43	-0.75	0.000012	0.000092
RABL2A	4.36	4.58	10.29	11.18	1.53	0.000000	0.000000
SELENOS	51.91	46.56	59.44	53.40	0.51	0.000003	0.000022
HNRNPH3	116.26	106.65	67.37	60.57	-0.33	0.000150	0.000970
CDKL5	1.14	1.22	1.44	1.47	0.52	0.002033	0.010346
MBD5	4.99	3.45	5.44	4.58	0.50	0.003870	0.018340
PHF19	27.11	26.89	3.99	4.07	-2.44	0.000000	0.000000
ZMYND19	15.50	16.23	9.12	7.54	-0.59	0.001838	0.009467
RPL22L1	32.51	27.30	9.54	7.35	-1.54	0.000000	0.000000
CDCA4	6.05	7.64	1.52	1.21	-2.22	0.000000	0.000000
MOV10	82.35	82.43	48.74	46.59	-0.38	0.000010	0.000079
FGFRL1	32.21	35.57	10.17	11.13	-1.33	0.000000	0.000000
WDR36	9.40	8.09	5.61	5.72	-0.39	0.000752	0.004227
ERCC6L	2.87	2.74	0.13	0.07	-4.51	0.000000	0.000000
HM13	153.75	146.48	186.11	179.93	0.62	0.000000	0.000000
PSME2	75.21	72.76	45.68	48.76	-0.34	0.003968	0.018753
REPS1	10.37	9.65	5.65	4.90	-0.71	0.000119	0.000787
TNFRSF6B	4.49	5.84	1.70	2.32	-1.00	0.010259	0.043152
TNPO3	15.01	15.10	8.66	7.73	-0.55	0.000001	0.000008
SLC35A3	4.12	3.83	5.62	4.99	0.61	0.000527	0.003076
TMEM267	4.41	4.26	7.62	6.34	0.98	0.000089	0.000601
PGA4	0.09	0.00	0.60	0.31	4.12	0.004647	0.021545
SYT7	0.74	0.32	0.95	0.79	2.71	0.000000	0.000000
F11R	0.30	0.21	0.92	0.53	1.74	0.009825	0.041549
PIDD1	15.31	15.31	22.37	25.00	1.12	0.000000	0.000000
PDCD4	22.53	23.77	36.16	37.09	0.92	0.000000	0.000000
ZNF506	1.56	1.31	1.96	1.96	0.87	0.002191	0.011067
UBAP1L	1.99	1.81	1.97	2.27	0.71	0.000620	0.003556
GBP3	6.62	7.15	9.30	7.22	0.60	0.000614	0.003525
CSDC2	9.88	9.76	10.85	11.48	0.51	0.000333	0.002016
CNTROB	27.32	29.50	15.10	15.98	-0.46	0.000876	0.004854
NOL8	13.33	12.54	8.02	6.66	-0.53	0.000238	0.001478

SNAP29	8.25	8.48	5.87	4.19	-0.54	0.007643	0.033323
DENND5B	2.43	1.92	1.07	1.21	-0.71	0.000787	0.004403
TAF10	48.62	59.48	28.31	24.06	-0.73	0.000020	0.000148
ERH	41.26	42.98	22.62	16.31	-0.78	0.000025	0.000184
GSR	13.04	12.74	4.81	6.09	-0.90	0.000000	0.000000
NUFIP1	3.42	3.94	1.48	1.36	-1.04	0.000019	0.000143
UBR7	9.06	7.86	2.51	2.35	-1.60	0.000000	0.000000
LIN9	1.23	1.14	0.25	0.16	-2.23	0.000052	0.000360
MCM8	3.43	2.78	0.32	0.19	-2.98	0.000000	0.000000
RFC3	6.12	4.93	0.51	0.51	-3.09	0.000000	0.000000
AL034430.1	1.31	1.16	0.00	0.04	-4.82	0.005820	0.026234
GATA6	2.66	2.31	3.13	2.84	0.60	0.005927	0.026664
PCDHGA3	1.60	1.93	3.44	3.45	1.39	0.000000	0.000000
NR2F1	43.40	47.91	48.53	55.18	0.58	0.000000	0.000000
L3MBTL2-AS1	0.76	0.72	2.41	2.64	2.32	0.000000	0.000000
LPCAT3	14.81	14.06	20.70	19.58	0.84	0.000000	0.000000
SASS6	1.91	1.70	0.27	0.32	-2.28	0.000000	0.000000
ZNF432	7.32	7.52	11.95	13.09	1.18	0.000000	0.000000
AC005050.2	0.00	0.06	0.64	0.46	4.54	0.011381	0.047193
INO80D	0.97	0.92	1.05	1.04	0.48	0.008182	0.035396
PARPBP	3.41	3.63	0.83	0.50	-2.11	0.000000	0.000001
AC104083.1	0.00	0.00	0.14	0.11	4.01	0.010282	0.043233
MSC	8.33	9.73	12.74	15.17	0.95	0.000000	0.000000
TYRO3	14.98	13.29	14.80	15.09	0.53	0.000007	0.000054
ATP13A1	47.61	60.12	45.24	64.52	0.42	0.000027	0.000198
CPSF1	108.95	122.27	70.67	84.61	-0.27	0.002001	0.010193
DNM1L	22.57	21.21	16.31	15.39	-0.31	0.006193	0.027715
PYCR2	18.90	23.79	12.96	15.06	-0.41	0.004825	0.022241
POLRMT	26.55	21.47	14.77	13.86	-0.47	0.000586	0.003380
PLEKHG3	7.45	7.69	3.36	4.44	-0.53	0.003497	0.016758
TMTC1	13.02	12.60	7.69	7.21	-0.56	0.000000	0.000002
RNF168	3.02	3.18	1.81	1.54	-0.60	0.002653	0.013136
ZC3H4	12.00	11.34	5.45	5.28	-0.65	0.000001	0.000005
MRPL54	18.46	20.40	9.59	8.25	-0.76	0.009139	0.038932
CHRAC1	7.23	6.04	2.64	3.27	-0.83	0.004587	0.021278
TELO2	26.60	27.82	11.25	14.35	-0.86	0.000000	0.000000
IL6R	3.36	3.42	1.27	0.85	-1.18	0.000000	0.000002
C5orf34	1.92	1.59	0.64	0.55	-1.28	0.006395	0.028548
IL17RE	1.63	2.08	0.81	0.58	-1.37	0.004202	0.019743
AMOT	5.65	5.00	1.18	1.57	-1.46	0.000000	0.000000
SNHG16	15.56	15.02	8.09	6.08	-0.70	0.000011	0.000089
DTX1	0.00	0.03	0.69	0.33	4.79	0.005920	0.026637
TRAPPC9	2.43	2.87	1.32	1.48	-0.76	0.002356	0.011798

CD44	804.42	766.18	405.53	374.87	-0.67	0.000000	0.000000
NSF	15.03	14.07	9.06	8.93	-0.41	0.000250	0.001553
BABAM1	33.21	40.71	24.00	22.17	-0.46	0.002052	0.010429
TFAP4	5.74	5.48	0.96	0.99	-1.63	0.000000	0.000003
FGD4	0.45	0.47	0.57	0.88	1.15	0.002193	0.011075
SLK	6.96	6.71	4.28	3.93	-0.40	0.000517	0.003020
AP3B1	20.72	19.64	12.72	11.17	-0.39	0.000275	0.001692
LOXL2	572.45	598.53	778.31	850.43	0.81	0.000000	0.000000
OAS3	2.71	3.04	0.46	0.43	-2.37	0.000000	0.000000
HSD17B12	31.36	31.13	41.27	36.11	0.71	0.000000	0.000000
CTR9	15.41	14.43	10.38	7.96	-0.40	0.000632	0.003613
ITPR2	1.73	1.46	2.50	2.26	0.83	0.000000	0.000000
P2RY11	6.11	6.16	9.14	8.82	0.84	0.000105	0.000697
KANSL1L	1.48	1.20	2.55	1.85	1.00	0.002198	0.011097
YARS2	7.14	6.96	6.22	3.56	-0.67	0.002727	0.013444
NELFE	29.59	29.82	19.27	16.12	-0.35	0.010716	0.044805
SLC44A3-AS1	0.40	0.14	1.12	0.57	2.61	0.005218	0.023814
PELP1	26.76	28.28	13.75	14.57	-0.63	0.000000	0.000001
RNFT2	1.25	1.24	0.26	0.31	-1.75	0.004369	0.020435
NCOA1	3.43	3.21	3.78	4.28	0.58	0.000073	0.000498
PCDHB10	0.31	0.26	2.35	2.16	3.36	0.000000	0.000000
SALL1	0.95	1.21	2.78	2.28	1.74	0.000000	0.000000
CDS2	9.14	8.39	14.72	14.09	0.96	0.000000	0.000000
FAM162A	9.62	11.28	14.50	11.72	0.61	0.009714	0.041158
TMEM115	32.11	34.79	35.71	37.57	0.47	0.000003	0.000023
CLASP1	15.10	15.37	13.18	13.63	0.30	0.000671	0.003814
SVIP	0.88	1.26	0.84	0.83	-1.20	0.010251	0.043134
ARHGAP21	31.89	33.47	21.03	21.09	-0.23	0.007387	0.032298
SNRNP70	170.67	164.55	159.70	160.59	0.29	0.000096	0.000641
C2orf66	0.46	0.43	1.01	1.03	1.53	0.004033	0.019052
ATP13A2	12.54	13.96	32.93	37.55	1.84	0.000000	0.000000
SH3BGRL2	0.32	0.32	0.86	0.64	1.57	0.000291	0.001784
YDJC	5.00	5.94	2.33	2.60	-0.80	0.010883	0.045400
PIK3CD	5.90	7.28	2.25	2.18	-1.25	0.000000	0.000000
SLC25A5	77.48	79.09	25.19	22.17	-1.46	0.000000	0.000000
AKAP1	8.50	10.47	3.45	4.02	-0.95	0.000000	0.000000
AL591845.1	0.41	0.34	0.68	0.87	1.38	0.000694	0.003932
SKA1	4.24	3.58	0.12	0.26	-3.73	0.000000	0.000000
LCLAT1	5.92	5.65	7.25	6.82	0.77	0.000000	0.000000
IMMT	35.13	33.87	23.12	20.21	-0.35	0.000456	0.002694
LINC01191	0.33	0.07	0.96	1.36	3.09	0.002092	0.010605
AC010168.2	0.24	0.18	0.49	0.49	1.56	0.001403	0.007420
CHMP7	10.87	9.25	4.75	4.98	-0.70	0.000033	0.000236

NUP35	6.80	5.84	2.97	1.53	-1.18	0.000364	0.002189
RAB4B-EGLN2	0.00	0.00	0.23	0.33	5.31	0.000123	0.000812
TMEM35A	5.42	4.39	12.12	10.73	1.56	0.000000	0.000000
POLR2H	32.10	31.72	18.69	15.85	-0.56	0.001115	0.006028
TTLL4	19.39	18.06	12.80	13.51	-0.59	0.000069	0.000471
UBE4B	14.59	13.98	8.53	9.76	-0.41	0.000474	0.002793
AC108010.1	5.25	4.41	5.98	6.29	0.69	0.005539	0.025137
MRM1	2.35	2.73	0.73	0.76	-1.45	0.000752	0.004227
GPR37	2.03	1.72	4.42	4.20	1.54	0.000000	0.000000
ACER2	0.53	0.43	1.13	1.02	1.50	0.001268	0.006778
SNHG12	32.15	28.52	18.44	16.63	-0.49	0.001587	0.008284
MED25	14.01	15.21	8.81	8.28	-0.41	0.001425	0.007521
PDCD1LG2	10.41	9.61	3.57	3.44	-1.18	0.000000	0.000000
LONRF2	0.20	0.22	0.28	0.28	1.34	0.000296	0.001814
HECTD1	39.93	34.60	28.90	25.04	-0.21	0.008559	0.036809
TEN1-CDK3	1.45	1.21	1.81	2.13	0.86	0.001673	0.008687
LAMTOR1	32.96	34.95	15.10	16.36	-0.75	0.000001	0.000012
ATRIP	3.23	4.87	1.82	1.22	-1.13	0.000011	0.000082
FSCN2	0.25	0.26	1.67	1.31	2.65	0.000107	0.000710
TMEM237	18.65	17.76	10.63	8.90	-0.66	0.000007	0.000057
PKN1	21.13	21.42	10.51	10.77	-0.61	0.000001	0.000010
CENPA	6.37	5.69	0.29	0.19	-4.13	0.000000	0.000000
YIF1B	44.64	47.94	43.62	47.48	0.33	0.006283	0.028097
PRXL2A	1.37	1.10	3.48	3.54	1.97	0.000000	0.000000
KCTD15	11.68	15.65	21.71	28.70	1.18	0.000000	0.000000
CDK11B	17.64	18.23	10.29	10.38	-0.53	0.000024	0.000179
TMEM87A	25.58	23.47	31.91	28.68	0.66	0.000000	0.000000
RBBP6	19.26	18.46	12.34	12.74	-0.32	0.000602	0.003465
PLEKHA8	2.57	2.50	3.40	3.03	0.79	0.000003	0.000022
CEP85L	1.83	1.37	2.44	1.83	0.58	0.005401	0.024572
VPS13B	9.20	8.31	10.89	9.30	0.53	0.000000	0.000002
UBTF	28.13	29.93	19.18	18.27	-0.31	0.000695	0.003939
RALGAPB	10.65	10.21	11.19	10.30	0.35	0.000157	0.001012
MRPS23	20.78	21.48	11.38	7.95	-0.64	0.000537	0.003122
POR	44.04	46.69	39.70	44.21	0.30	0.004057	0.019150
CWC22	6.49	6.13	3.62	3.15	-0.53	0.003472	0.016658
RCL1	4.73	6.16	2.18	1.94	-1.20	0.000057	0.000391
ZBTB37	0.98	0.83	0.93	0.90	0.57	0.002720	0.013420
NOA1	6.74	6.59	2.99	3.76	-0.64	0.004430	0.020669
ESCO2	2.77	2.31	0.16	0.09	-4.23	0.000000	0.000000
PRMT6	4.60	5.48	2.67	2.27	-0.68	0.003775	0.017918
TSC1	15.07	16.39	18.21	16.76	0.51	0.000000	0.000000
PATZ1	6.32	6.31	3.20	3.54	-0.70	0.000212	0.001333

SEPTIN10	19.52	18.46	10.71	9.90	-0.57	0.000012	0.000092
EMP3	319.39	338.61	220.05	225.32	-0.21	0.009834	0.041573
AKR7A2	16.26	16.17	8.05	7.53	-0.72	0.000227	0.001417
SMG1P7	5.67	5.70	11.84	9.31	1.25	0.000000	0.000000
PCDHGA7	4.68	4.79	5.11	5.11	0.49	0.001148	0.006192
OXCT1	12.39	12.03	7.90	7.29	-0.59	0.000020	0.000151
DDIT3	22.09	25.30	36.70	34.59	0.93	0.000000	0.000000
RMI2	2.50	2.19	0.94	0.51	-1.39	0.010583	0.044319
PCGF5	3.48	3.06	2.67	2.04	-0.64	0.004717	0.021811
MIR210HG	4.56	7.01	15.60	18.97	1.95	0.000000	0.000000
CABIN1	32.55	31.30	26.39	30.67	0.36	0.000337	0.002042
STAG2	10.81	9.47	7.12	6.26	-0.36	0.001477	0.007763
SMG9	35.90	37.80	22.81	24.76	-0.39	0.001953	0.009986
CXorf56	6.32	7.54	4.29	2.69	-0.75	0.006452	0.028770
HAUS8	8.22	7.05	1.00	1.13	-2.44	0.000000	0.000000
MCFD2	61.47	56.39	57.09	52.81	0.27	0.000409	0.002438
ACAP3	76.47	81.52	45.71	51.31	-0.26	0.004806	0.022174
WDFY3	8.52	10.70	9.54	8.69	0.28	0.004358	0.020395
ECPAS	21.40	20.49	15.79	18.60	-0.29	0.000674	0.003828
CERT1	5.63	5.41	5.95	5.24	0.40	0.009952	0.041982
CAMTA2	76.10	88.73	116.73	140.64	0.79	0.000000	0.000000
FRG1	14.90	12.58	7.84	5.86	-0.71	0.007172	0.031490
PLEKHH3	12.11	13.33	13.87	15.84	0.72	0.000000	0.000004
DMAC1	7.24	7.07	4.03	2.84	-1.04	0.001106	0.005990
MCUB	3.05	3.60	2.16	0.71	-1.49	0.000122	0.000805
EGF	0.92	2.10	2.93	4.49	1.61	0.000002	0.000019
COX6A1	156.33	159.43	95.28	92.52	-0.38	0.001094	0.005932
FOPNL	8.64	8.53	3.46	3.15	-1.00	0.000007	0.000055
FLOT2	23.28	23.54	13.59	14.23	-0.43	0.000164	0.001054
SUFU	6.30	6.19	3.70	3.88	-0.42	0.005575	0.025287
ZNF507	3.86	3.48	2.75	2.57	-0.42	0.011508	0.047671
RTL5	1.26	1.18	2.41	2.47	1.31	0.000000	0.000001
DIPK1A	6.66	6.18	15.85	14.69	1.58	0.000000	0.000000
SUSD5	5.56	5.43	2.88	3.22	-0.53	0.001241	0.006654
SLC10A7	2.83	2.81	4.95	3.80	0.91	0.000468	0.002757
ENY2	29.72	32.57	19.84	14.25	-0.45	0.009418	0.040054
ZNF93	3.44	2.83	4.30	3.50	0.67	0.004519	0.021016
IDS	13.67	12.00	13.38	13.52	0.37	0.000890	0.004922
FOCAD	14.92	14.26	6.43	8.47	-0.71	0.000000	0.000000
RIOX1	4.37	4.12	1.67	2.08	-0.84	0.001656	0.008612
ALMS1	4.27	4.55	1.87	2.05	-0.50	0.001942	0.009934
CXADR	0.14	0.18	0.56	0.48	1.54	0.007712	0.033587
FAHD2A	13.05	14.55	7.23	7.01	-0.47	0.010812	0.045143

GRIA4	0.09	0.03	1.09	0.53	3.41	0.000067	0.000462
AC005674.2	2.48	2.60	4.57	4.12	1.11	0.000000	0.000000
FP565260.1	13.75	14.32	5.75	5.24	-1.01	0.000000	0.000000
GTF2F1	43.45	38.86	27.09	23.57	-0.36	0.000332	0.002012
TMED5	20.86	18.20	24.15	21.59	0.39	0.000138	0.000898
GPS1	51.25	52.27	26.73	31.12	-0.42	0.000123	0.000811
GOLGA2P7	22.82	22.02	22.46	23.97	0.35	0.000486	0.002855
LRRCC1	1.43	1.88	0.46	0.45	-1.60	0.000070	0.000477
DIAPH3	9.47	9.46	2.84	2.63	-1.53	0.000000	0.000000
TLE2	0.39	0.51	1.59	1.59	2.57	0.000003	0.000028
CCDC9B	29.50	32.04	11.40	13.84	-0.85	0.000000	0.000000
PINX1	5.35	5.50	2.50	2.17	-0.90	0.008087	0.035022
RGS16	0.06	0.02	0.30	0.43	3.40	0.002800	0.013765
RAB33A	0.71	1.15	3.15	2.54	1.95	0.000709	0.004013
CKAP5	44.39	42.78	18.20	17.13	-0.92	0.000000	0.000000
TNKS2	10.88	10.35	11.44	10.34	0.37	0.000135	0.000883
UHRF1BP1L	5.44	6.09	2.55	2.74	-0.66	0.000242	0.001507
C12orf65	5.57	4.99	2.99	2.16	-0.83	0.007072	0.031102
PRSS53	5.25	5.84	6.18	6.84	0.55	0.000704	0.003986
TMCO4	4.78	4.14	0.91	1.07	-2.04	0.000000	0.000000
MAP3K6	14.56	12.85	6.00	7.77	-0.76	0.000003	0.000027
LZTS1	2.43	2.42	3.68	4.23	1.02	0.000000	0.000000
ENTPD1	0.03	0.00	0.08	0.40	3.89	0.009595	0.040713
GLG1	110.70	113.82	114.07	115.96	0.32	0.000000	0.000001
TEX10	10.22	9.87	5.48	4.75	-0.60	0.000078	0.000529
UCK1	10.49	10.96	4.04	4.84	-0.96	0.000005	0.000041
RTCB	53.42	50.93	26.91	27.25	-0.67	0.000000	0.000000
INTS10	18.55	12.74	7.83	10.19	-0.56	0.000813	0.004538
EXOSC6	4.07	4.30	5.48	4.78	0.63	0.000019	0.000143
CHD1L	19.18	21.72	11.58	10.38	-0.45	0.000537	0.003121
EFL1	5.16	4.22	3.21	1.80	-0.68	0.002444	0.012197
ABCA12	0.03	0.03	0.14	0.18	2.98	0.000188	0.001190
ZNF688	4.07	2.85	3.75	4.19	0.74	0.008420	0.036319
CASP17P	1.52	1.07	2.55	2.85	1.87	0.000032	0.000233
ATL1	2.30	2.15	3.84	3.34	1.03	0.000030	0.000215
HERC2P3	9.36	14.74	19.93	21.23	0.89	0.000041	0.000289
NRSN2	23.08	25.07	26.37	30.28	0.51	0.000024	0.000175
MBTPS2	10.03	8.86	9.60	9.08	0.35	0.004836	0.022277
LSM3	2.78	2.52	1.07	0.97	-1.04	0.000277	0.001702
AC008147.2	0.20	0.22	0.56	0.32	1.41	0.011732	0.048487
IGDCC4	0.71	0.87	10.17	10.32	4.16	0.000000	0.000000
RGPD2	0.28	0.13	0.33	0.27	1.46	0.006316	0.028223
PYCARD	4.42	4.61	1.18	1.25	-1.53	0.007234	0.031710

ASTN2	1.49	1.91	2.91	2.97	1.35	0.000001	0.000006
ASNS	70.14	68.04	144.20	122.13	1.26	0.000000	0.000000
ACVR1	27.01	26.75	36.30	33.58	0.75	0.000000	0.000000
DONSON	9.13	13.06	4.63	4.39	-0.86	0.000019	0.000141
SCRG1	0.14	0.15	3.27	2.64	4.62	0.000000	0.000000
TRAK2	14.09	13.20	21.07	19.25	0.89	0.000000	0.000000
MOGS	62.82	63.73	64.27	68.65	0.44	0.000000	0.000000
PRKAB1	8.79	8.76	8.85	9.99	0.43	0.011890	0.049093
DHPS	37.19	40.57	21.43	25.51	-0.42	0.006504	0.028988
COA3	14.01	16.18	7.27	5.70	-0.86	0.002549	0.012673
NOC3L	11.59	11.13	6.62	5.26	-0.65	0.000009	0.000073
SMC1A	36.50	35.23	11.64	12.92	-1.23	0.000000	0.000000
ERV3-1	5.17	5.13	8.96	8.48	1.11	0.000000	0.000000
FAM214A	6.00	4.95	9.65	7.65	0.83	0.000004	0.000032
RGL1	3.06	2.76	3.39	3.45	0.55	0.001638	0.008524
CYB5R3	167.97	177.30	113.39	124.64	-0.20	0.007704	0.033559
POC1A	6.34	6.50	1.23	1.40	-1.99	0.000000	0.000000
ARPIN	2.02	2.93	1.21	0.99	-0.91	0.000046	0.000320
JAG1	9.96	10.25	19.63	18.55	1.15	0.000000	0.000000
CALR	1,613.76	1,613.94	1,513.49	1,502.58	0.24	0.000007	0.000059
HIC2	1.60	1.56	1.94	1.77	0.56	0.004585	0.021278
ANP32A	53.24	59.68	35.92	35.37	-0.27	0.012034	0.049564
YY1AP1	24.80	25.98	15.70	16.24	-0.32	0.005079	0.023248
FAM20B	9.04	8.24	4.80	4.65	-0.48	0.000110	0.000732
HNRNPL	125.79	135.28	69.15	69.36	-0.55	0.000000	0.000000
SIVA1	21.75	24.59	8.50	11.21	-0.69	0.000528	0.003078
NEK2	7.21	6.96	0.25	0.22	-5.52	0.000000	0.000000
PGF	0.43	0.35	1.19	1.64	2.01	0.000784	0.004390
TMEM156	0.08	0.12	0.55	0.52	2.54	0.009807	0.041485
COL2A1	0.04	0.01	0.19	0.30	3.12	0.008577	0.036875
ALDH18A1	58.97	59.29	29.40	26.79	-0.80	0.000000	0.000000
TMEM117	4.01	3.22	5.87	4.77	0.86	0.000034	0.000241
BVES	2.57	2.59	5.26	4.74	1.19	0.000000	0.000000
SMARCC1	24.42	22.77	17.07	14.60	-0.27	0.001208	0.006487
SIM2	6.73	8.30	4.48	4.72	-0.42	0.009882	0.041720
GOLGA8O	1.12	0.81	1.70	1.65	1.13	0.000030	0.000215
CHROMR	2.55	2.35	3.47	3.79	0.76	0.000728	0.004107
ZNF561	17.36	20.50	32.00	28.90	0.94	0.000000	0.000000
DDX18	26.37	23.06	10.15	8.61	-1.00	0.000000	0.000000
LSM8	5.67	7.10	10.65	6.93	0.65	0.000015	0.000111
SYT16	0.10	0.33	0.35	0.19	2.25	0.000030	0.000220
MIA2	7.19	7.57	8.62	6.92	0.59	0.000635	0.003627
ZC3H14	28.88	26.61	20.94	19.07	-0.34	0.003259	0.015788

AC040160.1	27.11	30.27	4.19	5.59	-2.03	0.000000	0.000000
CDX1	0.21	0.09	0.71	0.72	2.58	0.009789	0.041418
STARD10	7.39	7.51	5.07	4.64	-0.88	0.012073	0.049706
COL25A1	0.26	0.29	0.62	0.66	1.76	0.000087	0.000589
TENT4A	13.19	14.55	10.26	9.44	-0.42	0.000816	0.004549
FPGS	30.19	33.16	13.73	21.61	-0.67	0.000004	0.000033
ZNF185	1.23	1.78	0.46	0.43	-1.44	0.000302	0.001847
RNF130	9.37	9.49	12.35	10.62	0.57	0.000845	0.004697
CCDC47	60.94	58.58	67.13	54.50	0.29	0.000454	0.002682
DVL3	43.32	45.56	44.87	52.23	0.41	0.000000	0.000000
RNASEH2A	8.12	8.43	1.44	0.77	-2.85	0.000000	0.000000
NPTXR	3.17	3.52	3.59	3.51	0.42	0.007476	0.032663
RASGRF2	2.22	2.09	3.97	4.12	1.34	0.000000	0.000000
LRP10	75.91	79.36	107.26	122.19	0.94	0.000000	0.000000
NBEAL2	16.32	19.83	7.75	10.22	-0.44	0.001011	0.005530
DLST	41.83	38.06	24.35	21.07	-0.51	0.000000	0.000002
NEXN-AS1	1.13	1.15	0.18	0.24	-2.11	0.000668	0.003796
GTF2IP12	1.74	2.12	1.96	2.49	0.65	0.003395	0.016347
NBPF8	25.39	23.64	14.92	14.62	-0.40	0.000002	0.000016
GGCT	10.54	9.82	4.79	4.79	-0.72	0.006177	0.027652
DPH2	16.69	16.96	6.31	6.92	-0.88	0.000001	0.000009
CDCA5	13.35	13.18	1.04	0.99	-3.82	0.000000	0.000000
FGL2	0.33	0.42	3.70	2.47	3.30	0.000000	0.000000
INTS1	26.20	29.48	14.34	18.62	-0.54	0.000000	0.000004
HSP90B1	1,129.14	1,106.91	1,060.01	951.09	0.19	0.001965	0.010039
CHSY1	29.20	28.83	30.33	27.98	0.35	0.000010	0.000075
CEMIP2	23.48	23.02	16.10	16.16	-0.26	0.003097	0.015077
NCKAP5L	19.56	21.41	21.37	24.45	0.47	0.000006	0.000049
KDM1A	45.21	42.75	21.34	22.49	-0.62	0.000000	0.000000
ACPP	0.00	0.00	0.38	0.26	5.06	0.000312	0.001902
GPRIN1	4.07	4.00	1.80	1.89	-0.79	0.000087	0.000591
CWF19L1	6.40	5.38	2.50	2.15	-0.95	0.000094	0.000630
WHRN	1.66	1.95	2.63	2.99	1.17	0.000025	0.000183
OBSCN-AS1	0.70	0.96	1.59	1.64	1.25	0.008586	0.036895
AP001972.5	1.20	0.52	1.81	2.00	1.50	0.000493	0.002893
UST	4.99	4.68	6.15	5.52	0.67	0.000026	0.000187
RRAD	0.61	0.71	1.62	1.96	1.75	0.001188	0.006393
ZNF239	7.20	7.42	3.82	3.48	-0.64	0.002470	0.012309
ZBTB38	27.69	21.97	10.29	11.25	-0.80	0.000000	0.000000
ZCRB1	10.73	12.13	5.36	6.96	-0.64	0.007273	0.031849
NARF	20.71	17.94	19.85	21.35	0.48	0.000274	0.001687
MTRR	9.54	13.30	5.83	6.45	-0.69	0.000101	0.000674
MMP11	4.54	3.52	72.74	81.61	4.68	0.000000	0.000000

BICD1	11.24	10.97	6.89	5.57	-0.50	0.000784	0.004390
FOXRED1	17.98	17.56	10.52	10.26	-0.53	0.001029	0.005611
NFS1	9.54	11.68	3.95	4.20	-0.93	0.000020	0.000146
NUP37	12.16	9.72	3.72	3.25	-1.33	0.000003	0.000027
SSC5D	23.48	21.69	26.31	30.22	0.69	0.000000	0.000000
ZNF195	8.22	9.40	8.60	8.07	0.44	0.002956	0.014450
SYT1	4.00	3.97	11.40	9.76	1.69	0.000000	0.000000
ZFYVE1	4.32	3.97	5.06	6.15	0.63	0.000335	0.002027
FEN1	23.24	23.45	2.89	3.29	-2.61	0.000000	0.000000
POLR2B	47.74	43.69	32.81	29.32	-0.24	0.006938	0.030605
AC016876.3	4.57	4.95	1.75	2.76	-0.74	0.000666	0.003788
LY6E	81.78	83.28	36.71	38.84	-0.78	0.000000	0.000000
OGN	0.50	0.52	4.41	3.30	3.90	0.000000	0.000000
DDX23	42.06	41.31	22.26	21.47	-0.63	0.000000	0.000000
STT3A	236.14	228.83	244.14	229.94	0.31	0.000001	0.000006
HELZ2	6.93	8.05	3.38	7.29	-0.42	0.006626	0.029406
IL17RD	0.55	0.43	2.62	2.42	3.02	0.000000	0.000000
SMYD5	13.88	13.44	5.40	6.33	-0.75	0.000012	0.000090
SH3KBP1	14.54	14.34	16.32	12.89	0.36	0.003074	0.014978
CEP55	12.72	12.42	0.65	0.66	-3.88	0.000000	0.000000
ARF5	42.52	46.35	24.21	25.74	-0.40	0.004731	0.021866
CKAP4	403.51	409.16	408.98	394.80	0.32	0.000000	0.000000
LAS1L	17.96	18.16	8.57	8.33	-0.65	0.000002	0.000014
SRRM1	51.97	48.77	32.58	32.35	-0.39	0.000008	0.000065
MMP10	0.03	0.17	3.05	2.36	4.93	0.000000	0.000000
KCNT2	0.31	0.34	2.10	2.11	2.90	0.000000	0.000000
DCUN1D4	12.11	13.22	20.35	15.36	0.59	0.000006	0.000049
MED22	11.78	13.15	6.69	6.78	-0.54	0.000027	0.000195
SUV39H1	4.70	5.53	1.13	0.96	-1.99	0.000000	0.000000
ARL2	24.28	25.86	7.04	6.46	-1.52	0.000000	0.000000
PURPL	0.72	0.21	3.68	3.64	3.57	0.000000	0.000000
PCDHB13	0.22	0.16	0.94	0.99	2.67	0.000000	0.000000
RPLP0P2	1.44	0.31	2.27	3.06	2.19	0.000009	0.000074
AKAP8L	46.73	50.99	57.92	62.51	0.60	0.000000	0.000000
IVD	15.85	19.30	10.19	12.79	-0.41	0.001767	0.009130
TRAP1	47.00	48.07	18.93	20.75	-1.10	0.000000	0.000000
MYO15A	0.32	0.11	0.55	0.54	2.25	0.001330	0.007076
DUSP8	2.68	3.41	1.05	0.73	-1.19	0.000598	0.003446
LCMT2	2.99	3.32	1.85	1.52	-0.74	0.007638	0.033309
SLF1	2.13	2.32	0.99	0.49	-1.76	0.000000	0.000003
QSER1	6.22	6.24	3.03	2.37	-0.94	0.000000	0.000000
SHQ1	5.40	5.65	2.81	2.85	-0.61	0.002878	0.014122
NPTN	191.32	181.93	246.75	248.75	0.72	0.000000	0.000000

PCSK7	41.43	48.97	42.24	49.15	0.31	0.000498	0.002916
USP32P3	1.48	1.77	3.14	2.60	1.61	0.000000	0.000000
HAUS2	3.62	4.71	2.04	1.52	-0.66	0.011482	0.047572
RPAP3	11.24	10.50	5.01	5.24	-0.69	0.000067	0.000457
CCDC115	6.15	4.83	1.93	2.24	-0.90	0.001380	0.007308
AC241952.2	5.94	6.01	6.05	6.28	0.37	0.008510	0.036647
FOXP2	0.31	0.68	0.70	0.38	1.36	0.003177	0.015433
FBXO45	2.14	2.10	0.63	0.68	-1.27	0.000009	0.000071
MAN2B1	56.71	59.04	54.26	64.17	0.46	0.000002	0.000015
AP002990.1	2.57	2.49	3.63	3.76	0.75	0.000173	0.001101
ZNF395	3.10	2.96	3.00	3.92	0.54	0.008589	0.036897
GRIN2C	0.06	0.15	0.42	0.45	2.22	0.001516	0.007946
PIM3	11.54	12.02	4.36	4.03	-1.21	0.000000	0.000000
SOS2	3.32	3.53	4.44	3.53	0.45	0.005083	0.023260
SMARCD3	37.52	38.28	35.22	41.03	0.33	0.003322	0.016051
PHKA2	13.00	11.65	5.29	6.28	-0.78	0.000000	0.000001
CENPQ	1.36	1.26	0.18	0.29	-2.11	0.001889	0.009699
MPHOSPH9	6.68	6.07	3.61	4.17	-0.84	0.000014	0.000110
TSHZ1	5.31	5.44	2.66	2.97	-0.57	0.000394	0.002353
ADAMTS9-AS1	0.00	0.00	0.33	0.37	4.01	0.010452	0.043838
KMT5B	9.36	9.60	12.01	10.50	0.43	0.001697	0.008789
LARS2	10.67	11.40	6.23	6.24	-0.48	0.000056	0.000386
RASGRF2-AS1	0.00	0.02	0.54	0.34	4.90	0.004418	0.020629
SLC26A10	5.96	6.39	11.25	13.99	1.26	0.000000	0.000000
NBR2	2.25	1.91	3.45	3.27	1.19	0.000410	0.002438
MEF2A	8.36	8.38	11.12	10.93	0.72	0.000000	0.000000
MRPS25	26.38	29.03	18.43	15.53	-0.32	0.005759	0.026010
OTUB1	40.08	42.93	25.44	25.74	-0.33	0.002590	0.012855
C11orf68	23.08	24.78	9.84	10.71	-0.89	0.000000	0.000000
FOXE1	0.53	0.35	0.06	0.05	-2.64	0.002240	0.011273
DERL3	0.31	0.28	1.10	1.11	2.00	0.001358	0.007210
RASSF3	8.14	8.47	12.35	13.24	0.87	0.000000	0.000000
MRPL14	14.37	15.55	7.53	6.94	-0.70	0.003336	0.016112
IL27RA	3.98	3.78	1.57	1.74	-0.89	0.000383	0.002294
CBR3	4.54	3.96	1.56	0.92	-1.44	0.003304	0.015975
DDX58	3.78	3.35	4.09	3.80	0.51	0.002582	0.012819
VDAC3	41.44	37.86	27.64	21.83	-0.44	0.000606	0.003484
AIP	17.72	20.54	9.31	10.67	-0.60	0.001583	0.008266
RFWD3	9.17	9.32	2.58	2.27	-1.68	0.000000	0.000000
CHRNA1	1.21	1.05	7.70	6.47	2.97	0.000000	0.000000
SDC2	38.19	34.46	82.99	69.35	1.18	0.000000	0.000000
TGFBR2	33.94	30.56	48.55	43.94	0.86	0.000000	0.000000
ABHD4	7.08	7.56	10.18	11.03	0.84	0.000000	0.000000

VMP1	49.40	56.11	75.36	62.43	0.74	0.000000	0.000000
CLDN12	9.75	8.81	11.21	10.27	0.47	0.000802	0.004480
TOMM7	35.52	34.84	21.00	23.05	-0.55	0.004922	0.022621
BCL7A	2.24	2.50	0.80	0.69	-1.26	0.000029	0.000210
FARP1	44.15	47.92	25.83	21.94	-0.52	0.000000	0.000000
DNTTIP2	20.12	19.36	10.15	9.13	-0.66	0.000002	0.000015
TRIB2	7.66	7.15	12.87	9.95	1.21	0.000000	0.000000
CHERP	21.05	22.41	9.05	9.68	-0.88	0.000000	0.000000
MACROD1	2.75	2.00	0.30	0.53	-3.02	0.000167	0.001068
LINC01128	4.41	4.04	4.54	4.29	0.58	0.000605	0.003482
PRKAR1B	6.23	7.45	3.01	2.58	-0.89	0.000639	0.003650
ALDH16A1	9.72	14.76	4.21	5.66	-1.09	0.000000	0.000000
TEAD3	23.91	24.91	32.83	38.21	0.87	0.000000	0.000000
RAD9A	8.40	7.20	3.66	4.46	-0.66	0.008256	0.035674
ARHGAP19	3.75	4.20	1.03	0.97	-1.56	0.000000	0.000000
DNAJC9	14.08	13.67	6.48	5.62	-0.91	0.000000	0.000000
SH3PXD2A	20.75	20.61	30.74	31.71	0.96	0.000000	0.000000
EFHC1	12.06	10.37	16.09	12.23	0.59	0.000128	0.000842
SHC4	0.51	0.50	1.19	0.98	1.24	0.001193	0.006416
EXOSC10	39.41	37.84	24.69	24.07	-0.45	0.000007	0.000053
CD2BP2	29.58	31.44	21.16	17.83	-0.42	0.000099	0.000660
TIMM23	45.30	39.19	26.20	22.68	-0.45	0.000983	0.005396
MOB1A	9.97	8.57	7.61	6.17	-0.42	0.001113	0.006019
CCNL1	61.13	57.68	54.21	53.27	0.39	0.000002	0.000017
VAMP3	23.03	21.90	17.11	15.77	-0.45	0.000583	0.003369
STK17A	15.86	18.31	11.27	10.18	-0.47	0.001551	0.008117
POLR1B	7.17	7.55	2.93	2.73	-1.14	0.000000	0.000000
RPIA	3.51	3.03	1.14	1.12	-1.19	0.001251	0.006701
TAF9B	4.27	4.64	1.96	1.71	-1.22	0.000002	0.000020
GPER1	1.67	2.01	1.01	0.89	-1.07	0.007743	0.033717
UBL7	16.43	16.58	9.76	9.74	-0.52	0.004067	0.019186
CLSPN	4.46	3.84	0.17	0.12	-4.27	0.000000	0.000000
APEH	44.08	41.00	19.50	24.56	-0.57	0.000000	0.000004
PPP2R5C	17.03	18.66	9.53	7.82	-0.72	0.000001	0.000009
PLEK2	1.63	1.09	0.21	0.55	-2.23	0.007166	0.031481
NPC1	8.02	10.17	14.52	13.23	0.92	0.000000	0.000000
SPARC	5,306.67	5,367.04	12,643.46	12,591.83	1.48	0.000000	0.000000
IFITM2	110.18	123.12	24.52	27.83	-1.78	0.000000	0.000000
KAT6A	4.93	4.68	3.73	3.19	-0.34	0.006018	0.027010
SHLD2	6.98	7.12	4.33	3.53	-0.53	0.001976	0.010080
ASIC1	6.70	7.46	9.56	10.50	0.74	0.000000	0.000002
NXF1	62.47	64.10	56.13	58.41	0.23	0.003113	0.015148
TMEM167A	19.62	18.92	18.10	16.14	0.45	0.000042	0.000294

GATA2	10.22	9.38	3.71	4.14	-1.07	0.000000	0.000000
DHX15	54.72	52.70	31.66	31.05	-0.54	0.000000	0.000000
TIMM10	21.74	23.87	7.28	8.79	-1.20	0.000014	0.000105
IQSEC3	0.05	0.07	0.22	0.47	2.56	0.003207	0.015569
RUFY1	12.75	11.88	7.59	6.77	-0.43	0.008846	0.037860
CTPS2	5.27	5.50	3.15	2.02	-0.70	0.000944	0.005190
SUPT20H	28.59	24.50	28.00	26.85	0.34	0.000871	0.004829
TNK2	16.86	18.68	9.54	10.11	-0.40	0.002112	0.010700
LGALS3	80.62	75.75	42.37	35.71	-0.69	0.000000	0.000000
PCDHB16	0.26	0.79	1.21	1.23	2.24	0.000000	0.000001
AC073869.1	7.15	7.70	8.11	8.68	0.51	0.000169	0.001083
LINC00893	0.86	2.35	4.29	4.27	1.39	0.005432	0.024710
CSRNP2	12.86	12.23	12.36	11.98	0.34	0.002368	0.011857
DYSF	0.07	0.14	0.58	0.74	2.93	0.000000	0.000002
AC011511.4	11.19	11.42	6.00	6.32	-0.54	0.003386	0.016317
TRIP12	54.34	57.61	43.03	37.49	-0.21	0.005615	0.025451
ABI3	0.22	0.53	0.00	0.00	-4.13	0.004515	0.021005
RASL11B	0.64	0.78	2.81	2.66	2.24	0.000000	0.000000
CCDC130	21.43	24.57	21.81	23.57	0.38	0.006591	0.029279
SS18	35.50	36.02	37.95	39.53	0.24	0.007283	0.031891
BLOC1S1	28.18	27.09	12.91	11.91	-0.69	0.004446	0.020729
QDPR	6.65	6.93	3.04	2.81	-1.01	0.000435	0.002582
DHX9	76.06	77.15	38.30	35.58	-0.72	0.000000	0.000000
UBE2T	14.06	12.89	1.35	1.36	-2.91	0.000000	0.000000
HMBOX1	8.51	9.59	18.33	17.22	1.30	0.000000	0.000000
TALDO1	94.33	97.17	37.73	40.40	-0.98	0.000000	0.000000
MRPL43	22.34	26.36	12.16	13.96	-0.49	0.009717	0.041159
VGLL4	8.66	9.96	10.78	11.88	0.55	0.000023	0.000172
WDR34	14.39	15.54	6.12	6.12	-0.99	0.000000	0.000002
ANP32E	16.01	15.64	8.26	9.19	-0.90	0.000000	0.000000
ASB1	21.57	23.41	12.77	11.04	-0.63	0.000000	0.000001
ITGA9	0.04	0.05	0.88	0.95	4.70	0.000000	0.000000
EPS15	7.22	7.01	12.24	11.03	0.83	0.000000	0.000000
SRSF7	75.69	76.76	31.62	32.05	-0.81	0.000000	0.000000
TMEM106C	19.31	19.66	7.98	6.44	-0.90	0.000013	0.000103
HEBP1	21.04	21.22	10.99	9.13	-0.72	0.000098	0.000656
DTYMK	17.75	16.87	2.74	2.28	-2.46	0.000000	0.000000
ERO1A	17.54	14.03	32.80	30.74	1.28	0.000000	0.000000
NXPH4	3.35	3.90	15.08	15.90	2.44	0.000000	0.000000
JPH2	0.21	0.19	0.47	0.68	2.16	0.000148	0.000959
APPL2	16.77	19.69	9.03	11.24	-0.63	0.000005	0.000040
SLC23A3	1.01	0.98	1.96	1.92	1.39	0.000781	0.004372
EBLN3P	8.06	7.88	8.67	7.77	0.41	0.001948	0.009964

IL1RAP	5.24	4.62	6.71	7.35	0.98	0.000000	0.000000
ANKRA2	5.11	3.11	8.78	6.99	0.96	0.000816	0.004548
DDX27	21.93	21.23	11.93	11.21	-0.52	0.000006	0.000049
ATG4A	3.01	2.62	3.70	3.57	0.69	0.010013	0.042222
PSENEN	26.72	28.19	37.75	34.22	0.78	0.000004	0.000037
POLR2D	8.30	7.17	2.76	2.32	-1.22	0.000000	0.000000
ZNF587	11.36	9.59	10.69	10.55	0.31	0.007603	0.033178
COMMD9	16.46	15.41	9.40	8.12	-0.56	0.007233	0.031710
RAB8A	22.06	20.86	9.13	7.54	-1.08	0.000000	0.000000
FOXL2NB	0.01	0.03	0.17	0.32	3.81	0.009662	0.040976
WDR63	0.33	0.47	1.19	1.56	1.66	0.000465	0.002742
CYREN	12.05	11.98	7.45	6.82	-0.54	0.008919	0.038135
AIDA	11.51	10.53	4.46	3.97	-1.03	0.000000	0.000000
ZFYVE26	4.69	4.10	4.17	4.84	0.38	0.003819	0.018108
CMAS	7.90	7.99	4.42	4.33	-0.61	0.009695	0.041101
WASH5P	38.61	43.02	48.83	49.30	0.70	0.000000	0.000000
DNA2	3.85	4.15	1.70	1.42	-1.31	0.000003	0.000028
ATG3	17.51	19.65	11.75	9.07	-0.54	0.001637	0.008524
SPEN	17.55	19.73	9.39	11.62	-0.27	0.002259	0.011356
CCDC15	0.89	1.00	0.35	0.22	-1.29	0.011204	0.046565
NOL10	7.20	7.06	3.09	2.97	-0.91	0.000000	0.000001
MEG8	17.60	14.52	35.64	31.85	1.33	0.000000	0.000000
OSGIN1	9.68	10.79	1.91	2.05	-2.05	0.000000	0.000000
PRKACB	5.79	5.14	7.65	6.21	0.83	0.000000	0.000000
SLC9A1	30.39	30.66	43.24	45.67	0.93	0.000000	0.000000
PDS5A	10.02	10.46	6.77	5.75	-0.37	0.000555	0.003215
AP001273.2	0.00	0.07	0.54	0.28	3.77	0.000386	0.002307
FILIP1L	14.93	15.20	29.37	27.20	1.21	0.000000	0.000000
EIF2AK3	12.23	10.92	16.47	15.02	0.77	0.000000	0.000000
FUT8	9.33	9.60	12.03	9.27	0.52	0.000372	0.002233
CHMP2B	7.14	6.75	3.76	3.31	-0.69	0.001019	0.005572
RGS12	18.13	11.31	7.90	7.76	-0.44	0.003350	0.016164
THOC3	13.36	13.14	4.76	4.87	-1.29	0.000000	0.000000
COPB2	119.32	118.07	87.16	78.41	-0.20	0.003277	0.015867
UHRF1	37.04	39.28	4.82	5.55	-2.60	0.000000	0.000000
NRGN	6.52	7.33	0.42	0.30	-3.99	0.000000	0.000000
LRRC3	0.08	0.13	0.32	0.21	1.65	0.011599	0.047987
RNF138	3.83	3.25	1.62	1.31	-0.80	0.011292	0.046883
SMC6	7.58	6.40	4.85	4.12	-0.40	0.008537	0.036721
SUZ12	8.14	7.70	4.19	3.44	-0.71	0.000002	0.000018
FER1L4	6.70	5.59	10.69	12.81	1.04	0.000000	0.000000
NPIPA7	7.44	10.45	16.80	12.17	1.00	0.000183	0.001161
AGO4	5.17	2.80	4.67	5.22	0.71	0.000013	0.000102

CCNE2	2.66	3.65	0.22	0.23	-3.42	0.000000	0.000000
TLCD4	0.04	0.08	0.21	0.23	2.18	0.001967	0.010039
KBTBD11	0.16	0.12	0.33	0.37	1.64	0.001418	0.007484
BTN3A1	8.14	7.94	14.58	13.44	1.10	0.000000	0.000000
SNRPE	34.06	37.40	17.41	15.46	-0.90	0.000011	0.000088
POLR2C	28.58	27.16	13.31	16.37	-0.68	0.000004	0.000034
NPAT	4.31	2.62	1.16	1.37	-0.82	0.000287	0.001761
ADAMTS16	0.02	0.03	0.40	0.36	4.02	0.000006	0.000050
METTL9	25.43	25.41	18.19	16.25	-0.36	0.007249	0.031765
KLHL25	2.95	3.03	1.36	1.55	-0.69	0.004713	0.021801
PISD	23.99	24.06	13.44	14.52	-0.41	0.001068	0.005801
MASP1	16.96	18.98	17.92	18.56	0.36	0.000856	0.004749
SNHG3	21.83	21.09	13.13	12.24	-0.42	0.000604	0.003478
FIP1L1	31.61	30.48	25.26	19.03	-0.42	0.000688	0.003900
P3H1	198.48	206.10	278.16	291.90	0.87	0.000000	0.000000
LRP3	10.61	12.24	4.06	6.10	-0.78	0.000020	0.000150
QTRT1	27.63	30.98	12.01	14.67	-0.70	0.000014	0.000110
CIAO2B	26.61	28.01	12.86	13.46	-0.69	0.002379	0.011905
CORO1A	4.00	4.63	1.38	1.77	-1.05	0.001363	0.007228
VPS13C	9.06	8.86	9.21	9.77	0.41	0.000118	0.000778
ALG1	33.99	33.96	29.09	32.43	0.28	0.008668	0.037197
DUS2	7.81	7.46	3.49	4.07	-0.65	0.004521	0.021016
CEP85	7.37	8.00	2.32	2.71	-1.29	0.000000	0.000000
SIDT2	13.48	13.89	20.75	23.09	1.07	0.000000	0.000000
NDUFS8	48.74	56.36	25.00	30.06	-0.47	0.002268	0.011393
UPF2	8.59	7.51	4.44	3.79	-0.63	0.000005	0.000041
KRI1	17.47	17.75	9.37	8.81	-0.67	0.000000	0.000002
AL390728.4	20.57	15.88	21.34	19.22	0.52	0.005439	0.024733
NOL11	17.92	16.45	8.03	7.51	-0.77	0.000000	0.000000
BANF1	83.93	85.08	42.27	37.71	-0.73	0.000000	0.000000
CWC27	9.89	8.61	5.41	4.00	-0.64	0.001532	0.008023
DNM3OS	10.71	11.44	36.66	33.47	1.93	0.000000	0.000000
TRIM27	21.55	20.34	13.53	14.11	-0.29	0.010035	0.042300
APCDD1	0.57	1.06	3.27	3.22	2.31	0.000000	0.000000
ANAPC13	12.81	12.98	8.57	7.30	-0.63	0.001887	0.009692
TXLNG	4.92	4.41	2.30	1.71	-0.92	0.000003	0.000023
CADM4	3.64	3.64	4.44	5.23	0.75	0.001380	0.007308
TXN	87.36	86.99	34.29	30.85	-1.08	0.000000	0.000000
SRI	37.32	37.37	17.21	17.60	-0.76	0.000005	0.000040
FAM171B	3.03	2.89	4.56	4.28	0.92	0.000000	0.000000
PHF5A	12.27	11.76	6.28	4.74	-0.79	0.002800	0.013765
DPF1	3.98	4.59	1.03	2.04	-1.68	0.000009	0.000074
SNRPA1	28.57	30.32	16.75	13.36	-0.50	0.001696	0.008788

KDM7A	1.46	0.75	2.65	2.03	1.32	0.000000	0.000000
AC058822.1	0.00	0.00	0.47	0.75	6.28	0.000002	0.000016
DGKH	1.65	1.65	2.85	2.36	1.21	0.000000	0.000000
SMARCA4	77.78	77.96	50.36	48.40	-0.41	0.000000	0.000000
GCN1	54.80	62.17	35.98	36.94	-0.49	0.000000	0.000000
CNNM4	2.75	3.18	1.35	1.56	-0.59	0.007995	0.034667
SLC35F2	7.44	7.39	2.64	2.73	-0.92	0.000007	0.000054
CIAO1	17.31	17.23	10.45	11.46	-0.30	0.007906	0.034345
BLM	2.48	2.49	0.66	0.74	-3.79	0.000000	0.000000
BORA	3.27	2.41	0.44	0.26	-2.64	0.000000	0.000000
CARD9	1.44	1.85	2.63	3.15	1.15	0.000038	0.000270
HSD17B14	1.09	1.76	4.90	4.47	1.94	0.000021	0.000158
TMEM147	30.83	28.69	16.19	16.56	-0.47	0.007225	0.031687
MKNK2	17.20	19.05	22.36	21.55	0.65	0.000000	0.000000
PRPF31	32.04	31.17	18.62	20.14	-0.40	0.001828	0.009420
NAGK	48.08	52.93	53.65	51.94	0.57	0.000000	0.000000
HARS2	21.48	19.22	12.20	12.52	-0.44	0.001302	0.006938
RBM33	11.13	11.17	8.20	9.87	0.27	0.010750	0.044913
HMCES	10.98	10.49	5.00	5.15	-0.74	0.000107	0.000710
NPTN-IT1	1.95	1.43	3.64	3.01	1.31	0.000013	0.000099
POGLUT2	15.38	14.37	17.06	14.96	0.43	0.001907	0.009776
FZD1	4.46	4.58	6.72	6.13	0.84	0.000000	0.000000
DSTYK	4.86	4.38	2.90	2.78	-0.37	0.006214	0.027804
MRPL58	13.55	13.91	6.41	5.86	-0.81	0.002537	0.012620
SH3D19	9.74	9.45	5.25	4.68	-0.61	0.000009	0.000069
RCAN1	20.73	20.58	10.09	9.49	-0.79	0.000000	0.000000
TUBA4A	11.47	13.64	3.97	4.66	-1.31	0.000000	0.000001
APEX2	6.35	6.54	3.37	2.98	-0.73	0.000073	0.000497
SLC39A10	15.43	14.87	18.25	15.58	0.44	0.000007	0.000059
OSBPL6	2.45	2.02	3.02	2.93	0.68	0.000794	0.004439
SAMD5	0.51	0.65	1.09	0.84	1.06	0.000643	0.003671
ABL1	38.16	40.20	22.64	25.30	-0.28	0.000741	0.004174
RUNDC1	3.16	2.61	3.91	3.32	0.65	0.001405	0.007425
TTC27	7.64	7.40	2.93	2.64	-1.12	0.000000	0.000000
CCDC84	17.09	17.18	8.62	9.87	-0.53	0.003530	0.016901
RNPEP	30.35	32.07	18.45	19.89	-0.51	0.000003	0.000027
MBOAT7	110.37	111.05	108.61	123.61	0.34	0.000004	0.000035
UBE2G1	11.86	11.78	6.16	5.29	-0.59	0.000048	0.000334
TRPC1	3.07	3.11	3.71	3.11	0.54	0.007159	0.031457
PDE4C	0.02	0.18	1.16	2.13	5.23	0.000000	0.000001
PCDHA4	0.01	0.00	0.23	0.10	5.35	0.001986	0.010126
ARVCF	7.73	7.22	8.39	8.56	0.56	0.001249	0.006693
NNMT	46.73	47.55	49.87	47.91	0.35	0.004279	0.020054

PCDHGB5	22.69	22.30	28.08	29.26	0.68	0.000000	0.000000
RMI1	4.04	3.09	1.81	1.34	-0.74	0.006564	0.029178
NECTIN2	131.33	145.14	191.33	199.18	0.88	0.000000	0.000000
XIRP1	0.02	0.00	0.27	0.52	6.65	0.000023	0.000167
LYAR	7.60	7.36	2.03	2.05	-1.51	0.000000	0.000001
UQCRC2	69.82	71.21	50.17	44.68	-0.37	0.000118	0.000778
CACNA1G	0.00	0.04	0.16	0.09	3.69	0.002256	0.011345
COL5A3	11.11	11.41	29.08	33.56	1.83	0.000000	0.000000
DEPDC1B	2.65	1.85	0.22	0.43	-2.81	0.000000	0.000000
MRPS27	20.81	19.12	8.73	7.33	-0.94	0.000000	0.000000
KLHL4	0.18	0.22	0.71	0.52	1.95	0.000507	0.002964
AR	0.88	1.15	0.37	0.38	-0.98	0.000582	0.003362
CFAP298	10.25	12.77	4.63	4.39	-0.85	0.000123	0.000807
CSE1L	31.54	28.45	9.84	8.35	-1.36	0.000000	0.000000
MPP6	6.25	3.94	2.53	2.00	-0.75	0.001596	0.008329
PCLAF	18.05	13.84	1.18	0.43	-4.16	0.000000	0.000000
CUL4A	33.39	33.84	17.96	20.16	-0.73	0.000000	0.000000
PIGG	31.10	30.19	32.99	30.30	0.38	0.000027	0.000198
USP24	11.78	13.96	11.40	11.45	-0.31	0.001096	0.005940
EIF2AK1	20.35	18.32	13.31	11.46	-0.40	0.000255	0.001579
KIFBP	15.53	14.70	9.02	8.16	-0.49	0.000640	0.003652
COMMD3-BMI1	0.77	0.92	0.00	0.00	-4.61	0.000934	0.005144
DUBR	2.22	2.61	3.11	2.96	0.89	0.000258	0.001593
MPHOSPH10	18.82	18.34	11.94	10.69	-0.46	0.000612	0.003518
ERI3	17.61	18.16	9.38	10.18	-0.50	0.002717	0.013406
APOLD1	1.15	1.75	0.24	0.26	-1.77	0.000032	0.000230
EFNB1	12.25	12.97	24.88	24.20	1.30	0.000000	0.000000
PBX3	4.25	4.03	6.32	4.73	0.62	0.005238	0.023894
FYCO1	6.49	6.46	4.32	3.80	-0.31	0.010090	0.042501
ME2	13.06	11.09	6.10	5.09	-0.74	0.000007	0.000059
HSPA4L	1.89	1.71	2.63	2.02	0.77	0.000623	0.003567
AC004943.2	0.87	0.57	0.21	0.18	-1.57	0.001917	0.009824
NLGN3	0.28	0.30	0.59	0.72	1.69	0.002318	0.011631
HPDL	0.63	0.50	0.03	0.03	-3.93	0.004175	0.019626
FOXS1	0.00	0.05	0.91	1.74	6.12	0.000093	0.000623
BMP1	69.33	75.21	120.20	126.78	0.98	0.000000	0.000000
PPP5C	38.58	40.64	23.54	20.50	-0.56	0.000000	0.000004
PLPPR3	0.80	1.59	1.64	1.95	1.33	0.000796	0.004447
MAPKAPK2	21.40	21.34	8.64	11.38	-0.90	0.000000	0.000000
NAB2	27.72	29.21	12.68	13.59	-0.79	0.000000	0.000000
RNFT1	4.51	4.84	7.26	7.14	0.99	0.000012	0.000090
ESD	38.15	39.01	13.73	13.05	-1.10	0.000000	0.000000
SNX9	15.63	15.80	10.65	9.85	-0.28	0.007296	0.031941

DAGLB	14.58	15.88	7.93	8.71	-0.59	0.000022	0.000166
MSC-AS1	3.13	3.71	4.60	5.06	0.74	0.000245	0.001519
CCDC74A	3.69	2.78	5.22	7.08	1.15	0.000631	0.003609
TICRR	4.71	4.87	0.48	0.38	-3.09	0.000000	0.000000
PIP5KL1	2.03	1.72	2.53	4.96	1.38	0.000182	0.001155
GGT7	19.75	19.01	22.42	23.22	0.57	0.000000	0.000002
SDF4	232.30	251.59	225.92	251.76	0.26	0.000971	0.005332
TXLNB	0.00	0.00	0.16	0.23	5.56	0.000045	0.000315
RAE1	20.75	17.43	12.69	9.75	-0.44	0.002373	0.011879
NPIPP1	45.26	40.85	41.76	48.42	0.39	0.001517	0.007950
PSMD10	14.95	14.66	8.51	7.56	-0.52	0.006073	0.027221
PRPF4	9.14	9.30	3.73	2.49	-1.29	0.000000	0.000000
REXO5	2.18	2.34	0.44	0.72	-2.37	0.000012	0.000090
GMIP	7.01	7.06	10.22	8.73	0.47	0.005042	0.023112
SORBS1	0.16	0.15	0.50	0.33	2.08	0.006828	0.030175
CACFD1	3.85	3.36	5.64	5.94	1.08	0.000000	0.000001
GPC4	0.72	0.51	1.16	0.78	0.99	0.006007	0.026968
ATG2A	7.93	9.87	3.99	6.00	-0.58	0.000396	0.002365
Z83843.1	2.30	2.51	3.88	3.81	1.01	0.000001	0.000005
PLCXD1	6.57	7.88	3.26	5.21	-0.78	0.000132	0.000866
FBXL7	5.25	5.49	8.23	7.06	0.86	0.000000	0.000000
PDIA3	600.56	576.94	759.99	655.50	0.58	0.000000	0.000000
RB1	5.34	5.80	3.59	3.68	-0.49	0.002918	0.014287
PTCHD4	0.30	0.40	1.92	1.22	2.45	0.000000	0.000000
SNHG17	20.08	20.27	11.92	12.33	-0.44	0.003207	0.015569
ODF2	29.96	30.19	17.12	18.42	-0.42	0.000016	0.000124
PCNA	55.05	52.87	21.24	18.29	-1.11	0.000000	0.000000
RILPL2	0.62	0.74	0.27	0.20	-1.19	0.004755	0.021966
MAPK1IP1L	35.88	31.82	19.52	21.43	-0.56	0.000001	0.000006
USP1	9.27	9.02	2.71	2.17	-1.57	0.000000	0.000000
SLC3A2	240.74	233.51	230.70	228.73	0.25	0.000136	0.000885
C2orf27A	2.52	2.50	4.62	4.80	1.32	0.000001	0.000009
TRPS1	2.71	1.93	3.35	2.59	0.72	0.000117	0.000772
RHOBTB1	4.90	4.54	6.54	6.95	0.68	0.000016	0.000122
ORC3	8.69	7.67	4.50	3.84	-0.62	0.001252	0.006706
IKBKE	6.95	7.76	3.42	3.81	-0.57	0.001653	0.008599
BID	28.57	29.23	15.74	12.40	-0.90	0.000000	0.000000
WBP1	30.72	30.89	39.64	42.95	0.88	0.000000	0.000000
ZNF841	8.16	8.00	9.64	11.77	0.68	0.000000	0.000002
AC137936.2	0.15	0.19	1.21	1.05	3.05	0.004078	0.019232
BNC1	2.96	2.75	1.50	1.37	-0.64	0.004242	0.019906
HTATSF1	18.69	19.49	10.42	9.74	-0.60	0.000002	0.000013
PLA2G15	14.45	14.29	15.07	15.77	0.39	0.002688	0.013286

SLC35C1	11.45	11.46	11.67	12.20	0.36	0.003510	0.016808
ERMAP	4.86	4.58	5.24	4.55	0.59	0.001115	0.006028
EPM2AIP1	4.44	4.97	5.55	4.03	0.35	0.005719	0.025858
TRMT6	5.54	5.12	2.48	2.34	-0.96	0.000022	0.000162
STAT5B	14.04	16.12	15.42	15.85	0.30	0.005136	0.023481
PAF1	27.96	26.91	16.45	17.08	-0.39	0.000704	0.003988
CDR2	23.93	21.83	11.47	11.73	-0.67	0.000000	0.000000
USP37	1.41	1.23	0.60	0.51	-0.76	0.004057	0.019150
LEF1	0.00	0.00	0.62	0.57	4.95	0.000480	0.002823
IPO9	24.22	18.11	11.14	10.67	-0.45	0.000000	0.000003
MEPCE	13.09	15.42	6.33	6.22	-0.86	0.000000	0.000000
HSPBP1	14.27	16.34	6.80	6.48	-0.92	0.000001	0.000011
NAT10	22.50	19.62	11.23	10.26	-0.91	0.000000	0.000000
DGCR6L	19.67	24.63	10.69	11.13	-0.69	0.000273	0.001686
C5AR1	1.11	1.20	1.94	2.42	1.40	0.000250	0.001552
ERGIC1	68.62	70.45	33.03	32.29	-0.80	0.000000	0.000000
MCM2	20.59	20.23	1.32	1.29	-3.57	0.000000	0.000000
BRWD3	3.65	2.67	2.60	3.64	0.40	0.003764	0.017874
HMGN2	119.68	115.79	29.85	28.72	-1.81	0.000000	0.000000
NMD3	18.46	17.84	10.70	9.10	-0.46	0.000316	0.001922
CDCA7	7.81	7.29	0.87	0.70	-2.86	0.000000	0.000000
NAXE	27.53	28.20	12.24	10.64	-0.92	0.000000	0.000001
ATP6V0E2	10.33	12.88	2.66	2.47	-1.83	0.000000	0.000000
APOBEC3B	11.33	12.60	0.27	0.34	-5.08	0.000000	0.000000
AL109976.1	0.07	0.05	0.21	0.22	2.24	0.003901	0.018470
MIR503HG	148.80	145.82	217.49	213.29	0.85	0.000000	0.000000
KIRREL1	53.57	52.48	56.46	58.25	0.45	0.000000	0.000000
KIAA0586	7.85	8.02	3.64	3.41	-0.77	0.000006	0.000049
PCDHB5	0.28	0.24	1.05	1.12	2.44	0.000000	0.000002
WDTC1	6.86	7.87	3.88	3.94	-0.54	0.000223	0.001395
POLR3K	5.18	5.97	1.94	2.22	-1.08	0.001127	0.006085
SENP7	5.84	4.20	7.64	6.75	0.72	0.000030	0.000219
NDST3	0.14	0.15	0.42	0.39	2.05	0.002946	0.014409
CARS1	58.23	56.77	63.27	65.82	0.50	0.000000	0.000000
MARCKS	31.30	29.03	12.24	9.46	-1.14	0.000000	0.000000
ADAM33	4.45	4.13	6.91	6.33	0.96	0.000000	0.000002
CCDC146	0.85	1.22	1.71	1.35	1.14	0.009583	0.040668
ZMPSTE24	29.28	28.21	30.41	26.42	0.32	0.000769	0.004315
ACD	10.58	12.30	4.08	4.49	-1.04	0.000005	0.000038
YIPF2	39.55	38.88	33.62	38.81	0.36	0.001173	0.006313
LRRC37A3	3.02	2.73	2.55	2.96	0.64	0.005979	0.026869
CMTM7	11.11	11.93	7.52	6.35	-0.59	0.008143	0.035250
SKI	16.38	18.01	8.66	9.77	-0.51	0.000001	0.000009

AL353807.5	6.35	5.92	2.81	2.29	-0.92	0.004388	0.020505
ATP5PD	91.17	89.40	47.20	46.36	-0.52	0.000082	0.000558
ZNF142	8.16	7.51	4.53	5.44	-0.51	0.000319	0.001941
TTF2	9.58	9.02	1.89	2.33	-1.78	0.000000	0.000000
TMEM64	2.94	3.54	3.96	4.14	0.66	0.000551	0.003195
FBXO5	8.93	8.28	1.75	1.09	-2.23	0.000000	0.000000
NFE2L3	13.77	14.08	4.68	4.19	-1.22	0.000000	0.000000
CHST12	10.92	10.82	5.31	4.78	-0.45	0.004273	0.020033
EZH2	13.80	13.62	6.11	5.20	-0.95	0.000000	0.000000
COMMD4	33.39	34.65	10.71	11.18	-1.09	0.000000	0.000000
MYO10	40.73	33.45	25.84	27.01	-0.17	0.010128	0.042640
CLDN11	222.95	220.67	56.55	55.12	-1.65	0.000000	0.000000
SOD3	3.93	3.87	5.54	5.36	0.82	0.002823	0.013860
TMEM184A	1.74	1.01	0.31	0.19	-1.75	0.001473	0.007752
SLC46A3	0.39	0.35	1.78	1.24	2.73	0.000000	0.000000
VKORC1	74.53	74.69	98.90	91.17	0.71	0.000000	0.000000
FZR1	17.71	19.31	8.15	8.64	-0.60	0.000002	0.000017
PPP1R3C	7.90	8.02	12.44	11.78	0.94	0.000000	0.000000
DDX3X	83.69	80.56	54.55	52.07	-0.25	0.000350	0.002110
CYP2R1	4.11	2.88	7.94	6.51	1.02	0.000051	0.000352
RNF121	18.04	17.31	18.98	20.80	0.43	0.001185	0.006375
VGF	3.00	2.91	0.34	0.39	-2.80	0.000000	0.000000
PDE1A	0.60	0.68	1.30	1.16	1.61	0.000202	0.001271
NDUFB9	48.17	50.09	23.51	23.54	-0.71	0.000023	0.000172
C18orf54	1.41	1.50	0.37	0.57	-1.43	0.000086	0.000585
TTL	14.66	12.99	8.55	7.63	-0.40	0.000944	0.005190
ADAMTS3	1.27	1.47	0.71	0.55	-0.78	0.005912	0.026607
OCLN	0.50	0.44	1.03	0.57	1.00	0.007243	0.031744
PTEN	14.21	15.18	21.95	15.76	0.68	0.000000	0.000000
XRCC2	1.96	1.57	0.10	0.20	-3.21	0.000000	0.000000
PIK3R1	3.27	2.19	2.73	3.59	0.64	0.000321	0.001948
STK19	9.95	11.71	14.00	13.52	0.77	0.000026	0.000192
ARHGAP33	13.02	18.87	5.51	6.36	-0.91	0.000000	0.000002
RHBDD2	23.64	22.91	32.30	36.40	0.80	0.000000	0.000000
PCDHGA1	0.70	0.71	1.10	1.09	0.97	0.004559	0.021177
TJP1	17.81	16.65	11.29	9.33	-0.47	0.000002	0.000016
ORMDL3	19.10	19.96	23.90	22.39	0.60	0.000001	0.000007
ING5	12.54	12.95	5.24	9.84	-0.47	0.001714	0.008869
SART1	28.71	23.63	13.43	12.01	-0.55	0.000002	0.000014
POLDIP3	23.15	23.72	13.24	14.77	-0.39	0.000472	0.002780
TCTN3	23.47	24.24	12.90	12.34	-0.57	0.000001	0.000007
LYPD6B	0.53	0.59	0.00	0.00	-4.25	0.002924	0.014310
PLPPR2	25.19	28.06	30.23	32.95	0.60	0.000000	0.000000

TK1	45.90	52.59	2.77	3.27	-3.72	0.000000	0.000000
EIF3G	128.42	135.12	71.67	79.95	-0.45	0.000002	0.000019
GAS2L3	5.71	3.62	0.45	0.77	-2.41	0.000000	0.000000
SLC8B1	6.22	7.08	7.66	8.48	0.59	0.000502	0.002941
SESN1	3.65	3.34	7.94	5.44	1.04	0.000001	0.000009
HTR1B	0.31	0.23	0.73	0.77	1.80	0.008820	0.037771
ITPRIPL2	5.79	6.02	7.94	7.67	0.71	0.000000	0.000000
PLN	0.02	0.00	0.46	0.50	6.17	0.000124	0.000813
ACTR8	15.17	15.29	9.66	7.77	-0.42	0.009532	0.040497
HEMK1	10.17	11.76	8.67	11.74	0.45	0.000059	0.000405
C2CD5	9.07	7.16	4.35	5.64	-0.43	0.008592	0.036906
GREM2	3.42	3.69	1.37	1.13	-1.17	0.000000	0.000003
MAD2L2	17.15	18.10	6.26	4.91	-1.31	0.000000	0.000000
TMEM189	19.73	20.56	23.01	23.38	0.55	0.000001	0.000009
SRP54	31.81	32.06	23.71	18.65	-0.35	0.003663	0.017461
SAPCD1	6.91	6.61	1.58	1.28	-1.87	0.000476	0.002800
MYCT1	0.48	0.56	1.14	0.97	1.31	0.004736	0.021885
ACHE	0.20	0.36	0.73	0.91	1.82	0.006594	0.029281
AC107959.1	0.72	0.63	1.34	1.90	1.54	0.000034	0.000241
LAMC2	2.20	2.08	3.08	2.92	0.85	0.000005	0.000039
AFF3	9.67	7.59	10.97	10.63	0.57	0.000000	0.000004
LRRC1	2.91	2.91	5.40	5.77	1.13	0.000000	0.000001
COL5A1	311.86	327.09	877.34	950.58	1.95	0.000000	0.000000
CHD2	27.29	35.98	33.01	34.72	0.36	0.000004	0.000033
RIN1	30.73	33.08	8.25	9.38	-1.71	0.000000	0.000000
DOK3	7.84	6.47	10.25	10.78	0.84	0.000011	0.000087
PTGDR2	0.25	0.10	1.42	2.00	3.66	0.000000	0.000000
FZD4	1.80	2.06	2.50	2.23	0.63	0.000393	0.002346
ZNF473	3.21	3.02	1.57	1.95	-0.76	0.002394	0.011963
TCOF1	21.72	22.09	8.52	8.56	-1.08	0.000000	0.000000
PTHLH	3.20	3.57	9.00	10.03	1.76	0.000000	0.000000
ASIC3	3.18	3.35	3.54	6.18	1.26	0.000342	0.002068
NUP43	6.14	5.16	2.57	2.18	-0.86	0.000019	0.000144
MIOX	0.06	0.00	0.75	0.52	5.12	0.003692	0.017569
EHMT1	23.45	27.51	13.21	17.65	-0.36	0.001340	0.007123
IGFN1	0.16	0.18	0.02	0.02	-2.81	0.007264	0.031819
A2M	0.00	0.02	1.62	1.54	7.40	0.000000	0.000000
ACADSB	3.65	2.96	1.33	1.42	-0.99	0.000023	0.000171
FAM110A	2.12	2.38	0.37	0.26	-2.57	0.000004	0.000031
SPOUT1	14.30	15.70	6.49	5.61	-0.78	0.000000	0.000002
GCLM	9.57	8.23	3.40	2.84	-1.41	0.000000	0.000000
CHRM2	37.63	36.66	19.60	17.34	-0.67	0.000000	0.000000
DNAAF5	12.79	11.01	4.99	5.11	-0.77	0.000000	0.000004

E2F1	8.54	9.48	0.34	0.53	-4.04	0.000000	0.000000
NUP58	12.61	11.01	8.57	6.01	-0.35	0.004759	0.021976
LAMP1	195.14	189.91	212.27	197.19	0.42	0.000000	0.000000
PPP1R14A	1.17	1.85	4.81	5.31	2.04	0.001038	0.005651
MPND	3.52	3.49	0.90	1.67	-1.15	0.007330	0.032066
RALGPS2	9.53	11.63	5.83	5.16	-0.72	0.000001	0.000010
RBM19	14.08	13.89	8.27	8.66	-0.47	0.000135	0.000884
AC012651.1	0.24	0.26	0.57	0.47	1.46	0.010559	0.044235
MYO19	34.62	39.34	18.86	19.42	-0.62	0.000000	0.000000
SRC	12.02	14.14	7.31	7.80	-0.67	0.000003	0.000027
FAM111B	5.69	4.24	0.13	0.19	-4.69	0.000000	0.000000
LNPEP	3.73	3.63	4.56	3.96	0.31	0.005744	0.025954
RNPEPL1	14.85	17.30	5.51	8.88	-0.88	0.000001	0.000009
UBE2C	47.26	48.37	1.76	1.68	-4.58	0.000000	0.000000
CALCRL	0.56	0.36	0.82	1.05	1.96	0.000042	0.000294
C19orf57	1.10	0.32	0.00	0.10	-3.68	0.011028	0.045915
TMEM200C	2.26	3.42	5.12	4.96	1.30	0.000000	0.000000
BAG4	3.72	5.30	1.93	1.91	-0.71	0.001364	0.007229
AC025423.2	23.10	24.38	39.95	42.85	1.18	0.000002	0.000018
ARHGAP29	5.82	5.68	1.88	1.70	-1.25	0.000000	0.000000
S100A13	59.84	65.91	38.27	32.79	-0.41	0.005399	0.024570
SBF2	12.13	11.74	7.72	8.18	-0.38	0.001001	0.005484
ABCC10	20.71	21.78	22.61	21.90	0.49	0.000004	0.000033
CYB5RL	1.77	1.25	0.59	0.53	-1.02	0.002199	0.011102
TRAF3	6.26	5.64	3.34	2.71	-0.64	0.000001	0.000011
WDR81	5.96	7.12	3.79	5.11	-0.47	0.004384	0.020491
PHF13	7.55	7.34	4.33	4.56	-0.41	0.008863	0.037920
MAD2L1	11.15	10.33	0.90	0.51	-4.28	0.000000	0.000000
CIB1	20.67	22.96	12.40	11.74	-0.52	0.003702	0.017610
LRRC40	5.90	4.80	2.46	2.47	-0.78	0.000421	0.002500
HROB	3.15	2.56	0.24	0.36	-2.95	0.000000	0.000000
IL20RB	1.47	0.85	3.15	2.96	1.93	0.000000	0.000001
B3GAT3	34.16	36.92	32.48	41.56	0.44	0.000664	0.003781
RANBP2	16.29	16.12	11.33	9.68	-0.26	0.000888	0.004914
SRSF2	110.56	110.51	53.01	59.46	-0.61	0.000000	0.000000
CARS2	35.59	35.58	19.61	22.33	-0.47	0.000063	0.000437
ENO2	27.75	28.66	26.99	28.82	0.33	0.002599	0.012894
CDC25A	4.37	3.94	0.60	0.78	-2.38	0.000000	0.000000
NOC4L	14.24	16.04	6.63	7.59	-0.88	0.000006	0.000050
DDX51	12.55	13.87	7.49	9.70	-0.48	0.000841	0.004677
HMGB1P6	96.21	88.24	29.38	26.61	-1.36	0.000000	0.000000
ZNF436	2.56	2.77	3.38	2.98	0.58	0.003139	0.015255
HACD1	26.78	24.17	32.67	29.27	0.63	0.000426	0.002530

TMEM41A	5.10	4.86	2.83	2.56	-0.62	0.006307	0.028192
ATXN3	3.85	2.73	5.19	3.16	0.89	0.001204	0.006466
LRPPRC	23.34	20.33	14.42	11.90	-0.59	0.000000	0.000000
XPO5	17.77	16.39	11.23	9.77	-0.54	0.000001	0.000006
GEMIN5	7.45	8.01	3.47	3.39	-1.34	0.000000	0.000000
TAP2	18.16	11.35	14.06	11.62	0.37	0.000354	0.002133
LAMTOR4	35.11	36.58	19.93	17.63	-0.60	0.004466	0.020800
PTPN12	19.44	18.76	21.33	17.64	0.35	0.000989	0.005427
PRKRIP1	17.07	17.52	17.14	17.71	0.31	0.009564	0.040597
TAGLN3	1.46	1.82	0.00	0.28	-3.08	0.001574	0.008223
ZNF528	8.62	5.35	10.47	10.66	0.91	0.000000	0.000000
ACTR1B	20.86	22.43	11.72	10.64	-0.62	0.000013	0.000098
MSL3	7.13	6.84	6.54	6.73	0.50	0.001775	0.009165
NET1	7.95	7.32	2.72	4.54	-0.91	0.000001	0.000007
NUDT3	4.38	3.88	2.36	1.96	-0.60	0.000014	0.000109
SF3A2	46.47	52.62	27.41	29.04	-0.53	0.000011	0.000087
KCTD9	17.39	15.21	10.99	7.71	-0.48	0.000352	0.002122
GEMIN6	3.17	3.01	1.74	0.94	-1.11	0.006869	0.030332
FIBCD1	4.32	4.87	6.46	6.21	0.81	0.000018	0.000133
CRNKL1	5.46	6.56	4.41	3.12	-0.72	0.000214	0.001343
RAB7B	4.46	4.64	0.61	0.64	-2.41	0.000000	0.000000
METTL16	5.59	5.64	3.54	3.91	-0.46	0.009710	0.041148
TMEM260	6.62	5.47	7.80	7.40	0.69	0.000001	0.000010
HMBS	23.57	22.61	11.38	10.91	-0.77	0.000001	0.000010
UBN2	1.48	1.37	1.66	1.38	0.48	0.002160	0.010923
TRAM1	135.40	124.75	186.53	143.70	0.54	0.000000	0.000000
ARMC1	7.44	6.71	3.79	5.05	-0.52	0.005093	0.023295
MAST2	29.90	31.76	32.02	35.12	0.47	0.000000	0.000001
SFPQ	210.54	205.74	131.12	118.75	-0.43	0.000000	0.000000
MFSD6	2.47	1.86	2.74	2.93	0.74	0.001157	0.006236
MIR22HG	38.44	40.43	37.95	39.08	0.32	0.000398	0.002375
DCPS	4.74	6.70	2.13	2.34	-0.97	0.000037	0.000265
JAM3	27.89	24.96	29.31	26.73	0.41	0.000011	0.000088
GALNS	33.37	34.46	34.44	35.49	0.40	0.000023	0.000168
SYAP1	10.32	8.15	4.03	3.97	-0.75	0.000000	0.000000
CEP70	3.70	2.78	0.87	0.97	-1.32	0.000849	0.004717
ARHGAP11A	22.84	19.12	1.96	1.76	-2.83	0.000000	0.000000
AGA	19.27	16.48	22.37	21.67	0.51	0.001173	0.006313
DIMT1	9.78	9.55	4.42	4.95	-0.77	0.000913	0.005040
NPAS4	1.09	0.91	1.51	2.30	1.50	0.000086	0.000584
SNX5	46.14	45.45	26.09	25.30	-0.54	0.000000	0.000000
TNFRSF10C	1.00	1.65	4.87	4.15	2.05	0.000000	0.000000
LINC02615	1.39	1.51	3.72	3.77	1.72	0.000002	0.000020

ZNF154	2.78	2.24	4.30	4.40	1.15	0.000000	0.000000
COPS8	18.59	18.90	10.27	10.11	-0.50	0.002247	0.011307
CPSF3	16.71	18.39	9.20	8.14	-0.76	0.000000	0.000004
PPAT	5.14	5.60	1.46	1.55	-1.41	0.000000	0.000000
TNPO2	48.95	50.81	29.44	35.71	-0.28	0.001672	0.008687
ORC1	4.64	4.33	0.24	0.23	-3.90	0.000000	0.000000
TET2	3.50	3.47	4.76	4.37	0.71	0.000000	0.000000
MAPK11	7.62	8.74	8.64	9.66	0.45	0.007686	0.033499
LINC00174	1.72	2.08	2.66	2.34	0.93	0.000753	0.004234
NUP98	33.87	37.40	17.24	18.71	-0.71	0.000000	0.000000
RASSF1	15.85	15.51	7.24	6.74	-0.83	0.000001	0.000006
CCNB2	18.93	19.81	1.16	1.37	-3.56	0.000000	0.000000
BAZ2A	30.24	28.86	30.85	32.96	0.30	0.000319	0.001938
SOX12	8.34	8.14	8.67	9.31	0.46	0.000116	0.000765
SCFD2	7.30	7.46	2.87	2.45	-1.26	0.000000	0.000000
CDK13	17.19	16.36	19.82	16.71	0.34	0.000897	0.004956
NLE1	10.63	10.21	3.51	3.67	-1.04	0.000000	0.000000
CNP	31.68	32.25	17.83	18.86	-0.51	0.000002	0.000014
SNRNP40	21.69	20.03	10.01	8.80	-0.80	0.000001	0.000006
TM9SF4	53.67	54.50	52.28	52.81	0.29	0.000031	0.000224
ZNF226	7.55	10.02	8.17	7.89	0.40	0.011421	0.047344
ELOA	17.73	18.25	11.38	11.84	-0.34	0.001441	0.007596
KCND3	0.05	0.00	0.24	0.16	3.33	0.000386	0.002310
ADAM15	44.61	48.17	18.61	21.93	-0.78	0.000000	0.000000
BTBD11	0.47	0.47	1.76	1.28	1.93	0.000000	0.000000
SKIL	8.42	6.49	10.91	10.96	0.87	0.000000	0.000000
NPAS2	18.45	17.47	19.49	23.43	0.95	0.000000	0.000000
WDR74	29.25	23.65	18.05	13.58	-0.53	0.002041	0.010379
CLNS1A	39.56	33.56	21.46	17.05	-0.63	0.000301	0.001841
UBE2N	33.76	25.95	13.98	13.82	-0.75	0.000000	0.000001
CMSS1	8.32	7.57	1.79	1.45	-1.90	0.000000	0.000000
LRRC45	7.83	7.65	2.71	2.62	-1.29	0.000000	0.000000
BAG2	4.07	3.96	2.09	1.90	-0.68	0.000023	0.000167
DEPP1	1.33	1.47	3.27	2.66	1.38	0.000028	0.000203
PTPN1	14.91	15.51	8.44	8.28	-0.54	0.000002	0.000015
TMEM43	63.47	63.86	64.49	60.96	0.31	0.000006	0.000046
SHC2	0.65	0.13	0.60	0.84	2.46	0.001480	0.007779
SHMT1	4.38	4.09	0.91	0.82	-1.97	0.000000	0.000000
MBTD1	2.72	2.46	2.69	2.61	0.55	0.005270	0.024031
C5orf66	0.80	1.04	1.84	2.00	1.36	0.000681	0.003862
ERN1	2.81	2.60	4.35	4.43	1.07	0.000000	0.000000
TRAPPC2L	31.76	30.45	13.28	18.26	-0.52	0.005138	0.023482
EXOSC2	17.09	14.22	7.49	6.48	-1.04	0.000000	0.000000

TAP1	16.45	16.57	38.08	38.31	1.55	0.000000	0.000000
PTTG1	44.76	44.51	4.35	3.11	-3.22	0.000000	0.000000
IRF1	10.64	11.71	12.32	10.62	0.52	0.001700	0.008803
NCEH1	23.93	24.16	6.98	5.91	-1.71	0.000000	0.000000
DIS3L	6.27	6.51	1.67	2.04	-1.23	0.000000	0.000004
TRAIP	6.40	5.34	1.13	1.07	-2.51	0.000000	0.000000
GOT2	37.30	35.28	18.17	15.93	-0.80	0.000000	0.000000
HADH	6.74	7.86	3.47	3.52	-0.74	0.000333	0.002020
LIPA	20.33	19.70	15.19	12.13	-0.36	0.005784	0.026096
FSD1L	1.42	1.80	2.79	2.03	1.09	0.000000	0.000003
LRRC2	1.38	1.06	0.44	0.46	-0.98	0.006727	0.029780
DGAT1	15.19	13.96	13.78	15.59	0.39	0.011544	0.047780
PITHD1	12.95	11.51	7.24	6.46	-0.55	0.005364	0.024434
AGK	11.80	12.49	8.98	6.43	-0.55	0.000495	0.002905
KLF4	2.05	2.13	3.89	3.73	1.32	0.000000	0.000002
NBPF11	5.66	5.50	5.67	5.39	0.36	0.010506	0.044056
PDZRN3	8.45	10.00	9.33	12.05	0.48	0.000464	0.002737
BRCA2	3.74	1.96	0.20	0.49	-2.10	0.000000	0.000000
PHF3	7.10	6.49	3.92	3.72	-0.55	0.000010	0.000080
TYMS	42.70	40.54	7.97	6.41	-2.24	0.000000	0.000000
ABCF2	15.77	17.10	8.01	8.51	-0.86	0.000000	0.000000
FAM171A1	12.29	13.96	7.82	7.56	-0.42	0.000446	0.002641
MGST3	33.16	32.57	15.55	14.50	-0.73	0.000067	0.000457
GSTO2	0.28	0.29	0.06	0.04	-2.21	0.011814	0.048792
AP002026.1	0.96	0.65	1.85	1.79	1.65	0.000001	0.000009
DYRK1B	4.81	7.00	8.58	8.41	0.83	0.000054	0.000372
MTMR9LP	4.72	5.01	8.34	8.53	1.18	0.000000	0.000000
MFSD2A	12.62	13.66	6.10	5.54	-0.72	0.000026	0.000193
TMEM106A	5.93	5.78	8.52	7.57	0.65	0.000755	0.004240
TTC3	64.46	60.35	37.80	42.93	-0.24	0.000296	0.001815
SLC27A3	7.94	8.87	13.26	19.00	1.02	0.000000	0.000000
CLN8	5.84	5.07	10.40	9.79	1.31	0.000000	0.000000
BEST1	1.02	1.12	2.22	2.41	1.39	0.000005	0.000041
RNF114	28.31	28.07	16.97	13.71	-0.52	0.000023	0.000167
SLC40A1	0.35	0.26	1.12	1.11	2.30	0.000001	0.000005
ITPRIPL1	1.44	1.74	0.20	0.16	-2.88	0.000000	0.000003
AC010976.2	0.60	0.75	3.47	5.33	3.06	0.000000	0.000002
UTP14A	12.26	11.81	5.66	5.11	-0.81	0.000000	0.000004
IREB2	8.85	6.92	8.95	8.49	0.41	0.001547	0.008098
THUMPD3-AS1	12.13	11.16	15.62	13.88	0.80	0.000000	0.000004
DCAF4	4.40	7.74	2.70	2.72	-0.73	0.003411	0.016409
MAP3K14	8.34	8.24	3.50	3.78	-0.85	0.000000	0.000001
FAM214B	9.19	9.55	9.78	11.46	0.47	0.001197	0.006432

LARGE1	2.81	3.47	4.13	3.47	0.54	0.004580	0.021264
CEBPD	12.66	13.33	5.47	4.60	-1.03	0.000008	0.000064
THBS3	31.73	33.53	27.53	32.00	0.33	0.001707	0.008838
GJC1	15.71	15.78	8.10	6.06	-0.69	0.000000	0.000000
ADD1	87.09	85.65	63.32	65.86	-0.26	0.000280	0.001724
SYNPO2	0.85	1.13	2.83	2.68	1.84	0.000000	0.000000
GART	35.49	40.53	25.64	18.89	-0.63	0.000000	0.000000
AZIN2	3.04	3.20	2.47	4.40	0.74	0.006003	0.026957
PARD3B	2.61	2.28	3.93	3.00	0.77	0.000000	0.000004
H2AC6	2.93	2.79	7.37	8.01	1.78	0.000000	0.000000
MAT2B	14.82	15.21	8.04	7.22	-0.66	0.000018	0.000133
STIM1	11.28	12.41	14.46	14.28	0.67	0.000000	0.000000
PSMB3	80.29	85.22	42.49	38.81	-0.67	0.000000	0.000002
SPECC1L	12.64	12.70	7.06	7.31	-0.55	0.000000	0.000000
RAD23A	34.63	38.46	20.14	22.96	-0.45	0.000842	0.004680
H2BC19P	1.99	1.91	2.15	3.73	0.84	0.001973	0.010063
NUDT15	6.39	5.62	2.50	1.90	-1.11	0.000034	0.000241
GRB10	9.95	10.63	15.47	14.23	0.70	0.000000	0.000000
NIPA2	19.81	18.24	23.10	20.12	0.54	0.000003	0.000021
AL137003.1	0.89	0.63	1.53	1.72	1.44	0.000358	0.002152
H4C8	0.86	1.09	4.24	4.24	3.02	0.000000	0.000000
SNX12	18.81	17.09	8.28	7.80	-0.75	0.000004	0.000032
RCC2	24.05	24.40	22.59	22.23	0.22	0.008918	0.038135
XYLB	1.92	1.39	0.36	0.41	-1.39	0.002423	0.012101
EDIL3-DT	0.78	0.39	1.63	0.71	1.58	0.002625	0.013009
ANKLE2	45.65	46.21	52.90	49.78	0.38	0.000001	0.000011
DAAM2	1.44	1.41	1.84	1.99	0.72	0.000667	0.003792
RSAD1	14.70	15.97	7.79	8.63	-0.49	0.003648	0.017392
ZNF711	2.88	2.37	5.73	4.82	1.43	0.000000	0.000000
PDLIM3	1.19	1.20	5.76	5.15	2.45	0.000000	0.000000
SLC38A4	2.10	2.12	2.96	2.20	0.65	0.006552	0.029134
MRPS2	25.48	28.12	13.15	13.31	-0.87	0.000000	0.000003
EIF2B1	15.99	15.50	8.17	7.81	-0.68	0.000003	0.000024
CLDN4	0.97	1.22	0.12	0.12	-2.80	0.000092	0.000622
AC015813.1	9.98	9.00	10.33	10.19	0.45	0.000307	0.001874
NUP88	31.99	38.23	24.54	18.15	-0.66	0.000001	0.000009
QPCT	1.72	1.98	2.90	2.84	1.00	0.006570	0.029198
EXOSC5	6.60	6.24	1.14	1.61	-1.85	0.000012	0.000093
LRWD1	14.30	16.83	7.23	7.95	-0.70	0.000006	0.000047
OSGEPL1	2.87	2.70	1.04	0.68	-1.10	0.008645	0.037114
USB1	16.86	15.31	8.04	9.89	-0.37	0.010051	0.042353
ZNF467	0.54	0.32	0.84	0.75	1.61	0.002759	0.013580
SLC4A8	5.13	3.28	1.67	0.78	-1.71	0.000000	0.000000

CILP2	0.66	0.94	1.66	1.74	1.43	0.000003	0.000028
RHBDD3	19.01	22.13	19.55	22.83	0.47	0.004440	0.020710
ATP5MF	127.18	136.04	69.64	78.88	-0.42	0.010395	0.043631
MIEN1	10.63	13.05	6.19	5.25	-0.73	0.009026	0.038525
AGPS	5.86	6.61	7.68	6.12	0.39	0.004351	0.020367
PROCR	33.09	31.29	52.54	53.39	1.06	0.000000	0.000000
SLC7A7	1.11	1.77	4.12	5.43	2.04	0.000000	0.000000
PDXP	7.82	9.74	3.93	4.08	-0.79	0.000817	0.004553
AKNA	7.18	8.96	14.22	15.17	1.22	0.000000	0.000000
RAB31	4.90	5.69	3.34	2.67	-0.50	0.007920	0.034400
SSH3	12.76	9.33	4.47	5.61	-0.75	0.000120	0.000790
PNP	8.91	8.79	2.01	1.10	-2.28	0.000000	0.000000
ELAC2	30.65	33.83	15.41	15.80	-0.77	0.000000	0.000000
PKP2	0.39	0.13	0.52	0.61	1.57	0.011325	0.046985
TRAF5	16.16	13.81	26.81	25.81	1.10	0.000000	0.000000
TRHDE-AS1	7.37	6.54	4.01	3.26	-0.56	0.000333	0.002016
WDR11	20.67	15.37	19.20	17.28	0.35	0.001087	0.005899
NUP160	12.33	9.92	7.61	7.40	-0.40	0.000744	0.004185
HECTD4	12.54	11.80	12.63	14.47	0.25	0.008369	0.036118
LAP3	21.90	21.03	9.20	10.13	-0.83	0.000000	0.000000
ZNF117	5.78	4.88	9.12	8.19	0.95	0.000000	0.000000
NDOR1	12.74	12.98	7.15	8.04	-0.43	0.009940	0.041946
KIF1A	0.00	0.00	0.23	0.12	4.83	0.000741	0.004175
PRRX1	26.33	24.86	13.36	11.56	-0.52	0.000010	0.000081
PDIA6	333.40	320.36	339.13	307.97	0.32	0.000000	0.000001
ETFB	29.25	31.93	12.57	15.22	-0.75	0.000028	0.000203
KDM1B	3.50	2.55	1.31	1.09	-0.95	0.000676	0.003839
KDM2B	11.79	10.05	5.84	5.68	-0.49	0.000624	0.003572
SLC2A11	6.24	7.42	14.86	13.26	1.58	0.000000	0.000000
CNKSR3	6.27	6.58	2.19	2.20	-1.14	0.000000	0.000000
TONSL	8.94	9.17	1.20	1.19	-2.58	0.000000	0.000000
MYL6B	37.53	40.34	23.52	21.10	-0.58	0.000127	0.000834
NACAD	3.58	4.31	2.16	2.05	-0.77	0.000243	0.001510
INPP5D	0.13	0.32	0.38	0.62	2.04	0.004144	0.019506
ITGBL1	38.18	38.16	69.24	64.89	1.03	0.000000	0.000000
PKD1P5	1.26	0.94	2.04	2.57	0.72	0.001680	0.008722
PHAX	21.81	19.22	13.59	11.17	-0.47	0.004774	0.022041
CASP1	8.70	8.49	12.55	11.74	0.76	0.000281	0.001730
APOL1	0.62	0.39	1.15	1.20	1.47	0.004585	0.021278
POLA2	8.16	8.34	1.21	1.40	-2.66	0.000000	0.000000
CNOT7	16.75	16.62	8.15	9.83	-0.42	0.004942	0.022707
BAG3	30.01	30.14	12.46	11.81	-0.99	0.000000	0.000000
KLHDC7B	0.05	0.08	0.93	0.62	4.34	0.000000	0.000000

ST8SIA1	0.14	0.03	0.27	0.20	3.81	0.008535	0.036721
FKBP14	30.65	24.56	39.43	35.60	0.57	0.000000	0.000000
RPS25	270.26	269.89	165.09	145.61	-0.39	0.000089	0.000603
PXYLP1	5.56	5.70	2.64	3.15	-0.57	0.004823	0.022239
IRF2	4.60	4.86	4.13	2.39	-0.65	0.011618	0.048050
SNHG29	118.94	121.88	69.48	67.89	-0.41	0.000003	0.000028
UTP20	6.03	5.62	1.61	1.28	-1.68	0.000000	0.000000
PTGES2	29.70	31.30	16.57	16.76	-0.56	0.000022	0.000164
UBXN4	33.33	30.58	35.60	31.64	0.34	0.000112	0.000741
ADM2	3.45	4.24	13.99	14.34	2.22	0.000000	0.000000
WDR46	47.13	49.00	28.71	31.59	-0.45	0.000095	0.000637
LGR4	2.97	2.70	6.59	6.52	1.47	0.000000	0.000000
IK	47.85	48.51	27.78	29.27	-0.48	0.000002	0.000014
JUNB	14.28	15.32	14.79	15.68	0.38	0.007986	0.034635
ATP6V0A1	22.87	21.94	25.00	25.46	0.49	0.000001	0.000007
MGAT1	74.86	77.90	53.00	53.10	-0.22	0.003273	0.015852
HSPB11	9.74	9.37	3.86	2.88	-1.22	0.000311	0.001895
ZNF773	6.21	5.07	8.29	6.96	0.90	0.000022	0.000162
INTS6L	5.18	4.68	8.93	7.93	1.14	0.000000	0.000000
TOR4A	12.05	13.29	5.84	6.03	-0.76	0.000000	0.000000
MICAL1	61.15	62.37	71.93	78.73	0.60	0.000000	0.000000
RDX	32.41	26.60	18.40	15.38	-0.43	0.000052	0.000359
BNIP3L	16.22	16.17	18.88	17.24	0.37	0.010718	0.044806
WNT9A	0.20	0.22	0.86	0.90	2.42	0.000000	0.000004
GEMIN4	14.92	14.86	6.51	7.81	-0.51	0.000092	0.000622
DNAJC3	13.88	12.33	15.46	12.86	0.45	0.000018	0.000133
INTS14	11.43	11.21	6.50	6.04	-0.60	0.000519	0.003032
PCDHB14	0.41	0.51	2.72	1.70	2.71	0.000000	0.000000
CBLB	12.33	10.33	17.49	18.32	0.90	0.000000	0.000000
TEDC2	9.35	7.50	1.81	1.77	-1.76	0.000000	0.000000
SNHG15	11.82	11.90	5.64	5.54	-0.51	0.005903	0.026577
MIR34AHG	7.47	8.22	11.47	12.22	0.93	0.000000	0.000000
UBQLN4	16.65	18.27	12.85	12.60	-0.30	0.009022	0.038517
LRIG2	3.64	2.65	4.30	3.62	0.43	0.002236	0.011258
ATAD5	1.30	1.04	0.11	0.12	-2.99	0.000000	0.000000
ESF1	7.06	6.83	2.94	2.94	-0.83	0.000008	0.000061
ATF5	9.69	11.39	12.18	12.87	0.55	0.000557	0.003223
WFDC1	8.71	8.27	31.32	29.80	2.22	0.000000	0.000000
DMPK	52.32	56.99	53.78	63.78	0.49	0.000000	0.000003
GAL	1.34	1.39	3.26	3.65	1.69	0.003555	0.017012
AC243919.1	83.59	76.06	47.98	37.90	-0.54	0.000880	0.004875
LINC01963	4.51	4.10	1.21	1.82	-1.17	0.000008	0.000061
SMAD6	3.05	3.14	1.61	1.13	-1.12	0.000006	0.000052

ZNF471	1.49	1.31	2.58	2.16	0.99	0.000007	0.000054
GDE1	21.52	20.73	29.69	23.74	0.73	0.000000	0.000000
HNRNPA3	112.72	109.17	64.40	53.49	-0.42	0.000000	0.000000
RPUSD3	16.03	18.79	6.92	8.61	-0.76	0.000270	0.001670
CBX5	14.64	13.02	6.70	5.67	-0.93	0.000000	0.000000
MAP3K5	4.77	4.39	1.84	1.87	-0.97	0.000000	0.000001
FOXM1	22.95	26.20	1.39	1.53	-3.92	0.000000	0.000000
MRPL51	50.09	50.59	25.02	24.57	-0.75	0.000000	0.000004
MAN2B2	33.37	34.67	31.14	32.55	0.23	0.002743	0.013513
TSPAN10	1.27	1.56	0.45	0.37	-1.69	0.005154	0.023549
TMCO3	33.83	34.58	20.49	23.51	-0.32	0.000726	0.004098
IDH3A	17.65	12.92	7.24	5.99	-0.92	0.000000	0.000001
DNAJA2	20.49	20.55	11.97	11.77	-0.46	0.000152	0.000983
RABAC1	115.70	126.27	116.27	126.24	0.36	0.000522	0.003049
TSPAN4	101.87	118.62	57.90	61.53	-0.51	0.000001	0.000011
EPHA3	0.37	0.25	1.47	1.20	2.52	0.000000	0.000000
RHPN2	1.56	1.18	2.59	1.87	0.83	0.007157	0.031454
MTMR4	9.71	12.22	6.02	7.32	-0.53	0.000044	0.000306
GARS-DT	10.00	9.28	8.89	11.16	0.47	0.004514	0.021005
CDC42SE1	32.86	33.65	41.61	38.43	0.62	0.000000	0.000000
MTAP	10.39	10.11	5.97	4.28	-0.64	0.000033	0.000238
PSMC1	112.63	115.31	76.09	67.78	-0.30	0.001024	0.005591
PKN2	9.28	9.20	6.76	7.77	-0.30	0.009854	0.041641
SMU1	6.97	6.79	4.30	3.76	-0.44	0.000298	0.001820
RNF112	5.62	8.22	9.10	12.14	0.96	0.000000	0.000000
EIF2D	18.91	19.51	10.75	11.67	-0.39	0.003609	0.017249
ADAMTS12	20.36	19.42	33.44	33.28	1.15	0.000000	0.000000
TINAGL1	0.51	0.98	1.87	2.20	1.95	0.000003	0.000026
XRCC3	8.53	6.14	0.70	1.06	-2.99	0.000000	0.000000
C11orf98	32.66	31.32	14.94	12.04	-0.87	0.000086	0.000582
FCF1	16.04	18.00	9.85	9.20	-0.44	0.011958	0.049318
GPD2	7.63	8.21	2.92	2.53	-0.94	0.000000	0.000000
ADSS2	14.86	13.29	7.68	7.17	-0.63	0.000018	0.000137
G2E3	7.75	6.65	3.65	4.35	-0.48	0.006622	0.029396
PKN3	4.72	4.90	0.77	1.43	-2.31	0.000000	0.000000
CASP3	9.97	9.44	5.75	5.09	-0.42	0.010878	0.045386
KMT2C	7.50	7.00	6.91	7.38	0.32	0.000547	0.003176
BBS10	3.15	3.29	4.16	3.51	0.59	0.002967	0.014497
LEMD2	36.25	39.62	39.52	42.77	0.51	0.000000	0.000002
POLR3E	17.35	19.99	9.00	11.80	-0.41	0.005601	0.025389
HPRT1	10.69	7.83	4.59	3.85	-0.76	0.004653	0.021561
SLCO4A1	1.93	1.71	0.23	0.05	-3.63	0.000001	0.000005
MGME1	5.53	6.05	3.51	2.03	-0.84	0.001165	0.006274

PPP2R5D	22.17	21.63	10.35	12.30	-0.69	0.000000	0.000000
TXLNA	29.12	29.57	19.56	19.15	-0.26	0.001189	0.006395
ATP5PO	68.32	62.50	44.58	33.42	-0.38	0.011956	0.049318
COG6	7.43	8.40	5.20	3.84	-0.41	0.011921	0.049199
ERCC5	20.29	21.11	24.66	21.66	0.39	0.000165	0.001060
NXT1	4.86	7.07	2.22	2.08	-1.13	0.003469	0.016652
FZD6	28.29	25.43	28.23	25.15	0.36	0.000081	0.000550
RTF1	16.48	16.37	9.23	7.24	-0.30	0.010318	0.043348
IFRD1	15.27	16.61	19.16	18.49	0.58	0.000005	0.000042
ATRN	22.73	20.67	24.58	21.66	0.42	0.000000	0.000001
SMARCE1	45.06	45.73	26.38	23.71	-0.39	0.000032	0.000232
EPS15L1	13.72	14.03	7.17	7.35	-0.64	0.000011	0.000083
SLC2A9	0.70	0.90	1.64	1.83	1.67	0.003239	0.015705
PYGL	23.68	23.51	12.98	13.57	-0.57	0.000001	0.000007
SETD3	8.40	7.48	4.93	4.39	-0.46	0.009601	0.040729
TIMM50	49.48	43.44	28.55	31.22	-0.50	0.000308	0.001877
PARP2	13.78	14.40	8.30	5.89	-0.74	0.000152	0.000982
LPIN2	8.57	8.95	6.26	5.55	-0.34	0.003792	0.017983
MCCC2	7.67	7.23	3.41	3.23	-0.80	0.000011	0.000086
TMEM181	14.98	14.83	16.25	14.55	0.38	0.000029	0.000210
CACTIN	12.25	13.59	7.19	8.62	-0.41	0.005382	0.024511
EIF4A3	36.79	34.22	16.87	13.35	-0.90	0.000000	0.000000
CLEC11A	37.90	45.06	17.10	18.09	-0.90	0.000000	0.000000
RPL34	231.55	236.02	61.52	50.39	-1.60	0.000000	0.000000
FAM49A	1.20	1.04	1.72	2.23	1.40	0.000021	0.000158
YIF1A	54.97	59.06	59.51	60.00	0.47	0.000015	0.000114
HECTD3	25.97	21.98	24.22	25.02	0.35	0.000836	0.004651
TECPR2	7.56	6.20	3.34	4.81	-0.35	0.008972	0.038355
HHAT	0.91	0.98	2.27	2.31	1.76	0.000000	0.000000
SLCO2A1	1.27	1.27	3.76	4.48	2.23	0.000000	0.000000
DDR2	52.83	49.29	86.58	82.69	0.99	0.000000	0.000000
SLC33A1	11.78	11.91	16.50	16.48	0.77	0.000000	0.000000
GLUD1	26.73	24.77	17.38	14.42	-0.39	0.000214	0.001343
KIF24	1.19	1.16	0.13	0.09	-3.19	0.000000	0.000000
C14orf28	0.60	0.65	1.96	1.98	1.84	0.000002	0.000018
UBL4A	10.40	10.61	4.82	5.66	-0.62	0.000835	0.004644
GNL2	42.93	40.69	26.02	26.35	-0.66	0.000000	0.000000
RIMS3	0.08	0.01	0.13	0.13	2.78	0.003931	0.018596
SLBP	18.19	19.35	7.90	7.70	-0.99	0.000000	0.000000
NAA15	11.03	11.19	4.00	3.56	-1.36	0.000000	0.000000
FNDC1	0.19	0.18	0.35	0.28	1.35	0.011368	0.047157
CAMK1	9.54	12.37	5.53	6.28	-0.64	0.009405	0.040005
CRYZL2P	1.50	1.32	0.45	0.40	-1.20	0.010549	0.044198

MAN2A2	23.91	27.32	11.89	15.78	-0.49	0.000003	0.000027
ATP2A2	157.73	150.96	148.23	141.83	0.24	0.000032	0.000229
EPN2	10.87	13.97	5.78	8.39	-0.35	0.009987	0.042122
MALSU1	13.71	12.45	6.21	6.01	-1.02	0.001620	0.008447
HDAC3	37.54	39.34	26.34	23.57	-0.40	0.000954	0.005246
POLA1	3.99	3.37	0.80	0.69	-1.93	0.000000	0.000000
SLC5A6	30.34	30.12	12.81	13.28	-0.78	0.000000	0.000000
JKAMP	36.82	36.35	40.60	32.98	0.40	0.001848	0.009515
PLGLB2	0.89	1.18	2.05	1.67	1.14	0.001184	0.006371
GALM	4.57	4.95	2.21	2.04	-0.72	0.006910	0.030498
FLRT2	19.11	18.75	24.58	21.78	0.99	0.000000	0.000000
SLC22A17	32.01	34.22	40.00	42.23	0.62	0.000000	0.000000
TNFRSF10A	1.80	1.56	2.16	2.92	1.29	0.000138	0.000898
SSBP2	12.27	12.76	17.85	19.46	0.91	0.000000	0.000000
TPT1-AS1	20.73	19.30	26.84	26.96	0.70	0.000000	0.000000
PRMT5	46.18	42.31	35.20	23.72	-0.66	0.000000	0.000000
VAPA	54.87	51.64	30.48	24.47	-0.63	0.000001	0.000012
DAXX	42.29	39.68	19.14	18.82	-0.82	0.000000	0.000000
RAD54B	3.06	3.30	1.55	3.07	-1.30	0.000624	0.003572
GPR180	3.00	2.76	4.09	3.60	0.75	0.000000	0.000000
TRIM66	4.31	4.61	5.20	4.41	0.53	0.000060	0.000416
MDN1	14.89	12.29	4.13	5.08	-1.27	0.000000	0.000000
HCG18	6.64	6.58	7.64	7.24	0.54	0.000049	0.000343
FHOD1	43.67	47.43	14.82	17.58	-1.19	0.000000	0.000000
KDM3A	10.93	8.50	19.55	16.62	1.23	0.000000	0.000000
NISCH	62.93	70.58	61.28	69.80	0.32	0.000211	0.001326
XAB2	18.59	21.58	11.05	13.36	-0.38	0.004131	0.019451
AMPD2	86.02	86.96	45.57	52.53	-0.50	0.000000	0.000004
LRRC58	2.59	2.44	1.39	1.29	-0.57	0.001341	0.007126
CAMK1D	1.93	1.50	2.13	2.05	0.99	0.000004	0.000032
C11orf96	0.14	0.15	1.05	1.01	3.17	0.000756	0.004246
NUP155	8.19	7.35	3.87	3.36	-0.90	0.000000	0.000000
KIAA0100	37.75	43.51	24.37	25.94	-0.56	0.000000	0.000000
SLC25A11	21.67	25.78	10.06	13.61	-0.72	0.000011	0.000087
GPR155	1.59	1.34	3.89	2.79	1.28	0.000000	0.000000
ZNF90	1.17	1.09	0.23	0.23	-1.72	0.002707	0.013368
MAGEL2	0.16	0.10	0.36	0.34	1.77	0.008174	0.035373
PDP2	2.68	3.57	2.14	1.40	-0.56	0.010330	0.043380
RRBP1	321.32	322.82	317.65	338.76	0.36	0.000000	0.000000
RNASEH2C	8.55	9.06	5.83	5.68	-0.58	0.003635	0.017348
TNFSF10	0.14	0.04	1.31	0.87	3.83	0.000012	0.000093
BTN2A1	31.76	31.52	36.49	38.57	0.56	0.000000	0.000000
AL162458.1	0.41	0.36	0.54	0.61	0.92	0.005388	0.024533

XPO7	17.16	18.92	8.37	9.87	-0.70	0.000000	0.000000
NSD2	27.13	27.34	7.86	7.39	-1.71	0.000000	0.000000
MELK	13.20	12.86	2.03	2.03	-2.34	0.000000	0.000000
H2BC21	2.95	1.94	4.21	4.76	1.22	0.000009	0.000073
BAG1	8.81	9.06	4.59	3.86	-0.79	0.002713	0.013392
TCEAL3	13.66	14.37	6.01	5.76	-0.91	0.000112	0.000744
C5orf24	11.20	11.76	13.46	13.64	0.43	0.000120	0.000792
MTHFD1	36.96	34.39	10.64	8.39	-1.62	0.000000	0.000000
BCL6	15.11	15.11	19.73	21.53	0.76	0.000000	0.000000
PRR5L	1.87	1.17	4.53	3.42	1.57	0.000009	0.000071
HPS6	6.55	7.03	4.07	3.23	-0.56	0.004587	0.021278
DYNC1I2	49.54	49.56	35.06	31.17	-0.28	0.002064	0.010480
LURAP1L	4.32	4.83	5.74	5.43	0.60	0.003432	0.016506
SLC38A6	9.98	8.59	16.05	13.16	0.83	0.000028	0.000202
PNPO	4.13	3.70	1.59	2.13	-0.69	0.004842	0.022301
CDAN1	8.25	8.05	5.50	3.67	-0.74	0.000003	0.000024
NCAPD3	13.23	11.93	4.14	2.77	-2.01	0.000000	0.000000
FHL2	76.27	80.61	48.66	50.17	-0.34	0.000193	0.001222
ZBED4	4.40	4.41	2.77	2.51	-0.40	0.005554	0.025197
SNHG1	84.86	80.97	41.12	40.18	-0.66	0.000000	0.000000
DHRS7	44.06	42.17	65.47	51.75	0.79	0.000000	0.000000
FAM216A	11.95	10.84	5.40	3.40	-0.97	0.000366	0.002200
SLC6A16	1.90	1.59	3.16	2.61	1.29	0.000032	0.000233
UNC50	12.74	9.72	15.17	12.91	0.67	0.000371	0.002224
ZNF593	13.38	14.01	4.99	4.91	-1.11	0.001414	0.007469
COL22A1	0.35	0.20	1.14	0.68	1.69	0.000134	0.000874
AIMP2	17.16	16.79	8.66	8.14	-0.65	0.001118	0.006046
SLC25A20	6.05	5.56	3.19	2.54	-0.73	0.006810	0.030105
GLI1	14.50	13.29	16.25	20.67	0.81	0.000000	0.000000
WDR20	8.54	7.96	4.25	4.31	-0.58	0.002319	0.011634
GSTM4	9.17	11.47	3.58	3.92	-1.08	0.000010	0.000081
MRPL11	15.00	18.32	5.32	5.35	-1.23	0.000001	0.000008
WASH7P	18.78	22.35	24.09	29.81	0.73	0.000003	0.000026
LRP6	10.99	11.08	21.43	16.63	0.72	0.000000	0.000000
GLI3	3.67	4.26	2.00	2.21	-0.42	0.006985	0.030768
XBP1	70.52	66.77	81.25	73.81	0.51	0.000000	0.000000
RASA3	19.82	20.71	8.89	9.51	-0.80	0.000000	0.000000
MXD1	1.92	1.39	3.54	3.75	1.57	0.000000	0.000000
RELL1	2.49	2.20	3.63	2.99	0.81	0.000883	0.004890
H2BC12	9.48	17.93	23.38	24.52	1.20	0.002818	0.013842
RWDD4	5.03	5.52	2.83	2.46	-0.60	0.010709	0.044790
FBXO22	12.91	17.95	15.44	12.91	0.72	0.000000	0.000000
MCRS1	26.65	27.24	17.02	19.00	-0.33	0.011206	0.046567

NOM1	9.80	10.18	5.90	7.62	-0.38	0.008645	0.037114
TWISTNB	3.80	3.41	2.02	2.39	-0.57	0.008432	0.036351
ANKRD27	7.07	8.01	3.91	4.52	-0.57	0.000421	0.002499
DNPH1	11.30	13.62	2.94	4.28	-1.38	0.000254	0.001571
BCAR3	12.63	11.56	5.63	5.72	-0.90	0.000000	0.000000
CCDC102B	0.46	1.07	2.73	1.67	2.22	0.000034	0.000245
TM7SF3	37.25	41.24	66.29	54.97	0.93	0.000000	0.000000
ITGB3BP	6.26	5.33	1.41	1.01	-2.24	0.000002	0.000014
ETV1	5.82	4.86	5.95	5.98	0.60	0.004715	0.021806
PRKCD	4.35	4.99	1.69	2.72	-0.96	0.000438	0.002593
TNKS1BP1	104.15	115.74	92.51	102.07	0.21	0.009219	0.039252
HGH1	11.63	13.66	5.71	6.61	-0.83	0.000007	0.000054
ZWINT	31.84	29.93	1.65	1.64	-3.69	0.000000	0.000000
CUTA	111.98	117.16	46.99	45.11	-0.92	0.000000	0.000000
TSPAN18	0.37	0.31	1.12	1.25	2.61	0.000000	0.000000
VAMP1	9.16	8.71	11.65	13.99	0.76	0.000001	0.000008
CYB5D2	3.57	3.51	5.06	4.16	0.69	0.011301	0.046903
GPAM	2.89	2.74	1.64	1.54	-0.49	0.008199	0.035461
PPIL1	9.46	10.31	5.29	3.97	-0.75	0.001255	0.006717
PPP1R12B	4.07	2.16	6.61	4.59	0.68	0.000001	0.000005
ORC6	9.56	9.31	1.66	1.49	-2.36	0.000000	0.000000
AL358472.6	1.70	1.80	3.52	2.46	1.10	0.002066	0.010485
HIVEP3	2.49	2.55	1.49	1.85	-0.74	0.000293	0.001797
EMP1	78.77	60.16	31.43	27.72	-0.93	0.000000	0.000000
AF117829.1	5.34	4.40	5.56	5.65	0.61	0.002356	0.011799
PSMA3	57.68	54.86	33.60	27.24	-0.52	0.000209	0.001316
DDIT4	55.09	61.84	92.91	98.06	1.01	0.000000	0.000000
ACE	1.14	1.28	0.37	0.81	-1.03	0.008714	0.037368
EPB41L1	10.84	12.28	5.86	5.40	-0.54	0.001517	0.007950
THBS2	87.74	89.74	124.25	127.18	0.82	0.000000	0.000000
PLEKHA6	6.81	7.01	4.13	3.21	-0.52	0.000128	0.000841
MRPL9	26.42	28.10	17.29	13.32	-0.60	0.000537	0.003122
SEPHS1	26.97	23.22	13.68	11.38	-0.69	0.000000	0.000001
SHC3	3.77	3.06	2.00	1.22	-0.71	0.000012	0.000091
FBXW7	5.52	4.77	9.06	10.24	0.92	0.000000	0.000000
THOC7	17.76	16.37	9.27	6.88	-0.74	0.002569	0.012760
FAM234B	2.98	2.94	1.38	1.23	-0.81	0.001017	0.005560
DNAH1	2.11	2.55	2.90	3.24	0.79	0.000007	0.000057
DHX33	9.37	9.14	2.89	3.15	-1.30	0.000000	0.000000
AHRR	11.82	13.08	6.94	6.65	-0.63	0.000000	0.000000
PITRM1	43.29	45.71	21.52	20.55	-0.81	0.000000	0.000000
EXTL3	13.15	13.82	12.43	13.53	0.29	0.000893	0.004938
HCFC1R1	28.56	27.15	11.94	12.97	-0.83	0.000255	0.001579

PUS7L	3.75	2.46	1.67	1.22	-0.68	0.000047	0.000327
AC011498.7	1.25	1.44	0.23	0.17	-2.39	0.000184	0.001164
GAL3ST4	4.72	4.01	10.24	13.34	1.64	0.000000	0.000000
NKRF	4.47	3.96	2.20	2.06	-0.59	0.005802	0.026160
COPS7A	26.43	27.68	17.88	15.78	-0.40	0.001998	0.010181
CFAP97	6.57	5.19	2.51	2.32	-0.91	0.000000	0.000003
NT5C3B	21.52	23.47	12.61	13.05	-0.48	0.003893	0.018444
PSPC1	18.26	17.33	10.72	9.72	-0.53	0.000253	0.001566
PHLDB1	75.29	73.93	78.27	78.98	0.41	0.000000	0.000000
DCTN3	28.67	29.37	17.40	13.18	-0.55	0.002440	0.012183
AVIL	2.44	2.84	7.44	7.60	1.67	0.000000	0.000000
PDHA1	73.82	72.80	44.07	44.85	-0.38	0.001360	0.007216
DDX50	13.72	13.10	8.63	6.27	-0.47	0.003589	0.017160
EIPR1	8.56	8.21	4.00	4.37	-0.61	0.007368	0.032219
MSH6	18.78	17.97	9.05	6.54	-1.04	0.000000	0.000000
SIK1	3.19	4.56	9.51	6.25	1.81	0.000000	0.000000
NDFIP2	8.05	7.48	11.69	10.48	0.72	0.000001	0.000009
CCDC25	7.37	6.72	4.13	3.32	-0.74	0.000510	0.002980
ANO4	5.06	3.62	5.13	4.92	0.63	0.000903	0.004986
DENND2A	7.39	8.11	9.26	11.45	0.75	0.000000	0.000000
AGT	0.35	0.25	13.50	12.16	5.77	0.000000	0.000000
ARAP3	10.05	9.55	3.86	4.19	-0.92	0.000000	0.000000
PSMD13	61.92	64.85	39.98	34.32	-0.49	0.000001	0.000010
SETD5	43.20	43.31	38.31	39.02	0.22	0.004486	0.020892
COL11A2	0.78	0.71	1.33	1.18	1.12	0.000141	0.000917
ZNF654	1.99	1.74	3.04	2.74	0.84	0.000008	0.000067
FBXO43	0.31	0.40	0.04	0.02	-3.47	0.003898	0.018460
FIBP	37.83	41.01	24.14	21.66	-0.48	0.000396	0.002363
EOGT	22.21	19.10	21.23	21.48	0.33	0.001738	0.008986
PLEKHG2	60.17	58.54	63.23	69.87	0.57	0.000000	0.000000
PSMC5	66.86	69.36	41.31	38.04	-0.40	0.000056	0.000389
QTRT2	8.70	7.68	3.87	3.29	-0.96	0.000000	0.000001
C1QTNF2	1.23	1.05	0.30	0.25	-1.72	0.002634	0.013052
SOX11	2.68	2.50	1.23	1.22	-0.74	0.000014	0.000108
ZNF608	5.52	5.52	6.01	6.03	0.56	0.000150	0.000969
EIF2S2	34.02	33.09	17.84	16.03	-0.65	0.000000	0.000000
RETSAT	20.65	22.06	24.75	26.29	0.56	0.000000	0.000000
SETD2	13.65	13.40	9.51	9.37	-0.24	0.008468	0.036492
CCHCR1	9.30	10.57	4.27	4.18	-1.12	0.000000	0.000000
EMG1	13.86	12.35	6.90	6.38	-0.53	0.003416	0.016431
YTHDF2	26.84	26.53	16.56	15.37	-0.39	0.000162	0.001043
NEURL2	0.75	0.69	1.98	2.47	1.88	0.000582	0.003360
PTPRU	4.68	6.82	7.54	8.06	0.91	0.000000	0.000000

SNX19	24.86	22.92	23.00	22.04	0.23	0.003094	0.015066
CBS	67.80	71.58	93.69	102.65	0.84	0.000000	0.000000
BRCA1	4.05	4.38	0.52	1.57	-2.80	0.000000	0.000000
AC005747.1	1.63	2.77	5.26	6.19	2.01	0.000000	0.000000
DARS1	18.22	18.30	10.86	12.69	-0.40	0.002895	0.014186
USP39	20.74	21.73	11.57	10.01	-0.61	0.000021	0.000153
SMAGP	5.34	6.16	1.00	0.63	-2.18	0.000000	0.000000
AC010422.8	2.19	1.99	2.34	2.71	0.61	0.003000	0.014653
ACAT1	21.28	22.55	7.43	6.47	-1.31	0.000000	0.000000
RAC2	11.16	12.16	1.86	2.41	-2.08	0.000000	0.000000
TRA2B	73.98	72.27	42.18	35.93	-0.37	0.000046	0.000324
NICN1	5.32	5.85	9.28	8.79	0.69	0.001522	0.007977
SERTAD2	3.56	4.38	2.24	1.39	-0.87	0.000013	0.000100
EXOSC3	16.42	11.45	6.75	6.86	-0.91	0.000684	0.003883
CILP	0.06	0.06	4.43	4.46	6.58	0.000000	0.000000
TENM2	0.27	0.16	0.57	0.59	1.27	0.002756	0.013564
IP6K1	9.21	11.41	5.50	5.58	-0.53	0.000140	0.000912
CENPU	9.07	8.22	0.49	0.58	-3.75	0.000000	0.000000
SPDL1	20.71	20.00	6.30	5.22	-1.56	0.000000	0.000000
TATDN2	12.72	11.12	4.53	5.62	-0.73	0.000001	0.000006
CCDC137	16.75	24.10	9.02	9.57	-0.55	0.000282	0.001732
KANTR	2.18	2.18	2.87	2.77	0.74	0.000743	0.004182
NBAS	19.83	20.84	20.55	18.85	0.25	0.004901	0.022533
PCNT	18.83	17.72	8.59	9.78	-0.53	0.000001	0.000011
SNRNP25	14.24	15.22	6.13	6.26	-0.88	0.000105	0.000702
ASXL1	35.72	33.39	23.32	26.62	-0.22	0.006048	0.027126
PRKAB2	5.56	5.21	10.97	9.05	1.26	0.000000	0.000000
WDR76	7.12	5.17	0.39	0.56	-3.52	0.000000	0.000000
DAD1	109.42	108.56	129.44	121.67	0.56	0.000000	0.000000
NOP58	44.16	43.02	20.14	17.77	-0.87	0.000000	0.000000
CRYZL2P-SEC	0.84	0.66	1.00	1.03	0.79	0.008006	0.034704
WDR12	18.28	17.54	9.19	6.60	-1.07	0.000000	0.000000
PCK2	30.90	32.91	55.06	61.48	1.19	0.000000	0.000000
EEF1AKNMT	11.15	12.01	6.19	6.46	-0.66	0.000009	0.000071
FAM229A	2.95	2.65	4.27	3.42	0.85	0.000367	0.002200
AP3B2	0.05	0.40	0.30	0.74	3.44	0.002260	0.011359
PHRF1	17.59	18.26	10.57	11.19	-0.45	0.000016	0.000124
CAD	36.35	41.79	16.18	19.22	-0.86	0.000000	0.000000
ANKH	8.09	6.23	26.02	22.21	2.27	0.000000	0.000000
ROGDI	10.67	10.21	23.80	23.44	1.33	0.000000	0.000000
ACVR2A	3.11	2.34	4.91	3.16	0.88	0.000148	0.000960
GUCY1B1	0.97	1.04	2.41	2.05	1.52	0.000001	0.000012
CCNB1	60.24	59.23	3.62	3.31	-3.87	0.000000	0.000000

TYMP	1.72	1.89	3.46	3.70	1.17	0.004718	0.021813
TXNDC16	0.91	0.94	0.24	0.27	-1.54	0.000466	0.002748
BTN3A3	9.75	9.74	18.05	17.60	1.25	0.000000	0.000000
TIAL1	59.06	55.98	58.18	54.76	0.27	0.003255	0.015772
NCBP1	15.83	13.87	8.21	8.49	-0.54	0.000001	0.000007
MYD88	13.98	14.81	7.16	7.54	-0.57	0.000062	0.000426
PBK	11.99	10.80	0.45	0.39	-4.60	0.000000	0.000000
CLDN1	6.09	5.70	16.38	14.39	1.67	0.000000	0.000000
PLAGL2	3.94	3.64	2.13	2.15	-0.49	0.004792	0.022115
MRPL23	21.12	18.56	9.13	7.84	-0.85	0.002338	0.011717
MAB21L2	11.28	10.91	6.56	5.95	-0.49	0.001639	0.008528
AURKAIP1	41.68	42.90	20.90	22.27	-0.66	0.000026	0.000188
MSTO1	35.99	37.42	19.55	22.12	-0.51	0.000069	0.000475
RGS11	0.63	0.55	1.10	1.40	1.46	0.001665	0.008654
CLCN3	15.77	15.53	24.68	18.53	0.74	0.000000	0.000000
ABCB8	18.25	22.32	18.94	21.98	0.45	0.000352	0.002122
PFKFB4	8.64	7.48	19.23	21.97	1.68	0.000000	0.000000
AC139256.3	14.28	15.07	18.25	16.29	0.60	0.000001	0.000007
SDC3	66.06	68.81	60.02	62.51	0.19	0.004118	0.019397
CRLF3	2.33	1.99	1.04	0.82	-0.95	0.007015	0.030876
PPP1R26	2.57	3.46	1.38	1.50	-0.71	0.002225	0.011208
SND1	123.73	121.71	71.91	72.68	-0.45	0.000000	0.000000
CACNA1C	2.88	3.03	5.74	5.89	1.12	0.000000	0.000000
MBNL2	10.66	10.93	6.51	6.49	-0.38	0.003612	0.017260
THSD4	38.35	36.72	25.39	25.21	-0.26	0.000380	0.002276
APBA1	2.18	1.90	2.05	2.61	0.67	0.004520	0.021016
NOMO1	119.62	125.46	140.83	134.36	0.51	0.000000	0.000000
RAD54L	10.67	9.31	0.42	1.16	-3.80	0.000000	0.000000
PRIM1	4.45	4.08	0.62	0.38	-3.74	0.000000	0.000000
SIL1	42.89	41.01	52.25	50.65	0.65	0.000000	0.000000
ZNF667	1.90	2.21	2.76	2.01	0.84	0.003956	0.018707
ZDBF2	1.15	1.18	1.71	1.53	0.68	0.000235	0.001464
MBD1	34.93	38.37	30.48	40.21	0.33	0.000588	0.003391
ARHGEF25	10.72	11.73	12.46	13.97	0.69	0.000001	0.000011
IL11	26.45	25.64	13.30	14.24	-0.74	0.000000	0.000000
DNAJC1	18.71	16.34	32.32	28.35	1.13	0.000000	0.000000
WBP11	14.85	12.93	7.56	6.80	-0.61	0.000000	0.000002
BAMBI	9.61	9.91	12.98	10.49	0.60	0.000624	0.003572
PSMB7	74.14	76.29	47.65	42.63	-0.40	0.000404	0.002407
LMBR1L	25.04	21.18	44.62	42.56	1.27	0.000000	0.000000
ARHGAP42	1.60	1.78	1.47	1.80	0.62	0.005358	0.024410
TPBG	41.56	40.94	59.93	56.05	0.82	0.000000	0.000000
YOD1	1.19	1.27	1.58	1.38	0.61	0.008602	0.036941

WDR5	15.91	14.98	5.85	6.26	-1.01	0.000000	0.000000
C2CD2	10.64	11.39	13.93	10.98	0.61	0.000000	0.000000
SLC13A3	2.12	3.24	3.93	3.17	1.34	0.000001	0.000011
IFITM3	440.69	471.90	243.78	278.55	-0.45	0.000001	0.000006
GTSE1	15.84	16.08	0.97	0.74	-3.96	0.000000	0.000000
C18orf32	7.24	5.65	9.46	6.61	0.63	0.009725	0.041182
TMEM127	16.50	17.06	18.40	19.93	0.61	0.000000	0.000000
L3MBTL2	11.84	12.67	5.36	6.81	-0.74	0.000001	0.000013
CDC45	16.52	15.69	0.80	0.84	-4.26	0.000000	0.000000
XRCC1	13.95	12.66	6.46	7.94	-0.61	0.000231	0.001440
TANC1	7.82	6.75	8.79	8.48	0.55	0.000000	0.000003
ADK	11.27	10.56	5.73	4.63	-0.82	0.000169	0.001082
LMO4	34.45	35.12	20.91	18.40	-0.53	0.000054	0.000372
RECQL	17.68	16.53	10.16	9.14	-0.49	0.000032	0.000231
ITPRIP	22.75	23.85	15.63	16.28	-0.28	0.002134	0.010808
GGCX	32.02	36.85	35.04	35.98	0.47	0.000000	0.000001
ASL	22.40	25.61	12.86	11.63	-0.60	0.000035	0.000247
CASP8AP2	4.94	4.57	2.12	1.60	-0.88	0.000000	0.000001
BAIAP2	12.01	15.86	5.94	5.45	-0.98	0.000000	0.000001
CYP4F26P	6.90	6.68	0.99	1.52	-2.17	0.000000	0.000000
FBXO21	7.73	8.81	3.80	3.82	-0.71	0.000003	0.000028
PAFAH1B3	19.39	23.45	9.42	10.36	-0.66	0.002751	0.013551
MCAT	4.20	3.30	2.09	1.38	-1.08	0.002711	0.013385
FAM120C	2.13	1.60	1.08	1.02	-0.70	0.006357	0.028395
GPRC5A	37.89	36.34	10.74	9.80	-1.66	0.000000	0.000000
LAMA1	9.13	8.79	4.75	4.80	-0.38	0.001303	0.006945
FIGNL1	6.22	5.84	1.59	1.65	-1.55	0.000000	0.000000
CBX2	2.81	3.28	0.83	1.04	-1.45	0.000000	0.000001
MBD6	23.88	25.15	31.15	31.53	0.62	0.000000	0.000000
MTMR2	10.71	10.32	7.35	5.74	-0.42	0.001995	0.010170
C1QTNF5	5.08	5.31	5.97	6.22	0.73	0.008510	0.036647
GLI4	14.64	15.57	14.39	19.18	0.46	0.009018	0.038504
PPA1	52.85	47.20	23.62	21.94	-0.82	0.000000	0.000000
TPMT	3.68	3.63	1.54	1.15	-1.11	0.000021	0.000155
MFSD14A	13.29	13.01	18.28	15.17	0.68	0.000000	0.000000
POP7	18.77	18.98	6.80	7.34	-1.07	0.000012	0.000091
STPG1	2.41	2.23	0.93	0.77	-1.05	0.005790	0.026113
CAPN3	0.36	0.81	1.77	1.10	1.55	0.000961	0.005279
ZNF385A	3.52	3.75	5.08	5.02	0.79	0.000346	0.002091
PRDM4	10.24	10.33	5.89	6.05	-0.45	0.001027	0.005599
H2BC20P	0.94	0.66	1.61	1.37	1.23	0.000003	0.000026
NQO1	203.84	190.80	22.90	21.52	-2.82	0.000000	0.000000
SLC39A13	79.76	97.38	79.29	88.94	0.27	0.005054	0.023155

ATP5F1C	70.07	62.62	41.02	33.35	-0.55	0.000006	0.000046
RIOK2	15.03	13.99	7.77	8.46	-0.41	0.010030	0.042285
PCNX1	17.31	15.98	21.07	23.43	0.75	0.000000	0.000000
DDA1	22.48	24.18	12.67	13.59	-0.43	0.012033	0.049564
FXR1	52.74	49.81	35.62	29.73	-0.34	0.000792	0.004430
OSBPL7	9.56	10.58	5.26	5.67	-0.51	0.001544	0.008084
EIF3I	97.60	97.40	36.19	31.58	-1.28	0.000000	0.000000
CERS6	2.99	2.64	3.84	3.36	0.69	0.000005	0.000038
STOML1	10.78	10.74	12.87	13.50	0.50	0.002182	0.011028
LRPAP1	128.24	140.17	134.16	133.72	0.30	0.000159	0.001021
NOP56	165.38	162.50	72.71	71.73	-0.83	0.000000	0.000000
EDEM1	14.82	14.45	27.06	20.45	1.10	0.000000	0.000000
GAREM1	1.05	0.54	1.04	0.61	0.98	0.004039	0.019072
OPA1	20.97	17.42	11.71	11.38	-0.48	0.000033	0.000235
MEX3D	5.06	5.12	1.98	2.10	-0.98	0.000017	0.000129
ZNF767P	9.13	8.75	12.82	12.49	0.83	0.000000	0.000000
SETBP1	0.04	0.09	0.31	0.34	2.75	0.000001	0.000009
LMNB1	31.91	32.71	2.54	2.63	-3.29	0.000000	0.000000
SPECC1	12.35	12.81	15.24	14.96	0.58	0.000000	0.000004
ANKRD17	26.93	23.65	16.79	13.59	-0.25	0.001084	0.005884
PARVB	17.99	16.92	7.66	7.46	-0.78	0.000015	0.000117
PCBP2	257.14	246.39	172.57	161.78	-0.33	0.000001	0.000006
PEG10	16.16	16.25	7.05	7.24	-0.77	0.000000	0.000000
NUP50	15.07	13.72	10.50	9.30	-0.35	0.003139	0.015255
AL590617.2	2.84	2.52	14.02	13.30	2.61	0.000000	0.000000
ADAMTSL2	0.00	0.01	0.32	0.41	5.96	0.000152	0.000979
TMEM63B	24.20	24.17	36.40	35.37	0.88	0.000000	0.000000
RGS20	2.40	2.32	0.39	1.36	-2.08	0.000142	0.000919
CFLAR	9.02	9.13	13.22	11.58	0.81	0.000000	0.000000
DCTPP1	9.88	10.29	2.52	2.61	-1.64	0.000000	0.000000
GNG10	13.07	15.21	7.64	7.31	-0.58	0.007179	0.031514
AC008012.1	1.04	1.57	2.77	2.67	1.39	0.000001	0.000005
AJM1	1.68	1.76	2.25	2.68	0.86	0.000194	0.001222
HAUS6	6.06	4.80	2.14	2.24	-0.98	0.000000	0.000000
MRPL19	10.77	10.18	5.32	5.08	-0.51	0.003411	0.016409
BCL7B	13.32	14.27	7.91	7.39	-0.54	0.004830	0.022255
TIMM8A	2.80	2.49	0.87	0.86	-1.19	0.010302	0.043288
CPNE7	20.74	25.63	7.64	11.52	-1.15	0.000000	0.000000
SLC12A2	5.66	6.78	9.31	6.92	0.57	0.000009	0.000072
MMP23B	2.04	2.50	4.10	5.43	1.56	0.000200	0.001263
E2F8	1.49	1.93	0.12	0.06	-4.29	0.000000	0.000000
CLPP	38.20	33.91	21.21	23.74	-0.47	0.004037	0.019064
POLQ	3.39	3.33	0.11	0.15	-3.76	0.000000	0.000000

ACIN1	180.35	174.45	181.04	181.15	0.18	0.006497	0.028966
DNAJC18	5.81	6.24	7.84	5.68	0.74	0.000146	0.000944
DDX24	66.60	63.38	43.97	38.84	-0.30	0.000264	0.001632
LARP4B	13.82	14.59	10.32	10.95	-0.29	0.004790	0.022113
ITPK1	6.91	9.45	3.66	3.68	-0.93	0.000007	0.000059
RASA1	17.17	15.83	10.62	9.52	-0.40	0.000189	0.001196
HEATR1	10.47	9.89	5.78	5.79	-0.60	0.000000	0.000000
FAM227A	1.52	0.49	1.31	0.69	1.20	0.000035	0.000247
NECTIN1	3.11	3.89	4.19	4.83	0.68	0.000014	0.000104
TPD52L1	2.21	2.74	3.63	4.28	1.29	0.001048	0.005704
CDC20	68.19	67.29	2.16	2.14	-4.51	0.000000	0.000000
TRIM58	0.45	0.61	0.11	0.06	-2.26	0.000230	0.001437
EARS2	12.81	12.03	4.54	5.18	-0.70	0.000002	0.000020
SNRPD1	19.39	19.46	10.45	6.92	-0.90	0.000001	0.000013
THEM6	4.32	3.75	1.37	1.13	-1.39	0.000041	0.000286
CES1	0.57	0.43	4.97	3.84	3.86	0.000000	0.000000
GTF2H5	4.12	3.67	2.23	1.79	-0.75	0.010047	0.042342
VCL	236.25	225.36	229.12	223.73	0.27	0.000001	0.000010
EDA2R	6.59	5.95	11.48	10.34	1.13	0.000000	0.000000
PRDX2	94.44	107.05	44.34	52.27	-0.71	0.000000	0.000000
PAQR4	4.44	5.32	0.36	0.69	-2.94	0.000000	0.000000
NOC2L	70.27	74.70	34.69	37.19	-0.66	0.000000	0.000000
PARP1	41.26	39.01	11.43	10.57	-1.51	0.000000	0.000000
FES	8.59	6.30	2.49	2.66	-1.38	0.000001	0.000008
FARP2	9.20	10.77	4.33	5.08	-0.44	0.001687	0.008747
SF1	112.16	111.46	79.94	86.75	-0.35	0.000003	0.000027
NCAM2	3.98	2.83	5.06	3.87	0.72	0.000229	0.001426
SNRPG	32.12	32.37	12.91	10.03	-1.18	0.000003	0.000022
EGR1	24.07	24.18	13.15	14.70	-0.45	0.000034	0.000242
ENTPD7	5.03	5.11	6.04	5.57	0.53	0.000001	0.000011
SYNGR2	18.53	17.59	23.84	20.71	0.55	0.000116	0.000768
GFPT2	11.52	12.44	5.64	6.05	-0.72	0.000002	0.000019
NFIB	2.07	1.84	1.47	1.13	-0.70	0.009484	0.040300
STAG1	8.40	7.71	4.30	3.84	-0.73	0.000000	0.000001
MTA1	58.97	63.33	37.73	38.47	-0.51	0.000001	0.000006
RIMKLB	8.30	7.40	11.98	11.67	0.77	0.000000	0.000001
TMEM130	7.64	7.94	4.10	4.99	-0.59	0.003660	0.017450
FAM131A	4.80	4.45	1.57	1.68	-1.07	0.000086	0.000582
IMP3	12.16	14.10	7.26	6.71	-0.57	0.008245	0.035633
PAK1IP1	12.68	11.82	5.27	4.06	-1.06	0.000001	0.000011
GPR137	16.79	15.83	15.14	20.32	0.57	0.001337	0.007107
ATF6	9.99	9.45	12.39	10.15	0.49	0.000000	0.000002
ITGA1	73.50	57.53	154.36	137.47	1.43	0.000000	0.000000

PAPLN	1.19	0.69	1.63	2.15	1.53	0.000001	0.000009
DRAM1	12.04	11.74	12.41	11.55	0.41	0.001356	0.007200
RNF4	18.07	16.22	10.33	11.17	-0.32	0.008524	0.036682
DENND5A	35.21	34.63	22.59	24.93	-0.28	0.001682	0.008728
ATP11A	7.68	7.18	6.70	5.35	-0.35	0.003475	0.016670
SLC2A4RG	39.83	43.49	23.37	27.17	-0.57	0.000028	0.000202
RIOK1	7.55	7.29	3.45	3.28	-0.82	0.000104	0.000696
BTN2A2	7.25	6.48	8.28	7.69	0.53	0.000743	0.004181
OSTC	55.86	55.15	70.25	58.25	0.56	0.000001	0.000008
CHCHD3	20.76	20.02	13.32	12.62	-0.45	0.002808	0.013798
FH	35.39	36.71	13.26	10.61	-1.28	0.000000	0.000000
UROS	18.49	19.64	11.85	9.69	-0.53	0.001108	0.005997
PSMD1	56.70	58.32	40.77	36.58	-0.45	0.000000	0.000003
BAHCC1	9.32	10.21	7.46	8.73	-0.41	0.000640	0.003652
CBX1	28.71	27.78	14.86	14.41	-0.72	0.000000	0.000000
GTPBP6	19.91	22.24	10.24	11.94	-0.57	0.000101	0.000674
ELP2	41.85	37.26	27.65	23.33	-0.38	0.000483	0.002837
CTH	3.50	3.55	7.07	7.07	1.36	0.000000	0.000000
PLEKHA4	14.67	15.53	7.63	8.26	-0.90	0.000000	0.000000
WRNIP1	17.48	17.89	10.75	10.12	-0.38	0.009927	0.041896
SPAG7	67.71	67.73	91.56	101.56	0.90	0.000000	0.000000
ZNF532	25.49	24.79	25.34	25.01	0.47	0.000000	0.000000
GPN1	14.37	12.16	5.92	6.72	-0.75	0.000034	0.000242
PRMT7	29.43	31.60	18.42	21.89	-0.49	0.001083	0.005879
TTC39B	2.52	2.29	7.07	4.90	1.56	0.000000	0.000000
ABCF2	16.10	14.98	8.27	6.75	-0.71	0.000005	0.000037
AMT	3.28	4.26	7.68	7.12	1.19	0.000000	0.000001
PCDHGB4	9.06	9.80	11.53	10.66	0.56	0.000000	0.000004
TGFBRAP1	4.12	3.85	2.11	1.99	-0.57	0.001333	0.007090
CRYAB	3.23	5.40	0.95	1.00	-1.97	0.000275	0.001692
WDR4	9.25	8.98	4.39	3.65	-1.01	0.000005	0.000044
ITGB1BP1	35.09	31.19	23.60	19.26	-0.45	0.002893	0.014182
PCDHGA4	3.85	3.00	6.28	6.15	1.35	0.000000	0.000000
FLCN	28.05	27.92	33.16	33.63	0.55	0.000000	0.000000
EXO1	4.95	5.06	0.43	0.24	-3.94	0.000000	0.000000
PEPD	19.34	19.00	10.56	10.10	-0.56	0.000093	0.000624
CES2	16.40	16.46	18.27	18.04	0.48	0.000056	0.000389
DDX31	6.81	7.12	3.91	3.69	-0.55	0.004069	0.019191
SERPINH1	1,241.99	1,320.37	1,721.83	1,757.27	0.78	0.000000	0.000000
UBALD1	12.05	13.83	13.17	15.34	0.56	0.000669	0.003802
RUFY3	18.04	16.61	24.01	19.17	0.71	0.000000	0.000000
ZNF274	7.26	7.99	9.91	10.50	0.72	0.000000	0.000003
DNAJB12	15.05	16.84	15.90	16.55	0.37	0.001903	0.009762

PRICKLE2	5.15	4.65	6.78	7.82	0.62	0.000000	0.000001
ALYREF	48.67	48.76	18.91	20.02	-1.01	0.000000	0.000000
LMF1	7.40	8.46	12.89	11.90	0.99	0.000000	0.000000
KRTAP1-5	1.95	2.11	0.19	0.33	-2.62	0.000395	0.002359
BRWD1	3.24	3.42	4.25	4.06	0.65	0.000000	0.000001
ETHE1	21.75	23.19	6.63	6.40	-1.46	0.000000	0.000000
RBBP8	6.29	6.60	2.38	1.70	-1.29	0.000000	0.000000
KIF23	30.02	30.60	6.07	7.16	-1.99	0.000000	0.000000
PPM1F	17.16	17.29	8.07	10.71	-0.52	0.000010	0.000080
STIL	4.22	4.22	0.81	0.72	-2.21	0.000000	0.000000
RNF217	6.71	6.52	11.45	10.89	0.92	0.000000	0.000000
PLAUR	111.57	110.57	67.71	71.34	-0.31	0.000272	0.001676
TRIM38	2.42	2.21	3.12	3.09	0.76	0.000000	0.000001
MFN2	27.46	27.80	14.63	14.90	-0.52	0.000000	0.000000
ARHGEF10	19.18	21.87	11.87	17.61	-0.30	0.002528	0.012577
EIF2B2	20.26	18.49	11.14	12.02	-0.49	0.006018	0.027010
RAB35	17.55	20.98	10.23	11.46	-0.49	0.000145	0.000941
TMEM45A	13.87	11.68	40.40	35.67	1.95	0.000000	0.000000
PLK4	9.29	8.91	0.82	0.77	-3.78	0.000000	0.000000
ADIPOR2	15.71	14.89	19.05	16.44	0.51	0.000000	0.000004
PI4K2B	4.06	3.38	1.83	1.34	-1.03	0.000019	0.000143
NDUFAB1	37.48	37.79	21.63	18.07	-0.58	0.003294	0.015930
SLC49A4	4.96	3.80	7.58	5.83	0.79	0.000087	0.000588
FBF1	8.80	10.14	3.65	6.14	-0.64	0.000658	0.003745
ANXA7	31.13	35.32	19.28	17.76	-0.48	0.000127	0.000831
TUB	4.15	4.68	1.83	1.88	-0.94	0.000000	0.000000
ADIRF-AS1	3.97	3.71	1.53	1.61	-1.08	0.000163	0.001046
LRRK1	3.57	3.53	1.54	1.73	-0.63	0.000325	0.001975
NSA2	27.37	25.58	13.77	11.87	-0.67	0.000026	0.000188
NPM3	17.12	18.40	8.59	10.49	-0.66	0.005966	0.026819
MRPL24	34.74	36.94	18.88	19.05	-0.57	0.000614	0.003526
ISLR	1.18	1.47	12.03	14.00	3.59	0.000000	0.000000
CANT1	29.82	29.27	20.43	19.67	-0.24	0.011607	0.048013
DYNC1LI2	52.49	53.60	74.46	71.94	0.67	0.000000	0.000000
NEURL4	28.73	32.30	15.91	18.91	-0.35	0.004215	0.019790
PID1	0.77	1.11	2.18	1.83	1.21	0.001556	0.008141
KLHL5	21.47	22.54	25.86	25.60	0.44	0.000004	0.000036
CHUK	7.32	6.50	4.93	3.15	-0.46	0.011269	0.046807
CDCA8	15.24	15.56	0.50	0.56	-4.51	0.000000	0.000000
KEAP1	22.20	21.90	9.31	11.76	-0.58	0.000008	0.000065
DSN1	6.62	6.06	1.83	1.88	-1.55	0.000000	0.000000
PLXNA2	0.44	0.41	0.82	0.75	1.29	0.000001	0.000011
HAPLN1	0.61	0.62	5.07	4.07	3.40	0.000000	0.000000

SNRPB2	27.21	26.92	14.44	10.35	-0.82	0.000005	0.000041
RRP1	11.73	14.46	6.08	7.07	-0.91	0.000000	0.000000
CBR1	40.73	41.83	19.89	17.92	-0.68	0.000001	0.000012
DLAT	9.35	9.07	4.85	3.59	-0.81	0.000001	0.000005
CENPJ	7.15	8.82	2.92	4.11	-1.17	0.000000	0.000000
HNMT	3.46	3.58	8.04	6.07	1.24	0.000005	0.000041
AC102945.2	8.09	7.19	9.59	10.70	0.75	0.000312	0.001900
CEP97	2.00	1.94	1.00	1.18	-0.62	0.010642	0.044541
MPP4	3.36	3.53	0.87	0.62	-1.98	0.000000	0.000000
CHST2	0.78	0.87	2.16	2.05	1.69	0.000000	0.000000
MAGED2	166.38	182.10	173.16	173.94	0.32	0.000005	0.000042
DDX12P	3.00	2.73	0.17	0.23	-3.49	0.000000	0.000000
ADPGK	52.37	47.75	49.38	44.88	0.28	0.000605	0.003478
WDR90	28.82	25.67	14.45	17.63	-0.68	0.000001	0.000012
RACGAP1	24.82	26.13	1.85	2.44	-3.10	0.000000	0.000000
SPATA13	7.46	7.68	3.56	5.43	-0.44	0.001415	0.007469
RPE	15.09	15.94	9.07	10.17	-0.41	0.009090	0.038749
METTL26	31.60	33.28	14.19	15.30	-0.77	0.000006	0.000048
SLC39A7	219.72	226.42	232.18	236.91	0.44	0.000000	0.000000
NDUFS2	40.24	41.53	29.39	29.20	-0.34	0.001406	0.007432
WDFY1	16.83	17.17	11.19	11.69	-0.37	0.001061	0.005769
AFG3L2	18.45	18.01	10.98	8.99	-0.58	0.000002	0.000018
RPS21	269.54	289.80	150.38	141.74	-0.52	0.000047	0.000329
PTBP1	114.03	131.48	70.01	73.66	-0.61	0.000000	0.000000
GUCD1	20.16	24.05	10.49	10.41	-0.61	0.000001	0.000011
AATF	23.35	21.76	10.95	10.19	-0.73	0.000000	0.000000
GRAMD1A	40.74	43.84	37.76	44.88	0.42	0.000104	0.000692
TMEM185A	10.68	10.55	11.64	9.69	0.67	0.000007	0.000054
ZFP36L1	37.37	36.77	31.93	35.54	0.25	0.005836	0.026300
YIPF6	7.99	7.61	8.26	5.79	0.60	0.000010	0.000078
MLEC	58.27	55.13	45.81	48.24	-0.21	0.003680	0.017524
NABP2	11.13	12.39	4.54	5.05	-0.95	0.000017	0.000129
CELSR1	0.65	0.65	3.74	2.50	2.42	0.000000	0.000000
CDH24	15.93	15.68	7.42	8.56	-0.66	0.000001	0.000008
LOXL1-AS1	7.21	6.87	2.13	3.04	-0.92	0.000728	0.004107
MED27	14.58	14.91	6.24	6.40	-0.73	0.000365	0.002191
MBTPS1	43.86	42.51	42.56	43.28	0.30	0.000046	0.000325
FAM156A	22.89	27.48	23.50	23.02	0.30	0.002348	0.011767
PUSL1	10.01	12.75	4.73	5.77	-0.71	0.004537	0.021083
LRRC8A	21.81	22.27	28.32	30.08	0.74	0.000000	0.000000
BTBD19	11.70	11.94	15.81	18.83	1.05	0.000000	0.000000
PHACTR4	11.84	13.27	7.90	7.33	-0.38	0.002921	0.014300
SRSF3	137.57	132.35	73.97	65.08	-0.38	0.000006	0.000050

CBX4	7.60	7.40	8.54	8.54	0.48	0.004283	0.020061
PTGER4	3.87	5.13	4.79	5.77	0.60	0.001444	0.007614
NAGLU	30.84	31.47	28.24	32.95	0.35	0.000957	0.005259
TARDBP	76.35	74.17	47.39	43.67	-0.26	0.001248	0.006692
GFM1	9.53	9.69	6.46	5.16	-0.43	0.002259	0.011356
HIC1	9.05	8.59	9.81	8.78	0.46	0.000319	0.001942
LINC00662	4.58	5.12	10.29	10.04	1.41	0.000000	0.000000
TMEM178A	0.91	0.73	1.65	2.11	1.45	0.002691	0.013298
GLIPR1	95.89	85.98	103.48	80.73	0.24	0.004573	0.021235
MSH2	8.86	8.50	2.02	1.51	-1.96	0.000000	0.000000
C5	1.26	0.99	0.25	0.26	-1.19	0.005085	0.023265
MAPKAPK3	10.01	10.87	3.18	3.95	-1.14	0.000000	0.000000
DPYSL4	11.91	14.26	16.99	20.53	0.77	0.000000	0.000000
LEO1	13.18	14.35	8.23	7.25	-0.55	0.000523	0.003056
DDB1	109.20	110.54	65.22	64.65	-0.56	0.000000	0.000000
STAT3	34.90	32.04	34.23	31.41	0.23	0.003962	0.018728
NUP214	11.68	13.87	6.59	5.86	-0.72	0.000000	0.000000
DOT1L	9.84	9.88	5.70	5.73	-0.49	0.000010	0.000076
TCF3	56.97	57.62	36.73	39.64	-0.40	0.000035	0.000248
OAT	37.03	34.60	19.02	16.04	-0.75	0.000000	0.000000
MYOSLID	0.56	0.74	1.78	1.17	1.68	0.003623	0.017294
RRM2	52.96	48.76	2.04	2.63	-4.62	0.000000	0.000000
ATAD1	17.48	16.87	21.61	17.45	0.51	0.000006	0.000050
TBRG4	33.09	33.68	16.69	21.15	-0.55	0.000297	0.001816
MYO15B	0.78	0.59	1.02	1.72	1.40	0.010582	0.044319
DDX46	24.95	21.53	15.84	17.55	-0.39	0.000103	0.000684
TPX2	37.45	35.49	2.79	2.76	-3.38	0.000000	0.000000
PUS1	19.52	17.19	10.27	11.44	-0.51	0.001675	0.008699
AL365203.2	11.54	11.30	6.56	6.43	-0.48	0.005078	0.023246
AC112128.1	0.52	0.54	0.90	0.75	0.98	0.001572	0.008216
C4B	4.45	4.89	17.67	17.86	3.03	0.000000	0.000000
PPIEL	7.78	8.91	9.74	11.37	0.53	0.010959	0.045685
NUP205	21.27	21.54	9.09	7.80	-0.94	0.000000	0.000000
RTTN	2.81	3.21	0.91	1.41	-1.15	0.000000	0.000002
HNRNPUL2	25.51	24.82	13.43	13.83	-0.64	0.000000	0.000000
HERPUD1	58.83	59.73	81.18	77.60	0.71	0.000000	0.000000
CTTNBP2NL	10.01	9.32	3.95	4.31	-0.83	0.000000	0.000000
CDK5RAP1	21.04	20.69	14.17	11.99	-0.47	0.003264	0.015811
LIG1	23.93	21.44	7.84	5.52	-1.46	0.000000	0.000000
ATP5F1D	42.12	44.79	21.25	23.43	-0.63	0.000175	0.001113
TOR2A	6.32	6.33	6.68	9.77	0.60	0.007308	0.031979
TOMM22	14.79	14.91	6.70	6.56	-1.06	0.000000	0.000000
KIAA1755	4.31	4.72	7.49	8.55	1.41	0.000000	0.000000

FANCM	1.83	1.18	0.90	0.96	-1.21	0.000164	0.001050
MTBP	2.16	1.85	0.57	0.32	-2.04	0.000000	0.000003
RBM10	25.06	26.92	16.19	18.12	-0.29	0.007635	0.033302
RAD51AP1	5.02	5.00	0.43	0.27	-4.24	0.000000	0.000000
ADCY7	11.61	13.73	4.88	5.25	-0.87	0.000000	0.000000
KIF18A	8.51	8.19	1.24	1.14	-2.53	0.000000	0.000000
RBM5	107.11	104.01	97.55	89.53	0.30	0.000019	0.000141
USP46	12.39	9.49	12.62	10.06	0.40	0.001022	0.005580
SERINC3	45.13	45.48	69.76	62.22	0.90	0.000000	0.000000
PARP6	34.48	34.89	35.12	37.22	0.48	0.000004	0.000034
NAA20	23.07	22.81	13.75	11.72	-0.47	0.010849	0.045281
SERINC5	1.70	1.80	3.55	3.43	1.37	0.000000	0.000000
CAMKK1	4.86	3.49	1.82	2.14	-0.74	0.002132	0.010799
PIK3R4	11.15	11.86	7.14	5.80	-0.53	0.000301	0.001838
TSPAN31	10.27	9.63	16.42	11.23	0.61	0.002431	0.012138
NUP133	9.15	7.34	5.21	4.59	-0.44	0.001055	0.005739
NFAT5	10.34	8.68	10.76	12.02	0.59	0.000000	0.000000
SETD6	6.71	5.25	2.64	2.61	-0.96	0.001817	0.009366
VOPP1	32.97	32.83	18.54	19.21	-0.52	0.000000	0.000000
TMEM9B-AS1	0.33	0.31	0.59	1.06	1.53	0.001583	0.008265
TWIST2	6.48	6.21	2.94	2.65	-0.84	0.005674	0.025688
ST8SIA4	0.08	0.11	0.32	0.30	2.28	0.000489	0.002873
LRP11	24.90	24.14	15.51	14.25	-0.32	0.001559	0.008157
CCDC77	6.35	5.67	1.97	2.05	-1.87	0.000000	0.000000
AC025165.6	0.34	0.35	0.95	1.33	2.39	0.000005	0.000041
KCTD16	0.05	0.11	0.26	0.21	1.86	0.000101	0.000676
SLC35F5	29.06	24.85	36.52	30.85	0.70	0.000000	0.000000
PLEKHO1	34.54	43.50	32.68	36.03	0.28	0.009007	0.038480
GNL1	31.84	32.59	21.60	22.20	-0.30	0.008518	0.036665
PHF20	7.65	7.38	2.21	2.49	-1.26	0.000000	0.000000
FTO	19.57	17.06	9.48	8.80	-0.64	0.000022	0.000165
EEF2KMT	4.32	4.73	2.14	1.48	-1.18	0.000153	0.000987
GLRX3	31.30	25.68	13.90	14.86	-0.60	0.000037	0.000263
SETD7	9.82	8.65	4.50	4.29	-0.68	0.000000	0.000000
HIF1A-AS3	0.07	0.21	0.60	1.03	3.68	0.000498	0.002916
AC002316.1	5.69	5.87	1.41	1.28	-1.81	0.000000	0.000000
FBLIM1	44.34	45.74	56.66	64.39	0.60	0.000000	0.000000
GNL3	74.74	69.41	38.12	35.41	-0.72	0.000000	0.000000
SH3D21	9.23	10.24	15.72	17.56	0.97	0.000000	0.000000
G6PD	179.05	195.35	48.61	54.08	-1.58	0.000000	0.000000
SARM1	6.29	6.37	8.34	6.79	0.53	0.000059	0.000409
HNRNPD	157.38	160.45	88.29	77.00	-0.63	0.000000	0.000000
PHF21A	13.50	10.27	13.99	15.06	0.69	0.000000	0.000000

VASN	100.05	110.34	106.04	118.55	0.43	0.000000	0.000002
NPHP3	10.08	9.02	10.38	8.53	0.38	0.002383	0.011916
SMAP2	17.97	17.52	9.38	8.77	-0.67	0.000000	0.000002
RARS1	60.36	56.13	33.67	27.37	-0.64	0.000000	0.000000
DDX47	28.99	29.21	14.84	13.50	-0.70	0.000000	0.000000
MAN1C1	0.88	0.65	1.58	2.09	0.96	0.004851	0.022322
HSD17B2	0.00	0.16	0.62	0.39	3.47	0.005719	0.025858
SLC30A7	5.58	5.24	7.20	6.26	0.69	0.000000	0.000000
NDRG4	8.62	10.03	4.02	5.05	-0.71	0.001715	0.008872
GLT8D1	57.21	52.54	58.17	56.37	0.34	0.000599	0.003451
BX255925.3	14.41	14.50	6.63	8.71	-0.57	0.001438	0.007584
LY6G5B	17.87	16.68	16.27	18.48	0.41	0.006832	0.030187
FXYD5	101.40	100.40	58.27	60.17	-0.42	0.000078	0.000528
ATP2A3	0.73	1.14	3.69	4.30	2.59	0.000000	0.000000
XYLT2	28.91	30.28	27.50	31.35	0.35	0.000273	0.001681
CNDP2	36.65	44.60	20.98	21.47	-0.57	0.000000	0.000003
LZTS2	36.76	42.44	33.34	40.61	0.31	0.002900	0.014207
SPRY4	6.58	6.19	9.25	9.84	0.82	0.000000	0.000000
TOMM70	15.53	15.51	9.57	9.10	-0.43	0.000125	0.000821
ERP29	116.88	123.05	133.41	130.32	0.48	0.000000	0.000000
GIT2	21.40	18.87	12.41	11.70	-0.51	0.000000	0.000004
NEU1	23.53	25.68	29.30	27.78	0.60	0.000000	0.000000
BTN3A2	8.40	8.80	8.97	8.86	0.55	0.000292	0.001789
DNMBP	10.69	10.13	6.79	6.14	-0.39	0.000796	0.004449
ERVMER34-1	0.04	0.06	0.28	0.28	2.93	0.006144	0.027515
MCAM	148.38	151.59	144.73	141.25	0.28	0.000025	0.000183
PRRT3	4.65	4.87	2.01	2.72	-0.85	0.000320	0.001944
DMXL2	11.55	11.57	11.45	9.82	0.36	0.001825	0.009405
OSTM1	11.47	9.12	16.32	15.44	0.78	0.000000	0.000000
TBL3	17.00	17.94	6.69	8.33	-0.76	0.000000	0.000000
TTI1	7.61	6.23	4.60	3.35	-0.60	0.000384	0.002296
SKA2	18.14	16.06	9.33	8.75	-0.63	0.001654	0.008602
GNPTG	27.71	26.25	26.91	29.01	0.35	0.005760	0.026010
MRPL15	31.75	31.08	14.74	11.96	-0.95	0.000000	0.000004
ATP6AP1	90.97	98.78	115.14	123.70	0.69	0.000000	0.000000
AL365205.1	2.20	2.10	2.78	3.33	0.84	0.000639	0.003647
PAK4	13.20	15.42	4.61	6.96	-1.10	0.000000	0.000000
IARS2	27.99	26.79	15.36	13.17	-0.58	0.000000	0.000000
PHLDB2	9.05	8.24	11.39	8.88	0.50	0.000034	0.000246
RCN1	264.02	244.93	298.57	264.96	0.48	0.000000	0.000000
GTF2IRD2	3.66	3.60	5.44	3.87	0.85	0.000503	0.002947
NCAPG	13.58	13.89	0.64	0.96	-4.14	0.000000	0.000000
NDUFB10	51.18	49.84	25.74	22.32	-0.78	0.000010	0.000078

MAPK10	0.93	1.25	4.69	3.73	2.03	0.000000	0.000000
CBFA2T2	3.47	3.38	4.34	4.85	0.72	0.000000	0.000004
SLC25A25-AS1	0.86	1.09	1.28	1.17	0.67	0.009944	0.041955
DDX1	46.59	45.53	29.71	25.90	-0.43	0.000006	0.000047
SLC22A18	7.67	8.99	8.82	10.35	0.57	0.009824	0.041549
PGAM5	9.07	10.65	3.80	3.56	-1.24	0.000000	0.000000
CASC15	1.00	0.89	4.21	3.67	2.35	0.000000	0.000000
TBL1XR1	17.60	14.90	12.11	10.75	-0.34	0.002074	0.010526
AP1M1	34.42	34.39	18.42	19.52	-0.52	0.000006	0.000049
RNF26	19.77	20.72	9.59	10.54	-0.67	0.000000	0.000001
PSMG1	26.96	24.71	16.75	12.00	-0.58	0.005996	0.026933
RGPD5	4.04	4.72	3.15	4.72	1.88	0.000132	0.000865
TIE1	2.49	1.23	0.39	0.15	-2.36	0.000002	0.000016
ADGRA2	43.50	44.23	75.82	79.77	1.16	0.000000	0.000000
PGD	141.91	139.85	77.84	76.06	-0.54	0.000000	0.000000
UTP25	2.51	2.37	1.10	1.20	-0.85	0.000010	0.000076
TBC1D31	2.07	2.13	0.82	0.79	-1.12	0.001153	0.006215
SMG1P1	9.23	7.38	8.90	12.09	0.73	0.000101	0.000677
CTU2	13.81	14.64	6.21	7.12	-0.82	0.000041	0.000289
FAM86C1	2.82	2.98	1.33	0.85	-1.20	0.001775	0.009165
POLD1	25.79	32.65	5.24	6.07	-2.12	0.000000	0.000000
EME2	14.36	12.67	13.27	15.68	0.40	0.000276	0.001700
RAMP1	17.89	18.10	48.79	47.30	1.77	0.000000	0.000000
LTBR	73.68	78.09	71.85	78.14	0.36	0.000026	0.000193
REV3L	6.74	5.50	6.77	7.61	0.44	0.000113	0.000748
CDC123	40.08	41.40	25.79	21.73	-0.38	0.002566	0.012751
DPYD	5.41	5.19	1.90	1.88	-1.17	0.000000	0.000000
PHB2	92.52	95.86	46.57	52.45	-0.66	0.000000	0.000000
LRBA	6.77	6.95	4.59	4.25	-0.44	0.002232	0.011243
TM6SF1	1.84	2.31	8.67	6.48	2.26	0.000000	0.000000
POLD3	8.21	7.49	2.21	1.40	-1.88	0.000000	0.000000
POMGNT1	35.83	37.22	20.41	21.74	-0.38	0.000404	0.002410
SMCHD1	17.58	15.68	11.35	9.40	-0.49	0.000002	0.000013
ZC3H15	30.97	29.74	17.06	16.82	-0.61	0.000000	0.000004
ARHGAP11B	5.54	3.64	1.76	0.86	-1.47	0.000016	0.000119
TSC22D3	4.51	3.31	17.65	16.77	2.47	0.000000	0.000000
SF3B4	57.72	58.64	28.63	30.34	-0.64	0.000000	0.000000
RPL23AP82	11.28	12.97	15.73	15.56	0.84	0.000056	0.000385
PSMA2	40.95	41.88	20.73	17.52	-0.59	0.000005	0.000045
BCKDK	18.70	22.14	10.58	12.07	-0.43	0.004828	0.022250
CHTF8	23.40	25.15	14.14	13.61	-0.49	0.000222	0.001389
LAMB3	1.42	1.49	0.28	0.47	-1.66	0.001003	0.005493
SPRED3	4.54	4.30	5.36	4.35	0.43	0.010118	0.042603

MYH10	44.32	46.29	47.23	51.42	0.44	0.000000	0.000000
RAB10	35.52	33.58	23.64	18.64	-0.33	0.004232	0.019864
TMEM11	12.79	12.87	6.18	6.00	-0.71	0.000996	0.005462
SH2D4A	12.43	12.68	7.72	6.73	-0.52	0.000786	0.004399
UBE2J2	29.84	29.37	12.42	14.43	-0.67	0.000007	0.000054
D2HGDH	14.75	16.42	8.19	7.72	-0.58	0.000031	0.000224
SLC16A1	37.97	35.58	46.47	43.04	0.56	0.000000	0.000000
SNCAIP	6.14	5.22	7.63	7.39	0.61	0.000446	0.002643
GTF2IP4	29.65	31.21	17.34	18.94	-0.41	0.000163	0.001048
LNCOG	0.85	1.58	2.43	3.25	1.45	0.001128	0.006088
EMD	39.20	39.60	25.81	24.26	-0.38	0.005987	0.026899
VLDLR-AS1	0.25	0.44	2.28	1.79	3.33	0.000000	0.000000
POLR2F	29.13	25.75	12.42	12.01	-0.87	0.000362	0.002178
SLC36A4	7.14	6.37	3.26	3.11	-0.59	0.002473	0.012318
ARHGEF2	79.70	83.15	81.74	83.01	0.47	0.000000	0.000000
TTC38	13.62	15.16	7.80	7.12	-0.65	0.000018	0.000136
TBC1D1	11.79	14.06	4.85	5.03	-1.24	0.000000	0.000000
NKTR	33.85	32.70	34.94	30.74	0.19	0.008429	0.036343
TAGLN2	205.64	212.39	88.80	87.70	-0.86	0.000000	0.000000
EML3	19.74	19.76	10.36	11.89	-0.65	0.000007	0.000053
ENPP2	17.89	17.46	4.65	4.44	-1.60	0.000000	0.000000
PSMD14	40.40	39.67	28.20	22.45	-0.32	0.008441	0.036383
SUPT5H	80.66	89.44	53.20	58.53	-0.28	0.001878	0.009649
TRIM25	19.07	19.12	7.86	9.04	-0.72	0.000000	0.000000
NFE2L1	141.06	123.36	256.69	233.92	1.12	0.000000	0.000000
NRK	2.77	2.26	0.91	0.78	-1.22	0.000000	0.000000
ZBED1	6.70	9.04	2.95	2.87	-0.92	0.000000	0.000000
ANKRD18B	5.27	4.53	1.16	1.25	-1.61	0.000000	0.000003
PRKACA	31.21	28.87	19.17	17.54	-0.38	0.000654	0.003722
CLU	733.44	724.37	798.79	761.62	0.44	0.000000	0.000000
VASH2	1.43	1.54	2.33	2.14	1.30	0.000008	0.000067
PI4KA	24.61	24.30	11.94	14.56	-0.60	0.000000	0.000000
LRRC37A4P	8.96	7.64	3.03	3.18	-1.15	0.000000	0.000000
MDH1	60.85	60.21	35.67	30.83	-0.54	0.000001	0.000011
EBPL	27.01	19.89	7.02	5.21	-1.55	0.000000	0.000000
PIK3CB	3.07	2.65	1.35	1.44	-0.70	0.001804	0.009305
ADGRG6	16.29	14.48	7.27	6.35	-0.88	0.000000	0.000000
ZNF584	5.62	5.71	2.32	3.05	-0.84	0.001016	0.005558
GDF5	24.82	24.91	4.51	5.23	-2.01	0.000000	0.000000
ALDH1A3	1.82	1.64	5.07	4.15	1.87	0.000000	0.000000
ADIPOR1	17.25	17.32	17.55	17.20	0.36	0.004628	0.021465
FBXO32	0.90	0.48	1.88	1.43	2.05	0.000000	0.000001
C1GALT1C1	13.99	12.52	7.58	7.40	-0.48	0.007665	0.033415

ILF3	135.04	132.37	72.34	70.86	-0.61	0.000000	0.000000
STAMBPL1	11.62	12.52	5.72	5.72	-0.74	0.000033	0.000237
PFDN2	45.13	48.42	22.88	22.57	-0.68	0.000389	0.002324
PCBP1-AS1	3.66	3.49	4.91	4.81	0.76	0.005519	0.025057
CC2D2A	10.11	12.38	7.04	6.93	-0.40	0.008841	0.037846
MRPS12	16.80	14.29	7.17	7.23	-0.79	0.000510	0.002984
GCAT	11.71	13.44	6.79	6.92	-0.62	0.003456	0.016602
MOK	12.27	9.75	4.90	3.79	-0.98	0.000004	0.000032
TRIM52	3.30	2.66	3.47	3.36	0.58	0.003288	0.015920
PAG1	0.96	1.98	2.80	2.40	1.88	0.000000	0.000000
XPC	22.95	21.64	25.15	25.17	0.47	0.000001	0.000005
ZC3H13	17.02	16.80	10.99	11.66	-0.22	0.010285	0.043237
MAP7D3	6.92	6.80	2.77	3.13	-0.91	0.000000	0.000001
RASGRP3	1.97	0.81	3.46	1.81	1.92	0.000000	0.000004
DNAH10OS	0.70	0.84	1.80	1.69	1.52	0.000000	0.000000
SRPX	42.87	43.37	27.07	24.86	-0.41	0.000064	0.000440
ZMYM2	20.48	11.46	21.05	15.14	0.56	0.000000	0.000001
PCM1	29.34	31.35	24.63	22.37	-0.31	0.001992	0.010153
FKBP9P1	1.64	1.75	2.83	3.09	1.14	0.000094	0.000632
SF3A1	51.17	51.73	21.99	26.94	-0.83	0.000000	0.000000
UTP4	28.38	28.15	14.38	11.84	-0.81	0.000000	0.000000
ADAM17	13.86	12.92	22.37	21.15	1.17	0.000000	0.000000
MAN1B1	47.01	49.59	65.75	70.44	0.82	0.000000	0.000000
AC244197.3	3.84	3.68	6.31	7.18	0.98	0.000000	0.000000
DCAF15	20.67	21.56	12.33	12.20	-0.55	0.000814	0.004539
SSBP1	70.25	75.04	46.14	34.91	-0.43	0.004916	0.022597
SATB1	1.57	1.73	2.84	1.38	0.95	0.001294	0.006903
ALAD	10.21	9.50	4.74	6.58	-0.49	0.003410	0.016409
VPS37B	9.73	11.26	5.41	4.92	-0.70	0.000038	0.000269
CCDC3	0.35	0.33	0.74	0.86	1.58	0.003738	0.017761
TCERG1	42.97	40.89	31.25	24.27	-0.42	0.000004	0.000037
SEL1L	27.42	26.81	41.70	36.74	0.76	0.000000	0.000000
BLCAP	20.20	20.22	32.24	31.77	1.10	0.000000	0.000000
USP9X	16.29	13.59	11.18	10.07	-0.26	0.001963	0.010030
KXD1	38.71	44.87	23.19	26.61	-0.37	0.006057	0.027161
NADK	22.46	26.53	15.27	17.04	-0.39	0.001870	0.009613
CHML	4.31	4.66	2.72	2.16	-0.47	0.001278	0.006827
ITSN1	21.68	23.08	13.42	15.43	-0.41	0.000071	0.000488
REXO1	14.85	15.87	7.92	10.61	-0.48	0.000536	0.003115
ARPP19	10.61	9.57	7.14	5.94	-0.39	0.002539	0.012624
FEM1A	1.96	2.36	1.26	1.24	-0.46	0.011787	0.048691
CRISPLD1	2.04	1.79	4.76	2.56	1.06	0.000256	0.001583
FBXL22	0.63	0.64	4.04	3.97	2.94	0.000000	0.000000

GLYR1	34.91	38.34	24.09	26.51	-0.28	0.001723	0.008913
ZNF300	4.65	5.78	6.44	5.89	0.59	0.000241	0.001499
QRICH2	3.85	4.24	7.26	7.37	1.17	0.000000	0.000000
HNRNPR	65.50	61.16	25.79	21.34	-1.00	0.000000	0.000000
AOC3	0.27	0.12	1.27	1.66	3.46	0.000000	0.000000
SPON1	0.07	0.06	0.64	0.77	3.75	0.000000	0.000000
RRM2B	3.86	4.68	9.84	6.81	0.88	0.000000	0.000001
STK38	13.16	13.25	15.57	14.05	0.50	0.000004	0.000032
OPLAH	3.01	2.48	3.61	4.30	1.20	0.000000	0.000000
RPUSD1	15.78	17.67	7.34	9.00	-0.72	0.000030	0.000214
AL035461.4	1.43	0.98	0.46	0.40	-1.14	0.000752	0.004229
SPTBN5	0.68	0.58	1.07	1.18	1.69	0.000000	0.000000
MAFF	7.57	8.21	3.64	4.37	-0.64	0.001384	0.007327
DNAJB11	83.92	74.45	88.64	78.58	0.42	0.000001	0.000010
SNRPF	10.81	12.56	5.58	4.25	-0.98	0.000049	0.000342
ENO3	5.00	5.01	7.81	7.07	1.05	0.000740	0.004170
FZD7	60.02	59.87	60.71	61.86	0.37	0.000000	0.000001
SERPINE2	35.27	32.37	245.74	217.11	3.10	0.000000	0.000000
THAP4	20.16	20.46	10.81	12.41	-0.57	0.000102	0.000679
SOCS5	12.73	11.63	8.32	6.58	-0.37	0.002951	0.014428
METTL3	30.59	28.35	18.15	17.00	-0.43	0.000213	0.001338
STOML2	37.82	45.53	21.00	18.38	-0.82	0.000000	0.000000
KDM3B	16.43	14.96	10.69	9.84	-0.31	0.001968	0.010042
ZNF493	2.36	1.73	2.08	2.51	0.84	0.001958	0.010009
XXYLT1	13.71	13.94	17.62	16.40	0.61	0.000000	0.000002
CCDC124	37.05	42.80	20.72	20.91	-0.63	0.000043	0.000304
C1orf112	4.12	3.76	0.79	0.53	-2.54	0.000000	0.000000
PDGFA	1.55	1.30	2.25	2.71	1.32	0.000201	0.001265
SRA1	20.05	21.94	23.41	22.99	0.56	0.000011	0.000087
PPL	0.82	0.67	0.22	0.32	-1.17	0.004537	0.021083
ARHGEF40	30.34	32.45	19.05	19.17	-0.33	0.000533	0.003104
DDX10	14.39	13.34	4.93	3.94	-1.32	0.000000	0.000000
UTP3	16.03	15.72	8.82	8.80	-0.51	0.000608	0.003492
SF3B3	140.30	141.97	71.28	72.69	-0.75	0.000000	0.000000
DDX6	17.03	14.88	11.39	9.75	-0.29	0.003252	0.015765
SEL1L3	39.28	38.58	57.81	56.53	0.94	0.000000	0.000000
TBC1D20	13.98	12.58	14.15	13.37	0.38	0.000418	0.002484
ATP8B2	27.18	30.66	27.68	30.36	0.43	0.000000	0.000000
GSTM2	39.72	37.40	21.62	22.72	-0.48	0.000605	0.003480
NONO	220.42	205.60	138.51	134.61	-0.38	0.000000	0.000000
SDC4	82.18	80.95	46.13	40.98	-0.57	0.000000	0.000000
KIFC3	33.71	36.11	16.74	18.31	-0.69	0.000000	0.000001
FLII	134.94	139.64	90.66	104.26	-0.25	0.002607	0.012926

EIF3D	106.65	104.98	54.10	55.64	-0.67	0.000000	0.000000
TMEM26	0.23	0.14	2.09	1.57	3.63	0.000000	0.000000
RBMS2	16.69	14.33	9.81	8.65	-0.43	0.000007	0.000053
GAS2L1	11.68	12.56	6.45	6.99	-0.60	0.000093	0.000628
H2AX	43.21	48.23	6.75	6.27	-2.50	0.000000	0.000000
APBB3	12.87	13.36	14.30	16.88	0.57	0.000865	0.004797
RHOBTB2	4.28	4.51	2.28	2.80	-0.59	0.001478	0.007768
CDC7	4.01	3.67	1.02	0.81	-1.68	0.000000	0.000000
PLA2G6	4.87	5.38	6.04	9.17	0.91	0.000171	0.001093
C1RL-AS1	4.33	4.51	4.13	7.67	0.57	0.006664	0.029543
RASL12	0.90	0.81	3.50	3.85	2.42	0.000000	0.000000
ULK1	14.60	16.24	20.14	22.39	0.81	0.000000	0.000000
ZDHHC5	26.52	27.85	31.76	31.74	0.56	0.000000	0.000000
ENG	108.12	118.16	61.25	70.33	-0.44	0.000000	0.000004
SEMA6D	8.02	7.11	16.42	14.91	1.45	0.000000	0.000000
ISG20L2	10.03	10.02	5.82	7.03	-0.59	0.000590	0.003403
MAPK1	21.07	20.27	14.77	15.99	-0.24	0.009875	0.041707
AC009533.1	2.71	3.10	0.87	0.93	-1.35	0.000006	0.000048
C1orf43	44.08	43.02	24.28	20.83	-0.58	0.000001	0.000011
FUBP1	66.82	62.12	43.67	42.29	-0.32	0.000172	0.001098
TM9SF2	57.80	54.45	64.59	56.73	0.45	0.000000	0.000000
GRHPR	57.84	60.41	36.50	38.03	-0.28	0.008056	0.034895
FAM47E-STBD	1.11	1.73	2.95	2.50	1.17	0.000561	0.003245
DFFA	10.84	10.59	6.36	5.10	-0.60	0.000106	0.000708
DZIP1L	9.80	10.15	10.91	11.18	0.46	0.006408	0.028597
KIAA0319L	21.51	24.40	25.76	23.77	0.47	0.000000	0.000000
EPS8L2	12.21	13.93	15.76	13.76	0.43	0.002588	0.012850
SDHA	49.84	53.54	23.95	22.31	-0.86	0.000000	0.000000
SCHIP1	1.17	1.66	1.24	2.63	1.08	0.000630	0.003604
ETV6	7.11	6.87	9.54	9.62	0.69	0.000000	0.000000
TP53I11	11.33	13.22	19.23	19.45	1.05	0.000000	0.000000
URB2	5.37	4.98	1.92	1.85	-1.22	0.000000	0.000000
LENG8	202.55	215.96	205.38	245.55	0.44	0.000000	0.000000
USP14	26.18	24.14	18.19	14.26	-0.35	0.000915	0.005048
KPNA1	16.70	14.65	18.32	15.40	0.36	0.000948	0.005213
PRDM1	1.03	2.48	2.98	2.17	1.09	0.000022	0.000166
CEBPG	6.87	6.35	8.42	7.65	0.64	0.000012	0.000095
NFRKB	9.71	9.32	5.58	5.58	-0.50	0.000160	0.001031
MATR3	3.36	4.83	1.07	0.32	-2.33	0.000501	0.002937
SERPINA3	1.20	1.38	4.66	4.71	2.28	0.000000	0.000000
MATN2	25.05	26.70	53.51	46.75	1.16	0.000000	0.000000
ELOVL5	51.87	48.37	57.74	49.38	0.42	0.000000	0.000004
SMC2	7.35	7.02	1.29	0.93	-2.44	0.000000	0.000000

VEPH1	6.44	9.28	5.56	4.49	-0.79	0.001399	0.007401
GTF2F2	13.44	12.09	6.90	5.32	-0.73	0.000027	0.000196
ARHGAP5	6.21	4.82	5.74	5.56	0.34	0.011891	0.049093
RCC1	25.29	26.22	7.95	7.39	-1.41	0.000000	0.000000
EIF3K	87.09	101.59	46.23	45.06	-0.66	0.000000	0.000001
YLPM1	25.03	24.09	17.14	15.58	-0.38	0.000010	0.000080
SERTAD4	0.09	0.12	0.97	0.47	2.14	0.000494	0.002900
SYDE2	0.79	0.88	1.35	1.14	0.96	0.002042	0.010379
TBC1D14	10.26	10.82	6.52	6.65	-0.36	0.004152	0.019530
POLR1C	19.91	19.35	10.68	9.64	-0.63	0.000410	0.002441
CBX6	16.45	17.08	9.67	10.89	-0.40	0.000061	0.000424
TMEM67	3.75	3.00	7.25	5.19	0.97	0.000017	0.000131
ANKRD11	71.44	71.32	66.23	72.64	0.22	0.001004	0.005498
ILF2	101.74	101.62	48.65	41.39	-0.88	0.000000	0.000000
HNRNPA0	11.67	12.04	5.69	4.90	-0.83	0.000000	0.000000
TWNK	4.91	6.10	2.95	3.17	-0.87	0.000041	0.000287
MLPH	26.79	23.61	5.46	6.52	-1.74	0.000000	0.000000
NCAPG2	13.54	12.69	1.30	1.23	-3.11	0.000000	0.000000
E2F2	0.94	0.94	0.00	0.02	-6.03	0.000000	0.000001
CNTNAP3	0.55	0.41	1.63	1.59	2.10	0.000000	0.000001
STMN3	9.54	8.75	6.15	4.17	-0.64	0.000725	0.004094
SORBS3	55.92	67.03	27.97	35.72	-0.54	0.000009	0.000074
SNRPB	80.77	84.74	32.77	32.63	-1.02	0.000000	0.000000
CAMKK2	9.35	9.93	5.30	4.88	-0.53	0.000084	0.000568
PSMD4	85.97	88.66	56.12	56.86	-0.39	0.000062	0.000424
RELN	18.60	19.28	6.68	7.11	-1.00	0.000000	0.000000
FAM83G	7.46	8.05	3.55	3.79	-0.74	0.000000	0.000001
SF3B5	38.57	44.08	21.88	21.16	-0.59	0.001389	0.007347
PPM1G	41.64	41.49	17.72	17.67	-0.89	0.000000	0.000000
TCAF1	27.67	28.78	17.43	16.99	-0.26	0.003730	0.017732
LEPROTL1	15.96	16.63	22.04	20.68	0.59	0.000039	0.000275
IER2	15.44	15.25	8.73	9.16	-0.38	0.006403	0.028579
APOL2	10.44	7.98	9.93	10.18	0.46	0.004461	0.020782
EMC7	46.34	43.73	56.85	47.43	0.55	0.000006	0.000052
CRMP1	2.88	2.39	3.52	3.85	0.81	0.000566	0.003275
KDM5B	24.57	24.61	30.62	27.41	0.47	0.000000	0.000000
NOP14	17.37	17.52	7.83	6.99	-0.86	0.000000	0.000000
TMEM87B	13.39	12.02	20.38	19.80	0.88	0.000000	0.000000
TMEM179B	22.93	22.30	26.67	27.04	0.58	0.000167	0.001070
CASC4	23.38	21.12	22.55	20.17	0.34	0.000539	0.003129
SH3BP5-AS1	4.99	4.39	6.19	5.67	0.67	0.000016	0.000123
ZBTB7B	9.58	10.15	10.63	10.86	0.49	0.000341	0.002059
CD320	47.43	55.70	22.40	23.13	-0.83	0.000000	0.000000

OLA1	32.22	31.60	17.16	15.78	-0.61	0.000011	0.000088
WDR45	24.25	24.36	26.86	30.64	0.55	0.000068	0.000464
ZNF558	5.98	6.31	9.10	8.80	0.70	0.000014	0.000110
CEP295	4.44	4.95	1.85	2.27	-0.84	0.000006	0.000046
ATG9A	31.77	32.13	32.46	35.01	0.39	0.000010	0.000081
PPP4R3A	28.10	26.14	27.64	27.02	0.31	0.003448	0.016572
HELLS	11.32	9.94	2.09	1.53	-2.32	0.000000	0.000000
TGFB3	3.13	2.78	7.80	8.15	1.64	0.000000	0.000000
WIPI2	17.91	16.35	8.43	8.50	-0.68	0.000002	0.000014
ARL6IP6	4.94	3.90	3.01	2.60	-0.82	0.004200	0.019737
RNF20	11.31	9.92	5.48	6.90	-0.55	0.000049	0.000342
DLGAP1-AS2	1.07	0.58	2.14	2.03	1.52	0.000998	0.005468
ERLEC1	24.97	25.24	26.94	23.42	0.36	0.000719	0.004063
HERC4	40.25	36.74	13.94	12.08	-1.33	0.000000	0.000000
CLCC1	22.05	21.25	22.52	22.86	0.35	0.000527	0.003074
INSC	0.00	0.00	1.23	1.23	6.91	0.000000	0.000001
CUL3	26.32	24.80	17.58	15.80	-0.35	0.001301	0.006936
PIK3R2	13.13	15.00	5.80	8.05	-0.76	0.000000	0.000001
ECHS1	33.47	35.84	19.03	19.46	-0.51	0.000201	0.001267
TOLLIP	10.46	9.71	6.31	6.16	-0.46	0.004252	0.019951
SPCS3	32.45	27.30	35.63	29.14	0.34	0.000230	0.001433
EFNA5	1.59	1.35	4.14	3.48	1.45	0.000000	0.000003
AC126755.7	7.33	7.49	7.42	8.61	0.45	0.001109	0.006002
FADS3	64.94	73.98	89.32	91.16	0.64	0.000000	0.000000
ORMDL1	30.80	34.47	40.41	35.16	0.64	0.000000	0.000002
SECTM1	5.14	7.41	1.10	1.43	-1.97	0.000000	0.000000
WDHD1	5.97	4.73	1.29	1.10	-2.16	0.000000	0.000000
PARN	9.00	9.26	5.08	5.25	-0.56	0.000644	0.003672
SLC38A9	5.96	4.84	9.38	9.07	1.01	0.000002	0.000016
DES	2.46	3.32	8.62	5.91	1.59	0.000000	0.000000
UNKL	8.62	9.86	5.43	5.88	-0.55	0.001355	0.007197
SLFN11	26.86	26.86	17.75	14.75	-0.42	0.000017	0.000126
ECT2	13.64	13.18	2.61	2.16	-2.26	0.000000	0.000000
RECQL4	25.85	26.61	3.26	3.91	-2.70	0.000000	0.000000
BAZ2B	11.10	10.50	10.32	12.45	0.61	0.000003	0.000022
ETS1	15.02	12.46	7.63	8.30	-0.41	0.001022	0.005580
FAM122B	12.79	9.92	7.64	5.74	-0.67	0.000037	0.000260
DDHD1	4.47	4.59	7.69	7.07	1.01	0.000000	0.000000
ZMYND8	12.37	12.07	7.55	6.82	-0.43	0.000102	0.000682
CKAP2L	5.51	4.72	0.53	0.23	-4.04	0.000000	0.000000
DCTN1	120.45	116.06	63.19	66.33	-0.51	0.000000	0.000000
SF3B2	90.63	89.39	52.97	54.55	-0.44	0.000000	0.000000
ARMCX4	6.75	6.70	4.31	4.45	-0.43	0.001092	0.005925

PUDP	7.01	7.17	2.92	2.49	-1.06	0.000005	0.000044
XKR5	0.51	0.51	0.02	0.07	-3.17	0.000024	0.000174
SLC35F6	16.36	15.03	15.42	15.33	0.31	0.002670	0.013207
PCDHGB1	1.68	1.89	3.75	4.37	1.62	0.000000	0.000000
UBR5	41.62	43.17	29.00	26.42	-0.26	0.000881	0.004876
PIP5K1C	13.62	14.65	8.32	8.94	-0.38	0.001470	0.007736
SNRPD2	77.05	74.27	32.39	34.46	-0.84	0.000000	0.000000
SPC24	5.47	6.87	0.19	0.55	-3.94	0.000582	0.003360
NIPAL3	8.67	8.85	4.74	4.70	-0.54	0.000028	0.000206
CDC25C	4.34	5.05	0.07	0.26	-4.95	0.000000	0.000000
WDR35	3.05	2.59	1.71	1.55	-0.49	0.011519	0.047708
PLXNB2	192.27	201.75	220.37	244.90	0.64	0.000000	0.000000
THOC1	19.43	16.83	10.71	8.49	-0.65	0.000150	0.000971
MPP3	11.80	9.56	6.65	5.75	-0.55	0.010056	0.042365
HAUS3	4.84	4.13	2.29	2.13	-0.71	0.002012	0.010240
ID2	29.68	37.55	18.28	19.84	-0.48	0.003734	0.017746
LTBP3	87.81	87.66	80.92	89.98	0.28	0.000727	0.004101
SBF2-AS1	0.87	0.63	1.67	2.00	1.14	0.005749	0.025974
PUS7	7.57	10.74	3.62	5.31	-0.85	0.000002	0.000019
TNFRSF10B	78.12	80.70	144.65	145.98	1.17	0.000000	0.000000
NBEAL1	2.11	1.52	1.74	2.77	0.76	0.000022	0.000161
NUDCD1	10.28	9.13	5.06	3.71	-0.83	0.000013	0.000096
MCM5	33.63	35.20	2.53	3.58	-3.34	0.000000	0.000000
ARPC4-TTLL3	13.93	12.65	14.99	16.14	0.56	0.010730	0.044848
GEN1	7.13	7.04	3.40	5.00	-0.51	0.000569	0.003293
SDC1	54.02	55.63	17.60	21.22	-1.07	0.000000	0.000000
COX7C	70.30	72.51	42.96	39.87	-0.51	0.001410	0.007449
COX8A	117.17	119.89	66.35	59.91	-0.54	0.000169	0.001083
NDUFS5	178.07	177.42	119.95	97.53	-0.34	0.005727	0.025885
LINC01145	1.63	2.20	3.84	3.96	1.51	0.000000	0.000003
CST3	89.02	89.83	110.94	110.42	0.68	0.000000	0.000000
TRIM62	6.43	6.16	7.25	9.61	0.83	0.000000	0.000003
CLTC	120.68	108.08	76.84	68.24	-0.35	0.000000	0.000004
MAK16	6.96	6.87	3.83	2.86	-0.60	0.001121	0.006057
PAMR1	46.12	48.08	88.27	88.51	1.29	0.000000	0.000000
AP1S2	12.21	11.53	6.73	4.97	-0.80	0.000007	0.000056
ZNF577	3.79	2.91	5.72	6.32	0.80	0.000772	0.004329
ZNF331	7.02	6.77	1.84	1.26	-2.14	0.000000	0.000000
FANCL	6.97	6.36	4.00	2.76	-0.74	0.009077	0.038713
SPRY2	6.22	5.77	2.95	2.91	-0.70	0.001496	0.007855
SURF6	13.71	17.05	8.97	9.96	-0.38	0.004815	0.022207
TCEA2	10.51	11.46	12.02	13.89	0.70	0.000965	0.005302
DENND2B	29.92	30.31	42.95	43.75	0.68	0.000000	0.000000

PTN	95.31	91.54	38.42	35.95	-0.99	0.000000	0.000000
CPNE2	12.87	12.36	6.14	4.86	-0.89	0.000001	0.000011
NFKBIA	26.78	27.86	15.26	16.03	-0.47	0.001027	0.005599
TMPO	26.26	24.65	6.60	5.15	-1.98	0.000000	0.000000
CREB3	34.48	37.71	47.48	47.04	0.73	0.000000	0.000000
KCNN2	2.32	2.11	0.66	0.77	-1.74	0.000043	0.000305
SEMA3F	24.04	24.99	23.53	26.01	0.38	0.000357	0.002148
CKAP2	19.64	18.05	3.43	2.69	-2.50	0.000000	0.000000
TARBP1	12.53	11.54	5.86	6.99	-0.63	0.000016	0.000124
XPR1	11.78	11.45	22.55	18.71	1.11	0.000000	0.000000
PRRC2C	42.21	42.89	28.14	27.25	-0.37	0.000000	0.000001
TUBGCP2	23.08	25.18	15.20	12.74	-0.43	0.000047	0.000329
CISD3	7.43	9.03	3.24	2.47	-1.39	0.000005	0.000038
C1QTNF7	0.62	0.44	1.49	1.04	2.87	0.000000	0.000001
ALPK1	2.01	2.48	2.86	2.78	0.79	0.000214	0.001339
P3H4	56.46	62.95	68.63	64.36	0.52	0.000000	0.000000
ICOSLG	0.18	0.08	1.10	1.45	4.13	0.000000	0.000000
NUDT4	12.64	12.73	15.19	12.55	0.58	0.000000	0.000001
GOLM1	46.10	42.81	51.78	50.31	0.52	0.000000	0.000000
FAM120A	37.42	41.46	17.36	18.60	-0.68	0.000000	0.000000
ENOPH1	9.93	11.41	5.70	4.21	-0.81	0.000059	0.000407
KIFC1	28.93	26.52	0.98	1.01	-4.36	0.000000	0.000000
MYBBP1A	48.27	56.90	20.57	21.85	-0.90	0.000000	0.000000
TES	12.54	12.46	19.43	18.37	0.88	0.000000	0.000000
TMEM109	59.02	56.31	30.75	31.10	-0.67	0.000000	0.000000
MBD3	43.28	50.76	23.94	27.89	-0.55	0.000001	0.000011
STAU1	22.58	21.59	14.40	13.49	-0.34	0.000870	0.004821
AGPAT2	12.49	11.44	6.50	5.89	-0.62	0.003435	0.016517
AC091959.3	2.66	2.07	0.96	1.01	-0.92	0.002647	0.013112
CRLF1	27.18	24.91	45.39	36.63	0.94	0.000000	0.000000
EME1	8.89	9.80	0.99	0.47	-3.08	0.000000	0.000000
EPHX1	48.47	42.64	25.12	22.28	-0.63	0.000000	0.000000
DCTN2	73.43	73.61	55.30	42.50	-0.25	0.011047	0.045988
CXXC1	42.92	40.17	25.06	25.25	-0.46	0.000290	0.001777
ARHGAP20	0.73	0.63	1.10	1.23	1.14	0.000162	0.001040
PSMF1	37.31	38.89	22.97	22.88	-0.37	0.001941	0.009934
LTB4R	2.99	2.81	3.18	3.58	0.55	0.007439	0.032518
HOMER3	37.40	39.67	21.54	20.84	-0.62	0.000001	0.000013
AURKB	48.63	49.19	2.11	1.76	-4.27	0.000000	0.000000
TRRAP	14.38	14.56	7.00	7.67	-0.64	0.000000	0.000000
CREBRF	1.81	0.89	1.53	1.77	0.74	0.003389	0.016328
KDM4A	19.29	16.03	18.98	17.03	0.39	0.000035	0.000252
ST6GAL2	0.01	0.00	0.17	0.07	4.40	0.001760	0.009095

AKIRIN2	13.78	14.15	8.04	7.20	-0.49	0.003783	0.017947
STIMATE	5.80	9.17	5.51	4.58	-0.60	0.001499	0.007868
SMAD3	59.71	59.29	16.77	17.63	-1.44	0.000000	0.000000
DNASE2	20.00	20.44	21.31	19.28	0.36	0.004203	0.019745
RPF2	11.48	10.58	5.32	4.93	-1.07	0.000000	0.000000
MAP3K7CL	2.23	1.82	4.42	4.31	1.70	0.000000	0.000004
ATP5MC1	51.74	44.54	16.69	18.63	-0.92	0.000001	0.000007
NUP62	29.93	32.63	13.42	14.67	-0.80	0.000000	0.000000
TTC7B	10.41	11.75	4.83	5.00	-0.54	0.007301	0.031954
SLC52A2	36.74	40.44	42.76	43.62	0.45	0.000015	0.000114
WASH2P	13.27	13.65	25.32	28.54	1.30	0.000000	0.000000
CDK16	50.60	53.45	30.89	29.44	-0.59	0.000000	0.000000
PSMA3-AS1	13.24	14.69	20.02	18.88	0.79	0.000000	0.000000
MPDU1	61.64	56.28	51.51	53.21	0.28	0.008316	0.035930
TRMT112	41.58	46.96	23.68	20.03	-0.78	0.000000	0.000004
ARHGEF39	2.53	3.07	0.20	0.38	-2.87	0.000000	0.000001
HNRNPA2B1	438.42	426.68	254.53	230.65	-0.39	0.000000	0.000000
ZBTB21	5.10	3.88	2.50	2.36	-0.47	0.004391	0.020517
QARS1	121.93	113.86	69.82	69.96	-0.49	0.000000	0.000000
GTF2I	77.16	70.16	51.15	45.25	-0.29	0.000195	0.001233
PRDX3	48.18	45.97	32.34	29.97	-0.27	0.010786	0.045052
VSIG10	5.14	3.80	3.13	3.16	-0.65	0.010261	0.043152
PITPNM2	3.28	3.61	1.54	1.72	-0.64	0.000321	0.001948
MRPL37	36.29	40.54	12.40	13.45	-1.22	0.000000	0.000000
NSMCE4A	10.92	10.87	5.93	4.02	-0.87	0.000618	0.003545
LEFTY1	0.20	0.04	0.46	0.76	3.91	0.001021	0.005580
SSNA1	22.53	25.92	12.35	13.29	-0.58	0.003485	0.016711
NSUN5	7.55	8.60	3.89	4.08	-0.62	0.006449	0.028763
CPEB4	1.84	2.19	4.28	3.78	0.97	0.000001	0.000005
EEF1A1P5	17.92	18.01	9.35	9.21	-0.61	0.000413	0.002455
GRWD1	15.78	19.08	5.09	6.16	-1.32	0.000000	0.000000
GUSBP11	11.47	12.56	15.50	14.21	0.61	0.000001	0.000006
ABLIM3	37.57	38.55	20.18	21.67	-0.59	0.000000	0.000000
ARID1A	23.79	26.33	15.53	16.07	-0.34	0.000028	0.000201
TSPAN9	18.24	18.41	22.18	18.46	0.62	0.000000	0.000000
PLOD1	319.50	343.75	563.48	610.90	1.16	0.000000	0.000000
DCXR	26.36	29.03	12.14	9.79	-0.94	0.000011	0.000085
BBC3	6.90	6.31	11.83	14.45	1.33	0.000000	0.000000
AC118549.1	9.87	8.27	6.11	4.55	-0.48	0.000527	0.003076
ZNF10	4.45	4.05	5.32	5.37	0.55	0.002926	0.014316
MACO1	9.86	9.20	12.03	10.70	0.56	0.000010	0.000077
KRTCAP2	67.15	63.06	71.28	63.29	0.36	0.006667	0.029551
KPNA6	12.29	12.61	7.97	7.11	-0.37	0.000111	0.000737

B3GALNT2	8.15	7.54	9.70	7.60	0.35	0.008804	0.037706
RPS7	369.80	369.49	190.33	182.96	-0.61	0.000000	0.000000
ZFYVE16	10.49	7.75	8.68	9.28	0.35	0.007828	0.034038
CERCAM	72.87	76.74	74.49	81.50	0.39	0.000003	0.000025
ASPN	0.08	0.06	2.51	2.41	5.59	0.000000	0.000000
LETM1	23.86	23.73	13.93	16.77	-0.51	0.000001	0.000005
GADD45GIP1	14.12	16.03	6.72	8.53	-0.64	0.000493	0.002892
NELFB	15.72	15.98	9.40	9.87	-0.39	0.004280	0.020054
EMC8	10.74	12.05	5.98	6.02	-0.69	0.000830	0.004621
TSPYL2	41.29	39.86	52.07	50.58	0.66	0.000000	0.000000
CHAF1A	11.69	13.86	1.38	1.63	-2.83	0.000000	0.000000
HNRNPF	72.03	72.05	28.89	28.73	-1.04	0.000000	0.000000
NCOA3	5.16	4.77	5.88	5.15	0.52	0.000007	0.000059
MT-ND6	380.51	384.23	171.38	156.98	-0.86	0.000000	0.000000
RUNX1T1	2.93	1.98	3.31	4.63	1.19	0.000000	0.000000
MCM4	41.64	32.02	3.53	3.93	-3.11	0.000000	0.000000
SLC26A2	3.31	2.92	3.55	2.98	0.40	0.006409	0.028597
RPS13	284.59	282.98	177.71	151.70	-0.42	0.000032	0.000232
SGTA	30.34	32.13	14.28	16.44	-0.69	0.000000	0.000000
CDK2	28.74	31.69	5.94	7.08	-1.45	0.000000	0.000000
HDAC2	64.91	73.68	46.71	35.22	-0.44	0.000013	0.000098
PARP4	16.01	14.20	8.55	7.91	-0.59	0.000000	0.000000
HYOU1	161.84	164.59	215.08	222.02	0.81	0.000000	0.000000
INTU	3.64	3.44	7.55	7.75	1.39	0.000000	0.000000
CCDC18	8.08	6.72	1.47	0.92	-2.44	0.000000	0.000000
ADAMTS19	0.02	0.01	0.57	0.18	4.31	0.000118	0.000782
XPNPEP2	0.20	0.03	0.38	0.53	3.51	0.000333	0.002016
THAP12	10.85	11.56	4.99	5.44	-0.67	0.000014	0.000109
MUS81	34.97	37.06	41.93	51.23	0.53	0.000037	0.000261
TTLL3	22.48	24.90	29.52	33.02	0.78	0.000000	0.000000
COX5A	59.09	63.42	31.74	25.29	-0.77	0.000023	0.000169
ATXN1	4.14	4.69	6.54	8.35	1.02	0.000000	0.000000
VTA1	22.65	21.07	12.67	10.75	-0.51	0.000201	0.001268
KIF17	1.13	1.54	0.44	0.59	-1.57	0.002690	0.013293
TOMM40	46.64	45.08	18.63	21.41	-0.83	0.000000	0.000000
THRA	7.97	8.51	2.96	3.92	-0.99	0.000000	0.000000
RBL1	4.10	2.86	1.08	1.29	-1.70	0.000000	0.000000
MRE11	9.71	6.98	2.77	2.69	-1.22	0.000000	0.000000
DPP9	21.98	20.01	12.46	12.72	-0.30	0.004888	0.022487
ANTXR2	76.92	69.02	151.13	131.32	1.30	0.000000	0.000000
HIPK3	6.35	5.60	8.14	7.04	0.67	0.000000	0.000000
PGM2	8.67	6.40	3.16	3.52	-0.82	0.000036	0.000254
RBM42	33.31	34.47	15.58	17.58	-0.70	0.000000	0.000001

ATP6V0B	59.91	59.65	64.34	62.89	0.43	0.000014	0.000105
PHB	66.86	78.49	33.19	38.69	-0.64	0.000000	0.000000
RRP15	4.57	7.05	3.05	1.60	-1.11	0.000000	0.000003
BRMS1	20.64	22.22	11.06	12.55	-0.47	0.004128	0.019441
ITGB3	16.70	14.85	26.70	25.90	1.09	0.000000	0.000000
USP44	8.97	8.06	8.01	8.71	0.40	0.006302	0.028178
SRP72	34.04	32.51	23.50	19.32	-0.53	0.000000	0.000000
PPP1R7	22.14	21.54	13.92	11.98	-0.53	0.001944	0.009945
WDR82	17.89	18.45	10.24	9.78	-0.51	0.000001	0.000005
SLC39A9	14.31	13.71	16.07	15.20	0.56	0.000000	0.000000
FTSJ3	40.31	37.94	24.40	23.06	-0.61	0.000000	0.000000
AVL9	8.91	7.79	6.51	8.63	0.33	0.005905	0.026581
MMP1	128.65	121.43	26.53	25.92	-1.91	0.000000	0.000000
GBGT1	3.90	3.27	0.75	1.00	-1.47	0.000045	0.000317
SNRK-AS1	1.36	0.80	1.70	2.84	1.32	0.003347	0.016152
RANBP1	78.39	81.93	23.22	20.24	-1.49	0.000000	0.000000
DEK	31.61	27.07	9.63	8.21	-1.41	0.000000	0.000000
CCN4	2.25	1.72	18.73	17.73	3.34	0.000000	0.000000
AC145098.2	6.21	5.56	10.22	10.98	1.19	0.000000	0.000000
SNRNP200	75.28	75.12	40.08	43.41	-0.55	0.000000	0.000000
TUFM	105.72	106.77	63.77	67.26	-0.47	0.000000	0.000000
RBM8A	50.12	49.04	31.51	29.18	-0.41	0.001067	0.005798
APOBEC3C	22.93	25.65	14.35	16.33	-0.38	0.001281	0.006837
KNTC1	18.60	19.71	3.97	3.52	-2.12	0.000000	0.000000
MGP	1.48	0.60	10.08	8.53	3.48	0.000000	0.000000
FKBP5	5.00	4.20	1.55	1.64	-1.16	0.000000	0.000001
CEP152	3.32	3.37	0.93	0.67	-2.03	0.000000	0.000000
WASH6P	35.91	36.24	34.02	40.39	0.44	0.000257	0.001589
NT5DC3	2.40	3.50	1.40	1.52	-0.96	0.000011	0.000083
SOX4	8.28	8.06	10.95	10.86	0.75	0.000000	0.000000
CCNI	41.16	41.30	21.95	18.29	-0.64	0.000000	0.000000
ELK3	13.40	13.36	6.72	6.27	-0.71	0.000000	0.000000
CNTRL	4.04	3.96	1.46	1.05	-1.28	0.000000	0.000000
TM9SF3	62.23	56.33	74.89	62.83	0.48	0.000000	0.000000
EIF3C	215.86	220.52	141.03	154.11	-0.31	0.000021	0.000153
NINL	5.80	7.28	8.54	8.11	0.49	0.001376	0.007286
HAX1	52.97	53.58	55.86	52.07	0.32	0.003507	0.016799
GYPC	30.98	37.27	36.46	37.26	0.46	0.001087	0.005899
EFTUD2	52.33	65.20	37.11	29.37	-0.67	0.000000	0.000000
CHFR	14.86	15.88	19.26	19.71	0.49	0.000135	0.000880
SPAG5	30.20	32.95	3.66	4.10	-2.92	0.000000	0.000000
COX6B1	111.54	111.75	58.54	58.87	-0.57	0.000118	0.000777
SCUBE1	0.00	0.00	0.06	0.25	5.44	0.000076	0.000516

DUSP6	8.60	7.43	11.95	10.39	0.72	0.000006	0.000052
PSMB6	76.43	91.55	48.44	41.32	-0.59	0.000019	0.000142
RUVBL2	67.17	75.23	29.44	30.03	-0.91	0.000000	0.000000
IGF2R	60.12	66.72	86.20	85.17	0.90	0.000000	0.000000
CLCF1	14.54	15.05	6.22	6.09	-0.92	0.000000	0.000002
PYCR3	5.92	6.51	2.90	3.76	-0.68	0.001475	0.007757
ZNF160	11.96	11.69	15.45	15.79	0.64	0.000000	0.000000
DOHH	6.29	7.24	2.51	2.84	-0.99	0.000165	0.001058
MAP4	135.16	139.40	94.18	97.62	-0.20	0.000922	0.005081
SKP1	203.03	189.44	133.66	115.53	-0.33	0.000511	0.002986
C1QTNF1	4.68	4.74	2.27	1.81	-1.06	0.000011	0.000088
LOXL3	9.76	12.70	17.47	18.24	0.93	0.000000	0.000000
MYBL1	11.86	11.44	0.46	0.47	-4.29	0.000000	0.000000
ROR1	2.73	2.49	4.69	4.85	1.27	0.000000	0.000000
GRK6	13.83	13.07	5.79	8.40	-0.41	0.012113	0.049845
IGFBP2	42.31	45.81	78.82	83.79	1.22	0.000000	0.000000
DIO2	8.54	7.77	2.23	2.37	-1.36	0.000000	0.000000
POLR3G	3.94	3.08	1.04	0.95	-1.41	0.000038	0.000266
NPR2	25.05	22.17	21.83	20.53	0.41	0.000161	0.001032
CAPG	22.46	34.08	10.17	14.31	-0.80	0.000016	0.000123
KIF4A	10.60	10.42	0.38	0.47	-4.27	0.000000	0.000000
MAFK	18.08	18.47	8.81	9.70	-0.69	0.000000	0.000001
LDOC1	31.95	30.14	14.35	14.63	-0.73	0.000000	0.000000
AC010735.2	5.80	5.33	1.91	1.96	-1.01	0.000012	0.000095
ZNF519	1.90	2.40	0.35	0.98	-1.09	0.005782	0.026090
CCL11	0.00	0.07	0.59	0.97	4.90	0.004509	0.020987
DRAM2	13.63	12.37	16.46	13.25	0.50	0.006418	0.028629
UBAP2L	111.88	107.48	65.32	68.85	-0.47	0.000000	0.000000
WDR41	17.43	15.96	25.85	21.38	0.82	0.000000	0.000000
GPR89A	11.91	10.46	14.96	12.39	0.52	0.000594	0.003425
APEX1	87.75	93.54	42.95	44.36	-0.66	0.000000	0.000000
USP54	3.86	3.81	2.57	1.48	-0.66	0.004661	0.021589
SPATA18	1.65	2.23	5.11	4.03	1.41	0.000000	0.000000
POLR3C	14.66	12.17	8.42	7.89	-0.40	0.009078	0.038714
RBM15B	10.83	11.75	6.25	6.27	-0.52	0.000001	0.000010
SRPK1	15.04	13.45	8.12	6.61	-0.74	0.000000	0.000000
NUF2	11.03	13.01	0.35	0.20	-5.37	0.000000	0.000000
GCSH	9.59	6.98	3.59	2.78	-0.93	0.000815	0.004546
TAF1D	95.24	89.73	55.63	49.70	-0.51	0.000000	0.000000
RDH10	31.57	28.61	46.73	47.01	0.75	0.000000	0.000000
HLA-C	7.81	6.50	11.82	11.81	1.08	0.000000	0.000000
EPB41L3	24.17	21.01	10.70	8.26	-0.97	0.000000	0.000000
TLE5	109.72	121.83	70.85	75.84	-0.32	0.000373	0.002238

KIAA1109	21.13	18.33	19.48	18.46	0.24	0.008357	0.036074
EIF3M	123.09	119.90	74.75	60.20	-0.38	0.000181	0.001147
ANPEP	73.82	76.87	39.17	42.30	-0.55	0.000000	0.000000
CYHR1	19.20	21.31	19.28	22.38	0.49	0.000052	0.000359
MRPS15	32.48	33.18	21.07	14.59	-0.50	0.003788	0.017968
CHEK2	7.03	8.90	3.12	2.80	-1.16	0.000175	0.001112
NDFIP1	10.42	9.27	12.48	9.57	0.48	0.000587	0.003384
SSB	56.99	57.06	36.81	30.57	-0.59	0.000000	0.000001
LSM4	37.36	39.28	15.89	15.54	-0.94	0.000000	0.000000
EID1	70.69	76.88	34.95	32.38	-0.80	0.000000	0.000000
UQCRH	76.12	73.42	44.32	41.32	-0.44	0.005523	0.025072
EHD2	139.02	146.36	82.76	84.01	-0.45	0.000000	0.000000
FOXP1	15.96	16.00	10.76	10.43	-0.36	0.002215	0.011169
PKMYT1	29.68	33.70	2.50	1.88	-3.83	0.000000	0.000000
PGBD5	0.17	0.23	0.91	0.78	2.46	0.000001	0.000005
SAMD4A	13.22	13.89	9.05	8.90	-0.34	0.000892	0.004931
SNRPC	66.45	67.78	33.41	27.56	-0.78	0.000000	0.000000
WASH3P	24.69	24.18	26.12	33.54	0.59	0.000042	0.000298
DDX11	26.16	26.61	1.86	3.37	-3.04	0.000000	0.000000
SEC31B	7.32	6.53	7.45	6.98	0.41	0.005543	0.025152
CAPRIN1	100.92	94.10	65.91	58.28	-0.43	0.000000	0.000000
ARRDC4	1.07	0.86	1.63	1.35	0.96	0.001864	0.009587
UHRF1BP1	2.46	2.53	4.22	3.67	0.99	0.000000	0.000000
TMED2	79.62	82.00	83.00	75.88	0.38	0.000001	0.000009
AC027237.5	2.73	2.40	6.03	5.13	1.49	0.000000	0.000000
CCDC150	5.90	4.96	0.36	1.43	-2.90	0.000000	0.000000
DNAJC2	19.76	19.05	13.31	11.54	-0.41	0.002462	0.012274
EIF2S1	52.64	49.12	29.56	21.33	-0.62	0.000000	0.000001
BTBD2	30.66	34.45	14.01	16.81	-0.73	0.000000	0.000000
EIF4E	36.37	30.11	20.14	17.27	-0.57	0.000111	0.000737
FAM207A	14.07	12.49	4.93	4.65	-1.11	0.000096	0.000641
GRASP	3.14	3.15	4.83	5.15	1.02	0.002655	0.013144
TRIM55	2.06	1.77	0.55	0.65	-1.70	0.000083	0.000562
CERS4	1.12	1.64	2.92	2.38	1.22	0.005940	0.026718
CD4	8.94	8.65	10.84	9.60	0.56	0.000077	0.000526
LINC02693	8.13	8.51	8.78	8.81	0.33	0.002299	0.011540
SGSM2	51.79	54.37	51.75	59.12	0.48	0.000000	0.000001
COPS3	29.07	30.65	19.26	16.11	-0.37	0.007808	0.033967
MRPS35	13.86	13.56	8.37	7.15	-0.51	0.003022	0.014755
CCNF	9.85	10.35	0.94	0.97	-3.03	0.000000	0.000000
HYI	43.40	40.76	23.70	23.87	-0.53	0.000054	0.000374
NPRL3	20.44	22.32	11.92	12.63	-0.52	0.000091	0.000610
RBM12	33.69	30.23	15.43	15.69	-0.72	0.000000	0.000000

DDX19A	22.75	22.42	13.06	13.37	-0.47	0.000041	0.000289
FAS	22.03	20.74	31.41	28.63	0.88	0.000000	0.000000
SYNPO	21.03	22.21	26.64	29.60	0.72	0.000000	0.000000
TICAM1	5.57	6.10	3.12	3.21	-0.55	0.008390	0.036204
THSD1	5.30	5.04	1.59	1.61	-1.36	0.000000	0.000000
PRMT2	35.86	39.71	35.52	32.30	0.33	0.002238	0.011264
PSMC3	98.89	108.72	49.90	48.72	-0.67	0.000000	0.000000
NIP7	11.45	10.97	7.12	5.11	-0.59	0.001360	0.007216
ARID3A	10.75	12.21	12.47	12.05	0.37	0.001260	0.006744
GABPB1-AS1	3.86	3.03	4.98	4.33	0.49	0.003472	0.016658
SMOX	8.69	11.09	10.86	10.37	0.46	0.006682	0.029609
HSD17B4	33.20	37.77	21.40	21.26	-0.40	0.001025	0.005596
RBBP4	49.07	48.05	32.03	30.79	-0.28	0.004758	0.021976
HNRNPK	277.38	259.22	159.49	144.15	-0.58	0.000000	0.000000
ANKRD28	39.42	35.84	15.33	13.91	-1.18	0.000000	0.000000
PSMA5	30.62	28.40	16.16	15.46	-0.51	0.000072	0.000493
DCTD	38.99	37.71	21.22	23.03	-0.41	0.000342	0.002064
PRG4	0.02	0.00	1.95	2.24	9.03	0.000000	0.000000
DAZAP1	59.01	58.18	38.37	35.90	-0.24	0.006814	0.030120
GTF2IRD2B	5.30	5.33	7.05	8.58	0.86	0.000001	0.000006
SRP68	33.22	32.45	21.76	20.45	-0.32	0.000781	0.004372
RRM1	37.95	35.24	6.08	5.36	-2.21	0.000000	0.000000
MED1	10.80	10.21	6.80	5.70	-0.48	0.000009	0.000070
ERMP1	2.03	2.42	2.67	2.37	0.53	0.008973	0.038355
CCDC85C	8.23	7.77	4.30	4.31	-0.76	0.000003	0.000027
PHPT1	73.64	79.11	73.27	82.33	0.58	0.000005	0.000039
PPP4C	47.70	55.95	30.06	32.43	-0.33	0.006354	0.028387
WWC1	6.97	6.17	1.29	0.76	-3.04	0.000000	0.000000
KCTD12	0.47	0.62	1.16	0.93	1.27	0.000042	0.000299
GYG1	20.05	19.32	9.50	8.50	-0.79	0.000000	0.000001
GAMT	13.92	14.42	6.90	7.22	-0.89	0.000211	0.001324
SRSF6	91.05	85.99	78.59	75.66	0.21	0.003489	0.016726
LMBRD1	8.07	7.29	10.64	8.93	0.54	0.003066	0.014945
VPS9D1-AS1	8.99	10.67	2.39	2.51	-1.59	0.000000	0.000000
ZNF318	5.36	4.79	2.97	3.15	-0.38	0.002723	0.013427
URB1	8.21	7.37	3.73	3.56	-0.75	0.000000	0.000000
CAMK2N1	13.27	11.98	7.02	6.32	-0.55	0.000842	0.004682
CPQ	14.12	11.48	18.98	15.40	0.74	0.000001	0.000007
ZNF574	6.72	6.59	2.64	3.14	-0.87	0.000008	0.000067
PNO1	7.47	7.46	4.09	3.53	-0.70	0.001495	0.007851
JMY	1.16	0.77	1.75	1.68	1.32	0.000000	0.000000
SSRP1	69.64	68.90	24.38	26.40	-1.07	0.000000	0.000000
MAP4K5	26.77	26.81	21.21	17.90	-0.34	0.000504	0.002950

CD59	368.04	346.23	196.02	174.16	-0.70	0.000000	0.000000
MSRB3	21.13	21.47	29.16	25.96	0.66	0.000000	0.000000
NUP107	20.21	18.77	6.88	6.40	-1.33	0.000000	0.000000
RNF150	2.13	2.36	2.88	2.47	0.52	0.000300	0.001832
LTV1	18.42	19.05	9.15	7.81	-0.81	0.000000	0.000002
AP2S1	50.65	47.96	24.42	25.59	-0.74	0.000001	0.000006
CLCN7	33.30	35.85	30.19	37.71	0.32	0.001363	0.007228
FANCG	18.96	17.79	5.47	6.87	-1.40	0.000000	0.000000
MFSD1	24.33	21.54	26.91	23.77	0.56	0.000001	0.000007
CSRP1	214.50	224.00	196.70	205.26	0.29	0.000012	0.000092
SPG7	78.64	78.52	52.73	52.80	-0.22	0.001962	0.010026
ING4	10.05	9.52	10.53	12.45	0.61	0.008024	0.034761
ATIC	61.47	56.20	27.28	25.63	-0.85	0.000000	0.000000
UBQLN2	11.88	11.70	6.85	6.82	-0.45	0.000156	0.001007
SLC37A4	7.03	7.10	2.80	3.01	-0.98	0.000001	0.000013
MORC4	12.59	11.85	13.33	11.51	0.36	0.003404	0.016388
GBA	45.79	42.91	49.00	51.36	0.43	0.000001	0.000010
SPPL2A	34.59	36.90	47.23	47.37	0.59	0.000000	0.000000
CMTR1	15.35	16.91	9.59	9.89	-0.50	0.000017	0.000129
TNNT2	0.53	1.36	9.88	10.14	4.40	0.000000	0.000000
MT1E	29.53	28.81	4.57	4.37	-2.35	0.000000	0.000000
SPATA20	23.35	23.78	10.48	13.49	-0.65	0.000000	0.000003
NIPSNAP1	26.40	28.90	15.24	14.06	-0.55	0.000008	0.000065
SEH1L	9.01	8.62	4.29	4.32	-0.76	0.000019	0.000141
GCLC	15.33	14.53	19.49	17.23	0.71	0.000000	0.000000
HMOX1	25.98	30.40	5.30	5.51	-2.08	0.000000	0.000000
GPR108	29.24	29.15	33.49	31.24	0.46	0.000236	0.001468
AC007566.1	2.71	0.72	2.23	3.22	1.19	0.000088	0.000594
CCDC40	4.24	4.41	6.45	5.37	0.99	0.000040	0.000284
SPRED2	5.74	5.90	7.53	6.22	0.48	0.000820	0.004567
NEDD8	89.43	96.81	50.95	50.26	-0.51	0.000167	0.001072
CAPZA1	47.46	43.80	27.68	25.93	-0.51	0.000000	0.000001
COL27A1	25.66	29.44	23.58	28.61	0.37	0.000009	0.000072
IFT122	13.42	13.14	5.73	6.51	-0.72	0.000001	0.000011
DYNC2H1	5.90	6.99	5.56	3.48	-0.40	0.008706	0.037341
SOX9	8.85	8.94	4.27	4.16	-0.74	0.000000	0.000002
AL135999.1	4.48	4.21	5.69	6.17	0.79	0.000006	0.000052
ARHGEF1	58.41	58.72	33.78	38.60	-0.46	0.000022	0.000164
CDC42SE2	4.61	4.22	5.51	4.51	0.53	0.002801	0.013768
ANKRD34A	1.13	1.31	1.50	1.77	0.93	0.004297	0.020122
HSPA13	23.68	22.88	27.63	23.20	0.46	0.000001	0.000009
PAK1	14.92	14.72	10.25	8.54	-0.71	0.000000	0.000002
PSRC1	24.61	27.38	4.76	5.87	-1.94	0.000000	0.000000

BCCIP	26.25	25.38	10.07	8.47	-1.08	0.000000	0.000000
RPL22	86.95	87.96	59.74	50.10	-0.37	0.000057	0.000395
SUPT7L	16.16	15.38	17.81	16.92	0.48	0.000012	0.000096
SCARA3	76.50	78.90	23.78	24.44	-1.36	0.000000	0.000000
MEA1	25.12	30.21	13.30	12.18	-0.74	0.000048	0.000336
ADAMTS10	9.87	15.78	19.10	26.81	1.33	0.000000	0.000000
TRPC6	0.65	0.56	2.72	2.62	2.21	0.000000	0.000000
H1-0	12.43	12.84	14.86	14.87	0.57	0.000010	0.000081
ZNF526	5.12	5.42	2.96	3.39	-0.52	0.004430	0.020669
PPP1R8	23.89	22.67	12.50	11.48	-0.48	0.001037	0.005650
SUMF2	69.04	71.57	64.80	65.30	0.24	0.003206	0.015568
RTCA-AS1	0.33	1.08	0.54	0.99	1.23	0.006622	0.029396
WDR3	14.19	14.82	6.38	5.77	-1.05	0.000000	0.000000
UNC13B	4.32	4.57	4.72	4.71	0.41	0.001923	0.009853
C16orf58	57.78	55.58	54.21	61.03	0.38	0.000010	0.000078
NDUFAF3	18.25	21.05	9.20	9.67	-0.68	0.003311	0.016001
APLP1	15.93	16.87	20.64	21.56	0.56	0.000120	0.000792
SETD1B	10.10	9.69	9.12	9.76	0.27	0.004423	0.020644
BYSL	19.32	19.85	5.96	7.38	-1.23	0.000000	0.000000
AC022966.1	19.80	23.10	12.54	12.54	-0.57	0.000000	0.000001
GUSB	36.54	38.46	19.32	21.16	-0.59	0.000000	0.000003
USP10	26.25	26.49	14.27	12.15	-0.82	0.000000	0.000000
ACACB	0.98	0.92	2.18	2.24	1.77	0.000000	0.000000
FGD6	2.07	1.60	2.62	2.43	0.84	0.000000	0.000002
NUDC	46.27	46.70	25.71	22.50	-0.62	0.000000	0.000000
GDF15	52.26	53.71	141.89	149.88	1.80	0.000000	0.000000
ID1	9.08	10.99	1.80	1.48	-2.18	0.000000	0.000000
OLR1	2.18	2.33	5.14	5.62	1.94	0.000000	0.000000
FARSB	8.79	8.47	3.18	2.86	-1.18	0.000000	0.000000
NRBF2	7.98	6.68	9.52	7.19	0.53	0.010713	0.044800
NEURL1B	2.80	2.75	0.88	0.96	-1.26	0.000000	0.000000
NLGN2	41.88	45.06	40.89	46.39	0.32	0.000150	0.000970
DEPDC1	7.04	5.53	0.20	0.15	-4.71	0.000000	0.000000
TRIM14	3.84	3.54	1.28	1.16	-1.24	0.000000	0.000000
FBLN2	30.01	35.36	31.05	32.42	0.28	0.003689	0.017564
PRKAR2A	9.40	9.31	4.99	3.87	-0.69	0.000000	0.000001
MSH5-SAPCD1	5.25	5.17	1.49	1.53	-1.65	0.000000	0.000000
HS3ST3B1	3.09	3.28	6.43	5.80	1.26	0.000000	0.000000
PPFIA4	1.39	1.79	7.48	5.67	2.55	0.000000	0.000000
TTK	7.57	6.57	0.38	0.73	-3.81	0.000000	0.000000
OSBPL10	5.02	5.41	6.29	4.83	0.53	0.003510	0.016808
SNX3	23.95	22.94	14.72	13.15	-0.43	0.003051	0.014882
JADE2	7.89	7.36	1.73	2.36	-1.76	0.000000	0.000000

PDXDC1	45.54	43.19	30.63	31.50	-0.23	0.005389	0.024533
TOR3A	12.65	13.59	7.13	6.67	-0.65	0.000142	0.000921
TRIP6	58.27	62.00	25.06	27.92	-0.86	0.000000	0.000000
RRP1B	11.30	10.20	4.84	4.58	-0.86	0.000000	0.000000
ATP6V1G1	9.17	9.16	13.88	12.34	0.87	0.000001	0.000006
SLC16A2	23.87	22.72	31.09	31.03	0.75	0.000000	0.000000
TMEM131	22.99	19.86	29.93	28.91	0.73	0.000000	0.000000
FAAP100	17.78	18.04	9.22	9.37	-0.64	0.000000	0.000002
IFT140	7.35	4.90	5.58	5.83	0.48	0.002755	0.013564
MRPL4	31.34	35.08	13.74	16.86	-0.73	0.000005	0.000041
MFSD12	56.47	55.37	63.55	67.09	0.60	0.000000	0.000000
NOB1	22.33	19.32	8.07	8.02	-1.08	0.000000	0.000000
ZNF518A	2.44	2.49	2.98	2.69	0.49	0.001859	0.009568
REXO4	11.09	10.50	5.48	4.76	-0.70	0.000050	0.000346
FAM199X	3.08	2.86	3.54	3.85	0.62	0.000011	0.000087
RGS17	0.69	0.50	1.44	1.36	1.58	0.000000	0.000000
ATAT1	11.99	11.47	11.94	12.69	0.39	0.010687	0.044723
ELAVL1	29.33	33.01	17.60	16.26	-0.46	0.000021	0.000153
OCIAD1	57.38	59.25	63.64	61.52	0.42	0.000013	0.000101
DZIP3	5.32	5.70	3.28	3.19	-0.44	0.003708	0.017631
FP565260.3	0.28	0.17	1.68	2.61	3.51	0.000000	0.000000
AP006333.1	0.23	0.15	0.46	0.52	1.71	0.003760	0.017859
NUTF2	51.10	49.47	33.51	30.16	-0.37	0.002459	0.012263
PRC1	72.58	66.29	3.95	3.57	-3.83	0.000000	0.000000
AMOTL1	13.16	12.80	7.49	7.34	-0.60	0.000000	0.000000
SQOR	9.45	9.57	3.39	2.27	-1.33	0.000000	0.000002
AC091057.1	3.07	2.54	0.82	0.40	-2.40	0.000000	0.000000
OLFM2	7.77	10.02	16.92	16.96	1.54	0.000000	0.000000
RCC1L	15.60	17.56	8.50	9.00	-0.59	0.000031	0.000225
ZNF468	2.54	2.39	4.26	4.06	0.89	0.000017	0.000130
SLC44A2	47.02	47.46	51.68	47.58	0.37	0.000002	0.000014
MIS18BP1	7.77	6.63	2.76	3.28	-1.40	0.000000	0.000000
GOLT1B	17.93	17.94	25.01	21.56	0.51	0.000015	0.000115
UBE2L3	22.99	22.97	12.03	11.31	-0.64	0.000000	0.000000
KAT2A	23.04	21.02	12.07	14.88	-0.41	0.001061	0.005767
RPS15A	370.02	398.77	221.02	208.45	-0.45	0.000000	0.000002
CHTF18	31.38	30.00	3.32	6.09	-2.63	0.000000	0.000000
SLIT2	18.30	17.46	10.52	10.00	-0.47	0.000000	0.000000
MRPL20	45.67	45.35	25.76	22.86	-0.42	0.008145	0.035254
EFHD2	20.45	20.20	8.58	8.49	-0.93	0.000000	0.000000
EPPK1	0.06	0.05	0.56	0.61	3.82	0.000000	0.000000
EIF3L	102.37	101.50	56.78	58.95	-0.48	0.000000	0.000000
CHST3	9.76	9.51	12.43	12.49	0.71	0.000000	0.000000

STX12	13.88	10.98	12.58	12.69	0.36	0.011391	0.047228
SPAG9	23.21	22.23	17.30	14.35	-0.35	0.000189	0.001194
CCS	31.16	32.30	31.59	36.19	0.45	0.002218	0.011182
CSAD	34.70	38.53	46.23	47.64	0.62	0.000000	0.000001
PLCH2	1.94	2.26	0.39	0.35	-2.49	0.000000	0.000000
SPOCK1	29.02	33.08	43.44	33.46	0.93	0.000000	0.000000
NPIPA8	16.12	13.60	19.07	15.64	0.53	0.005030	0.023065
AK5	6.62	9.28	3.94	3.86	-1.36	0.000000	0.000000
TSR1	22.54	23.97	12.19	11.91	-0.65	0.000000	0.000000
SYNCRIP	86.01	84.14	40.21	41.48	-0.68	0.000000	0.000000
AK4	0.74	0.69	7.89	5.11	3.85	0.000000	0.000000
CDK1	17.60	15.25	1.32	1.19	-3.44	0.000000	0.000000
INKA2	1.63	1.37	2.65	2.51	1.23	0.000000	0.000000
N4BP2L2	23.35	20.42	40.21	43.49	0.88	0.000000	0.000000
MCM3	29.19	31.82	3.36	3.67	-2.79	0.000000	0.000000
POGK	8.18	7.59	4.50	4.61	-0.35	0.005069	0.023214
LUC7L	40.10	38.46	36.12	35.65	0.29	0.004666	0.021604
EIF5B	33.34	32.95	18.66	16.93	-0.63	0.000000	0.000000
CSNK1G1	4.53	4.67	4.69	5.08	0.37	0.003959	0.018721
SOCS6	6.02	6.28	3.34	3.54	-0.51	0.002706	0.013366
RALBP1	11.38	12.01	6.66	6.05	-0.61	0.000001	0.000007
MAP2K1	14.09	14.84	8.15	8.03	-0.42	0.002381	0.011910
BUB1B	15.53	13.18	0.94	0.75	-3.89	0.000000	0.000000
POP1	10.56	10.44	4.78	4.23	-0.75	0.000000	0.000000
GRPEL1	15.15	16.23	6.80	7.24	-0.78	0.000055	0.000378
ABCF1	38.02	37.11	21.61	20.54	-0.56	0.000000	0.000000
PLK1	41.25	40.72	3.28	2.90	-3.27	0.000000	0.000000
FANCA	24.53	22.06	1.73	2.89	-3.16	0.000000	0.000000
NRP1	153.03	146.84	61.77	52.68	-1.07	0.000000	0.000000
UBA2	28.77	28.64	15.08	12.47	-0.69	0.000000	0.000000
PNPLA7	1.07	1.18	1.13	1.73	0.92	0.004109	0.019365
NDUFS6	19.50	20.86	11.15	11.26	-0.64	0.001032	0.005626
FANCD2	13.34	10.26	1.33	0.48	-3.90	0.000000	0.000000
POLI	8.16	7.56	10.29	9.01	0.61	0.000275	0.001695
ADAM9	237.24	220.90	220.46	197.34	0.20	0.002266	0.011384
BMF	0.91	1.18	7.10	7.32	3.31	0.000000	0.000000
SRP9	53.62	53.69	31.78	25.88	-0.59	0.000002	0.000014
RPL9	321.22	310.76	188.13	156.27	-0.53	0.000000	0.000000
HSBP1	19.21	18.25	11.27	9.62	-0.54	0.000733	0.004134
SNX17	39.38	37.78	25.03	25.14	-0.38	0.000186	0.001175
SSBP4	39.25	47.39	22.76	28.80	-0.50	0.001304	0.006946
ASCC2	30.18	29.62	20.37	24.15	-0.38	0.000629	0.003597
TIGAR	7.03	7.58	7.67	6.70	0.94	0.000006	0.000048

TRAPPC1	70.68	75.28	33.12	34.52	-0.63	0.000004	0.000037
TSKU	22.62	24.02	37.06	39.29	1.05	0.000000	0.000000
WARS1	136.38	140.77	226.36	219.05	1.04	0.000000	0.000000
ABR	24.08	27.03	16.26	14.88	-0.55	0.000000	0.000000
SGO2	5.78	5.63	0.52	0.40	-3.38	0.000000	0.000000
TMX4	4.47	4.62	5.35	4.59	0.48	0.000398	0.002375
RANGAP1	62.38	68.19	21.56	23.10	-1.22	0.000000	0.000000
GSK3A	23.65	26.72	15.85	14.39	-0.35	0.009260	0.039409
IRF3	45.32	53.85	29.78	35.79	-0.33	0.007897	0.034310
CPE	4.59	6.18	7.70	7.13	1.16	0.000000	0.000000
RHBDF1	67.65	78.07	88.92	102.76	0.61	0.000000	0.000000
LIMK2	12.81	10.15	13.75	13.46	0.39	0.005491	0.024956
CAP2	9.98	9.89	11.17	8.94	0.38	0.008238	0.035609
KCTD5	10.74	10.47	5.33	5.33	-0.63	0.000169	0.001082
CACYBP	40.27	38.60	19.76	14.45	-0.90	0.000000	0.000000
NTMT1	35.92	39.06	18.70	20.90	-0.68	0.000034	0.000244
AMZ1	0.51	0.57	1.53	0.92	1.48	0.000080	0.000540
GOT1	19.25	20.46	25.57	25.77	0.58	0.000002	0.000014
COPRS	23.73	24.26	9.71	8.80	-1.02	0.000002	0.000015
PCDHGA10	6.66	6.02	10.17	9.88	1.00	0.000000	0.000000
ANKRD33B	1.63	1.50	0.80	0.81	-0.65	0.001575	0.008230
MEG9	24.65	25.26	43.17	48.40	1.27	0.000000	0.000000
CHAMP1	11.50	11.25	5.64	5.38	-0.64	0.000001	0.000013
MMP24OS	25.54	29.84	13.90	13.29	-0.69	0.000002	0.000017
MFAP2	23.62	23.93	58.40	58.06	1.66	0.000000	0.000000
MAGED4B	114.67	102.90	121.29	132.91	0.58	0.004113	0.019376
UTP18	13.57	14.62	6.24	5.64	-0.94	0.000000	0.000001
ITGB5	72.51	69.25	111.68	119.85	1.09	0.000000	0.000000
PFKL	47.41	53.79	52.26	58.69	0.47	0.000004	0.000030
NIFK	29.59	33.63	16.78	9.76	-0.81	0.000003	0.000028
XPOT	41.65	38.26	47.01	45.25	0.36	0.000002	0.000015
DNAJC11	31.21	30.11	17.25	17.10	-0.56	0.000000	0.000000
ST6GAL1	2.75	2.36	6.20	4.88	1.28	0.000000	0.000000
BMPR2	15.83	14.48	22.33	20.73	0.85	0.000000	0.000000
DNAJC8	46.02	43.37	23.60	21.48	-0.71	0.000000	0.000000
PSMB5	107.42	111.16	72.97	68.23	-0.30	0.001410	0.007450
LANCL1	7.40	8.26	4.77	3.71	-0.76	0.000006	0.000052
GDF6	0.20	0.24	1.16	1.10	2.70	0.000000	0.000000
PTCH2	1.45	1.22	2.33	3.13	1.36	0.000000	0.000001
GPAT4	52.27	44.14	48.31	45.02	0.37	0.000007	0.000060
POLE3	14.73	14.63	6.23	6.59	-0.88	0.000000	0.000001
EIF2B3	16.60	14.13	9.28	8.01	-0.48	0.004845	0.022304
CSNK1A1	54.17	52.94	39.91	34.97	-0.31	0.000137	0.000895

TRHDE	10.39	8.88	10.20	8.23	-0.65	0.000000	0.000000
TXNRD1	133.38	129.87	24.51	21.27	-2.19	0.000000	0.000000
CACHD1	5.42	4.82	5.83	5.76	0.49	0.000305	0.001859
HAUS5	7.71	7.47	1.88	2.29	-1.30	0.000000	0.000000
CEP95	14.05	15.01	17.78	15.44	0.52	0.000677	0.003847
PSMC2	31.93	30.69	22.23	18.66	-0.38	0.000565	0.003270
IMPDH2	111.25	111.49	50.12	44.61	-0.98	0.000000	0.000000
ANKZF1	18.38	20.12	26.54	27.97	0.70	0.000000	0.000000
GPM6B	2.04	2.42	0.40	0.30	-2.45	0.000000	0.000000
ERF	32.94	30.55	19.28	22.87	-0.36	0.003068	0.014955
UGDH	32.87	29.82	17.66	17.11	-0.46	0.000073	0.000498
WLS	142.08	132.05	233.91	201.09	0.97	0.000000	0.000000
TMEM39A	30.74	27.57	28.89	29.06	0.29	0.003573	0.017097
HMOX2	23.89	21.83	13.19	12.31	-0.60	0.000292	0.001792
KPNA3	16.56	15.25	9.38	8.29	-0.56	0.000002	0.000015
FAM20C	38.38	43.87	37.21	37.43	0.25	0.006112	0.027384
ARHGAP18	4.45	4.01	1.93	1.71	-0.92	0.000003	0.000027
LINC00632	4.41	4.64	7.44	5.50	0.80	0.000000	0.000000
CS	76.24	72.43	42.12	38.48	-0.61	0.000000	0.000000
PPIC	22.60	23.35	30.28	28.82	0.70	0.000000	0.000000
PLXDC1	0.89	1.55	4.63	2.76	1.99	0.000000	0.000000
MARCKSL1	85.57	84.95	46.61	42.66	-0.59	0.000000	0.000000
ZFP36L2	15.73	15.80	10.39	9.23	-0.35	0.001967	0.010039
IKBIP	50.96	43.18	51.44	44.49	0.38	0.000167	0.001070
PRMT1	133.89	146.81	57.11	59.93	-0.96	0.000000	0.000000
FBXO42	8.55	6.29	6.90	6.81	0.35	0.012034	0.049564
CCND3	20.68	22.43	8.39	8.46	-1.04	0.000000	0.000000
ADH5	74.23	65.79	48.46	41.40	-0.43	0.000004	0.000032
CYFIP1	32.47	34.48	19.18	21.44	-0.46	0.000001	0.000009
RITA1	5.92	6.12	3.00	3.55	-0.74	0.003398	0.016361
TSR3	22.75	23.50	11.75	11.95	-0.63	0.000234	0.001459
ATP1A1	138.40	139.52	80.34	76.89	-0.49	0.000000	0.000000
TIMM13	31.99	32.33	16.51	19.43	-0.44	0.006578	0.029227
INCENP	16.94	16.19	2.55	1.69	-2.68	0.000000	0.000000
DBF4B	10.00	9.96	1.50	1.89	-2.29	0.000000	0.000000
RBM3	148.26	145.82	100.92	96.46	-0.25	0.000743	0.004181
TRAF4	40.53	39.69	42.34	51.98	0.61	0.000000	0.000000
PWP1	25.59	22.09	16.18	11.19	-0.57	0.000006	0.000048
IFRD2	35.42	40.97	17.62	15.59	-0.96	0.000000	0.000000
ADGRE5	21.17	23.08	10.41	9.95	-1.09	0.000000	0.000000
GNPNAT1	9.10	9.60	4.68	4.06	-0.77	0.000000	0.000004
KDM4B	10.09	10.83	13.83	13.86	0.73	0.000000	0.000000
SENP3	22.58	24.13	15.53	14.88	-0.43	0.000649	0.003700

VEGFA	85.85	89.22	235.85	232.41	1.74	0.000000	0.000000
HLTF	7.97	7.63	3.86	3.89	-0.60	0.000037	0.000263
AHNAK	133.83	125.95	71.68	71.67	-0.54	0.000000	0.000000
NEK6	47.95	46.70	20.58	21.50	-0.75	0.000000	0.000000
NFIC	40.71	40.04	19.37	19.95	-0.62	0.000000	0.000000
NAA50	32.42	29.16	13.32	11.31	-0.90	0.000000	0.000000
GOLGA3	23.09	22.90	19.11	23.85	0.23	0.006959	0.030668
PGRMC2	66.70	59.44	92.65	76.43	0.76	0.000000	0.000000
ALG5	14.23	15.72	19.92	15.25	0.60	0.001146	0.006181
RHOU	0.30	0.18	0.50	0.54	1.45	0.009548	0.040549
TRIO	129.45	127.91	95.58	97.40	-0.24	0.000132	0.000865
IDUA	12.90	11.74	12.21	12.82	0.46	0.004000	0.018901
LRCH4	42.60	44.22	42.56	50.27	0.50	0.000002	0.000020
PGPEP1	4.31	3.52	5.24	6.46	1.00	0.000000	0.000000
RTF2	52.30	56.68	31.93	28.85	-0.51	0.000001	0.000009
CCDC80	416.38	404.92	591.34	552.79	0.70	0.000000	0.000000
ZMIZ1	33.02	32.65	16.42	18.29	-0.37	0.000003	0.000029
CH25H	0.06	0.07	0.89	0.70	3.90	0.000124	0.000815
GOLGA6L4	2.68	2.69	3.97	3.40	0.85	0.000063	0.000434
PPP2R3A	6.21	5.12	2.78	3.33	-0.58	0.000117	0.000776
RPS20	320.69	313.34	202.29	171.13	-0.40	0.000014	0.000110
C4orf3	10.49	10.28	11.38	10.12	0.39	0.004008	0.018934
CTXN1	2.82	3.64	4.31	4.80	0.84	0.011778	0.048662
UPF1	29.13	28.64	14.72	16.15	-0.57	0.000000	0.000000
PFDN1	20.08	21.55	13.01	11.03	-0.46	0.007229	0.031699
MRPS24	53.14	53.91	17.20	21.56	-1.12	0.000000	0.000000
HNRNPUL1	87.88	88.51	44.94	44.78	-0.74	0.000000	0.000000
XRCC6	136.55	133.15	64.94	57.66	-0.79	0.000000	0.000000
PDIA5	38.31	41.39	63.51	54.91	0.80	0.000000	0.000000
SEPTIN8	18.59	18.91	12.37	11.70	-0.30	0.004147	0.019512
CEP57	13.88	12.23	7.40	6.37	-0.50	0.000692	0.003922
GABARAPL1	18.20	21.09	37.86	32.86	1.26	0.000000	0.000000
LINC01583	0.64	0.44	2.01	1.70	2.11	0.000000	0.000002
EPHB3	4.88	6.08	12.32	12.82	1.58	0.000000	0.000000
CARM1	35.15	37.41	16.08	17.36	-0.83	0.000000	0.000000
DHX37	11.30	13.21	4.50	3.89	-1.15	0.000000	0.000000
NOTCH3	19.02	17.74	32.88	37.16	1.27	0.000000	0.000000
UCHL1	394.62	400.75	420.67	373.11	0.35	0.000000	0.000001
NRM	24.96	25.77	11.81	11.11	-0.83	0.000000	0.000001
PGM1	18.27	18.79	22.96	20.19	0.54	0.000002	0.000020
ITPR1	1.43	0.87	3.88	3.54	2.35	0.000000	0.000000
UGGT2	23.71	20.87	22.35	20.11	0.30	0.000900	0.004973
MXRA5	0.53	0.59	1.58	1.54	1.82	0.000000	0.000000

CD81	384.38	427.60	431.67	492.16	0.51	0.000000	0.000000
VASH1	4.56	4.80	4.73	5.73	0.46	0.000917	0.005057
DUSP7	6.95	6.76	2.92	3.13	-0.85	0.000002	0.000018
UCN2	6.81	7.52	16.03	18.44	1.61	0.000000	0.000000
NOMO3	64.18	56.32	66.62	56.36	0.27	0.000305	0.001862
KLHDC10	9.20	6.97	7.76	8.17	0.35	0.008414	0.036300
MGAT5B	1.27	1.62	0.35	0.71	-1.36	0.001343	0.007138
TMTC2	0.70	0.70	4.00	3.68	3.15	0.000000	0.000000
URI1	16.63	14.91	9.97	8.60	-0.42	0.003039	0.014824
HNRNPA1	633.65	637.34	217.15	200.37	-1.14	0.000000	0.000000
ATOH8	2.31	3.23	1.08	0.70	-1.29	0.000454	0.002685
TTLL12	12.88	13.94	4.12	4.94	-1.16	0.000000	0.000000
SARAF	51.94	50.87	90.21	79.15	1.04	0.000000	0.000000
RRP7A	14.61	13.09	7.42	7.64	-0.55	0.000028	0.000203
TSPAN2	0.44	0.69	5.87	6.21	4.07	0.000000	0.000000
PKD1P6	27.38	27.62	33.11	33.42	0.57	0.000004	0.000035
GOLGA6L5P	9.92	10.66	13.93	15.52	0.85	0.000000	0.000000
ZDHHC8	57.89	66.77	38.65	44.97	-0.36	0.001102	0.005974
ABCE1	28.06	27.73	11.55	9.44	-1.01	0.000000	0.000000
SIAE	8.29	7.79	8.38	8.85	0.47	0.001678	0.008710
WDR62	8.12	7.97	0.42	0.44	-3.92	0.000000	0.000000
LRRC8C	5.91	5.42	3.24	3.00	-0.53	0.000073	0.000499
GLB1	40.56	42.05	52.63	51.56	0.68	0.000000	0.000000
RHOG	22.51	23.08	11.37	12.63	-0.58	0.000413	0.002454
PRPF19	46.64	53.36	21.11	21.77	-0.96	0.000000	0.000000
MTMR1	19.19	15.14	12.65	9.63	-0.41	0.000796	0.004448
STMN1	129.44	125.08	23.84	19.51	-2.11	0.000000	0.000000
RNPS1	56.44	56.76	34.20	35.37	-0.30	0.001508	0.007907
TIMELESS	13.09	13.69	2.09	2.85	-2.05	0.000000	0.000000
AC110285.2	1.92	1.73	2.21	3.17	1.09	0.008519	0.036665
TUSC3	66.78	70.74	86.11	78.57	0.54	0.000000	0.000000
KIF14	3.45	3.62	0.14	0.19	-4.12	0.000000	0.000000
DEGS1	85.78	83.33	107.35	91.99	0.56	0.000000	0.000000
SYTL5	0.32	0.38	1.98	1.91	2.81	0.000000	0.000000
SPNS1	31.74	32.26	40.89	43.52	0.75	0.000000	0.000000
INAFM2	4.60	5.01	6.65	5.81	0.58	0.002058	0.010453
SPTLC2	19.86	17.02	25.73	21.80	0.45	0.000000	0.000004
CHKB	28.56	26.19	36.99	36.60	0.63	0.000002	0.000018
ACOX3	9.91	9.50	9.58	12.17	0.46	0.003359	0.016204
DDX54	20.37	20.28	11.10	9.54	-0.86	0.000000	0.000000
SLC31A1	13.91	14.46	26.88	22.94	1.16	0.000000	0.000000
WIZ	20.45	20.13	11.96	13.43	-0.27	0.007224	0.031687
SSH1	13.00	12.23	8.66	8.25	-0.39	0.000008	0.000065

ALG2	22.44	21.16	22.75	21.54	0.38	0.000932	0.005132
SYNJ2	19.51	19.98	10.37	10.23	-0.58	0.000000	0.000000
NPIPA1	106.23	103.95	119.92	119.00	0.61	0.000000	0.000000
RBM14	22.96	24.63	10.67	12.28	-0.79	0.000000	0.000000
ZGRF1	4.16	3.96	1.05	0.94	-1.76	0.000000	0.000000
USP19	15.66	17.29	10.39	10.70	-0.36	0.004362	0.020408
LGMN	19.17	19.14	37.56	36.87	1.29	0.000000	0.000000
MARK3	50.73	49.83	54.57	48.40	0.33	0.000116	0.000765
SLC38A7	15.03	15.39	16.32	16.20	0.46	0.000022	0.000161
FKTN	6.73	6.22	3.92	3.29	-0.48	0.000534	0.003105
GALNT5	22.21	20.56	14.81	13.11	-0.24	0.001835	0.009452
HMMR	11.28	10.90	0.33	0.49	-4.41	0.000000	0.000000
MAGED1	270.84	278.70	167.61	164.53	-0.42	0.000000	0.000000
THOP1	52.22	62.97	23.37	25.13	-0.95	0.000000	0.000000
ARNTL2	5.31	5.01	1.46	1.40	-1.53	0.000000	0.000000
LDB1	48.22	52.00	49.27	53.67	0.41	0.000003	0.000024
RABL6	41.99	42.74	24.83	25.16	-0.44	0.000001	0.000009
AC093908.1	0.33	0.32	1.06	0.89	1.93	0.000000	0.000000
PLXNC1	0.53	0.22	0.77	0.55	1.19	0.009775	0.041364
NFIX	36.38	37.29	19.80	22.20	-0.47	0.000000	0.000003
ZCCHC24	11.23	10.96	6.45	7.37	-0.35	0.004454	0.020752
CFAP44	1.04	2.42	4.60	2.95	1.64	0.000000	0.000000
LPAR6	1.70	1.52	2.65	2.60	0.99	0.005702	0.025797
MAMDC4	12.29	10.29	16.84	23.29	1.26	0.000000	0.000000
PHC1	12.00	13.52	12.02	17.07	0.46	0.000280	0.001724
H2AZ1	159.52	161.51	59.70	50.32	-1.15	0.000000	0.000000
RTL10	10.99	11.91	6.52	5.96	-0.47	0.000230	0.001435
ATF3	1.80	3.29	7.10	7.98	2.07	0.000000	0.000000
PRRC2A	104.93	112.70	68.79	77.92	-0.30	0.000279	0.001717
PTGFRN	2.53	1.49	2.38	1.68	0.70	0.003290	0.015926
GABRA5	4.70	2.86	1.17	1.19	-1.25	0.000247	0.001535
C2CD3	9.08	7.61	14.83	13.95	0.94	0.000000	0.000000
TOMM5	39.14	35.48	22.18	18.62	-0.56	0.002596	0.012882
TRIR	89.58	86.51	51.52	47.27	-0.48	0.000014	0.000104
SIPA1	18.75	18.96	10.22	14.24	-0.65	0.000042	0.000296
ASAP3	13.94	14.39	15.08	17.78	0.37	0.001582	0.008265
VPS9D1	14.12	13.16	20.81	23.17	1.02	0.000000	0.000000
COLGALT2	1.96	2.33	2.73	2.61	0.70	0.000870	0.004821
PTPRG	14.02	13.11	20.97	20.19	0.85	0.000000	0.000000
ITFG1	34.48	30.88	46.17	41.34	0.61	0.000000	0.000000
CRYBG1	1.70	1.79	0.36	0.32	-2.03	0.000000	0.000000
EGFR	32.54	30.79	15.27	15.12	-0.75	0.000000	0.000000
EHMT2	32.56	34.48	18.61	20.18	-0.59	0.000001	0.000005

EIF4B	104.53	104.14	59.19	60.39	-0.53	0.000000	0.000000
TECR	72.54	73.85	45.61	47.53	-0.32	0.002765	0.013605
COPS6	93.36	88.87	47.05	41.18	-0.70	0.000000	0.000000
ENSA	22.38	23.57	10.75	13.92	-0.41	0.002685	0.013276
RHOQ	17.22	15.58	22.04	19.43	0.41	0.000775	0.004344
IMP4	44.82	46.69	20.55	18.96	-0.86	0.000000	0.000000
LRIG1	52.38	54.96	60.67	65.62	0.52	0.000000	0.000000
MLLT6	15.04	15.35	10.47	11.01	-0.35	0.002710	0.013381
CARMN	18.88	19.87	39.43	39.76	1.42	0.000000	0.000000
GBE1	15.11	14.60	18.33	16.46	0.56	0.000001	0.000007
MINDY2	2.32	1.79	3.57	3.38	1.08	0.000000	0.000000
PDCD11	29.75	26.39	15.12	16.57	-0.46	0.000000	0.000000
CD55	5.03	5.80	10.66	8.24	1.10	0.000000	0.000002
SSU72	39.12	38.53	23.01	23.23	-0.35	0.003037	0.014816
AAK1	14.61	13.00	12.79	12.99	0.31	0.000780	0.004367
KIAA0895L	22.07	22.43	31.20	35.85	0.85	0.000000	0.000000
ATM	15.97	14.06	16.83	15.54	0.51	0.000000	0.000003
HOXA5	13.19	6.56	3.91	2.75	-1.19	0.000025	0.000181
YIPF4	7.69	6.65	10.02	7.18	0.49	0.002213	0.011163
GTPBP2	50.47	53.55	86.65	88.81	1.08	0.000000	0.000000
LRP5	28.43	29.57	18.26	18.94	-0.40	0.000115	0.000761
VAT1L	9.97	8.65	11.29	10.65	0.57	0.000007	0.000053
CNIH1	91.37	84.20	117.44	93.17	0.57	0.000000	0.000001
ROBO2	10.60	8.64	18.45	15.29	1.49	0.000000	0.000000
P4HB	1,069.70	1,037.99	1,534.58	1,539.61	0.78	0.000000	0.000000
HSPE1	79.74	77.05	41.01	30.69	-0.76	0.000012	0.000095
CYTH2	26.93	29.39	35.58	37.05	0.92	0.000000	0.000000
WDR77	22.46	19.96	7.76	8.49	-0.88	0.000000	0.000000
VTI1B	19.07	21.66	12.86	12.53	-0.40	0.005050	0.023142
ITGAV	50.45	49.20	91.89	83.25	1.12	0.000000	0.000000
LIFR	5.73	4.62	8.36	9.97	0.92	0.000000	0.000000
APOL6	1.29	1.35	2.16	2.26	1.08	0.000000	0.000000
FBL	101.26	123.35	34.40	33.07	-1.39	0.000000	0.000000
MRPS34	40.32	49.61	22.82	25.06	-0.58	0.000184	0.001167
UBE2I	58.73	51.54	35.97	32.59	-0.43	0.000055	0.000384
U2AF2	119.87	124.68	60.22	69.82	-0.62	0.000000	0.000000
ST6GALNAC3	2.44	1.91	5.34	4.96	1.73	0.000000	0.000000
POLR1A	10.61	10.83	5.32	5.70	-0.53	0.000000	0.000000
SSR1	118.95	117.98	139.75	114.71	0.39	0.000002	0.000017
CDH11	176.86	173.92	304.07	292.20	1.04	0.000000	0.000000
SLC25A22	18.43	21.59	8.31	10.18	-0.75	0.000000	0.000002
OGDH	19.86	20.95	11.24	12.52	-0.46	0.000057	0.000397
VANGL2	2.79	2.82	3.54	3.89	0.74	0.000011	0.000085

ATP5MC2	108.22	120.09	56.39	57.62	-0.62	0.000000	0.000000
PCDHGB7	10.28	10.23	12.53	11.75	0.61	0.000000	0.000000
ADAMTS7	13.86	15.17	23.60	27.78	1.19	0.000000	0.000000
ALDH3A2	4.63	4.07	1.82	2.47	-0.90	0.001364	0.007229
BRD3	5.62	6.42	3.67	3.23	-0.52	0.000528	0.003077
EIF3H	79.50	82.03	47.99	45.24	-0.45	0.000004	0.000035
GTF3C4	4.42	4.14	2.83	2.55	-0.40	0.003208	0.015571
WDR43	23.20	19.98	8.97	9.19	-0.73	0.000000	0.000000
AC104452.1	2.88	3.13	5.04	5.34	1.07	0.000000	0.000000
HSPH1	41.95	37.81	21.55	20.00	-0.71	0.000000	0.000000
ARCN1	69.16	69.48	50.31	44.53	-0.28	0.000173	0.001103
RUVBL1	21.96	22.93	10.68	8.64	-0.92	0.000000	0.000000
AC138969.3	2.84	3.17	2.85	3.38	0.39	0.006960	0.030668
KIF15	6.76	5.58	0.42	0.38	-3.98	0.000000	0.000000
GPR63	0.88	0.65	1.31	1.14	1.02	0.000356	0.002144
GLO1	25.23	24.51	14.51	13.26	-0.50	0.000047	0.000328
SLC9A9	1.88	1.14	2.59	2.03	1.66	0.000000	0.000001
ABHD12	42.91	44.03	41.46	43.91	0.29	0.003338	0.016118
FAM155A	0.85	0.80	1.20	0.93	0.70	0.002732	0.013461
NPEPL1	13.50	14.47	13.61	17.44	0.41	0.004425	0.020651
PSEN1	22.75	21.45	31.20	29.03	0.81	0.000000	0.000000
UBA1	152.55	159.67	90.23	99.38	-0.42	0.000000	0.000000
PAK2	10.79	12.68	7.10	8.54	-0.34	0.001941	0.009934
KANK1	8.89	8.57	4.94	4.41	-0.59	0.000017	0.000131
CD2AP	4.31	4.13	4.89	4.61	0.46	0.001884	0.009679
PCDH10	3.36	3.20	7.33	7.40	1.41	0.000000	0.000000
CTSS	1.12	0.56	3.62	2.29	1.93	0.000020	0.000148
SNX30	3.67	2.83	5.87	4.22	0.69	0.000005	0.000039
GALT	19.49	19.10	8.53	10.10	-0.71	0.000260	0.001608
GFPT1	13.98	14.84	23.30	19.15	0.66	0.000000	0.000000
RIPOR2	0.47	0.77	1.17	1.29	1.57	0.000041	0.000287
CAPN1	71.22	79.16	41.77	46.67	-0.48	0.000000	0.000000
HSD17B7	3.79	3.12	4.43	4.55	0.77	0.009556	0.040575
CLTCL1	5.96	6.39	11.64	13.00	1.21	0.000000	0.000000
HIGD1A	21.36	20.78	12.59	10.36	-0.53	0.001805	0.009309
CANX	446.27	413.99	488.01	433.81	0.36	0.000000	0.000000
NLRP1	8.28	9.32	7.92	8.53	0.33	0.006629	0.029410
COA4	25.96	25.80	11.81	9.90	-0.86	0.000092	0.000617
FARSA	44.07	49.61	21.70	20.09	-0.84	0.000000	0.000000
NOX4	4.32	3.29	9.66	9.21	1.80	0.000000	0.000000
RNF145	50.87	48.88	53.09	46.72	0.33	0.000105	0.000701
FAT1	318.36	332.21	293.71	291.05	0.25	0.000007	0.000054
INA	1.31	1.55	2.96	2.83	1.35	0.000000	0.000002

SLC7A14	1.48	1.21	0.54	0.28	-1.73	0.000000	0.000000
SRSF11	119.21	115.54	112.80	103.12	0.19	0.004346	0.020346
PLCB4	8.03	6.71	9.11	10.00	0.64	0.000000	0.000004
DST	123.46	116.93	81.47	80.94	-0.21	0.001216	0.006527
GDNF	1.17	1.11	2.19	2.08	1.25	0.000013	0.000102
JCAD	9.31	9.51	4.87	4.42	-0.55	0.000000	0.000002
VPS4A	17.71	17.97	11.91	11.43	-0.35	0.002160	0.010922
MN1	0.83	0.80	2.02	1.22	0.84	0.000459	0.002708
LMNB2	42.20	44.46	10.47	10.33	-1.85	0.000000	0.000000
STK17B	5.99	5.40	3.09	3.18	-0.57	0.001570	0.008206
SCAMP1	14.25	13.71	17.93	15.10	0.52	0.000088	0.000595
PNRC1	4.70	4.29	7.48	6.30	0.95	0.000012	0.000094
DCAF13	30.09	28.10	17.28	13.65	-0.39	0.001121	0.006057
KLF16	6.21	6.84	3.33	4.23	-0.54	0.005580	0.025307
ABCD4	18.84	16.86	20.52	19.56	0.40	0.003701	0.017609
DLGAP5	16.28	16.10	0.60	0.43	-4.62	0.000000	0.000000
CEP192	9.54	9.10	3.43	4.12	-0.93	0.000000	0.000000
MDH2	81.29	94.36	44.19	50.17	-0.53	0.000000	0.000000
MICAL3	19.76	21.35	11.77	14.54	-0.33	0.005761	0.026012
FAM219A	9.83	9.54	11.77	11.95	0.63	0.000000	0.000001
ANXA6	171.60	183.63	161.49	163.78	0.20	0.003775	0.017918
TMEM59L	9.02	8.65	10.45	12.19	0.67	0.000273	0.001683
TMED9	186.53	174.53	171.99	165.46	0.28	0.000028	0.000206
ATP5PB	103.56	104.95	52.81	55.36	-0.60	0.000000	0.000000
RNF13	8.74	10.09	12.34	10.21	0.52	0.006524	0.029048
RABEP1	14.91	12.47	7.34	8.88	-0.35	0.002863	0.014052
NUPR1	19.11	16.21	43.37	37.93	1.60	0.000000	0.000000
DYNLRB1	76.67	84.61	43.60	40.93	-0.57	0.000036	0.000253
ESYT1	142.34	149.10	153.37	159.72	0.45	0.000000	0.000000
RPL39	356.62	369.71	246.07	209.98	-0.29	0.011539	0.047773
MLXIP	13.77	13.11	13.39	17.64	0.52	0.000001	0.000005
STC2	158.01	168.49	280.15	268.97	1.02	0.000000	0.000000
IFNGR1	14.64	10.97	16.66	14.35	0.64	0.000030	0.000215
DNAJC10	48.80	45.12	65.09	53.79	0.47	0.000000	0.000000
DSP	2.97	3.04	3.75	3.45	0.56	0.000012	0.000091
ELF4	9.28	8.86	5.99	5.21	-0.41	0.002220	0.011185
UBB	402.23	425.57	290.46	285.53	-0.19	0.007891	0.034290
CD3EAP	7.47	6.70	1.36	1.91	-2.05	0.000000	0.000000
MTCH2	37.02	38.52	19.59	18.49	-0.49	0.000092	0.000617
TOMM34	20.82	24.22	6.77	6.15	-1.46	0.000000	0.000000
RPL28	323.90	355.45	201.35	219.96	-0.33	0.000126	0.000829
NDC80	10.52	9.83	0.44	0.28	-4.40	0.000000	0.000000
TMEM51-AS1	0.03	0.26	0.26	0.30	2.09	0.005018	0.023024

SNRPN	57.57	62.48	61.68	60.76	0.36	0.000095	0.000636
RAVER1	18.01	20.01	8.26	9.63	-0.69	0.000000	0.000000
AVEN	8.77	7.21	10.21	8.40	0.56	0.005864	0.026411
SLC25A39	49.28	51.67	25.33	32.24	-0.41	0.001314	0.006994
NUCKS1	33.05	31.59	14.30	12.23	-0.95	0.000000	0.000000
FAM118A	80.83	76.44	114.97	124.41	0.90	0.000000	0.000000
TRIM28	439.37	455.81	207.43	244.54	-0.68	0.000000	0.000000
CLSTN1	93.62	95.98	89.70	93.36	0.31	0.000003	0.000021
SLC25A36	13.93	12.34	17.96	16.81	0.74	0.000000	0.000000
CDK7	24.55	24.07	17.38	12.32	-0.42	0.008519	0.036665
JADE1	12.23	11.84	11.56	12.00	0.34	0.000628	0.003597
HSPA4	58.45	52.73	40.81	31.76	-0.63	0.000000	0.000000
GSTP1	328.68	371.49	191.03	205.41	-0.46	0.000000	0.000002
SCAF1	21.16	22.57	13.02	14.74	-0.38	0.000101	0.000676
TFDP1	37.18	33.36	13.06	13.15	-1.13	0.000000	0.000000
KIF26B	0.63	1.16	5.65	5.51	2.97	0.000000	0.000000
MST1	6.76	7.91	8.53	10.66	0.74	0.000928	0.005116
CCBE1	33.53	33.79	15.87	13.13	-1.31	0.000000	0.000000
CRABP2	13.96	12.14	20.27	20.02	0.99	0.000000	0.000003
KNL1	5.24	5.38	0.26	0.31	-3.73	0.000000	0.000000
RPL37	351.33	368.91	253.64	220.71	-0.21	0.007935	0.034451
SPTLC3	0.66	0.65	0.16	0.65	-1.85	0.006882	0.030386
DOCK6	7.11	11.00	16.03	20.50	1.35	0.000000	0.000000
KIF20B	8.32	8.47	0.80	0.96	-3.13	0.000000	0.000000
GFOD1	0.97	0.63	1.10	1.04	0.96	0.000475	0.002794
AC015813.2	9.54	9.18	8.74	12.03	0.49	0.006395	0.028548
F2RL1	5.22	5.75	3.24	2.59	-0.58	0.006499	0.028969
KLF12	4.55	4.44	5.13	4.49	0.32	0.008182	0.035396
APC2	0.69	0.31	2.18	2.75	2.60	0.000000	0.000000
UTRN	15.19	16.60	11.87	7.26	-0.31	0.001623	0.008458
IARS1	101.37	99.24	103.15	91.93	0.22	0.000764	0.004290
PRNP	95.36	96.19	97.23	91.71	0.37	0.000001	0.000006
SLC35B2	47.37	47.95	43.86	47.79	0.29	0.002219	0.011183
TSC22D1	15.89	15.20	19.34	18.64	0.53	0.000000	0.000002
TENT5A	3.98	3.26	6.29	6.19	1.08	0.000000	0.000000
HDGF	68.57	72.82	17.29	17.20	-1.86	0.000000	0.000000
TRMT61A	6.00	7.09	2.87	3.12	-0.79	0.000040	0.000284
TFE3	31.22	34.02	18.48	21.60	-0.48	0.000001	0.000006
CLGN	3.13	2.42	5.07	4.05	1.05	0.000006	0.000050
CEP170	42.70	40.82	27.84	27.41	-0.28	0.000213	0.001335
CNTNAP1	46.27	48.69	46.27	52.65	0.37	0.000004	0.000030
NFE2L2	51.17	48.13	34.76	32.50	-0.26	0.003598	0.017201
MYPN	2.21	2.21	0.20	0.11	-3.96	0.000000	0.000000

RPS28	196.77	205.58	112.61	111.24	-0.47	0.000026	0.000192
ATXN10	65.70	59.73	41.96	35.99	-0.44	0.000011	0.000086
QPRT	4.46	4.46	12.95	12.20	1.92	0.000000	0.000000
CAMK2D	22.27	22.22	13.37	11.03	-0.47	0.000019	0.000144
ABCC5	12.78	12.68	13.02	13.85	0.46	0.000005	0.000041
NOP2	52.66	54.81	26.40	27.30	-0.81	0.000000	0.000000
SSPO	0.28	0.68	0.35	1.30	1.68	0.000478	0.002813
RPL27	444.06	468.81	289.23	266.22	-0.34	0.000044	0.000306
CELSR3	0.91	0.62	0.82	0.98	0.74	0.009878	0.041712
UCK2	24.68	26.89	11.40	10.82	-0.92	0.000000	0.000000
AP3D1	63.98	79.39	42.11	46.84	-0.31	0.000293	0.001795
ELOVL1	93.62	97.84	115.59	112.42	0.57	0.000000	0.000000
OS9	179.96	174.74	219.11	220.26	0.64	0.000000	0.000000
GTPBP4	44.09	40.18	22.43	22.26	-0.63	0.000000	0.000000
GSDMD	18.12	19.87	6.69	8.51	-1.11	0.000000	0.000000
TCEA1	21.69	20.93	27.80	26.20	0.64	0.000000	0.000000
AC068831.8	3.22	3.32	0.25	0.25	-3.39	0.000000	0.000000
SLC25A37	15.25	14.82	14.51	14.33	0.33	0.001422	0.007504
VCAN	141.89	136.39	320.62	307.49	1.66	0.000000	0.000000
MYO1B	52.32	47.94	37.79	35.91	-0.24	0.001111	0.006012
EDEM2	18.32	19.47	20.23	21.15	0.47	0.000219	0.001373
SLC35A5	11.93	9.10	15.50	12.79	0.69	0.000016	0.000121
ICMT	24.36	20.89	13.41	11.58	-0.56	0.000000	0.000001
PPP1R13L	7.43	7.78	6.50	8.75	0.77	0.001064	0.005782
HMGB1	206.33	202.44	72.51	63.05	-1.12	0.000000	0.000000
ZFP91	13.81	12.27	7.47	6.67	-0.55	0.000000	0.000004
MANSC1	1.88	1.38	3.15	3.20	1.27	0.000027	0.000199
UBA7	33.27	34.92	19.39	25.96	-0.49	0.000559	0.003237
FABP5	35.64	33.73	11.88	7.57	-1.48	0.000000	0.000000
RRP9	17.86	20.57	6.25	4.84	-1.46	0.000000	0.000000
ROBO1	38.25	31.88	67.10	59.62	1.09	0.000000	0.000000
ACAN	1.60	1.24	0.07	0.12	-3.24	0.000000	0.000000
MIA3	55.57	45.97	49.09	49.09	0.41	0.000000	0.000002
PRKDC	98.90	95.86	35.75	33.54	-1.07	0.000000	0.000000
TRNP1	8.17	9.49	4.62	4.78	-0.70	0.001448	0.007633
LAYN	31.67	30.18	13.68	13.74	-0.85	0.000000	0.000000
RHOJ	5.93	7.07	11.55	11.04	1.00	0.000000	0.000000
ZFHX4	6.65	6.20	3.24	3.65	-0.55	0.000000	0.000001
SUN2	57.54	59.36	58.22	61.60	0.35	0.000010	0.000082
ERBIN	16.32	16.54	11.89	10.27	-0.50	0.000001	0.000008
ATAD2	11.01	10.51	1.18	1.17	-3.05	0.000000	0.000000
SORT1	11.81	11.69	17.68	16.04	0.86	0.000000	0.000000
POLR2L	48.26	55.92	18.26	20.46	-1.07	0.000000	0.000000

KRT8	15.62	19.02	21.28	20.55	0.68	0.000000	0.000003
KCNH1	0.56	0.65	4.17	3.43	2.58	0.000000	0.000000
METTL7A	2.42	2.76	4.96	6.83	1.02	0.000008	0.000067
MRPL3	39.24	40.63	19.81	17.47	-0.81	0.000000	0.000000
PON2	48.77	45.64	62.46	53.23	0.63	0.000000	0.000000
CPEB2	5.69	4.96	2.97	3.86	-0.36	0.011279	0.046835
GAB2	2.36	2.53	3.04	3.15	0.64	0.000154	0.000993
KLHL24	1.40	1.13	1.97	2.01	1.59	0.000000	0.000000
GREM1	575.11	559.12	82.61	68.03	-2.57	0.000000	0.000000
SLC2A3	28.79	25.96	64.90	64.06	1.47	0.000000	0.000000
MXD3	8.16	8.09	3.90	3.78	-0.98	0.000067	0.000457
HIPK2	13.02	12.93	5.42	5.23	-0.87	0.000000	0.000000
FANCI	12.86	13.01	1.96	1.41	-3.39	0.000000	0.000000
DCBLD1	34.95	34.35	75.60	67.45	1.39	0.000000	0.000000
DMWD	22.14	23.44	23.68	27.14	0.57	0.000000	0.000002
ENPP1	8.74	8.53	3.98	3.61	-1.02	0.000000	0.000000
CDK17	19.59	17.16	10.38	8.25	-0.74	0.000000	0.000000
GNA12	22.49	22.33	10.37	10.98	-0.75	0.000000	0.000000
ZNF146	30.86	28.91	18.84	18.54	-0.28	0.004397	0.020541
HMGB2	58.54	57.05	4.77	3.59	-3.53	0.000000	0.000000
TGFB1	72.83	78.16	108.31	112.74	0.87	0.000000	0.000000
GPATCH4	30.47	30.28	9.32	9.14	-1.25	0.000000	0.000000
MRC2	235.92	256.73	393.31	442.06	1.13	0.000000	0.000000
ACAD11	10.02	11.16	18.73	16.71	1.04	0.000000	0.000000
COA7	7.21	6.50	2.51	2.44	-0.90	0.000007	0.000053
CRKL	10.15	10.23	6.80	5.66	-0.41	0.000489	0.002872
HJURP	21.34	18.36	1.99	0.95	-4.43	0.000000	0.000000
FNDC3B	33.31	30.99	35.01	40.88	0.54	0.000000	0.000000
PUF60	78.55	86.11	49.85	55.98	-0.37	0.000553	0.003205
ST3GAL1	14.57	14.60	25.67	25.05	1.13	0.000000	0.000000
TAOK3	15.72	15.30	11.62	11.40	-0.35	0.003924	0.018570
PLCB3	19.21	21.16	18.03	20.74	0.33	0.001670	0.008676
PRSS12	97.42	94.10	51.96	50.10	-0.50	0.000000	0.000000
PLXNB1	49.53	47.61	52.83	47.77	0.47	0.000000	0.000001
NOL7	19.62	18.13	10.95	9.84	-0.55	0.000761	0.004275
BEX3	114.74	114.03	64.45	59.17	-0.55	0.000000	0.000002
POLH	7.30	7.08	7.68	7.23	0.35	0.003085	0.015025
C8orf58	16.81	18.03	21.40	24.10	0.73	0.000000	0.000001
PDK1	5.56	3.91	5.96	6.56	0.79	0.000000	0.000004
CNBP	70.30	68.67	34.95	30.26	-0.77	0.000000	0.000000
CAVIN3	32.44	34.24	20.59	18.65	-0.47	0.003375	0.016265
RNF103	5.94	4.62	8.56	7.43	0.96	0.000000	0.000000
UBE2V1	51.56	53.29	28.89	27.89	-0.65	0.000000	0.000000

SERPING1	0.38	0.46	1.71	1.73	2.54	0.000009	0.000073
LRRC32	13.45	12.90	40.63	42.97	1.99	0.000000	0.000000
TOMM6	48.69	53.44	25.59	24.91	-0.65	0.000361	0.002170
MATR3	111.64	101.55	61.42	55.24	-0.54	0.000000	0.000000
POLR3H	19.93	21.73	11.41	13.11	-0.50	0.000028	0.000207
NSUN2	30.39	30.33	16.43	17.13	-0.66	0.000000	0.000000
ZNHIT6	4.59	4.72	2.90	2.46	-0.46	0.002491	0.012403
TACC3	43.73	43.96	3.89	3.98	-3.17	0.000000	0.000000
CSK	30.06	33.21	15.34	19.90	-0.46	0.000368	0.002209
SCARF2	33.48	32.01	36.44	39.49	0.37	0.000103	0.000689
STK10	10.26	10.59	14.31	17.35	0.70	0.000000	0.000000
ADCY6	30.34	31.42	29.03	32.28	0.31	0.000143	0.000929
SNTB1	2.68	2.71	4.16	3.46	0.86	0.000002	0.000016
DCLK2	13.19	12.62	7.20	7.01	-0.53	0.000016	0.000122
ARSJ	32.88	29.75	22.83	19.53	-0.22	0.011906	0.049149
NR2C1	11.93	10.44	15.05	11.39	0.55	0.001785	0.009213
BACH1	5.75	6.72	6.59	6.01	0.36	0.008581	0.036883
PMAIP1	9.30	8.43	11.85	9.27	0.58	0.001022	0.005583
SCAMP4	32.13	34.95	32.28	35.60	0.32	0.002470	0.012309
IGFBP7	151.05	146.76	519.01	468.31	2.07	0.000000	0.000000
UQCRFS1	11.89	12.15	6.52	5.60	-0.65	0.000005	0.000041
KCNH2	1.00	1.03	8.69	8.06	3.49	0.000000	0.000000
CREB5	6.98	7.42	2.53	2.42	-0.87	0.000000	0.000004
CALM1	119.36	113.37	75.27	61.52	-0.29	0.002184	0.011034
PASK	4.82	5.68	0.85	1.25	-2.36	0.000000	0.000000
DNAJA1	74.61	69.67	46.21	41.56	-0.42	0.000001	0.000006
MT2A	432.35	503.58	79.46	79.06	-2.12	0.000000	0.000000
ARL6IP5	56.99	54.22	61.36	54.72	0.40	0.000004	0.000031
PTPN13	6.08	6.39	10.79	9.71	1.04	0.000000	0.000000
PRR13	46.78	48.25	29.73	27.72	-0.38	0.004444	0.020725
PIF1	26.68	21.76	3.18	5.61	-2.05	0.000000	0.000000
ACOT7	34.82	41.14	17.27	17.74	-0.78	0.000000	0.000000
ADSL	35.01	36.44	16.97	18.63	-0.68	0.000000	0.000001
PTPN11	32.52	32.64	15.78	15.41	-0.69	0.000000	0.000000
LITAF	33.98	34.60	10.99	10.26	-1.35	0.000000	0.000000
FDXR	14.84	16.53	19.81	20.65	0.70	0.000001	0.000005
RPS6KA4	17.50	16.80	8.42	8.20	-0.91	0.000000	0.000000
PLXDC2	4.89	4.06	35.33	29.85	3.27	0.000000	0.000000
C6orf89	26.97	24.47	30.57	27.47	0.38	0.000012	0.000093
AOPEP	10.39	12.17	22.57	21.25	1.18	0.000000	0.000000
ANO10	35.53	34.32	47.03	41.33	0.65	0.000000	0.000000
PANX1	20.44	18.42	23.65	21.60	0.56	0.000000	0.000001
HNRNPC	245.68	244.03	120.79	107.39	-0.77	0.000000	0.000000

ARHGAP17	33.79	34.04	20.46	17.68	-0.68	0.000000	0.000000
PSMB2	37.24	39.06	23.50	24.55	-0.36	0.000401	0.002392
MXRA8	344.92	357.76	314.50	353.28	0.30	0.000014	0.000108
SLC7A5	116.49	117.24	135.93	136.04	0.55	0.000000	0.000000
PUM3	38.81	42.47	22.90	22.06	-0.65	0.000000	0.000000
PACSIN2	25.10	29.21	17.31	17.15	-0.33	0.002042	0.010381
ZNF862	5.30	4.32	7.00	5.50	1.00	0.000000	0.000000
TPGS2	49.67	48.98	21.86	20.74	-0.78	0.000000	0.000000
ZBTB2	7.02	6.09	3.34	2.65	-0.80	0.000050	0.000350
CNIH3	0.70	1.27	5.30	4.58	3.01	0.000000	0.000000
TRPV2	12.98	13.87	13.90	15.81	0.68	0.000001	0.000008
HMCN1	13.54	12.90	24.36	23.90	1.22	0.000000	0.000000
SCUBE3	13.72	12.92	8.81	9.01	-0.24	0.006771	0.029942
LCNL1	1.28	1.26	2.33	2.49	1.24	0.000004	0.000036
CDK5RAP2	17.32	16.56	7.38	6.79	-1.08	0.000000	0.000000
ABCC3	13.27	13.86	3.92	3.45	-1.89	0.000000	0.000000
SCRN2	11.57	11.76	5.29	5.33	-0.68	0.003868	0.018333
CIT	11.08	9.35	0.38	0.86	-4.00	0.000000	0.000000
EIF3E	109.42	113.39	73.55	65.50	-0.29	0.001039	0.005654
IFT172	11.21	12.64	7.28	7.76	-0.47	0.000620	0.003558
RCN3	219.09	217.47	199.54	202.07	0.22	0.001655	0.008610
ABHD16A	26.28	26.03	32.29	32.68	0.72	0.000000	0.000000
RPL7L1	34.26	31.08	17.80	16.02	-0.69	0.000000	0.000000
DGKZ	37.51	37.72	22.08	23.81	-0.36	0.000992	0.005443
CHCHD2	103.21	104.74	62.21	60.21	-0.42	0.000123	0.000807
SLC6A8	28.83	27.75	29.56	27.92	0.36	0.000249	0.001544
CACNA2D1	20.25	17.60	31.36	21.05	0.64	0.000000	0.000000
AFF4	18.66	15.39	16.13	12.96	0.21	0.007825	0.034031
MCM7	72.11	74.94	14.65	14.82	-2.25	0.000000	0.000000
EIF1AX	20.42	20.89	10.08	7.90	-0.81	0.000000	0.000000
ANKRD1	1.23	1.27	3.37	3.46	1.79	0.000000	0.000000
TNPO1	36.79	38.32	23.38	20.66	-0.42	0.000000	0.000003
EXTL2	17.48	17.13	24.83	21.04	0.75	0.000000	0.000000
MYO9B	57.39	58.87	37.72	41.48	-0.22	0.003129	0.015218
DTX3	13.86	14.02	16.55	16.77	0.58	0.000107	0.000710
RAB5C	64.04	65.62	43.39	40.05	-0.27	0.004677	0.021651
PRPF8	122.25	118.97	57.89	71.95	-0.69	0.000000	0.000000
HNRNPM	123.13	120.25	60.33	50.69	-0.82	0.000000	0.000000
IER5	8.30	7.90	4.32	4.09	-0.61	0.000020	0.000149
NLN	11.03	9.93	4.82	4.54	-0.96	0.000000	0.000000
STK38L	5.61	5.43	14.21	13.60	1.61	0.000000	0.000000
MID1	12.77	13.10	6.15	5.28	-0.95	0.000000	0.000000
RPL23	459.00	447.37	261.59	238.00	-0.49	0.000000	0.000000

KCNK2	16.54	17.14	3.64	2.50	-1.88	0.000000	0.000000
CREB3L1	157.00	156.29	180.43	185.66	0.56	0.000000	0.000000
GTF3A	27.88	26.10	13.81	12.34	-0.71	0.000005	0.000041
NDRG1	15.78	17.35	18.91	20.41	0.80	0.000000	0.000000
DOP1B	3.82	3.84	2.23	2.59	-0.42	0.007959	0.034544
PROS1	1.49	1.43	2.94	2.51	1.25	0.000001	0.000011
FOXRED2	4.50	4.63	1.28	1.51	-1.31	0.000000	0.000000
PLEKHH2	1.63	1.54	5.77	5.10	1.69	0.000000	0.000000
BCAP31	112.23	124.16	76.60	76.91	-0.29	0.001251	0.006701
SRPX2	42.98	39.41	59.87	60.02	0.74	0.000000	0.000000
PGAP1	3.34	2.82	3.16	3.20	0.45	0.000863	0.004788
ATP5F1A	171.71	175.22	93.11	86.06	-0.64	0.000000	0.000000
DOCK5	9.02	8.12	4.05	4.32	-0.68	0.000000	0.000000
NOP16	23.01	22.39	9.37	11.16	-0.87	0.000007	0.000053
FBN1	340.43	346.79	538.45	553.54	1.08	0.000000	0.000000
LPP	10.32	10.28	11.21	12.37	0.38	0.000000	0.000004
FRMD5	1.90	1.46	2.56	2.83	1.00	0.000025	0.000182
GNAI2	167.03	179.30	106.22	106.01	-0.35	0.000000	0.000004
PRRG1	4.51	4.49	6.16	4.63	0.60	0.000500	0.002928
TMEM176B	0.12	0.23	1.64	1.62	3.66	0.000020	0.000150
MVP	129.15	137.28	85.93	94.87	-0.27	0.002603	0.012910
SLC43A3	20.68	17.80	4.17	3.64	-1.93	0.000000	0.000000
TMEM94	38.22	35.55	34.87	39.42	0.27	0.004274	0.020035
PPP1R12A	30.84	29.22	31.02	31.98	0.34	0.000206	0.001294
SUMO3	27.59	26.77	13.50	11.49	-0.79	0.000000	0.000000
VARS1	65.67	72.11	37.58	43.96	-0.57	0.000000	0.000000
LBR	20.95	19.38	11.10	10.77	-0.62	0.000000	0.000000
OLFML3	155.48	165.30	198.15	204.97	0.67	0.000000	0.000000
ZNF224	8.52	8.38	8.57	9.53	0.48	0.001615	0.008424
RSL1D1	48.98	50.92	22.95	22.18	-0.70	0.000000	0.000000
SCD5	4.18	3.61	7.84	7.36	1.31	0.000000	0.000000
AKT1	83.37	80.51	43.46	46.92	-0.50	0.000000	0.000000
RBCK1	53.49	56.71	64.14	73.54	0.62	0.000000	0.000000
KIAA1549L	4.09	3.87	1.46	1.17	-1.23	0.000000	0.000000
KIF5B	34.13	33.56	22.37	19.94	-0.34	0.000011	0.000088
MAP2K3	27.75	27.68	6.00	4.03	-2.39	0.000000	0.000000
GCNT4	0.31	0.32	1.02	0.90	1.96	0.000000	0.000002
KIRREL3	7.05	7.56	2.46	1.87	-1.63	0.000000	0.000000
NOLC1	44.57	42.28	17.59	15.90	-1.06	0.000000	0.000000
RPL36A	408.41	395.96	244.91	216.52	-0.40	0.000031	0.000224
UNC5B	7.59	7.74	38.13	43.06	2.75	0.000000	0.000000
TMEM176A	0.06	0.00	1.47	1.41	6.01	0.000215	0.001347
CASK	9.86	8.95	12.34	12.19	0.65	0.000000	0.000000

ARSA	20.50	20.70	21.73	24.92	0.45	0.000289	0.001772
DENND3	18.35	17.74	5.89	8.93	-1.19	0.000000	0.000000
RAI14	50.95	51.41	28.26	26.82	-0.52	0.000000	0.000000
CHD4	82.67	81.32	57.25	60.79	-0.17	0.008674	0.037216
DYNLL2	9.07	8.32	4.96	4.81	-0.50	0.000011	0.000083
ALPK2	32.23	29.83	40.58	41.10	0.64	0.000000	0.000000
C1QBP	86.76	86.34	35.97	30.22	-1.06	0.000000	0.000000
METRNL	28.07	27.45	15.78	16.57	-0.50	0.000807	0.004508
KHSRP	116.68	127.17	52.64	55.36	-0.77	0.000000	0.000000
MRPL12	34.76	35.71	13.41	13.70	-1.03	0.000000	0.000000
HYAL2	45.89	46.84	22.74	25.59	-0.63	0.000000	0.000000
PTGS2	1.59	1.26	3.26	3.89	1.40	0.000000	0.000000
PALM2AKAP2	39.95	39.39	22.70	22.27	-0.58	0.000000	0.000000
CYTOR	37.08	35.29	16.61	14.47	-0.69	0.000030	0.000215
SLC35E2B	21.19	21.46	19.02	20.79	0.24	0.003537	0.016930
PPP1CA	59.86	65.31	32.20	32.98	-0.62	0.000000	0.000000
SLC6A9	17.53	18.18	41.13	47.06	1.58	0.000000	0.000000
ARMC6	15.46	19.06	6.79	7.49	-0.86	0.000000	0.000002
CCT4	64.04	60.26	29.05	25.98	-0.84	0.000000	0.000000
RPL36	185.79	191.43	119.43	107.35	-0.40	0.000088	0.000595
GALC	5.49	4.56	5.20	5.40	0.46	0.006069	0.027212
TRO	37.97	36.05	38.24	44.98	0.53	0.000000	0.000002
LAMP2	55.03	53.04	60.93	53.40	0.32	0.000089	0.000603
GPSM1	63.35	65.46	41.68	47.06	-0.23	0.009562	0.040595
POFUT1	33.37	31.82	18.53	18.73	-0.54	0.000000	0.000000
GRPR	1.88	2.08	0.17	0.37	-2.53	0.000001	0.000005
UNC5C	0.77	0.65	1.05	1.27	1.17	0.000016	0.000118
CARD10	8.01	6.27	3.21	3.56	-0.72	0.000134	0.000873
PPARD	25.05	26.65	13.75	14.67	-0.52	0.000000	0.000003
ARHGEF12	20.00	17.77	13.37	12.68	-0.20	0.011086	0.046118
TGFB2	14.79	14.34	17.49	15.50	0.48	0.000002	0.000021
PEBP1	66.54	67.17	26.82	25.78	-1.00	0.000000	0.000000
AP1B1	28.07	31.56	15.88	16.98	-0.57	0.000000	0.000000
CD46	51.46	46.67	55.95	44.68	0.33	0.000036	0.000253
LRP4	9.03	8.81	12.66	13.83	0.96	0.000000	0.000000
AP5Z1	30.26	34.72	33.98	42.45	0.65	0.000000	0.000000
SNU13	48.12	50.59	27.53	26.28	-0.86	0.000000	0.000000
XPO6	74.38	71.92	33.25	36.01	-0.77	0.000000	0.000000
NORAD	31.41	30.41	31.16	27.50	0.26	0.000868	0.004813
TCF12	53.72	48.33	26.66	21.98	-0.73	0.000000	0.000000
ERAP1	21.27	23.93	25.42	24.50	0.43	0.000007	0.000055
ENTPD6	49.86	56.92	53.01	52.13	0.33	0.000250	0.001551
ADAMTSL4	8.48	8.68	3.43	4.53	-0.76	0.000113	0.000749

NUSAP1	33.26	33.62	1.62	1.32	-4.27	0.000000	0.000000
COL24A1	3.21	1.49	4.58	3.88	1.76	0.000000	0.000000
ETF1	32.13	29.01	19.32	16.76	-0.50	0.000000	0.000004
RPS9	414.54	431.16	244.27	253.70	-0.40	0.000000	0.000003
EMP2	8.61	7.11	0.92	1.12	-2.66	0.000000	0.000000
IKBKB	15.92	14.23	20.15	16.84	0.45	0.000041	0.000292
SYNE2	5.74	5.24	2.30	3.50	-1.00	0.000000	0.000000
FOSL2	23.29	23.70	13.07	13.30	-0.58	0.000000	0.000000
LAMA2	14.83	13.74	15.20	14.19	0.42	0.000000	0.000000
MMP14	322.45	336.84	788.34	822.15	1.63	0.000000	0.000000
SH3BP4	28.98	28.74	13.61	13.18	-0.78	0.000000	0.000000
PDGFRL	15.60	15.78	25.07	21.16	0.89	0.000000	0.000000
TRIM22	24.43	24.44	50.80	44.53	1.27	0.000000	0.000000
RPS16	387.57	419.98	218.36	208.13	-0.51	0.000000	0.000000
NARS1	76.49	73.82	89.42	74.72	0.32	0.000183	0.001162
NHP2	50.21	53.96	18.72	19.64	-1.03	0.000000	0.000000
VLDLR	11.03	10.36	41.49	37.23	2.15	0.000000	0.000000
RNF122	2.27	2.61	3.28	3.60	0.84	0.005169	0.023607
MDC1	15.90	16.69	7.95	8.36	-0.78	0.000000	0.000000
CYFIP2	3.43	3.70	4.90	5.62	1.05	0.000000	0.000000
SAMD11	5.76	8.04	13.69	12.46	1.03	0.000000	0.000000
RPL14	197.12	205.27	116.93	132.64	-0.49	0.000000	0.000000
ARMC9	12.64	15.71	15.26	19.80	0.64	0.000000	0.000002
BRIX1	20.99	23.55	10.01	9.77	-0.83	0.000000	0.000002
RPS27A	315.34	300.25	228.07	205.04	-0.24	0.003065	0.014945
SSR4	165.17	165.57	183.70	190.55	0.53	0.000000	0.000000
KIAA0930	20.58	22.95	28.08	28.21	0.64	0.000000	0.000000
ITGA5	351.36	369.60	630.50	654.76	1.18	0.000000	0.000000
PPIB	914.34	905.25	1,028.44	997.39	0.50	0.000000	0.000000
STX16	36.02	38.75	38.12	35.65	0.33	0.000038	0.000270
GATAD2A	24.16	26.52	13.91	12.74	-0.63	0.000000	0.000000
ESPL1	12.07	11.61	0.57	0.93	-4.15	0.000000	0.000000
KLHL18	7.29	8.68	6.90	8.30	0.38	0.005494	0.024962
NOL6	33.75	34.86	12.33	12.11	-1.20	0.000000	0.000000
DKC1	33.64	34.13	13.99	15.85	-1.05	0.000000	0.000000
TMEM200A	19.62	18.56	20.17	17.80	0.32	0.002325	0.011656
KIF11	12.28	11.70	0.86	0.69	-3.62	0.000000	0.000000
RAB34	112.77	121.08	60.19	67.49	-0.63	0.000000	0.000000
ZNF789	9.59	11.14	27.25	27.98	1.87	0.000000	0.000000
SLC25A23	6.85	7.18	8.54	9.11	0.60	0.000044	0.000311
ICAM1	43.25	46.01	111.80	118.09	1.70	0.000000	0.000000
RPL31	476.58	478.93	272.08	267.77	-0.40	0.000003	0.000023
DDR1	18.50	19.90	22.22	23.62	0.58	0.000000	0.000000

MAPKAP1	31.62	31.57	21.53	17.41	-0.35	0.001266	0.006768
COPZ1	55.38	58.25	41.37	39.73	-0.28	0.005439	0.024733
CLIC1	209.04	210.59	122.12	115.84	-0.48	0.000000	0.000000
STT3B	81.71	70.53	74.76	63.96	0.35	0.000011	0.000082
GPC6	19.60	17.82	35.46	32.67	1.19	0.000000	0.000000
DNAJB6	47.33	48.24	43.85	35.70	0.23	0.012002	0.049468
PSMD3	61.62	67.09	40.35	40.36	-0.37	0.000027	0.000196
TMEM158	29.64	27.95	16.79	17.41	-0.41	0.000725	0.004096
MRTO4	24.28	27.44	8.64	8.96	-1.17	0.000000	0.000000
AC138969.1	71.35	68.96	83.46	80.96	0.52	0.000000	0.000000
TAF15	63.87	64.54	46.03	42.71	-0.35	0.000196	0.001238
CLN5	13.28	12.66	16.51	13.01	0.49	0.000455	0.002686
RPS14	292.31	317.22	180.03	178.63	-0.41	0.000001	0.000008
ARHGEF17	64.47	71.24	72.09	86.20	0.53	0.000000	0.000000
RPL38	289.03	298.35	186.20	154.19	-0.40	0.000542	0.003144
LIMS2	13.21	15.43	33.17	39.30	1.71	0.000000	0.000000
NFKBIZ	10.94	8.62	12.69	12.71	0.63	0.000007	0.000059
C7orf50	35.00	39.09	20.38	23.11	-0.67	0.000005	0.000045
RFX7	5.74	5.65	7.84	7.36	0.68	0.000000	0.000000
SEC61A1	391.78	397.42	416.84	419.04	0.42	0.000000	0.000000
NDC1	14.78	12.36	3.81	2.77	-1.86	0.000000	0.000000
FLT1	2.65	2.60	4.36	3.26	0.68	0.000889	0.004918
SEMA4C	19.23	23.53	20.91	23.07	0.32	0.003692	0.017569
USP22	47.51	51.37	28.98	30.93	-0.37	0.000000	0.000002
MT-ND4L	6,924.37	6,626.35	7,373.90	6,942.36	0.50	0.000000	0.000000
MSH5	19.10	18.30	6.35	4.48	-1.31	0.000000	0.000000
NCSTN	109.00	109.43	117.11	109.94	0.36	0.000000	0.000001
KARS1	68.79	69.63	39.56	34.57	-0.60	0.000000	0.000000
IFT57	18.04	17.97	7.81	6.48	-1.02	0.000000	0.000001
POLR2E	98.62	112.78	41.62	49.16	-0.80	0.000000	0.000000
AGPAT4	4.65	3.64	5.46	5.41	0.76	0.000000	0.000000
HP1BP3	90.78	85.69	54.55	56.73	-0.32	0.000030	0.000218
MSN	202.21	204.89	131.17	127.39	-0.32	0.000000	0.000000
CTSO	2.10	2.03	2.96	2.83	0.83	0.001106	0.005990
CLUH	41.42	42.23	14.36	17.10	-1.07	0.000000	0.000000
BCR	9.80	11.80	6.84	5.86	-0.36	0.005448	0.024767
FTX	11.89	11.42	19.29	19.49	1.10	0.000000	0.000000
YIPF3	98.61	97.42	126.54	124.97	0.70	0.000000	0.000000
KRT7	5.83	6.49	15.73	20.59	2.09	0.000000	0.000000
MEGF8	21.39	23.45	24.13	25.95	0.47	0.000000	0.000000
DIRAS1	3.74	4.26	1.54	1.74	-0.94	0.000090	0.000605
ATP5MC3	69.18	72.15	38.80	36.87	-0.49	0.000038	0.000268
MAGI1	5.12	6.39	6.77	7.17	0.64	0.000000	0.000002

PTPRB	0.73	0.72	1.17	1.22	0.99	0.000137	0.000895
FAM234A	42.53	42.51	38.96	41.47	0.27	0.003612	0.017259
ST13	90.37	79.57	48.52	44.63	-0.38	0.000043	0.000304
NIN	14.89	13.83	7.17	6.24	-0.77	0.000000	0.000000
OSMR	61.10	58.13	68.28	63.52	0.48	0.000000	0.000000
COPS2	32.88	28.48	22.51	19.41	-0.30	0.010507	0.044056
MSANTD3	19.56	20.40	10.88	10.27	-0.54	0.000589	0.003395
AMD1	13.06	11.97	8.68	6.96	-0.43	0.000709	0.004013
PARK7	116.93	112.02	62.29	54.45	-0.65	0.000000	0.000000
BMP5	0.04	0.01	0.96	0.73	5.38	0.000000	0.000000
MPP1	17.66	18.57	8.56	7.73	-0.92	0.000000	0.000000
TPR	29.50	25.61	17.67	14.97	-0.47	0.000000	0.000000
ATAD3A	29.13	33.15	15.14	17.78	-0.63	0.000000	0.000000
HBP1	8.82	10.09	9.96	9.51	0.48	0.004098	0.019315
AHCY	50.22	53.91	27.69	28.29	-0.54	0.000000	0.000000
SYDE1	27.88	28.81	17.16	17.88	-0.42	0.000074	0.000507
SMS	70.04	70.83	20.64	17.33	-1.55	0.000000	0.000000
HSPA1A	20.88	21.92	21.48	21.31	0.35	0.001154	0.006217
COL15A1	17.43	17.50	162.80	165.17	3.60	0.000000	0.000000
GNA11	23.34	20.98	15.17	14.04	-0.38	0.000114	0.000755
RPL23A	365.84	363.34	190.42	171.28	-0.66	0.000000	0.000000
PPP1CC	35.35	35.79	21.16	18.66	-0.64	0.000000	0.000000
MLLT11	12.89	12.51	14.98	12.87	0.47	0.000377	0.002257
CTSZ	55.98	52.88	52.86	51.89	0.29	0.001914	0.009814
AKR1B1	67.37	76.70	27.89	28.18	-1.04	0.000000	0.000000
AXL	224.33	221.31	95.71	102.23	-0.84	0.000000	0.000000
SAE1	73.35	73.41	30.28	27.03	-0.96	0.000000	0.000000
CDC42	73.93	77.02	55.87	46.49	-0.25	0.010705	0.044784
RRS1	16.27	19.05	5.22	5.31	-1.40	0.000000	0.000000
RPL10A	467.41	504.35	236.54	225.38	-0.68	0.000000	0.000000
PERP	15.13	14.47	10.30	8.95	-0.29	0.009839	0.041583
SRXN1	18.94	19.68	9.22	8.52	-0.79	0.000000	0.000000
S100A6	630.77	670.09	194.08	195.90	-1.36	0.000000	0.000000
GPT2	22.00	24.58	27.68	30.42	0.61	0.000000	0.000000
MZT2B	47.88	57.41	22.90	22.04	-0.78	0.000005	0.000044
GPR107	22.53	19.54	23.08	21.96	0.57	0.000000	0.000000
FAP	88.48	88.54	85.18	78.33	0.31	0.000037	0.000265
TBC1D2B	17.76	17.76	20.37	21.29	0.46	0.000000	0.000001
CYP4V2	2.50	2.37	3.26	3.06	0.94	0.000000	0.000001
PTPRQ	1.91	1.44	0.43	0.54	-0.79	0.005174	0.023627
RPL35A	198.09	204.13	136.34	110.49	-0.34	0.002899	0.014207
AHSA1	83.38	79.54	62.45	47.25	-0.38	0.000531	0.003092
SAT1	20.70	18.44	29.62	27.93	0.87	0.000000	0.000001

HNRNPH1	370.23	363.13	269.07	270.42	-0.15	0.011059	0.046013
FOXP4	11.52	11.86	18.93	22.11	1.14	0.000000	0.000000
COL1A1	8,136.12	8,648.13	13,845.12	16,619.31	1.11	0.000000	0.000000
VPS13A	20.17	18.23	13.18	11.56	-0.49	0.000000	0.000000
CTSA	177.96	170.82	175.00	196.60	0.43	0.000000	0.000000
PSIP1	23.76	22.54	11.33	9.32	-0.94	0.000000	0.000000
KDELR2	107.82	99.55	101.72	91.13	0.19	0.011008	0.045864
CA12	29.09	28.86	9.51	7.89	-1.41	0.000000	0.000000
CEP350	6.89	5.47	4.73	2.86	-0.42	0.000417	0.002477
RNF187	26.03	25.72	36.17	37.98	0.79	0.000000	0.000000
NID2	386.94	389.84	412.86	385.36	0.45	0.000000	0.000000
CEP250	37.51	38.23	14.62	16.18	-1.08	0.000000	0.000000
PES1	44.81	47.59	22.78	25.88	-0.67	0.000000	0.000000
APP	457.50	465.00	567.19	566.85	0.64	0.000000	0.000000
C5orf15	31.92	30.78	46.91	39.83	0.69	0.000000	0.000000
PRICKLE1	3.45	2.76	3.76	3.74	0.63	0.001614	0.008420
KCNQ1OT1	0.43	0.38	0.42	0.40	0.35	0.003626	0.017305
TKT	307.96	311.24	123.68	137.09	-0.94	0.000000	0.000000
TGFBI	2,323.01	2,326.17	4,808.83	4,585.46	1.35	0.000000	0.000000
PCBP1	91.40	96.44	43.62	41.74	-0.80	0.000000	0.000000
CCDC85B	23.94	25.63	7.65	7.43	-1.37	0.000000	0.000000
MEG3	625.78	653.61	1,481.21	1,758.54	1.72	0.000000	0.000000
SLC6A15	0.52	0.86	3.39	3.23	3.21	0.000000	0.000000
CCNG1	33.12	30.58	34.17	33.03	0.34	0.000775	0.004344
KPNA4	7.76	7.62	4.93	4.40	-0.39	0.000214	0.001343
RHOBTB3	50.24	43.86	32.97	30.80	-0.45	0.000000	0.000000
ORAI2	12.55	14.18	8.24	7.95	-0.31	0.010542	0.044178
PRCP	52.11	50.88	32.37	29.71	-0.39	0.000005	0.000043
ADAM23	13.02	12.17	17.13	14.66	0.77	0.000000	0.000000
SQSTM1	138.41	145.27	75.99	81.14	-0.60	0.000000	0.000000
TRIB3	14.62	15.76	44.56	46.98	1.94	0.000000	0.000000
SPTBN1	57.36	53.89	53.74	53.44	0.24	0.000099	0.000661
CERS2	97.82	91.45	91.41	86.43	0.22	0.003666	0.017470
KIF18B	16.76	19.00	0.52	0.41	-4.73	0.000000	0.000000
UBQLN1	29.81	28.99	20.19	18.19	-0.26	0.004646	0.021544
ARHGAP6	0.65	0.89	1.65	1.34	1.52	0.000004	0.000037
RPL12	435.93	423.82	235.55	227.80	-0.51	0.000000	0.000000
HSPA9	118.71	118.27	129.41	117.02	0.27	0.000042	0.000297
RHOB	15.77	18.74	9.27	9.63	-0.53	0.000243	0.001509
SGCE	21.34	19.97	28.29	23.21	0.61	0.000002	0.000013
CORO6	8.98	8.14	8.92	9.68	0.49	0.003226	0.015644
POLR1E	22.54	24.02	5.03	5.10	-1.86	0.000000	0.000000
DUSP1	35.29	35.58	16.17	16.87	-0.76	0.000000	0.000000

CCDC86	23.06	22.96	6.26	7.42	-1.40	0.000000	0.000000
PTGDS	4.61	4.39	14.36	15.08	2.04	0.000000	0.000000
INHBA	51.56	50.26	32.11	32.85	-0.23	0.000826	0.004601
TENM3	25.98	25.44	24.51	21.86	0.22	0.002317	0.011625
TUBB4B	283.48	308.08	73.93	79.26	-1.60	0.000000	0.000000
ASS1	12.96	12.00	40.45	38.33	2.08	0.000000	0.000000
UBE3C	20.53	20.40	14.68	13.15	-0.31	0.000802	0.004479
GLIS2	5.43	6.47	6.47	7.93	0.62	0.000183	0.001158
PTGIS	1.53	0.70	6.42	7.90	3.14	0.000000	0.000000
CKB	10.88	12.45	18.85	20.89	0.86	0.000001	0.000010
RPL37A	548.19	625.55	336.94	328.59	-0.44	0.000001	0.000011
PRKCSH	395.35	400.32	348.81	387.05	0.29	0.000180	0.001141
AC092683.1	13.66	14.37	15.01	15.33	0.54	0.000147	0.000950
BUB1	13.42	15.21	0.48	0.40	-4.63	0.000000	0.000000
AP2B1	54.65	49.73	39.20	34.70	-0.19	0.008915	0.038135
ANXA5	598.18	592.98	399.68	351.20	-0.34	0.000000	0.000002
KDELR1	211.24	204.92	196.03	182.72	0.25	0.000337	0.002042
GPX8	90.87	80.40	95.18	79.57	0.40	0.000004	0.000033
SMC4	49.55	44.62	10.36	10.68	-1.88	0.000000	0.000000
FKBP10	756.51	811.03	952.52	1,037.99	0.77	0.000000	0.000000
KLF7	6.40	5.46	7.03	9.26	0.70	0.000000	0.000000
MAMDC2	4.49	4.05	24.47	21.51	2.76	0.000000	0.000000
DDX5	454.80	427.85	404.30	377.05	0.26	0.000007	0.000055
SLC1A4	15.65	15.81	43.85	44.43	1.78	0.000000	0.000000
CREB3L2	37.16	34.05	16.35	14.73	-0.85	0.000000	0.000000
GOLGA8A	28.09	25.91	41.25	41.11	0.85	0.000000	0.000000
CADM1	112.32	101.56	210.26	209.30	1.20	0.000000	0.000000
RPL21	363.49	362.81	222.02	191.62	-0.52	0.000000	0.000000
GLRX	62.91	59.03	23.08	22.28	-0.79	0.000000	0.000000
TMEM30A	73.30	68.24	93.79	82.74	0.61	0.000000	0.000000
AOX1	4.20	4.51	0.94	0.42	-2.78	0.000000	0.000000
MAPKBP1	16.20	18.83	17.80	23.68	0.59	0.000000	0.000000
PPP2CA	53.40	47.84	27.74	22.70	-0.59	0.000000	0.000000
PTPRS	54.30	55.91	54.03	52.74	0.30	0.000038	0.000267
ARNT2	6.71	6.10	3.07	3.35	-0.54	0.000096	0.000641
SCN8A	5.98	5.56	5.33	5.77	0.29	0.006761	0.029911
DLG1	17.65	14.14	26.90	22.65	0.90	0.000000	0.000000
RBMX	110.47	105.05	63.65	55.91	-0.45	0.000000	0.000000
RPN2	371.03	368.75	420.38	407.51	0.51	0.000000	0.000000
PPT1	39.80	33.43	21.94	18.23	-0.59	0.000000	0.000001
LMAN1	87.64	82.31	83.20	75.58	0.20	0.004371	0.020441
FOXC2	3.74	4.62	1.74	1.72	-0.94	0.000203	0.001276
POLDIP2	35.25	36.42	21.21	19.46	-0.51	0.000000	0.000004

RRP12	18.26	19.23	8.98	10.72	-0.72	0.000000	0.000000
IGF2BP2	41.60	37.38	25.23	26.68	-0.41	0.000010	0.000078
CCT6A	75.54	78.67	38.12	33.38	-0.83	0.000000	0.000000
DNMT1	67.12	65.33	15.78	17.23	-2.08	0.000000	0.000000
ARHGEF28	2.89	3.24	3.40	3.50	0.50	0.004383	0.020491
CPD	33.30	30.25	36.57	31.37	0.21	0.004692	0.021716
RGS2	5.12	4.87	8.52	8.70	1.05	0.000015	0.000114
ARPC1B	71.04	84.87	36.61	39.78	-0.65	0.000000	0.000000
TSPYL1	20.10	20.48	11.42	11.40	-0.49	0.000005	0.000038
SUMF1	16.82	16.01	8.76	9.24	-0.49	0.000854	0.004740
GAA	23.19	26.54	26.28	32.90	0.66	0.000000	0.000000
RND3	95.51	91.65	62.11	58.40	-0.30	0.000198	0.001247
RPL41	690.11	695.27	435.23	408.84	-0.36	0.000003	0.000028
EEF1B2	312.86	294.38	211.62	195.26	-0.26	0.000994	0.005454
IPO4	31.50	32.63	11.92	12.43	-1.06	0.000000	0.000000
C21orf58	5.07	8.65	1.31	1.70	-1.78	0.000000	0.000000
CCT7	143.09	145.86	70.63	64.61	-0.78	0.000000	0.000000
ZNF281	7.88	7.20	10.02	8.89	0.65	0.000000	0.000001
DNAJB4	20.96	17.77	11.21	10.47	-0.48	0.000711	0.004022
FAM111A	13.16	13.40	5.70	5.55	-0.93	0.000000	0.000000
ITGA2	35.69	30.68	36.85	33.13	0.40	0.000000	0.000004
APBB2	13.19	13.73	16.52	14.27	0.39	0.000256	0.001587
ERLIN1	32.21	30.73	12.20	11.48	-1.03	0.000000	0.000000
PIP5K1A	36.29	31.16	21.29	23.18	-0.27	0.002887	0.014157
SPSB1	11.00	10.83	11.87	14.00	0.65	0.000004	0.000032
RPL18	417.22	429.58	212.18	222.78	-0.58	0.000000	0.000000
FAT4	7.56	7.03	15.22	15.44	1.44	0.000000	0.000000
TXNL4A	37.29	37.68	23.69	20.38	-0.44	0.005850	0.026360
RBM39	129.74	123.30	137.83	135.20	0.41	0.000000	0.000000
KIF2C	24.16	23.23	0.81	1.18	-4.35	0.000000	0.000000
NME4	116.20	122.98	56.71	61.43	-0.65	0.000000	0.000000
CNTN3	4.97	5.88	7.08	4.74	0.47	0.006530	0.029063
IL16	0.20	0.47	0.67	0.53	1.78	0.000222	0.001387
PSMA7	119.85	121.95	70.57	68.55	-0.46	0.000001	0.000010
FKBP8	84.80	92.84	54.95	61.88	-0.27	0.007440	0.032518
SMARCC2	45.65	39.54	22.12	23.83	-0.58	0.000000	0.000000
NCR3LG1	3.17	2.83	3.82	3.62	0.65	0.000023	0.000172
PRDX5	91.55	102.14	111.41	112.12	0.57	0.000000	0.000000
METAP2	42.18	43.56	22.71	24.52	-0.52	0.000002	0.000016
BTG2	3.89	3.49	10.59	10.00	1.81	0.000000	0.000000
B3GNT9	9.41	9.69	9.56	9.86	0.36	0.010926	0.045553
BCAM	48.70	51.92	61.57	71.81	0.66	0.000000	0.000000
CAPN15	31.84	34.09	15.87	17.86	-0.65	0.000000	0.000000

B4GAT1	13.91	12.84	5.04	5.49	-1.00	0.000000	0.000000
MPP5	9.05	8.12	5.49	4.72	-0.35	0.007510	0.032799
KIF22	34.51	35.89	2.85	3.91	-3.18	0.000000	0.000000
PDLIM2	51.68	60.91	54.41	74.80	1.00	0.000000	0.000000
PDE5A	34.37	34.99	48.25	42.31	0.68	0.000000	0.000000
ZPR1	18.71	19.19	11.90	13.10	-0.41	0.002243	0.011285
SRSF1	87.42	82.73	41.01	34.82	-0.69	0.000000	0.000000
LEPROT	13.72	13.47	16.36	14.73	0.39	0.000411	0.002448
STARD7	25.73	24.08	15.86	13.74	-0.43	0.000072	0.000491
MRAS	11.57	12.33	10.55	11.97	0.49	0.000189	0.001195
PAPSS2	29.53	27.83	17.30	15.39	-0.48	0.000000	0.000002
LARP1	52.71	55.69	36.12	38.52	-0.24	0.000626	0.003581
POGLUT3	29.10	29.34	18.11	15.32	-0.53	0.000000	0.000000
EIF3J	38.70	37.34	23.42	20.90	-0.45	0.000133	0.000870
PSME4	25.04	27.05	18.89	17.57	-0.27	0.001457	0.007674
PABPC4	87.39	85.83	42.79	38.88	-0.84	0.000000	0.000000
ADCY3	34.76	31.03	21.20	22.40	-0.34	0.000615	0.003532
C20orf27	25.47	24.04	7.65	7.71	-1.43	0.000000	0.000000
SLC12A4	65.85	72.13	71.89	79.76	0.49	0.000000	0.000000
NEO1	23.42	21.43	23.28	22.06	0.28	0.001314	0.006994
POLE	26.53	24.15	6.88	4.48	-2.05	0.000000	0.000000
RAB7A	67.70	69.77	45.32	41.14	-0.39	0.000006	0.000047
CSTB	12.49	12.36	6.83	6.43	-0.52	0.001036	0.005644
RPL35	566.31	593.65	314.77	305.39	-0.52	0.000000	0.000000
PPIF	36.99	40.13	11.64	12.62	-1.41	0.000000	0.000000
PKP4	17.03	17.13	9.50	10.47	-0.41	0.000212	0.001328
SLC38A1	32.19	29.33	53.33	48.36	1.09	0.000000	0.000000
RPL26	583.97	586.06	350.84	291.35	-0.50	0.000000	0.000000
STOX2	0.24	0.41	0.45	0.78	1.75	0.000010	0.000077
PYGB	48.36	49.07	48.12	52.47	0.35	0.000002	0.000021
RPS18	1,013.28	1,082.21	579.09	547.77	-0.52	0.000000	0.000000
ABLIM1	2.80	2.30	4.03	4.49	0.93	0.000000	0.000000
AC138932.1	10.26	10.06	10.80	14.17	0.66	0.000000	0.000001
SLC2A10	13.33	12.91	16.62	15.85	0.69	0.000000	0.000000
YWHAH	41.26	42.79	15.38	16.02	-1.08	0.000000	0.000000
MAP7D1	70.54	76.97	48.11	45.20	-0.35	0.000022	0.000164
RPS11	677.58	725.04	425.72	432.38	-0.34	0.000004	0.000033
RIC8A	70.13	71.33	52.20	53.84	-0.26	0.008425	0.036334
RAD21	28.43	28.75	11.26	10.35	-1.03	0.000000	0.000000
SOD1	66.75	73.92	39.97	32.37	-0.60	0.000003	0.000022
IL7R	40.95	36.69	17.74	15.44	-1.00	0.000000	0.000000
PAICS	58.60	57.89	23.74	20.00	-1.05	0.000000	0.000000
STAT6	52.13	52.97	32.42	31.91	-0.42	0.000001	0.000009

BICD2	11.08	11.40	6.37	6.45	-0.46	0.000078	0.000532
STK40	15.46	16.02	14.21	15.84	0.29	0.008857	0.037899
RPS26	202.95	207.15	117.22	99.63	-0.54	0.000000	0.000002
B3GALT2	1.06	1.15	3.38	2.74	1.81	0.000000	0.000000
TROAP	24.88	27.75	0.77	1.00	-4.35	0.000000	0.000000
PBXIP1	27.44	28.94	64.13	65.12	1.54	0.000000	0.000000
COLEC12	3.04	2.56	1.08	1.05	-1.07	0.000001	0.000010
SELENON	69.77	68.80	36.68	39.39	-0.53	0.000000	0.000000
RPL10	779.40	832.92	406.57	407.33	-0.55	0.000000	0.000000
SERINC1	75.18	71.93	116.31	101.78	0.90	0.000000	0.000000
SEMA5A	9.76	8.83	13.58	13.37	0.85	0.000000	0.000000
SLC25A6	191.06	208.27	92.99	98.78	-0.72	0.000000	0.000000
NCAPD2	30.89	29.51	4.75	5.15	-2.53	0.000000	0.000000
RPL24	394.54	391.53	250.58	216.24	-0.36	0.000031	0.000224
SIK2	5.98	4.73	3.34	3.00	-0.58	0.000006	0.000051
SESN2	16.00	16.02	39.29	41.49	1.67	0.000000	0.000000
MOXD1	15.60	16.99	25.90	22.78	0.94	0.000000	0.000000
DRAP1	166.39	169.27	60.97	55.64	-1.16	0.000000	0.000000
MMP23A	0.84	1.72	5.06	4.26	2.22	0.000002	0.000014
S1PR2	6.79	6.55	6.74	7.09	0.39	0.007010	0.030860
TJP2	21.99	19.95	4.88	4.86	-1.77	0.000000	0.000000
NHS	1.04	1.63	1.71	1.75	1.07	0.000000	0.000001
NCS1	26.55	30.41	16.20	15.58	-0.57	0.000000	0.000001
PCBP4	33.65	41.01	37.55	42.54	0.47	0.000025	0.000181
CENPE	8.23	7.02	0.38	0.28	-4.29	0.000000	0.000000
ZNF37BP	6.50	5.24	2.97	3.35	-0.52	0.002220	0.011185
PLIN3	67.22	70.85	35.62	44.94	-0.51	0.000000	0.000002
HNRNPAB	79.44	78.81	33.30	31.59	-1.01	0.000000	0.000000
RAB6A	22.87	21.96	23.48	18.72	0.29	0.003205	0.015568
MALAT1	285.56	264.35	350.51	346.90	0.68	0.000000	0.000000
VCP	164.30	168.36	94.99	95.03	-0.51	0.000000	0.000000
TENT5B	4.40	4.15	1.25	1.14	-1.50	0.000000	0.000002
COL13A1	17.33	18.07	40.49	48.07	1.54	0.000000	0.000000
CALM2	285.94	275.62	167.97	147.12	-0.41	0.000000	0.000000
RPS8	519.95	546.10	287.34	270.86	-0.58	0.000000	0.000000
JAZF1	4.60	5.58	7.35	5.67	0.69	0.000073	0.000498
ABI3BP	6.16	7.67	42.13	33.10	2.72	0.000000	0.000000
APCDD1L	79.46	79.18	47.80	47.60	-0.40	0.000000	0.000000
SLC1A5	283.22	290.36	380.22	393.11	0.77	0.000000	0.000000
RPS17	561.19	569.24	388.02	349.87	-0.24	0.003019	0.014741
CDK4	82.77	96.87	37.75	39.43	-0.88	0.000000	0.000000
DDX17	117.18	115.67	122.08	118.83	0.37	0.000000	0.000000
PSME3	49.32	49.50	23.21	27.49	-0.59	0.000000	0.000000

ZHX3	11.46	12.55	5.58	5.49	-0.67	0.000000	0.000000
LONP1	55.01	59.40	70.14	76.90	0.66	0.000000	0.000000
RPL29	259.72	281.77	131.79	135.57	-0.68	0.000000	0.000000
AZIN1	24.66	23.55	17.25	15.14	-0.28	0.004266	0.020007
TSPAN6	7.40	6.87	11.64	9.61	0.79	0.000000	0.000000
CD63	814.26	773.92	802.00	771.56	0.32	0.000000	0.000000
FOXO3	7.96	7.58	5.05	4.74	-0.41	0.000294	0.001799
RPS3A	397.05	390.29	230.52	203.47	-0.50	0.000000	0.000000
RNF213	26.67	25.91	15.64	15.97	-0.28	0.000640	0.003652
RDH11	59.34	58.88	100.09	97.56	1.10	0.000000	0.000000
AFAP1	41.40	40.69	21.75	20.71	-0.63	0.000000	0.000000
RPS5	388.04	415.81	217.13	217.68	-0.53	0.000000	0.000000
MRVI1	7.44	7.52	17.30	18.16	1.50	0.000000	0.000000
PTPRK	31.08	27.89	27.21	26.31	0.22	0.004369	0.020435
ASCC3	19.87	22.07	23.02	21.98	0.33	0.000148	0.000958
B4GALT2	37.97	39.33	20.52	23.41	-0.49	0.000018	0.000135
RPS10	540.28	581.54	308.84	321.72	-0.47	0.000000	0.000000
TTC14	20.86	14.56	17.90	14.81	0.45	0.000036	0.000253
NUAK2	8.96	8.51	3.40	3.44	-1.01	0.000000	0.000000
PEAR1	36.71	36.16	13.48	15.54	-0.99	0.000000	0.000000
MIAT	1.22	1.09	4.63	4.90	2.40	0.000000	0.000000
NRIP1	7.58	6.26	3.29	3.43	-0.71	0.000000	0.000000
SDK1	1.24	1.18	2.46	3.06	1.54	0.000000	0.000000
EHD4	4.65	5.06	2.83	2.90	-0.43	0.003757	0.017849
SEC13	161.54	170.38	99.69	99.46	-0.40	0.000000	0.000002
IGF1R	19.01	16.46	15.16	16.08	0.22	0.005012	0.023001
MRGPRF	43.77	44.11	55.61	57.01	0.69	0.000000	0.000000
NT5E	138.68	131.71	41.87	38.11	-1.36	0.000000	0.000000
MIR4435-2HG	49.66	53.90	27.12	26.38	-0.46	0.000129	0.000846
FKBP11	43.36	44.27	42.95	40.95	0.31	0.006733	0.029798
SF3B1	110.65	116.28	110.59	106.87	0.25	0.000043	0.000305
ZMAT3	5.56	6.44	8.73	8.20	0.89	0.000000	0.000000
RPS3	700.54	739.39	397.05	396.35	-0.52	0.000000	0.000000
GSPT1	58.84	57.54	33.21	28.64	-0.65	0.000000	0.000000
JPT1	108.42	111.66	57.04	56.73	-0.68	0.000000	0.000000
SUMO2	72.22	67.44	44.11	31.40	-0.58	0.000001	0.000008
PIK3R3	6.87	6.49	7.46	6.91	0.32	0.010336	0.043398
KRT19	7.92	9.17	2.27	2.34	-1.57	0.000000	0.000001
SGSH	30.24	29.76	15.48	18.48	-0.48	0.000017	0.000129
SEC63	54.75	53.28	89.88	85.82	0.84	0.000000	0.000000
ADM	204.73	207.90	90.72	91.46	-0.84	0.000000	0.000000
PIGK	23.54	21.06	16.77	14.25	-0.46	0.000263	0.001622
OBSCN	21.01	19.36	24.77	26.99	0.75	0.000000	0.000000

BTAF1	14.23	14.11	15.99	14.50	0.42	0.000002	0.000017
EML5	1.55	1.67	0.22	0.35	-1.95	0.000000	0.000000
UHMK1	7.22	6.43	4.46	4.26	-0.31	0.006309	0.028194
SIPA1L3	9.08	10.89	3.00	4.13	-0.83	0.000000	0.000000
MYOCD	5.82	6.08	2.27	2.17	-1.27	0.000000	0.000000
GGH	22.98	23.39	7.99	5.75	-1.44	0.000000	0.000000
TLN2	8.70	7.50	3.39	6.07	-0.41	0.000193	0.001220
EIF4G1	249.47	257.69	137.93	141.32	-0.55	0.000000	0.000000
PDAP1	70.82	67.23	34.61	32.60	-0.70	0.000000	0.000000
ANLN	56.01	47.46	3.30	3.88	-3.88	0.000000	0.000000
PSPH	12.63	17.96	20.45	20.15	0.68	0.000017	0.000129
BSG	416.58	439.97	408.50	425.40	0.30	0.000004	0.000034
HGSNAT	34.68	32.92	31.86	31.23	0.24	0.001838	0.009467
MDM2	29.88	31.52	48.81	48.93	1.11	0.000000	0.000000
GNB2	111.09	122.54	67.42	76.69	-0.28	0.001889	0.009699
SLC16A3	233.01	250.15	239.15	263.00	0.40	0.000000	0.000000
PLK3	19.39	20.62	27.28	33.31	0.97	0.000000	0.000000
ZEB2	12.90	12.12	7.11	7.45	-0.45	0.000055	0.000381
RIC1	14.89	14.44	15.18	13.33	0.29	0.001896	0.009728
ARF6	14.84	14.40	8.35	7.21	-0.58	0.000001	0.000011
LRRC17	120.81	114.24	339.41	288.77	1.72	0.000000	0.000000
RAC1	107.00	106.11	63.33	58.27	-0.51	0.000000	0.000000
BDH2	11.72	12.11	18.54	15.77	0.80	0.000000	0.000000
COL4A1	845.82	834.53	1,460.87	1,404.70	1.14	0.000000	0.000000
PA2G4	94.73	100.17	41.09	36.09	-1.05	0.000000	0.000000
YWHAB	52.86	52.86	34.19	29.87	-0.44	0.000000	0.000001
STIP1	98.87	105.21	47.80	46.19	-0.79	0.000000	0.000000
HR	2.96	2.56	0.48	0.29	-2.84	0.000000	0.000000
RPL15	615.65	631.38	344.42	321.67	-0.69	0.000000	0.000000
C1QTNF6	5.61	5.96	6.17	6.48	0.46	0.007818	0.034003
RPLP2	420.82	457.29	300.32	273.35	-0.24	0.007884	0.034269
NFATC4	64.53	59.37	54.89	60.50	0.32	0.001714	0.008869
GALNT10	50.14	46.15	49.40	50.59	0.30	0.000034	0.000243
HLA-B	181.73	197.53	331.78	327.40	1.28	0.000000	0.000000
EVA1A	18.42	20.86	7.46	9.79	-0.89	0.000015	0.000114
TM4SF1	48.57	45.64	11.98	11.74	-1.66	0.000000	0.000000
RPS6	471.01	473.99	280.58	251.76	-0.52	0.000000	0.000000
EPG5	15.96	17.10	7.87	8.43	-0.62	0.000000	0.000000
MYC	28.69	29.16	13.21	11.66	-0.85	0.000000	0.000000
EMID1	1.11	1.28	2.10	3.35	1.38	0.000292	0.001787
AC243964.4	4.65	7.29	7.12	8.78	0.75	0.001104	0.005985
RPS24	662.21	656.67	419.04	356.21	-0.39	0.000001	0.000006
LAMB2	431.45	444.98	399.14	450.96	0.30	0.000247	0.001535

ACTN1	415.06	438.07	285.35	306.96	-0.25	0.000116	0.000766
PCSK5	0.52	0.66	1.55	1.05	1.54	0.000001	0.000007
TNFRSF10D	82.85	79.40	139.15	128.67	1.06	0.000000	0.000000
NASP	92.18	93.18	30.86	28.98	-1.36	0.000000	0.000000
RPL5	326.61	331.57	203.89	182.76	-0.47	0.000000	0.000000
RPL13A	681.89	692.59	406.23	387.64	-0.48	0.000000	0.000000
PITPNB	26.25	25.84	19.17	14.44	-0.35	0.001999	0.010185
CCNL2	174.73	163.31	213.13	212.50	0.68	0.000000	0.000000
LMCD1	7.87	11.47	16.69	15.38	1.23	0.000000	0.000000
GDI2	81.42	79.05	52.33	42.04	-0.40	0.000015	0.000113
UBE2S	57.42	67.51	14.48	17.67	-1.65	0.000000	0.000000
TNFRSF21	4.73	5.02	6.45	6.27	0.72	0.000004	0.000036
YWHAQ	104.99	104.70	54.36	41.46	-0.81	0.000000	0.000000
NACA	279.04	276.95	150.25	134.28	-0.52	0.000000	0.000000
POLD2	50.81	56.48	19.94	23.40	-1.08	0.000000	0.000000
CCT3	143.34	145.14	76.43	71.09	-0.66	0.000000	0.000000
RPL3	772.04	835.31	381.23	388.16	-0.73	0.000000	0.000000
EIF3B	103.60	96.83	54.33	51.48	-0.79	0.000000	0.000000
AP2M1	261.50	280.89	180.26	183.49	-0.29	0.000037	0.000264
IPO7	31.25	29.25	15.49	13.83	-0.75	0.000000	0.000000
CCT2	106.89	108.57	52.69	48.29	-0.85	0.000000	0.000000
DYNLL1	177.74	172.41	102.13	88.00	-0.52	0.000001	0.000006
GM2A	4.90	4.58	0.46	0.56	-2.85	0.000000	0.000000
MT-RNR2	2,889.35	2,904.27	3,616.96	3,205.96	0.58	0.000000	0.000000
IPO5	64.87	58.35	30.43	26.18	-0.87	0.000000	0.000000
RPL30	480.74	494.30	301.23	260.36	-0.43	0.000002	0.000021
GOLGA4	21.83	21.98	12.89	11.72	-0.51	0.000000	0.000000
AHNAK2	5.12	4.99	2.57	2.53	-0.50	0.000409	0.002435
UBA52	278.02	308.11	170.20	169.16	-0.41	0.000005	0.000042
KIF20A	32.41	34.48	0.98	0.70	-4.88	0.000000	0.000000
SEZ6L2	9.58	11.08	15.82	19.05	1.08	0.000000	0.000000
TMBIM6	239.96	231.78	316.12	289.80	0.56	0.000000	0.000000
CCN3	5.87	5.78	0.42	0.62	-3.15	0.000000	0.000000
EBNA1BP2	54.49	57.89	22.80	21.60	-1.02	0.000000	0.000000
SULF2	0.97	0.84	7.49	7.46	3.49	0.000000	0.000000
PLOD2	117.76	110.80	225.78	195.23	1.20	0.000000	0.000000
HEXA	85.35	87.96	84.22	88.91	0.29	0.000243	0.001508
MATN3	4.20	4.14	5.11	4.75	0.65	0.002107	0.010678
ELFN2	2.57	2.73	4.42	5.63	1.19	0.000000	0.000000
FSTL1	886.35	820.23	889.69	842.39	0.31	0.000000	0.000002
LGALS9	5.78	6.37	11.47	13.23	1.32	0.000000	0.000000
GNB1	118.80	123.01	68.55	60.81	-0.52	0.000000	0.000000
MAZ	96.26	106.29	52.56	54.80	-0.55	0.000000	0.000000

S1PR1	5.18	5.54	7.50	6.87	0.78	0.000012	0.000093
NEMP1	7.58	6.60	2.46	2.41	-1.24	0.000000	0.000000
PHLDA1	22.47	22.99	14.74	13.10	-0.35	0.000048	0.000333
DPY19L3	15.73	14.05	14.78	16.57	0.33	0.000637	0.003638
RPS23	501.09	485.51	267.06	243.00	-0.57	0.000000	0.000000
PRKCA	19.40	19.00	11.41	11.11	-0.43	0.000000	0.000000
AC126755.1	60.02	58.90	53.53	75.45	0.57	0.000001	0.000013
BOP1	29.00	34.29	11.14	14.18	-1.03	0.000000	0.000000
PHLDA3	25.60	32.72	30.68	33.27	0.51	0.000019	0.000140
NADSYN1	39.94	38.19	45.67	51.64	0.65	0.000000	0.000000
PNISR	62.79	59.32	64.05	62.34	0.39	0.000000	0.000001
OSBPL3	17.73	15.81	18.06	21.83	0.31	0.002149	0.010876
COMP	0.46	0.65	13.66	15.61	5.07	0.000000	0.000000
SV2A	19.85	20.37	27.71	27.44	0.80	0.000000	0.000000
ATP13A3	46.54	44.88	36.76	29.46	-0.26	0.000721	0.004072
CLTB	39.92	44.22	20.93	24.33	-0.52	0.000360	0.002167
LIMS1	17.90	20.04	21.78	22.18	0.65	0.000000	0.000000
TNFSF15	0.04	0.02	1.40	1.22	5.60	0.000000	0.000000
KYNU	1.44	1.52	0.39	0.39	-1.86	0.002726	0.013443
RTN3	49.99	45.78	69.24	57.89	0.72	0.000000	0.000000
TNS1	35.00	36.66	49.01	54.00	0.82	0.000000	0.000000
EIF2AK4	21.82	21.66	12.35	11.76	-0.78	0.000000	0.000000
GALNT15	3.43	5.14	0.21	0.25	-4.51	0.000000	0.000000
PLD3	324.95	359.61	428.02	475.78	0.76	0.000000	0.000000
FAF2	27.57	26.51	17.93	15.91	-0.41	0.000004	0.000034
L1CAM	0.23	0.77	1.36	1.39	2.62	0.000000	0.000000
HLA-E	66.14	71.90	71.51	69.20	0.35	0.000004	0.000035
ARHGAP22	20.67	23.07	8.41	8.04	-1.09	0.000000	0.000000
F3	205.21	187.31	262.70	256.80	0.65	0.000000	0.000000
DPF3	14.44	15.18	3.47	2.84	-1.91	0.000000	0.000000
SHOC2	7.20	7.59	10.83	12.00	0.80	0.000000	0.000000
TMED10	177.30	171.68	218.67	203.64	0.32	0.000032	0.000230
PXDN	309.90	311.54	467.31	490.38	0.94	0.000000	0.000000
RSU1	49.88	52.16	55.48	52.80	0.47	0.000000	0.000002
CCT8	93.55	91.84	55.71	48.51	-0.50	0.000000	0.000000
EGR2	0.30	0.24	5.86	6.62	5.10	0.000000	0.000000
PRDX4	132.54	128.73	187.67	167.54	0.79	0.000000	0.000000
GYS1	33.05	37.09	38.01	40.41	0.50	0.000000	0.000000
NME2	199.22	230.04	127.01	126.93	-0.41	0.000012	0.000095
SLC18B1	3.09	2.02	4.79	4.19	1.15	0.000009	0.000072
PSAT1	68.09	72.02	114.95	96.27	0.93	0.000000	0.000000
PKDCC	117.36	125.67	149.66	146.42	0.71	0.000000	0.000000
PTPN14	15.10	13.48	7.41	7.16	-0.65	0.000000	0.000000

TMEM47	19.02	17.63	12.13	9.97	-0.40	0.000418	0.002485
EIF4EBP1	61.41	68.46	69.85	65.42	0.41	0.000537	0.003120
CD302	2.72	2.63	1.11	1.15	-0.90	0.000456	0.002693
RPL11	633.46	650.40	365.74	332.97	-0.52	0.000000	0.000000
LDHB	324.06	314.76	170.26	136.56	-0.74	0.000000	0.000000
ID3	23.88	26.59	5.88	6.13	-1.71	0.000000	0.000000
RASSF2	0.60	0.67	3.58	3.20	2.75	0.000000	0.000000
CDCA3	30.55	27.48	1.01	1.10	-4.05	0.000000	0.000000
PPDPF	66.68	72.27	34.44	38.29	-0.57	0.000031	0.000223
JUN	33.90	35.04	16.77	15.54	-0.76	0.000000	0.000000
SLC39A1	49.65	51.22	24.85	27.96	-0.59	0.000000	0.000000
TMSB4X	913.13	969.55	609.07	547.37	-0.29	0.000019	0.000143
PSMD2	157.90	165.49	76.09	84.06	-0.83	0.000000	0.000000
F2RL2	29.39	25.34	31.15	28.12	0.41	0.000008	0.000060
DYNC1H1	290.47	279.66	189.97	192.67	-0.22	0.000181	0.001152
H3-3B	188.06	203.17	130.64	131.02	-0.25	0.002172	0.010979
KPNB1	219.34	220.05	119.81	116.53	-0.64	0.000000	0.000000
PTGES3	71.77	70.78	40.75	35.28	-0.57	0.000000	0.000000
TSPAN12	0.57	0.79	2.70	3.17	2.94	0.000000	0.000000
RPLP1	221.64	229.03	139.34	126.95	-0.39	0.000000	0.000001
PPRC1	40.99	41.31	22.37	24.57	-0.53	0.000000	0.000000
NANS	49.38	48.68	27.44	27.36	-0.50	0.000036	0.000259
RPL6	579.29	570.84	322.57	295.20	-0.54	0.000000	0.000000
PSG5	8.77	9.30	1.22	1.21	-2.47	0.000000	0.000000
SLC9A3-AS1	40.71	40.28	20.86	23.52	-0.49	0.000451	0.002668
LPCAT1	37.05	34.04	17.50	15.27	-0.67	0.000000	0.000000
TNS3	45.74	45.13	16.01	15.99	-1.21	0.000000	0.000000
CDK2AP1	77.95	81.62	54.97	45.65	-0.33	0.000680	0.003859
BTF3	184.79	190.28	106.32	94.23	-0.56	0.000000	0.000000
CTNNAL1	28.31	26.49	7.06	5.96	-1.74	0.000000	0.000000
LRP1	207.69	215.43	258.78	302.87	0.78	0.000000	0.000000
SLC2A6	6.04	6.13	9.34	10.65	1.03	0.000000	0.000000
RAB1B	70.69	77.98	41.05	47.56	-0.40	0.000152	0.000983
TMEM123	81.63	76.05	59.02	47.48	-0.31	0.001754	0.009069
NOTCH1	11.23	11.75	13.59	15.38	0.67	0.000000	0.000000
ASAP1	45.17	50.39	24.51	30.67	-0.50	0.000000	0.000000
FKBP4	21.80	22.28	11.67	10.69	-0.74	0.000000	0.000000
CYCS	31.31	29.45	15.47	14.55	-0.60	0.000004	0.000032
MTHFD2	62.13	59.60	73.21	65.23	0.41	0.000001	0.000012
GPX1	121.02	125.74	40.50	41.65	-1.23	0.000000	0.000000
KCNG1	12.20	15.26	29.51	31.08	1.48	0.000000	0.000000
TIMP2	280.83	269.65	303.60	290.53	0.44	0.000000	0.000000
PDPR	10.70	10.40	6.39	6.50	-0.41	0.000019	0.000142

TUBB2B	6.45	7.63	10.02	9.17	0.79	0.000020	0.000147
IDH1	29.67	29.71	46.70	42.04	0.89	0.000000	0.000000
UBR4	135.76	134.43	114.28	122.93	0.18	0.004998	0.022941
PDGFD	7.74	7.03	15.28	13.34	1.28	0.000000	0.000000
IL13RA1	23.53	23.00	28.09	24.29	0.51	0.000000	0.000001
SEMA3D	9.38	8.60	8.79	8.23	0.44	0.000529	0.003081
FMOD	0.72	0.68	3.02	3.35	2.52	0.000000	0.000000
RPL18A	464.68	493.62	276.95	272.81	-0.45	0.000000	0.000000
SGIP1	51.74	42.76	47.05	48.52	0.57	0.000000	0.000000
MET	17.94	15.49	8.73	8.17	-0.63	0.000000	0.000000
CSDE1	116.43	116.29	77.35	72.03	-0.39	0.000000	0.000000
MTCH1	97.31	101.36	64.05	61.69	-0.33	0.000020	0.000149
FAIM2	4.27	3.61	7.98	8.12	1.51	0.000000	0.000000
RAB13	153.71	161.24	91.74	83.69	-0.50	0.000000	0.000000
TCP1	94.02	94.65	62.00	54.35	-0.44	0.000001	0.000007
IQGAP3	12.50	14.06	0.60	0.77	-3.72	0.000000	0.000000
PYCR1	67.08	78.47	66.92	70.40	0.28	0.002903	0.014220
CLSTN3	20.68	23.24	26.36	28.97	0.58	0.000000	0.000000
C12orf75	37.92	39.01	16.28	15.50	-1.04	0.000000	0.000000
PHLDA2	21.86	24.08	11.79	12.89	-0.55	0.005859	0.026393
NYNRIN	2.39	2.54	3.82	3.77	0.96	0.000000	0.000000
JPT2	23.33	23.22	11.16	11.18	-0.67	0.000000	0.000000
OAZ1	334.08	363.17	218.58	215.93	-0.31	0.000059	0.000406
EEF1D	397.10	414.39	214.62	255.16	-0.46	0.000000	0.000000
IRAK1	40.67	43.53	18.62	20.02	-0.90	0.000000	0.000000
FMN1	1.93	1.67	0.62	1.12	-0.78	0.000346	0.002086
ERRFI1	32.17	29.47	50.26	43.42	0.95	0.000000	0.000000
GPX4	178.11	200.49	72.59	72.83	-1.01	0.000000	0.000000
MACF1	118.03	110.81	112.60	111.50	0.35	0.000000	0.000000
RRAS2	26.02	25.84	15.69	16.67	-0.53	0.000122	0.000804
NUCB2	92.68	81.26	112.80	98.19	0.58	0.000000	0.000000
IL1B	3.95	4.32	0.34	0.38	-3.03	0.000000	0.000000
ASPM	14.77	11.66	0.43	0.45	-4.60	0.000000	0.000000
CLCN6	13.09	13.55	17.86	17.01	0.69	0.000000	0.000000
PIGT	104.06	100.10	93.07	92.99	0.24	0.000908	0.005011
RPL32	394.07	404.14	226.81	225.86	-0.51	0.000000	0.000000
CPZ	5.60	5.75	11.69	14.07	1.59	0.000000	0.000000
DNAH5	1.02	1.21	0.71	0.79	0.59	0.005481	0.024914
GJA1	178.35	170.50	221.32	199.80	0.61	0.000000	0.000000
CAV1	350.47	330.97	160.49	148.18	-0.83	0.000000	0.000000
SCRN1	27.60	27.55	14.56	13.34	-0.68	0.000000	0.000000
FOSL1	78.81	80.68	12.98	13.19	-2.29	0.000000	0.000000
RPL17	517.47	512.02	287.86	253.23	-0.58	0.000000	0.000000

THY1	492.64	515.03	486.58	513.82	0.24	0.000104	0.000696
PRDX1	289.62	282.74	142.04	115.54	-0.82	0.000000	0.000000
TTC17	51.09	49.75	57.09	57.38	0.53	0.000000	0.000000
SYNE1	26.81	24.76	27.14	28.61	0.39	0.000000	0.000002
SLC26A6	50.05	52.33	49.18	51.56	0.28	0.002658	0.013157
PGK1	159.80	163.12	191.72	188.85	0.57	0.000000	0.000000
ATP5F1B	297.15	305.07	162.74	154.05	-0.61	0.000000	0.000000
SIPA1L2	5.36	5.92	14.77	15.93	1.67	0.000000	0.000000
AUXG0100005	3.02	2.99	8.98	9.05	1.92	0.000000	0.000000
WSB2	27.90	28.24	16.24	17.42	-0.38	0.000929	0.005120
SPON2	430.68	443.16	485.90	501.76	0.55	0.000000	0.000000
LPAR1	95.32	89.67	59.35	54.41	-0.33	0.000005	0.000038
EFCC1	0.08	0.06	0.73	0.93	4.03	0.000007	0.000058
PPP2R1A	87.61	88.97	59.70	64.87	-0.26	0.001188	0.006393
HHIP	3.05	2.64	12.03	11.36	2.35	0.000000	0.000000
CUL9	20.81	21.50	11.06	13.31	-0.74	0.000000	0.000003
ADGRL2	15.24	13.57	22.58	19.78	1.02	0.000000	0.000000
FTH1	1,421.53	1,530.83	569.80	508.26	-1.10	0.000000	0.000000
ZC3H7B	38.58	41.63	22.27	24.94	-0.43	0.000001	0.000006
CLK1	27.63	29.16	32.07	30.75	0.51	0.000001	0.000006
BOC	2.18	2.75	4.82	4.18	1.42	0.000000	0.000000
FAXDC2	1.49	1.70	3.65	3.53	1.08	0.000144	0.000934
AHSA2P	32.91	30.37	36.59	34.43	0.57	0.000000	0.000000
VDAC1	97.81	105.05	60.38	57.83	-0.49	0.000000	0.000000
LIF	96.89	100.62	35.78	42.35	-1.07	0.000000	0.000000
PTPRM	18.07	18.17	34.60	30.49	1.15	0.000000	0.000000
CCT5	188.52	198.46	89.14	83.66	-0.87	0.000000	0.000000
GRAMD2B	5.76	5.63	9.80	8.17	1.03	0.000000	0.000000
ATP10D	16.57	16.81	22.08	19.75	0.60	0.000000	0.000000
PTP4A2	80.49	80.09	60.96	60.86	-0.36	0.000101	0.000673
SYNM	2.67	3.01	7.17	6.73	1.54	0.000000	0.000000
BDKRB1	122.33	125.44	37.87	39.65	-1.33	0.000000	0.000000
FOXL1	13.04	14.25	12.82	14.29	0.31	0.009036	0.038562
SET	153.17	139.92	83.79	68.10	-0.63	0.000000	0.000000
ATP8B4	0.12	0.36	0.99	0.61	1.96	0.001222	0.006559
FJX1	17.52	16.14	11.02	10.58	-0.41	0.004707	0.021779
RAN	369.31	369.92	172.02	138.76	-0.93	0.000000	0.000000
GADD45A	28.34	27.09	30.61	29.09	0.47	0.000176	0.001120
ASAH1	27.56	26.54	24.64	23.49	0.31	0.005058	0.023167
CAPNS1	405.82	422.65	280.73	275.95	-0.23	0.001609	0.008394
CACNA1H	10.44	10.50	25.00	28.04	1.80	0.000000	0.000000
IL21R	0.86	1.09	7.89	8.32	3.51	0.000000	0.000000
SHROOM3	2.70	2.61	3.61	4.21	0.70	0.000001	0.000005

MRFAP1	133.50	154.41	88.68	81.17	-0.40	0.000001	0.000005
BRD8	43.60	38.95	29.11	25.10	-0.35	0.000598	0.003446
PDLIM7	277.85	295.58	312.52	372.22	0.51	0.000000	0.000000
FAT3	3.28	1.93	4.12	2.57	0.75	0.000002	0.000017
CEACAM19	18.72	16.95	28.52	31.55	1.09	0.000000	0.000000
MTPN	25.48	24.36	15.21	13.99	-0.44	0.000004	0.000036
RPS15	415.09	413.32	209.85	219.00	-0.57	0.000000	0.000000
EZR	41.69	42.77	21.60	21.99	-0.62	0.000000	0.000000
SRSF5	122.90	121.62	120.61	117.24	0.33	0.000022	0.000162
LGALS3BP	170.26	185.43	159.61	179.13	0.29	0.000286	0.001759
TIMP1	1,549.65	1,533.43	2,172.76	2,199.53	0.85	0.000000	0.000000
NDST1	53.42	57.71	51.50	56.56	0.28	0.000050	0.000345
DDOST	286.22	286.85	264.88	262.68	0.21	0.000315	0.001920
CLTA	82.26	81.84	49.08	42.18	-0.51	0.000003	0.000022
UACA	71.48	68.88	49.49	48.94	-0.23	0.001026	0.005597
ABCC1	82.70	85.27	49.44	57.10	-0.31	0.000017	0.000125
GNG12	25.20	25.51	14.57	11.94	-0.60	0.000000	0.000000
GASK1B	7.50	5.82	9.17	9.46	0.96	0.000000	0.000000
ALDOC	10.77	11.93	18.03	19.93	1.12	0.000000	0.000000
YWHAE	127.37	126.60	62.83	50.17	-0.85	0.000000	0.000000
SH2D5	9.30	10.30	1.69	1.30	-2.16	0.000000	0.000000
DPYSL3	43.86	42.46	51.49	48.28	0.45	0.000000	0.000000
PDGFRB	135.18	136.46	239.93	256.87	1.17	0.000000	0.000000
NACC1	38.72	46.58	27.62	30.95	-0.39	0.000209	0.001314
ATP6AP2	113.60	106.31	133.61	119.61	0.46	0.000000	0.000000
PAWR	13.65	13.56	21.60	22.16	0.65	0.000000	0.000002
HSP90AB1	548.23	540.11	235.81	216.77	-0.93	0.000000	0.000000
TPCN1	7.74	9.98	8.18	10.60	0.43	0.000543	0.003153
ABHD5	7.44	7.25	4.10	3.26	-0.60	0.003780	0.017937
LACTB	26.46	25.74	15.43	13.73	-0.51	0.000224	0.001397
PTK7	147.48	147.74	310.26	331.14	1.51	0.000000	0.000000
S100A16	58.38	62.67	31.33	35.27	-0.46	0.000118	0.000780
RBPMS	82.01	83.26	82.67	85.64	0.40	0.000001	0.000012
HS6ST1	38.24	41.26	17.26	18.71	-0.81	0.000000	0.000000
FMN2	5.23	5.74	2.19	2.45	-0.61	0.001921	0.009845
ADGRL3	0.27	0.25	1.43	1.64	2.95	0.000000	0.000000
EIF4H	120.57	119.16	86.09	79.27	-0.24	0.000528	0.003078
TMEM59	99.72	96.27	146.42	128.30	0.81	0.000000	0.000000
SCAP	29.82	32.86	14.62	19.13	-0.61	0.000000	0.000000
MGST1	203.44	180.47	109.51	99.39	-0.59	0.000000	0.000000
PKM	1,058.84	1,102.48	674.61	702.70	-0.34	0.000000	0.000000
RPL27A	501.89	528.47	326.51	286.29	-0.43	0.000000	0.000000
ZNF106	17.24	12.99	9.94	9.42	-0.36	0.000066	0.000451

SHROOM2	7.15	8.06	2.99	3.12	-0.88	0.000000	0.000001
EBP	26.54	25.19	37.74	35.63	0.73	0.000000	0.000001
SLC25A3	215.48	220.82	151.82	135.21	-0.21	0.003583	0.017140
RHOA	181.94	182.34	112.99	102.73	-0.38	0.000000	0.000003
BNIP3	22.76	22.52	41.98	35.55	1.18	0.000000	0.000000
CFH	11.67	12.28	33.17	30.16	1.95	0.000000	0.000000
PDE1C	9.83	10.82	5.14	4.11	-0.76	0.000000	0.000001
ENC1	10.34	10.10	11.54	12.13	0.72	0.000000	0.000000
FRZB	2.54	2.55	23.93	24.56	3.59	0.000000	0.000000
NXN	69.15	69.15	40.32	47.31	-0.39	0.000002	0.000015
PTMA	414.25	418.57	116.38	107.86	-1.52	0.000000	0.000000
PLTP	46.36	48.39	103.97	110.21	1.54	0.000000	0.000000
HSPA5	638.88	620.46	674.32	628.92	0.39	0.000000	0.000000
LAMC1	249.56	235.70	304.21	288.49	0.62	0.000000	0.000000
RPS4X	418.48	421.53	221.63	206.63	-0.63	0.000000	0.000000
GRN	172.41	186.81	160.13	190.28	0.29	0.001460	0.007688
SC5D	25.07	24.37	43.69	40.24	1.05	0.000000	0.000000
KCTD10	33.11	29.05	33.59	31.99	0.45	0.000000	0.000001
TMEM9	48.27	51.25	22.90	24.32	-0.83	0.000000	0.000000
SERPINB7	8.56	6.69	3.05	2.05	-1.40	0.000000	0.000000
TNFSF4	5.70	4.64	19.13	17.26	2.21	0.000000	0.000000
TUBA1C	524.70	530.84	122.71	134.59	-1.65	0.000000	0.000000
PCMTD1	6.38	5.41	9.21	8.23	0.93	0.000000	0.000000
FBLN5	179.52	163.50	257.10	239.41	0.85	0.000000	0.000000
SVIL	9.76	14.86	12.40	12.06	0.64	0.000000	0.000000
RPL7A	909.64	978.17	428.63	435.89	-0.78	0.000000	0.000000
HSPD1	183.87	183.09	95.37	84.33	-0.70	0.000000	0.000000
LYPD1	16.23	16.80	7.83	7.18	-0.71	0.000000	0.000002
NCL	375.87	401.16	150.79	156.31	-1.00	0.000000	0.000000
ERCC2	37.57	38.08	20.56	22.94	-0.56	0.000000	0.000000
AGRN	163.93	170.80	167.98	190.47	0.44	0.000000	0.000000
NBL1	21.22	25.16	72.35	86.12	2.02	0.000000	0.000000
RPS2P5	164.83	166.18	100.32	82.68	-0.51	0.000000	0.000003
NME1	106.55	109.16	59.84	59.21	-0.51	0.000000	0.000002
ADAMTS9	0.46	0.72	1.55	2.65	2.64	0.000000	0.000000
PAM	98.34	91.25	96.04	90.75	0.32	0.000010	0.000079
LRRC59	75.18	77.43	43.33	44.37	-0.46	0.000000	0.000000
TUBB	896.68	942.73	321.28	323.98	-1.17	0.000000	0.000000
LINC02593	1.99	2.13	4.04	4.51	1.43	0.000000	0.000000
LARP6	37.05	40.03	36.72	33.78	0.40	0.000678	0.003849
SEMA4D	10.09	0.78	3.20	3.11	2.06	0.000000	0.000000
EEF1G	736.01	807.06	454.82	453.62	-0.42	0.000000	0.000000
FABP3	1.48	1.61	13.47	13.47	3.55	0.000000	0.000000

ARF1	195.23	218.56	106.49	111.15	-0.59	0.000000	0.000000
CSRP2	34.94	36.39	63.25	50.01	1.10	0.000000	0.000000
COL16A1	248.63	258.08	251.06	272.63	0.48	0.000000	0.000000
CDH2	59.48	56.68	134.10	121.98	1.48	0.000000	0.000000
RARRES2	5.98	3.37	43.88	51.58	3.75	0.000000	0.000000
STC1	9.95	8.73	16.06	15.03	1.04	0.000000	0.000000
SHISA3	0.76	0.32	1.73	1.49	1.92	0.000063	0.000435
TRABD2A	13.12	12.38	6.95	5.89	-0.67	0.000048	0.000338
PTPRN	33.91	36.88	51.89	58.94	1.03	0.000000	0.000000
EEF2	813.85	870.07	439.69	468.81	-0.55	0.000000	0.000000
RUNX1	11.62	10.59	15.04	15.25	0.62	0.000000	0.000000
ILK	151.74	153.69	174.39	182.80	0.48	0.000000	0.000000
EPN1	52.28	58.13	29.65	32.48	-0.41	0.000066	0.000453
SLC14A1	26.73	19.06	8.45	7.42	-1.04	0.000000	0.000000
WWC2	16.95	18.62	11.45	10.91	-0.40	0.000062	0.000424
ELOVL6	14.24	13.73	20.32	18.97	0.87	0.000000	0.000000
HDLBP	368.16	361.59	264.83	271.10	-0.32	0.000000	0.000000
COL8A1	82.05	84.72	111.29	109.80	0.68	0.000000	0.000000
VSTM4	1.87	1.80	3.51	3.02	1.19	0.000000	0.000000
PANK3	5.12	4.55	6.27	5.27	0.59	0.000000	0.000002
MARCHF4	4.98	5.57	2.60	2.61	-0.68	0.000042	0.000297
ARHGAP28	1.00	1.72	3.00	2.73	1.91	0.000000	0.000000
SRM	103.50	108.31	26.77	31.84	-1.58	0.000000	0.000000
HEPH	32.51	32.98	54.45	51.10	0.99	0.000000	0.000000
EIF6	118.35	127.28	60.76	64.31	-0.61	0.000000	0.000000
TTC28	6.21	6.87	7.68	7.84	0.50	0.000001	0.000012
MAT2A	197.02	187.29	135.71	127.60	-0.22	0.000633	0.003619
RACK1	722.66	768.68	411.19	407.54	-0.51	0.000000	0.000000
YKT6	47.29	50.44	36.55	33.61	-0.22	0.012125	0.049887
PLS3	58.65	57.34	55.99	52.05	0.21	0.005724	0.025875
NF2	17.75	18.65	7.18	6.40	-1.19	0.000000	0.000000
CHAC1	30.09	32.54	50.72	55.29	1.10	0.000000	0.000000
STEAP3	26.87	28.56	27.38	30.43	0.36	0.000123	0.000812
SYNC	25.90	26.67	12.60	12.01	-0.66	0.000000	0.000000
AKR1C1	10.25	11.25	4.04	2.76	-1.16	0.000035	0.000247
HEG1	82.07	73.77	43.07	43.67	-0.62	0.000000	0.000000
DAPK3	68.80	70.24	67.61	74.03	0.30	0.003617	0.017273
TPP1	57.60	57.06	64.81	62.60	0.50	0.000000	0.000000
DSTN	105.63	107.49	119.73	98.87	0.34	0.000101	0.000675
HSPB6	68.66	69.47	23.11	22.69	-1.25	0.000000	0.000000
HIF1A	137.03	123.62	64.17	53.85	-0.85	0.000000	0.000000
SZRD1	42.02	44.49	28.38	29.36	-0.26	0.004146	0.019511
ACSL3	43.69	40.02	88.12	74.19	1.08	0.000000	0.000000

S100A11	348.93	355.34	381.44	360.80	0.44	0.000000	0.000000
PKD1	127.55	136.46	154.88	189.87	0.91	0.000000	0.000000
RPL19	716.11	784.97	432.77	430.60	-0.45	0.000000	0.000000
CCDC14	65.69	58.95	40.38	32.31	-0.34	0.000011	0.000086
GPR176	22.56	21.30	14.01	12.93	-0.35	0.000341	0.002061
MPZL1	67.07	47.52	79.25	69.97	0.39	0.000000	0.000002
ASPH	313.74	285.96	211.59	181.91	-0.29	0.000008	0.000065
MKI67	29.95	26.72	0.95	0.71	-5.07	0.000000	0.000000
SERBP1	109.12	103.24	49.37	47.33	-0.84	0.000000	0.000000
SNED1	4.15	4.54	4.26	5.90	0.93	0.000000	0.000000
TUBB3	119.08	132.09	47.51	54.89	-0.99	0.000000	0.000000
RIPOR3	0.65	1.51	3.99	5.12	2.76	0.000000	0.000000
TBC1D2	10.84	12.21	6.69	6.94	-0.46	0.001864	0.009587
TNFRSF1B	5.54	5.80	1.66	1.58	-1.47	0.000000	0.000000
CALM3	149.77	150.46	68.03	63.46	-0.90	0.000000	0.000000
PCDH7	15.63	14.98	28.63	28.29	1.22	0.000000	0.000000
PRELID1	71.13	76.49	43.68	40.93	-0.48	0.000004	0.000032
CYBA	101.15	114.01	31.34	38.64	-1.26	0.000000	0.000000
CRTAP	148.05	147.35	99.66	79.58	-0.32	0.000011	0.000086
MCC	3.80	4.37	6.83	7.14	0.93	0.000000	0.000000
FRMD4A	69.79	72.84	52.70	38.63	-0.30	0.000305	0.001859
RPL13	667.77	709.87	370.35	374.08	-0.57	0.000000	0.000000
SOD2	76.55	76.03	22.95	22.59	-1.28	0.000000	0.000000
SARS1	116.32	119.97	149.16	144.43	0.63	0.000000	0.000000
SORL1	0.89	1.94	0.61	0.79	-0.74	0.004845	0.022304
PTPRF	83.14	82.94	72.86	84.22	0.27	0.000366	0.002198
PLA2G3	0.43	0.37	2.04	1.95	2.65	0.000000	0.000000
EIF3F	103.76	111.11	60.38	65.16	-0.34	0.000019	0.000142
NSG1	3.10	2.23	10.97	10.13	2.28	0.000000	0.000000
MYO1C	84.46	90.06	55.64	55.26	-0.35	0.000000	0.000004
PIEZO2	1.09	0.68	6.17	4.71	3.37	0.000000	0.000000
HAS2	13.55	12.40	5.54	4.58	-1.02	0.000000	0.000000
GALNT6	11.87	14.11	4.00	4.35	-1.40	0.000000	0.000000
CDKN2B	3.29	3.28	13.45	11.90	2.18	0.000000	0.000000
ALDH2	19.84	22.61	22.51	23.13	0.46	0.000062	0.000429
LMOD1	16.62	17.34	23.09	24.79	0.83	0.000000	0.000000
ADD3	22.82	22.78	33.40	27.95	0.53	0.000000	0.000004
LIPG	0.71	0.64	14.31	13.10	4.76	0.000000	0.000000
ARHGAP23	34.17	37.66	16.49	18.28	-0.76	0.000000	0.000000
ANXA1	125.32	125.89	85.05	76.23	-0.33	0.000069	0.000471
MANBA	12.37	11.38	15.46	14.03	0.69	0.000000	0.000000
BASP1	68.68	66.15	33.30	28.86	-0.78	0.000000	0.000000
CDC25B	46.98	42.82	10.12	12.36	-1.58	0.000000	0.000000

CALCOCO1	24.05	23.81	31.50	34.60	0.86	0.000000	0.000000
PDE4B	3.70	3.69	1.26	1.22	-1.71	0.000000	0.000000
COL6A3	1,662.19	1,642.15	2,105.01	2,165.53	0.71	0.000000	0.000000
GARS1	215.41	223.23	269.97	269.98	0.61	0.000000	0.000000
EIF4A1	637.83	628.67	295.01	269.51	-0.88	0.000000	0.000000
NSDHL	16.41	15.39	17.07	17.55	0.48	0.000525	0.003065
EDN1	2.43	1.66	5.53	5.13	1.72	0.000000	0.000000
PRUNE2	0.81	0.72	2.79	3.25	2.28	0.000000	0.000000
WNK4	13.16	13.94	3.81	4.92	-1.19	0.000000	0.000000
LOXL4	17.29	16.88	38.49	39.95	1.54	0.000000	0.000000
SEMA3B	20.91	19.43	9.97	10.70	-0.66	0.000001	0.000006
C16orf72	9.33	8.84	10.81	8.71	0.44	0.000022	0.000161
IMPAD1	17.19	16.40	10.34	8.64	-0.48	0.000001	0.000006
EIF4G2	254.39	251.24	192.43	159.40	-0.27	0.000025	0.000182
NRG1	4.10	4.44	11.63	10.66	1.83	0.000000	0.000000
RPL7	610.56	593.83	354.74	297.67	-0.50	0.000000	0.000000
COL12A1	579.62	544.90	937.72	910.68	1.17	0.000000	0.000000
GNAS	363.10	411.79	330.03	354.95	0.17	0.009227	0.039276
TMEM97	20.98	18.61	12.31	9.51	-0.73	0.000005	0.000038
ANKRD10	29.03	25.19	46.45	40.15	1.14	0.000000	0.000000
KREMEN1	2.08	1.58	2.06	2.38	0.65	0.002325	0.011656
AC055839.2	39.32	37.97	9.45	9.71	-1.67	0.000000	0.000000
FST	238.57	231.04	77.94	76.66	-1.25	0.000000	0.000000
FERMT2	74.14	71.42	118.31	104.08	0.97	0.000000	0.000000
NPM1	731.25	709.33	283.82	251.07	-1.17	0.000000	0.000000
RFLNB	10.06	11.64	3.56	3.81	-1.22	0.000000	0.000000
PSAP	618.94	639.18	927.01	918.79	0.83	0.000000	0.000000
RHOC	188.60	206.54	120.88	125.94	-0.33	0.000125	0.000819
F2R	83.48	78.89	102.84	90.47	0.59	0.000000	0.000000
C1orf198	26.40	25.66	37.34	40.93	0.78	0.000000	0.000000
GRK5	5.29	5.50	6.04	5.62	0.43	0.000299	0.001827
MAN1A1	7.54	7.34	10.19	8.67	0.68	0.000000	0.000000
MYO1E	12.14	13.95	27.96	20.94	1.04	0.000000	0.000000
ITGB8	2.68	2.51	4.02	3.62	0.89	0.000000	0.000000
LMNA	343.56	367.23	173.34	181.21	-0.64	0.000000	0.000000
PCDH18	42.29	39.35	61.02	61.23	1.05	0.000000	0.000000
POLR2A	49.74	50.20	26.48	41.75	-0.74	0.000000	0.000000
FADS1	28.78	29.95	50.69	45.94	1.10	0.000000	0.000000
MT-ND4	7,072.75	6,830.18	7,186.24	6,582.94	0.33	0.000000	0.000000
MGLL	30.81	24.37	10.86	10.75	-0.83	0.000000	0.000000
CENPF	42.80	39.15	1.66	1.54	-4.51	0.000000	0.000000
PPIA	911.47	918.11	566.16	504.97	-0.39	0.000000	0.000000
ADAMTSL1	8.70	9.39	2.32	1.86	-1.96	0.000000	0.000000

DDX21	41.03	37.82	19.02	17.36	-0.78	0.000000	0.000000
PLPP1	14.22	15.12	34.92	31.92	1.53	0.000000	0.000000
NIBAN2	111.31	127.42	59.58	67.37	-0.57	0.000000	0.000000
FKBP9	136.26	135.45	180.75	174.90	0.68	0.000000	0.000000
MICA	34.59	34.27	42.49	43.85	0.68	0.000000	0.000000
LGALS1	1,777.17	1,999.91	919.53	948.46	-0.65	0.000000	0.000000
SLC20A2	20.94	19.51	24.73	25.65	0.74	0.000000	0.000000
RPL8	767.76	835.69	417.97	454.85	-0.55	0.000000	0.000000
RPSA	577.46	601.73	332.92	328.72	-0.47	0.000000	0.000000
FLNA	1,847.68	1,951.21	1,563.79	1,825.11	0.23	0.002086	0.010580
HLA-A	98.85	98.37	159.28	160.40	1.04	0.000000	0.000000
FSTL3	57.05	64.22	68.97	73.11	0.62	0.000000	0.000000
PXN	72.89	73.11	42.30	48.02	-0.30	0.000549	0.003184
NEDD9	3.12	2.52	7.64	6.63	1.72	0.000000	0.000000
FENDRR	45.56	45.72	52.62	54.05	0.55	0.000000	0.000000
MT-ATP8	8,191.26	7,957.64	10,396.43	9,096.32	0.76	0.000000	0.000000
NREP	96.71	94.25	95.76	88.63	0.25	0.001152	0.006211
CCPG1	8.52	8.96	38.18	33.79	2.25	0.000000	0.000000
AC159540.2	7.84	8.66	8.52	8.85	0.44	0.001006	0.005504
FSCN1	116.32	122.19	69.89	73.23	-0.42	0.000000	0.000000
SCARB2	73.82	76.17	112.00	98.35	0.68	0.000000	0.000000
YWHAG	49.75	47.93	32.79	30.42	-0.29	0.000132	0.000866
TGM2	58.94	59.32	76.47	78.40	0.67	0.000000	0.000000
XYLT1	9.91	9.33	10.69	10.74	0.48	0.000000	0.000000
SLC39A6	38.91	34.10	38.75	34.94	0.42	0.000000	0.000005
GBP1	31.42	30.80	69.45	69.59	1.25	0.000000	0.000000
ITM2C	127.32	134.41	134.04	133.20	0.35	0.000000	0.000004
LHFPL2	11.78	10.93	13.48	12.29	0.48	0.000011	0.000085
RNF24	7.76	7.65	8.41	8.69	0.32	0.005049	0.023138
SLITRK6	0.88	0.73	9.20	8.50	3.75	0.000000	0.000000
CDCP1	4.41	4.03	5.52	5.67	0.70	0.000000	0.000002
ANO1	3.62	4.56	7.43	8.08	1.39	0.000000	0.000000
AMOTL2	51.49	52.06	16.65	17.14	-1.29	0.000000	0.000000
PDE7B	5.32	4.94	1.49	1.24	-1.54	0.000000	0.000000
PDLIM5	26.78	24.03	31.09	30.55	0.74	0.000000	0.000000
SERPINF1	49.41	54.61	103.06	105.44	1.35	0.000000	0.000000
DCHS1	8.04	8.72	11.80	12.76	0.89	0.000000	0.000000
RPLP0	1,000.32	1,040.96	594.03	585.42	-0.43	0.000000	0.000000
MVK	7.87	7.80	10.70	11.80	0.74	0.000051	0.000355
COL4A2	928.69	953.23	1,297.21	1,342.94	0.86	0.000000	0.000000
PTCH1	11.41	13.46	28.83	22.94	1.31	0.000000	0.000000
EFNB2	7.73	7.90	11.23	10.68	0.83	0.000000	0.000000
DAB2	64.36	61.32	31.12	28.81	-0.81	0.000000	0.000000

CHN1	35.25	32.15	39.55	33.49	0.46	0.000008	0.000065
TPM3	164.08	164.06	99.39	92.14	-0.42	0.000000	0.000000
ADAM12	23.95	22.70	61.36	57.35	1.68	0.000000	0.000000
CNN1	55.21	67.25	133.38	138.52	1.48	0.000000	0.000000
SEPTIN9	206.47	215.39	106.47	107.61	-0.73	0.000000	0.000000
UAP1	37.64	37.49	21.58	18.18	-0.62	0.000000	0.000000
CITED2	67.79	71.71	34.08	31.79	-0.77	0.000000	0.000000
BCAT1	36.76	33.07	42.07	37.48	0.35	0.000001	0.000011
ABCG1	0.40	0.28	1.82	2.01	2.97	0.000000	0.000000
SQLE	34.45	31.62	89.18	75.35	1.64	0.000000	0.000000
NUAK1	3.58	3.04	17.96	17.21	2.80	0.000000	0.000000
GK	6.23	6.20	3.15	2.57	-0.98	0.000001	0.000006
TM7SF2	8.53	5.98	11.27	12.59	1.20	0.000000	0.000000
KCNMA1	17.09	17.58	9.20	8.23	-0.55	0.000000	0.000002
CD82	49.08	45.45	76.87	76.53	1.11	0.000000	0.000000
DAP	62.02	62.73	38.18	34.97	-0.44	0.000000	0.000001
TRANK1	6.73	4.44	7.12	8.58	0.76	0.000000	0.000000
P3H2	28.16	27.88	16.88	17.45	-0.64	0.000000	0.000000
TGFBR3	4.14	4.23	2.26	2.75	-0.59	0.002540	0.012629
RPL4	1,101.93	1,134.43	595.57	566.92	-0.62	0.000000	0.000000
GLCE	14.02	13.15	17.02	15.04	0.62	0.000000	0.000000
ARHGDIA	247.16	273.56	147.54	153.00	-0.54	0.000000	0.000000
VASP	47.79	50.16	53.99	53.44	0.45	0.000000	0.000001
CD248	393.08	430.06	226.32	259.95	-0.42	0.000000	0.000002
FGFR1	123.14	126.91	135.47	138.68	0.41	0.000000	0.000000
STAT2	46.25	46.36	50.88	48.72	0.45	0.000000	0.000000
TPM4	461.74	452.02	330.04	286.81	-0.19	0.002965	0.014488
TNFAIP6	17.85	16.51	31.92	26.28	1.09	0.000000	0.000000
TGOLN2	68.82	64.74	43.00	43.49	-0.33	0.000000	0.000005
SCARB1	30.64	32.58	38.17	40.99	0.66	0.000000	0.000000
ODC1	52.04	52.95	24.09	21.06	-0.87	0.000000	0.000000
IGFBP3	260.21	247.42	679.47	603.71	1.68	0.000000	0.000000
ROR2	8.11	9.42	10.02	10.90	0.68	0.000000	0.000000
SLC44A1	5.55	5.74	6.23	5.09	0.42	0.001112	0.006015
YWHAZ	194.07	197.84	142.27	123.38	-0.32	0.000002	0.000021
YBX1	300.42	290.10	159.25	138.40	-0.65	0.000000	0.000000
RUSC2	20.67	21.56	21.52	24.42	0.43	0.000009	0.000071
ACO1	71.36	71.04	26.60	24.28	-1.14	0.000000	0.000000
SREBF1	57.02	60.62	77.34	92.72	0.87	0.000000	0.000000
LAMB1	280.70	282.81	544.97	537.50	1.24	0.000000	0.000000
ANKRD13A	57.24	57.89	18.09	18.25	-1.39	0.000000	0.000000
DUSP5	16.41	16.63	8.57	7.77	-0.69	0.000000	0.000004
TWSG1	31.66	29.38	55.47	47.78	1.07	0.000000	0.000000

LAMA5	54.89	66.20	92.67	110.70	1.21	0.000000	0.000000
CYTL1	7.16	5.71	0.73	1.41	-2.28	0.000001	0.000007
CNN2	253.45	272.88	163.27	172.65	-0.45	0.000000	0.000000
EDNRB	0.24	0.11	1.40	1.50	3.46	0.000000	0.000000
MMP2	669.33	709.33	1,147.29	1,188.32	1.13	0.000000	0.000000
BMPER	16.86	17.22	6.64	5.88	-1.37	0.000000	0.000000
GAPDH	3,264.83	3,450.61	2,321.21	2,398.97	-0.17	0.003342	0.016135
HSP90AA1	378.09	364.25	119.71	102.96	-1.41	0.000000	0.000000
WSB1	58.27	57.98	79.33	72.83	0.70	0.000000	0.000000
PABPC1	360.51	337.23	185.03	164.38	-0.69	0.000000	0.000000
NPTX1	19.93	18.41	1.41	1.79	-3.53	0.000000	0.000000
TUFT1	10.90	11.14	10.55	11.06	0.33	0.010324	0.043362
PRSS23	67.26	63.17	68.20	67.76	0.33	0.000028	0.000201
FGF5	22.13	12.88	3.44	4.00	-1.55	0.000000	0.000000
ATP10A	12.75	12.25	15.75	16.88	0.76	0.000000	0.000000
TUBA1B	1,196.69	1,225.80	337.15	335.68	-1.51	0.000000	0.000000
WNT5B	47.60	45.84	28.29	33.21	-0.27	0.007946	0.034494
ADGRA3	29.45	28.02	19.80	16.84	-0.41	0.000002	0.000016
CTSB	625.06	660.54	411.13	439.21	-0.26	0.000004	0.000033
RAD23B	42.54	42.36	26.49	22.29	-0.38	0.000053	0.000369
ATP2B4	93.76	91.43	58.13	56.46	-0.37	0.000000	0.000000
AMIGO2	12.56	12.30	30.53	29.11	1.62	0.000000	0.000000
SCN7A	0.95	1.23	5.09	4.94	2.69	0.000000	0.000000
PCSK9	0.60	0.56	5.14	6.11	3.61	0.000000	0.000000
ITM2B	121.33	117.32	143.06	128.10	0.44	0.000000	0.000001
NRXN3	7.51	7.59	12.01	11.03	0.94	0.000000	0.000000
COL1A2	5,162.00	5,007.18	7,742.15	7,351.31	0.89	0.000000	0.000000
CALU	733.21	674.49	733.69	656.90	0.28	0.000039	0.000279
GPC1	213.82	231.53	248.55	277.32	0.54	0.000000	0.000000
TPM2	1,279.15	1,358.77	1,364.55	1,376.27	0.40	0.000000	0.000000
ALS2CL	1.28	1.23	1.75	2.15	0.81	0.005653	0.025604
POSTN	456.05	434.01	1,455.79	1,298.11	1.97	0.000000	0.000000
RPS2	1,377.94	1,479.78	766.20	806.01	-0.53	0.000000	0.000000
NRP2	93.29	87.20	92.50	89.47	0.39	0.000000	0.000000
HSPA8	989.88	944.34	420.00	368.29	-0.95	0.000000	0.000000
SLC4A4	5.97	5.85	3.61	2.97	-0.54	0.000031	0.000225
COTL1	161.29	164.36	64.95	62.07	-1.00	0.000000	0.000000
COL11A1	5.35	6.04	37.78	33.73	3.06	0.000000	0.000000
EEF1A1	4,195.96	4,140.03	2,202.40	1,945.09	-0.69	0.000000	0.000000
MYL9	358.78	379.16	366.92	402.09	0.41	0.000000	0.000001
PPME1	35.78	34.29	95.76	93.07	1.77	0.000000	0.000000
ACTB	3,509.84	3,792.46	1,149.33	1,256.91	-1.24	0.000000	0.000000
ITGA11	25.79	27.34	222.49	229.71	3.42	0.000000	0.000000

COL5A2	438.86	447.56	611.44	627.60	0.82	0.000000	0.000000
FGF7	34.50	30.76	36.77	29.44	0.36	0.002806	0.013789
CAPN2	147.16	158.81	94.38	98.17	-0.33	0.000000	0.000002
SH3BGRL3	275.84	288.37	147.06	158.61	-0.53	0.000000	0.000000
MGAT5	38.33	35.92	44.53	42.03	0.50	0.000000	0.000000
BMP4	3.67	4.12	25.21	23.85	3.14	0.000000	0.000000
MT-ND2	2,480.68	2,367.01	2,911.64	2,643.63	0.54	0.000000	0.000000
NOTCH2	49.15	45.83	44.78	44.23	0.24	0.000092	0.000618
FLNC	173.49	178.86	87.90	98.84	-0.58	0.000000	0.000000
PTX3	211.49	203.34	105.40	93.11	-0.73	0.000000	0.000000
EIF5A	292.01	298.79	136.57	133.11	-0.77	0.000000	0.000000
CYP51A1	23.81	20.72	44.98	36.80	1.24	0.000000	0.000000
STARD4	13.45	10.51	22.00	18.90	0.99	0.000000	0.000000
AKAP12	20.99	19.61	8.33	7.70	-1.03	0.000000	0.000000
EMILIN1	218.39	234.91	269.61	297.25	0.69	0.000000	0.000000
SRF	20.95	21.30	19.85	20.88	0.28	0.001790	0.009235
CAP1	178.39	180.51	188.21	176.37	0.31	0.000002	0.000017
FIBIN	6.11	7.09	19.80	17.14	1.82	0.000000	0.000000
FBLN1	120.30	124.20	333.19	355.52	1.79	0.000000	0.000000
HMGA1	207.98	231.57	34.81	38.44	-2.24	0.000000	0.000000
COL6A1	1,996.59	2,122.23	3,032.46	3,360.45	1.01	0.000000	0.000000
FN1	8,520.90	8,452.40	13,704.54	13,442.30	1.06	0.000000	0.000000
ACTN4	305.50	337.59	201.95	210.90	-0.30	0.000169	0.001082
PSG4	47.33	47.44	5.26	6.09	-2.63	0.000000	0.000000
LPIN1	12.18	11.64	12.68	13.00	0.32	0.001967	0.010039
CTSC	178.60	175.88	135.20	110.00	-0.24	0.002393	0.011963
KRT18	70.80	74.62	129.63	134.24	1.20	0.000000	0.000000
COL6A2	1,811.68	1,956.07	2,592.14	2,829.93	0.88	0.000000	0.000000
CRISPLD2	28.80	29.73	17.12	18.55	-0.46	0.000000	0.000001
PRDX6	162.93	160.32	223.66	210.83	0.73	0.000000	0.000000
TCF21	9.18	8.26	4.39	4.38	-0.65	0.000011	0.000084
TXNIP	3.20	3.25	7.50	7.37	1.50	0.000000	0.000000
WDR1	275.53	283.06	170.92	169.72	-0.37	0.000000	0.000000
LAPTM4A	233.46	217.05	258.62	214.41	0.41	0.000001	0.000005
NES	42.94	43.33	45.61	46.18	0.43	0.000000	0.000000
LOX	469.42	467.80	496.67	452.16	0.40	0.000000	0.000000
FLNB	90.28	88.76	114.79	119.76	0.72	0.000000	0.000000
OXTR	8.35	8.97	4.14	3.46	-0.79	0.000000	0.000001
TOP2A	54.15	49.40	1.72	2.17	-4.49	0.000000	0.000000
TUBB6	220.72	233.28	152.68	151.35	-0.27	0.000067	0.000462
CEMIP	66.84	67.07	46.27	46.95	-0.17	0.005633	0.025522
PTPRD	0.19	0.48	1.26	1.44	2.29	0.000000	0.000000
TAGLN	810.01	920.42	1,099.08	1,102.78	0.71	0.000000	0.000000

TUBA1A	503.10	519.14	346.87	334.12	-0.26	0.000010	0.000076
CFL1	678.00	726.51	437.73	435.66	-0.33	0.000000	0.000001
CALD1	200.97	193.88	236.87	215.62	0.60	0.000000	0.000000
VIM	1,532.94	1,556.99	713.63	682.97	-0.81	0.000000	0.000000
CTSK	8.35	8.41	13.85	12.94	1.03	0.000000	0.000000
THBS1	1,808.88	1,758.50	1,264.11	1,270.67	-0.17	0.001370	0.007258
PFN1	800.55	838.56	578.44	556.00	-0.17	0.005507	0.025010
RAB3B	10.89	10.21	4.19	4.36	-0.97	0.000000	0.000000
ACTG2	434.90	463.66	1,239.65	1,243.32	1.82	0.000000	0.000000
DDAH1	15.94	16.24	18.28	15.89	0.42	0.000041	0.000287
FBN2	43.12	41.45	67.77	64.80	0.99	0.000000	0.000000
MTATP6P1	980.39	927.71	1,118.14	964.49	0.48	0.000000	0.000000
NEAT1	132.37	130.14	78.14	69.84	0.27	0.000014	0.000109
IDI1	13.95	15.78	22.49	18.59	0.78	0.000000	0.000000
B2M	1,009.43	909.12	1,082.62	924.78	0.43	0.000000	0.000000
SCN9A	40.75	39.20	43.83	41.20	0.45	0.000000	0.000000
LUM	72.86	67.49	197.04	169.06	1.72	0.000000	0.000000
SEPTIN11	120.49	99.45	114.61	105.27	0.29	0.000014	0.000104
MT-ND3	1,287.26	1,229.49	2,201.38	1,989.94	1.13	0.000000	0.000000
TMSB10	2,307.00	2,500.58	1,119.36	1,153.02	-0.71	0.000000	0.000000
COL4A5	21.91	29.67	77.72	89.83	2.14	0.000000	0.000000
FGFR4	4.37	6.42	6.62	7.31	0.59	0.002444	0.012198
MFAP4	248.28	252.79	797.50	861.49	2.07	0.000000	0.000000
EXT1	148.02	150.50	87.62	82.74	-0.53	0.000000	0.000000
EDNRA	6.94	7.14	30.22	27.90	2.44	0.000000	0.000000
MT-CO3	9,163.73	9,219.48	9,071.83	8,327.24	0.27	0.000001	0.000010
TENM4	9.80	7.94	23.15	16.94	1.16	0.000000	0.000000
PALLD	78.99	77.87	97.76	93.06	0.63	0.000000	0.000000
MT-ND1	2,466.73	2,401.22	2,942.59	2,812.79	0.59	0.000000	0.000000
MEST	117.11	111.67	399.19	361.33	2.08	0.000000	0.000000
CDH6	69.12	63.90	33.41	34.98	-0.53	0.000000	0.000000
ARL6IP1	57.83	51.96	12.73	10.55	-1.93	0.000000	0.000000
MT-ATP6	5,786.75	5,698.81	6,351.99	5,654.18	0.42	0.000000	0.000000
TNFRSF19	40.53	36.88	45.02	43.14	0.52	0.000000	0.000000
PEA15	148.10	154.77	89.89	94.91	-0.27	0.000246	0.001529
MT-CO2	11,652.17	#######	11,128.51	10,183.35	0.21	0.000132	0.000861
CORO1C	153.63	139.89	61.58	61.74	-1.01	0.000000	0.000000
SERPINE1	863.65	862.63	892.82	912.67	0.40	0.000000	0.000000
COLEC10	4.15	3.50	1.41	1.12	-1.26	0.000002	0.000015
PODN	21.73	22.92	47.89	54.95	1.60	0.000000	0.000000
FTL	1,622.19	1,751.46	375.74	406.65	-1.76	0.000000	0.000000
HMGA2	29.76	30.38	14.00	12.26	-0.80	0.000000	0.000000
C7	3.31	0.63	18.66	18.94	3.94	0.000000	0.000000

SMURF2	68.28	66.13	26.60	27.24	-1.03	0.000000	0.000000
CXCL12	45.24	43.69	7.15	6.05	-2.41	0.000000	0.000000
ABCA1	8.02	7.39	12.41	11.53	0.98	0.000000	0.000000
ACACA	24.41	25.50	27.76	23.55	0.29	0.000146	0.000946
COL4A6	2.21	2.07	14.94	15.23	3.20	0.000000	0.000000
COL7A1	103.35	109.13	224.92	257.95	1.73	0.000000	0.000000
SCG2	2.63	2.79	5.78	5.46	1.39	0.000000	0.000000
SLC7A2	12.56	10.95	5.89	5.06	-0.80	0.000000	0.000000
MT-CYB	2,685.05	2,641.80	3,373.47	3,208.88	0.65	0.000000	0.000000
MICAL2	81.10	82.56	92.34	92.93	0.53	0.000000	0.000000
CORIN	23.97	24.37	13.02	14.14	-0.49	0.000003	0.000024
ACLY	110.82	111.28	106.61	114.16	0.29	0.000006	0.000048
ELN	27.36	32.56	307.27	331.76	3.78	0.000000	0.000000
ITGA3	110.46	121.06	112.55	123.59	0.41	0.000000	0.000000
ADAMTS5	0.56	0.50	0.93	0.85	0.97	0.000959	0.005269
JUP	14.45	15.45	30.10	32.33	1.40	0.000000	0.000000
LTBP2	114.69	119.52	131.69	140.72	0.59	0.000000	0.000000
MT-CO1	14,759.11	#######	13,486.87	12,570.34	0.17	0.001262	0.006752
HSPB1	336.12	382.91	395.91	432.14	0.55	0.000000	0.000000
BHLHE40	14.41	16.98	30.55	31.79	1.33	0.000000	0.000000
CCND1	113.66	115.42	39.39	43.46	-1.14	0.000000	0.000000
FDFT1	40.66	43.13	51.39	43.95	0.54	0.000000	0.000001
VCAM1	22.10	19.71	26.27	23.58	0.59	0.000000	0.000000
SREBF2	36.32	38.20	65.22	70.19	1.12	0.000000	0.000000
SMPDL3A	8.83	8.08	25.30	21.73	1.80	0.000000	0.000000
COL18A1	81.15	85.83	74.42	81.87	0.24	0.001385	0.007330
CDKN1A	170.22	178.12	266.29	264.45	0.94	0.000000	0.000000
LAMA4	164.06	154.30	255.86	251.24	1.05	0.000000	0.000000
LTBP1	228.15	225.33	217.41	211.32	0.29	0.000000	0.000002
ACAT2	26.30	26.03	33.60	29.14	0.56	0.000004	0.000033
SVEP1	8.93	8.06	13.11	13.01	0.96	0.000000	0.000000
ADARB1	50.02	32.04	5.25	6.41	-2.10	0.000000	0.000000
SGCD	5.86	5.62	26.72	23.10	2.74	0.000000	0.000000
CAVIN1	263.97	276.51	147.46	146.60	-0.54	0.000000	0.000000
STOM	54.88	47.19	59.00	50.60	0.45	0.000000	0.000003
SULF1	44.28	45.52	108.58	94.71	1.55	0.000000	0.000000
HTRA3	28.43	29.03	6.82	8.42	-1.56	0.000000	0.000000
MT-ND5	1,318.62	1,274.24	1,978.64	1,878.53	0.91	0.000000	0.000000
TIMP3	104.82	97.41	312.57	294.87	1.92	0.000000	0.000000
EFEMP1	145.77	133.06	100.80	94.70	-0.25	0.000139	0.000904
IGFBP4	583.30	616.49	240.74	249.67	-0.95	0.000000	0.000000
ACTA2	644.30	673.84	2,027.71	1,989.79	1.96	0.000000	0.000000
INSIG1	11.75	9.99	20.79	18.33	1.10	0.000000	0.000000

ACTG1	3,916.29	4,273.01	2,202.97	2,450.25	-0.55	0.000000	0.000000
FDPS	84.61	90.19	125.85	135.12	0.84	0.000000	0.000000
LDLR	38.83	37.89	55.46	60.60	0.88	0.000000	0.000000
NID1	98.63	94.40	144.33	143.61	0.92	0.000000	0.000000
MVD	33.49	38.05	50.66	53.43	0.86	0.000000	0.000000
ANXA2	1,231.99	1,253.32	706.58	656.41	-0.53	0.000000	0.000000
FRMD6	59.39	56.96	35.41	32.12	-0.48	0.000000	0.000000
PMP22	34.95	32.75	46.09	41.79	0.73	0.000000	0.000000
MSMO1	28.84	27.90	76.95	63.70	1.65	0.000000	0.000000
SEMA7A	41.61	44.51	101.57	102.96	1.64	0.000000	0.000000
IL4R	18.80	20.68	17.93	19.45	0.43	0.000100	0.000667
LTBP4	103.72	105.89	136.61	149.03	0.94	0.000000	0.000000
CPA4	93.71	93.33	197.22	190.16	1.40	0.000000	0.000000
CYP1B1	5.64	5.56	9.11	8.62	1.00	0.000000	0.000000
LRRC15	14.04	14.00	5.77	5.91	-0.92	0.000000	0.000000
PVR	104.21	106.78	139.57	141.92	0.81	0.000000	0.000000
PTGS1	84.21	87.23	183.59	187.33	1.38	0.000000	0.000000
TNFRSF11B	177.82	171.90	22.60	20.09	-2.79	0.000000	0.000000
CRIM1	140.80	136.31	87.59	88.57	-0.35	0.000000	0.000000
SCD	119.38	114.88	506.42	465.41	2.39	0.000000	0.000000
TPM1	597.46	590.99	729.65	704.14	0.68	0.000000	0.000000
HMGCR	33.01	31.28	58.96	57.94	1.23	0.000000	0.000000
DHCR7	54.57	53.06	93.60	92.84	1.08	0.000000	0.000000
FADS2	176.10	172.63	443.24	455.11	1.66	0.000000	0.000000
HGF	74.50	59.69	108.27	96.19	1.01	0.000000	0.000000
TNC	559.03	539.98	919.05	983.34	1.16	0.000000	0.000000
CCN1	441.04	429.28	392.90	373.05	0.17	0.002385	0.011925
C1S	174.57	170.16	244.54	222.72	0.79	0.000000	0.000000
COL14A1	3.02	3.41	27.44	29.05	3.52	0.000000	0.000000
C1R	242.91	254.56	221.28	228.39	0.22	0.001038	0.005651
ANTXR1	44.55	39.23	50.52	44.00	0.56	0.000000	0.000000
ADAMTS1	168.71	161.40	64.94	63.61	-1.00	0.000000	0.000000
ADAM19	61.88	61.95	185.24	183.81	1.90	0.000000	0.000000
ATP8B1	39.10	40.90	22.02	20.34	-0.55	0.000000	0.000000
GAS6	438.36	464.15	273.75	297.91	-0.32	0.000005	0.000038
COL3A1	1,415.60	1,445.89	3,437.75	3,558.49	1.63	0.000000	0.000000
IL1R1	18.81	18.79	19.33	17.17	0.35	0.000195	0.001232
HMGCS1	28.95	26.22	62.95	52.63	1.38	0.000000	0.000000
IL6ST	87.86	81.42	93.89	92.63	0.43	0.000000	0.000000
PLPP3	39.63	36.18	16.47	16.70	-0.86	0.000000	0.000000
SMOC1	8.64	7.83	9.02	9.81	0.46	0.002212	0.011160
CXCL1	4.34	5.25	11.41	8.95	1.37	0.000000	0.000002
DCN	527.54	488.84	878.71	750.70	0.96	0.000000	0.000000

QSOX1	1,080.95	1,127.99	1,600.59	1,713.63	0.91	0.000000	0.000000
IGFBP5	2,078.76	2,014.17	1,177.42	1,182.01	-0.46	0.000000	0.000000
C3	2.11	0.31	4.75	7.54	2.47	0.000000	0.000000
ADAMTS8	11.04	10.09	28.67	28.87	1.79	0.000000	0.000000
PLAT	134.25	132.58	241.96	235.72	1.15	0.000000	0.000000
PDGFRA	88.25	77.05	125.39	120.32	0.92	0.000000	0.000000
TRPA1	15.53	13.08	17.36	16.19	0.48	0.000004	0.000030
DHCR24	98.47	96.02	96.46	100.86	0.32	0.000000	0.000002
PLAU	393.03	377.04	350.85	338.57	0.18	0.001684	0.008733
BDKRB2	41.71	42.07	25.69	25.13	-0.37	0.000002	0.000014
TFPI2	50.90	47.81	15.91	15.99	-1.45	0.000000	0.000000
