# Supplementary material for: FRMD6 determines the cell fate towards senescence: involvement of the Hippo-YAP-CCN3 axis
Source: Cell Death Differ. 2024 Jun 26;31(11):1398–409. doi: 10.1038/s41418-024-01333-2 (PMC11519602; doi:10.1038/s41418-024-01333-2)
Supplement: Supplementary file 4 — Table S3 [file 41418_2024_1333_MOESM4_ESM.pdf]

Table S3. List of senescence related genes in GFP and GFP-FRMD6 expressed cells.

Symbol	EXP:GFP-1	EXP:GFP-2	EXP:GFP-FRMD6-1	EXP:GFP-FRMD6-2	DEG: LOG2FC (GFP-FRMD6	P-value	Q-value
AKT1	83.37	80.51	43.46	46.92	-0.5	0	0
ATM	15.97	14.06	16.83	15.54	0.51	0	3E-06
ATR	9.17	9.84	7.33	8	-0.32	0.0065	0.02904
CALM1	119.36	113.37	75.27	61.52	-0.29	0.0022	0.01103
CALM2	285.94	275.62	167.97	147.12	-0.41	0	0
CALM3	149.77	150.46	68.03	63.46	-0.9	0	0
CALML6	0.68	0.22	2.21	1.71	2.62	2E-05	0.00011
CAPN1	71.22	79.16	41.77	46.67	-0.48	0	0
CAPN2	147.16	158.81	94.38	98.17	-0.33	0	2E-06
CCNA2	14.78	15.8	1.31	1.06	-3.36	0	0
CCNB1	60.24	59.23	3.62	3.31	-3.87	0	0
CCNB2	18.93	19.81	1.16	1.37	-3.56	0	0
CCND1	113.66	115.42	39.39	43.46	-1.14	0	0
CCND2	0.19	0.34	3.26	2.87	4.32	0	0
CCND3	20.68	22.43	8.39	8.46	-1.04	0	0
CCNE1	2.18	2.64	0.86	0.77	-1.55	0.0008	0.00433
CCNE2	2.66	3.65	0.22	0.23	-3.42	0	0
CDK1	17.6	15.25	1.32	1.19	-3.44	0	0
CDK2	28.74	31.69	5.94	7.08	-1.45	0	0
CDK4	82.77	96.87	37.75	39.43	-0.88	0	0
CDKN1A	170.22	178.12	266.29	264.45	0.94	0	0
CDKN2B	3.29	3.28	13.45	11.9	2.18	0	0
CHEK1	7.64	8.47	3.96	3.63	-1.04	0	5E-06
CHEK2	7.03	8.9	3.12	2.8	-1.16	0.0002	0.00111
E2F1	8.54	9.48	0.34	0.53	-4.04	0	0
EIF4EBP1	61.41	68.46	69.85	65.42	0.41	0.0005	0.00312
ETS1	15.02	12.46	7.63	8.3	-0.41	0.001	0.00558
FOXM1	22.95	26.2	1.39	1.53	-3.92	0	0
FOXO3	7.96	7.58	5.05	4.74	-0.41	0.0003	0.0018
GADD45A	28.34	27.09	30.61	29.09	0.47	0.0002	0.00112
HIPK2	13.02	12.93	5.42	5.23	-0.87	0	0
HIPK3	6.35	5.6	8.14	7.04	0.67	0	0
IGFBP3	260.21	247.42	679.47	603.71	1.68	0	0
ITPR1	1.43	0.87	3.88	3.54	2.35	0	0
ITPR2	1.73	1.46	2.5	2.26	0.83	0	0
ITPR3	3.08	3.31	0.73	0.99	-1.56	0	0
LIN9	1.23	1.14	0.25	0.16	-2.23	5E-05	0.00036
MAP2K1	14.09	14.84	8.15	8.03	-0.42	0.0024	0.01191
MAP2K3	27.75	27.68	6	4.03	-2.39	0	0

MAP2K6	1.21	1.33	3.68	2.73	1.31	0	4E-06
MAPK1	21.07	20.27	14.77	15.99	-0.24	0.0099	0.04171
MAPK11	7.62	8.74	8.64	9.66	0.45	0.0077	0.0335
MAPKAPK2	21.4	21.34	8.64	11.38	-0.9	0	0
MDM2	29.88	31.52	48.81	48.93	1.11	0	0
MRE11	9.71	6.98	2.77	2.69	-1.22	0	0
MYBL2	17.72	18.85	1.11	1.21	-3.65	0	0
MYC	28.69	29.16	13.21	11.66	-0.85	0	0
NBN	10.83	8.76	7.08	4.81	-0.49	0.0006	0.0032
NFKB1	10.63	9.92	4.41	3.77	-0.98	0	0
PIK3CB	3.07	2.65	1.35	1.44	-0.7	0.0018	0.00931
PIK3CD	5.9	7.28	2.25	2.18	-1.25	0	0
PIK3R1	3.27	2.19	2.73	3.59	0.64	0.0003	0.00195
PIK3R2	13.13	15	5.8	8.05	-0.76	0	1E-06
PIK3R3	6.87	6.49	7.46	6.91	0.32	0.0103	0.0434
PPP1CA	59.86	65.31	32.2	32.98	-0.62	0	0
PPP1CC	35.35	35.79	21.16	18.66	-0.64	0	0
RAD9A	8.4	7.2	3.66	4.46	-0.66	0.0083	0.03567
RB1	5.34	5.8	3.59	3.68	-0.49	0.0029	0.01429
RBBP4	49.07	48.05	32.03	30.79	-0.28	0.0048	0.02198
RBL1	4.1	2.86	1.08	1.29	-1.7	0	0
RRAS2	26.02	25.84	15.69	16.67	-0.53	0.0001	0.0008
SERPINE1	863.65	862.63	892.82	912.67	0.4	0	0
SIRT1	5.01	5.38	2.9	2.42	-0.68	0.0006	0.0032
SLC25A5	77.48	79.09	25.19	22.17	-1.46	0	0
SLC25A6	191.06	208.27	92.99	98.78	-0.72	0	0
SMAD3	59.71	59.29	16.77	17.63	-1.44	0	0
TGFB1	72.83	78.16	108.31	112.74	0.87	0	0
TGFB2	14.79	14.34	17.49	15.5	0.48	2E-06	2.1E-05
TGFB3	3.13	2.78	7.8	8.15	1.64	0	0
TGFBR2	33.94	30.56	48.55	43.94	0.86	0	0
TSC1	15.07	16.39	18.21	16.76	0.51	0	0
VDAC1	97.81	105.05	60.38	57.83	-0.49	0	0
VDAC3	41.44	37.86	27.64	21.83	-0.44	0.0006	0.00348
ZFP36L2	15.73	15.8	10.39	9.23	-0.35	0.002	0.01004
