# Supplementary material for: FRMD6 determines the cell fate towards senescence: involvement of the Hippo-YAP-CCN3 axis
Source: Cell Death Differ. 2024 Jun 26;31(11):1398–409. doi: 10.1038/s41418-024-01333-2 (PMC11519602; doi:10.1038/s41418-024-01333-2)
Supplement: Supplementary file 5 — Table S4 [file 41418_2024_1333_MOESM5_ESM.pdf]

Table S4. List of YAP target genes in GFP and GFP-FRMD6 expressed cells.

Symbol	EXP:GFP-1	EXP:GFP-2	EXP:GFP-FRMD6-1	EXP:GFP-FRMD6-2	DEG: LOG2FC (GFP-FRMD6 vs [illegible]	P-value	Q-value
BIRC5	14.34	13.76	0.35	0.88	-4.72	5.5E-58	5.2E-56
MCM2	20.59	20.23	1.32	1.29	-3.57	8.3E-110	2.3E-107
CDK1	17.60	15.25	1.32	1.19	-3.44	2.0E-45	1.4E-43
LMNB1	31.91	32.71	2.54	2.63	-3.29	7.8E-110	2.1E-107
CCN3	5.87	5.78	0.42	0.62	-3.15	5.1E-23	1.6E-21
MCM3	29.19	31.82	3.36	3.67	-2.79	1.2E-102	3.0E-100
POLA2	8.16	8.34	1.21	1.40	-2.66	2.8E-27	1.1E-25
POLA1	3.99	3.37	0.80	0.69	-1.93	5.4E-18	1.3E-16
CCND1	113.66	115.42	39.39	43.46	-1.14	5.3E-66	6.2E-64
CCND3	20.68	22.43	8.39	8.46	-1.04	2.4E-12	4.0E-11
MYC	28.69	29.16	13.21	11.66	-0.85	1.6E-11	2.5E-10
VIM	1,532.94	1,556.99	713.63	682.97	-0.81	4.2E-51	3.3E-49
AMOTL1	13.16	12.80	7.49	7.34	-0.60	1.4E-11	2.2E-10
RAC1	107.00	106.11	63.33	58.27	-0.51	1.1E-8	1.3E-7
ZEB2	12.90	12.12	7.11	7.45	-0.45	5.5E-5	3.8E-4
RHOA	181.94	182.34	112.99	102.73	-0.38	2.6E-7	2.5E-6
CDC42	73.93	77.02	55.87	46.49	-0.25	1.1E-2	4.5E-2
ZEB1	31.96	29.15	39.04	36.69	0.67	5.3E-18	1.3E-16
CDH2	59.48	56.68	134.10	121.98	1.48	1.8E-112	5.3E-110
ANKRD1	1.23	1.27	3.37	3.46	1.79	4.6E-8	4.8E-7
CCND2	0.19	0.34	3.26	2.87	4.32	4.0E-52	3.2E-50
