# Supplementary material for: FRMD6 determines the cell fate towards senescence: involvement of the Hippo-YAP-CCN3 axis
Source: Cell Death Differ. 2024 Jun 26;31(11):1398–409. doi: 10.1038/s41418-024-01333-2 (PMC11519602; doi:10.1038/s41418-024-01333-2)
Supplement: Supplementary file 6 — Original Data [file 41418_2024_1333_MOESM6_ESM.pdf]

Fig. 1

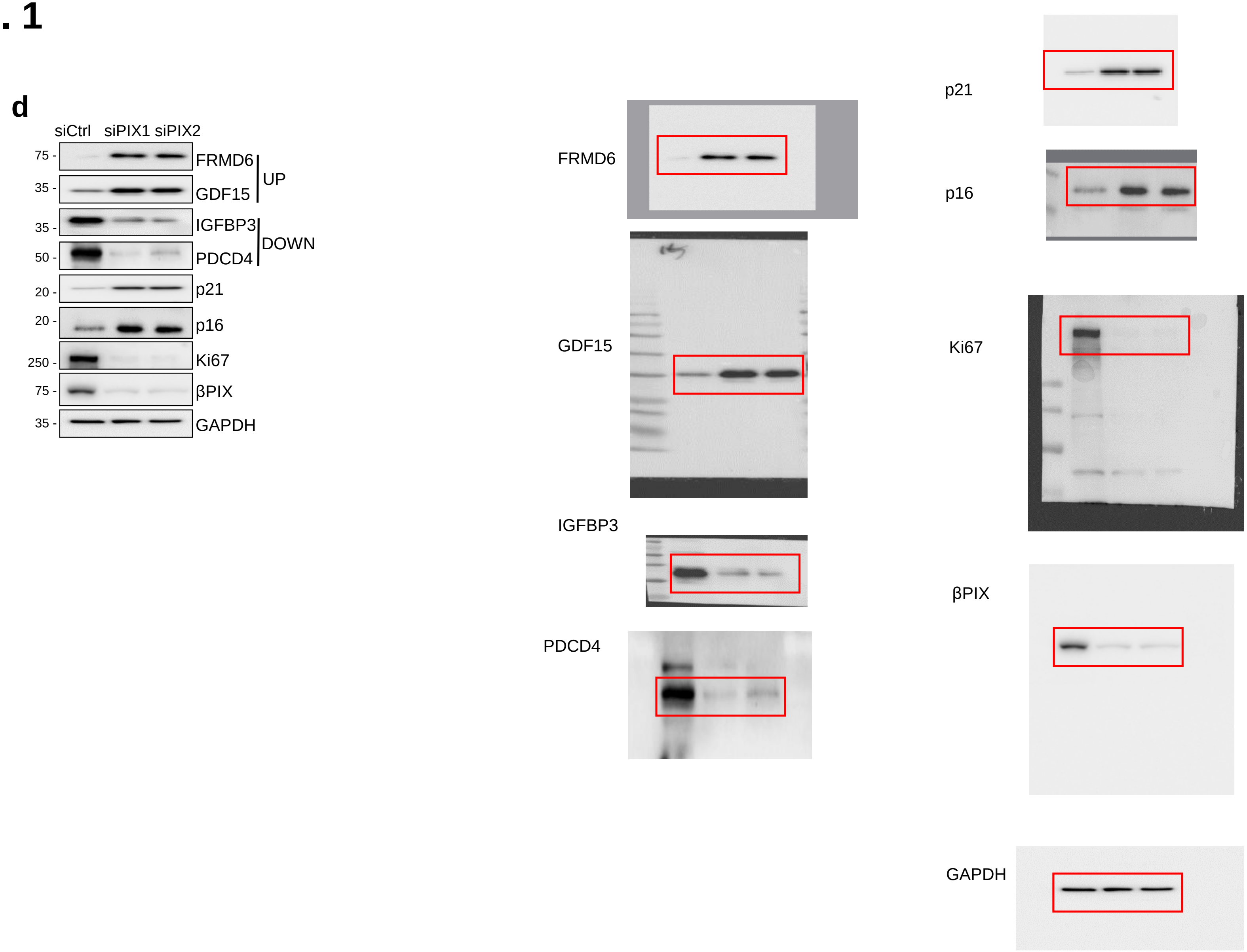
d
siCtrl
siPIX1
siPIX2
75 -
35 -
35 -
50 -
20 -
20 -
250 -
75 -
35 -
FRMD6
GDF15
UP
IGFBP3
PDCD4
DOWN
p21
p16
Ki67
βPIX
GAPDH
FRMD6
GDF15
IGFBP3
PDCD4
p21
p16
Ki67
βPIX
GAPDH

Fig. 1

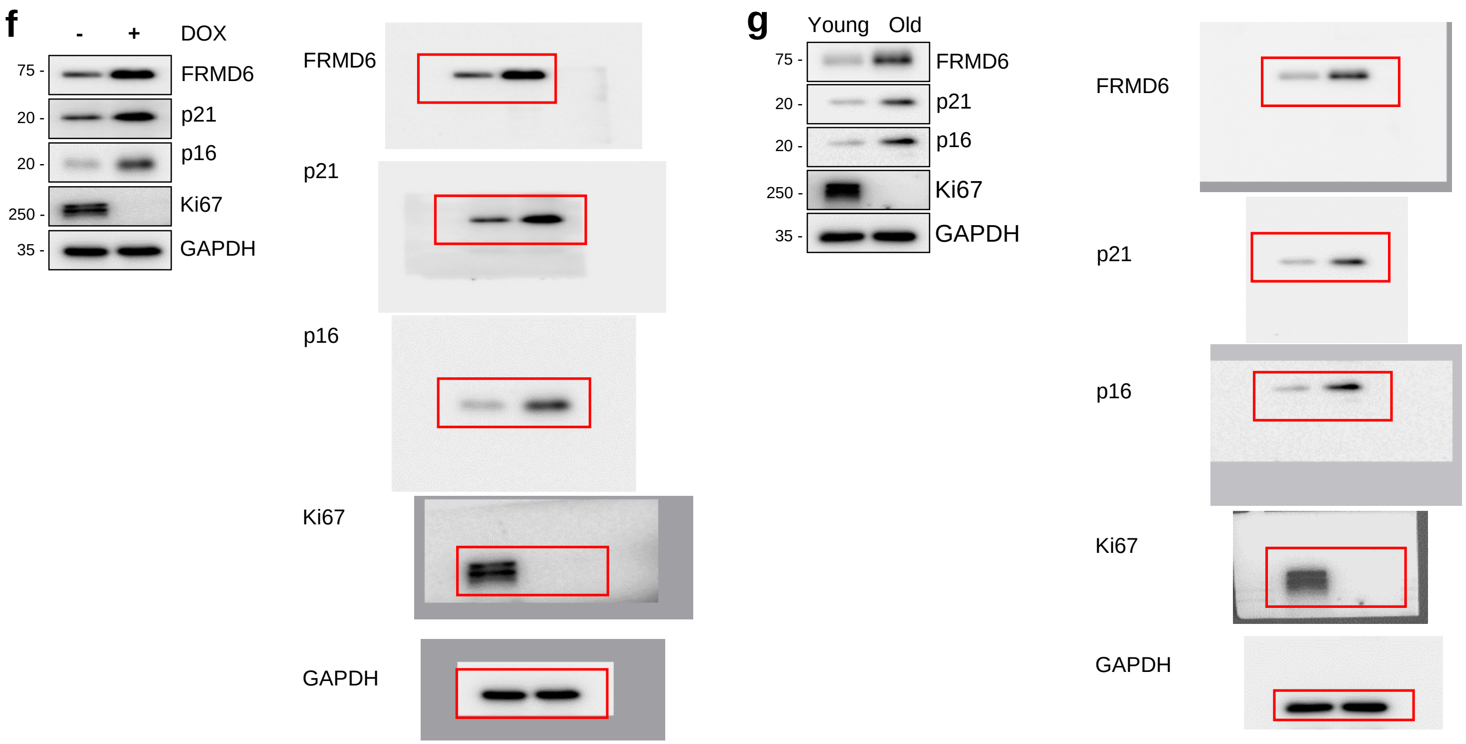
f
-
+
DOX
75 -
FRMD6
20 -
p21
20 -
p16
250 -
Ki67
35 -
GAPDH
FRMD6
p21
p16
Ki67
GAPDH
g
Young
Old
75 -
FRMD6
20 -
p21
20 -
p16
250 -
Ki67
35 -
GAPDH
FRMD6
p21
p16
Ki67
GAPDH

Fig. 2

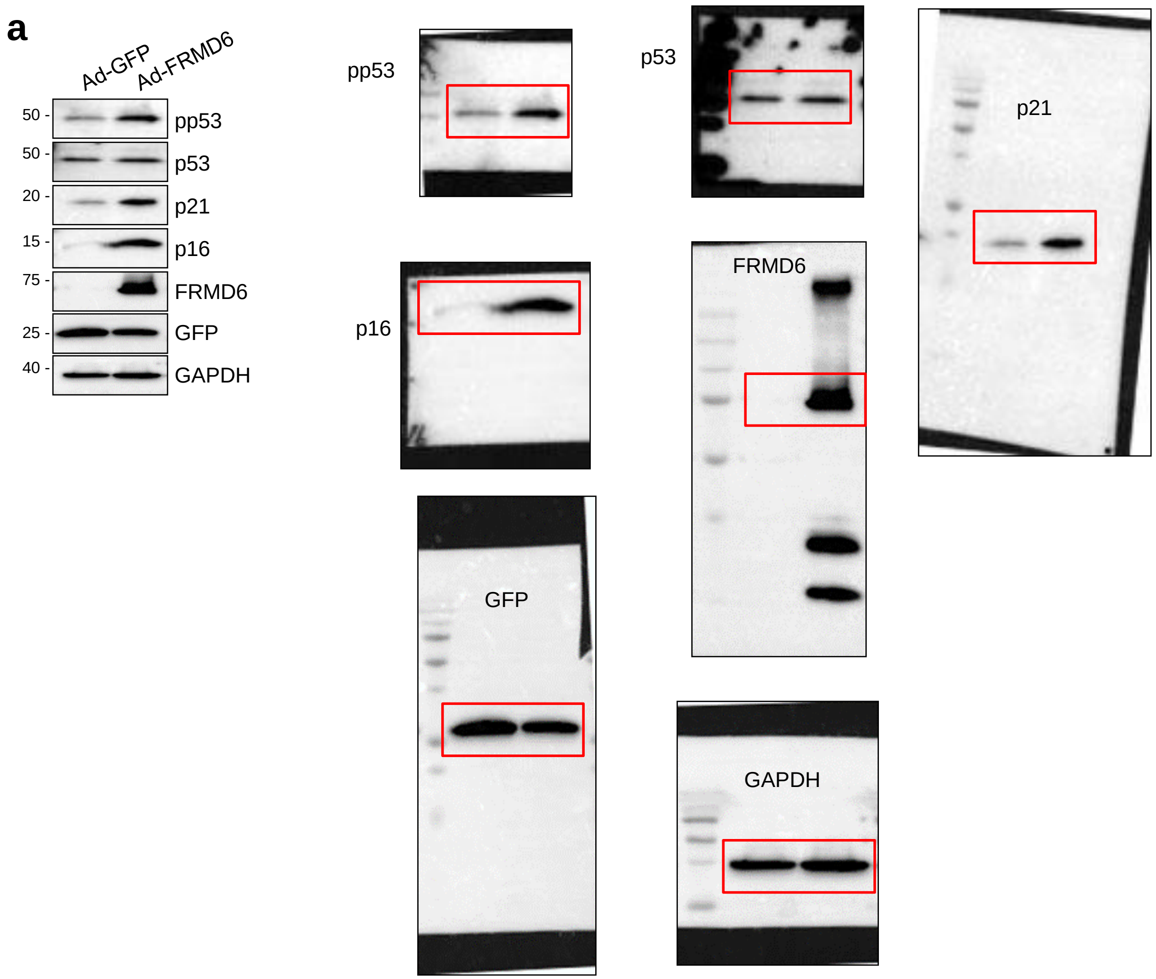
a
Ad-GFP
Ad-FRMD6
50 -
pp53
50 -
p53
20 -
p21
15 -
p16
75 -
FRMD6
25 -
GFP
40 -
GAPDH
pp53
p53
p21
p16
FRMD6
GFP
GAPDH

Fig. 2

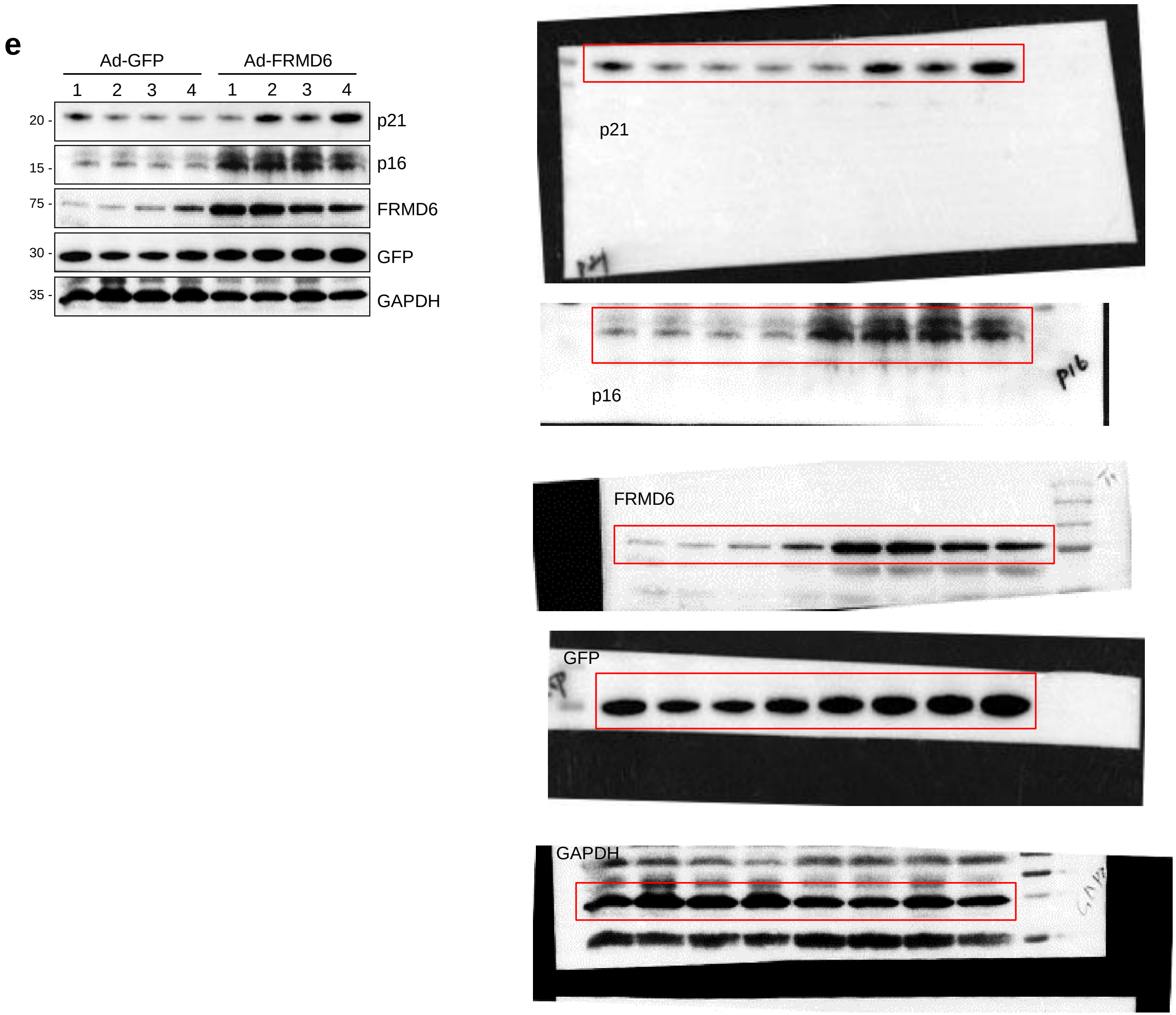
e
Ad-GFP
Ad-FRMD6
1 2 3 4 1 2 3 4
20 -
15 -
75 -
30 -
35 -
p21
p16
FRMD6
GFP
GAPDH
p21
p16
FRMD6
GFP
GAPDH

Fig. 3

a

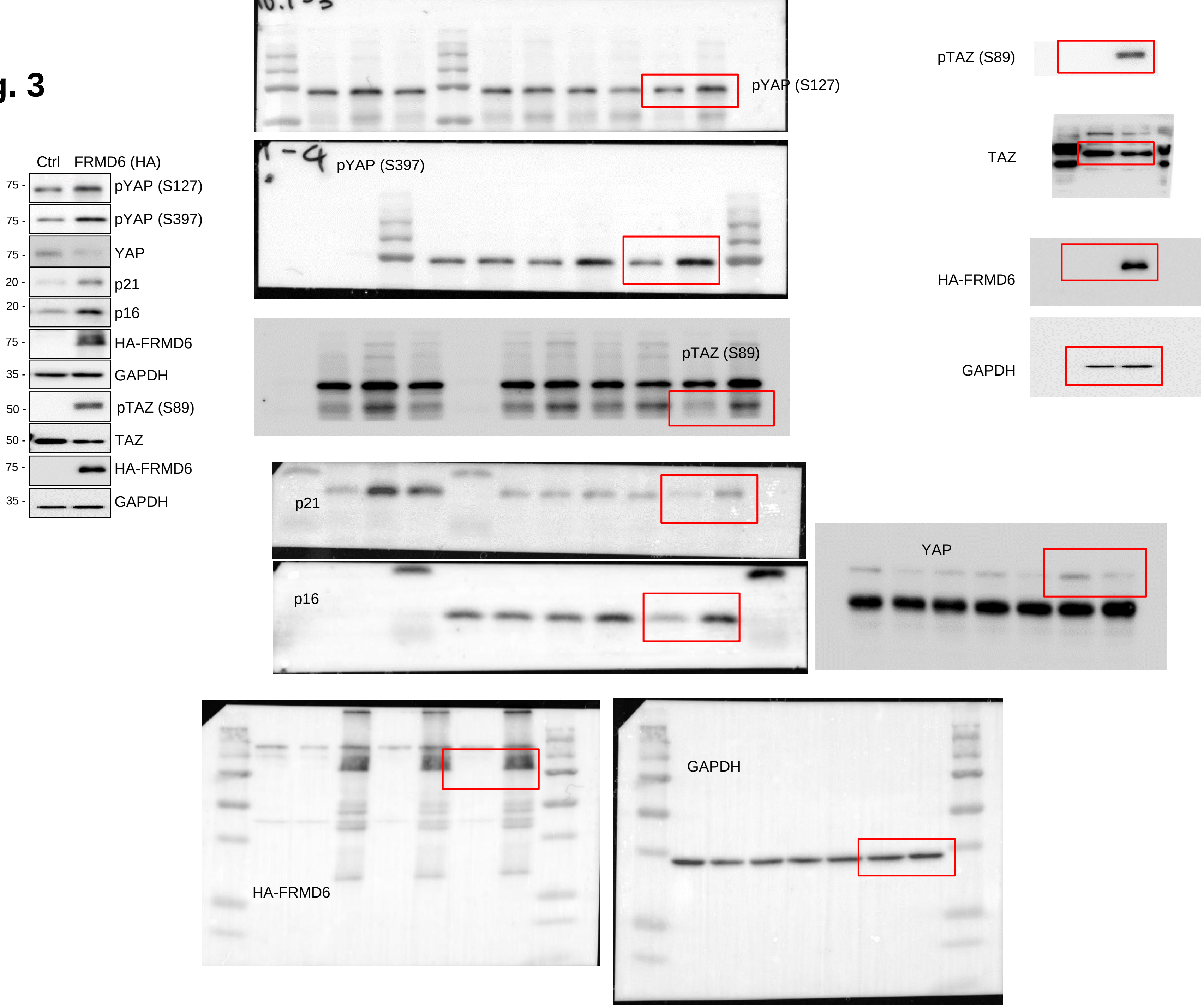
Ctrl
FRMD6 (HA)
75 -
pYAP (S127)
75 -
pYAP (S397)
75 -
YAP
20 -
p21
20 -
p16
75 -
HA-FRMD6
35 -
GAPDH
50 -
pTAZ (S89)
50 -
TAZ
75 -
HA-FRMD6
35 -
GAPDH
pYAP (S127)
pYAP (S397)
pTAZ (S89)
p21
p16
YAP
HA-FRMD6
GAPDH
pTAZ (S89)
TAZ
HA-FRMD6
GAPDH

Fig. 3

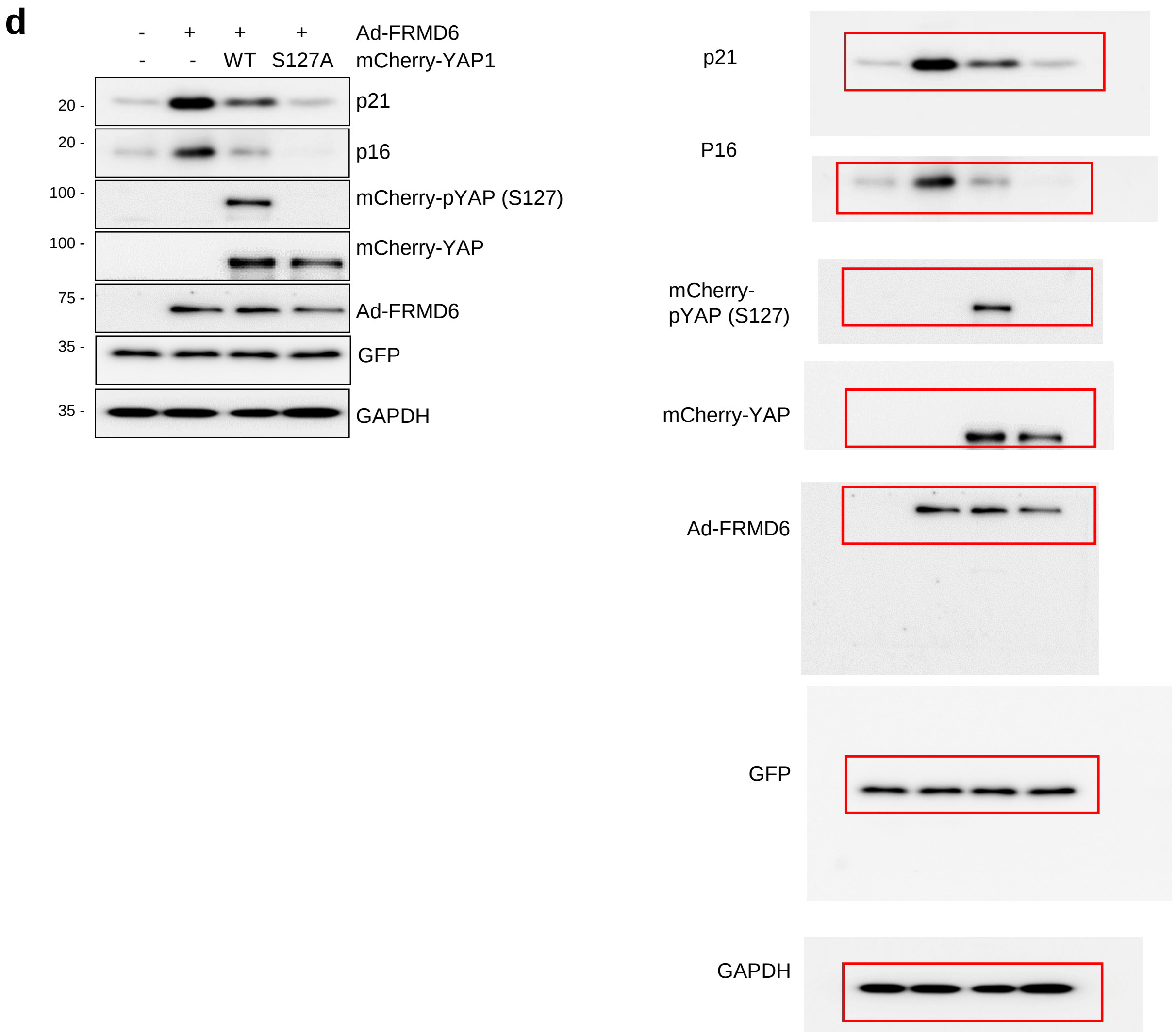
d
- + + + Ad-FRMD6
- - WT S127A mCherry-YAP1
20 -
p21
20 -
p16
100 -
mCherry-pYAP (S127)
100 -
mCherry-YAP
75 -
Ad-FRMD6
35 -
GFP
35 -
GAPDH
p21
P16
mCherry-pYAP (S127)
mCherry-YAP
Ad-FRMD6
GFP
GAPDH

Fig. 4

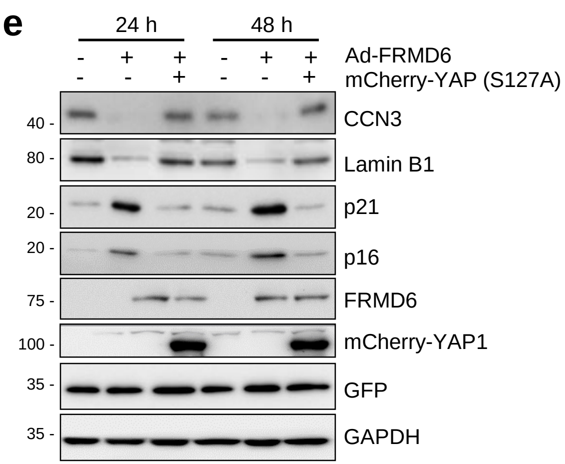
e
24 h
48 h
- + + - + +
Ad-FRMD6
- - + - - +
mCherry-YAP (S127A)
40 -
CCN3
80 -
Lamin B1
20 -
p21
20 -
p16
75 -
FRMD6
100 -
mCherry-YAP1
35 -
GFP
35 -
GAPDH

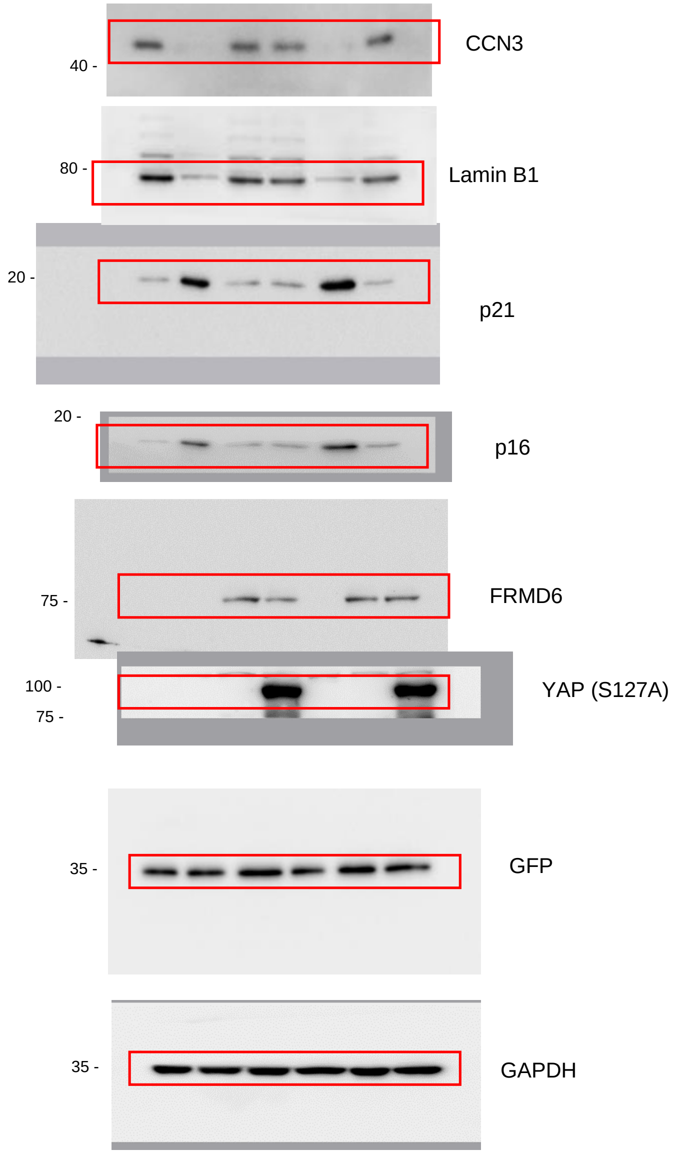
40 -
CCN3
80 -
Lamin B1
20 -
p21
20 -
p16
75 -
FRMD6
100 -
75 -
YAP (S127A)
35 -
GFP
35 -
GAPDH

Fig. 4

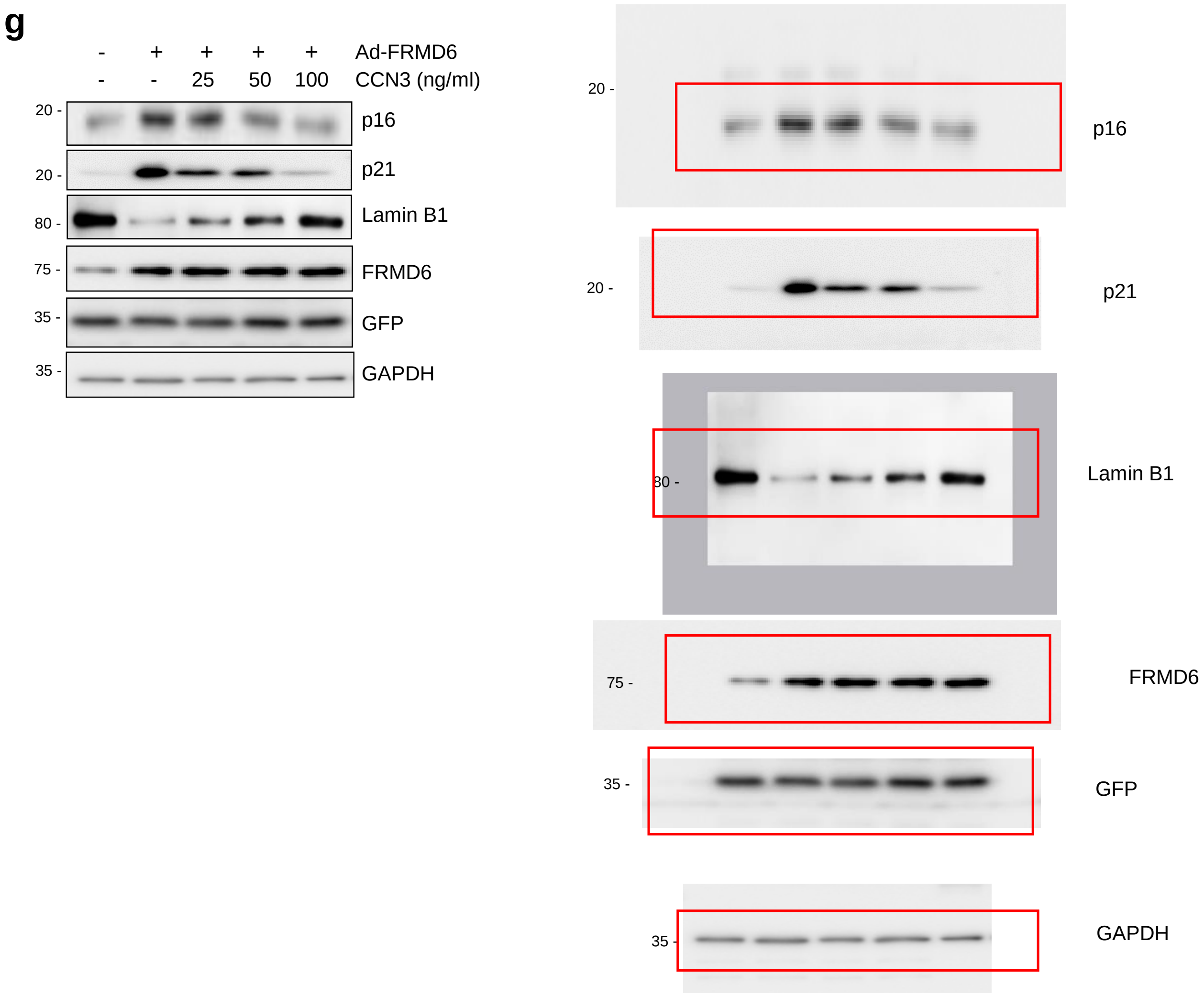
g
- + + + + Ad-FRMD6
- - 25 50 100 CCN3 (ng/ml)
20 -
p16
20 -
p21
80 -
Lamin B1
75 -
FRMD6
35 -
GFP
35 -
GAPDH
20 -
p16
20 -
p21
80 -
Lamin B1
75 -
FRMD6
35 -
GFP
35 -
GAPDH

Fig. 5

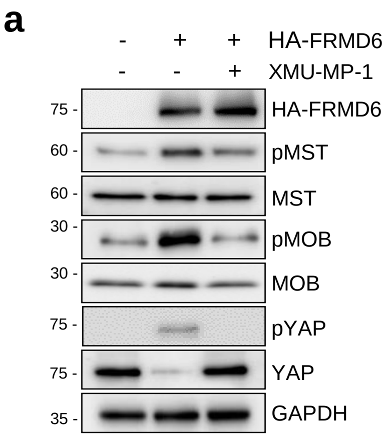
a
- + + HA-FRMD6
- - + XMU-MP-1
75 - HA-FRMD6
60 - pMST
60 - MST
30 - pMOB
30 - MOB
75 - pYAP
75 - YAP
35 - GAPDH

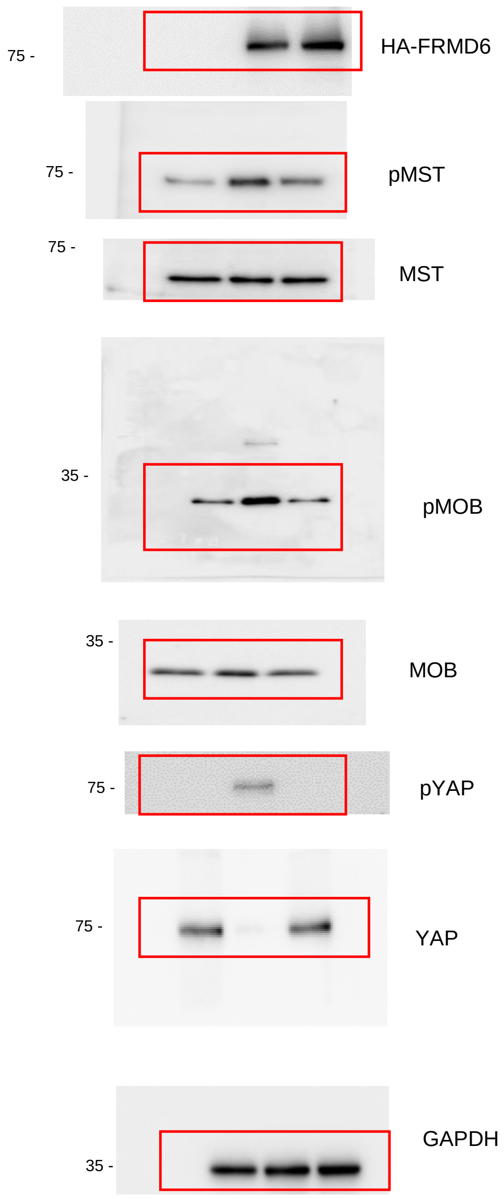
75 - HA-FRMD6
75 - pMST
75 - MST
35 - pMOB
35 - MOB
75 - pYAP
75 - YAP
35 - GAPDH

Fig. 5

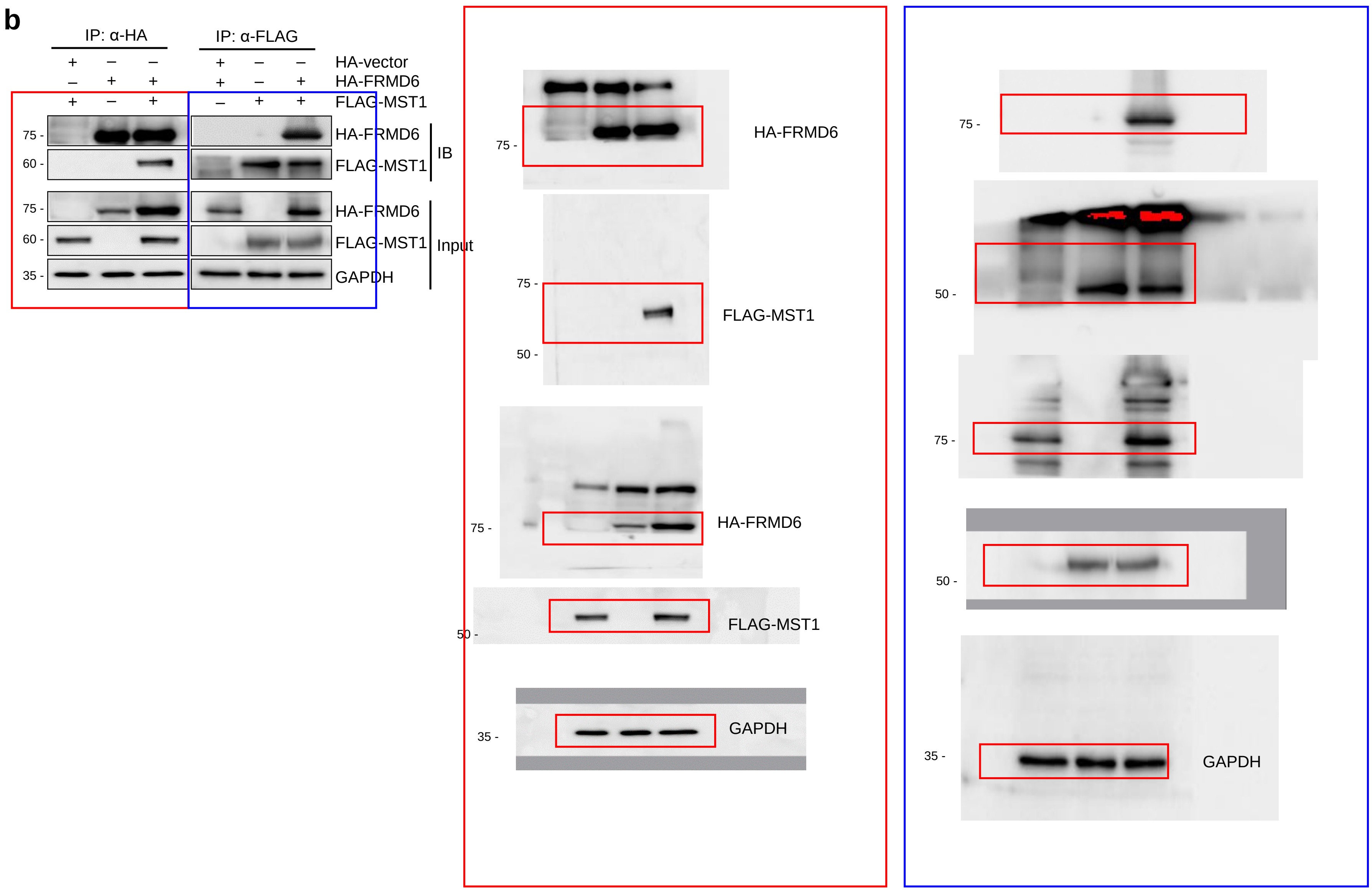
b
IP: α-HA
IP: α-FLAG
HA-vector
HA-FRMD6
FLAG-MST1
75 -
60 -
75 -
60 -
35 -
HA-FRMD6
FLAG-MST1
IB
HA-FRMD6
FLAG-MST1
Input
GAPDH
75 -
HA-FRMD6
75 -
50 -
FLAG-MST1
75 -
HA-FRMD6
50 -
FLAG-MST1
35 -
GAPDH
75 -
50 -
75 -
50 -
35 -
GAPDH

Fig. 5

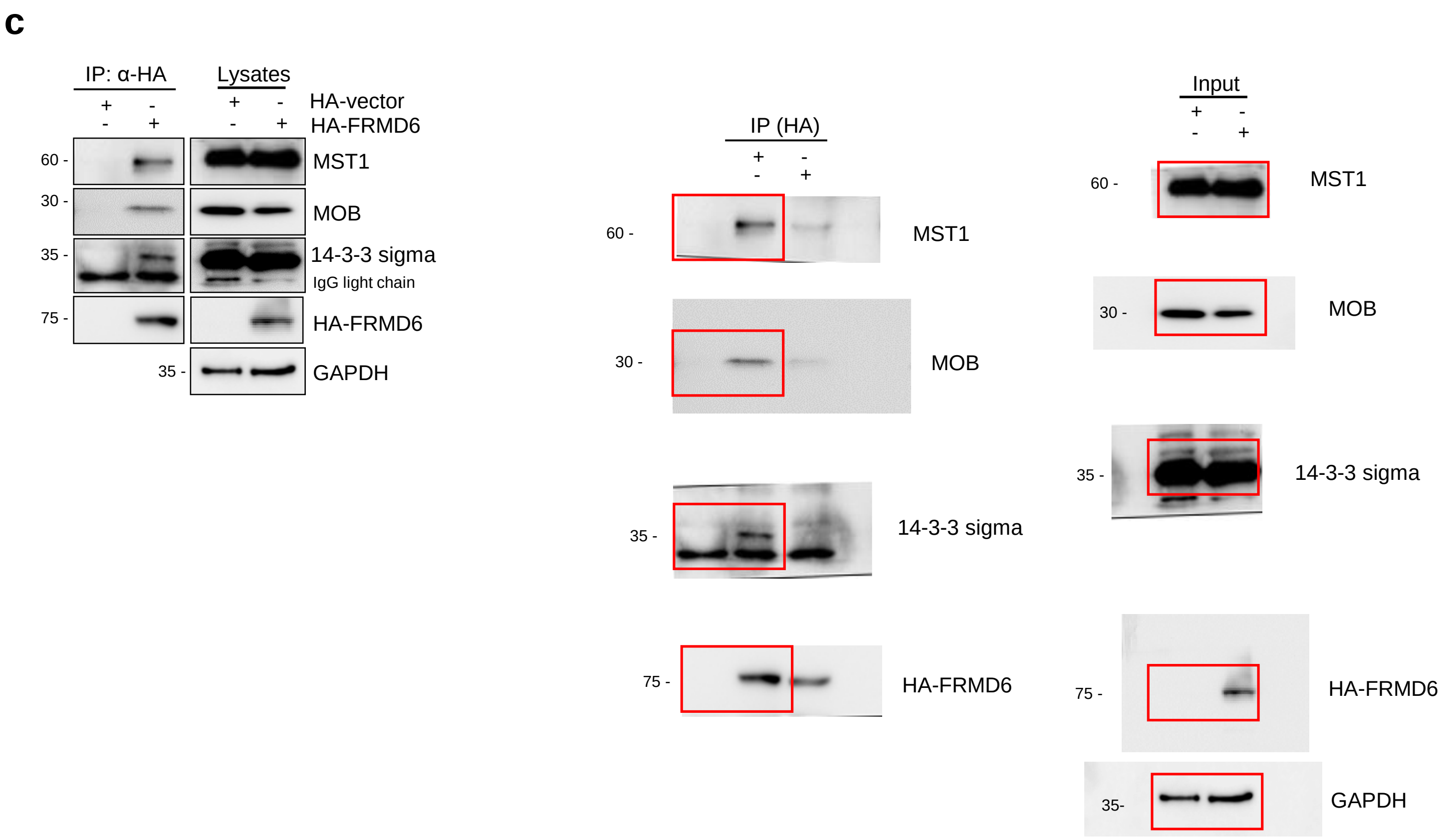
c
IP: α-HA
Lysates
HA-vector
HA-FRMD6
60 -
30 -
35 -
75 -
MST1
MOB
14-3-3 sigma
IgG light chain
HA-FRMD6
GAPDH
IP (HA)
Input
35-

Fig. 5

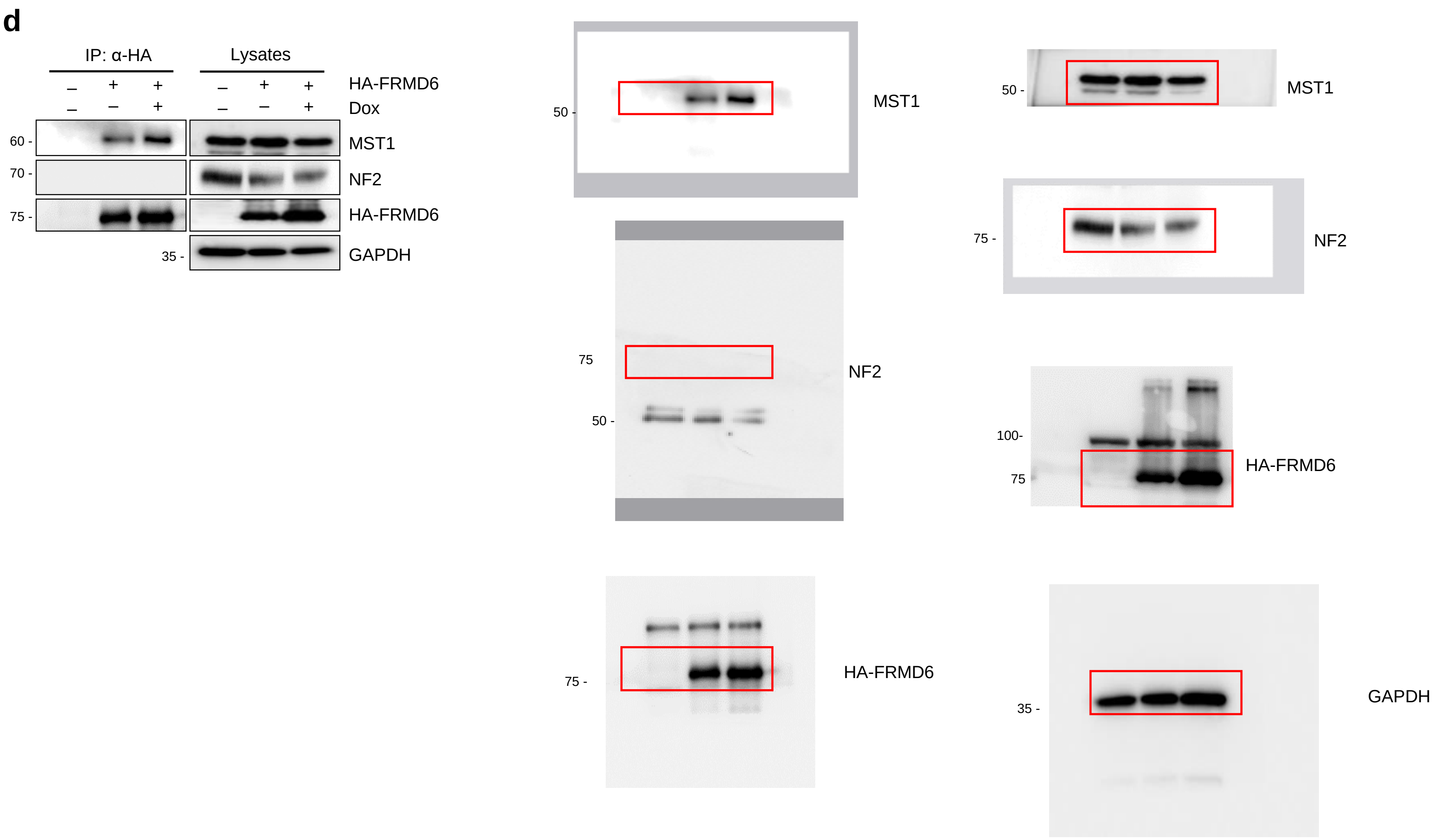
d
IP: α-HA
Lysates
HA-FRMD6
Dox
60 -
70 -
75 -
35 -
MST1
NF2
HA-FRMD6
GAPDH
50 -
75
50 -
100-
75

Fig. 5

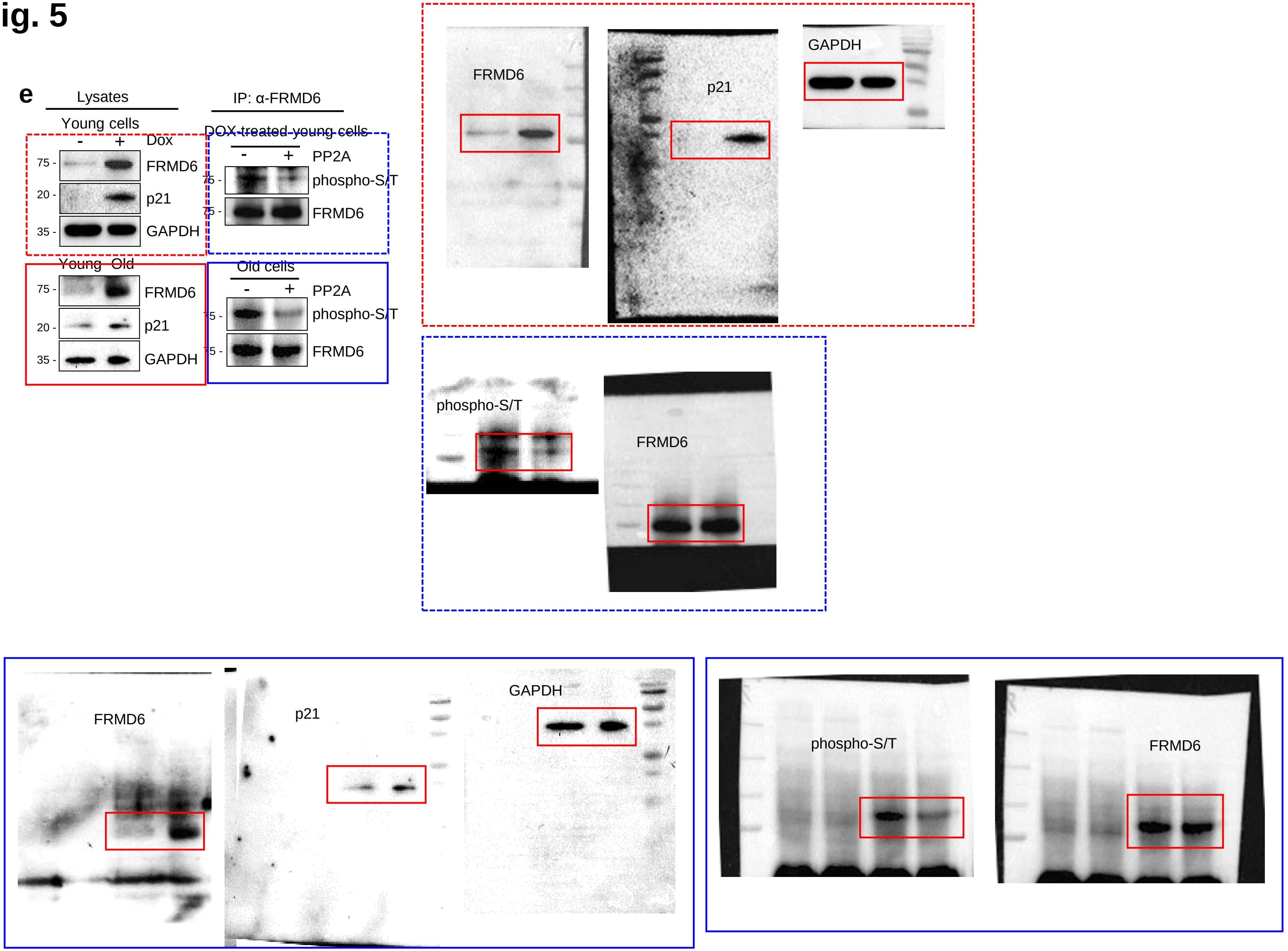

e
Lysates
IP: α-FRMD6
Young cells
DOX-treated young cells
- + Dox
- + PP2A
75 -
FRMD6
20 -
p21
35 -
GAPDH
phospho-S/T
FRMD6
Young Old
Old cells
FRMD6
p21
GAPDH
PP2A
phospho-S/T
FRMD6
FRMD6
p21
GAPDH
phospho-S/T
FRMD6
FRMD6
p21
GAPDH
phospho-S/T
FRMD6

Fig. 5

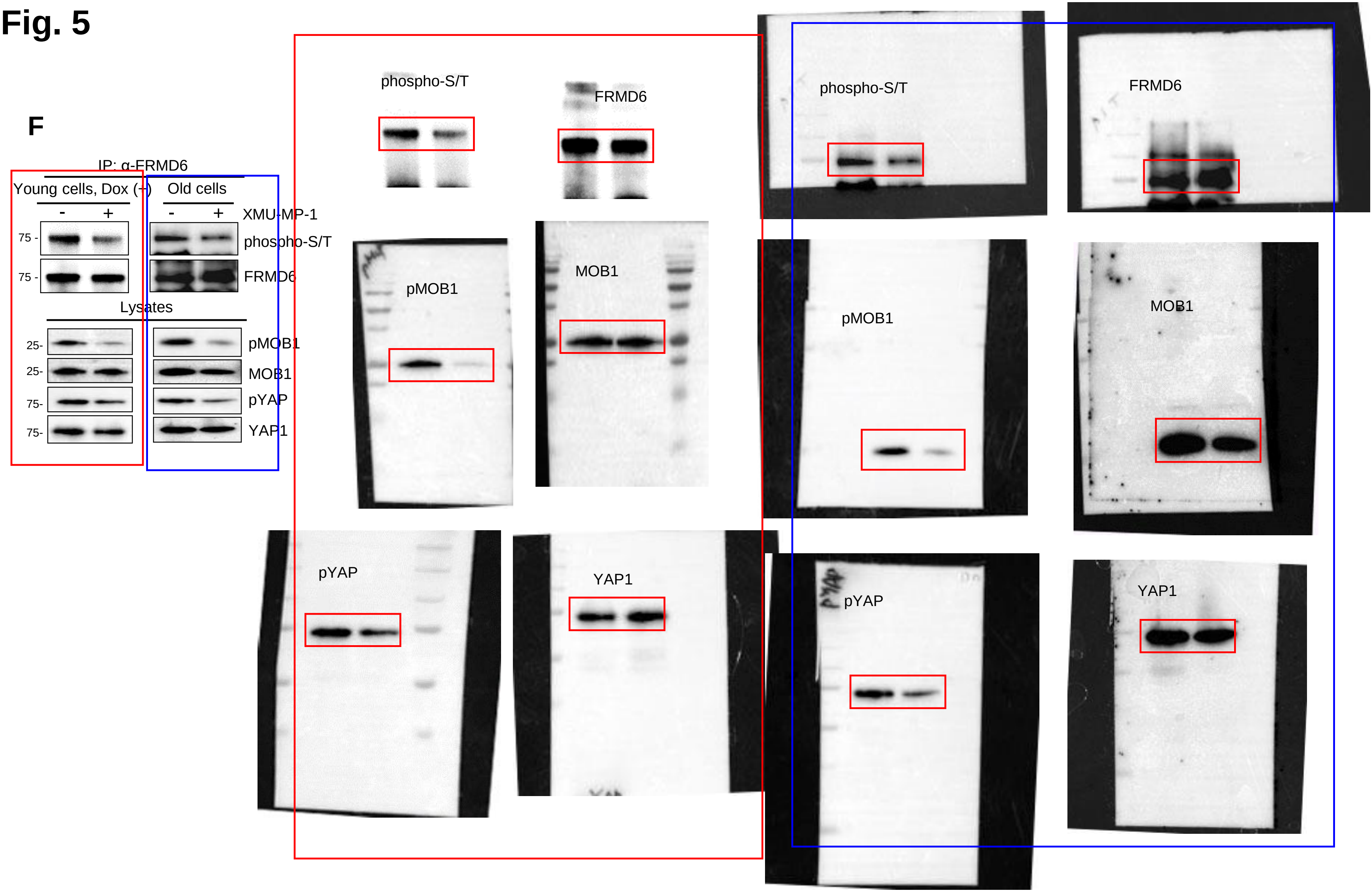
F
IP: α-FRMD6
Young cells, Dox (+)
Old cells
- + - +
XMU-MP-1
75 -
phospho-S/T
75 -
FRMD6
Lysates
25-
pMOB1
25-
MOB1
75-
pYAP
75-
YAP1
phospho-S/T
FRMD6
pMOB1
MOB1
pYAP
YAP1
phospho-S/T
FRMD6
pMOB1
MOB1
pYAP
YAP1

Fig. 5

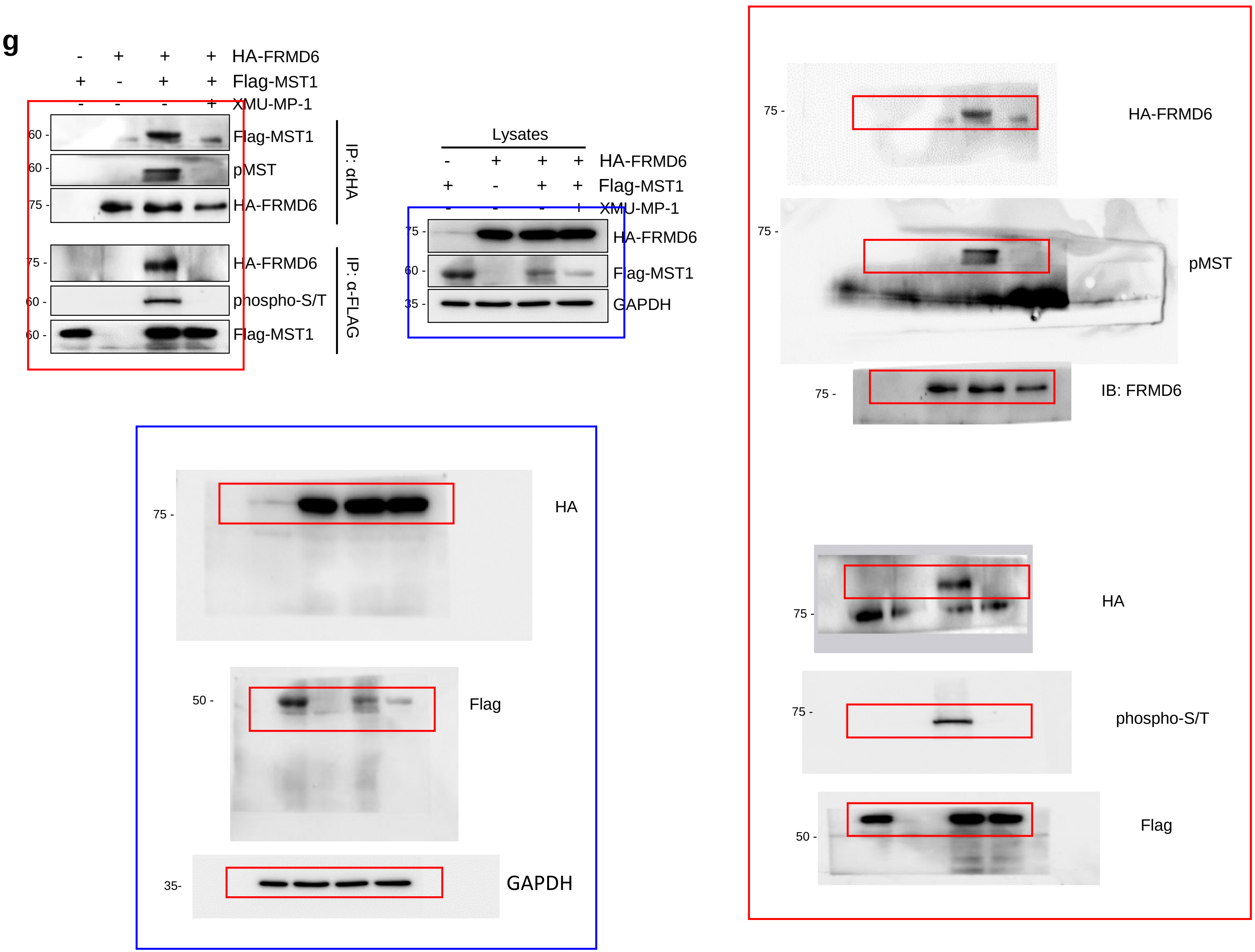
g
HA-FRMD6
Flag-MST1
XMU-MP-1
Flag-MST1
pMST
HA-FRMD6
HA-FRMD6
phospho-S/T
Flag-MST1
IP: αHA
IP: α-FLAG
Lysates
HA-FRMD6
Flag-MST1
XMU-MP-1
HA-FRMD6
Flag-MST1
GAPDH
HA
Flag
GAPDH
HA-FRMD6
pMST
IB: FRMD6
HA
phospho-S/T
Flag

Fig. 6

b

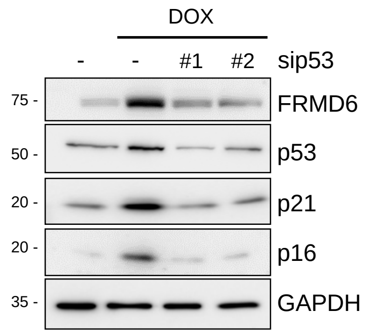
DOX
- - #1 #2 sip53
75 - FRMD6
50 - p53
20 - p21
20 - p16
35 - GAPDH

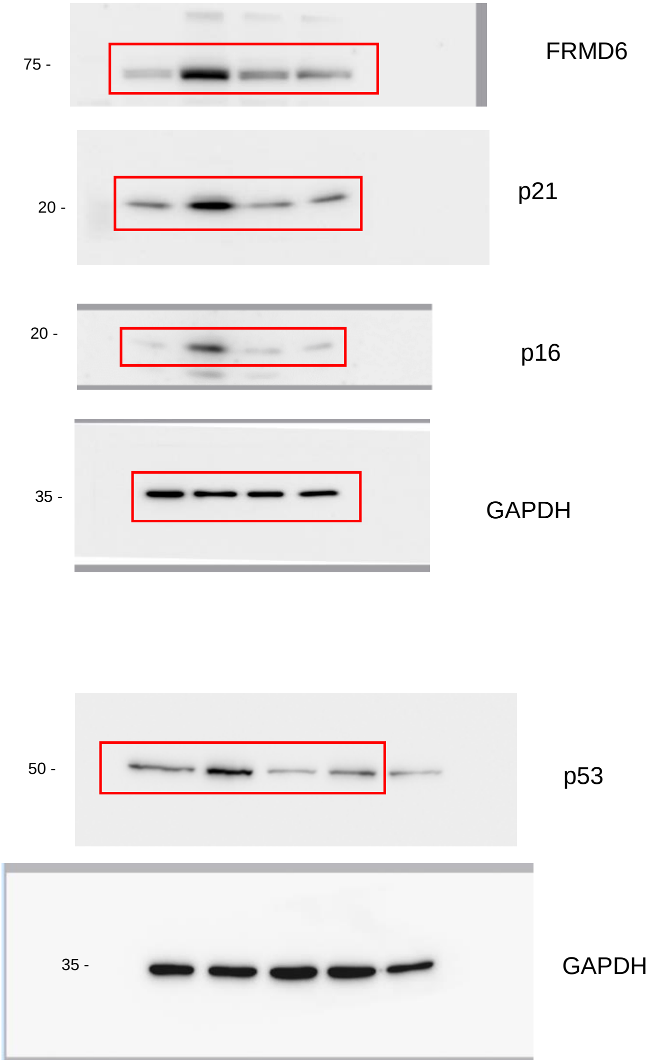
75 - FRMD6
20 - p21
20 - p16
35 - GAPDH
50 - p53
35 - GAPDH

Fig. 6

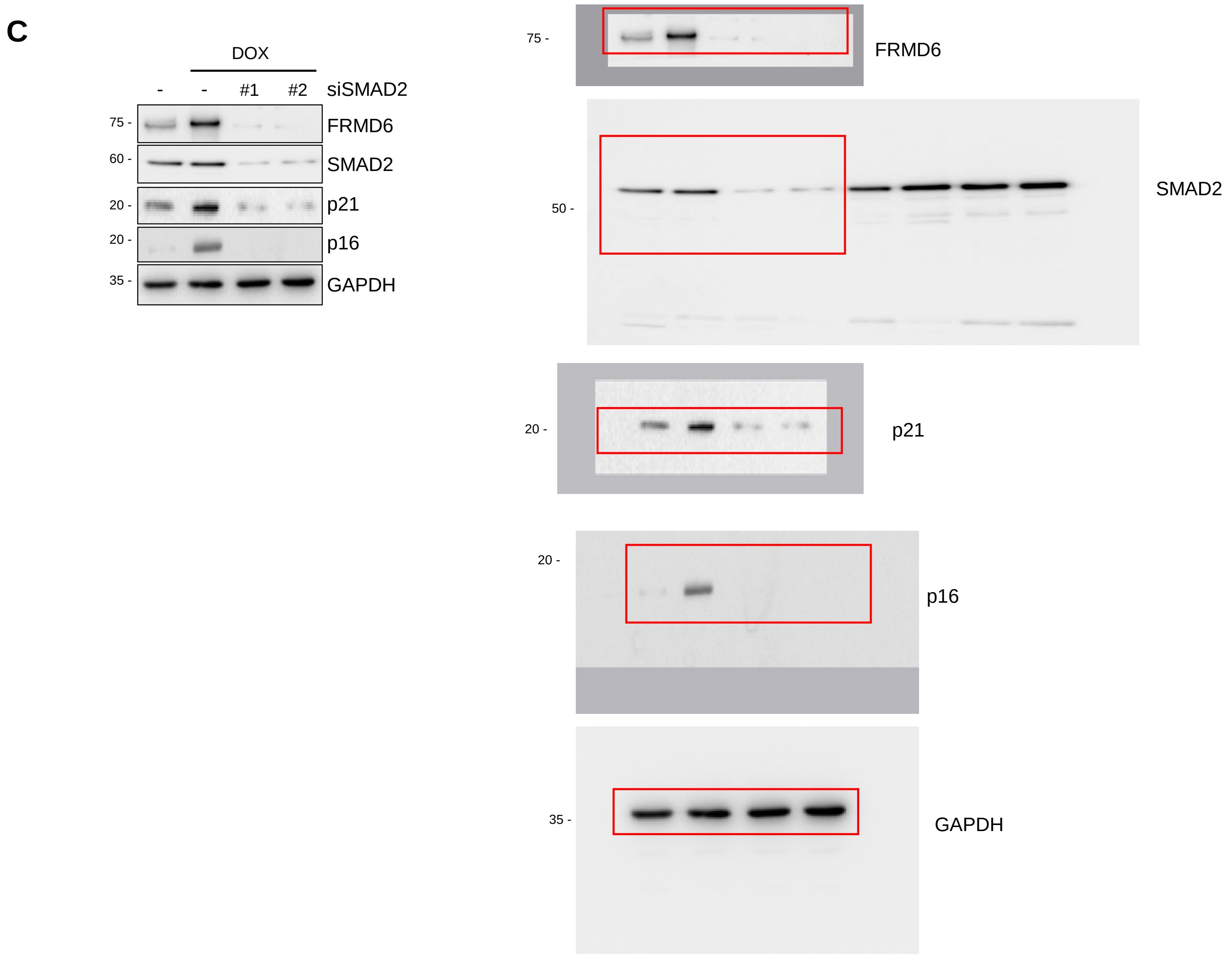
C
DOX
-
-
#1
#2
siSMAD2
75 -
FRMD6
60 -
SMAD2
20 -
p21
20 -
p16
35 -
GAPDH
75 -
FRMD6
50 -
SMAD2
20 -
p21
20 -
p16
35 -
GAPDH

Fig. 6

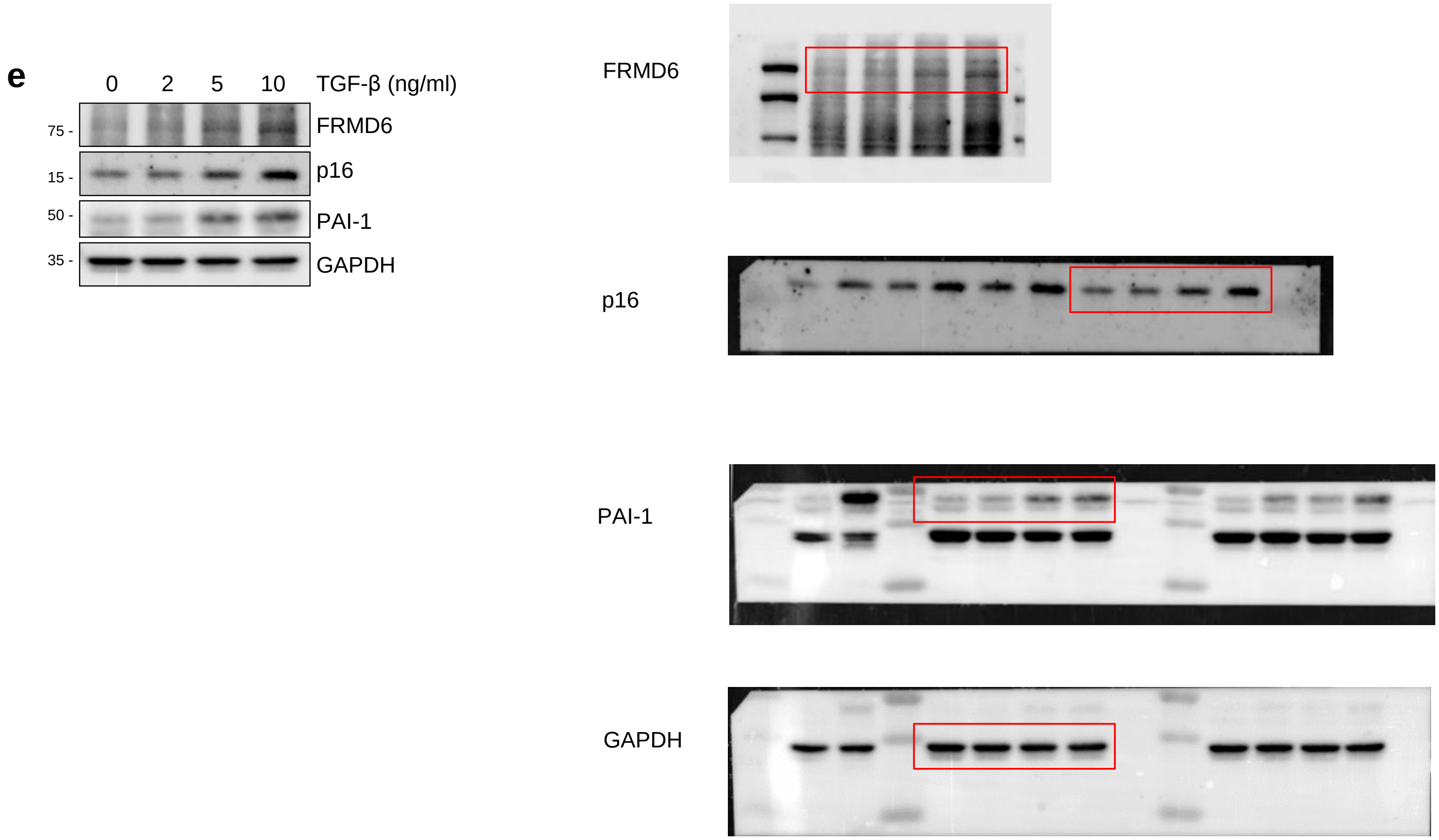

e
0
2
5
10
TGF-β (ng/ml)
75 -
FRMD6
15 -
p16
50 -
PAI-1
35 -
GAPDH
FRMD6
p16
PAI-1
GAPDH

Fig. 6

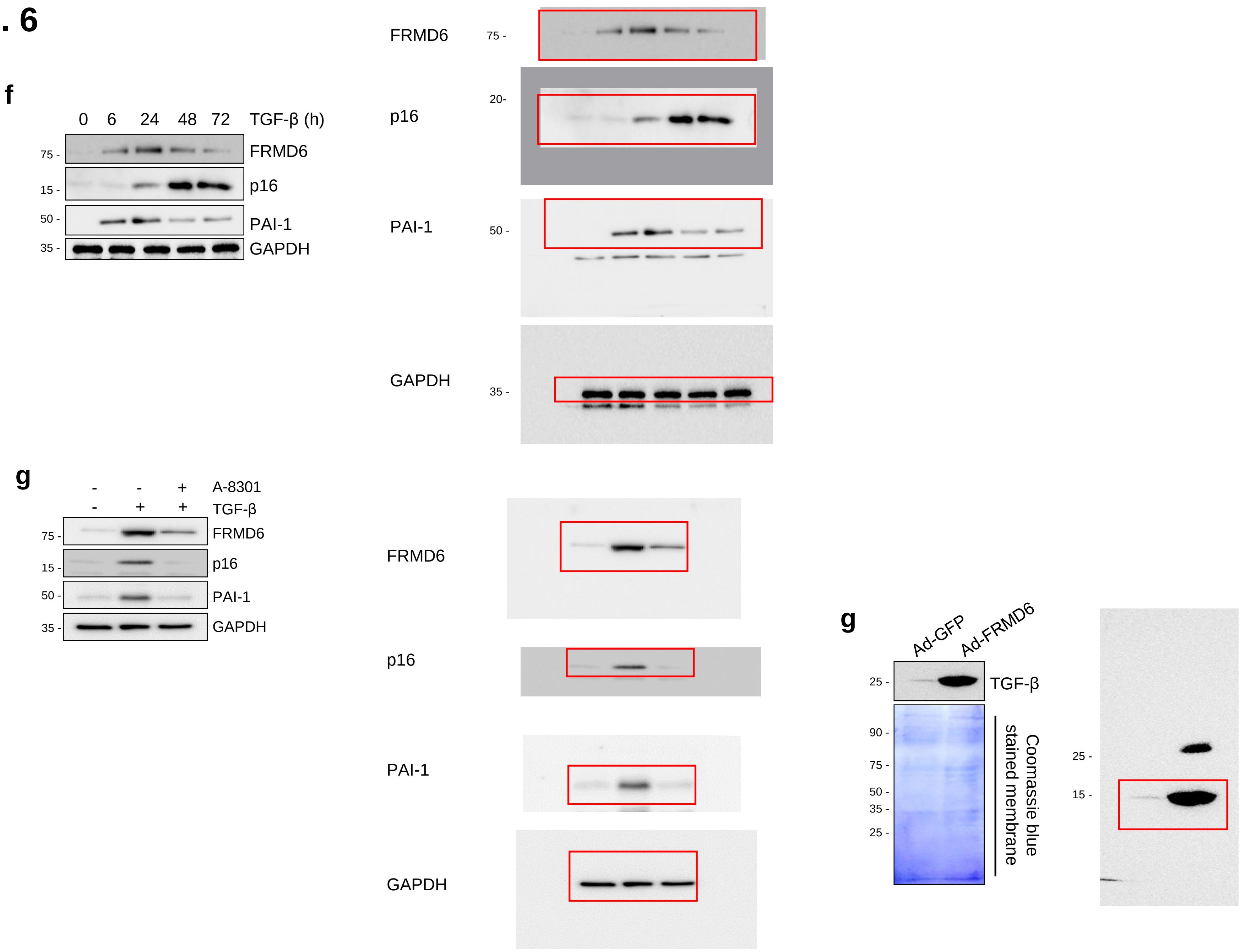
f
0
6
24
48
72
TGF-β (h)
75 -
FRMD6
15 -
p16
50 -
PAI-1
35 -
GAPDH
FRMD6
75 -
p16
20-
PAI-1
50 -
GAPDH
35 -
g
-
-
+
A-8301
-
+
+
TGF-β
75 -
FRMD6
15 -
p16
50 -
PAI-1
35 -
GAPDH
FRMD6
p16
PAI-1
GAPDH
g
Ad-GFP
Ad-FRMD6
25 -
TGF-β
90 -
75 -
50 -
35 -
25 -
Coomassie blue stained membrane
25 -
15 -

Fig. 6

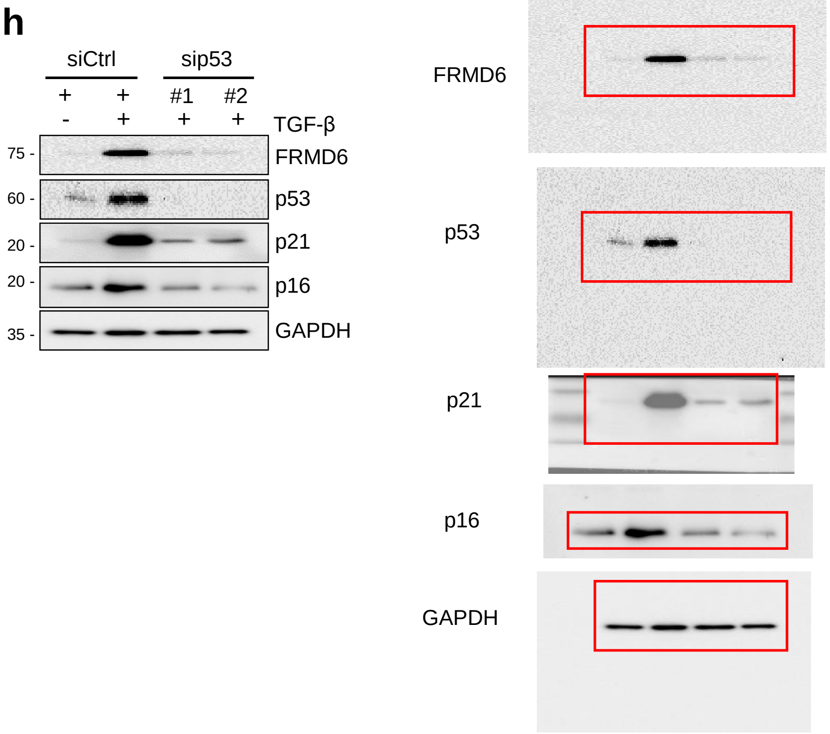
h
siCtrl
sip53
+ + #1 #2
- + + + TGF-β
75 -
FRMD6
60 -
p53
20 -
p21
20 -
p16
35 -
GAPDH
FRMD6
p53
p21
p16
GAPDH

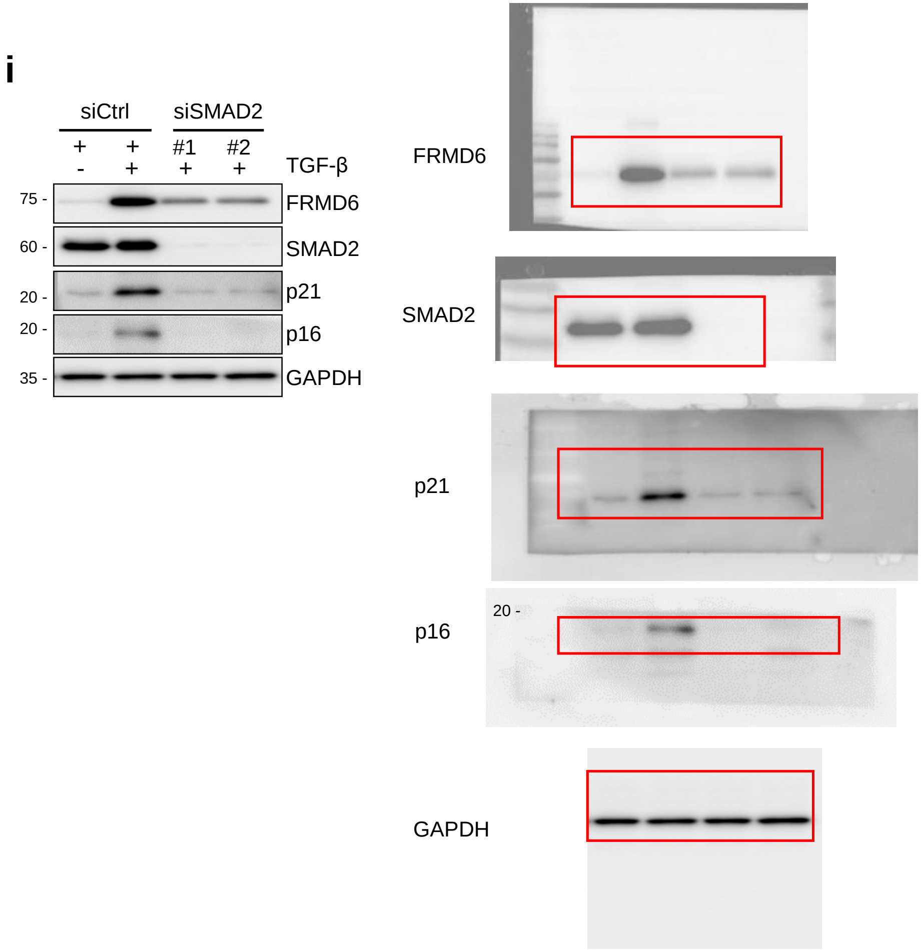
i
siCtrl
siSMAD2
+ + #1 #2
- + + + TGF-β
75 -
FRMD6
60 -
SMAD2
20 -
p21
20 -
p16
35 -
GAPDH
FRMD6
SMAD2
p21
p16
20 -
GAPDH

Fig. 7

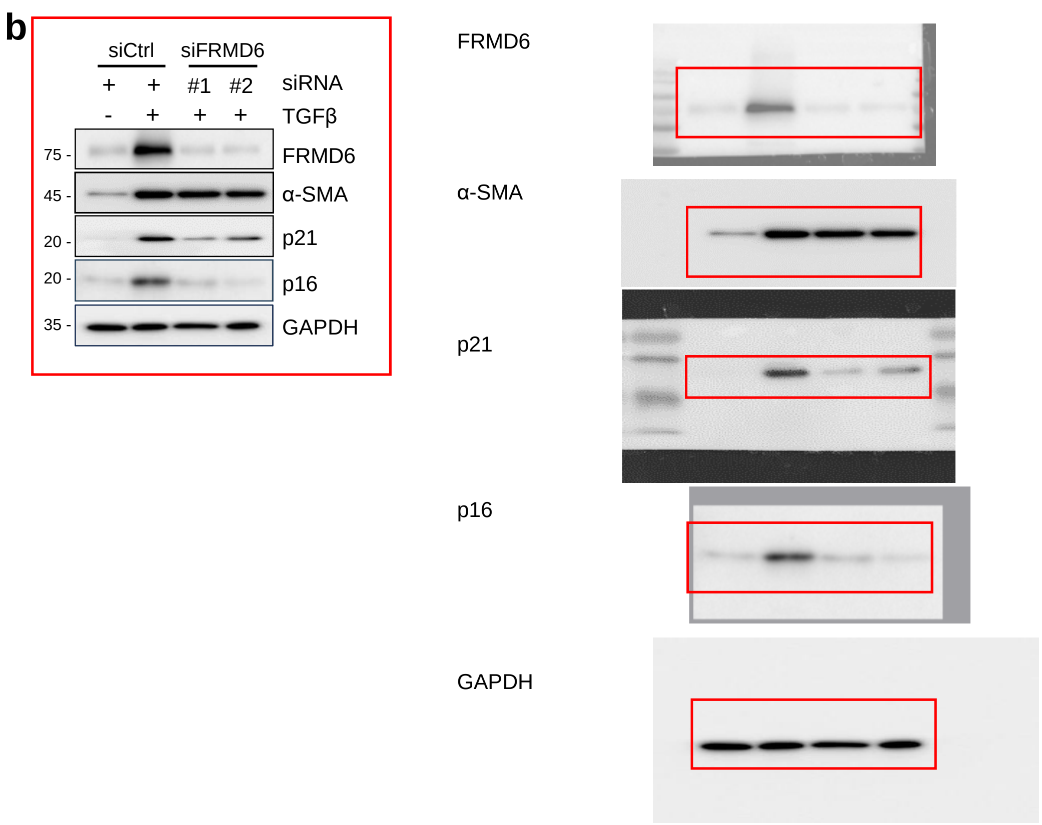
b
siCtrl
siFRMD6
+
+
#1
#2
siRNA
-
+
+
+
TGFβ
75 -
FRMD6
45 -
α-SMA
20 -
p21
20 -
p16
35 -
GAPDH
FRMD6
α-SMA
p21
p16
GAPDH

Fig. S1

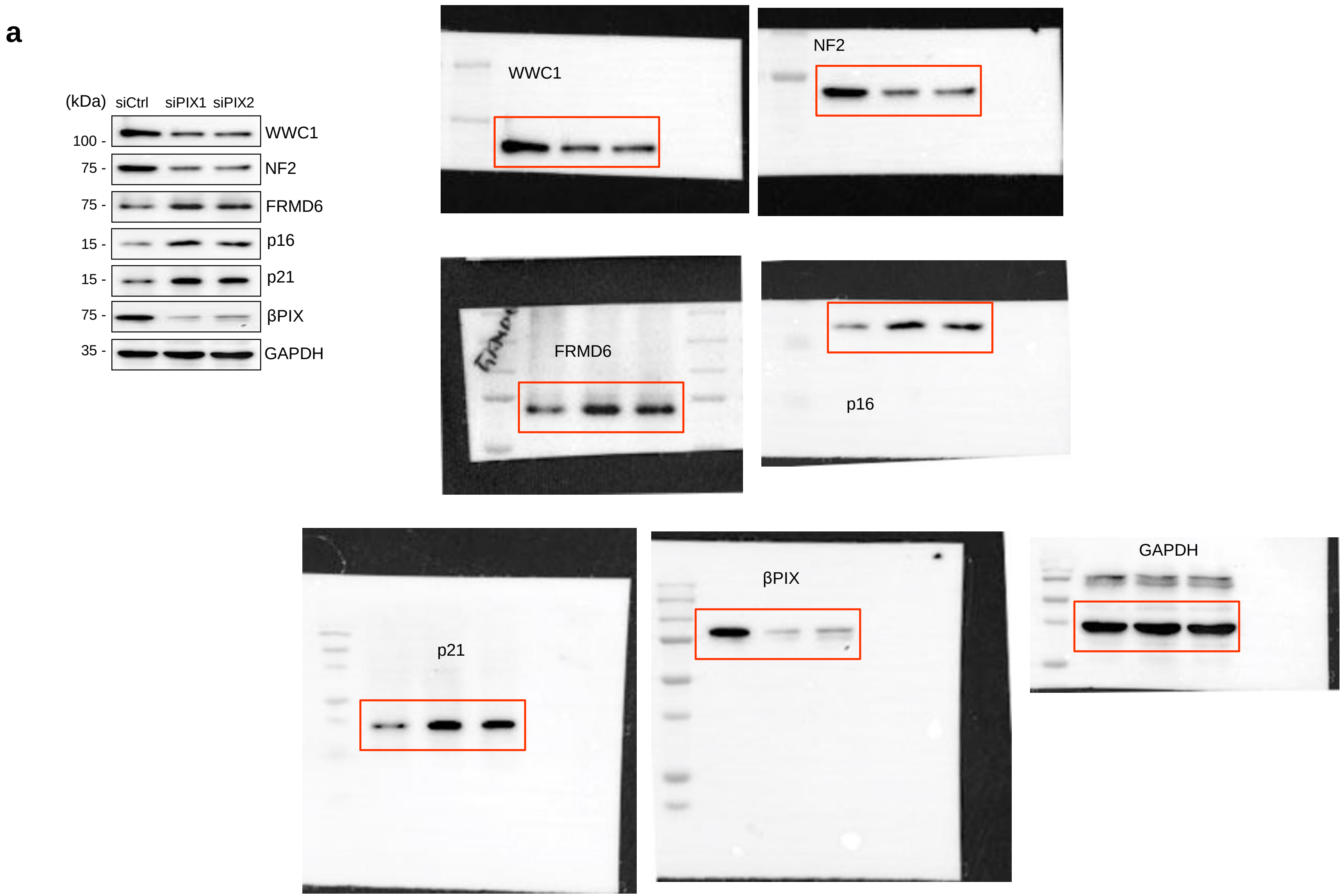
a
(kDa) siCtrl siPIX1 siPIX2
100 -
WWC1
75 -
NF2
75 -
FRMD6
15 -
p16
15 -
p21
75 -
βPIX
35 -
GAPDH
WWC1
NF2
FRMD6
p16
p21
βPIX
GAPDH

Fig. S1

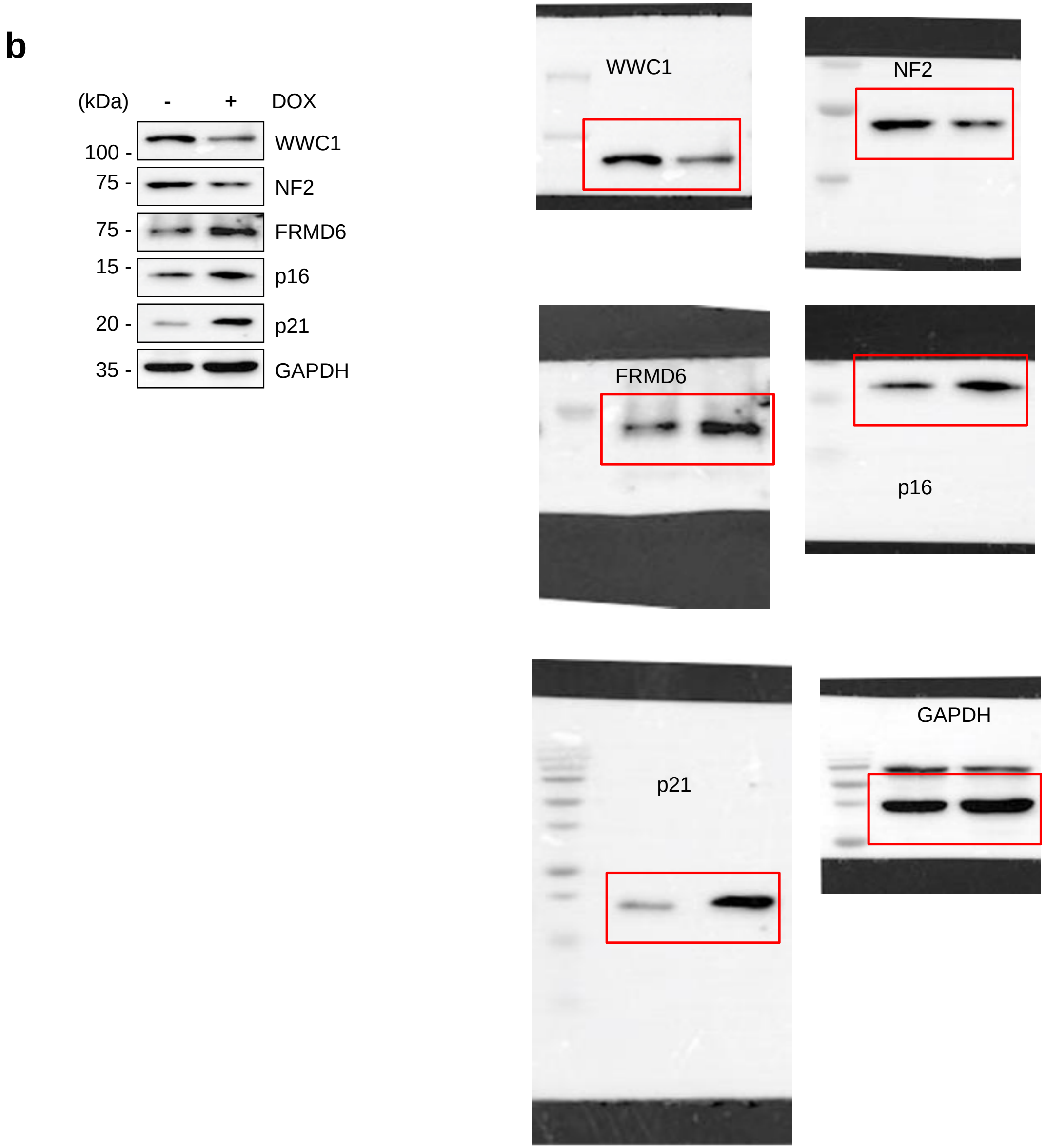
b
(kDa)
-
+
DOX
100 -
WWC1
75 -
NF2
75 -
FRMD6
15 -
p16
20 -
p21
35 -
GAPDH
WWC1
NF2
FRMD6
p16
p21
GAPDH

Fig. S1

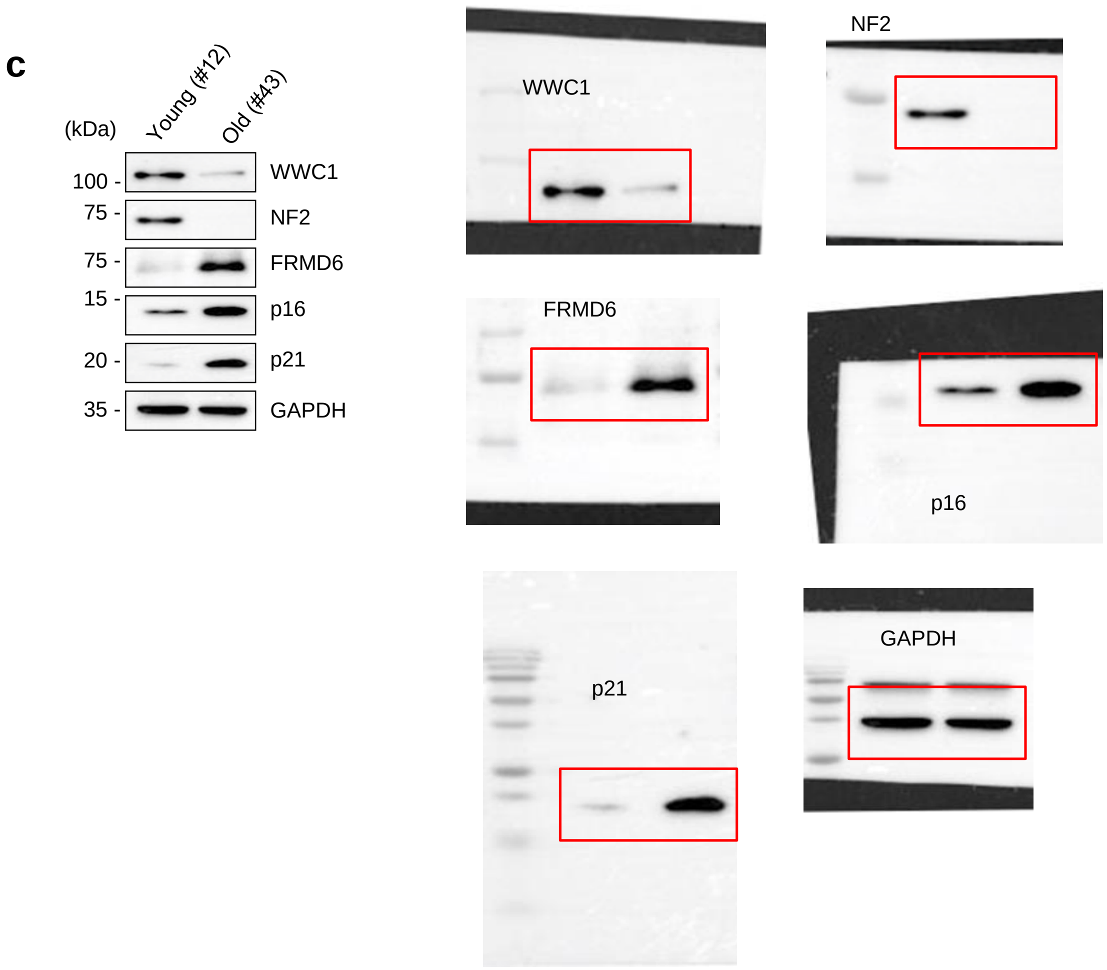
c
(kDa)
Young (#12)
Old (#43)
100 -
75 -
75 -
15 -
20 -
35 -
WWC1
NF2
FRMD6
p16
p21
GAPDH
WWC1
NF2
FRMD6
p16
p21
GAPDH

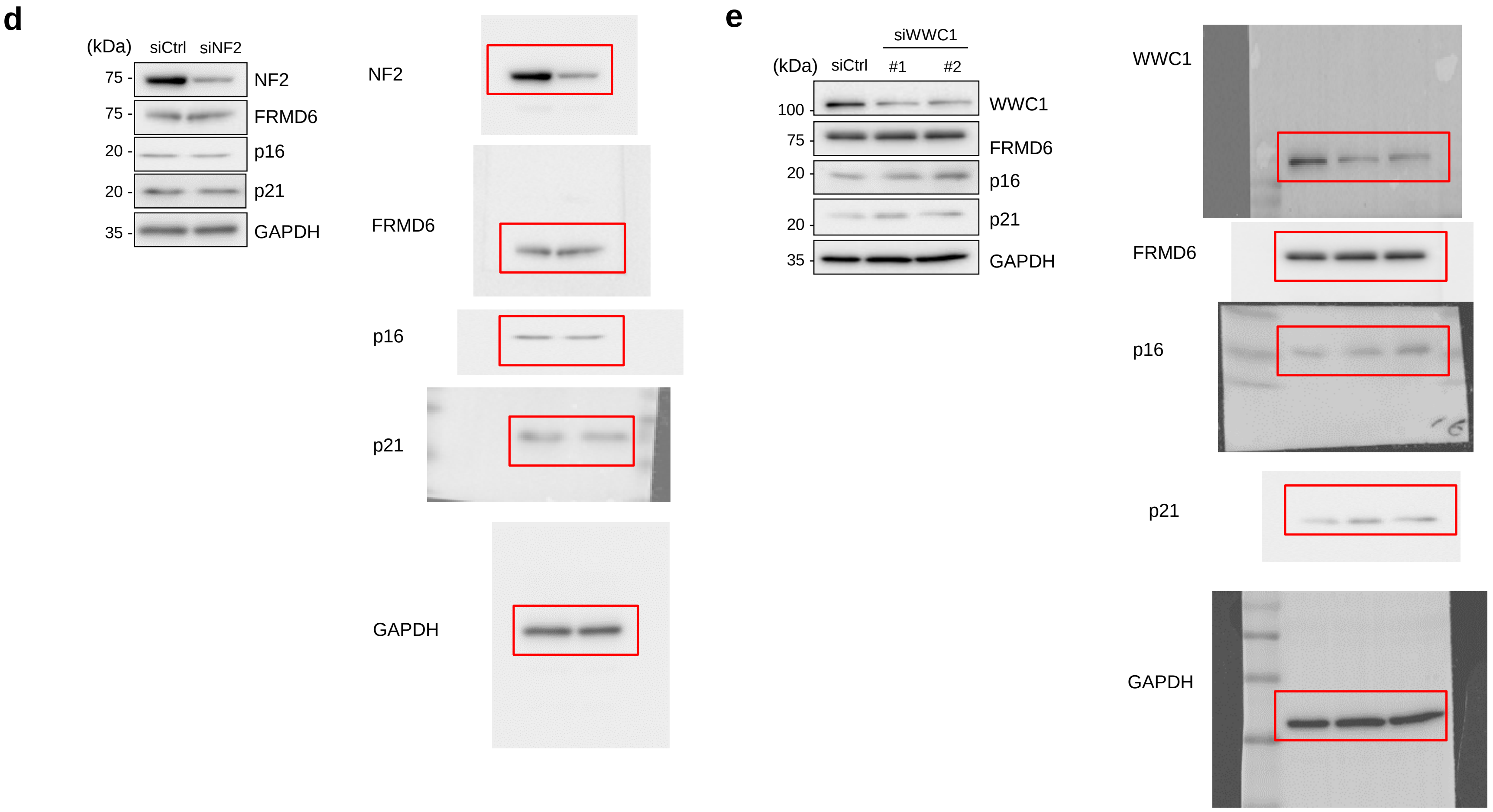

d
(kDa)
siCtrl
siNF2
75
75
20
20
35
NF2
FRMD6
p16
p21
GAPDH
NF2
FRMD6
p16
p21
GAPDH
e
siWWC1
(kDa)
siCtrl
#1
#2
100
75
20
20
35
WWC1
FRMD6
p16
p21
GAPDH
WWC1
FRMD6
p16
p21
GAPDH

Fig. S3

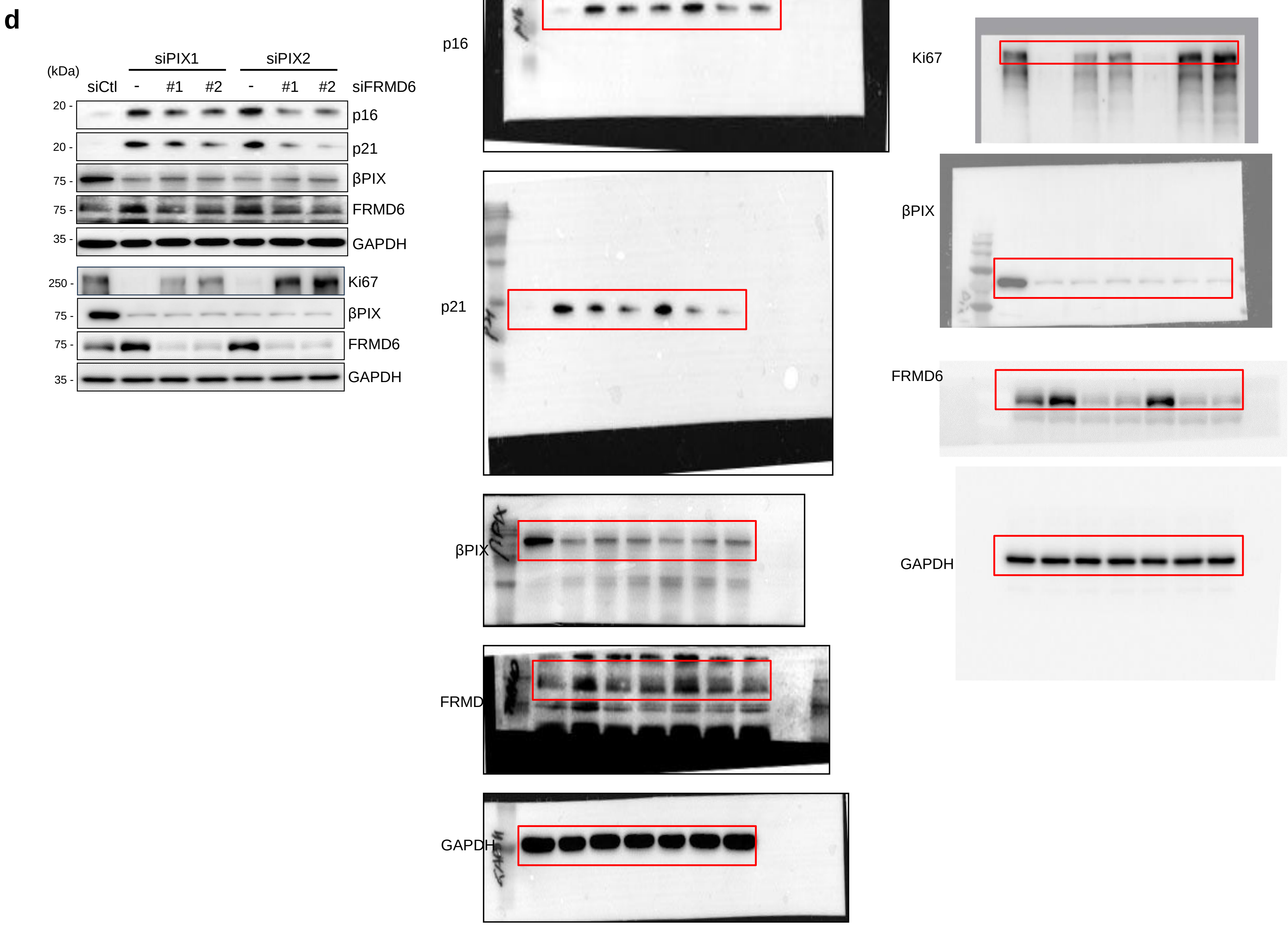

d
siPIX1
siPIX2
(kDa)
siCtl
-
#1
#2
-
#1
#2
siFRMD6
20 -
p16
20 -
p21
75 -
βPIX
75 -
FRMD6
35 -
GAPDH
250 -
Ki67
75 -
βPIX
75 -
FRMD6
35 -
GAPDH
p16
p21
βPIX
FRMD
GAPDH
Ki67
βPIX
FRMD6
GAPDH

Fig. S3

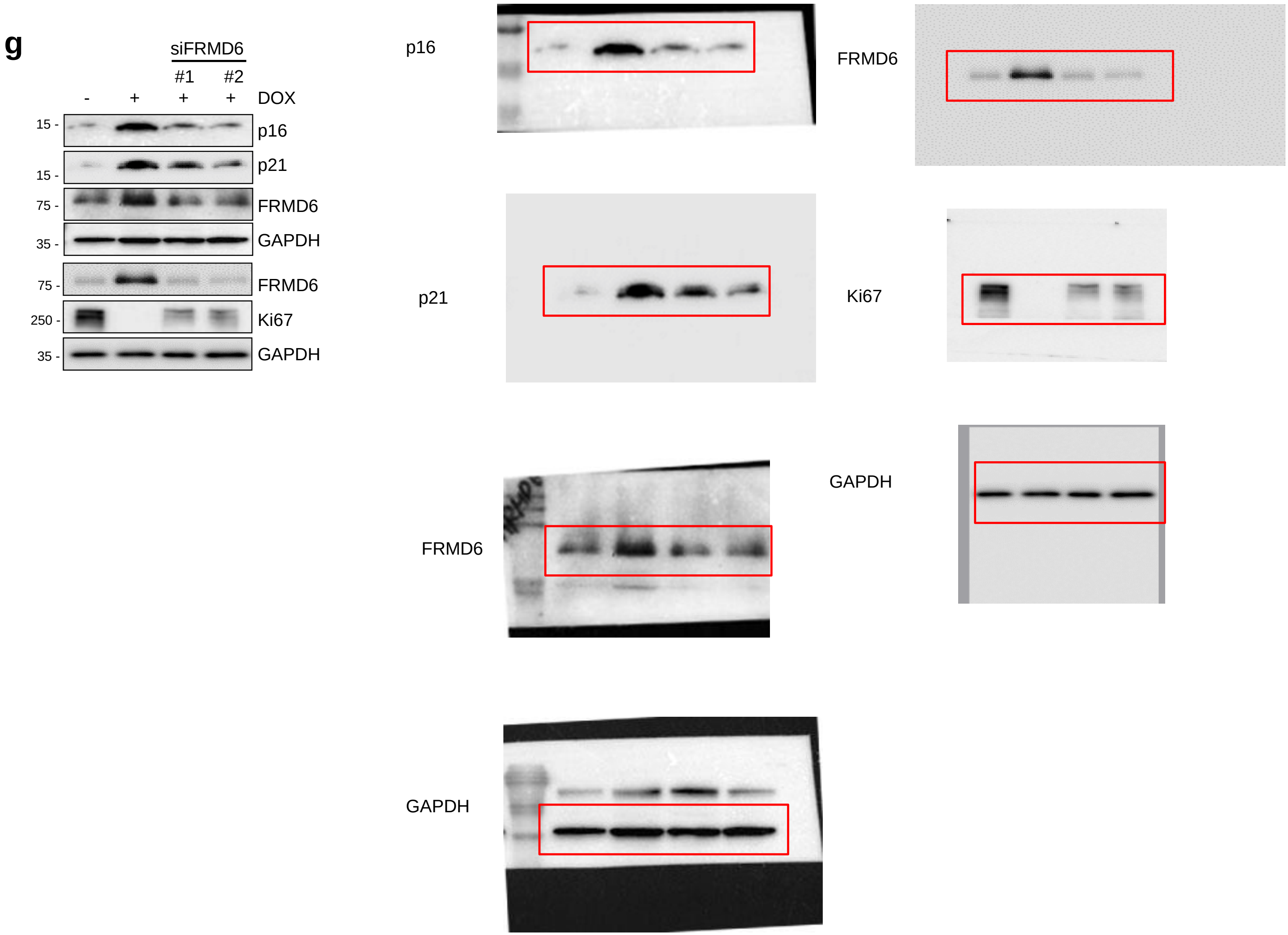
g
siFRMD6
#1
#2
-
+
+
+
DOX
15 -
p16
15 -
p21
75 -
FRMD6
35 -
GAPDH
75 -
FRMD6
250 -
Ki67
35 -
GAPDH
p16
FRMD6
p21
Ki67
FRMD6
GAPDH
GAPDH

Fig. S3

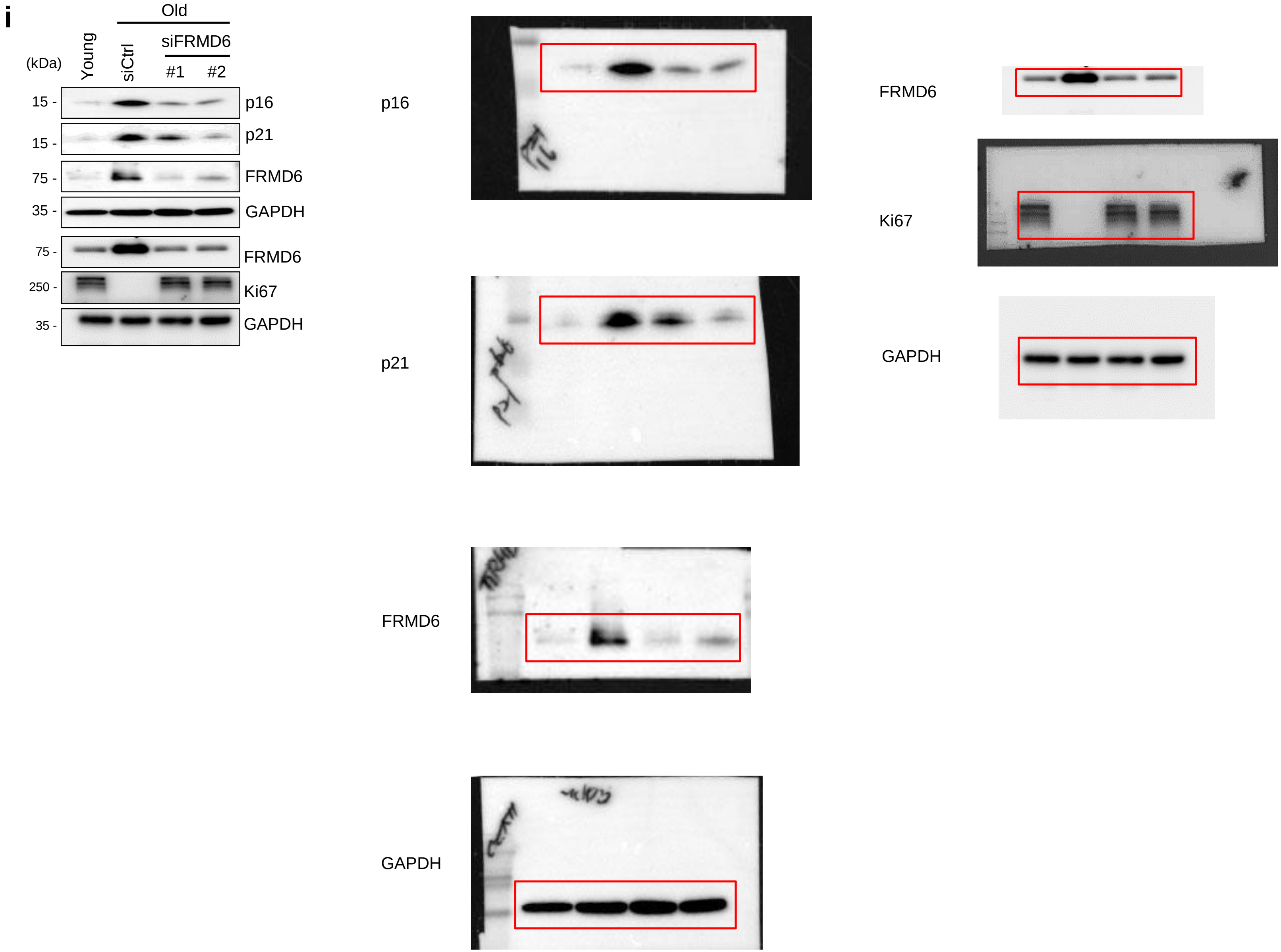
i
Old
Young
siCtrl
siFRMD6
#1
#2
(kDa)
15 -
p16
15 -
p21
75 -
FRMD6
35 -
GAPDH
75 -
FRMD6
250 -
Ki67
35 -
GAPDH
p16
p21
FRMD6
GAPDH
FRMD6
Ki67
GAPDH

Fig. S4

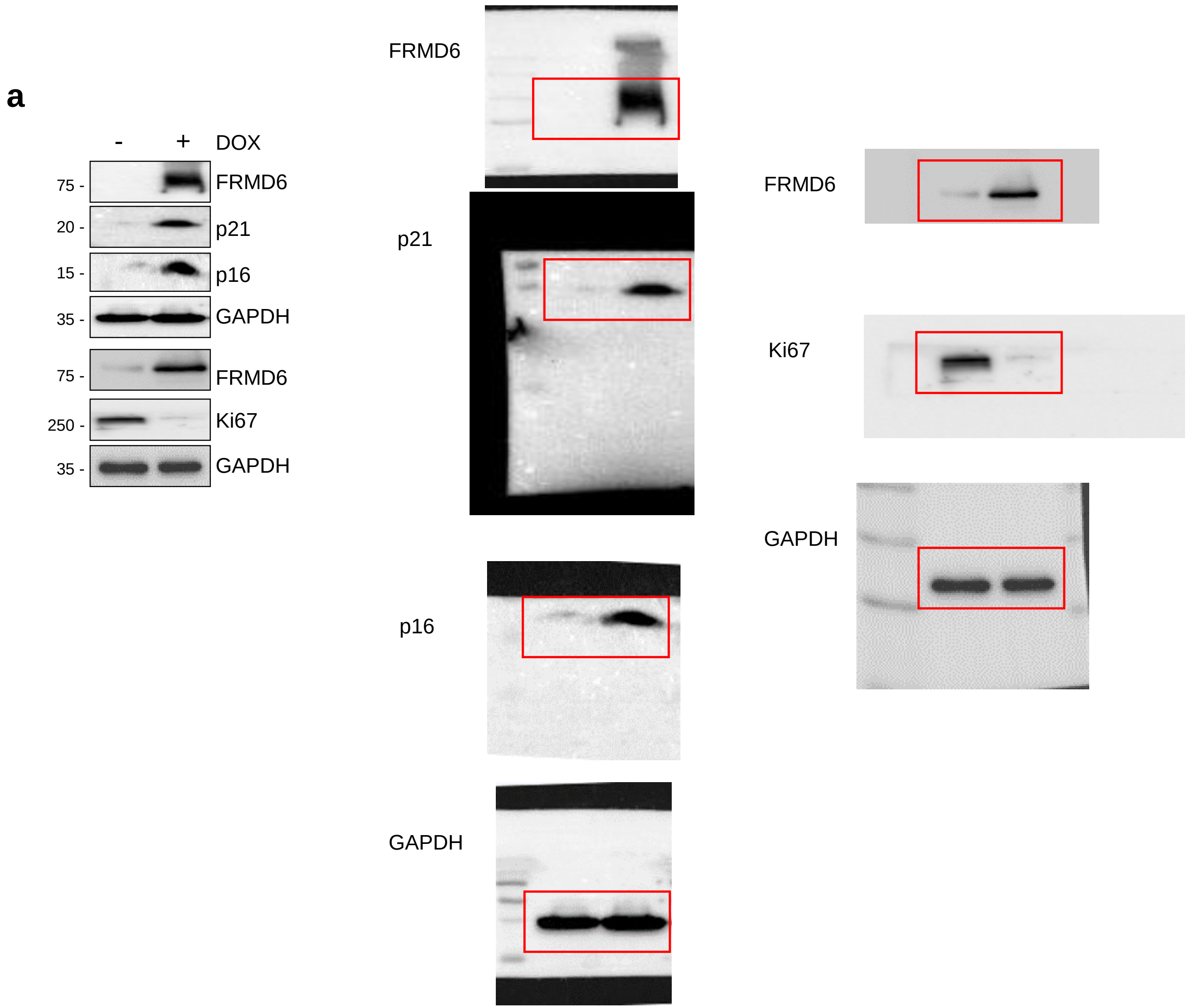

a
- + DOX
75 - FRMD6
20 - p21
15 - p16
35 - GAPDH
75 - FRMD6
250 - Ki67
35 - GAPDH
FRMD6
p21
p16
GAPDH
FRMD6
Ki67
GAPDH

Fig. S4

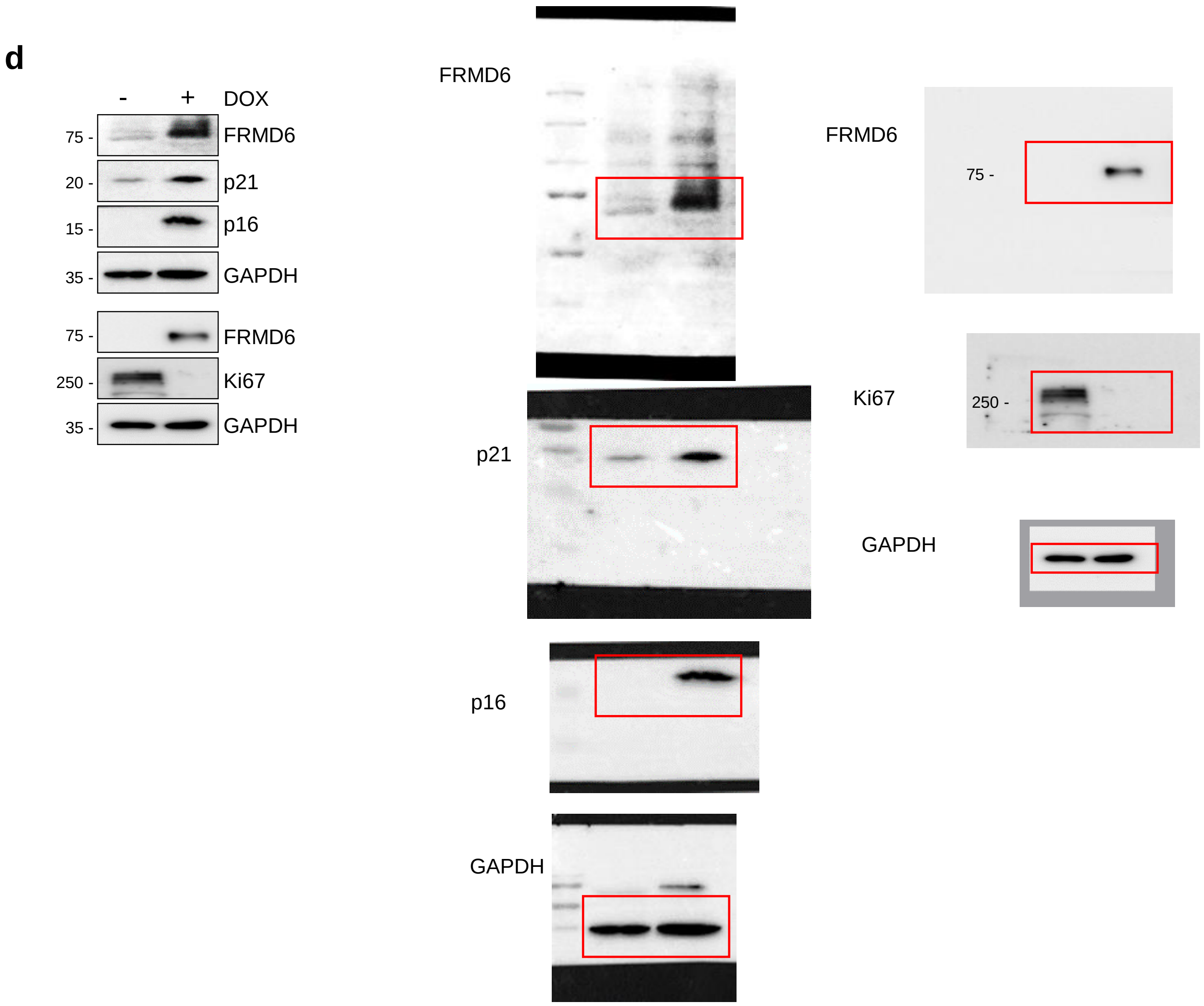
d
-
+
DOX
75 -
FRMD6
20 -
p21
15 -
p16
35 -
GAPDH
75 -
FRMD6
250 -
Ki67
35 -
GAPDH
FRMD6
p21
p16
GAPDH
FRMD6
75 -
Ki67
250 -
GAPDH

Fig. S5

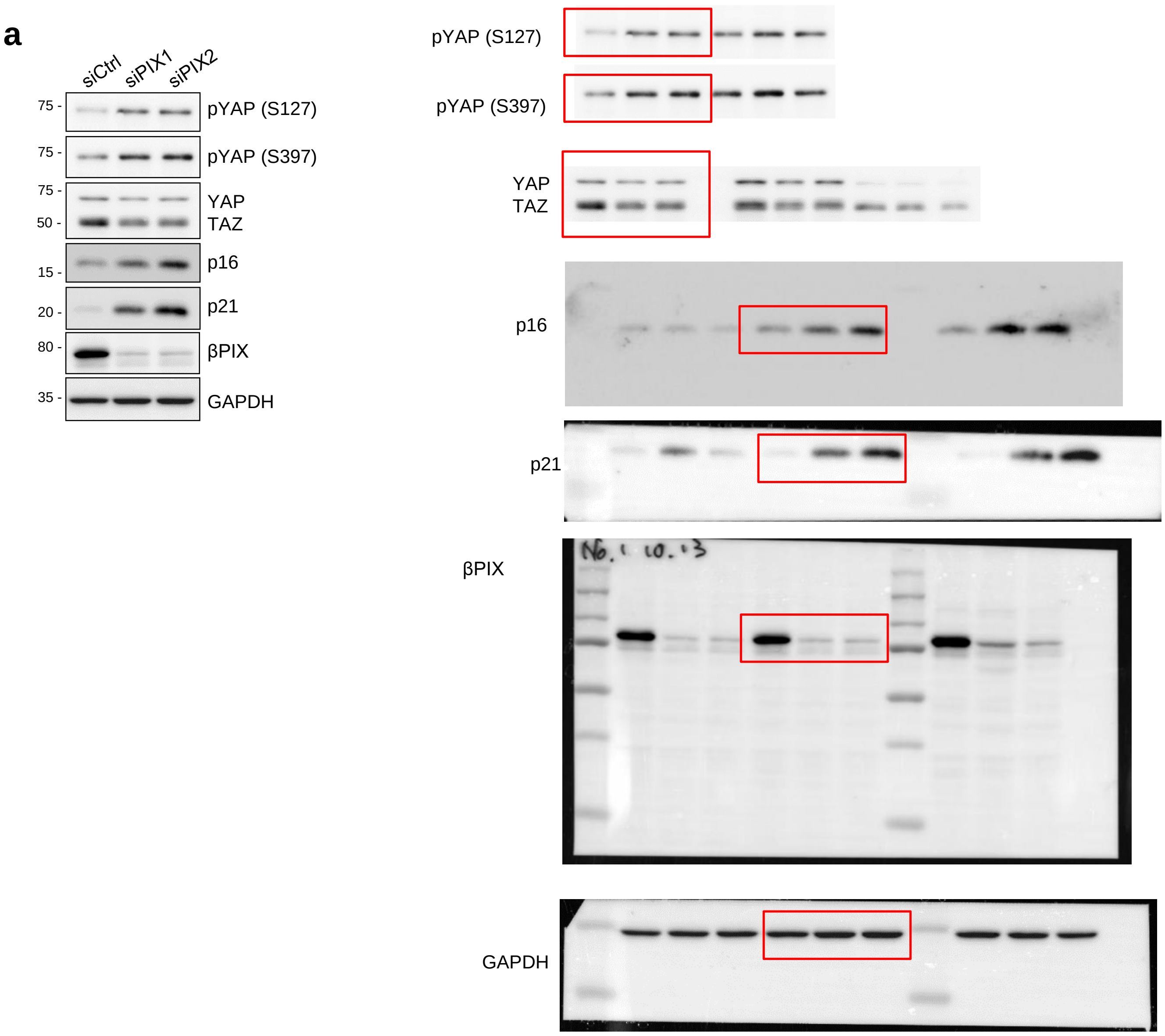

a
siCtrl
siPIX1
siPIX2
75 -
pYAP (S127)
75 -
pYAP (S397)
75 -
50 -
YAP
TAZ
15 -
p16
20 -
p21
80 -
βPIX
35 -
GAPDH
pYAP (S127)
pYAP (S397)
YAP
TAZ
p16
p21
βPIX
No. 1 10.13
GAPDH

Fig. S5

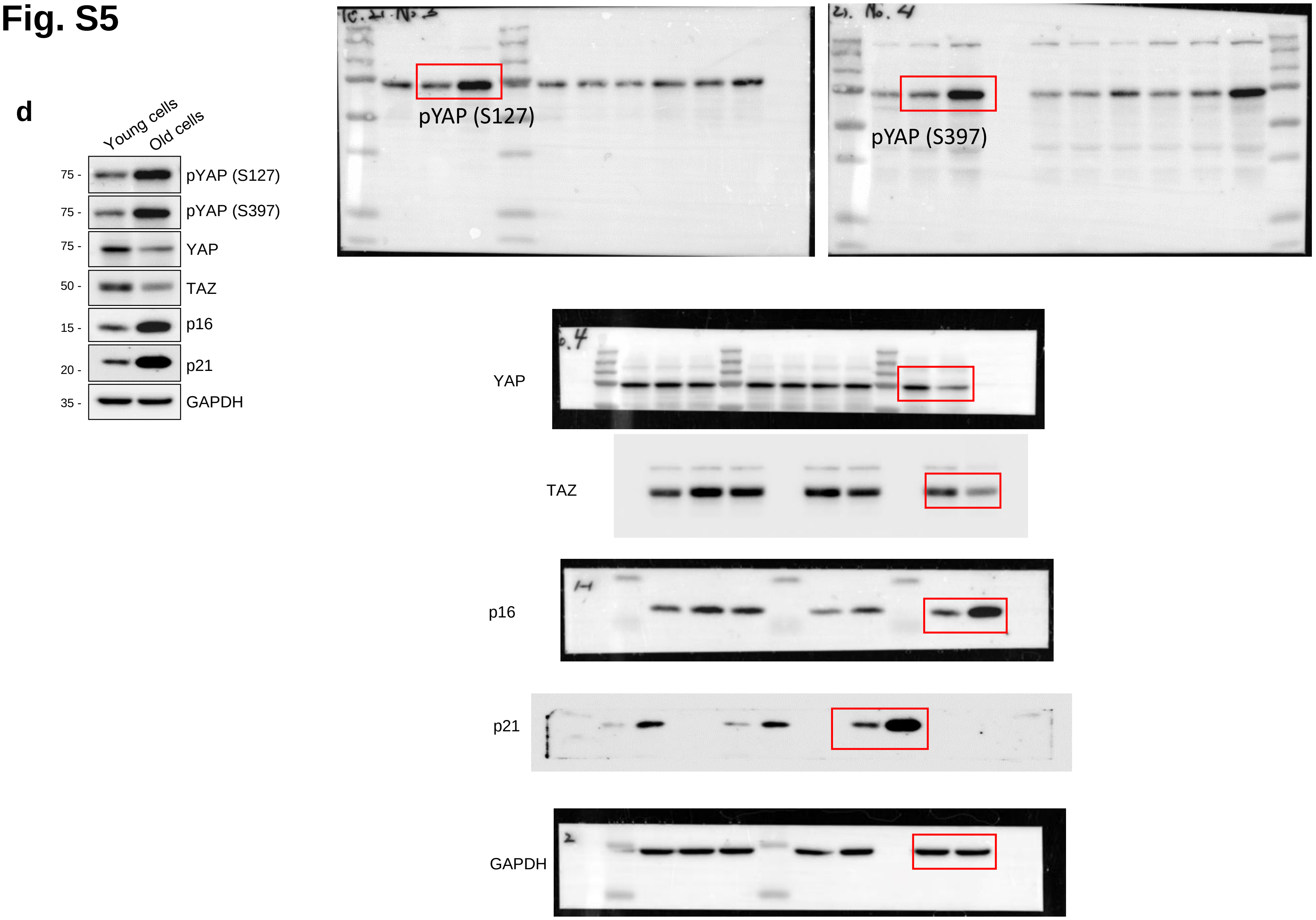
d
Young cells
Old cells
75 -
pYAP (S127)
75 -
pYAP (S397)
75 -
YAP
50 -
TAZ
15 -
p16
20 -
p21
35 -
GAPDH
pYAP (S127)
pYAP (S397)
YAP
TAZ
p16
p21
GAPDH

Fig. S5

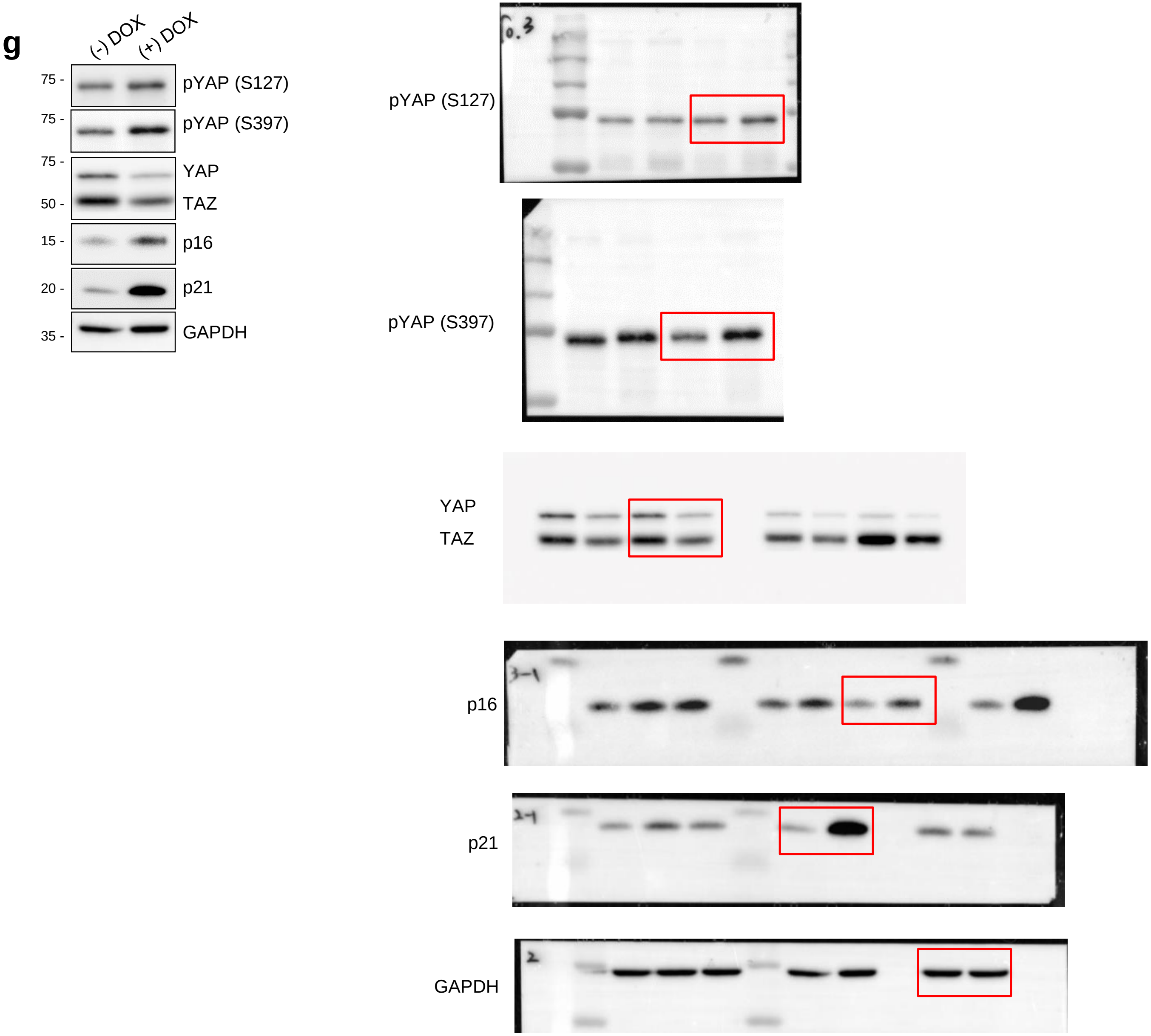
g
(-) DOX
(+) DOX
75 -
pYAP (S127)
75 -
pYAP (S397)
75 -
YAP
50 -
TAZ
15 -
p16
20 -
p21
35 -
GAPDH
pYAP (S127)
pYAP (S397)
YAP
TAZ
p16
p21
GAPDH

Fig. S6

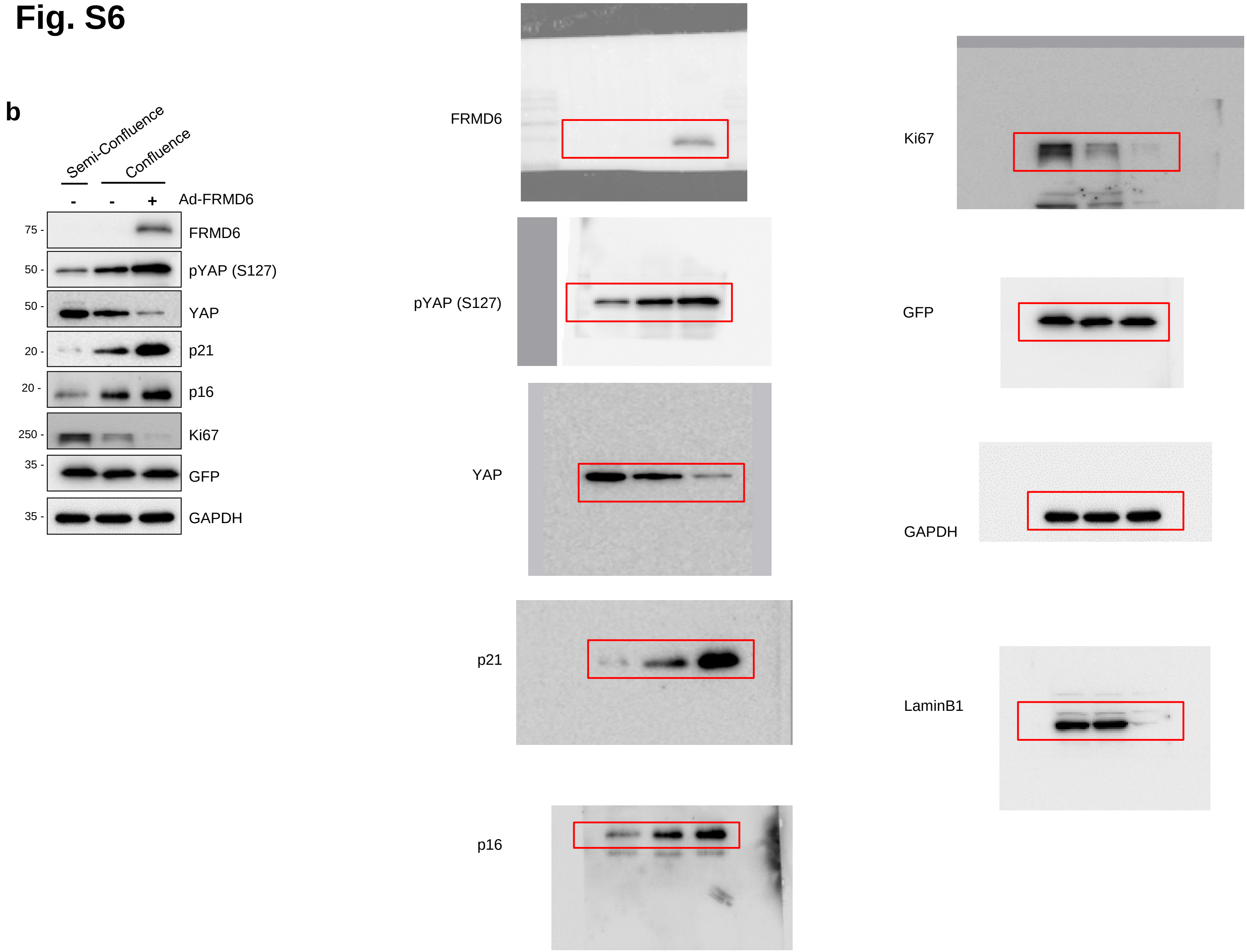
b
Semi-Confluence
Confluence
- - + Ad-FRMD6
75 - FRMD6
50 - pYAP (S127)
50 - YAP
20 - p21
20 - p16
250 - Ki67
35 - GFP
35 - GAPDH
FRMD6
pYAP (S127)
YAP
p21
p16
Ki67
GFP
GAPDH
LaminB1

Fig. S7

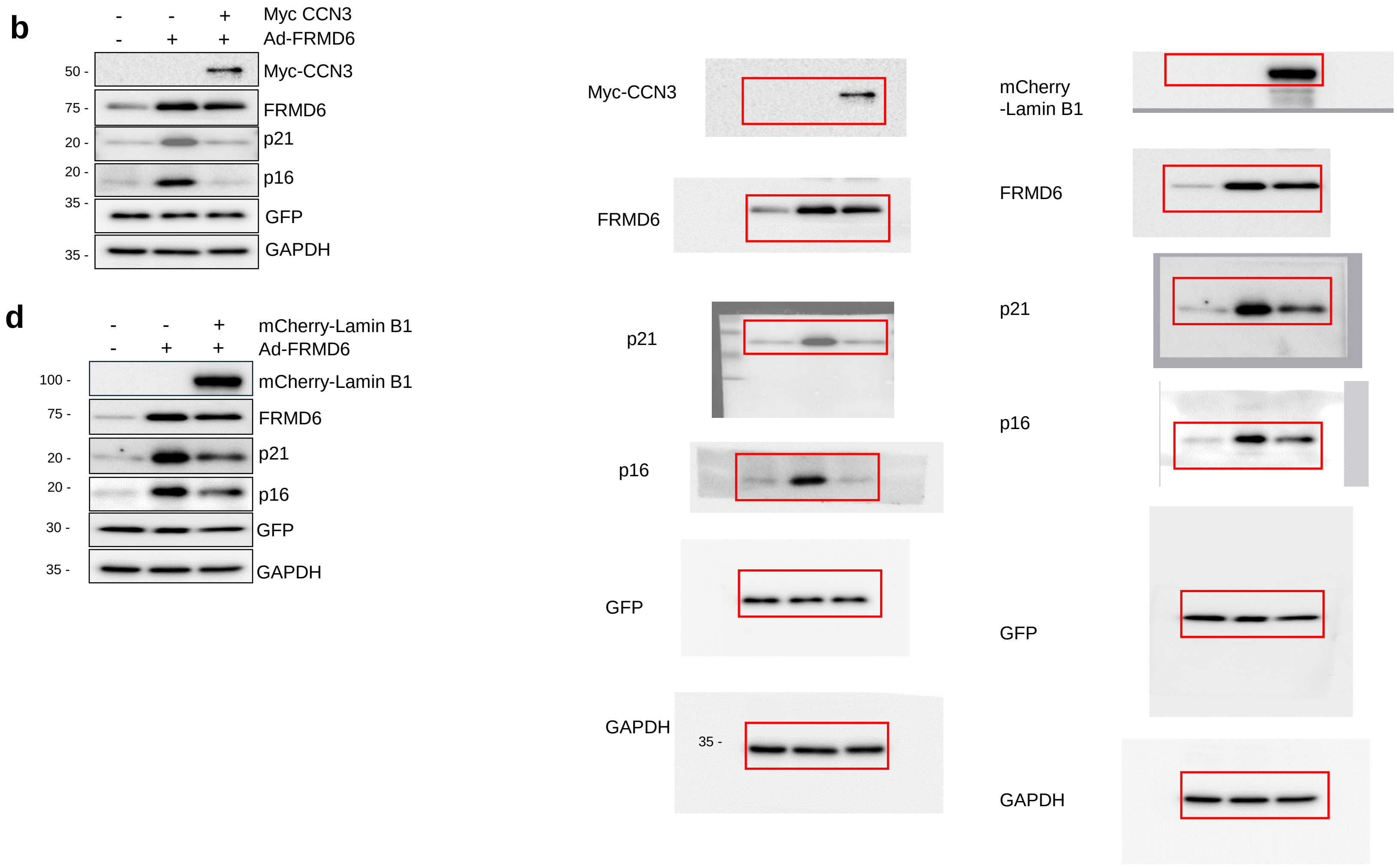
b
- - + Myc CCN3
- + + Ad-FRMD6
50 - Myc-CCN3
75 - FRMD6
20 - p21
20 - p16
35 - GFP
35 - GAPDH
d
- - + mCherry-Lamin B1
- + + Ad-FRMD6
100 - mCherry-Lamin B1
75 - FRMD6
20 - p21
20 - p16
30 - GFP
35 - GAPDH
Myc-CCN3
FRMD6
p21
p16
GFP
GAPDH
35 -
mCherry
-Lamin B1
FRMD6
p21
p16
GFP
GAPDH
